# Supplementary material for: Mass Spectrometry Imaging of In Vitro Cryptosporidium parvum-Infected Cells and Host Tissue
Source: Biomolecules. 2023 Jul 31;13(8):1200. doi: 10.3390/biom13081200 (PMC10452350; doi:10.3390/biom13081200)
Supplement: Supplementary file 1 [file biomolecules-13-01200-s001.zip › biomolecules-2476677-supplementary.pdf]

Mass Spectrometry Imaging of In Vitro *Cryptosporidium parvum*-Infected Cells and Host Tissue

Nils H. Anschütz[1], Stefanie Gerbig[1], Parviz Ghezellou[1], Liliana M. R. Silva[2], Juan Diego Velez Muñoz[2], Carlos Hermosilla[2], Anja Taubert[2], Bernhard Spengler[1,*]

[1]: Institute of Inorganic and Analytical Chemistry, Justus Liebig University Giessen, Giessen, Germany

[2]: Institute of Parasitology, Biomedical Research Center Seltersberg, Justus Liebig University Giessen, Giessen, Germany

*: Corresponding author

SUPPLEMENTARY INFORMATION Supplementary Table S1: Posthoc test HCT-8 for infected cells in positive-ion mode 1

Supplementary Table S2: Posthoc test HCT-8 for infected cells in negative-ion mode 31

Supplementary Table S3: Posthoc test HCT-8 for control cells in positive-ion mode 60

Supplementary Table S4: Posthoc test HCT-8 for control cells in negative-ion mode 96

Supplementary Table S5: Annotations for upregulated ion signals in positive-ion mode 132

Supplementary Table S6: Annotations for upregulated ion signals in negative-ion mode 218

Supplementary Table S7: Annotations for downregulated ion signals in positive-ion mode 298

Supplementary Table S8: Annotations for downregulated ion signals in negative-ion mode 360

Supplementary Table S9: LipidMatch Flow settings 388

Supplementary Table S10: Identified markers for upregulated ion signals in positive-ion mode 388

Supplementary Table S11: Identified markers for downregulated ion signals in positive-ion mode 389

Supplementary Table S12: Identified markers for upregulated ion signals in negative-ion mode 390

Supplementary Table S13: Identified markers for downregulated ion signals in negative-ion mode 390

Supplementary Table S14: Comparison of identified infection markers with literature[26.27] 391

Supplementary Table S1: Posthoc test HCT-8 for infected cells in positive-ion mode

Control	Infected	C: ANOVA Significant	N: -Log ANOVA p value	N: ANOVA q-value	MALDI m/z
		+	222.952	0.0264185	305.1581210
-222.943	222.943				
-189.611	189.611	+	189.614	0.0453985	307.1374261
-260.874	260.874	+	260.902	0.0189657	308.1329555
		+	473.081	0	318.1785405

-461.878	461.878				
-281.138	281.138	+	28.119	0.0150965	319.1374607
		+	218.322	0.0285282	319.1738783
-218.314	218.314				
-229.638	229.638	+	229.649	0.0247199	321.1642990
		+	301.183	0.0136566	321.1894564
-301.089	301.089				
-280.308	280.308	+	280.358	0.0152017	322.1479899
		+	243.808	0.0214391	323.1325226
-243.792	243.792				
-385.228	385.228	+	386.721	0.00833333	325.3211755
		+	232.729	0.0234052	329.0822886
-232.717	232.717				
-206.767	206.767	+	206.773	0.0348555	331.1373706
		+	293.939	0.0142824	332.1942686
-293.863	293.863				
-233.708	233.708	+	233.721	0.0238036	333.1531016
		+	28.617	0.014598	334.1481652
-28.611	28.611				
-291.945	291.945	+	292.016	0.0151299	336.1264686
		+	318.686	0.0149223	336.1640500
-318.525	318.525				
-244.248	244.248	+	244.265	0.0214554	342.0711476
		+	524.116	0	343.3317952
-524.177	524.177				
-227.169	227.169	+	227.179	0.0250804	344.1218463
		+	231.791	0.0236745	344.3351246
-23.178	23.178				
-201.473	201.473	+	201.478	0.0379728	345.1531976
		+	236.041	0.0231429	346.1482559
-236.028	236.028				
-259.272	259.272	+	259.299	0.0183772	347.1687687
		+	299.951	0.0135817	347.2051247
-29.986	29.986				
-242.328	242.328	+	242.344	0.0219892	348.1640942
		+	224.305	0.026381	349.1480156
-224.296	224.296				
-234.408	234.408	+	234.421	0.023524	350.1433888

		+	253.989	0.018749	350.1791782
-253.966	253.966				
-215.494	215.494	+	215.501	0.0304198	353.3196232
		+	215.238	0.0306987	353.3203860
-215.231	215.231				
-201.511	201.511	+	201.516	0.0379927	356.0852469
		+	288.898	0.0145827	359.1686511
-288.833	288.833				
-251.928	251.928	+	251.949	0.0187254	360.1640399
		+	271.471	0.0167426	361.1843408
-271.433	271.433				
-186.128	186.128	+	186.131	0.0489769	361.2735192
		+	216.794	0.029686	362.1433136
-216.786	216.786				
-293.621	293.621	+	293.696	0.014449	362.1794275
		+	290.686	0.014594	363.1636839
-290.618	290.618				
-243.089	243.089	+	243.106	0.0219845	364.0534685
		+	257.538	0.0185455	364.1588324
-257.512	257.512				
-251.527	251.527	+	251.549	0.0189179	364.1953474
		+	376.937	0.00909091	365.3142346
-375.873	375.873				
-238.103	238.103	+	238.118	0.0223455	366.1384877
		+	253.035	0.0186755	368.3435833
-253.013	253.013				
-214.405	214.405	+	214.412	0.0310211	369.3472729
		+	21.785	0.0290184	370.3548077
-217.842	217.842				
-311.626	311.626	+	311.756	0.0141485	371.3581572
		+	186.911	0.0479491	372.1529331
-186.908	186.908				
-222.602	222.602	+	222.611	0.026459	373.1843581
		+	253.855	0.0186988	374.1783753
-253.832	253.832				
-234.125	234.125	+	234.137	0.0236804	375.1995820
		+	238.986	0.0224276	375.2003728

-238.971	238.971				
-258.623	258.623	+	258.649	0.0181466	376.1587361
		+	2.584	0.0180672	376.1953814
-258.374	258.374				
-277.877	277.877	+	277.924	0.0154757	377.1791788
		+	186.333	0.0486886	378.0332175
-18.633	18.633				
-192.595	192.595	+	192.599	0.042828	378.1746463
		+	209.084	0.0337869	378.2114299
-209.078	209.078				
-285.298	285.298	+	285.357	0.0143173	379.1585383
		+	185.497	0.049413	380.9122341
-185.494	185.494				
-238.529	238.529	+	238.543	0.0222914	382.1819579
		+	249.467	0.0190074	382.3790119
-249.447	249.447				
-231.843	231.843	+	231.854	0.0237368	383.3823424
		+	187.562	0.0472444	387.1991509
-187.559	187.559				
-212.144	212.144	+	21.215	0.0313464	387.2010035
		+	184.805	0.049872	388.1213534
-184.802	184.802				
-275.708	275.708	+	275.752	0.0157812	388.1585608
		+	194.805	0.0414998	388.1951821
-194.801	194.801				
-281.267	281.267	+	281.319	0.015163	389.2156576
		+	277.515	0.0154262	390.1648975
-277.469	277.469				
-320.626	320.626	+	320.798	0.0150056	390.1746250
		+	336.048	0.00892086	390.2111229
-335.772	335.772				
-282.141	282.141	+	282.194	0.0152528	391.1949168
		+	259.909	0.018742	392.1895046
-259.882	259.882				
-259.411	259.411	+	259.438	0.0184324	392.1904242
		+	277.224	0.0155372	392.2267104
-277.178	277.178				
-222.856	222.856	+	222.865	0.0263554	393.1865842

-27.223	27.223	+	272.269	0.0167747	402.1745898
-28.284	28.284	+	282.894	0.0151632	402.2110343
		+	235.518	0.0233742	402.8940685
-235.504	235.504				
-202.363	202.363	+	202.368	0.0371332	403.1947850
		+	227.519	0.0252427	404.1904323
-227.509	227.509				
-18.629	18.629	+	186.293	0.0487943	404.2019914
		+	260.248	0.0190031	404.2265147
-26.022	26.022				
-220.345	220.345	+	220.354	0.0277112	405.1857930
		+	216.945	0.0296589	406.2424444
-216.937	216.937				
-364.267	364.267	+	364.975	0.0101143	407.2025922
		+	276.488	0.0159838	408.1851531
-276.443	276.443				
-226.164	226.164	+	226.174	0.0256254	413.1788664
		+	302.895	0.0130871	416.1912626
-302.796	302.796				
-234.473	234.473	+	234.486	0.0234534	418.2057537
		+	285.349	0.0142488	418.2426266
-285.291	285.291				
-211.838	211.838	+	211.845	0.0315488	419.2014600
		+	281.509	0.0153318	420.1851121
-281.457	281.457				
-215.993	215.993	+	2.16	0.0303362	420.2214937
		+	232.112	0.0236222	421.1787760
-232.101	232.101				
-199.916	199.916	+	19.992	0.0387843	421.1816213
		+	2.009	0.0383983	424.8759642
-200.895	200.895				
-2.003	2.003	+	200.304	0.0385968	425.0723509
		+	300.864	0.0139463	426.2373468
-30.077	30.077				
-234.787	234.787	+	2.348	0.0234694	428.1885862
		+	23.407	0.0237287	428.1914504

-234.058	234.058				
-328.014	328.014	+	328.231	0.0126329	429.1741833
		+	257.187	0.0184126	430.2056849
-257.162	257.162				
-280.324	280.324	+	280.374	0.0152348	433.2055549
		+	268.464	0.017296	433.2165985
-268.429	268.429				
-28.949	28.949	+	289.556	0.0148556	434.2008199
		+	184.944	0.0498567	434.2369750
-184.941	184.941				
-338.031	338.031	+	338.329	0.0096124	435.2327218
		+	195.639	0.0410342	439.2030227
-195.635	195.635				
-347.366	347.366	+	347.768	0.00996078	444.2215666
		+	259.639	0.0185719	445.2165350
-259.612	259.612				
-222.058	222.058	+	222.067	0.0267243	446.2012658
		+	276.317	0.0158551	446.2368759
-276.273	276.273				
-22.851	22.851	+	228.521	0.0246525	447.1938730
		+	266.761	0.0179691	447.2325220
-266.728	266.728				
-239.236	239.236	+	23.925	0.0221636	449.2117487
		+	403.384	0.00555556	449.2478405
-400.738	400.738				
-237.117	237.117	+	237.131	0.022786	450.2320295
		+	303.676	0.0132721	456.1049847
-303.574	303.574				
-262.266	262.266	+	262.295	0.0190321	457.1082308
		+	239.053	0.0224171	457.2321743
-239.039	239.039				
-227.063	227.063	+	227.073	0.02502	458.2271266
		+	26.171	0.0191379	458.2357052
-261.682	261.682				
-337.315	337.315	+	337.606	0.00932331	461.2116482
		+	411.087	0.00645161	461.2474443
-407.684	407.684				
-223.366	223.366	+	223.375	0.0264571	462.2320582

		+	191.589	0.0438768	463.1913219
-191.586	191.586				
-211.769	211.769	+	211.775	0.0315104	463.2265060
		+	228.065	0.0250415	463.2638092
-228.055	228.055				
-309.152	309.152	+	309.272	0.0133884	471.2476332
		+	247.872	0.0202262	472.2520657
-247.853	247.853				
-192.206	192.206	+	192.209	0.0436137	473.2122307
		+	201.452	0.0379655	474.2321724
-201.448	201.448				
-222.197	222.197	+	222.206	0.0266559	474.2675295
		+	30.173	0.0135017	475.3049532
-301.634	301.634				
-323.437	323.437	+	323.625	0.0143626	476.3082546
		+	193.707	0.0420272	478.0457243
-193.704	193.704				
-306.714	306.714	+	306.826	0.0130469	478.0868742
		+	277.327	0.0155694	478.2377271
-277.281	277.281				
-28.438	28.438	+	284.437	0.0144507	479.0897629
		+	285.371	0.0143518	479.0905220
-285.312	285.312				
-23.285	23.285	+	232.862	0.0234679	479.2223119
		+	215.741	0.0303127	480.0925936
-215.734	215.734				
-276.018	276.018	+	276.062	0.0156349	481.2292840
		+	239.985	0.0221042	481.2318368
-23.997	23.997				
-368.016	368.016	+	368.822	0.00819672	483.2476980
		+	202.991	0.0367974	484.2161067
-202.986	202.986				
-309.515	309.515	+	309.637	0.0135	484.2518131
		+	256.369	0.0187705	485.2555618
-256.345	256.345				
-248.841	248.841	+	24.886	0.0190889	485.2634049
		+	211.338	0.0317404	485.3114294

-211.331	211.331				
-235.631	235.631	+	235.644	0.0233175	486.1952374
		+	193.071	0.0425237	486.2582157
-193.067	193.067				
-258.155	258.155	+	258.181	0.0184163	486.2668272
		+	264.828	0.018897	486.3143866
-264.797	264.797				
-337.253	337.253	+	337.543	0.00925373	487.2262030
		+	223.132	0.0263581	489.2427566
-223.123	223.123				
-203.256	203.256	+	203.261	0.0366556	490.2270477
		+	233.638	0.0237174	491.2223642
-233.625	233.625				
-22.884	22.884	+	228.851	0.0246832	491.2582624
		+	185.132	0.0496132	492.2543553
-18.513	18.513				
-206.504	206.504	+	20.651	0.0348433	492.3793266
		+	211.367	0.0317183	492.3808804
-211.361	211.361				
-23.242	23.242	+	232.432	0.0234433	496.3286006
		+	283.634	0.0153039	497.2873459
-283.578	283.578				
-19.394	19.394	+	193.944	0.0418209	500.0680295
		+	193.533	0.0421009	503.5011314
-193.529	193.529				
-245.974	245.974	+	245.991	0.0207459	504.3057323
		+	337.917	0.00939394	505.2331022
-337.623	337.623				
-233.978	233.978	+	23.399	0.023642	505.3671694
		+	205.697	0.03518	506.2345142
-205.692	205.692				
-262.435	262.435	+	262.464	0.0189021	506.3706000
		+	204.012	0.0364276	507.2473952
-204.007	204.007				
-209.991	209.991	+	209.997	0.0332224	508.2479884
		+	255.301	0.0189141	508.3392286
-255.278	255.278				
-255.664	255.664	+	255.688	0.0189106	508.3407506

		+	23.837	0.02228	509.2653969
-238.355	238.355				
-281.809	281.809	+	281.862	0.0150811	509.3427213
		+	217.669	0.0291506	516.0649178
-217.661	217.661				
-233.361	233.361	+	233.373	0.0236105	516.3055851
		+	209.001	0.0338248	517.2738674
-208.995	208.995				
-24.286	24.286	+	242.876	0.0219978	517.9494457
		+	224.846	0.0259566	518.0792498
-224.836	224.836				
-235.674	235.674	+	235.687	0.0233397	518.3123381
		+	298.327	0.0144635	519.0830303
-298.241	298.241				
-256.399	256.399	+	256.424	0.0187972	520.3392168
		+	256.867	0.0188155	520.3411708
-256.843	256.843				
-285.602	285.602	+	285.661	0.0144563	521.3426250
		+	236.866	0.0231589	522.3549188
-236.853	236.853				
-19.163	19.163	+	191.634	0.0438061	522.5239147
		+	233.532	0.0236959	523.2581710
-233.519	233.519				
-211.287	211.287	+	211.293	0.0317867	524.1368702
		+	295.832	0.0147152	524.3587187
-295.752	295.752				
-260.628	260.628	+	260.656	0.0189362	524.3625957
		+	392.308	0.00666667	525.3637028
-390.495	390.495				
-217.823	217.823	+	217.831	0.0290389	526.2885253
		+	19.791	0.0398968	527.2578473
-197.906	197.906				
-27.189	27.189	+	271.929	0.0166815	530.3211705
		+	272.515	0.0166194	531.2918413
-272.476	272.476				
-29.038	29.038	+	290.448	0.0145149	531.3246242
		+	24.066	0.0217395	532.2480776

-240.644	240.644				
-212.408	212.408	+	212.414	0.031328	532.3368805
		+	27.522	0.0160619	533.3052728
-275.177	275.177				
-313.808	313.808	+	313.947	0.0147418	534.1786152
		+	205.224	0.0354522	536.3697517
-205.219	205.219				
-205.219	205.219	+	205.224	0.0354718	536.3706216
		+	252.992	0.0186509	540.0579520
-25.297	25.297				
-314.468	314.468	+	31.461	0.0140566	540.3056555
		+	259.952	0.0187994	541.3089645
-259.925	259.925				
-284.364	284.364	+	284.421	0.0144169	543.3247471
		+	193.222	0.0424633	543.4936201
-193.218	193.218				
-251.154	251.154	+	251.175	0.0187497	545.3398350
		+	251.175	0.0187735	545.3402334
-251.154	251.154				
-193.874	193.874	+	193.878	0.0418307	546.2639560
		+	18.985	0.0450349	546.2950686
-189.847	189.847				
-249.127	249.127	+	249.147	0.0191589	546.3434303
		+	409.764	0.00625	547.3456290
-406.502	406.502				
-259.601	259.601	+	259.628	0.0185438	549.2640042
		+	205.734	0.0351079	550.1737687
-205.728	205.728				
-275.391	275.391	+	275.435	0.0156893	550.3861708
		+	238.648	0.0222249	556.3371745
-238.633	238.633				
-208.699	208.699	+	208.705	0.0340827	558.2954268
		+	201.916	0.037656	559.2986433
-201.911	201.911				
-219.848	219.848	+	219.856	0.0277282	564.3058250
		+	2.173	0.0292687	565.3090615
-217.292	217.292				
-214.639	214.639	+	214.646	0.0310045	565.3114035

		+	22.404	0.0264128	565.5665116
-22.403	22.403				
-223.946	223.946	+	223.955	0.0264006	566.3127378
		+	209.126	0.0338662	566.3215387
-20.912	20.912				
-241.278	241.278	+	241.294	0.0216288	566.5681762
		+	230.422	0.0242487	566.5703761
-230.411	230.411				
-189.019	189.019	+	189.022	0.0456649	567.2330651
		+	216.495	0.0300186	567.3248770
-216.488	216.488				
-191.153	191.153	+	191.156	0.0441637	568.1846633
		+	186.811	0.0479949	568.3274467
-186.808	186.808				
-238.933	238.933	+	238.948	0.0224049	568.3376904
		+	185.449	0.049455	569.0971185
-185.446	185.446				
-33.723	33.723	+	33.752	0.00918519	569.3380404
		+	239.705	0.022213	569.3412380
-23.969	23.969				
-219.481	219.481	+	219.489	0.0278182	569.3683506
		+	215.547	0.0304794	570.1126685
-215.539	215.539				
-198.516	198.516	+	198.521	0.0395165	570.3545328
		+	244.587	0.0216758	572.3685687
-24.457	24.457				
-188.457	188.457	+	18.846	0.0463062	573.9976115
		+	237.073	0.0228388	576.3271616
-237.059	237.059				
-286.845	286.845	+	286.906	0.01464	577.5552615
		+	364.152	0.00997183	583.3843688
-363.464	363.464				
-248.005	248.005	+	248.024	0.0199758	583.5082923
		+	261.104	0.0190548	584.2937095
-261.076	261.076				
-353.281	353.281	+	353.769	0.00897778	585.5238681
		+	362.607	0.0106316	586.5272636

-361.953	361.953				
-34.199	34.199	+	342.328	0.00982759	587.5392124
		+	193.156	0.0425365	588.3122104
-193.152	193.152				
-258.826	258.826	+	258.853	0.0180795	589.5552125
		+	191.916	0.043838	590.0818977
-191.912	191.912				
-186.014	186.014	+	186.017	0.0491362	590.3215582
		+	342.437	0.01	590.5585786
-342.098	342.098				
-318.497	318.497	+	318.658	0.0148454	592.1213285
		+	201.308	0.0381511	592.3371033
-201.303	201.303				
-221.558	221.558	+	221.567	0.0270823	592.3582493
		+	196.965	0.04031	592.5742948
-196.961	196.961				
-311.647	311.647	+	311.777	0.0142105	593.1246481
		+	374.898	0.00892857	593.2483639
-373.906	373.906				
-185.759	185.759	+	185.762	0.0493278	593.3393661
		+	255.599	0.0188579	593.5866296
-255.575	255.575				
-192.523	192.523	+	192.527	0.0429068	594.1264470
		+	242.096	0.0218471	594.2520347
-24.208	24.208				
-221.299	221.299	+	221.308	0.0270957	611.4156262
		+	315.197	0.0142584	611.5393275
-315.052	315.052				
-316.273	316.273	+	316.423	0.0141872	611.5403721
		+	260.232	0.0189737	612.6074937
-260.204	260.204				
-411.403	411.403	+	415.281	0.008	613.5553758
		+	304.519	0.0133919	614.1033163
-304.415	304.415				
-466.151	466.151	+	477.445	0	614.5585174
		+	193.316	0.0423094	614.6232562
-193.312	193.312				
-185.852	185.852	+	185.855	0.0492648	615.1069676

		+	318.608	0.0147692	617.2460831
-318.447	318.447				
-204.829	204.829	+	204.835	0.0356614	618.2481340
		+	213.972	0.0311686	618.2520005
-213.965	213.965				
-200.554	200.554	+	200.558	0.0384404	618.3411240
		+	188.764	0.0458992	619.2635092
-188.761	188.761				
-379.235	379.235	+	380.436	0.00943396	619.6021869
		+	368.545	0.00806452	621.2794369
-367.746	367.746				
-367.436	367.436	+	368.226	0.00793651	621.2805369
-26.936	26.936	+	269.397	0.0168283	623.1015912
		+	230.311	0.0241869	624.2113020
-2.303	2.303				
-239.313	239.313	+	239.327	0.0221107	625.4314428
		+	265.691	0.0186981	626.4897524
-265.658	265.658				
-265.639	265.639	+	265.672	0.0186667	626.4907125
-302.277	302.277	+	302.375	0.012863	627.4940370
		+	254.809	0.0188384	630.3397822
-254.786	254.786				
-254.786	254.786	+	254.809	0.0188126	630.3402027
		+	187.812	0.0468841	631.1880949
-187.809	187.809				
-255.391	255.391	+	255.415	0.0189404	631.3433620
		+	286.342	0.0144236	631.5646357
-286.282	286.282				
-296.461	296.461	+	296.543	0.0148957	632.3469945
		+	255.789	0.0187972	633.2577090
-255.765	255.765				
-229.296	229.296	+	229.307	0.0245776	633.2607873
		+	263.312	0.0190242	636.2885484
-263.282	263.282				
-222.936	222.936	+	222.945	0.0263988	636.2914890
		+	223.689	0.0263861	637.2965855

-22.368	22.368				
-287.542	287.542	+	287.605	0.0145354	637.5763957
		+	288.838	0.0144688	638.3063938
-288.773	288.773				
-281.585	281.585	+	281.637	0.015435	639.3095568
		+	281.704	0.0154697	639.3103867
-281.652	281.652				
-22.093	22.093	+	220.938	0.0272895	639.4552214
		+	192.757	0.0427189	640.3213070
-192.754	192.754				
-288.021	288.021	+	288.084	0.0145026	640.4674625
		+	193.254	0.0423899	641.3831436
-193.251	193.251				
-266.665	266.665	+	266.698	0.0179384	641.4711194
		+	285.978	0.0144915	643.2640506
-285.919	285.919				
-376.409	376.409	+	377.493	0.00925926	644.3556078
		+	38.743	0.00851064	645.3585743
-385.899	385.899				
-218.134	218.134	+	218.142	0.0287342	648.4725538
		+	24.264	0.0219389	649.4759508
-242.624	242.624				
-34.325	34.325	+	343.602	0.00907143	652.3220180
		+	295.356	0.0146707	652.4674513
-295.277	295.277				
-188.368	188.368	+	188.371	0.0463326	653.4628094
		+	22.471	0.0261253	653.4709796
-224.701	224.701				
-259.694	259.694	+	259.721	0.0186282	654.0956157
		+	189.136	0.0455717	654.6158053
-189.133	189.133				
-329.584	329.584	+	329.812	0.0104575	655.0989713
		+	193.777	0.0418987	656.1040850
-193.773	193.773				
-311.926	311.926	+	312.058	0.0144643	656.3556224
		+	290.362	0.0147459	657.3586322
-290.294	290.294				
-27.783	27.783	+	277.876	0.0153782	657.3624725

		+	193.038	0.042577	658.2743734
-193.034	193.034				
-251.764	251.764	+	251.786	0.0187577	658.3713618
		+	352.548	0.00868817	659.2790905
-352.078	352.078				
-342.034	342.034	+	342.373	0.00991304	659.2807185
		+	252.358	0.0187415	659.3747307
-252.336	252.336				
-311.907	311.907	+	312.039	0.0144	660.2888060
		+	311.436	0.0139655	660.2905254
-311.307	311.307				
-273.956	273.956	+	273.998	0.0164934	660.3777385
		+	346.495	0.00949533	661.2923132
-346.109	346.109				
-219.726	219.726	+	219.734	0.0278224	662.2950355
		+	274.866	0.0161927	662.4491660
-274.824	274.824				
-275.298	275.298	+	275.341	0.0156589	662.4504984
		+	208.747	0.0341026	663.3933360
-208.741	208.741				
-208.778	208.778	+	208.784	0.034084	664.2837149
		+	241.917	0.0218237	664.2960755
-241.901	241.901				
-184.801	184.801	+	184.804	0.0498516	664.4674917
		+	250.871	0.018855	665.4690845
-25.085	25.085				
-252.326	252.326	+	252.347	0.0187171	665.4709606
		+	192.547	0.0429265	666.3371162
-192.543	192.543				
-189.574	189.574	+	189.577	0.0454225	666.4831847
		+	186.723	0.0480389	667.4865293
-18.672	18.672				
-226.182	226.182	+	226.192	0.0256458	667.5271735
		+	212.895	0.0309864	668.4894093
-212.888	212.888				
-217.444	217.444	+	217.452	0.0291846	669.4932666
		+	220.865	0.0271718	669.5022301

-220.857	220.857				
-221.076	221.076	+	221.084	0.027159	670.0047913
		+	198.413	0.0396176	670.5053944
-198.409	198.409				
-196.541	196.541	+	196.545	0.0403161	674.2478415
		+	200.038	0.0387416	674.3659270
-200.033	200.033				
-213.319	213.319	+	213.326	0.0311325	677.5686701
		+	213.763	0.031073	677.5702813
-213.756	213.756				
-221.806	221.806	+	221.815	0.0269226	678.3385161
		+	213.324	0.0311131	678.3407334
-213.317	213.317				
-310.384	310.384	+	310.509	0.0137288	678.5722790
		+	199.974	0.038702	679.3433554
-199.969	199.969				
-191.517	191.517	+	191.521	0.0438736	679.5845353
		+	2.669	0.018031	680.3537088
-266.866	266.866				
-274.658	274.658	+	274.701	0.0166833	680.4984399
		+	194.481	0.0416543	680.5879603
-194.477	194.477				
-205.434	205.434	+	20.544	0.0352765	681.2628200
		+	285.312	0.0142148	681.3571085
-285.254	285.254				
-22.053	22.053	+	220.539	0.0275129	681.4930952
		+	263.996	0.0192733	682.2726997
-263.966	263.966				
-286.623	286.623	+	286.683	0.0146035	682.3674826
		+	339.474	0.00984127	682.3711028
-339.165	339.165				
-235.414	235.414	+	235.427	0.0232864	683.2760371
		+	284.817	0.0142494	683.3736600
-284.759	284.759				
-279.414	279.414	+	279.463	0.0153521	688.4650196
		+	252.495	0.0187906	689.4683352
-252.473	252.473				
-249.529	249.529	+	249.549	0.0189544	690.2985580

		+	237.824	0.0227232	691.3066108
-23.781	23.781				
-205.552	205.552	+	205.557	0.0351517	691.9477277
		+	215.053	0.0307589	692.3146207
-215.046	215.046				
-239.542	239.542	+	239.556	0.0222016	693.3179406
		+	25.405	0.0186433	693.3216227
-254.027	254.027				
-277.438	277.438	+	277.484	0.0156017	695.4984544
		+	231.677	0.0236332	696.2287077
-231.666	231.666				
-237.902	237.902	+	237.916	0.0224379	696.2315330
		+	237.755	0.0227008	696.3481289
-237.741	237.741				
-202.501	202.501	+	202.505	0.0370355	697.3521509
		+	185.603	0.0493511	698.2653855
-1.856	1.856				
-189.932	189.932	+	189.935	0.0450062	699.5328485
		+	213.141	0.0311407	700.2795483
-213.134	213.134				
-21.391	21.391	+	213.917	0.0311094	700.2809747
		+	278.368	0.0154017	702.2972662
-27.832	27.832				
-281.453	281.453	+	281.505	0.0152978	702.3363252
		+	208.906	0.0339802	703.3383584
-2.089	2.089				
-221.471	221.471	+	221.479	0.0270233	703.3420714
		+	228.618	0.0245414	704.3136776
-228.607	228.607				
-19.867	19.867	+	198.674	0.0394111	705.3570181
		+	203.661	0.0363825	706.3718111
-203.656	203.656				
-191.415	191.415	+	191.419	0.0439386	707.3732641
		+	289.118	0.0146984	707.4984743
-289.053	289.053				
-352.196	352.196	+	352.668	0.00878261	707.5019769
		+	190.419	0.0447929	707.5970027

-190.415	190.415				
-197.593	197.593	+	197.597	0.0401147	708.5020147
		+	341.963	0.00966102	711.5339723
-341.629	341.629				
-217.775	217.775	+	217.782	0.0291366	712.2268418
		+	186.097	0.0489564	712.5444608
-186.094	186.094				
-290.742	290.742	+	290.811	0.0147143	713.5475705
		+	200.816	0.0383946	713.5568792
-200.811	200.811				
-266.791	266.791	+	266.824	0.018	716.3146208
		+	244.749	0.0217006	717.3224878
-244.732	244.732				
-210.321	210.321	+	210.327	0.0329288	717.5896963
		+	210.327	0.0329485	717.5906498
-210.321	210.321				
-274.393	274.393	+	274.435	0.0166195	718.3293661
		+	274.951	0.0160309	718.3307058
-274.909	274.909				
-215.952	215.952	+	215.959	0.0303726	718.5934758
		+	255.023	0.0189421	719.3379728
-254.999	254.999				
-249.493	249.493	+	249.513	0.0189077	719.6062048
		+	209.677	0.0334004	720.1355444
-209.671	209.671				
-222.649	222.649	+	222.658	0.0264124	720.3460522
		+	234.253	0.0236648	721.3492178
-234.241	234.241				
-23.421	23.421	+	234.223	0.0236431	721.3504016
		+	231.416	0.0237043	722.3523537
-231.404	231.404				
-191.078	191.078	+	191.082	0.0443715	724.2823479
		+	213.925	0.0311291	724.5704276
-213.918	213.918				
-304.738	304.738	+	304.843	0.0134412	728.1374619
		+	220.352	0.0276914	728.2022509
-220.343	220.343				
-303.246	303.246	+	303.347	0.0131329	729.1394185

		+	302.511	0.0129072	729.1409846
-302.413	302.413				
-350.762	350.762	+	351.212	0.00936082	729.2643544
		+	206.482	0.0349038	729.4834928
-206.476	206.476				
-188.475	188.475	+	188.478	0.0462363	729.5591099
		+	185.652	0.0493711	729.5608669
-185.649	185.649				
-197.087	197.087	+	197.091	0.040222	729.5789339
		+	220.886	0.0272109	729.5825625
-220.878	220.878				
-369.042	369.042	+	369.878	0.00847458	730.1428615
		+	195.308	0.0412131	730.2372005
-195.304	195.304				
-249.548	249.548	+	249.568	0.0189778	731.2791179
		+	250.197	0.01895	731.2807116
-250.177	250.177				
-239.613	239.613	+	239.628	0.0222704	731.4977136
		+	222.867	0.026375	731.5020751
-222.858	222.858				
-236.042	236.042	+	236.056	0.0231649	732.4975700
		+	305.348	0.0133184	732.5015360
-305.241	305.241				
-293.859	293.859	+	293.934	0.0142405	733.5138512
		+	223.506	0.026497	734.3043284
-223.496	223.496				
-201.088	201.088	+	201.093	0.0384142	735.5039363
		+	274.348	0.0165562	735.5294586
-274.306	274.306				
-274.388	274.388	+	27.443	0.0165878	735.5304427
		+	193.758	0.0419259	736.3422665
-193.755	193.755				
-271.939	271.939	+	271.978	0.0167435	737.5455255
		+	316.029	0.0139806	737.5558890
-31.588	31.588				
-188.638	188.638	+	188.641	0.0462064	738.5392080
		+	209.887	0.0333017	739.5693832

-209.881	209.881				
-219.639	219.639	+	219.647	0.0277949	739.5716497
		+	207.481	0.0344128	741.3218693
-207.475	207.475				
-19.922	19.922	+	199.224	0.0391323	743.3340952
		+	215.602	0.0302868	746.2895416
-215.595	215.595				
-215.963	215.963	+	215.971	0.0303926	746.2912600
		+	259.544	0.0184602	747.2946621
-259.517	259.517				
-290.738	290.738	+	290.807	0.014674	750.1191640
		+	28.719	0.0144623	750.1206567
-287.128	287.128				
-199.223	199.223	+	199.228	0.0391521	750.5769684
		+	213.554	0.0309561	751.1241131
-213.547	213.547				
-187.689	187.689	+	187.692	0.0470428	751.5807613
		+	24.152	0.021721	752.3392717
-241.504	241.504				
-241.649	241.649	+	241.664	0.0217906	752.3407802
		+	189.302	0.04556	752.5926977
-189.299	189.299				
-204.482	204.482	+	204.487	0.0362578	754.3541630
		+	228.785	0.0246628	755.4983179
-228.774	228.774				
-19.281	19.281	+	192.814	0.0428176	756.5878945
		+	202.273	0.0371272	756.6066935
-202.268	202.268				
-336.064	336.064	+	336.343	0.00905109	757.2957156
		+	2.382	0.0223897	757.5914582
-238.186	238.186				
-200.142	200.142	+	200.146	0.0386987	758.3032219
		+	215.051	0.030739	759.6088877
-215.044	215.044				
-224.732	224.732	+	224.742	0.0260681	760.5763392
		+	21.064	0.0327855	762.5387743

-210.633	210.633				
-214.008	214.008	+	214.015	0.0312081	762.5409885
		+	253.035	0.0187001	762.6009366
-253.013	253.013				
-304.965	304.965	+	305.071	0.0131704	764.5562275
		+	244.362	0.0215283	765.5593343
-244.345	244.345				
-243.959	243.959	+	243.976	0.0214955	765.5606543
		+	285.004	0.0140804	766.3561075
-284.946	284.946				
-227.862	227.862	+	227.873	0.0251828	766.5719673
		+	28.162	0.0154004	767.3591403
-281.568	281.568				
-281.439	281.439	+	281.491	0.0152639	767.3608575
		+	239.505	0.0221788	767.5754068
-23.949	23.949				
-223.921	223.921	+	22.393	0.0263204	768.1312365
		+	230.404	0.0242281	770.6046728
-230.393	230.393				
-282.827	282.827	+	282.881	0.0151284	771.6082510
		+	294.884	0.0145826	771.6104393
-294.806	294.806				
-205.923	205.923	+	205.928	0.0350022	772.3069297
		+	311.716	0.014087	773.3137679
-311.586	311.586				
-290.882	290.882	+	290.951	0.0147548	774.3224522
-31.088	31.088	+	311.007	0.0138462	775.3258329
		+	325.801	0.0137485	776.3283728
-3.256	3.256				
-247.548	247.548	+	247.567	0.0202683	776.4835510
-252.694	252.694	+	252.716	0.0188895	776.5926986
		+	307.606	0.01336	777.5961410
-307.491	307.491				
-22.254	22.254	+	222.549	0.0264395	778.6082688
		+	291.485	0.0149609	779.6119065
-291.415	291.415				
-290.466	290.466	+	290.534	0.0145543	780.3726750

		+	262.216	0.0189712	780.5878989
-262.187	262.187				
-352.975	352.975	+	353.459	0.00887912	781.5893424
		+	345.805	0.00940741	781.5914233
-345.427	345.427				
-23.119	23.119	+	231.201	0.0238438	784.6089281
		+	219.297	0.0278103	784.6446581
-219.289	219.289				
-191.948	191.948	+	191.952	0.0438068	788.3376916
		+	2.319	0.0236699	788.5562539
-231.888	231.888				
-236.487	236.487	+	2.365	0.0233879	789.5592847
		+	2.365	0.0233654	789.5604805
-236.487	236.487				
-186.711	186.711	+	186.714	0.0480406	790.5719182
		+	199.911	0.0388155	791.5753994
-199.906	199.906				
-313.045	313.045	+	313.181	0.0143379	792.3721321
		+	42.265	0.00869565	792.5419526
-417.833	417.833				
-23.508	23.508	+	235.093	0.0234799	792.5881219
		+	229.467	0.0245138	792.5905171
-229.456	229.456				
-229.947	229.947	+	229.958	0.0244044	793.5441614
		+	261.733	0.0191683	793.5870105
-261.704	261.704				
-291.292	291.292	+	291.362	0.0149192	793.5917922
		+	274.619	0.0166513	794.3873746
-274.576	274.576				
-212.298	212.298	+	212.305	0.0313317	794.6034364
		+	185.291	0.0495871	794.6865832
-185.288	185.288				
-244.509	244.509	+	244.526	0.0216018	795.2950012
		+	303.846	0.0133665	795.3893280
-303.743	303.743				
-303.364	303.364	+	303.465	0.0132254	795.3908831
		+	213.671	0.0310145	795.6069199

-213.664	213.664				
-222.916	222.916	+	222.925	0.0263398	795.6893766
		+	222.925	0.0263594	795.6906418
-222.916	222.916				
-301.535	301.535	+	30.163	0.0134102	796.3051893
		+	280.024	0.0153853	796.3940970
-279.974	279.974				
-287.546	287.546	+	287.609	0.0145722	797.2879993
		+	35.803	0.00928736	797.3084961
-357.468	357.468				
-277.095	277.095	+	27.714	0.0157113	797.6627971
		+	201.062	0.0383742	798.4658304
-201.058	201.058				
-191.997	191.997	+	1.92	0.0437556	798.6255574
		+	228.869	0.024724	798.6363542
-228.858	228.858				
-267.134	267.134	+	267.168	0.0178897	798.6660621
		+	250.732	0.0189333	799.6409867
-250.712	250.712				
-219.424	219.424	+	219.432	0.0277987	800.3125367
		+	278.304	0.0153036	800.5569700
-278.257	278.257				
-228.612	228.612	+	228.623	0.0245616	800.6526380
		+	276.443	0.0159514	802.3153138
-276.398	276.398				
-251.922	251.922	+	251.943	0.0187012	802.4992167
		+	251.943	0.018677	802.5002253
-251.922	251.922				
-188.421	188.421	+	188.424	0.0462662	802.6681900
		+	271.737	0.0166199	803.5026320
-271.698	271.698				
-276.335	276.335	+	276.379	0.0159192	804.5053506
		+	25.681	0.0189044	804.5732351
-256.785	256.785				
-394.515	394.515	+	396.619	0.0047619	804.5869063
		+	300.017	0.0136262	806.3480713
-299.926	299.926				
-205.602	205.602	+	205.608	0.0351605	806.6263709

		+	185.472	0.0494755	808.2655450
-185.469	185.469				
-190.103	190.103	+	190.107	0.0448135	808.6385789
		+	252.556	0.0188152	809.6663692
-252.535	252.535				
-184.903	184.903	+	184.906	0.0498361	811.6671728
		+	413.921	0.00714286	811.7147932
-410.202	410.202				
-193.056	193.056	+	19.306	0.0425039	812.2985776
		+	209.779	0.0334401	812.3016000
-209.773	209.773				
-18.505	18.505	+	185.053	0.0496531	812.6693362
		+	184.866	0.0497542	812.6709137
-184.863	184.863				
-310.268	310.268	+	310.393	0.0136709	814.3148981
		+	216.855	0.0297059	814.3537004
-216.848	216.848				
-230.999	230.999	+	231.011	0.0238066	815.3567193
		+	201.377	0.0380303	815.5755617
-201.372	201.372				
-21.124	21.124	+	211.247	0.031828	816.3278342
		+	213.887	0.0310898	816.3687801
-21.388	21.388				
-216.209	216.209	+	216.216	0.0300119	816.3707496
		+	233.402	0.0236531	816.5878394
-23.339	23.339				
-193.857	193.857	+	193.861	0.0417914	817.3728343
		+	226.942	0.02504	817.5884509
-226.932	226.932				
-231.165	231.165	+	231.176	0.0238231	817.5913964
		+	28.312	0.0152333	818.2884879
-283.065	283.065				
-270.443	270.443	+	27.048	0.016591	818.2911512
		+	211.907	0.0315873	818.3852930
-211.901	211.901				
-231.547	231.547	+	231.559	0.0236585	818.6035184
		+	331.118	0.0101351	818.6265427

-330.881	330.881				
-214.601	214.601	+	214.608	0.0309647	819.6068150
		+	215.545	0.0304595	820.6192369
-215.538	215.538				
-215.309	215.309	+	215.317	0.0306086	820.6209284
		+	286.151	0.0145623	821.3135159
-286.092	286.092				
-23.064	23.064	+	230.651	0.0242228	821.6234442
		+	224.084	0.0264331	821.6658729
-224.074	224.074				
-209.029	209.029	+	209.035	0.0338058	822.5843563
		+	295.178	0.0146265	822.6345256
-2.951	2.951				
-243.944	243.944	+	243.961	0.0214713	823.6382678
		+	206.227	0.0349714	823.6781353
-206.222	206.222				
-202.274	202.274	+	202.279	0.0371468	823.6809463
		+	223.805	0.0263722	824.6494876
-223.796	223.796				
-223.883	223.883	+	223.892	0.0262805	824.6509344
		+	196.343	0.0404981	824.6692591
-196.339	196.339				
-196.546	196.546	+	19.655	0.0403358	824.6704148
		+	218.377	0.0284184	824.6816930
-218.369	218.369				
-217.832	217.832	+	21.784	0.0289986	825.5748648
		+	259.642	0.0186	825.6537607
-259.615	259.615				
-294.087	294.087	+	294.163	0.0143669	825.6940646
		+	20.364	0.0363629	826.5568119
-203.635	203.635				
-193.302	193.302	+	193.305	0.0423166	826.5615029
		+	232.788	0.0234469	826.6975636
-232.776	232.776				
-315.467	315.467	+	315.614	0.0138462	827.3226930
		+	238.589	0.0222583	827.6992700
-238.575	238.575				
-23.532	23.532	+	235.333	0.0232645	828.3295980

		+	235.302	0.0232427	828.3307486
-235.289	235.289				
-273.365	273.365	+	273.406	0.0165887	828.6088209
		+	27.194	0.0167124	828.6104189
-271.901	271.901				
-233.025	233.025	+	233.037	0.023552	829.3340823
		+	288.954	0.0146211	829.6126437
-288.889	288.889				
-262.095	262.095	+	262.124	0.0191005	830.6245858
		+	361.072	0.0102278	832.3643785
-36.045	36.045				
-208.471	208.471	+	208.477	0.0341381	833.6495292
		+	208.599	0.0340035	833.6507455
-208.594	208.594				
-237.381	237.381	+	237.394	0.0227241	833.6663397
		+	274.736	0.0165308	834.2818967
-274.693	274.693				
-333.142	333.142	+	333.397	0.0103448	834.3791101
		+	335.697	0.00885714	834.3816229
-335.423	335.423				
-246.461	246.461	+	24.648	0.0203302	834.6543495
		+	359.119	0.00950588	835.2881439
-358.537	358.537				
-394.829	394.829	+	396.957	0.005	835.2922245
		+	398.018	0.00512821	835.3839439
-395.811	395.811				
-253.819	253.819	+	253.842	0.0186738	836.2967349
		+	319.292	0.0149462	837.2992722
-319.127	319.127				
-319.275	319.275	+	31.944	0.0151087	837.3009422
		+	252.648	0.0188647	837.6966538
-252.626	252.626				
-190.153	190.153	+	190.156	0.0448731	839.7089849
		+	190.156	0.044893	839.7104394
-190.153	190.153				
-397.317	397.317	+	39.965	0.00526316	839.7459913
		+	278.828	0.0155345	840.6083344

-27.878	27.878				
-287.954	287.954	+	288.017	0.0144655	840.6104769
		+	414.065	0.00740741	840.7490653
-410.329	410.329				
-41.083	41.083	+	414.632	0.00769231	840.7504188
		+	1.911	0.0443179	841.6067929
-191.096	191.096				
-280.853	280.853	+	280.904	0.0150635	841.6678490
		+	191.149	0.044169	841.7039784
-191.145	191.145				
-254.904	254.904	+	254.927	0.0188642	842.3456503
		+	249.697	0.0190248	842.4887502
-249.677	249.677				
-245.717	245.717	+	245.735	0.0208419	842.4916787
		+	209.724	0.0334005	842.6715371
-209.718	209.718				
-188.422	188.422	+	188.425	0.0462862	843.6067477
		+	184.822	0.0498227	843.6135718
-184.819	184.819				
-219.797	219.797	+	219.805	0.0279208	844.6180637
		+	208.121	0.0341953	844.6292249
-208.116	208.116				
-185.335	185.335	+	185.338	0.0495453	845.6436905
-20.067	20.067	+	200.675	0.0383252	848.7694772
		+	200.675	0.038345	848.7704419
-20.067	20.067				
-203.911	203.911	+	203.916	0.0364466	851.6974170
		+	303.874	0.0134143	851.7093541
-303.772	303.772				
-303.772	303.772	+	303.874	0.0134624	851.7103929
		+	271.319	0.0167119	852.5746330
-271.281	271.281				
-209.764	209.764	+	20.977	0.0334203	852.6794723
-245.063	245.063	+	24.508	0.0212308	852.7130196
		+	19.544	0.0411481	853.5810880
-195.436	195.436				
-422.121	422.121	+	42.764	0.00526316	853.6062113

		+	26.638	0.0182184	853.7253567
-266.347	266.347				
-249.955	249.955	+	249.975	0.0191194	854.3459053
		+	259.046	0.0182407	854.7286099
-25.902	25.902				
-258.977	258.977	+	259.004	0.0181867	854.7302388
		+	204.916	0.0356125	855.3525602
-20.491	20.491				
-212.447	212.447	+	212.453	0.0312857	855.4967465
		+	207.682	0.0344721	856.3187272
-207.677	207.677				
-226.292	226.292	+	226.302	0.0255962	856.3216235
		+	228.282	0.0247837	856.3595843
-228.271	228.271				
-228.151	228.151	+	228.162	0.0251028	856.3617048
		+	242.155	0.0218707	857.3651194
-242.139	242.139				
-287.594	287.594	+	287.657	0.0146463	858.2791219
		+	281.579	0.0153661	858.2814774
-281.527	281.527				
-271.095	271.095	+	271.133	0.0166813	858.3675485
		+	184.872	0.0497747	858.5705983
-18.487	18.487				
-203.572	203.572	+	203.577	0.0362259	861.5448343
		+	205.606	0.035141	861.5724999
-2.056	2.056				
-239.908	239.908	+	239.922	0.0220124	862.5759072
		+	30.813	0.0135772	862.5927937
-308.013	308.013				
-215.531	215.531	+	215.538	0.0304397	863.5862415
		+	250.073	0.0188792	864.1534438
-250.053	250.053				
-242.824	242.824	+	24.284	0.0219736	864.6086034
		+	243.392	0.0219111	864.6104323
-243.376	243.376				
-189.265	189.265	+	189.268	0.0455002	865.1567309
		+	276.208	0.01576	865.6125540
-276.163	276.163				
-201.406	201.406	+	20.141	0.0380501	865.7252575

		+	284.193	0.0148345	866.3068476
-284.137	284.137				
-287.305	287.305	+	287.367	0.0144987	866.6250396
		+	245.284	0.0213041	867.3149172
-245.266	245.266				
-246.326	246.326	+	246.344	0.0203283	867.6835672
		+	230.068	0.0243612	867.7395852
-230.057	230.057				
-230.057	230.057	+	230.068	0.0243818	867.7410870
		+	484.075	0	867.7773539
-472.932	472.932				
-249.752	249.752	+	249.772	0.0190957	868.3188729
		+	259.132	0.0182679	868.3227046
-259.106	259.106				
-291.586	291.586	+	291.657	0.0150028	868.6390860
		+	29.111	0.0148778	868.6410106
-291.041	291.041				
-481.972	481.972	+	492.356	0	868.7795334
		+	492.385	0	868.7809965
-482.005	482.005				
-255.985	255.985	+	256.009	0.0186648	868.9639476
		+	229.334	0.0246389	869.3269602
-229.323	229.323				
-218.814	218.814	+	218.822	0.0283081	869.6090202
		+	219.314	0.0278492	869.6103745
-219.306	219.306				
-32.194	32.194	+	322.119	0.0147746	869.6991083
		+	322.119	0.0146897	869.7003473
-32.194	32.194				
-230.099	230.099	+	23.011	0.024359	869.7930370
		+	198.393	0.0397079	870.5859735
-198.389	198.389				
-379.449	379.449	+	380.658	0.00961538	870.6219535
		+	272.896	0.0164644	870.6555199
-272.856	272.856				
-271.458	271.458	+	271.497	0.0167735	870.7027040
		+	214.202	0.0313436	870.7964003

-214.195	214.195				
-197.617	197.617	+	197.621	0.0401544	874.2547111
		+	18.966	0.0453962	874.5365994
-189.657	189.657				
-233.728	233.728	+	23.374	0.0238253	874.7855872
		+	206.936	0.0346704	876.2886395
-206.931	206.931				
-20.836	20.836	+	208.366	0.0341635	876.2905494
		+	212.575	0.0310585	876.3324668
-212.569	212.569				
-201.062	201.062	+	201.067	0.0383942	876.7996313
		+	201.052	0.0383344	876.8013617
-201.047	201.047				
-194.278	194.278	+	194.282	0.041717	877.5670385
		+	19.012	0.0448333	877.8047054
-190.117	190.117				
-225.264	225.264	+	225.273	0.0259969	878.5888999
		+	225.787	0.0259147	878.5907823
-225.777	225.777				
-244.769	244.769	+	244.786	0.0217503	878.6969892
		+	217.413	0.029193	879.3482739
-217.405	217.405				
-200.167	200.167	+	200.171	0.0386359	879.5815585
		+	341.932	0.00957983	879.7397006
-341.599	341.599				
-341.589	341.589	+	341.922	0.0095	879.7410984
		+	215.729	0.0302928	880.3627548
-215.722	215.722				
-189.201	189.201	+	189.204	0.0456115	880.5845411
		+	187.106	0.0477503	880.5921774
-187.102	187.102				
-317.835	317.835	+	317.993	0.0145455	880.6059789
-30.326	30.326	+	303.361	0.0131789	880.7444148
-35.911	35.911	+	359.704	0.00985366	881.6092335
		+	359.754	0.00997531	881.6104292
-359.159	359.159				
-282.337	282.337	+	282.391	0.0152877	881.7567963

		+	216.176	0.0299921	882.3069490
-216.168	216.168				
-276.701	276.701	+	276.746	0.0161145	882.7595658
		+	276.746	0.0160816	882.7604057
-276.701	276.701				
-197.533	197.533	+	197.538	0.0401046	883.3085627
		+	222.719	0.026323	883.3112558
-22.271	22.271				
-229.374	229.374	+	229.384	0.0245562	883.7149051
		+	230.434	0.0242694	883.7516089
-230.423	230.423				
-24.088	24.088	+	240.896	0.0215521	884.5388682
		+	24.457	0.0216511	884.5406520
-244.553	244.553				
-18.949	18.949	+	189.493	0.0455209	885.6085692
		+	187.807	0.0468439	885.6107081
-187.804	187.804				
-319.593	319.593	+	31.976	0.0151913	886.5931430
		+	403.253	0.00540541	888.3561184
-400.619	400.619				
-433.644	433.644	+	44.123	0.00714286	888.5732593
		+	218.308	0.0285086	888.6086429
-2.183	2.183				
-218.559	218.559	+	218.567	0.0282899	888.6106341
		+	197.594	0.0400949	889.5768726
-19.759	19.759				
-221.095	221.095	+	221.104	0.0271786	890.6242363
		+	186.512	0.0484081	893.7566660
-186.509	186.509				
-251.408	251.408	+	251.429	0.0188455	894.3383665
		+	2.484	0.0194576	894.3409632
-24.838	24.838				
-410.047	410.047	+	413.746	0.00689655	894.6216745
		+	265.316	0.0185729	895.3461926
-265.284	265.284				
-358.225	358.225	+	358.801	0.00939535	895.7149201
		+	20.002	0.0387218	895.7723462
-200.015	200.015				
-319.792	319.792	+	31.996	0.0153591	895.8085533

		+	24.408	0.0215197	896.3540864
-244.064	244.064				
-247.139	247.139	+	247.158	0.0202426	896.7182671
		+	246.359	0.0203521	897.3577212
-246.341	246.341				
-242.951	242.951	+	242.967	0.0219361	897.3609526
		+	207.347	0.0343324	897.6388735
-207.341	207.341				
-208.044	208.044	+	20.805	0.0342135	897.6409423
		+	39.429	0.00681818	897.7294762
-392.348	392.348				
-392.494	392.494	+	394.447	0.00697674	897.7307950
		+	203.686	0.0364218	898.3575995
-203.681	203.681				
-220.261	220.261	+	220.269	0.0276519	898.3626757
		+	347.201	0.00986408	898.7339159
-346.806	346.806				
-27.108	27.108	+	271.118	0.0166508	899.6211335
		+	191.582	0.0438569	899.7092219
-191.579	191.579				
-191.579	191.579	+	191.582	0.0438371	899.7105798
		+	223.981	0.0264207	901.7253539
-223.972	223.972				
-27.679	27.679	+	276.836	0.0161807	902.5888527
		+	276.661	0.0160489	902.5906496
-276.616	276.616				
-224.516	224.516	+	224.526	0.0264827	902.8169142
		+	228.478	0.0247109	903.5934129
-228.467	228.467				
-226.783	226.783	+	226.793	0.0251994	903.7394940
		+	227.117	0.0250602	903.7412512
-227.107	227.107				
-215.939	215.939	+	215.947	0.0303327	903.8193194
		+	215.947	0.0303526	903.8207287
-215.939	215.939				
-288.795	288.795	+	28.886	0.0145065	904.1459460
		+	199.675	0.0389211	904.6052341

-19.967	19.967				
-237.196	237.196	+	23.721	0.0227329	904.8326454
		+	351.303	0.00841667	905.1470344
-350.852	350.852				
-220.865	220.865	+	220.874	0.0271914	905.6086215
		+	22.064	0.0274892	905.6104696
-220.632	220.632				
-241.876	241.876	+	241.892	0.0218002	905.7568189
		+	189.616	0.0454185	905.8360346
-189.613	189.613				
-361.558	361.558	+	362.203	0.010359	906.6170440
		+	436.131	0.00588235	906.6217860
-429.334	429.334				
-467.324	467.324	+	478.618	0	907.6253648
		+	357.275	0.00918182	907.7723890
-356.727	356.727				
-269.193	269.193	+	269.229	0.016769	908.6372910
		+	313.281	0.0144037	908.7757011
-313.144	313.144				
-323.549	323.549	+	323.737	0.0145325	909.6395983
		+	323.737	0.0144471	909.6409057
-323.549	323.549				
-32.702	32.702	+	32.723	0.0121707	909.7882175
		+	311.022	0.0139056	910.3130329
-310.895	310.895				
-287.588	287.588	+	287.651	0.0146091	910.3383746
		+	289.485	0.014816	910.3405658
-289.419	289.419				
-185.888	185.888	+	185.891	0.0492865	910.6532545
		+	306.158	0.0126996	910.7896721
-306.048	306.048				
-306.058	306.058	+	306.168	0.0127481	910.7914278
		+	330.683	0.0100671	911.3389939
-330.449	330.449				
-338.139	338.139	+	338.437	0.0096875	911.3418827
		+	20.572	0.0350884	911.6564780
-205.714	205.714				
-228.147	228.147	+	228.158	0.0250823	911.7462158

		+	188.532	0.046213	911.7932619
-188.528	188.528				
-207.432	207.432	+	207.438	0.0343931	911.8039174
		+	235.451	0.0233083	912.5009034
-235.438	235.438				
-33.257	33.257	+	33.282	0.010274	912.5716069
		+	292.393	0.0152593	912.6086795
-29.232	29.232				
-440.451	440.451	+	449.244	0	913.7618788
		+	321.614	0.0151771	914.6245385
-321.437	321.437				
-317.221	317.221	+	317.376	0.0144	914.7654652
		+	304.151	0.0135108	915.6257943
-304.047	304.047				
-296.917	296.917	+	2.97	0.0144149	915.7774640
		+	429.696	0.00555556	915.7984189
-423.875	423.875				
-233.382	233.382	+	233.394	0.0236318	916.7815190
		+	273.256	0.0165574	916.8022097
-273.215	273.215				
-288.931	288.931	+	288.997	0.0146596	919.5667180
		+	264.284	0.0191724	921.6686213
-264.253	264.253				
-263.062	263.062	+	263.092	0.0189325	921.6705232
		+	426.278	0.005	923.7461617
-420.955	420.955				
-310.231	310.231	+	310.356	0.0136134	924.7490712
		+	31.162	0.014026	924.7505174
-311.491	311.491				
-263.156	263.156	+	263.186	0.0189935	925.7252582
		+	489.617	0	925.7619640
-478.907	478.907				
-328.791	328.791	+	329.013	0.0115128	926.7653390
		+	243.879	0.0213751	927.7395958
-243.863	243.863				
-243.865	243.865	+	243.882	0.0213991	927.7411831
		+	208.229	0.0341042	927.7776460
-208.223	208.223				
-187.282	187.282	+	187.285	0.0476814	928.6057367

		+	199.647	0.0388815	928.7445069
-199.642	199.642				
-217.234	217.234	+	217.242	0.029249	929.6088526
		+	218.706	0.028299	929.6108213
-218.698	218.698				
-250.102	250.102	+	250.122	0.0189263	929.7567981
		+	292.684	0.0147736	930.4033029
-292.612	292.612				
-222.524	222.524	+	222.533	0.02642	930.5790256
		+	23.022	0.0242102	930.5809087
-230.209	230.209				
-246.635	246.635	+	246.653	0.0203783	930.6194828
		+	24.608	0.0203738	930.6215587
-246.063	246.063				
-202.854	202.854	+	202.859	0.0367702	930.7593752
		+	202.859	0.0367507	930.7607181
-202.854	202.854				
-310.546	310.546	+	310.672	0.0137872	931.4064876
		+	196.288	0.0405362	931.5843985
-196.284	196.284				
-240.009	240.009	+	240.024	0.0221272	931.6250391
		+	345.039	0.00923636	931.7725041
-344.671	344.671				
-346.792	346.792	+	347.187	0.00976923	932.3190330
		+	342.007	0.00974359	932.3215834
-341.673	341.673				
-214.251	214.251	+	214.258	0.0312374	932.6367643
		+	256.631	0.0188506	932.7758845
-256.606	256.606				
-22.162	22.162	+	221.628	0.0269563	933.6392369
		+	221.886	0.0268296	933.6411075
-221.877	221.877				
-191.865	191.865	+	191.868	0.0437783	933.7881776
		+	205.565	0.0351712	934.6531352
-20.556	20.556				
-194.548	194.548	+	194.552	0.0416464	934.7915814
		+	231.095	0.0237818	935.6573542

-231.084	231.084				
-204.196	204.196	+	204.201	0.0363027	936.6673770
		+	215.118	0.0306891	937.6993640
-215.111	215.111				
-215.111	215.111	+	215.118	0.030709	937.7007635
		+	220.069	0.027594	937.8190182
-220.061	220.061				
-219.761	219.761	+	219.769	0.0278617	937.8204722
		+	315.709	0.013913	938.5148810
-315.562	315.562				
-24.743	24.743	+	247.449	0.0203636	938.6843180
		+	290.695	0.0146339	938.7036021
-290.626	290.626				
-384.907	384.907	+	386.383	0.00816327	939.5180857
		+	384.839	0.008	939.5206918
-38.344	38.344				
-210.762	210.762	+	210.768	0.0324511	939.6877989
		+	231.632	0.0236126	941.5681756
-23.162	23.162				
-228.409	228.409	+	22.842	0.0247725	941.5723587
		+	238.259	0.0224341	941.8143594
-238.245	238.245				
-291.039	291.039	+	291.109	0.0148366	942.3648296
		+	23.833	0.0224563	942.5794876
-238.316	238.316				
-240.998	240.998	+	241.013	0.0216203	942.5811963
		+	207.676	0.0344524	942.8178392
-20.767	20.767				
-276.729	276.729	+	276.774	0.0161475	943.3677443
		+	236.812	0.0231141	943.7152899
-236.798	236.798				
-351.711	351.711	+	352.175	0.00859574	943.8290005
		+	352.175	0.00850526	943.8308261
-351.711	351.711				
-319.175	319.175	+	31.934	0.015027	947.6848088
		+	219.688	0.027834	948.6288674
-219.679	219.679				
-214.608	214.608	+	214.614	0.0309846	948.6315019

		+	190.576	0.0447092	949.6361167
-190.573	190.573				
-305.585	305.585	+	305.693	0.0130415	950.3292996
		+	306.915	0.0131496	950.3313393
-306.802	306.802				
-236.099	236.099	+	236.112	0.023187	950.7039922
		+	423.094	0.00909091	951.3348212
-418.216	418.216				
-298.842	298.842	+	29.893	0.0145559	952.3850339
		+	223.342	0.0264174	953.7224179
-223.332	223.332				
-18.528	18.528	+	185.283	0.0495666	953.7325374
		+	269.929	0.0166214	953.7568897
-269.893	269.893				
-307.829	307.829	+	307.945	0.0134677	954.6430309
		+	19.756	0.0401244	954.7354111
-197.556	197.556				
-191.908	191.908	+	191.911	0.0437982	954.7608491
		+	294.227	0.0144095	955.7726199
-294.151	294.151				
-243.821	243.821	+	243.837	0.0213512	956.6372179
		+	209.068	0.0337671	956.6586088
-209.062	209.062				
-20.918	20.918	+	209.186	0.0338472	956.6603626
		+	2.396	0.0222474	956.7761007
-239.585	239.585				
-220.159	220.159	+	220.167	0.0276726	957.6416269
		+	225.282	0.0260172	957.6622551
-225.273	225.273				
-238.541	238.541	+	238.555	0.0223137	957.7882781
		+	238.386	0.0223022	958.6742330
-238.372	238.372				
-218.646	218.646	+	218.654	0.0283094	958.7918107
		+	258.962	0.0181598	959.6778395
-258.936	258.936				
-253.117	253.117	+	253.139	0.0186172	964.3465930
		+	216.289	0.0300317	964.5652432
-216.282	216.282				
-261.567	261.567	+	261.596	0.019205	965.3482185

		+	239.772	0.0221325	965.3516760
-239.757	239.757				
-200.347	200.347	+	200.352	0.0386049	965.5720422
		+	20.618	0.0349882	965.8494426
-206.175	206.175				
-206.175	206.175	+	20.618	0.0349687	965.8510802
		+	203.877	0.0364269	966.5790794
-203.872	203.872				
-204.502	204.502	+	204.507	0.0363173	966.5813598
		+	237.556	0.022791	967.5850982
-237.542	237.542				
-334.844	334.844	+	335.112	0.00943662	968.5973577
		+	337.991	0.00953846	968.6011431
-337.697	337.697				
-368.359	368.359	+	369.175	0.00833333	968.6789743
		+	359.676	0.00973494	968.6808205
-359.083	359.083				
-198.768	198.768	+	198.772	0.0394904	968.6994384
		+	198.238	0.0397488	968.7011497
-198.234	198.234				
-394.782	394.782	+	396.907	0.00487805	969.6032284
		+	201.551	0.0379079	969.6840329
-201.546	201.546				
-195.497	195.497	+	195.501	0.0411396	969.7041206
		+	255.005	0.0189161	970.6090940
-254.982	254.982				
-2.777	2.777	+	277.747	0.015523	970.6122476
		+	331.435	0.0102041	970.6953010
-331.195	331.195				
-311.772	311.772	+	311.903	0.0143363	971.6170534
		+	334.059	0.00930556	971.6207551
-333.799	333.799				
-308.412	308.412	+	30.853	0.0136327	971.6985607
		+	308.116	0.0135223	971.7004752
-3.08	3.08				
-244.473	244.473	+	24.449	0.0215528	972.3133318
		+	194.853	0.0414719	972.6276650

-194.849	194.849				
-197.694	197.694	+	197.698	0.0398475	972.6304153
		+	23.615	0.0232092	973.3168278
-236.136	236.136				
-201.641	201.641	+	201.645	0.0377787	973.6289447
		+	206.483	0.0349234	973.6315265
-206.477	206.477				
-24.738	24.738	+	247.399	0.0203393	973.6978599
		+	204.288	0.0363422	975.6069745
-204.283	204.283				
-215.354	215.354	+	215.361	0.0305104	976.6094488
		+	215.456	0.0304	976.6108009
-215.449	215.449				
-255.613	255.613	+	255.637	0.0188842	977.6132783
		+	217.415	0.0292127	978.5075049
-217.407	217.407				
-209.672	209.672	+	209.678	0.0334201	978.5120384
		+	223.951	0.0263605	978.6255995
-223.941	223.941				
-266.419	266.419	+	266.452	0.0182496	978.6385393
		+	258.725	0.0182	978.6415913
-258.699	258.699				
-1.9	1.9	+	190.003	0.0449332	987.6938550
		+	265.421	0.0186353	992.5963556
-265.389	265.389				
-258.451	258.451	+	258.477	0.0180936	993.8820289
		+	236.869	0.0231814	994.0751667
-236.855	236.855				
-185.078	185.078	+	185.081	0.0495927	994.6129757
		+	270.395	0.016711	994.7165265
-270.358	270.358				
-229.204	229.204	+	229.214	0.0246197	995.7190079
		+	229.219	0.0246401	995.7208011
-229.208	229.208				
-249.102	249.102	+	249.121	0.0191355	996.7322653
		+	313.488	0.014537	997.5623420
-313.351	313.351				
-317.809	317.809	+	317.967	0.0144724	997.5884212

		+	316.588	0.0142574	997.5913256
-316.437	316.437				
-263.488	263.488	+	263.518	0.0191169	997.7355615
		+	307.386	0.013254	998.5687808
-307.272	307.272				
-28.298	28.298	+	283.035	0.0151982	998.5727114
		+	239.006	0.0224503	998.5923940
-238.991	238.991				
-275.729	275.729	+	275.773	0.0158121	999.5762137
		+	230.645	0.0242021	1004.3384250
-230.634	230.634				
-232.773	232.773	+	232.785	0.023426	1004.3407390
		+	350.846	0.00926531	1006.6018800
-350.402	350.402				
-195.211	195.211	+	195.215	0.0412101	1011.6747380
		+	254.941	0.0188901	1012.3049380
-254.918	254.918				
-200.084	200.084	+	200.089	0.0387614	1012.6787940
		+	200.824	0.0384344	1012.6810560
-20.082	20.082				
-258.829	258.829	+	258.856	0.0181062	1013.3079990
		+	206.461	0.0349404	1013.5839290
-206.456	206.456				
-194.869	194.869	+	194.872	0.041444	1013.6893050
		+	194.925	0.0414358	1013.6908940
-194.921	194.921				
-19.656	19.656	+	196.564	0.0403752	1014.5880370
		+	200.371	0.0385795	1014.5916040
-200.366	200.366				
-201.162	201.162	+	201.166	0.0382155	1014.6939730
		+	192.184	0.0435938	1015.5991450
-19.218	19.218				
-192.212	192.212	+	192.215	0.0436337	1015.6004530
		+	190.424	0.0447683	1015.6723980
-19.042	19.042				
-218.396	218.396	+	218.404	0.028438	1016.5992540
		+	216.604	0.0299454	1016.6028570
-216.597	216.597				
-208.099	208.099	+	208.105	0.0342333	1016.6182940

		+	186.915	0.0479695	1016.6219180
-186.912	186.912				
-293.001	293.001	+	293.074	0.0148588	1017.6054350
		+	206.322	0.0349546	1019.6389840
-206.316	206.316				
-205.567	205.567	+	205.572	0.0352102	1019.6411990
		+	239.219	0.022141	1020.6232090
-239.205	239.205				
-204.721	204.721	+	204.726	0.0358025	1020.6449910
		+	233.845	0.0238687	1024.3725750
-233.832	233.832				
-221.249	221.249	+	221.258	0.027076	1025.5924600
		+	217.758	0.0291168	1030.5992720
-21.775	21.775				
-217.436	217.436	+	217.443	0.0292324	1030.6007910
		+	196.089	0.0405349	1031.3624820
-196.085	196.085				
-206.964	206.964	+	206.969	0.0347098	1032.3689430
		+	206.754	0.0348161	1032.3710090
-206.749	206.749				
-209.564	209.564	+	20.957	0.0335207	1035.5654950
		+	346.699	0.00958491	1035.6366290
-34.631	34.631				
-202.444	202.444	+	202.449	0.0371021	1036.5688270
		+	197.143	0.0401435	1036.5722660
-197.139	197.139				
-218.478	218.478	+	218.486	0.0283887	1037.6545290
		+	19.227	0.0435215	1038.6576140
-192.267	192.267				
-196.815	196.815	+	196.819	0.040231	1039.5873540
		+	213.442	0.0310282	1040.1622890
-213.436	213.436				
-219.208	219.208	+	219.216	0.0278412	1041.6040450
		+	218.533	0.0283393	1041.6238830
-218.525	218.525				
-255.911	255.911	+	255.935	0.0187792	1044.3288830
		+	252.108	0.018774	1044.3317930

-252.086	252.086				
-329.376	329.376	+	329.603	0.0103226	1046.3545470
		+	256.859	0.0189314	1046.6589330
-256.834	256.834				
-260.974	260.974	+	261.002	0.0189953	1046.6619800
		+	220.889	0.0272305	1046.6711630
-220.881	220.881				
-236.681	236.681	+	236.695	0.0233591	1047.3574670
		+	260.013	0.0188282	1047.6631760
-259.985	259.985				
-251.617	251.617	+	251.638	0.0189422	1047.6728290
		+	245.033	0.0213211	1048.6756290
-245.016	245.016				
-326.083	326.083	+	326.287	0.0126265	1056.6152580
		+	243.146	0.0220088	1057.6187660
-243.129	243.129				
-241.468	241.468	+	241.484	0.0216979	1057.6213160
		+	339.754	0.01	1058.6275440
-339.443	339.443				
-278.703	278.703	+	278.751	0.0155011	1058.6319020
		+	230.921	0.0239173	1059.6351690
-23.091	23.091				
-195.556	195.556	+	19.556	0.0410911	1065.6825490
		+	297.163	0.0145047	1068.5734960
-297.079	297.079				
-2.637	2.637	+	263.731	0.0192104	1071.3487670
		+	276.094	0.0156972	1071.3518920
-27.605	27.605				
-231.006	231.006	+	231.017	0.0238271	1072.3585290
		+	232.024	0.0236271	1072.3608060
-232.012	232.012				
-196.795	196.795	+	196.799	0.0402875	1073.3630230
		+	188.621	0.0461664	1075.5319000
-188.618	188.618				
-188.785	188.785	+	188.788	0.0458957	1076.5590120
		+	189.124	0.0455955	1076.5619680
-189.121	189.121				
-329.732	329.732	+	329.961	0.0105263	1078.5931450

		+	326.615	0.012097	1079.5980980
-326.409	326.409				
-335.195	335.195	+	335.467	0.00879433	1079.6010630
		+	440.284	0.00666667	1084.6428010
-432.845	432.845				
-33.768	33.768	+	337.975	0.00946565	1085.6468260
		+	225.367	0.026058	1085.7128920
-225.357	225.357				
-297.928	297.928	+	298.014	0.014327	1086.3433710
		+	247.169	0.0202667	1086.6575390
-24.715	24.715				
-254.116	254.116	+	254.139	0.0186937	1086.6607990
		+	277.768	0.0155556	1087.3468270
-277.722	277.722				
-306.177	306.177	+	306.287	0.0128462	1087.6631840
		+	2.695	0.0166809	1087.7283810
-269.464	269.464				
-270.576	270.576	+	270.613	0.016651	1087.7307240
		+	208.307	0.0341239	1088.7321230
-208.301	208.301				
-184.855	184.855	+	184.858	0.0497338	1092.6717480
		+	304.922	0.0134908	1096.6048580
-304.816	304.816				
-187.973	187.973	+	187.976	0.0467062	1099.6908430
		+	192.774	0.042778	1104.6120010
-19.277	19.277				
-306.492	306.492	+	306.604	0.0129961	1105.6142770
		+	241.892	0.0217768	1106.4080470
-241.876	241.876				
-304.371	304.371	+	304.475	0.0133431	1106.6246620
		+	340.553	0.00934426	1107.6276480
-340.234	340.234				
-305.542	305.542	+	30.565	0.0129925	1107.6311370
		+	206.749	0.0347964	1108.3256700
-206.744	206.744				
-204.501	204.501	+	204.506	0.0362974	1108.6387810
		+	204.152	0.0363373	1108.6409660
-204.147	204.147				
-208.614	208.614	+	20.862	0.0340233	1109.6438000

		+	289.406	0.0147766	1111.6185970
-28.934	28.934				
-296.058	296.058	+	296.139	0.0148049	1111.6214080
		+	227.369	0.025141	1112.6228660
-227.359	227.359				
-214.429	214.429	+	214.436	0.031041	1114.5169920
		+	492.235	0	1114.5219960
-481.834	481.834				
-192.249	192.249	+	192.252	0.0435474	1118.5860110
		+	211.549	0.0315937	1122.6750480
-211.543	211.543				
-218.716	218.716	+	218.724	0.0283186	1125.7052250
		+	235.617	0.0232955	1126.6164720
-235.604	235.604				
-234.051	234.051	+	234.063	0.023707	1126.6208560
		+	265.845	0.0187614	1127.6181520
-265.812	265.812				
-281.154	281.154	+	281.206	0.0151297	1127.6216960
		+	208.333	0.0341437	1127.7190800
-208.327	208.327				
-210.066	210.066	+	210.072	0.0331255	1127.7212950
		+	29.105	0.0147956	1128.7243750
-290.981	290.981				
-255.521	255.521	+	255.545	0.018993	1130.7487030
		+	251.515	0.0188937	1130.7509800
-251.494	251.494				
-258.024	258.024	+	25.805	0.0185349	1132.7445120
		+	261.374	0.0191146	1142.5864090
-261.346	261.346				
-203.205	203.205	+	20.321	0.0366699	1143.5926240
		+	244.304	0.0214796	1144.5972250
-244.287	244.287				
-218.958	218.958	+	218.966	0.0280695	1144.6032350
		+	199.269	0.0391411	1145.6069340
-199.265	199.265				
-191.042	191.042	+	191.045	0.0443118	1145.6114480
		+	224.221	0.0263405	1146.6160600

-224.212	224.212				
-197.798	197.798	+	197.803	0.0398375	1147.6866410
		+	21.772	0.0290971	1148.3173350
-217.713	217.713				
-219.643	219.643	+	219.651	0.0278145	1148.6330310
		+	209.353	0.0337042	1149.6989040
-209.347	209.347				
-211.228	211.228	+	211.234	0.03185	1149.7026100
		+	185.278	0.0495461	1150.6028860
-185.275	185.275				
-235.528	235.528	+	235.541	0.0233239	1150.7060390
		+	205.779	0.035147	1151.6093670
-205.774	205.774				
-205.814	205.814	+	205.819	0.0351304	1151.6116850
		+	215.642	0.0303266	1152.6148730
-215.635	215.635				
-211.779	211.779	+	211.785	0.0315296	1153.6176330
		+	246.631	0.0203542	1153.6335770
-246.613	246.613				
-211.539	211.539	+	211.546	0.0315745	1154.6468740
		+	214.924	0.0307915	1154.6510330
-214.917	214.917				
-285.354	285.354	+	285.412	0.0143865	1155.6483060
		+	276.643	0.0160163	1155.6520550
-276.598	276.598				
-223.698	223.698	+	223.707	0.026406	1156.7652930
		+	366.706	0.00921212	1158.7791260
-365.955	365.955				
-365.074	365.074	+	365.802	0.00894118	1158.7814640
		+	516.518	0	1159.7844130
-512.825	512.825				
-213.016	213.016	+	213.023	0.0310827	1166.6091900
		+	212.831	0.0310501	1166.6114110
-212.824	212.824				
-191.762	191.762	+	191.765	0.0437641	1167.6087330
		+	225.726	0.0258738	1167.6144190
-225.716	225.716				
-214.126	214.126	+	214.133	0.0312836	1173.5932240
		+	212.866	0.0309672	1174.5966460

-212.859	212.859				
-220.212	220.212	+	22.022	0.0276322	1189.5957730
		+	29.859	0.0145096	1194.6390110
-298.503	298.503				
-304.318	304.318	+	304.422	0.0132945	1194.6423550
-28.432	28.432	+	284.377	0.0146355	1195.6457200

Supplementary Table S2: Posthoc test HCT-8 for infected cells in negative-ion mode

Control	Infected	C: ANOVA Significant	N: -Log ANOVA p value	N: ANOVA q-value	MALDI m/z
		+	344.386	0.00191087	252.0286090
-179.785	179.785	+	179.787	0.0434725	253.0979338
		+	257.863	0.011083	258.4364192
-257.838	257.838				
-270.512	270.512	+	27.055	0.00794059	261.0156458
		+	191.572	0.0352225	264.0261131
-191.568	191.568				
-252.504	252.504	+	252.526	0.0121023	268.2366776
		+	215.579	0.0236064	271.0249145
-215.572	215.572				
-180.585	180.585	+	180.588	0.0430621	271.0366794
		+	233.258	0.0172317	275.5749191
-233.246	233.246				
-187.287	187.287	+	18.729	0.0375515	280.0345327
		+	320.402	0.00300901	280.2363495
-320.232	320.232				
-193.284	193.284	+	193.287	0.0339901	282.2438263
		+	211.836	0.0255986	285.0384969
-211.829	211.829				
-185.146	185.146	+	185.149	0.0389169	289.0292097
		+	256.192	0.011664	289.0470701
-256.168	256.168				
-19.204	19.204	+	192.044	0.0349557	296.0287298
		+	249.844	0.0128231	297.0381067

-249.824	249.824				
-289.844	289.844	+	289.911	0.00527888	298.2467429
		+	262.784	0.00970245	301.0222241
-262.755	262.755				
-32.022	32.022	+	320.389	0.0030045	301.0456565
		+	281.066	0.00649892	301.2172010
-281.014	281.014				
-219.626	219.626	+	219.634	0.0215773	303.2328955
		+	345.113	0.00192115	304.2361879
-344.743	344.743				
-28.425	28.425	+	284.307	0.00622958	305.2485472
		+	205.434	0.0280066	311.0553850
-205.429	205.429				
-184.215	184.215	+	184.218	0.0398466	312.0454147
-199.507	199.507				
-344.025	344.025	+	199.512	0.0303933	251.2023165

		+	191.366	0.035372	313.1731354
-191.362	191.362				
-199.948	199.948	+	199.953	0.0302842	315.2539707
		+	174.332	0.0476077	317.0408295
-17.433	17.433				
-211.393	211.393	+	211.399	0.0257681	321.0464278
		+	218.296	0.02218	326.0426850
-218.288	218.288				
-272.395	272.395	+	272.435	0.00745838	326.0523488
		+	226.732	0.0189675	327.2329824
-226.722	226.722				
-306.606	306.606	+	306.718	0.00376032	329.2485741
		+	201.699	0.0297396	330.2438810
-201.694	201.694				
-250.035	250.035	+	250.055	0.0127715	331.2390335
		+	178.886	0.044079	332.0271794
-178.884	178.884				
-205.316	205.316	+	205.321	0.0279448	335.0430584
		+	202.913	0.0292491	339.3265704
-202.908	202.908				
-175.814	175.814	+	175.816	0.0465919	341.0374941
		+	182.997	0.040776	342.0454789
-182.995	182.995				
-257.559	257.559	+	257.584	0.0110332	342.2259587
		+	182.582	0.0409106	343.0482240
-182.579	182.579				
-267.679	267.679	+	267.714	0.00836965	344.2957510
		+	324.047	0.00276074	347.0429663
-323.857	323.857				
-179.112	179.112	+	179.114	0.0440103	348.0560759
		+	297.074	0.00417822	354.0477667
-29.699	29.699				
-198.617	198.617	+	198.621	0.0307705	354.1444396
		+	194.689	0.033251	355.0408716
-194.685	194.685				
-187.171	187.171	+	187.174	0.03759	356.0442340
		+	187.431	0.0375227	358.0393550

-187.427	187.427				
-238.878	238.878	+	238.893	0.0153007	359.0427678
		+	207.716	0.026807	360.2543851
-20.771	20.771				
-175.461	175.461	+	175.463	0.0467859	362.0437593
		+	186.174	0.0381321	362.2707854
-186.171	186.171				
-192.296	192.296	+	192.299	0.0348954	363.0372421
		+	185.215	0.0388158	363.0618614
-185.212	185.212				
-196.305	196.305	+	196.309	0.0319898	364.0431590
		+	295.819	0.0044143	365.3420088
-295.739	295.739				
-215.332	215.332	+	21.534	0.0239148	367.0450984
		+	198.303	0.0309607	367.3577025
-198.298	198.298				
-248.286	248.286	+	248.306	0.013303	368.1615468
		+	192.622	0.0347388	368.3614491
-192.618	192.618				
-1.932	1.932	+	193.203	0.0340662	369.0240902
		+	172.472	0.0492817	374.0655213
-17.247	17.247				
-193.586	193.586	+	19.359	0.0338721	375.0359686
		+	198.277	0.0309902	376.0451254
-198.273	198.273				
-172.301	172.301	+	172.303	0.0492508	376.2259635
		+	184.999	0.0393155	382.1753038
-184.996	184.996				
-24.489	24.489	+	244.907	0.013815	384.1929519
		+	191.458	0.0353882	385.1873320
-191.455	191.455				
-172.435	172.435	+	172.437	0.0492024	388.2854437
		+	316.667	0.00339623	394.0440419
-316.516	316.516				
-212.092	212.092	+	212.098	0.0255703	394.1631332
		+	186.554	0.0379218	395.0570056

-186.551	186.551				
-213.668	213.668	+	213.675	0.0248642	395.3888422
		+	197.163	0.0317481	397.0554485
-197.159	197.159				
-203.535	203.535	+	20.354	0.0288838	397.1026185
-23.596	23.596	+	235.974	0.0165689	402.1436004
		+	299.069	0.00401011	404.0514405
-29.898	29.898				
-242.385	242.385	+	242.401	0.014639	409.0448104
		+	182.352	0.041071	410.1565979
-18.235	18.235				
-218.858	218.858	+	218.866	0.0219975	419.0615310
-212.772	212.772	+	212.779	0.025081	421.0772280
		+	191.142	0.035645	422.0947273
-191.138	191.138				
-27.308	27.308	+	27.312	0.00750353	423.2879060
		+	210.888	0.0260969	431.0724753
-210.882	210.882				
-215.748	215.748	+	215.755	0.0234051	433.2712757
		+	222.664	0.0205893	434.0723418
-222.655	222.655				
-173.302	173.302	+	173.304	0.0480633	435.2866905
		+	193.833	0.0336905	436.0652007
-193.829	193.829				
-187.954	187.954	+	187.957	0.0373513	437.0657902
		+	190.225	0.0360153	437.0728556
-190.222	190.222				
-20.845	20.845	+	208.456	0.0266999	437.2312944
		+	345.963	0.00193852	440.0477582
-345.584	345.584				
-287.834	287.834	+	287.897	0.00557548	442.2325483
		+	176.435	0.0462176	445.2517059
-176.433	176.433				
-214.335	214.335	+	214.342	0.0244958	446.0838084
		+	182.731	0.0408552	446.1838357
-182.728	182.728				
-190.439	190.439	+	190.443	0.0358736	449.0832511

		+	292.382	0.00503704	450.1152006
-29.231	29.231				
-210.201	210.201	+	210.208	0.0263717	452.1670257
		+	217.402	0.0222737	452.2855385
-217.395	217.395				
-221.679	221.679	+	221.688	0.0209096	453.0677793
		+	214.865	0.024017	454.0826905
-214.858	214.858				
-264.157	264.157	+	264.188	0.00917557	454.2717852
		+	178.388	0.0445363	455.0630669
-178.386	178.386				
-250.455	250.455	+	250.476	0.0127342	457.0766131
		+	208.199	0.0266335	457.2361694
-208.193	208.193				
-244.434	244.434	+	244.451	0.0141507	457.2685864
		+	232.882	0.0173773	459.0685040
-23.287	23.287				
-183.445	183.445	+	183.448	0.0404594	459.2030792
		+	194.016	0.0334594	459.2148017
-194.012	194.012				
-358.528	358.528	+	35.911	0.00113163	459.2844377
		+	201.252	0.0301576	460.2440610
-201.247	201.247				
-185.558	185.558	+	185.561	0.0384266	462.0783052
		+	299.303	0.00402028	463.0818600
-299.214	299.214				
-255.284	255.284	+	255.308	0.0117898	466.2947810
		+	211.314	0.025738	469.2922248
-211.308	211.308				
-210.691	210.691	+	210.697	0.0261854	470.2642521
		+	222.128	0.0208482	471.2672979
-222.119	222.119				
-218.245	218.245	+	218.253	0.0221523	472.2793714
		+	2.258	0.0196231	472.2808406
-22.579	22.579				
-174.546	174.546	+	174.548	0.0474758	473.0637550
		+	246.242	0.0137207	474.2632691

-246.224	246.224				
-184.307	184.307	+	18.431	0.0397254	476.2788830
		+	224.505	0.0201143	476.2809102
-224.495	224.495				
-175.076	175.076	+	175.078	0.0469476	477.1174234
		+	269.621	0.00805108	477.2831977
-269.585	269.585				
-204.231	204.231	+	204.236	0.0283738	478.0737026
		+	179.796	0.0435102	478.1855635
-179.794	179.794				
-214.419	214.419	+	214.426	0.0243568	479.0761262
		+	180.613	0.0430389	479.2554781
-180.611	180.611				
-273.084	273.084	+	273.124	0.00751111	479.2837354
		+	172.876	0.0488236	479.3137220
-172.874	172.874				
-19.884	19.884	+	198.844	0.0308025	480.0868199
		+	226.726	0.0190142	481.1737436
-226.716	226.716				
-249.718	249.718	+	249.738	0.0129769	482.2661433
		+	264.301	0.00918434	482.2858710
-26.427	26.427				
-176.938	176.938	+	176.941	0.0459815	483.0687828
		+	25.661	0.0114769	483.0953463
-256.586	256.586				
-241.527	241.527	+	241.543	0.0149352	483.2153997
		+	181.425	0.0421058	484.2829627
-181.423	181.423				
-22.104	22.104	+	221.049	0.0212169	486.0679080
		+	390.344	0.000655087	488.1857406
-38.865	38.865				
-202.968	202.968	+	202.973	0.0292965	488.1934750
		+	192.106	0.0349727	489.1869049
-192.103	192.103				
-289.183	289.183	+	289.249	0.00554839	489.2130254
		+	214.091	0.024617	490.2077683
-214.084	214.084				
-172.771	172.771	+	172.773	0.0488595	490.2116515

		+	304.422	0.00417368	490.2843663
-304.318	304.318				
-190.874	190.874	+	190.877	0.0357132	493.0626796
		+	178.897	0.0441358	493.1743656
-178.895	178.895				
-206.827	206.827	+	206.833	0.0273248	493.3185703
		+	181.545	0.0419735	494.2663228
-181.543	181.543				
-21.882	21.882	+	218.827	0.0219837	495.2741718
		+	203.206	0.0291751	496.3187074
-203.201	203.201				
-251.103	251.103	+	251.124	0.0125452	498.2635084
		+	263.099	0.00967424	499.0653453
-263.069	263.069				
-183.696	183.696	+	183.699	0.0403432	499.0775144
		+	283.742	0.00628509	499.1850689
-283.687	283.687				
-244.041	244.041	+	244.058	0.0142219	500.0742315
		+	317.764	0.00330205	500.2790249
-317.607	317.607				
-313.819	313.819	+	313.959	0.0035983	500.2803651
		+	332.926	0.00233061	501.2823736
-332.675	332.675				
-179.204	179.204	+	179.207	0.0439758	502.0768275
		+	205.177	0.0280905	502.1859905
-205.172	205.172				
-242.674	242.674	+	24.269	0.0146728	502.2079344
		+	332.285	0.002323	502.2112457
-332.039	332.039				
-344.737	344.737	+	345.107	0.00191771	502.2947278
		+	194.204	0.0334406	504.2257120
-1.942	1.942				
-177.797	177.797	+	177.799	0.0450021	504.2366957
		+	206.482	0.0275682	506.1646329
-206.476	206.476				
-203.594	203.594	+	203.599	0.0288674	506.2048779
		+	309.253	0.00370149	506.3263029

**-309.132**	**309.132**				
**-178.115**	**178.115**	+	**178.118**	**0.0446689**	**507.3277019**
		+	**208.112**	**0.0266035**	**508.2330896**
**-208.107**	**208.107**				
**-227.088**	**227.088**	+	**227.098**	**0.0189735**	**509.2534894**
		+	**17.392**	**0.0478302**	**510.1052925**
**-173.918**	**173.918**				
**-196.227**	**196.227**	+	**196.232**	**0.0319574**	**510.1853454**
		+	**262.819**	**0.00972075**	**511.1464472**
**-262.789**	**262.789**				
**-176.019**	**176.019**	+	**176.022**	**0.0464426**	**511.1525940**
		+	**173.754**	**0.0477881**	**511.1743064**
**-173.752**	**173.752**				
**-220.661**	**220.661**	+	**22.067**	**0.0212388**	**511.1856464**
		+	**425.528**	**0**	**514.2580500**
**-420.312**	**420.312**				
**-209.877**	**209.877**	+	**209.883**	**0.0263576**	**514.3286818**
		+	**219.657**	**0.0215909**	**515.2163899**
**-219.649**	**219.649**				
**-291.585**	**291.585**	+	**291.656**	**0.00511164**	**516.2022900**
		+	**261.416**	**0.0101547**	**518.1939730**
**-261.387**	**261.387**				
**-219.673**	**219.673**	+	**219.681**	**0.0216045**	**518.2050732**
		+	**265.681**	**0.00881617**	**518.2928854**
**-265.649**	**265.649**				
**-276.242**	**276.242**	+	**276.286**	**0.00744756**	**519.3605714**
		+	**19.422**	**0.033474**	**521.0848335**
**-194.217**	**194.217**				
**-321.534**	**321.534**	+	**321.711**	**0.00304097**	**521.1063590**
		+	**221.571**	**0.0209529**	**521.3761269**
**-221.562**	**221.562**				
**-17.684**	**17.684**	+	**176.843**	**0.0460496**	**522.2977393**
		+	**206.777**	**0.0272942**	**522.3215031**
**-206.771**	**206.771**				
**-224.429**	**224.429**	+	**224.438**	**0.0200743**	**522.3791916**
		+	**221.527**	**0.0209871**	**522.3804288**
**-221.518**	**221.518**				
**-182.055**	**182.055**	+	**182.058**	**0.0413357**	**523.1549555**

		+	325.252	0.00280374	524.2648989
-325.055	325.055				
-290.717	290.717	+	290.786	0.00506353	524.3141182
		+	256.913	0.0112199	526.1007445
-256.888	256.888				
-217.342	217.342	+	21.735	0.0222599	526.2765355
		+	216.656	0.022688	528.0829141
-216.649	216.649				
-179.878	179.878	+	179.881	0.0434909	528.2872807
		+	198.363	0.0309767	530.2538940
-198.358	198.358				
-195.495	195.495	+	195.499	0.0325809	530.2776778
		+	182.397	0.0410466	533.2159435
-182.394	182.394				
-202.046	202.046	+	20.205	0.0296021	533.2920146
		+	410.444	0.000260355	533.3075573
-407.111	407.111				
-173.485	173.485	+	173.487	0.0479285	534.2022063
		+	418.426	0.000278481	534.2468712
-414.163	414.163				
-223.799	223.799	+	223.808	0.0200368	535.3054088
		+	174.128	0.0477133	535.3552911
-174.126	174.126				
-236.982	236.982	+	236.995	0.0161898	536.0957200
		+	25.816	0.0110435	536.2937434
-258.134	258.134				
-267.837	267.837	+	267.871	0.00830019	536.3015330
		+	229.374	0.0180849	537.2735003
-229.363	229.363				
-232.595	232.595	+	232.607	0.0174535	537.2844996
		+	197.899	0.0313423	537.3088246
-197.894	197.894				
-177.034	177.034	+	177.036	0.0459376	537.3713175
		+	308.464	0.0036716	538.1119966
-308.346	308.346				
-173.023	173.023	+	173.025	0.0486429	538.2453672
		+	257.335	0.0110877	539.3247503

-25.731	25.731				
-314.048	314.048	+	314.188	0.0036034	540.2363719
		+	342.087	0.00188732	540.2854520
-341.752	341.752				
-213.583	213.583	+	21.359	0.0248196	541.1117228
		+	222.965	0.0203945	541.1959776
-222.956	222.956				
-192.161	192.161	+	192.165	0.0348571	541.2966026
		+	171.717	0.0499744	542.0722082
-171.715	171.715				
-32.094	32.094	+	321.113	0.00302719	542.0956328
		+	282.165	0.00630553	542.2164823
-282.112	282.112				
-304.523	304.523	+	304.628	0.00403166	543.2563653
		+	176.642	0.0461629	543.2966148
-17.664	17.664				
-202.329	202.329	+	202.334	0.029532	544.1853084
		+	225.536	0.0197905	545.2929680
-225.526	225.526				
-181.812	181.812	+	181.815	0.0415639	546.0768064
		+	275.803	0.00741675	546.2957995
-275.759	275.759				
-210.368	210.368	+	210.374	0.026232	547.0985300
		+	255.965	0.0116434	547.2241209
-255.941	255.941				
-242.946	242.946	+	242.962	0.0145604	547.2430862
		+	173.154	0.0484214	548.1085314
-173.152	173.152				
-191.774	191.774	+	191.777	0.0351211	548.1933517
		+	334.128	0.00190789	548.3115170
-333.868	333.868				
-376.862	376.862	+	377.964	0.000816143	549.1238202
		+	222.982	0.0204078	549.2127859
-222.973	222.973				
-182.981	182.981	+	182.984	0.0407971	549.2676775
		+	238.387	0.015883	550.1343947
-238.373	238.373				
-260.938	260.938	+	260.966	0.01029	550.2336154

		+	217.988	0.0220971	550.2444849
-21.798	21.798				
-338.698	338.698	+	339.002	0.00183248	552.0889841
		+	32.437	0.00276498	552.0912533
-324.178	324.178				
-204.627	204.627	+	204.632	0.0282542	552.3323298
		+	215.011	0.0240219	553.0978371
-215.004	215.004				
-286.408	286.408	+	286.468	0.00587919	553.1232180
		+	239.017	0.0153238	553.3045889
-239.002	239.002				
-176.701	176.701	+	176.703	0.0461336	553.3663193
		+	416.199	0.000274143	554.1055482
-412.211	412.211				
-298.782	298.782	+	29.887	0.004	554.1655953
		+	209.992	0.0263728	556.1223098
-209.986	209.986				
-187.545	187.545	+	187.548	0.0375477	556.2228960
		+	236.736	0.016195	556.3186408
-236.723	236.723				
-236.431	236.431	+	236.445	0.0162121	557.3355798
		+	288.793	0.0055103	558.1851691
-288.729	288.729				
-198.476	198.476	+	198.481	0.0307227	558.3159301
		+	173.974	0.0477417	558.3227937
-173.972	173.972				
-222.903	222.903	+	222.912	0.0204253	559.2847555
		+	331.954	0.00231545	559.2934766
-331.711	331.711				
-21.827	21.827	+	218.278	0.0221661	559.3043433
		+	198.139	0.0311251	559.3313145
-198.135	198.135				
-239.569	239.569	+	239.584	0.0152309	560.0955086
		+	3.339	0.00204926	560.2965667
-333.642	333.642				
-176.028	176.028	+	17.603	0.0464253	560.3370295
		+	348.073	0.00174135	562.2431586

-347.667	347.667				
-307.272	307.272	+	307.386	0.00378552	562.3159277
		+	178.832	0.0442075	563.1304497
-17.883	17.883				
-323.295	323.295	+	323.482	0.00274809	563.2846548
		+	266.753	0.00850775	563.3012299
-266.719	266.719				
-175.363	175.363	+	175.366	0.0467719	564.1137723
		+	187.521	0.0375574	564.2132488
-187.518	187.518				
-178.641	178.641	+	178.643	0.0443132	564.3331814
		+	219.764	0.0216593	564.3683880
-219.756	219.756				
-22.944	22.944	+	229.451	0.0180614	565.1457695
		+	316.382	0.00337662	565.3155963
-316.231	316.231				
-263.097	263.097	+	263.127	0.00968341	566.1271635
		+	186.321	0.0380735	566.2044549
-186.318	186.318				
-224.473	224.473	+	224.482	0.0200876	566.3474318
		+	19.252	0.0347308	567.1012415
-192.516	192.516				
-243.472	243.472	+	243.488	0.0141116	567.2137712
		+	208.565	0.0265795	567.2357957
-208.559	208.559				
-177.846	177.846	+	177.848	0.044972	567.2824390
		+	174.758	0.0471362	567.3229253
-174.756	174.756				
-179.592	179.592	+	179.595	0.0437508	567.3322523
		+	207.503	0.0269534	568.2936459
-207.497	207.497				
-309.748	309.748	+	309.871	0.00374211	568.3265962
		+	214.417	0.0244072	569.1168529
-21.441	21.441				
-300.248	300.248	+	30.034	0.00404076	569.3023042
		+	186.495	0.0379448	569.3615036
-186.492	186.492				
-449.833	449.833	+	460.029	0	570.0989352

		+	359.702	0.00114286	570.1011343
-359.108	359.108				
-229.199	229.199	+	22.921	0.0182306	570.1233190
		+	201.534	0.0299968	570.1377863
-201.529	201.529				
-331.924	331.924	+	332.169	0.00231922	570.2243306
		+	272.892	0.00748089	570.2470244
-272.852	272.852				
-301.723	301.723	+	301.819	0.0040929	570.2740296
		+	260.235	0.0105222	570.3083298
-260.207	260.207				
-256.376	256.376	+	256.401	0.0116025	571.2445261
		+	215.426	0.0237901	571.2782515
-215.419	215.419				
-174.781	174.781	+	174.783	0.0471193	571.2875518
		+	341.743	0.00187741	571.2910907
-341.412	341.412				
-36.681	36.681	+	367.583	0.00120251	571.3155356
		+	324.877	0.00279503	572.1161468
-324.682	324.682				
-309.211	309.211	+	309.332	0.00370652	572.3025433
		+	202.535	0.0294655	574.3152704
-20.253	20.253				
-268.054	268.054	+	268.088	0.00822244	575.2459902
		+	201.299	0.0301737	575.3251335
-201.295	201.295				
-380.469	380.469	+	381.724	0.0006097	575.3367691
		+	277.259	0.00720755	576.2948790
-277.213	277.213				
-289.191	289.191	+	289.257	0.00555479	576.3325487
		+	199.628	0.0303762	576.3684604
-199.623	199.623				
-249.396	249.396	+	249.416	0.0130123	577.1088729
		+	272.985	0.00749597	577.1113807
-272.945	272.945				
-398.144	398.144	+	40.055	0.000238482	577.3025515
		+	252.359	0.0121633	577.3162348

-252.338	252.338				
-1.974	1.974	+	197.404	0.0317351	578.0947926
		+	179.179	0.0439845	579.1248718
-179.176	179.176				
-241.137	241.137	+	241.153	0.0149453	580.1072616
		+	225.275	0.0197242	580.2440108
-225.266	225.266				
-227.888	227.888	+	227.898	0.0185202	580.3637606
		+	17.206	0.049651	582.3148324
-172.058	172.058				
-25.041	25.041	+	250.431	0.0128033	583.0961049
		+	210.468	0.0261799	584.0779477
-210.462	210.462				
-190.542	190.542	+	190.546	0.0358352	584.2168357
		+	191.218	0.0355232	584.7441336
-191.214	191.214				
-230.971	230.971	+	230.982	0.0177677	585.1119682
		+	206.478	0.0275528	585.3087715
-206.472	206.472				
-289.647	289.647	+	289.714	0.00549767	585.3439248
		+	177.713	0.0450793	586.2661049
-177.711	177.711				
-341.645	341.645	+	341.979	0.00188401	587.2364288
		+	179.647	0.0436412	588.2839213
-179.645	179.645				
-22.772	22.772	+	227.731	0.0185208	589.3421990
		+	175.106	0.046967	590.3120233
-175.104	175.104				
-202.311	202.311	+	202.316	0.0294844	590.3477523
		+	19.479	0.0331431	591.0876647
-194.786	194.786				
-177.329	177.329	+	177.331	0.0454117	591.3332242
		+	282.731	0.00623721	592.3268394
-282.677	282.677				
-259.014	259.014	+	25.904	0.0107799	593.1066907
		+	248.367	0.0133355	593.2158453
-248.348	248.348				
-320.524	320.524	+	320.695	0.00301807	594.2024189

		+	258.129	0.0110335	595.2915462
-258.103	258.103				
-183.081	183.081	+	183.083	0.0407309	595.3143398
		+	227.815	0.0184695	596.1068815
-227.805	227.805				
-176.331	176.331	+	176.334	0.0462191	596.2266586
		+	282.438	0.00621692	596.2372636
-282.385	282.385				
-237.239	237.239	+	237.253	0.0161517	597.2343381
		+	179.956	0.0435059	597.2627043
-179.954	179.954				
-212.264	212.264	+	21.227	0.0253569	597.3065103
		+	177.983	0.0448558	598.1128008
-17.798	17.798				
-187.889	187.889	+	187.893	0.037381	599.0878445
		+	188.191	0.0371357	599.0916267
-188.188	188.188				
-293.147	293.147	+	293.221	0.00497349	599.3222123
		+	230.923	0.0177551	600.2843685
-230.912	230.912				
-210.821	210.821	+	210.827	0.0260817	600.3074757
		+	180.107	0.0434946	601.1068324
-180.104	180.104				
-205.978	205.978	+	205.984	0.027594	601.3159148
		+	17.601	0.0464715	601.3262785
-176.008	176.008				
-244.532	244.532	+	24.455	0.014173	601.3423397
		+	215.817	0.0234482	602.3366850
-215.809	215.809				
-211.861	211.861	+	211.867	0.0256136	603.0932166
		+	193.558	0.0338553	603.3054887
-193.554	193.554				
-34.772	34.772	+	348.127	0.00174453	603.3338883
		+	262.939	0.00972993	603.3684301
-26.291	26.291				
-295.376	295.376	+	295.455	0.00464865	604.0982914
		+	295.114	0.0047335	604.3268780

-295.036	295.036				
-192.244	192.244	+	192.247	0.0348613	606.3448764
		+	213.218	0.0249822	607.1123039
-213.211	213.211				
-194.447	194.447	+	194.451	0.0333527	607.2431676
		+	231.934	0.017588	608.2149909
-231.922	231.922				
-194.451	194.451	+	194.455	0.0333694	608.2264717
		+	201.504	0.0300277	609.2050579
-201.499	201.499				
-219.621	219.621	+	219.629	0.0215637	609.2124473
		+	259.474	0.0106213	609.2235197
-259.447	259.447				
-296.182	296.182	+	296.263	0.00441975	609.2956144
		+	270.618	0.00794846	609.3056022
-270.581	270.581				
-176.274	176.274	+	176.276	0.0462747	610.1455015
		+	173.308	0.0480828	610.3168403
-173.306	173.306				
-189.733	189.733	+	189.737	0.0361933	610.4030919
		+	266.248	0.00856812	611.2368250
-266.215	266.215				
-176.008	176.008	+	17.601	0.0464522	611.3231126
		+	172.467	0.0492619	613.2059961
-172.465	172.465				
-201.038	201.038	+	201.042	0.0300923	613.2823199
		+	188.385	0.0369771	613.3023603
-188.382	188.382				
-362.755	362.755	+	363.427	0.00117312	614.2150456
		+	288.054	0.00558182	614.3358007
-287.991	287.991				
-189.423	189.423	+	189.426	0.0361923	615.2674414
		+	240.995	0.0149339	615.3061695
-240.979	240.979				
-207.922	207.922	+	207.928	0.0265735	615.3265047
		+	272.183	0.00742857	615.3336633
-272.144	272.144				
-231.945	231.945	+	231.957	0.0176006	615.3443307

		+	247.458	0.0135258	615.3683401
-247.439	247.439				
-220.509	220.509	+	220.518	0.0212755	616.2547597
		+	171.957	0.0497402	616.3159421
-171.955	171.955				
-193.542	193.542	+	193.545	0.0338385	617.0959143
		+	206.013	0.0276093	617.3223705
-206.007	206.007				
-212.727	212.727	+	212.734	0.0251182	617.3842645
		+	206.762	0.027279	618.2952554
-206.756	206.756				
-25.582	25.582	+	255.845	0.0116127	618.3318200
		+	272.796	0.00746586	618.4881892
-272.756	272.756				
-20.844	20.844	+	208.446	0.0266848	619.3264189
		+	199.778	0.0302699	620.3122985
-199.774	199.774				
-316.441	316.441	+	316.592	0.0033913	622.2256641
		+	172.569	0.0490887	622.2431543
-172.567	172.567				
-187.662	187.662	+	187.665	0.0375417	623.3132060
		+	3.212	0.00303177	624.2053544
-321.025	321.025				
-213.439	213.439	+	213.446	0.0248405	624.2138957
		+	179.037	0.0439725	624.2346827
-179.035	179.035				
-179.218	179.218	+	179.221	0.0440138	624.3060752
		+	237.879	0.016003	624.3824784
-237.865	237.865				
-17.345	17.345	+	173.452	0.047909	625.2036679
		+	21.056	0.0262408	625.2163021
-210.554	210.554				
-440.637	440.637	+	449.462	0	625.2868647
		+	356.155	0.00127692	626.2954876
-355.627	355.627				
-191.356	191.356	+	191.359	0.0353549	626.3981997
		+	244.938	0.0138259	627.2251077

-24.492	24.492				
-205.159	205.159	+	205.164	0.0281235	627.3432120
		+	207.116	0.0271462	628.4139637
-207.111	207.111				
-188.274	188.274	+	188.278	0.0370789	629.3128848
		+	24.988	0.0128337	629.3466306
-249.859	249.859				
-17.844	17.844	+	178.442	0.0444767	630.1422961
		+	207.746	0.0268221	631.3029600
-207.741	207.741				
-208.548	208.548	+	208.554	0.0265494	631.3639305
		+	245.703	0.0136988	632.3470015
-245.685	245.685				
-270.848	270.848	+	270.886	0.00767063	632.3582450
		+	389.433	0.000651852	633.2960411
-387.792	387.792				
-174.159	174.159	+	174.161	0.0477525	633.3158178
		+	177.389	0.0452893	634.2925239
-177.386	177.386				
-334.269	334.269	+	334.533	0.00191419	634.3031504
		+	188.445	0.0369668	635.3234493
-188.442	188.442				
-261.052	261.052	+	26.108	0.0102177	636.2228046
		+	306.085	0.00389404	636.3072588
-305.976	305.976				
-67.729	67.729	+	591.841	0	637.2270039
		+	182.728	0.040837	637.3270633
-182.726	182.726				
-292.533	292.533	+	292.606	0.00504306	637.3361564
		+	184.755	0.0395666	638.2372825
-184.752	184.752				
-192.029	192.029	+	192.032	0.0349387	638.2855216
		+	25.896	0.0107701	638.3232774
-258.934	258.934				
-1.877	1.877	+	187.703	0.0375766	639.3438357
		+	57.545	0	641.2262809
-629.599	629.599				
-218.382	218.382	+	21.839	0.0221388	641.3331495

		+	174.037	0.047761	642.2139687
-174.034	174.034				
-213.688	213.688	+	213.695	0.0248263	642.3056119
		+	281.236	0.00650592	642.3545094
-281.185	281.185				
-299.446	299.446	+	299.536	0.00402538	642.3932128
		+	176.546	0.0461937	643.1341962
-176.543	176.543				
-179.161	179.161	+	179.163	0.0439655	643.3974754
		+	177.394	0.045282	644.1846621
-177.392	177.392				
-18.138	18.138	+	181.383	0.0420873	644.4086331
		+	190.048	0.0360629	645.1511712
-190.045	190.045				
-223.924	223.924	+	223.933	0.0200632	645.3064970
		+	289.991	0.00517895	645.3430335
-289.924	289.924				
-250.964	250.964	+	250.985	0.012621	646.3140406
		+	198.127	0.031093	646.3473532
-198.122	198.122				
-214.403	214.403	+	21.441	0.0243925	646.3627601
		+	385.002	0.000621176	646.3745821
-383.595	383.595				
-277.562	277.562	+	277.608	0.00723789	647.1175881
		+	275.537	0.00749226	647.2148919
-275.494	275.494				
-206.679	206.679	+	206.684	0.0272486	647.3138953
		+	230.647	0.017705	647.3231861
-230.636	230.636				
-276.721	276.721	+	276.766	0.00725417	647.3337354
		+	229.217	0.0181821	648.2063122
-229.206	229.206				
-236.086	236.086	+	236.099	0.0164702	648.2451981
		+	242.946	0.0145491	648.3254932
-24.293	24.293				
-338.473	338.473	+	338.775	0.00182935	648.3437077
		+	178.892	0.0441168	649.2254668

**-17.889**	**17.889**				
**-193.387**	**193.387**	+	**193.391**	**0.0339535**	**649.2329751**
		+	**227.152**	**0.0189864**	**649.3368763**
**-227.142**	**227.142**				
**-202.538**	**202.538**	+	**202.543**	**0.029434**	**652.3029971**
		+	**237.933**	**0.0159432**	**653.1262350**
**-237.919**	**237.919**				
**-207.651**	**207.651**	+	**207.657**	**0.0268803**	**654.2335189**
		+	**227.578**	**0.0185683**	**654.3364514**
**-227.568**	**227.568**				
**-214.547**	**214.547**	+	**214.554**	**0.0242246**	**655.3039370**
		+	**219.292**	**0.0217621**	**655.3238863**
**-219.284**	**219.284**				
**-208.697**	**208.697**	+	**208.703**	**0.0265097**	**655.3382264**
		+	**266.068**	**0.00863645**	**656.3719171**
**-266.035**	**266.035**				
**-196.013**	**196.013**	+	**196.017**	**0.0321194**	**657.2333733**
		+	**172.365**	**0.0492904**	**657.5252865**
**-172.363**	**172.363**				
**-241.551**	**241.551**	+	**241.567**	**0.0149466**	**658.3877884**
		+	**262.107**	**0.00984817**	**658.4080639**
**-262.078**	**262.078**				
**-32.478**	**32.478**	+	**324.976**	**0.00279938**	**658.4108978**
		+	**307.278**	**0.00378046**	**659.1278628**
**-307.165**	**307.165**				
**-330.495**	**330.495**	+	**330.729**	**0.00230048**	**659.1308911**
		+	**363.361**	**0.00117073**	**659.3331302**
**-36.269**	**36.269**				
**-182.943**	**182.943**	+	**182.945**	**0.0407425**	**660.1364271**
		+	**172.873**	**0.0488039**	**660.4263594**
**-172.871**	**172.871**				
**-176.297**	**176.297**	+	**1.763**	**0.0462733**	**661.3032977**
		+	**249.626**	**0.0129555**	**661.3258010**
**-249.606**	**249.606**				
**-206.301**	**206.301**	+	**206.306**	**0.0275909**	**662.2255216**
-17.385	17.385	+	**173.852**	**0.0477912**	**662.3237802**
		+	**266.243**	**0.00855985**	**662.3579548**

-26.621	26.621				
-289.552	289.552	+	289.618	0.00548492	663.1620920
-211.535	211.535	+	211.541	0.0257832	663.3174400
		+	220.369	0.0213783	664.3741946
-220.361	220.361				
-273.627	273.627	+	273.668	0.00748269	665.1317576
		+	244.527	0.0141618	665.2256452
-24.451	24.451				
-252.816	252.816	+	252.838	0.0121645	665.2858765
		+	25.715	0.0111365	667.3377997
-257.125	257.125				
-25.535	25.535	+	255.374	0.0118002	667.3751828
		+	278.072	0.0070444	668.1252748
-278.025	278.025				
-181.557	181.557	+	18.156	0.0419408	668.2369772
		+	226.857	0.0189348	668.3339888
-226.847	226.847				
-176.482	176.482	+	176.484	0.0461759	669.1474475
		+	201.115	0.0301403	669.2041791
-201.111	201.111				
-324.526	324.526	+	324.721	0.00278207	669.3547212
		+	229.529	0.018074	669.3653236
-229.518	229.518				
-196.899	196.899	+	196.903	0.0319898	670.3866516
		+	313.727	0.0035831	672.1242623
-313.588	313.588				
-229.179	229.179	+	22.919	0.0182179	674.1376522
		+	303.803	0.00414099	674.1410453
-303.701	303.701				
-180.208	180.208	+	180.211	0.0434139	674.1960862
		+	171.636	0.0499792	674.3071796
-171.634	171.634				
-244.553	244.553	+	24.457	0.0141953	674.3820212
		+	302.263	0.00411948	674.4041802
-302.165	302.165				
-177.389	177.389	+	177.392	0.0452628	675.1230697

		+	298.292	0.00408521	675.1458857
-298.205	298.205				
-187.054	187.054	+	187.058	0.0377167	675.3439579
		+	237.507	0.0160268	676.3031144
-237.493	237.493				
-171.735	171.735	+	171.737	0.0499944	676.3243489
		+	223.133	0.0202695	676.4220500
-223.124	223.124				
-225.185	225.185	+	225.195	0.0197567	677.1416345
		+	231.829	0.0175755	678.2837586
-231.817	231.817				
-355.235	355.235	+	355.757	0.0012696	678.3541181
		+	379.247	0.000825397	678.3661709
-378.095	378.095				
-273.157	273.157	+	273.197	0.00751871	678.3963105
		+	224.981	0.0199252	681.3273545
-224.971	224.971				
-274.526	274.526	+	274.568	0.00751329	681.3943192
		+	209.672	0.0263885	682.3242808
-209.666	209.666				
-183.964	183.964	+	183.966	0.0400882	683.1419595
		+	258.627	0.0107913	683.4062713
-2.586	2.586				
-198.006	198.006	+	19.801	0.0311822	684.3270503
		+	262.465	0.00979323	686.3063358
-262.435	262.435				
-21.863	21.863	+	218.638	0.0221666	687.3647103
		+	181.231	0.0422355	687.3768480
-181.228	181.228				
-277.347	277.347	+	277.393	0.00721511	688.1179644
		+	343.643	0.00190071	688.1209185
-343.291	343.291				
-289.423	289.423	+	28.949	0.00547222	688.3445224
		+	319.192	0.00297771	688.3627944
-319.029	319.029				
-191.694	191.694	+	191.697	0.0351957	689.1521690
-20.224	20.224	+	202.245	0.0294686	689.5628157

		+	214.207	0.0245889	690.1334724
-2.142	2.142				
-234.937	234.937	+	23.495	0.016883	690.3036361
		+	171.911	0.0498205	690.3431613
-171.909	171.909				
-17.536	17.536	+	175.362	0.0467526	691.3521361
		+	264.724	0.00920191	692.1520691
-264.692	264.692				
-224.603	224.603	+	224.613	0.0201277	692.2964809
-251.626	251.626	+	251.648	0.0125556	693.2931602
		+	21.241	0.0252507	693.3046202
-212.403	212.403				
-278.009	278.009	+	278.056	0.00703696	693.3167805
		+	176.085	0.0463997	694.3135925
-176.082	176.082				
-240.306	240.306	+	240.321	0.0151114	694.3472248
		+	210.564	0.0262561	695.1436268
-210.558	210.558				
-189.268	189.268	+	189.272	0.036269	695.4065857
		+	22.224	0.0207745	696.2938459
-222.232	222.232				
-193.855	193.855	+	193.859	0.033569	698.1516955
		+	30.986	0.00373699	698.3824116
-309.737	309.737				
-276.756	276.756	+	276.801	0.00726173	698.4177009
		+	244.712	0.0141325	700.2759178
-244.695	244.695				
-203.546	203.546	+	203.551	0.0288995	703.2525895
		+	196.882	0.0319735	703.3133723
-196.878	196.878				
-189.816	189.816	+	189.819	0.0362448	704.3236112
		+	185.387	0.0385556	704.3338740
-185.384	185.384				
-298.348	298.348	+	298.435	0.00409548	704.3450448
		+	196.722	0.0318921	705.1449679
-196.718	196.718				
-243.658	243.658	+	243.675	0.0141445	705.3045918

		+	223.992	0.0200897	705.3332967
-223.983	223.983				
-293.938	293.938	+	294.014	0.0048097	705.3430507
		+	32.168	0.00303636	705.3536492
-321.504	321.504				
-184.327	184.327	+	18.433	0.0397433	706.3478083
		+	245.828	0.0137207	706.3641289
-24.581	24.581				
-229.607	229.607	+	229.618	0.0179581	707.2964459
		+	189.087	0.0363792	707.3150206
-189.084	189.084				
-216.717	216.717	+	216.725	0.0226256	708.3538821
		+	172.462	0.049242	708.3738688
-17.246	17.246				
-174.835	174.835	+	174.837	0.0471219	709.3145085
		+	202.086	0.0295708	709.3369267
-202.081	202.081				
-368.044	368.044	+	36.885	0.00121263	709.4225778
		+	194.209	0.0334573	711.4378920
-194.205	194.205				
-272.178	272.178	+	272.218	0.007436	712.3967740
		+	244.794	0.0139652	713.1378346
-244.776	244.776				
-187.914	187.914	+	187.917	0.0373984	716.3746731
		+	180.398	0.0433097	716.5162538
-180.395	180.395				
-176.337	176.337	+	176.339	0.0462384	717.1684414
		+	186.593	0.0379392	717.2925742
-18.659	18.659				
-344.354	344.354	+	344.719	0.00191429	717.4938492
		+	230.382	0.0176552	718.1447316
-230.371	230.371				
-215.592	215.592	+	215.599	0.0236208	718.3152600
		+	20.101	0.0300764	719.3334135
-201.005	201.005				
-179.939	179.939	+	179.941	0.0434681	719.3561399
		+	172.979	0.0486977	720.3275602

-172.977	172.977				
-25.636	25.636	+	256.385	0.0115922	720.3433969
		+	194.007	0.0334428	720.3649399
-194.004	194.004				
-269.423	269.423	+	26.946	0.00804318	721.3746430
		+	182.123	0.0413002	722.3173281
-18.212	18.212				
-199.815	199.815	+	19.982	0.0302857	722.3728903
		+	295.194	0.00473929	722.3833697
-295.115	295.115				
-185.343	185.343	+	185.346	0.0385781	722.3928540
		+	179.076	0.0439914	723.3157865
-179.074	179.074				
-219.798	219.798	+	219.807	0.0217005	723.3535565
		+	19.591	0.0321293	723.4027746
-195.906	195.906				
-236.746	236.746	+	236.759	0.0162069	724.3770270
		+	314.246	0.00361364	724.3830579
-314.106	314.106				
-348.054	348.054	+	348.465	0.00175413	725.2846393
		+	188.439	0.0369495	725.4173266
-188.436	188.436				
-192.187	192.187	+	19.219	0.0348741	726.3757596
		+	463.339	0	726.3843296
-4.528	4.528				
-183.891	183.891	+	183.893	0.0401494	727.1350772
		+	395.563	0.000455959	727.3672414
-393.535	393.535				
-182.866	182.866	+	182.868	0.0408153	728.2959266
		+	257.625	0.0110432	728.3555283
-257.599	257.599				
-274.314	274.314	+	274.356	0.00750562	728.3934316
		+	206.198	0.0275756	729.3263973
-206.192	206.192				
-190.816	190.816	+	19.082	0.0357845	730.5420894
		+	196.877	0.0319572	731.3073175
-196.873	196.873				
-191.464	191.464	+	191.467	0.0354053	731.5456022

		+	379.826	0.00083105	732.3535872
-37.865	37.865				
-222.133	222.133	+	222.142	0.0208618	732.4942022
		+	232.344	0.0175629	732.5477644
-232.332	232.332				
-185.701	185.701	+	185.703	0.0383339	733.3243375
		+	209.993	0.026388	733.4366311
-209.987	209.987				
-308.619	308.619	+	308.738	0.00367655	734.3239703
		+	186.766	0.0378304	734.3668065
-186.763	186.763				
-251.187	251.187	+	251.208	0.0125029	735.3261178
		+	288.564	0.005504	735.3632364
-288.499	288.499				
-181.094	181.094	+	181.096	0.0425419	735.4844749
		+	204.701	0.0282697	736.3243477
-204.696	204.696				
-178.674	178.674	+	178.677	0.0443322	736.3846869
		+	274.332	0.00749796	737.3069934
-274.291	274.291				
-175.461	175.461	+	175.463	0.0467666	737.3849323
-21.013	21.013	+	210.136	0.0264338	737.4976846
-20.931	20.931	+	209.316	0.026389	737.5009255
		+	178.213	0.0446661	738.1460083
-17.821	17.821				
-207.333	207.333	+	207.338	0.0270864	738.3144334
		+	175.555	0.0467347	739.3545183
-175.553	175.553				
-242.737	242.737	+	242.753	0.0146842	739.3675526
		+	182.981	0.0407789	739.5556861
-182.978	182.978				
-198.489	198.489	+	198.494	0.0307386	740.3438160
		+	192.484	0.0347366	740.3789567
-192.481	192.481				
-192.498	192.498	+	192.502	0.0347707	740.3812817
		+	194.546	0.0332618	741.1611126

-194.542	194.542				
-271.385	271.385	+	271.423	0.00758607	741.3750957
		+	1.999	0.0303174	742.1439990
-199.896	199.896				
-247.033	247.033	+	247.051	0.0135865	743.3449452
		+	211.364	0.0257531	744.2542625
-211.357	211.357				
-200.214	200.214	+	200.219	0.0303113	744.4136770
		+	176.208	0.0463003	745.3240511
-176.206	176.206				
-192.928	192.928	+	192.932	0.0343984	746.2358757
		+	20.489	0.0283146	747.1571007
-204.885	204.885				
-323.389	323.389	+	323.576	0.00275229	747.3761426
		+	209.674	0.0264037	747.4148017
-209.668	209.668				
-206.162	206.162	+	206.168	0.0275602	747.5685017
		+	208.216	0.0266486	747.5715463
-208.211	208.211				
-245.063	245.063	+	24.508	0.0137781	748.3720922
		+	245.851	0.0137316	748.5724029
-245.833	245.833				
-236.202	236.202	+	236.215	0.0163503	749.3437239
		+	217.988	0.0220834	749.3916612
-21.798	21.798				
-179.215	179.215	+	179.217	0.0439948	749.4310781
		+	218.177	0.0221385	750.3486921
-218.169	218.169				
-251.593	251.593	+	251.614	0.012545	750.3516606
		+	189.429	0.0362093	753.4943844
-189.426	189.426				
-173.125	173.125	+	173.127	0.0484407	755.3544457
		+	190.217	0.0359809	755.3654530
-190.214	190.214				
-183.075	183.075	+	183.078	0.0407126	756.3259477
		+	500.937	0	756.3374274

-492.102	492.102				
-248.297	248.297	+	248.316	0.0133247	756.3422707
		+	380.958	0.000606897	756.4240341
-379.736	379.736				
-177.631	177.631	+	177.633	0.0450784	757.3455922
		+	326.309	0.00282575	757.3739258
-326.105	326.105				
-192.412	192.412	+	192.416	0.0348135	757.3846510
		+	226.774	0.0190061	758.3554737
-226.763	226.763				
-231.617	231.617	+	231.629	0.017633	758.5733422
		+	288.067	0.00558817	759.1717120
-288.004	288.004				
-301.637	301.637	+	301.733	0.00408237	759.3381405
		+	326.436	0.00283912	759.3431508
-326.231	326.231				
-338.024	338.024	+	338.322	0.00182313	760.3726280
		+	289.237	0.005542	761.2323453
-289.171	289.171				
-18.175	18.175	+	181.752	0.0417047	761.4157882
		+	177.007	0.0459182	761.4725649
-177.005	177.005				
-199.893	199.893	+	199.897	0.0303016	761.5843851
		+	233.793	0.0170628	762.3259156
-233.781	233.781				
-323.204	323.204	+	323.391	0.0027439	762.5430084
		+	221.533	0.0210006	763.4291944
-221.525	221.525				
-196.084	196.084	+	196.088	0.0320466	763.4319444
		+	194.892	0.0331156	764.3761267
-194.888	194.888				
-196.964	196.964	+	196.969	0.0320225	764.4371645
		+	181.341	0.0421709	764.4424044
-181.338	181.338				
-204.247	204.247	+	204.252	0.0283893	764.5471111
		+	218.019	0.0221109	765.3368662
-218.011	218.011				
-34.925	34.925	+	349.677	0.0017671	766.2361318

		+	208.559	0.0265645	766.3831717
-208.553	208.553				
-22.338	22.338	+	223.389	0.020228	767.3165278
		+	252.741	0.0121541	769.3947211
-252.719	252.719				
-200.603	200.603	+	200.608	0.0304368	770.3346533
		+	207.679	0.0268954	770.4990393
-207.674	207.674				
-2.093	2.093	+	209.306	0.0263739	770.5011388
		+	205.456	0.0279734	772.3355865
-205.451	205.451				
-308.045	308.045	+	308.162	0.00366174	773.2558447
		+	221.875	0.0208745	773.3260923
-221.867	221.867				
-248.437	248.437	+	248.457	0.013216	773.3417849
		+	173.929	0.0478693	774.1814638
-173.927	173.927				
-271.301	271.301	+	27.134	0.00768588	774.3483830
		+	292.959	0.00495558	774.3514260
-292.885	292.885				
-175.062	175.062	+	175.064	0.0469678	774.3628932
		+	364.256	0.00118275	774.3868088
-363.565	363.565				
-175.468	175.468	+	17.547	0.0468053	775.2439062
		+	180.521	0.0431014	775.2523578
-180.519	180.519				
-209.607	209.607	+	209.613	0.0263582	775.3349247
		+	196.039	0.0321356	775.3454548
-196.035	196.035				
-286.904	286.904	+	286.965	0.00589899	775.3550171
		+	261.998	0.00994757	776.1629277
-261.969	261.969				
-224.689	224.689	+	224.698	0.0200905	776.4047014
		+	177.354	0.0453903	777.2342957
-177.352	177.352				
-204.464	204.464	+	204.469	0.0282388	777.3870808
		+	209.449	0.0264495	778.3555893

-209.443	209.443				
-190.955	190.955	+	190.959	0.0356665	778.4186976
		+	197.688	0.0314136	778.4217154
-197.684	197.684				
-214.844	214.844	+	214.851	0.0239879	778.4427869
		+	221.145	0.0211689	779.1952899
-221.137	221.137				
-260.295	260.295	+	260.322	0.0104504	779.2550429
		+	261.299	0.0101453	779.4262985
-261.271	261.271				
-219.797	219.797	+	219.805	0.0216867	779.4375642
		+	212.832	0.0251256	779.4645508
-212.825	212.825				
-28.609	28.609	+	286.149	0.00586607	780.3970555
		+	197.114	0.0318896	780.4370805
-19.711	19.711				
-244.724	244.724	+	244.741	0.0141436	781.4424260
		+	17.693	0.0459621	782.2452579
-176.927	176.927				
-34.383	34.383	+	344.188	0.00190747	783.3849888
		+	232.858	0.0173649	784.3729768
-232.846	232.846				
-211.774	211.774	+	21.178	0.0256399	785.1615304
		+	193.868	0.0335281	785.3659066
-193.864	193.864				
-248.279	248.279	+	248.298	0.0132922	786.3864810
		+	204.051	0.0284665	786.4947312
-204.046	204.046				
-235.254	235.254	+	235.267	0.016694	787.3729905
		+	234.338	0.0170589	787.3832349
-234.326	234.326				
-455.851	455.851	+	466.681	0	787.4434947
		+	222.177	0.0208753	788.1619575
-222.168	222.168				
-243.518	243.518	+	243.535	0.0141226	788.3347495
		+	275.509	0.00748454	789.4234611
-275.466	275.466				
-214.499	214.499	+	214.505	0.0241954	790.4069234

		+	178.808	0.0442726	791.4158178
-178.805	178.805				
-281.911	281.911	+	281.963	0.0064173	791.4382851
		+	219.097	0.0219698	791.4640155
-219.089	219.089				
-174.825	174.825	+	174.827	0.0471025	792.3983807
		+	208.222	0.0266637	792.4026163
-208.216	208.216				
-232.319	232.319	+	232.331	0.0175503	792.4235241
		+	237.558	0.0160387	794.3538652
-237.544	237.544				
-173.499	173.499	+	173.501	0.0479479	794.4152141
		+	194.296	0.0334134	795.3839388
-194.292	194.292				
-341.999	341.999	+	342.337	0.00189065	795.3972892
		+	172.299	0.049231	795.4475386
-172.297	172.297				
-210.335	210.335	+	210.341	0.0262016	796.2454191
		+	195.096	0.0330796	796.3725386
-195.092	195.092				
-202.943	202.943	+	202.948	0.0292807	796.4324574
		+	195.933	0.0321031	797.3766331
-195.929	195.929				
-30.945	30.945	+	309.572	0.00371662	798.3367799
		+	213.459	0.0248701	798.3860663
-213.452	213.452				
-184.557	184.557	+	18.456	0.0396053	799.3452113
		+	186.454	0.038012	799.5367912
-186.451	186.451				
-312.661	312.661	+	312.795	0.00367598	800.5119444
		+	195.928	0.0320869	801.4235617
-195.924	195.924				
-252.938	252.938	+	25.296	0.0121854	802.3563561
		+	230.022	0.0177127	802.3642176
-230.011	230.011				
-208.388	208.388	+	208.394	0.0266546	802.5637336
		+	203.707	0.0287722	803.1517602
-203.702	203.702				
-201.091	201.091	+	201.095	0.0301243	803.1721577

		+	217.719	0.0220149	803.4033497
-217.711	217.711				
-19.409	19.409	+	194.094	0.0335262	803.4146667
		+	297.017	0.00417305	803.4383586
-296.934	296.934				
-230.841	230.841	+	230.852	0.0177175	803.5673376
		+	20.727	0.0270561	804.2544941
-207.264	207.264				
-199.096	199.096	+	199.101	0.0306423	805.3561583
		+	287.731	0.00556285	805.4552658
-287.669	287.669				
-181.291	181.291	+	181.294	0.0422113	806.3662657
		+	17.428	0.0476604	806.4146515
-174.278	174.278				
-324.355	324.355	+	324.548	0.0027735	807.3629555
		+	203.506	0.0289159	807.3744945
-203.501	203.501				
-221.913	221.913	+	221.922	0.0208212	807.3844372
		+	247.563	0.0135367	808.2732999
-247.544	247.544				
-208.665	208.665	+	208.671	0.0264946	809.3430725
		+	176.953	0.0459832	809.3551129
-176.951	176.951				
-194.284	194.284	+	194.288	0.0333967	809.3959980
		+	188.486	0.0369059	809.4143009
-188.483	188.483				
-175.228	175.228	+	17.523	0.0470058	809.5221022
		+	178.718	0.0443512	809.5478884
-178.716	178.716				
-306.164	306.164	+	306.274	0.00375033	810.3643398
		+	224.484	0.0201009	810.4072220
-224.474	224.474				
-293.065	293.065	+	293.139	0.00496751	810.4120747
		+	259.786	0.0106605	810.6864131
-259.759	259.759				
-199.762	199.762	+	199.767	0.0303001	811.3833096
		+	18.587	0.0382881	811.3951452

-185.867	185.867				
-18.171	18.171	+	181.712	0.0417676	811.4435786
		+	18.962	0.0362252	812.4470882
-189.617	189.617				
-251.147	251.147	+	251.167	0.0125662	813.3967935
		+	185.204	0.0387982	813.5069301
-185.202	185.202				
-315.636	315.636	+	315.784	0.00335244	814.1743119
		+	180.546	0.0430818	814.2532422
-180.543	180.543				
-200.285	200.285	+	20.029	0.0303432	814.4052794
		+	341.425	0.0018676	815.3358089
-341.097	341.097				
-21.382	21.382	+	213.827	0.0247358	815.3443376
		+	215.466	0.0236899	815.3936127
-215.459	215.459				
-191.653	191.653	+	191.657	0.0352043	815.4138884
		+	188.251	0.0371063	815.4374342
-188.248	188.248				
-195.297	195.297	+	195.301	0.0327944	816.3966648
		+	226.588	0.0191189	816.4235432
-226.578	226.578				
-273.546	273.546	+	273.587	0.00747508	816.5429501
		+	233.647	0.0172055	817.3832317
-233.635	233.635				
-206.705	206.705	+	206.711	0.0272638	817.4175750
		+	216.969	0.0226099	819.3250229
-216.962	216.962				
-258.629	258.629	+	258.655	0.0108011	819.4053696
		+	196.546	0.0319329	819.4348850
-196.542	196.542				
-274.941	274.941	+	274.983	0.00743852	820.3943614
		+	213.889	0.0246978	821.3770922
-213.883	213.883				
-1.771	1.771	+	177.102	0.0457755	821.4522179
		+	174.375	0.047555	822.3364591
-174.372	174.372				
-180.083	180.083	+	180.085	0.0435054	822.5264357

		+	176.947	0.0459638	823.3755567
-176.945	176.945				
-174.417	174.417	+	174.419	0.0475745	823.3940321
		+	208.375	0.0266395	823.5368599
-208.369	208.369				
-223.588	223.588	+	223.598	0.0201207	825.3556556
		+	179.646	0.0436223	825.3748455
-179.644	179.644				
-214.517	214.517	+	214.524	0.02421	825.3862386
		+	250.539	0.0126717	826.2839849
-250.518	250.518				
-188.474	188.474	+	188.477	0.0368887	826.3744319
		+	210.485	0.0261951	828.3553803
-210.479	210.479				
-236.518	236.518	+	236.532	0.016236	828.3848589
		+	428.892	0	828.4343793
-42.319	42.319				
-307.091	307.091	+	307.204	0.0037754	829.3833525
		+	402.321	0.00024581	829.4166639
-399.767	399.767				
-227.038	227.038	+	227.048	0.0189477	829.4268824
		+	237.706	0.0160507	829.4323554
-237.692	237.692				
-244.541	244.541	+	244.558	0.0141841	831.3465936
		+	200.706	0.0303683	831.3637494
-200.701	200.701				
-291.648	291.648	+	291.718	0.00512381	831.3966458
		+	173.923	0.0478497	832.3561713
-173.921	173.921				
-216.559	216.559	+	216.566	0.0229035	832.3939525
		+	192.909	0.0343815	832.4071766
-192.905	192.905				
-225.232	225.232	+	225.242	0.019711	832.4276874
		+	265.351	0.00904415	832.4327623
-265.319	265.319				
-230.277	230.277	+	230.289	0.0176428	833.3766270
		+	196.122	0.0320182	834.3741201
-196.118	196.118				
-186.993	186.993	+	186.997	0.0376993	834.4136677

		+	185.503	0.0384492	834.4542899
-185.501	185.501				
-197.133	197.133	+	197.138	0.031824	835.1749054
		+	339.735	0.00184192	836.3862175
-339.424	339.424				
-175.964	175.964	+	175.967	0.0464695	836.4637198
		+	183.218	0.0406649	837.3733669
-183.215	183.215				
-189.346	189.346	+	189.349	0.0363032	837.4263972
		+	209.782	0.0264092	837.5336967
-209.776	209.776				
-212.006	212.006	+	212.013	0.0255553	839.2762274
		+	254.231	0.0117639	839.3658575
-254.208	254.208				
-180.306	180.306	+	180.308	0.0433138	839.3869479
		+	256.145	0.0116537	839.3938483
-256.121	256.121				
-185.685	185.685	+	185.688	0.0383165	839.6064607
		+	289.433	0.0055612	840.3469210
-289.367	289.367				
-19.078	19.078	+	190.784	0.0357924	840.3846032
		+	357.322	0.00128433	840.4161907
-356.773	356.773				
-310.207	310.207	+	310.332	0.00375241	840.4225731
		+	287.306	0.00565237	840.5429509
-287.244	287.244				
-222.312	222.312	+	222.321	0.0207126	841.2552816
		+	255.376	0.0118105	841.3333942
-255.352	255.352				
-258.651	258.651	+	258.677	0.0108109	841.3835585
		+	204.807	0.0282835	841.4052988
-204.801	204.801				
-204.122	204.122	+	204.127	0.0284386	841.5380910
		+	211.872	0.0256287	842.3859483
-211.866	211.866				
-197.475	197.475	+	19.748	0.0316259	842.4024528
		+	279.934	0.006873	843.3645366

-279.885	279.885				
-254.512	254.512	+	254.535	0.0118049	843.3859519
		+	190.669	0.0358311	843.4334629
-190.666	190.666				
-173.548	173.548	+	17.355	0.0479788	844.3841878
		+	416.747	0.000275	844.4144196
-412.692	412.692				
-265.289	265.289	+	265.321	0.00903547	844.4276377
		+	180.131	0.0434368	845.3445642
-180.129	180.129				
-256.359	256.359	+	256.383	0.0115819	845.4029485
		+	237.152	0.0161277	845.4524951
-237.138	237.138				
-207.926	207.926	+	207.932	0.0265885	846.3963184
		+	172.149	0.0494446	846.4455313
-172.147	172.147				
-215.111	215.111	+	215.118	0.0240511	847.4662774
		+	188.544	0.0369405	847.5039385
-188.541	188.541				
-179.784	179.784	+	179.787	0.0434536	848.3865202
		+	221.028	0.0212033	848.4245759
-221.019	221.019				
-234.479	234.479	+	234.491	0.0169432	849.3850679
		+	176.793	0.0460303	849.4058060
-176.791	176.791				
-39.349	39.349	+	395.516	0.00045478	850.4044763
		+	174.039	0.0477806	850.5222486
-174.037	174.037				
-195.025	195.025	+	195.029	0.0331112	851.2064188
		+	177.643	0.0450602	852.2660198
-17.764	17.764				
-217.846	217.846	+	217.854	0.0220559	852.4565577
		+	184.933	0.0393503	853.1967094
-18.493	18.493				
-286.184	286.184	+	286.244	0.00587263	853.4046619
		+	189.214	0.0363968	854.3959717
-18.921	18.921				
-334.477	334.477	+	334.742	0.00191736	854.4738029

		+	220.898	0.0212641	854.5349484
-22.089	22.089				
-234.896	234.896	+	234.909	0.0168707	855.6015135
-18.912	18.912	+	189.123	0.0363962	856.3567047
-2.012	2.012	+	201.205	0.0301416	856.4663403
		+	179.434	0.0437512	856.6050921
-179.431	179.431				
-189.502	189.502	+	189.505	0.036185	857.4246085
		+	197.381	0.0317188	857.5333813
-197.377	197.377				
-172.307	172.307	+	172.309	0.0492706	857.5540790
		+	227.053	0.0189605	857.6169101
-227.043	227.043				
-254.115	254.115	+	254.137	0.0117435	858.4448416
		+	270.443	0.00801186	859.4270188
-270.406	270.406				
-28.018	28.018	+	28.023	0.00688034	860.4247406
		+	244.076	0.014233	861.4067933
-244.059	244.059				
-252.274	252.274	+	252.295	0.0121426	862.4049304
		+	251.611	0.0125345	862.4774133
-25.159	25.159				
-204.143	204.143	+	204.148	0.0284216	862.5379869
		+	336.101	0.00193012	863.4133977
-335.824	335.824				
-252.709	252.709	+	252.731	0.0121437	863.4450181
		+	180.851	0.0429416	863.5577271
-180.848	180.848				
-276.132	276.132	+	276.176	0.00743212	864.3840017
		+	255.074	0.0117487	864.4173736
-25.505	25.505				
-253.035	253.035	+	253.057	0.0121959	866.2756719
		+	402.055	0.000244444	866.4359877
-399.524	399.524				
-24.547	24.547	+	245.488	0.0138329	868.3756005
		+	219.731	0.0216319	868.4152888

-219.723	219.723				
-28.153	28.153	+	281.582	0.006527	868.4531410
		+	228.655	0.0184171	869.2671932
-228.645	228.645				
-191.437	191.437	+	191.441	0.035354	869.3634794
		+	234.141	0.0170094	869.5333274
-234.128	234.128				
-204.405	204.405	+	20.441	0.0283722	869.5448070
		+	200.493	0.0303886	869.5989445
-200.488	200.488				
-198.897	198.897	+	198.901	0.030743	869.6010844
		+	202.485	0.0294496	870.2432727
-202.481	202.481				
-209.701	209.701	+	209.707	0.0264341	870.4676556
		+	176.632	0.0461435	870.5467501
-17.663	17.663				
-237.236	237.236	+	237.249	0.0161397	872.2767813
		+	197.842	0.03131	873.4455243
-197.838	197.838				
-226.217	226.217	+	226.227	0.0194143	874.3942305
		+	243.646	0.0141335	875.3766422
-24.363	24.363				
-303.861	303.861	+	303.964	0.00415183	875.4970889
		+	224.087	0.0201162	876.3845326
-224.078	224.078				
-212.836	212.836	+	212.842	0.0251405	877.3929734
		+	219.375	0.0217758	877.4044812
-219.367	219.367				
-171.886	171.886	+	171.888	0.0498006	877.4360649
		+	328.463	0.0025728	877.4770495
-328.244	328.244				
-20.034	20.034	+	200.344	0.0303591	878.3859500
		+	21.534	0.0239294	878.4242280
-215.333	215.333				
-279.147	279.147	+	279.195	0.00697447	879.4161253
		+	22.399	0.0200764	880.4159773
-22.398	22.398				
-191.691	191.691	+	191.695	0.0351786	881.2213245

		+	376.234	0.001	881.3875638
-375.196	375.196				
-236.438	236.438	+	236.452	0.016224	881.4247326
		+	220.748	0.021266	881.4534516
-22.074	22.074				
-276.988	276.988	+	277.034	0.00719247	882.3946912
		+	20.233	0.0295161	884.4467124
-202.325	202.325				
-221.554	221.554	+	221.562	0.0209394	885.4572223
		+	252.124	0.0122203	886.4155517
-252.102	252.102				
-233.048	233.048	+	23.306	0.0173333	887.4349882
		+	281.044	0.00649194	887.4737286
-280.993	280.993				
-178.888	178.888	+	17.889	0.0440979	887.5446102
		+	315.836	0.00335725	888.4563553
-315.688	315.688				
-173.337	173.337	+	173.339	0.0480503	888.5284343
		+	215.357	0.0239439	889.3839817
-21.535	21.535				
-227.193	227.193	+	227.203	0.0189393	889.4771893
		+	291.083	0.00508146	889.5137185
-291.013	291.013				
-245.895	245.895	+	245.913	0.0137536	890.4350651
		+	194.194	0.0334239	890.5455433
-19.419	19.419				
-225.843	225.843	+	225.853	0.0196364	891.4050502
		+	177.053	0.045957	891.5276974
-177.051	177.051				
-173.284	173.284	+	173.286	0.0482124	892.3562530
		+	192.868	0.0345044	892.4155203
-192.865	192.865				
-189.507	189.507	+	189.511	0.0362021	893.3633321
		+	233.443	0.0172565	893.4060751
-233.431	233.431				
-171.703	171.703	+	171.705	0.0499345	894.3948100
		+	229.626	0.0179706	895.3754543

-229.615	229.615				
-209.179	209.179	+	209.185	0.026379	896.3877686
		+	224.763	0.0200266	896.4248395
-224.753	224.753				
-36.347	36.347	+	364.159	0.00118033	896.4446398
		+	246.484	0.0135756	897.4233545
-246.466	246.466				
-275.029	275.029	+	275.072	0.00744615	897.4446129
		+	251.956	0.0123449	899.4363960
-251.935	251.935				
-189.572	189.572	+	189.575	0.036208	900.5365573
		+	193.355	0.0339634	901.4536910
-193.352	193.352				
-1.807	1.807	+	180.702	0.0429435	901.4755188
		+	239.531	0.0152079	901.5485198
-239.516	239.516				
-173.328	173.328	+	17.333	0.0480308	903.4926880
		+	24.409	0.0142553	904.4530736
-244.073	244.073				
-301.659	301.659	+	301.755	0.00408763	905.3956232
		+	277.678	0.00712329	905.4240072
-277.632	277.632				
-210.108	210.108	+	210.114	0.0264185	905.5075596
		+	209.149	0.0263489	906.3841748
-209.143	209.143				
-203.811	203.811	+	203.816	0.0287878	907.4537904
		+	297.148	0.00404467	909.4551804
-297.065	297.065				
-209.249	209.249	+	209.255	0.0264091	910.3777484
		+	249.597	0.0129449	911.4735802
-249.577	249.577				
-37.773	37.773	+	378.867	0.00082167	912.3949106
		+	254.444	0.0117946	912.4339515
-254.421	254.421				
-254.171	254.171	+	254.194	0.0117537	913.4859025
		+	259.978	0.0106704	914.4476867
-259.951	259.951				
-247.171	247.171	+	24.719	0.0135149	914.4523890

		+	178.335	0.0444983	915.2743770
-178.333	178.333				
-251.907	251.907	+	251.928	0.0123241	915.4328864
		+	196.677	0.0319207	915.4932517
-196.673	196.673				
-215.369	215.369	+	215.376	0.0239049	915.5036078
		+	18.802	0.0373451	915.5278142
-188.017	188.017				
-215.804	215.804	+	215.811	0.0234338	916.4649427
		+	200.471	0.0303726	916.4865935
-200.466	200.466				
-371.651	371.651	+	372.567	0.00124406	916.5569399
		+	202.919	0.0292649	916.5615301
-202.914	202.914				
-317.711	317.711	+	317.868	0.00318704	917.4721504
		+	176.558	0.0461728	917.5439600
-176.556	176.556				
-248.812	248.812	+	248.831	0.0131765	918.4181375
		+	268.902	0.00802742	918.4225674
-268.866	268.866				
-178.362	178.362	+	178.364	0.0445173	918.4823833
		+	258.527	0.0108867	918.5038641
-258.501	258.501				
-306.144	306.144	+	306.254	0.00374536	919.4861332
		+	254.287	0.0117741	920.4475775
-254.264	254.264				
-179.947	179.947	+	17.995	0.043487	921.2451450
		+	196.847	0.0319409	921.3844500
-196.843	196.843				
-213.542	213.542	+	213.549	0.0248998	922.5434806
		+	2.1	0.0264032	923.4454810
-209.994	209.994				
-228.758	228.758	+	228.768	0.0184426	924.3950296
		+	228.607	0.0184651	925.4864566
-228.597	228.597				
-204.945	204.945	+	204.951	0.0283302	926.4353279
		+	209.486	0.0264647	927.4053931

-20.948	20.948				
-273.498	273.498	+	273.539	0.0074599	927.4332337
		+	287.401	0.00565876	927.4669767
-287.339	287.339				
-268.138	268.138	+	268.173	0.00823047	928.2216349
		+	342.497	0.00189399	929.4129766
-342.157	342.157				
-190.285	190.285	+	190.289	0.0359656	929.4463983
		+	252.023	0.0121997	929.5069870
-252.002	252.002				
-202.589	202.589	+	202.594	0.0293031	930.4442607
		+	187.286	0.0375342	930.4658373
-187.283	187.283				
-172.224	172.224	+	172.226	0.0492734	931.2633615
		+	22.664	0.0191448	932.4828286
-22.663	22.663				
-275.707	275.707	+	275.751	0.00740909	932.5257074
		+	177.794	0.044983	933.5037987
-177.792	177.792				
-310.513	310.513	+	310.639	0.00376276	934.4150412
		+	212.358	0.025221	935.4235535
-212.352	212.352				
-206.922	206.922	+	206.928	0.0272752	935.5440094
		+	233.903	0.0170751	936.4263521
-23.389	23.389				
-222.057	222.057	+	222.066	0.0208347	936.4322637
		+	259.598	0.0106311	937.4265334
-259.571	259.571				
-189.919	189.919	+	189.922	0.0362205	937.4346541
		+	24.831	0.0133138	938.4455883
-24.829	24.829				
-301.205	301.205	+	3.013	0.00406146	938.4567103
		+	312.651	0.00365556	939.4340329
-312.517	312.517				
-63.644	63.644	+	577.986	0	939.4665885
		+	190.614	0.0357967	939.5032099
-19.061	19.061				
-178.208	178.208	+	17.821	0.0446471	940.4532570

		+	361.045	0.00115431	940.4640392
-360.425	360.425				
-278.832	278.832	+	27.888	0.00695966	941.3745312
		+	206.784	0.0273095	941.3947129
-206.779	206.779				
-224.234	224.234	+	224.243	0.0200927	942.5035409
		+	185.898	0.0382828	943.4259401
-185.895	185.895				
-253.863	253.863	+	253.885	0.011903	943.4633569
		+	178.946	0.0441169	944.6282124
-178.943	178.943				
-18.083	18.083	+	180.832	0.0429228	944.6305962
		+	388.177	0.000642336	945.4050644
-386.606	386.606				
-186.138	186.138	+	186.141	0.0381815	946.4367874
		+	292.942	0.00494964	946.4632029
-292.868	292.868				
-200.595	200.595	+	2.006	0.0304207	947.3853326
		+	298.043	0.0040801	948.4328276
-297.958	297.958				
-200.193	200.193	+	200.198	0.0302953	948.4548327
		+	228.457	0.0184524	948.4762999
-228.446	228.446				
-217.292	217.292	+	2.173	0.0224143	949.4257887
		+	199.531	0.0304092	950.2753058
-199.527	199.527				
-232.506	232.506	+	232.518	0.017441	950.3742043
		+	187.627	0.0375243	950.4244457
-187.624	187.624				
-209.386	209.386	+	209.392	0.0264192	950.5350009
		+	274.246	0.00749032	951.5482083
-274.204	274.204				
-249.192	249.192	+	249.212	0.0131204	951.5520890
		+	218.765	0.0219561	952.4624048
-218.757	218.757				
-200.356	200.356	+	200.361	0.0303751	953.2044895
		+	191.437	0.0353369	953.4156886

-191.434	191.434				
-347.767	347.767	+	348.174	0.00174771	953.4552397
		+	212.678	0.0251034	954.4858571
-212.671	212.671				
-217.746	217.746	+	217.753	0.0220286	955.4258405
		+	179.126	0.0439879	957.4771164
-179.123	179.123				
-213.665	213.665	+	213.672	0.0248493	957.5376383
		+	25.296	0.012175	958.3061049
-252.938	252.938				
-25.542	25.542	+	255.444	0.0117366	958.4746541
		+	183.456	0.0404776	959.6433015
-183.453	183.453				
-17.353	17.353	+	173.532	0.047987	962.4133293
		+	193.028	0.0343277	962.6420912
-193.025	193.025				
-205.322	205.322	+	205.327	0.0279602	963.5865950
		+	271.534	0.0075015	964.4349656
-271.496	271.496				
-324.421	324.421	+	324.615	0.00277778	964.5074727
		+	175.373	0.0467912	965.3847055
-175.371	175.371				
-358.719	358.719	+	359.305	0.00113386	965.4453494
		+	263.045	0.00965595	966.2140820
-263.015	263.015				
-25.913	25.913	+	259.157	0.0107996	966.4237273
		+	220.895	0.0212505	966.5243311
-220.887	220.887				
-184.673	184.673	+	184.676	0.0395487	968.4351722
		+	190.218	0.0359981	968.5463002
-190.215	190.215				
-172.639	172.639	+	172.641	0.0489972	969.2142525
		+	178.261	0.0445848	969.4156771
-178.259	178.259				
-373.658	373.658	+	374.641	0.0012549	969.5144685
		+	214.769	0.0240266	970.4540039
-214.762	214.762				
-284.612	284.612	+	28.467	0.00614602	971.4339783
		+	23.838	0.0158711	971.4943869

-238.366	238.366				
-228.125	228.125	+	228.135	0.0184787	972.2230781
		+	206.627	0.0272825	973.6174736
-206.622	206.622				
-197.972	197.972	+	197.976	0.0312157	974.2151763
		+	200.187	0.0302794	974.5059281
-200.182	200.182				
-288.952	288.952	+	289.017	0.00552294	975.5533715
		+	181.645	0.041788	975.6337046
-181.643	181.643				
-176.088	176.088	+	17.609	0.046419	977.4064982
		+	242.104	0.0146279	977.4333287
-242.088	242.088				
-178.248	178.248	+	178.251	0.0446476	978.4057352
		+	185.894	0.0382653	979.4254604
-185.891	185.891				
-183.464	183.464	+	183.466	0.0404957	979.5945547
		+	221.716	0.020861	982.4361373
-221.707	221.707				
-208.424	208.424	+	20.843	0.0266697	983.4453424
		+	324.754	0.00278638	991.5662198
-32.456	32.456				
-189.993	189.993	+	189.996	0.0360704	995.4333686
-172.231	172.231	+	172.233	0.0492932	995.5763207

Supplementary Table S3: Posthoc test HCT-8 for control cells in positive-ion mode

Control	Infected	C: ANOVA Significant	N: -Log ANOVA p value	N: ANOVA q-value	MALDI m/z
		+	330.681	0.01	303.0371324
330.447	-330.447				
196.366	-196.366	+	19.637	0.0404691	306.0807259
		+	188.792	0.0459156	307.0438995
188.789	-188.789				
198.801	-198.801	+	198.806	0.0394055	309.1442331
273.885	-273.885	+	273.926	0.0164621	309.2258596

		+	273.111	0.0165263	311.1345616
27.307	-27.307				
191.545	-191.545	+	191.548	0.0438229	311.3138060
		+	241.172	0.0215603	312.1177177
241.157	-241.157				
192.551	-192.551	+	192.554	0.0429462	312.2030639
		+	206.009	0.0350413	312.3258246
206.003	-206.003				
299.029	-299.029	+	299.117	0.0143742	313.0516908
		+	273.054	0.0164953	313.2232912
273.014	-273.014				
200.808	-200.808	+	200.812	0.0383747	313.2344929
		+	221.933	0.0268492	320.3310806
221.925	-221.925				
276.954	-276.954	+	2.77	0.016214	322.1871007
		+	276.349	0.0158871	324.1919527
276.305	-276.305				
191.931	-191.931	+	191.934	0.0438125	325.1979807
		+	2.756	0.0157198	326.0013399
275.557	-275.557				
236.594	-236.594	+	236.607	0.0234104	327.2137600
		+	21.781	0.0290674	329.0145302
217.802	-217.802				
189.863	-189.863	+	189.866	0.0450106	330.0728286
		+	188.265	0.0463955	330.9955654
188.262	-188.262				
211.901	-211.901	+	211.907	0.0316066	332.0757174
		+	242.518	0.022024	335.0934992
242.502	-242.502				
18.481	-18.481	+	184.813	0.0498465	337.1250760
		+	203.227	0.0366895	337.2215775
203.222	-203.222				
21.613	-21.613	+	216.138	0.0300383	338.1832886
		+	215.267	0.0305887	339.0684122
21.526	-21.526				
197.049	-197.049	+	197.053	0.0402514	339.1430875
		+	234.938	0.0235349	339.2389238
234.925	-234.925				
229.484	-229.484	+	229.495	0.0245342	339.2528015

		+	233.143	0.0236155	340.2422868
233.131	-233.131				
213.624	-213.624	+	213.631	0.0309755	341.2546005
		+	20.445	0.0362926	342.1881899
204.445	-204.445				
198.217	-198.217	+	198.222	0.0397289	342.2135459
		+	187.239	0.0477888	342.2991495
187.236	-187.236				
206.637	-206.637	+	206.642	0.0347788	344.1464660
		+	319.274	0.0148663	344.2040063
319.109	-319.109				
2.02	-2.02	+	202.005	0.0375389	344.2289157
		+	261.094	0.019025	344.2421278
261.066	-261.066				
198.956	-198.956	+	198.961	0.0392267	346.0411041
		+	193.846	0.0418657	346.0929795
193.842	-193.842				
223.979	-223.979	+	223.989	0.0264409	346.1737427
		+	25.382	0.0186489	347.0249245
253.797	-253.797				
18.812	-18.812	+	188.123	0.0466374	347.0448930
		+	230.495	0.0241401	348.0280860
230.484	-230.484				
222.919	-222.919	+	222.928	0.0263791	348.0702381
		+	200.743	0.0383549	349.0735007
200.739	-200.739				
213.779	-213.779	+	213.786	0.0310926	350.0858398
		+	229.649	0.0246992	351.2494329
229.638	-229.638				
22.953	-22.953	+	229.541	0.0245547	351.2504608
		+	190.733	0.0444114	352.2542349
190.729	-190.729				
221.809	-221.809	+	221.817	0.0269423	353.2166191
		+	274.209	0.0165247	353.2545715
274.167	-274.167				
246.065	-246.065	+	246.083	0.0203977	353.2660582
		+	218.754	0.0282689	354.2136281

218.746	-218.746				
189.044	-189.044	+	189.047	0.0456611	355.2084988
		+	218.283	0.0284695	355.2338309
218.275	-218.275				
261.468	-261.468	+	261.496	0.0191748	356.2039426
		+	251.214	0.0187975	356.2290028
251.193	-251.193				
214.666	-214.666	+	214.673	0.0310245	356.2387988
		+	250.744	0.0189572	356.2411170
250.724	-250.724				
20.639	-20.639	+	206.395	0.0349181	356.2653992
		+	239.494	0.0221561	357.2243288
239.479	-239.479				
18.938	-18.938	+	189.383	0.0455323	357.2479844
		+	212.834	0.0310693	358.2192995
212.828	-212.828				
186.791	-186.791	+	186.794	0.0480169	358.9276468
		+	264.769	0.0188344	361.0524166
264.737	-264.737				
30.959	-30.959	+	309.712	0.0135565	362.0846581
		+	204.655	0.0359628	364.0652056
20.465	-20.465				
212.945	-212.945	+	212.952	0.0310248	364.1031108
		+	204.089	0.0364193	366.2493171
204.084	-204.084				
207.728	-207.728	+	207.733	0.0345118	366.2510541
		+	245.706	0.0208177	367.2081596
245.688	-245.688				
366.399	-366.399	+	367.161	0.00935385	367.2841813
		+	201.701	0.0377857	368.0235828
201.696	-201.696				
185.588	-185.588	+	185.591	0.0493306	368.2288058
		+	222.496	0.0264006	368.2874719
222.487	-222.487				
283.993	-283.993	+	284.049	0.0148	369.0068955
		+	242.399	0.0219761	369.0256963
242.383	-242.383				
192.979	-192.979	+	192.983	0.0425839	369.2244099

		+	196.948	0.0402902	369.2487317
196.944	-196.944				
191.744	-191.744	+	191.748	0.0437442	369.2608285
		+	22.932	0.0246185	370.0084609
229.309	-229.309				
230.071	-230.071	+	230.082	0.0244024	370.0105297
		+	259.558	0.018488	370.0521735
259.532	-259.532				
266.308	-266.308	+	266.341	0.0181874	370.2194340
		+	26.726	0.0179206	370.2205635
267.226	-267.226				
231.592	-231.592	+	231.603	0.023592	370.3316916
		+	213.317	0.0310936	371.0542189
21.331	-21.331				
257.597	-257.597	+	257.622	0.0185991	371.2395957
		+	259.296	0.0183498	371.2405419
259.269	-259.269				
193.922	-193.922	+	193.926	0.0418012	372.0561399
		+	229.567	0.0245958	372.0676526
229.556	-229.556				
288.577	-288.577	+	288.642	0.014615	372.2353043
		+	25.058	0.0188622	372.2589020
25.056	-25.056				
224.545	-224.545	+	224.554	0.0264463	372.2606534
		+	25.216	0.0188229	373.2557008
252.138	-252.138				
184.885	-184.885	+	184.887	0.0497951	374.2389533
		+	187.152	0.0477704	375.2134333
187.149	-187.149				
271.063	-271.063	+	271.101	0.0166204	375.2237899
		+	254.495	0.0186594	376.1248762
254.472	-254.472				
365.449	-365.449	+	366.186	0.00907463	380.0951243
		+	219.993	0.0276967	380.2851022
219.985	-219.985				
19.191	-19.191	+	191.914	0.0438181	381.2489824
		+	196.246	0.0405165	382.2447756
196.242	-196.242				
248.649	-248.649	+	248.668	0.0191408	382.2559581

		+	203.113	0.0367692	383.2394126
203.108	-203.108				
188.932	-188.932	+	188.935	0.0456944	383.2409789
		+	194.317	0.0417289	383.9967746
194.313	-194.313				
220.192	-220.192	+	2.202	0.0276923	384.2352420
		+	254.713	0.0187357	384.2593818
25.469	-25.469				
253.073	-253.073	+	253.095	0.0185926	384.2605378
		+	219.804	0.0279011	384.2718066
219.796	-219.796				
284.908	-284.908	+	284.966	0.014283	384.9808127
		+	20.821	0.0340844	385.2191156
208.204	-208.204				
269.241	-269.241	+	269.277	0.0167986	385.2556900
		+	256.154	0.0187175	385.9841607
25.613	-25.613				
210.489	-210.489	+	210.496	0.0328475	386.0260292
		+	306.227	0.0127969	386.0470419
306.117	-306.117				
195.987	-195.987	+	195.991	0.0406015	386.1090639
		+	185.753	0.0492866	386.1113788
18.575	-18.575				
198.521	-198.521	+	198.526	0.0395364	386.2150057
		+	254.163	0.0187189	386.2872316
25.414	-25.414				
229.991	-229.991	+	230.002	0.024425	387.0520416
		+	228.755	0.0246425	387.2350681
228.744	-228.744				
269.954	-269.954	+	269.991	0.0166512	388.1028036
		+	200.819	0.0384145	388.2553927
200.815	-200.815				
206.205	-206.205	+	20.621	0.0349518	390.9660484
		+	191.959	0.0437812	392.0149188
191.955	-191.955				
277.664	-277.664	+	27.771	0.0154906	392.0340217
		+	23.575	0.0231719	393.2241452

235.737	-235.737				
217.561	-217.561	+	217.568	0.0291959	396.2350646
		+	217.826	0.0290191	396.2587633
217.818	-217.818				
222.633	-222.633	+	222.642	0.0264786	396.2605670
		+	202.893	0.0367898	397.2444859
202.888	-202.888				
203.584	-203.584	+	203.589	0.0362649	397.2555976
		+	229.618	0.0246784	398.2508717
229.607	-229.607				
243.286	-243.286	+	243.302	0.0220576	398.2759719
		+	26.169	0.0191076	399.0992456
261.662	-261.662				
197.411	-197.411	+	197.416	0.0401065	399.1269906
		+	266.083	0.0186553	399.2350157
26.605	-26.605				
222.687	-222.687	+	222.696	0.0263035	399.9711018
		+	214.213	0.0312997	400.1345336
214.206	-214.206				
255.235	-255.235	+	255.259	0.018888	400.2286722
		+	234.997	0.0236007	400.2307765
234.985	-234.985				
243.456	-243.456	+	243.473	0.0217347	400.3418830
		+	190.716	0.044481	400.9409852
190.713	-190.713				
216.505	-216.505	+	216.512	0.0299322	400.9546376
		+	210.542	0.0328664	401.3440434
210.536	-210.536				
235.282	-235.282	+	235.295	0.023221	402.0219290
		+	221.402	0.027135	402.0926660
221.393	-221.393				
205.502	-205.502	+	205.507	0.0352961	402.2458644
		+	199.516	0.0389558	403.0282536
199.511	-199.511				
204.815	-204.815	+	20.482	0.0356967	404.1179710
		+	236.259	0.0232536	404.1207901
236.246	-236.246				
263.152	-263.152	+	263.182	0.018963	406.9626552

		+	18.561	0.0493716	407.1127429
185.607	-185.607				
227.404	-227.404	+	227.415	0.0251613	407.2387980
		+	241.195	0.0215831	407.2415284
24.118	-24.118				
285.232	-285.232	+	28.529	0.014181	408.0079177
		+	319.021	0.0146316	408.0288668
318.858	-318.858				
239.043	-239.043	+	239.058	0.0224399	408.1272765
		+	196.473	0.0404598	408.9920403
196.469	-196.469				
223.274	-223.274	+	223.283	0.0263976	409.0122195
		+	203.624	0.036304	410.0058736
203.619	-203.619				
202.518	-202.518	+	202.523	0.0370021	410.9853245
25.086	-25.086	+	25.088	0.0188788	411.2349859
19.603	-19.603	+	196.034	0.0405728	411.3077645
		+	2.598	0.0186849	412.1781658
259.773	-259.773				
238.543	-238.543	+	238.557	0.022336	412.2916574
		+	19.981	0.0388269	413.2482695
199.805	-199.805				
19.543	-19.543	+	195.434	0.0411283	413.2509879
		+	320.605	0.0149213	413.3234542
320.434	-320.434				
27.578	-27.578	+	275.824	0.0158431	414.0140309
		+	232.949	0.0235309	414.2457787
232.937	-232.937				
227.233	-227.233	+	227.243	0.0251006	414.2821627
		+	233.676	0.0237389	414.3071631
233.664	-233.664				
3.342	-3.342	+	334.463	0.00937063	414.3268243
		+	187.883	0.0467291	415.0759662
18.788	-18.788				
187.153	-187.153	+	187.156	0.0477907	415.2433265
		+	207.848	0.0343742	415.2663034

207.842	-207.842				
23.488	-23.488	+	234.893	0.023513	415.3306350
		+	193.547	0.0421403	416.2252921
193.543	-193.543				
207.231	-207.231	+	207.236	0.0344721	416.2614539
		+	214.146	0.0313036	417.2455632
214.139	-214.139				
241.573	-241.573	+	241.588	0.0217441	418.1965813
		+	205.814	0.0351666	419.0024232
205.809	-205.809				
207.875	-207.875	+	207.881	0.034394	419.2761924
		+	233.701	0.0237604	422.2146427
233.688	-233.688				
227.456	-227.456	+	227.466	0.0252019	422.9228193
		+	204.151	0.0363176	422.9365216
204.146	-204.146				
202.762	-202.762	+	202.767	0.036783	423.2714329
		+	2.393	0.0221863	423.9818616
239.285	-239.285				
25.777	-25.777	+	257.795	0.0186261	424.0028244
		+	193.001	0.0426036	424.1952666
192.997	-192.997				
313.459	-313.459	+	313.597	0.0146047	424.2916802
		+	203.663	0.0364022	424.9319025
203.658	-203.658				
197.527	-197.527	+	197.531	0.0400849	424.9862442
		+	203.061	0.0368367	425.0112169
203.056	-203.056				
197.785	-197.785	+	197.789	0.0398177	426.1028049
		+	189.026	0.0456849	426.2708884
189.022	-189.022				
288.168	-288.168	+	288.232	0.0145398	426.2820947
		+	235.552	0.0232514	426.9910893
235.539	-235.539				
186.274	-186.274	+	186.277	0.0487533	427.0854725
		+	283.317	0.0152685	427.2660383

283.262	-283.262				
258.909	-258.909	+	258.935	0.0181329	427.3025803
		+	215.321	0.0306285	427.9944823
215.314	-215.314				
184.779	-184.779	+	184.782	0.0498311	428.0363803
		+	237.367	0.0226797	428.2615234
237.353	-237.353				
222.065	-222.065	+	222.074	0.0267636	428.2976742
		+	186.526	0.0484489	429.1150460
186.523	-186.523				
186.344	-186.344	+	186.347	0.0486874	429.2224794
		+	28.662	0.0145672	429.2566000
28.656	-28.656				
299.817	-299.817	+	299.908	0.0135375	429.2819732
		+	30.688	0.013098	429.2973105
306.768	-306.768				
238.224	-238.224	+	238.239	0.0224119	429.9893541
23.888	-23.888	+	238.894	0.0223822	429.9905159
		+	233.742	0.023847	430.0108146
23.373	-23.373				
237.088	-237.088	+	237.102	0.0227638	430.2655765
		+	193.642	0.0421049	430.2769317
193.638	-193.638				
234.568	-234.568	+	234.581	0.0234968	430.3017584
192.052	-192.052	+	192.056	0.0436586	434.1166813
		+	185.246	0.0495052	436.2060275
185.243	-185.243				
224.525	-224.525	+	224.534	0.0264259	436.2321733
		+	353.917	0.00907865	438.2975672
353.426	-353.426				
202.763	-202.763	+	202.768	0.0368025	439.2258018
		+	210.576	0.0328862	439.2659599
21.057	-21.057				
207.354	-207.354	+	20.736	0.0343913	439.3018409
		+	210.502	0.032887	439.3388827
210.496	-210.496				
206.958	-206.958	+	206.963	0.0346901	440.1774988

		+	220.396	0.0277509	440.2976626
220.388	-220.388				
280.535	-280.535	+	280.586	0.0153013	440.3132441
		+	220.134	0.0276529	440.9842836
220.125	-220.125				
232.924	-232.924	+	232.936	0.0235098	441.2566496
		+	246.444	0.0202824	441.2817288
246.426	-246.426				
227.346	-227.346	+	227.356	0.0251208	441.3174311
		+	206.662	0.0347984	441.3547131
206.657	-206.657				
192.628	-192.628	+	192.631	0.0428017	442.2670750
		+	2.105	0.0328673	442.2768774
210.494	-210.494				
223.092	-223.092	+	223.101	0.0263383	442.9650235
		+	209.938	0.033262	443.2614299
209.932	-209.932				
21.701	-21.701	+	217.017	0.0293905	443.2730233
		+	194.694	0.0417257	443.9681757
19.469	-19.469				
241.351	-241.351	+	241.367	0.0216748	444.1537417
		+	193.313	0.0423363	444.2927847
193.309	-193.309				
203.522	-203.522	+	203.527	0.0362602	445.1684585
		+	264.304	0.0192039	445.9638803
264.273	-264.273				
235.028	-235.028	+	235.041	0.023436	445.9847043
		+	241.358	0.0216518	448.9730364
241.342	-241.342				
240.942	-240.942	+	240.957	0.0215747	449.9763814
		+	206.899	0.0348209	450.0180281
206.894	-206.894				
228.103	-228.103	+	228.113	0.0250619	450.2118890
		+	188.366	0.0463126	450.2448764
188.363	-188.363				
204.841	-204.841	+	204.846	0.035681	450.9773101
		+	262.254	0.0190016	451.0154839

262.225	-262.225				
215.098	-215.098	+	215.106	0.030734	452.0231491
		+	213.826	0.0310309	452.1046651
213.819	-213.819				
188.978	-188.978	+	188.982	0.0456906	455.2027155
		+	238.739	0.0222472	455.2722951
238.725	-238.725				
314.534	-314.534	+	314.676	0.0141232	456.1625690
		+	278.313	0.0153689	456.2927310
278.266	-278.266				
225.204	-225.204	+	225.213	0.025936	456.3287382
		+	21.333	0.031152	456.9579156
213.324	-213.324				
190.933	-190.933	+	190.937	0.0442971	457.2757801
		+	222.826	0.0263358	457.2877344
222.817	-222.817				
215.586	-215.586	+	215.593	0.0303126	457.4075896
		+	185.988	0.0492228	458.2724329
185.985	-185.985				
215.597	-215.597	+	215.604	0.0303067	459.2673150
		+	195.775	0.040929	459.2927604
195.771	-195.771				
188.277	-188.277	+	18.828	0.0464155	460.1026418
		+	31.187	0.0142731	460.2791995
31.174	-31.174				
195.231	-195.231	+	195.235	0.0412299	462.0170817
		+	24.056	0.0216712	462.2447773
240.544	-240.544				
222.893	-222.893	+	222.902	0.0263946	462.2957802
		+	250.323	0.0188385	464.9468527
250.303	-250.303				
197.258	-197.258	+	197.263	0.0401632	465.9496137
		+	197.299	0.0402226	465.9504668
197.295	-197.295				
19.858	-19.858	+	198.585	0.0395761	465.9921872
		+	198.901	0.0392355	466.0741327
198.896	-198.896				
296.519	-296.519	+	296.601	0.0149415	466.1355194

		+	197.024	0.0402807	466.2762327
197.019	-197.019				
212.996	-212.996	+	213.003	0.0310441	466.3651761
		+	203.494	0.0362407	466.9450570
203.489	-203.489				
224.917	-224.917	+	224.927	0.0258526	466.9514099
		+	212.923	0.0310056	467.1372823
212.916	-212.916				
198.979	-198.979	+	198.983	0.0392158	468.3288165
		+	201.349	0.0381189	468.3444660
201.344	-201.344				
229.582	-229.582	+	229.593	0.0246164	469.2877387
		+	19.521	0.0411904	469.3481597
195.207	-195.207				
248.573	-248.573	+	248.592	0.0191176	470.9548876
		+	347.958	0.0100594	471.2566480
347.553	-347.553				
264.728	-264.728	+	264.759	0.0188033	471.2923334
		+	276.265	0.0157916	471.3285208
276.221	-276.221				
230.939	-230.939	+	230.951	0.0239379	471.9580288
		+	224.494	0.0264419	471.9994139
224.485	-224.485				
224.497	-224.497	+	224.506	0.0264622	472.0002670
		+	277.892	0.0154105	472.2868622
277.846	-277.846				
320.249	-320.249	+	320.419	0.014838	472.3238912
		+	253.899	0.0187239	472.9588144
253.877	-253.877				
207.693	-207.693	+	207.699	0.034492	473.0037055
		+	242.754	0.0219254	473.2847440
242.738	-242.738				
204.496	-204.496	+	204.501	0.0362776	474.0045700
		+	196.447	0.0404401	474.0159106
196.443	-196.443				
202.467	-202.467	+	202.472	0.0370688	475.2548095
		+	186.719	0.0480186	476.0134401

186.716	-186.716				
307.371	-307.371	+	307.485	0.0133068	476.2967653
		+	215.655	0.030273	478.2758423
215.648	-215.648				
188.946	-188.946	+	188.949	0.0456707	478.3011640
		+	21.991	0.0277479	479.2726106
219.902	-219.902				
198.217	-198.217	+	198.221	0.0397092	480.2558867
		+	201.022	0.0383664	480.9208522
201.018	-201.018				
240.963	-240.963	+	240.979	0.0215975	482.1092449
		+	228.947	0.0247444	482.1113900
228.937	-228.937				
19.354	-19.354	+	193.544	0.0421206	482.2622400
		+	226.903	0.0250999	483.0416141
226.893	-226.893				
249.505	-249.505	+	249.525	0.018931	483.2167812
		+	214.784	0.03102	484.0859678
214.777	-214.777				
187.606	-187.606	+	187.609	0.047133	484.2844941
		+	19.558	0.0410145	484.3239635
195.576	-195.576				
227.728	-227.728	+	227.738	0.0252026	486.3034144
		+	256.014	0.0186911	486.9285230
25.599	-25.599				
186.174	-186.174	+	186.177	0.0488631	487.2428955
		+	243.353	0.0218868	487.2628471
243.337	-243.337				
194.302	-194.302	+	194.306	0.0417565	487.2850342
		+	220.545	0.0274695	487.3591145
220.537	-220.537				
217.854	-217.854	+	217.862	0.0290383	487.3606525
		+	23.386	0.0238905	487.9322843
233.848	-233.848				
238.875	-238.875	+	238.889	0.0223596	487.9740880
		+	299.592	0.0144675	488.1175832
299.502	-299.502				
212.275	-212.275	+	212.281	0.0313125	488.9268246

		+	207.783	0.0345316	488.9773755
207.778	-207.778				
199.645	-199.645	+	19.965	0.0389013	489.1141781
		+	229.313	0.024598	490.1167538
229.302	-229.302				
330.307	-330.307	+	33.054	0.00993377	491.2360630
		+	253.659	0.0187413	492.2317340
253.637	-253.637				
256.776	-256.776	+	256.801	0.0188775	492.9367873
		+	192.129	0.0435539	493.2875976
192.125	-192.125				
239.941	-239.941	+	239.956	0.0220812	493.9820960
		+	205.128	0.0354879	494.9855052
205.123	-205.123				
21.611	-21.611	+	216.117	0.0301504	495.1024105
		+	209.129	0.0338861	496.2870930
209.123	-209.123				
185.154	-185.154	+	185.157	0.0495512	496.3030180
		+	215.208	0.0306788	497.3188764
215.201	-215.201				
190.551	-190.551	+	190.555	0.044714	498.0771692
		+	210.487	0.0328278	498.3032085
21.048	-21.048				
186.495	-186.495	+	186.498	0.0483877	499.0152152
		+	338.763	0.00976378	499.1861957
338.462	-338.462				
209.332	-209.332	+	209.338	0.0336843	499.2859946
		+	188.068	0.0466833	499.3585465
188.065	-188.065				
192.967	-192.967	+	192.971	0.0425642	499.3608990
		+	185.759	0.0493072	500.0827569
185.756	-185.756				
265.339	-265.339	+	265.371	0.018604	500.1886221
		+	232.119	0.0235336	500.1918946
232.107	-232.107				
29.241	-29.241	+	292.482	0.0153029	500.2533406
		+	214.068	0.0312676	501.1833033

214.061	-214.061				
187.615	-187.615	+	187.618	0.0471531	501.3385078
		+	229.558	0.0245753	502.2536344
229.547	-229.547				
185.243	-185.243	+	185.246	0.0495256	502.3051904
		+	230.582	0.0241607	502.9027250
230.571	-230.571				
198.936	-198.936	+	19.894	0.0392069	503.3173740
		+	205.566	0.0351907	503.9478886
205.561	-205.561				
242.366	-242.366	+	242.382	0.0219283	504.0648212
		+	302.611	0.0129965	504.0920042
302.512	-302.512				
270.053	-270.053	+	27.009	0.016681	505.0226021
		+	324.461	0.0136667	506.0259234
324.268	-324.268				
26.041	-26.041	+	260.438	0.0188775	506.0678012
		+	215.079	0.0307788	506.2639174
215.072	-215.072				
231.947	-231.947	+	231.959	0.0236907	507.0712926
		+	207.054	0.0347319	508.0628544
207.049	-207.049				
219.719	-219.719	+	219.727	0.0278028	508.0837408
		+	226.436	0.0256574	508.3030031
226.426	-226.426				
249.571	-249.571	+	24.959	0.0190012	508.9107033
		+	20.392	0.0364664	509.2446013
203.915	-203.915				
198.269	-198.269	+	198.273	0.0398083	509.3064971
		+	276.085	0.015666	509.4923252
276.041	-276.041				
242.957	-242.957	+	242.973	0.0219603	509.9559624
		+	257.438	0.0184921	510.3033008
257.413	-257.413				
24.345	-24.345	+	243.466	0.0218486	510.9589973
		+	230.103	0.0243384	511.0960688
230.092	-230.092				
339.423	-339.423	+	339.734	0.00992	511.3346226

		+	196.297	0.0405756	511.9539014
196.293	-196.293				
207.162	-207.162	+	207.167	0.0346378	512.0983553
		+	260.643	0.0189068	512.1412743
260.616	-260.616				
223.944	-223.944	+	223.953	0.0263805	512.2944285
22.436	-22.436	+	224.369	0.0264012	513.2177220
		+	22.836	0.0248242	513.3507849
22.835	-22.835				
239.562	-239.562	+	239.577	0.0222245	514.2131749
203.574	-203.574	+	203.579	0.0362454	514.3489699
		+	203.752	0.0364416	514.3508044
203.747	-203.747				
226.084	-226.084	+	226.093	0.0257908	515.2130260
		+	186.286	0.0487738	515.3308164
186.283	-186.283				
232.633	-232.633	+	232.645	0.0233636	515.9640046
		+	300.038	0.0136711	516.2846723
299.947	-299.947				
304.165	-304.165	+	304.268	0.0132464	516.3135199
		+	213.008	0.0310634	517.2873143
213.002	-213.002				
293.228	-293.228	+	293.302	0.0149017	518.2258082
		+	201.662	0.0378184	519.2056874
201.657	-201.657				
268.918	-268.918	+	268.954	0.0169296	520.2623710
		+	243.882	0.0214231	520.3158592
243.865	-243.865				
2.708	-2.708	+	270.838	0.01656	520.9964662
		+	307.753	0.0134137	521.1678862
307.638	-307.638				
342.182	-342.182	+	342.522	0.0100885	521.3190212
		+	388.069	0.00869565	521.3222112
386.504	-386.504				
213.194	-213.194	+	213.201	0.0311172	521.9973220
		+	285.352	0.014283	522.0023525

285.294	-285.294				
234.179	-234.179	+	234.192	0.0236213	522.0417819
		+	239.669	0.0222934	522.0627633
239.654	-239.654				
358.978	-358.978	+	359.569	0.00961905	522.1718404
		+	194.467	0.0416346	523.0450172
194.463	-194.463				
21.666	-21.666	+	216.667	0.0297653	523.1643400
		+	191.939	0.0437869	524.0174734
191.936	-191.936				
216.431	-216.431	+	216.438	0.0299788	524.0572925
		+	232.162	0.0234695	524.2353085
23.215	-23.215				
207.043	-207.043	+	207.048	0.0347493	524.8845953
		+	185.671	0.0493083	525.2170412
185.668	-185.668				
209.021	-209.021	+	209.027	0.0338445	525.9294738
		+	209.109	0.0338067	525.9304281
209.103	-209.103				
185.205	-185.205	+	185.208	0.0495716	525.9516197
		+	216.468	0.0299987	526.0626037
216.461	-216.461				
278.578	-278.578	+	278.625	0.0154347	526.3343889
		+	192.178	0.0435738	527.0038044
192.174	-192.174				
18.475	-18.475	+	184.753	0.0498313	527.0438243
		+	281.972	0.0151837	528.0483892
28.192	-28.192				
281.846	-281.846	+	281.898	0.0151151	528.0502937
		+	234.829	0.0234912	528.1883354
234.817	-234.817				
275.992	-275.992	+	276.036	0.015604	529.1661204
		+	368.158	0.0078125	529.1922627
36.737	-36.737				
206.392	-206.392	+	206.398	0.0349573	529.2348227
		+	250.929	0.0189266	530.0439925

250.908	-250.908					
293.619	-293.619		+	293.694	0.014407	530.2848128
			+	242.397	0.0219522	530.8925710
242.381	-242.381					
227.806	-227.806		+	227.816	0.0251623	531.2885429
			+	26.465	0.0187414	531.9377890
264.619	-264.619					
207.344	-207.344		+	207.349	0.034352	533.0763428
			+	305.236	0.0132687	534.2950830
305.129	-305.129					
203.118	-203.118		+	203.123	0.0367889	535.1577010
			+	196.194	0.0404772	535.1620829
19.619	-19.619					
195.303	-195.303		+	195.307	0.0411933	535.1777971
			+	288.678	0.0146528	535.2984612
288.614	-288.614					
19.814	-19.814		+	198.145	0.0397194	536.0770938
			+	270.536	0.0166209	536.2937025
270.499	-270.499					
275.789	-275.789		+	275.833	0.0158743	536.3320580
			+	293.739	0.0144912	537.1421168
293.664	-293.664					
240.037	-240.037		+	240.052	0.0220459	537.3362190
			+	187.313	0.0475959	537.5236130
18.731	-18.731					
192.637	-192.637		+	192.641	0.0428214	538.0364723
			+	201.057	0.0383543	538.5268719
201.052	-201.052					
233.689	-233.689		+	233.702	0.023782	539.3659446
			+	18.749	0.0472693	540.1723321
187.487	-187.487					
194.135	-194.135		+	194.138	0.0417524	540.2085753
			+	202.526	0.0370217	540.3486362
202.521	-202.521					
191.246	-191.246		+	191.249	0.0440631	541.0249893
21.423		-21.423	+	214.237	0.0312174	541.3452018
			+	217.487	0.0291367	541.3817509

21.748	-21.748				
218.093	-218.093	+	218.101	0.0286753	542.0678778
		+	187.554	0.0472243	542.2040038
187.551	-187.551				
25.721	-25.721	+	257.235	0.018439	542.2537968
218.101	-218.101	+	218.109	0.0286949	543.0714808
		+	214.131	0.0312438	543.1497000
214.124	-214.124				
214.124	-214.124	+	214.131	0.0312637	543.1503189
		+	227.701	0.0251822	543.2466865
227.691	-227.691				
298.965	-298.965	+	299.053	0.014328	544.0072391
		+	28.617	0.0146339	544.0236943
286.111	-286.111				
292.274	-292.274	+	292.346	0.0152159	544.0446868
		+	210.193	0.0331446	544.0727318
210.186	-210.186				
19.876	-19.876	+	198.765	0.0394706	544.0835583
		+	222.812	0.0262967	544.3154045
222.803	-222.803				
258.579	-258.579	+	258.606	0.0181201	545.1458843
		+	192.424	0.0432239	546.0415598
192.421	-192.421				
254.036	-254.036	+	254.059	0.0186685	546.3323971
		+	21.482	0.030911	547.0442109
214.813	-214.813				
185.307	-185.307	+	18.531	0.0496076	547.2981629
		+	22.415	0.0264533	548.0550754
224.141	-224.141				
259.325	-259.325	+	259.352	0.0184048	548.3470003
		+	193.399	0.0422146	549.3135132
193.395	-193.395				
229.034	-229.034	+	229.045	0.0246821	549.3388997
		+	297.164	0.01455	549.3421062
29.708	-29.708				
190.559	-190.559	+	190.563	0.0447339	549.3517816
		+	214.361	0.0311293	552.0258186

214.354	-214.354				
295.923	-295.923	+	296.004	0.0147599	552.2654667
		+	185.407	0.049414	553.2728896
185.404	-185.404				
23.738	-23.738	+	237.394	0.0227019	554.3422188
		+	198.746	0.0394507	555.3614766
198.742	-198.742				
192.762	-192.762	+	192.766	0.0427583	556.2975021
		+	193.831	0.041893	556.3452217
193.827	-193.827				
267.906	-267.906	+	26.794	0.0179024	556.3563641
		+	187.725	0.0469923	557.3412612
187.722	-187.722				
199.235	-199.235	+	199.239	0.0391213	557.3995828
		+	198.467	0.0396072	557.4006975
198.462	-198.462				
296.125	-296.125	+	296.206	0.0148502	558.0636035
		+	278.656	0.0154678	559.1243125
278.608	-278.608				
304.071	-304.071	+	304.174	0.0135596	559.9972858
		+	296.666	0.0149877	560.0188656
296.584	-296.584				
306.811	-306.811	+	306.924	0.0132016	560.0787790
		+	329.801	0.0103896	560.1276611
329.573	-329.573				
25.745	-25.745	+	257.475	0.0185187	561.0823944
		+	280.423	0.015268	561.1218320
280.372	-280.372				
213.494	-213.494	+	213.501	0.0309994	561.4487662
		+	192.517	0.0428871	562.2744630
192.514	-192.514				
252.406	-252.406	+	252.428	0.018766	562.3266530
		+	23.686	0.0231365	563.0073022
236.846	-236.846				
236.193	-236.193	+	236.207	0.0232314	563.3287107
		+	232.916	0.0234888	563.3308646
232.904	-232.904				
361.818	-361.818	+	362.469	0.0104935	563.4644585

		+	204.233	0.0363224	564.0109581
204.228	-204.228				
218.776	-218.776	+	218.784	0.0282885	564.0498123
		+	218.702	0.0283485	564.0504073
218.694	-218.694				
216.528	-216.528	+	216.536	0.0299521	564.2482075
		+	223.824	0.0263921	565.0536903
223.815	-223.815				
229.589	-229.589	+	2.296	0.024637	565.2524526
		+	197.818	0.039877	566.0258827
197.814	-197.814				
198.253	-198.253	+	198.257	0.0397686	566.0550421
		+	230.441	0.0242901	566.2642096
23.043	-23.043				
259.214	-259.214	+	25.924	0.0183224	566.3659827
		+	238.794	0.0223145	566.9961546
238.779	-238.779				
202.281	-202.281	+	202.286	0.0371664	568.0215592
		+	286.586	0.014495	568.0427494
286.525	-286.525				
261.381	-261.381	+	261.409	0.0191447	568.2416044
		+	206.745	0.0347767	569.2452660
20.674	-20.674				
256.514	-256.514	+	256.539	0.0188239	569.2816903
		+	208.442	0.0341833	570.0370911
208.436	-208.436				
189.727	-189.727	+	18.973	0.045348	570.3614143
		+	25.734	0.0184655	572.3526264
257.315	-257.315				
240.174	-240.174	+	240.189	0.0219874	574.0572575
		+	214.753	0.031	574.2879221
214.746	-214.746				
245.561	-245.561	+	245.579	0.0209188	575.0980131
		+	19.804	0.0397474	576.1025645
198.036	-198.036				
259.568	-259.568	+	259.595	0.0185158	576.3049014
		+	228.653	0.0245818	578.2045232

228.643	-228.643				
231.802	-231.802	+	231.814	0.0236953	578.9627202
		+	189.381	0.0455123	578.9817448
189.378	-189.378				
226.104	-226.104	+	226.114	0.0258113	580.0241576
		+	189.398	0.0455522	581.9968034
189.394	-189.394				
210.093	-210.093	+	210.099	0.0331054	582.0225394
		+	350.643	0.00917172	582.0596765
350.202	-350.202				
349.888	-349.888	+	350.325	0.00908	582.0608437
		+	188.188	0.0465478	582.3726019
188.185	-188.185				
286.294	-286.294	+	286.354	0.0144593	582.9538680
		+	200.122	0.0387301	583.0070943
200.118	-200.118				
255.188	-255.188	+	255.212	0.0188619	583.0641001
		+	294.319	0.0144955	583.2841137
294.243	-294.243				
217.301	-217.301	+	217.308	0.0292884	584.0159580
		+	197.999	0.0397576	584.0644731
197.995	-197.995				
247.234	-247.234	+	247.252	0.0202908	584.0764398
		+	226.956	0.02506	584.2855913
226.946	-226.946				
294.736	-294.736	+	294.814	0.0145389	584.3519835
		+	223.455	0.0264771	584.5247034
223.445	-223.445				
2.422	-2.422	+	242.216	0.0219179	584.9892559
		+	242.271	0.0219654	584.9908382
242.255	-242.255				
210.141	-210.141	+	210.147	0.0330853	585.3721158
		+	213.167	0.0310978	585.9927235
21.316	-21.316				
225.141	-225.141	+	225.151	0.0258754	586.0321873
		+	221.484	0.0270429	586.2295382
221.475	-221.475				
221.155	-221.155	+	221.163	0.0271259	586.2306359

		+	245.478	0.0210556	586.3321974
24.546	-24.546				
253.969	-253.969	+	253.991	0.0187742	587.0282385
		+	224.263	0.0263607	587.0356259
224.254	-224.254				
203.174	-203.174	+	203.179	0.0367037	587.2338225
		+	19.022	0.0449284	588.0366967
190.217	-190.217				
200.327	-200.327	+	200.331	0.0386166	588.2460760
		+	236.614	0.023433	588.3059182
2.366	-2.366				
199.116	-199.116	+	19.912	0.0391631	589.3085840
		+	230.864	0.0238557	589.4797142
230.852	-230.852				
230.852	-230.852	+	230.864	0.0238762	589.4803551
		+	239.049	0.0223943	590.4834383
239.035	-239.035				
193.963	-193.963	+	193.967	0.0418604	590.9139552
		+	18.763	0.0471733	591.4746881
187.626	-187.626				
209.291	-209.291	+	209.297	0.0337624	591.4867681
		+	287.726	0.0146837	591.4957920
287.663	-287.663				
253.423	-253.423	+	253.446	0.0187164	592.0186349
		+	186.911	0.0479288	592.1313554
186.907	-186.907				
300.483	-300.483	+	300.576	0.0138997	592.4989966
		+	298.234	0.0144177	592.5003822
298.147	-298.147				
207.359	-207.359	+	207.365	0.034411	593.4908570
22.208	-22.208	+	222.089	0.0266363	594.0034648
		+	186.953	0.0479474	596.0391479
18.695	-18.695				
239.122	-239.122	+	239.137	0.0222589	596.3157255
187.754	-187.754	+	187.757	0.0469164	597.3721310
		+	24.761	0.0202926	598.0345977

247.591	-247.591				
198.413	-198.413	+	198.418	0.0396375	598.3670529
		+	207.553	0.0344326	599.0037558
207.547	-207.547				
194.289	-194.289	+	194.293	0.0417367	599.3874479
		+	240.627	0.0216939	600.9632497
240.612	-240.612				
188.622	-188.622	+	188.625	0.0461864	601.0174603
		+	220.918	0.0272502	601.9666350
220.909	-220.909				
238.486	-238.486	+	2.385	0.022247	602.0060982
		+	308.628	0.0136885	602.2046757
30.851	-30.851				
207.047	-207.047	+	207.052	0.0347691	602.2444533
		+	289.362	0.0147374	602.3238734
289.296	-289.296				
195.501	-195.501	+	195.505	0.0411594	603.0093191
		+	195.272	0.0411538	603.0104554
195.268	-195.268				
207.348	-207.348	+	207.354	0.0343716	603.0937094
		+	238.634	0.0222026	604.0132644
238.619	-238.619				
362.497	-362.497	+	363.163	0.00956757	604.0427535
		+	3.202	0.0154444	605.0457305
320.031	-320.031				
254.366	-254.366	+	254.389	0.0186089	605.4540747
		+	210.152	0.0331248	605.9635680
210.146	-210.146				
24.931	-24.931	+	24.933	0.0189608	606.3352852
		+	20.208	0.0373481	606.4559021
202.075	-202.075				
242.362	-242.362	+	242.378	0.0219046	606.9711961
		+	24.695	0.0201467	607.4691926
246.931	-246.931				
24.537	-24.537	+	245.387	0.0213533	607.4705401
		+	24.279	0.0219495	607.9745699
242.773	-242.773				
219.223	-219.223	+	219.231	0.0277909	607.9933384

		+	242.476	0.022	608.0148690
24.246	-24.246				
187.147	-187.147	+	18.715	0.0477501	608.3056736
		+	217.075	0.0293432	609.0180744
217.068	-217.068				
213.872	-213.872	+	213.879	0.0310701	609.0216763
		+	193.863	0.041811	610.0182528
193.859	-193.859				
18.685	-18.685	+	186.853	0.0479932	610.0221222
		+	200.148	0.0387185	613.4032968
200.143	-200.143				
220.008	-220.008	+	220.017	0.0277163	614.3226420
		+	225.585	0.0258331	616.3367637
225.575	-225.575				
191.149	-191.149	+	191.152	0.0441888	616.9370848
		+	241.591	0.0217673	616.9930902
241.575	-241.575				
275.567	-275.567	+	27.561	0.0157505	617.3617197
		+	207.024	0.0347295	617.5113599
207.018	-207.018				
189.475	-189.475	+	189.478	0.0455448	617.9795410
		+	189.447	0.0455722	617.9804564
189.444	-189.444				
195.749	-195.749	+	195.753	0.0409093	618.3033573
		+	224.982	0.025795	618.3650608
224.972	-224.972				
243.882	-243.882	+	243.899	0.0214472	619.0569279
33.989	-33.989	+	340.206	0.00926829	619.3680979
18.882	-18.882	+	188.824	0.0458921	619.5180740
		+	313.294	0.01447	620.0166059
313.157	-313.157				
220.324	-220.324	+	220.332	0.0276717	620.0613831
		+	201.658	0.0377985	620.1022463
201.654	-201.654				
216.961	-216.961	+	216.968	0.0296118	620.3525307
		+	1.982	0.0397392	620.5291987

198.196	-198.196				
194.088	-194.088	+	194.092	0.0417602	620.5308062
		+	192.099	0.0436071	621.0189485
192.096	-192.096				
416.692	-416.692	+	421.333	0.00833333	622.2954315
		+	242.891	0.022022	622.9451188
242.875	-242.875				
193.469	-193.469	+	193.473	0.0421606	623.1260634
		+	240.113	0.022069	623.9881069
240.098	-240.098				
234.116	-234.116	+	234.129	0.0236587	623.9909712
		+	27.108	0.0165902	624.9824439
271.042	-271.042				
198.834	-198.834	+	198.839	0.039375	624.9924798
		+	215.161	0.0307289	625.0053099
215.154	-215.154				
187.804	-187.804	+	187.808	0.046864	625.4032252
		+	206.011	0.0350609	625.9932789
206.006	-206.006				
359.546	-359.546	+	360.149	0.0101	626.0242974
		+	188.289	0.0464355	626.0462694
188.286	-188.286				
226.422	-226.422	+	226.432	0.0256369	627.0041761
		+	212.724	0.0310735	627.0271793
212.717	-212.717				
217.123	-217.123	+	217.131	0.0293826	628.2850863
		+	214.181	0.0313235	628.3369038
214.174	-214.174				
207.408	-207.408	+	207.414	0.0343734	629.9983867
		+	240.633	0.0217167	630.3540195
240.618	-240.618				
189.967	-189.967	+	18.997	0.0449376	630.3875097
		+	218.299	0.028489	631.0022828
218.292	-218.292				
281.852	-281.852	+	281.905	0.0151493	633.4853143
		+	235.491	0.0233302	634.3161302

235.478	-235.478				
312.602	-312.602	+	312.737	0.0142081	634.3294522
		+	312.379	0.0145946	634.3306982
312.247	-312.247				
261.332	-261.332	+	26.136	0.0190846	634.4883133
		+	2.164	0.0299589	635.3262609
216.392	-216.392				
245.382	-245.382	+	2.454	0.021378	635.3341110
		+	214.516	0.0309448	635.3741220
214.509	-214.509				
195.388	-195.388	+	195.392	0.0411566	635.5010696
		+	209.574	0.0334814	636.0736077
209.568	-209.568				
249.733	-249.733	+	249.753	0.019072	638.9164228
		+	233.349	0.0235892	639.3437022
233.337	-233.337				
186.672	-186.672	+	186.675	0.0480422	639.9622761
		+	210.675	0.0325883	640.0044708
210.669	-210.669				
195.445	-195.445	+	195.449	0.0411679	640.3771987
		+	227.049	0.025	641.3523841
227.038	-227.038				
194.127	-194.127	+	19.413	0.0417326	641.4151630
		+	31.225	0.0145291	641.9984508
312.117	-312.117				
328.591	-328.591	+	328.812	0.0120764	642.0016659
		+	243.413	0.0218242	642.0841528
243.396	-243.396				
221.324	-221.324	+	221.332	0.0271153	642.3545372
		+	264.017	0.0193049	644.3684020
263.987	-263.987				
230.291	-230.291	+	230.303	0.0241664	645.3721056
		+	223.663	0.0263662	645.9559747
223.654	-223.654				
211.136	-211.136	+	211.143	0.0319927	645.9723734
		+	246.331	0.0203044	646.3170701
246.313	-246.313				
250.611	-250.611	+	250.631	0.0188858	646.9637488

		+	213.719	0.031034	646.9872835
213.713	-213.713				
316.015	-316.015	+	316.164	0.0140488	647.1134183
		+	25.173	0.0187336	648.3652702
251.709	-251.709				
190.165	-190.165	+	190.168	0.0449329	649.1053451
		+	192.887	0.0426778	649.3658060
192.884	-192.884				
213.997	-213.997	+	214.004	0.0311883	652.0525985
		+	301.714	0.0134558	652.1155129
301.618	-301.618				
191.561	-191.561	+	191.564	0.0438427	653.0559220
		+	211.571	0.0316129	653.3252791
211.564	-211.564				
190.156	-190.156	+	190.159	0.044913	653.3972896
		+	232.291	0.0234903	654.3277790
232.279	-232.279				
231.695	-231.695	+	231.707	0.0236538	654.3539431
		+	225.022	0.0258151	655.3174635
225.012	-225.012				
22.575	-22.575	+	22.576	0.0258942	655.4672325
		+	300.423	0.0138533	656.3126564
300.331	-300.331				
260.178	-260.178	+	260.205	0.0188862	656.3682871
		+	214.115	0.0312239	656.4707940
214.108	-214.108				
285.926	-285.926	+	285.986	0.0145268	657.2968404
		+	238.836	0.022337	657.3159645
238.821	-238.821				
205.026	-205.026	+	205.031	0.035559	657.3881396
		+	189.665	0.0453721	657.3926386
189.662	-189.662				
234.599	-234.599	+	234.612	0.0234259	657.9725996
		+	341.156	0.00942149	658.0787278
34.083	-34.083				
327.535	-327.535	+	327.749	0.0123975	658.0819829
		+	247.079	0.0202185	658.3173241

24.706	-24.706				
230.607	-230.607	+	230.618	0.0241814	661.3260822
		+	270.428	0.016741	661.5166485
270.391	-270.391				
292.767	-292.767	+	292.841	0.0148161	661.9306370
		+	193.504	0.0421803	662.5206024
1.935	-1.935				
249.682	-249.682	+	249.702	0.0190483	662.9370307
		+	186.517	0.0484285	664.5274557
186.514	-186.514				
244.526	-244.526	+	244.543	0.0216264	665.1004386
		+	191.204	0.0440936	665.3367967
1.912	-1.912				
327.734	-327.734	+	327.948	0.012475	666.0255485
		+	198.316	0.039818	666.1037764
198.312	-198.312				
219.533	-219.533	+	219.541	0.0278066	668.0265086
		+	213.333	0.0311715	668.9456914
213.327	-213.327				
199.878	-199.878	+	199.882	0.0387957	669.9888577
		+	202.153	0.0372778	669.9911040
202.148	-202.148				
218.817	-218.817	+	218.825	0.0283278	670.2684156
		+	206.308	0.034935	670.2726649
206.303	-206.303				
254.512	-254.512	+	254.535	0.0186848	670.3126695
		+	193.733	0.0419063	670.4244725
193.729	-193.729				
197.264	-197.264	+	197.268	0.040183	671.0871681
		+	306.387	0.0129457	671.4385133
306.276	-306.276				
267.466	-267.466	+	2.675	0.0179516	671.4421696
		+	294.281	0.0144524	672.2865831
294.204	-294.204				
232.449	-232.449	+	232.461	0.0234641	672.4442758
		+	204.961	0.0356518	673.2934699
204.955	-204.955				
21.303	-21.303	+	213.037	0.031102	674.0345285

		+	237.349	0.0226576	675.3071351
237.335	-237.335				
218.155	-218.155	+	218.163	0.0287539	678.0843076
		+	226.824	0.0252396	678.2952671
226.814	-226.814				
217.794	-217.794	+	217.802	0.0290476	678.5066671
		+	190.536	0.044694	678.5340931
190.532	-190.532				
201.583	-201.583	+	201.588	0.0378113	679.1252115
		+	24.641	0.020376	679.5095284
246.392	-246.392				
246.444	-246.444	+	246.462	0.0203062	679.5104966
		+	250.103	0.0189027	680.0435897
250.083	-250.083				
322.524	-322.524	+	322.707	0.0148605	681.0316180
		+	268.638	0.0170456	683.4047724
268.603	-268.603				
185.409	-185.409	+	185.412	0.0494345	683.5648530
		+	198.581	0.0395562	684.3984808
198.576	-198.576				
18.771	-18.771	+	187.713	0.047015	684.4564910
		+	319.153	0.014709	685.4192724
318.989	-318.989				
317.064	-317.064	+	317.218	0.0143284	685.4208259
		+	298.185	0.0143722	685.4584055
298.099	-298.099				
202.078	-202.078	+	202.083	0.0373678	685.4613222
		+	214.049	0.0312279	685.9648899
214.042	-214.042				
191.101	-191.101	+	191.104	0.0443378	686.3825567
		+	222.368	0.0264547	686.4155285
22.236	-22.236				
292.237	-292.237	+	292.309	0.0151728	686.4237502
		+	245.923	0.0208661	686.5090817
245.905	-245.905				
245.296	-245.296	+	245.313	0.0213287	686.5106124
		+	259.189	0.0182951	687.0822113

259.162	-259.162				
303.732	-303.732	+	303.835	0.0133191	687.2154575
		+	249.083	0.0191122	688.2825629
249.063	-249.063				
3.453	-3.453	+	345.676	0.0093211	688.3377238
		+	234.026	0.0236853	690.0084385
234.013	-234.013				
197.945	-197.945	+	197.949	0.0398173	690.1023212
		+	245.176	0.0212796	691.2776109
245.158	-245.158				
242.684	-242.684	+	2.427	0.0218536	691.2818075
		+	198.719	0.0394309	693.5622003
198.714	-198.714				
230.749	-230.749	+	230.761	0.0241578	694.2681040
		+	204.392	0.036253	695.4435459
204.386	-204.386				
261.634	-261.634	+	261.663	0.0190774	696.0948771
		+	264.754	0.0187723	697.0054594
264.723	-264.723				
290.258	-290.258	+	290.326	0.0147062	697.0978998
		+	221.445	0.0270036	698.4158003
221.436	-221.436				
2.59	-2.59	+	259.027	0.0182136	698.5564016
		+	222.069	0.0267439	699.0233920
222.061	-222.061				
202.896	-202.896	+	2.029	0.0368094	699.4549013
		+	221.711	0.026903	699.4638917
221.702	-221.702				
19.847	-19.847	+	198.475	0.0396271	700.0267579
		+	205.305	0.0354229	700.0669548
205.299	-205.299				
241.145	-241.145	+	24.116	0.0215375	700.4333991
		+	302.549	0.0129517	700.4762274
302.451	-302.451				
21.008	-21.008	+	210.086	0.0331452	700.4886686
		+	209.703	0.0334399	700.4906152
209.697	-209.697				
194.915	-194.915	+	194.919	0.041416	700.5159846

		+	188.139	0.0465456	701.4340989
188.135	-188.135				
230.275	-230.275	+	230.286	0.0242307	701.4921607
		+	202.761	0.0367635	702.0837254
202.756	-202.756				
257.818	-257.818	+	257.843	0.0186531	704.0161472
		+	248.673	0.019164	704.0584930
248.654	-248.654				
250.979	-250.979	+	2.51	0.0189506	704.0612150
		+	185.691	0.0493078	704.2552470
185.689	-185.689				
1.899	-1.899	+	189.903	0.0449863	705.3658369
		+	237.158	0.0227108	707.4024421
237.144	-237.144				
204.935	-204.935	+	204.941	0.0356322	707.5375351
		+	218.254	0.0285953	708.4054239
218.246	-218.246				
244.497	-244.497	+	244.514	0.0215773	709.0642025
		+	229.423	0.0245767	709.3973833
229.412	-229.412				
205.045	-205.045	+	20.505	0.0355786	711.4360967
		+	236.351	0.0233429	712.3573477
236.338	-236.338				
19.122	-19.122	+	191.224	0.0440684	712.4391648
		+	191.243	0.0440432	712.4403034
19.124	-19.124				
228.329	-228.329	+	228.339	0.0248039	713.4340928
		+	202.208	0.0372447	713.4428686
202.203	-202.203				
215.587	-215.587	+	215.595	0.0303325	713.4518465
		+	232.075	0.0235599	714.4551929
232.063	-232.063				
18.521	-18.521	+	185.212	0.0495921	714.4834896
		+	185.547	0.049371	714.9972680
185.544	-185.544				
232.412	-232.412	+	232.424	0.0234225	715.4868639
		+	209.828	0.0333412	715.4979111

209.822	-209.822				
185.549	-185.549	+	185.552	0.0493915	716.0412462
		+	212.414	0.0313087	716.3232072
212.407	-212.407				
34.401	-34.401	+	344.371	0.00915315	716.4626026
		+	206.667	0.0348181	716.4833072
206.661	-206.661				
293.927	-293.927	+	294.002	0.0143245	718.0769197
		+	203.386	0.0363412	718.1514296
203.381	-203.381				
197.615	-197.615	+	197.619	0.0401345	718.5384286
		+	309.286	0.013444	719.0787781
309.166	-309.166				
314.772	-314.772	+	314.916	0.0141905	719.0824539
		+	184.748	0.0498109	719.4067130
184.745	-184.745				
191.541	-191.541	+	191.544	0.0438482	719.5416219
		+	209.665	0.0333806	720.0154026
209.659	-209.659				
242.718	-242.718	+	242.734	0.0219014	720.0324334
		+	245.149	0.0212552	720.0535881
245.132	-245.132				
188.082	-188.082	+	188.085	0.0467034	720.1628379
		+	19.629	0.0405559	720.5431559
196.286	-196.286				
254.708	-254.708	+	254.731	0.0187613	721.0056593
		+	252.158	0.0187984	721.0241890
252.136	-252.136				
277.873	-277.873	+	277.919	0.015443	721.3627023
		+	371.758	0.00862069	721.4552988
370.867	-370.867				
251.342	-251.342	+	251.363	0.0188214	722.0080786
		+	271.815	0.0166506	722.0110093
271.776	-271.776				
220.347	-220.347	+	220.356	0.027731	722.0481468
		+	219.713	0.0277833	722.0506288
219.705	-219.705				
184.685	-184.685	+	184.688	0.0499509	722.2466237

		+	208.666	0.034043	723.0530288
20.866	-20.866				
204.561	-204.561	+	204.566	0.0361949	723.2379237
		+	209.118	0.0338265	723.3769367
209.112	-209.112				
208.538	-208.538	+	208.544	0.0340999	726.3435273
		+	259.825	0.0187134	726.4469514
259.798	-259.798				
212.779	-212.779	+	212.786	0.0310309	727.4956366
		+	217.541	0.0291762	727.9733185
217.534	-217.534				
291.609	-291.609	+	291.679	0.0150449	728.3350923
		+	300.397	0.0138073	730.5148764
300.304	-300.304				
25.106	-25.106	+	251.081	0.0189746	731.0452589
		+	265.022	0.0189285	733.2918069
26.499	-26.499				
217.124	-217.124	+	217.132	0.0294023	734.0488868
		+	216.391	0.0299391	734.0522479
216.383	-216.383				
214.741	-214.741	+	214.748	0.03098	734.3376604
		+	267.901	0.0178403	734.3428238
267.866	-267.866				
211.201	-211.201	+	211.207	0.0318307	735.3776947
		+	192.891	0.0426438	735.4144657
192.887	-192.887				
189.284	-189.284	+	189.287	0.0455201	735.5992175
		+	195.813	0.0408522	735.9724054
195.809	-195.809				
196.147	-196.147	+	196.151	0.0405061	736.4370173
		+	305.222	0.0132193	736.9792261
305.115	-305.115				
305.795	-305.795	+	305.904	0.0130909	736.9806665
		+	241.207	0.0216059	736.9974297
241.192	-241.192				
26.474	-26.474	+	264.771	0.0188657	737.3946766
		+	192.764	0.0427386	737.4274741

192.761	-192.761				
213.199	-213.199	+	213.206	0.0310742	737.4686068
		+	21.366	0.030995	737.4710486
213.653	-213.653				
257.572	-257.572	+	257.597	0.0185723	737.9826480
		+	188.681	0.0460747	738.0226585
188.678	-188.678				
209.477	-209.477	+	209.483	0.0335599	738.3763699
		+	263.324	0.019055	739.5290130
263.294	-263.294				
225.211	-225.211	+	22.522	0.0259562	739.5318488
		+	450.736	0	739.5526827
441.728	-441.728				
28.966	-28.966	+	289.726	0.0148954	740.0587018
		+	30.274	0.0130417	740.0617939
302.641	-302.641				
190.398	-190.398	+	190.401	0.0447975	740.3890230
		+	194.421	0.0416539	740.3907608
194.418	-194.418				
346.697	-346.697	+	347.091	0.00967619	741.0638106
		+	232.102	0.0235806	741.4655472
232.091	-232.091				
203.801	-203.801	+	203.806	0.0364613	742.3034776
		+	259.727	0.0186565	743.3447552
2.597	-2.597				
202.834	-202.834	+	202.839	0.0367116	743.5183768
		+	22.569	0.0258534	744.0517398
225.681	-225.681				
24.794	-24.794	+	247.959	0.020082	744.4940313
		+	40.447	0.00571429	744.5135148
401.727	-401.727				
214.494	-214.494	+	214.501	0.030925	744.5248634
		+	223.212	0.0263778	744.5788017
223.203	-223.203				
275.884	-275.884	+	275.928	0.0159369	745.4196316
		+	275.948	0.0159684	745.4205609
275.904	-275.904				
244.227	-244.227	+	244.244	0.0214312	746.4235543

		+	192.393	0.043204	747.0190115
192.389	-192.389				
19.285	-19.285	+	192.854	0.042793	747.0205246
		+	213.827	0.0310505	748.0522648
21.382	-21.382				
200.725	-200.725	+	200.729	0.0383351	748.5484093
		+	239.934	0.0220353	749.3928018
239.919	-239.919				
195.175	-195.175	+	195.178	0.0411707	750.4823433
		+	254.692	0.0187102	751.1059246
254.669	-254.669				
201.458	-201.458	+	201.462	0.0380052	751.4077178
		+	206.762	0.0348358	751.4856314
206.757	-206.757				
255.415	-255.415	+	255.439	0.0189667	752.4384834
22.793	-22.793	+	22.794	0.0252033	752.5077472
31.832	-31.832	+	31.848	0.0146939	753.4460704
		+	23.627	0.0232759	755.0732642
236.257	-236.257				
297.048	-297.048	+	297.131	0.0144596	755.2735314
		+	21.899	0.028089	756.0327163
218.982	-218.982				
23.762	-23.762	+	237.634	0.0226562	756.4781618
		+	211.035	0.0320145	756.4817467
211.029	-211.029				
270.742	-270.742	+	270.779	0.0165	756.4939036
		+	203.627	0.0363432	756.5297963
203.622	-203.622				
203.622	-203.622	+	203.627	0.0363236	756.5304098
		+	188.314	0.046409	757.1221722
188.311	-188.311				
238.776	-238.776	+	23.879	0.022292	758.5283728
		+	336.161	0.00898551	758.5468922
335.883	-335.883				
188.991	-188.991	+	188.994	0.0456687	759.3170567
		+	300.251	0.0137616	759.5506248

300.159	-300.159				
187.211	-187.211	+	187.214	0.0478111	760.0253455
		+	252.036	0.0187497	760.3959488
252.015	-252.015				
327.521	-327.521	+	327.735	0.012321	760.9975063
		+	19.675	0.0402678	761.0232810
196.746	-196.746				
220.101	-220.101	+	220.109	0.0276333	762.0390134
		+	222.779	0.0262772	762.0408320
22.277	-22.277				
190.461	-190.461	+	190.464	0.0447882	762.5019545
		+	189.521	0.0455409	763.0443735
189.518	-189.518				
193.947	-193.947	+	19.395	0.0418406	763.3744109
		+	205.874	0.0350943	763.5053668
205.869	-205.869				
260.186	-260.186	+	260.213	0.0189444	763.6319455
		+	227.763	0.025223	765.3254503
227.753	-227.753				
186.087	-186.087	+	18.609	0.0489358	766.0142822
		+	251.807	0.0187819	767.4020790
251.786	-251.786				
231.097	-231.097	+	231.108	0.0238024	768.4054815
		+	235.067	0.0234579	770.3461056
235.054	-235.054				
255.798	-255.798	+	255.822	0.0188499	770.5094535
		+	255.822	0.0188235	770.5101134
255.798	-255.798				
185.681	-185.681	+	185.684	0.0492872	771.0616941
		+	26.021	0.0189153	771.5129138
260.183	-260.183				
23.498	-23.498	+	234.993	0.0235788	772.5073209
		+	254.494	0.0186341	772.5161696
254.471	-254.471				
263.587	-263.587	+	263.617	0.0191792	773.5091899
		+	273.846	0.01662	773.5116943
273.805	-273.805				
198.256	-198.256	+	19.826	0.0397884	773.5285894

		+	197.436	0.0401262	773.5302916
197.432	-197.432				
196.547	-196.547	+	196.551	0.0403555	773.5489693
		+	193.901	0.0417619	773.5505879
193.897	-193.897				
188.747	-188.747	+	18.875	0.0459227	774.5128260
		+	235.504	0.0233522	774.5625857
235.491	-235.491				
200.473	-200.473	+	200.478	0.0385355	775.5665626
		+	318.026	0.0146193	777.2556299
317.868	-317.868				
269.757	-269.757	+	269.794	0.0165918	777.5854534
		+	199.111	0.0391235	778.0145158
199.107	-199.107				
219.037	-219.037	+	219.045	0.0281086	778.0991758
		+	219.577	0.0278456	778.1012988
219.569	-219.569				
194.614	-194.614	+	194.618	0.0416662	778.2972615
		+	186.682	0.0480625	778.4258004
186.679	-186.679				
285.155	-285.155	+	285.213	0.0141473	778.4758463
		+	193.447	0.0421876	779.0177884
193.444	-193.444				
228.681	-228.681	+	228.692	0.024602	780.4691903
		+	244.758	0.0217254	780.4711801
244.741	-244.741				
188.347	-188.347	+	188.351	0.0462926	781.4578387
		+	232.502	0.0234849	781.4744077
23.249	-23.249				
185.364	-185.364	+	185.367	0.0495178	782.4027782
		+	26.397	0.0192418	782.9806869
263.939	-263.939				
252.566	-252.566	+	252.588	0.0188399	783.3762203
		+	223.707	0.026426	784.0230389
223.698	-223.698				
225.121	-225.121	+	225.131	0.0258553	784.3783786
		+	223.937	0.0263404	784.3819840

223.928	-223.928				
188.571	-188.571	+	188.574	0.0461897	784.5252855
		+	249.309	0.0189376	785.0264387
249.289	-249.289				
198.002	-198.002	+	198.006	0.0397773	785.1217668
		+	194.457	0.0416736	785.5286366
194.453	-194.453				
194.147	-194.147	+	194.151	0.0417248	785.5303330
		+	281.373	0.0151965	787.4868309
281.322	-281.322				
201.731	-201.731	+	201.736	0.0378056	787.5635442
		+	313.687	0.0146729	787.7147190
313.549	-313.549				
196.084	-196.084	+	196.088	0.0405153	788.0685566
		+	19.311	0.0425632	789.3842926
193.107	-193.107				
269.601	-269.601	+	269.638	0.0167105	791.5989403
		+	268.325	0.0176154	791.6016304
26.829	-26.829				
209.283	-209.283	+	209.289	0.0337425	791.6635016
		+	380.801	0.00980392	792.3275961
379.585	-379.585				
201.576	-201.576	+	201.581	0.0377915	793.5319430
		+	236.785	0.0231884	794.4488396
236.771	-236.771				
241.655	-241.655	+	241.671	0.0218139	794.4521708
		+	243.791	0.0215268	796.7386591
243.775	-243.775				
290.152	-290.152	+	29.022	0.0146667	797.6353054
		+	219.519	0.0278571	798.5397941
219.511	-219.511				
219.511	-219.511	+	219.519	0.0278376	798.5408145
		+	260.192	0.0188571	798.9539452
260.165	-260.165				
221.555	-221.555	+	221.563	0.0270626	799.5441868
		+	209.471	0.0335401	799.9952254
209.465	-209.465				
223.816	-223.816	+	223.825	0.0264121	800.0822765

		+	213.813	0.0310113	800.3269824
213.806	-213.806				
212.584	-212.584	+	21.259	0.0310969	800.4836074
		+	204.731	0.0358222	800.5383734
204.726	-204.726				
216.334	-216.334	+	216.342	0.0299854	800.5480011
		+	23.039	0.0242075	801.0027306
230.379	-230.379				
21.707	-21.707	+	217.077	0.0293628	801.1164047
		+	185.981	0.049244	801.4870784
185.978	-185.978				
195.527	-195.527	+	195.531	0.0411792	801.5421058
		+	267.917	0.0178713	801.5543471
267.883	-267.883				
226.787	-226.787	+	226.797	0.0252195	802.5454603
		+	285.041	0.0141137	803.6266237
284.983	-284.983				
20.467	-20.467	+	204.675	0.0359474	804.6319859
		+	288.728	0.0146909	805.6428654
288.663	-288.663				
250.667	-250.667	+	250.687	0.0189095	805.6769291
		+	265.176	0.018709	806.0070283
265.144	-265.144				
225.391	-225.391	+	2.254	0.026099	807.5094313
		+	224.012	0.0264611	807.5111428
224.002	-224.002				
23.556	-23.556	+	235.573	0.0232734	808.3952694
		+	24.219	0.0218943	808.4861842
242.174	-242.174				
1.968	-1.968	+	196.804	0.0402603	808.4981407
		+	218.114	0.0287146	808.5031357
218.106	-218.106				
216.753	-216.753	+	21.676	0.0296662	809.3972497
		+	265.955	0.0186237	809.4691344
265.922	-265.922				
266.181	-266.181	+	266.214	0.0183265	809.4709816
		+	227.093	0.0250401	809.4887091

227.083	-227.083				
251.447	-251.447	+	251.468	0.0188696	809.4915043
		+	282.688	0.0150938	810.3958353
282.634	-282.634				
230.785	-230.785	+	230.796	0.0241785	810.4752648
		+	407.463	0.00588235	810.7893120
404.436	-404.436				
404.436	-404.436	+	407.463	0.00606061	810.7909201
		+	216.684	0.0297852	811.4845647
216.677	-216.677				
230.886	-230.886	+	230.898	0.0238967	811.7941952
		+	194.222	0.0417446	813.5023908
194.218	-194.218				
184.843	-184.843	+	184.846	0.0498021	814.0994794
		+	22.184	0.026962	815.1038165
221.831	-221.831				
327.767	-327.767	+	327.982	0.0125535	815.7461302
		+	221.971	0.0268689	816.5290409
221.963	-221.963				
233.231	-233.231	+	233.244	0.023658	816.5319977
		+	318.911	0.014555	816.7491335
318.749	-318.749				
318.749	-318.749	+	318.911	0.0144792	816.7504167
		+	225.185	0.0258956	817.6784605
225.175	-225.175				
225.929	-225.929	+	225.939	0.02575	817.6808344
		+	207.185	0.0345094	817.7525861
207.179	-207.179				
248.711	-248.711	+	24.873	0.0191873	819.2054425
		+	203.472	0.0362213	819.3754142
203.467	-203.467				
195.105	-195.105	+	195.109	0.0412027	819.6576412
		+	29.169	0.0150873	820.3948789
291.619	-291.619				
195.374	-195.374	+	195.378	0.0411368	821.4366080
		+	234.665	0.0234476	821.9811376
234.652	-234.652				
247.769	-247.769	+	247.788	0.0202019	822.4656647

		+	228.745	0.0246222	822.4788742
228.734	-228.734				
247.698	-247.698	+	247.717	0.0201777	822.4836473
		+	319.213	0.0147872	822.6433623
319.049	-319.049				
232.094	-232.094	+	232.105	0.0236014	822.9725570
		+	19.962	0.0388618	823.0982487
199.615	-199.615				
225.199	-225.199	+	225.209	0.0259158	823.4686912
		+	224.686	0.0261051	823.4708787
224.677	-224.677				
227.466	-227.466	+	227.476	0.0252223	823.4849924
		+	190.132	0.0448532	824.3734473
190.128	-190.128				
2.651	-2.651	+	265.132	0.0186778	824.7694327
		+	265.132	0.0186467	824.7705093
2.651	-2.651				
256.155	-256.155	+	256.179	0.018744	825.7734674
		+	213.066	0.0311213	826.4989035
213.059	-213.059				
212.173	-212.173	+	212.179	0.0312741	826.5004771
		+	21.977	0.0278814	827.5027550
219.762	-219.762				
220.091	-220.091	+	220.099	0.0276136	829.7047741
		+	230.957	0.0238723	830.3766502
230.946	-230.946				
234.276	-234.276	+	234.289	0.0235945	830.4432997
		+	18.552	0.0494336	830.5291226
185.518	-185.518				
185.631	-185.631	+	185.634	0.0493505	830.5309734
		+	253.431	0.0186915	830.7069567
253.408	-253.408				
210.514	-210.514	+	21.052	0.0329068	830.7287556
		+	210.403	0.0329281	830.7303846
210.396	-210.396				
200.337	-200.337	+	200.341	0.0386365	831.5342096
		+	269.719	0.0167402	831.7191639

269.682	-269.682				
26.876	-26.876	+	268.796	0.0170756	831.7207260
		+	288.868	0.0145445	832.1095225
288.803	-288.803				
285.357	-285.357	+	285.415	0.0144213	832.1117527
		+	286.613	0.014531	832.3826958
286.552	-286.552				
190.198	-190.198	+	190.202	0.0449084	832.4533711
		+	239.822	0.0221554	832.7234776
239.807	-239.807				
213.959	-213.959	+	213.965	0.0311488	833.1143926
		+	261.791	0.0191987	833.6746265
261.763	-261.763				
316.171	-316.171	+	316.321	0.0141176	834.5875480
		+	297.287	0.0145956	835.4867623
297.203	-297.203				
212.579	-212.579	+	212.586	0.0310777	836.2770483
		+	220.924	0.0272698	837.5024450
220.916	-220.916				
235.527	-235.527	+	23.554	0.0233019	838.8219333
		+	209.314	0.0337822	838.9456240
209.308	-209.308				
189.189	-189.189	+	189.192	0.0455916	839.4641003
		+	233.079	0.0235731	840.0744803
233.067	-233.067				
212.355	-212.355	+	212.362	0.0312895	843.7774545
		+	191.099	0.044298	844.4223519
191.095	-191.095				
225.453	-225.453	+	225.463	0.0260409	844.7794907
		+	225.463	0.0260614	844.7809346
225.453	-225.453				
238.589	-238.589	+	238.603	0.0222806	845.4141330
		+	362.968	0.00944	845.6989722
362.306	-362.306				
363.135	-363.135	+	363.816	0.00969863	845.7010555
		+	195.096	0.0411633	846.1122589
195.092	-195.092				
288.384	-288.384	+	288.448	0.0145773	846.5561508

		+	276.142	0.0157285	846.7025614
276.098	-276.098				
189.555	-189.555	+	189.558	0.0454465	848.4791487
		+	189.296	0.0455401	848.4813714
189.292	-189.292				
225.842	-225.842	+	225.852	0.0259352	848.6583099
		+	236.281	0.0232982	848.6609403
236.267	-236.267				
191.382	-191.382	+	191.386	0.0439188	849.4674131
		+	229.938	0.0244882	850.0427355
229.927	-229.927				
319.611	-319.611	+	319.778	0.0152747	850.5014763
		+	234.544	0.023475	854.0931214
234.531	-234.531				
20.029	-20.029	+	200.294	0.038577	855.6622812
		+	259.919	0.0187706	857.0216972
259.892	-259.892				
191.823	-191.823	+	191.827	0.0437385	857.6351552
		+	204.765	0.035732	858.0242270
20.476	-20.476				
263.425	-263.425	+	263.455	0.0190859	858.7386898
		+	192.803	0.0427978	859.6891904
192.799	-192.799				
192.663	-192.663	+	192.667	0.042795	859.6904796
		+	246.067	0.0203501	859.7514987
246.049	-246.049				
235.776	-235.776	+	23.579	0.0230989	860.7550511
		+	412.463	0.00666667	861.4882594
40.891	-40.891				
490.291	-490.291	+	499.453	0	861.4910567
		+	204.659	0.0359825	862.0860782
204.654	-204.654				
237.674	-237.674	+	237.688	0.0226785	864.4551413
		+	281.466	0.0152301	864.6321979
281.414	-281.414				
190.985	-190.985	+	190.989	0.044292	866.6480334
		+	248.039	0.02	866.8168031

24.802	-24.802				
216.587	-216.587	+	216.595	0.0299254	868.6637319
		+	263.616	0.019148	869.3064351
263.586	-263.586				
198.297	-198.297	+	198.302	0.0398481	871.6144358
		+	25.476	0.0187869	872.6172848
254.737	-254.737				
207.957	-207.957	+	207.963	0.034376	873.5149763
		+	3.366	0.00911765	873.7269870
336.318	-336.318				
306.246	-306.246	+	306.356	0.0128958	873.7310153
		+	24.734	0.020315	874.5435620
247.321	-247.321				
189.981	-189.981	+	189.985	0.0449133	874.7340561
		+	196.202	0.0404968	875.5041973
196.198	-196.198				
220.682	-220.682	+	220.691	0.0273482	876.0747487
		+	185.938	0.0492234	876.5329835
185.935	-185.935				
203.378	-203.378	+	203.383	0.0363217	877.0769823
		+	186.261	0.0487328	879.5688924
186.258	-186.258				
220.829	-220.829	+	220.837	0.027296	881.4357137
		+	190.009	0.0449531	881.5365707
190.005	-190.005				
197.799	-197.799	+	197.804	0.0398572	884.6343480
		+	194.687	0.0417059	885.4262263
194.683	-194.683				
253.341	-253.341	+	253.363	0.0186667	887.4236408
		+	207.313	0.0343434	889.7826871
207.308	-207.308				
241.816	-241.816	+	241.832	0.0218373	890.4681900
		+	250.883	0.0189027	890.4722210
250.863	-250.863				
208.758	-208.758	+	208.764	0.0340641	891.4740023
		+	217.171	0.0294418	892.0482389
217.164	-217.164				
237.388	-237.388	+	237.402	0.0227463	892.4671614

		+	277.956	0.0155085	892.4720425
27.791	-27.791				
222.653	-222.653	+	222.662	0.026432	892.5062181
		+	206.488	0.0348868	893.5347246
206.482	-206.482				
191.267	-191.267	+	191.271	0.0439928	894.5033257
		+	424.964	0.00952381	895.4957276
419.827	-419.827				
231.075	-231.075	+	231.086	0.0237612	896.4982200
		+	231.491	0.023725	896.5015173
231.479	-231.479				
194.485	-194.485	+	194.489	0.0416741	896.5174799
		+	293.511	0.0149449	897.0138459
293.437	-293.437				
209.119	-209.119	+	209.125	0.0338463	897.4865925
		+	211.929	0.0316259	898.0162891
211.923	-211.923				
198.283	-198.283	+	198.288	0.0398282	898.4890717
		+	275.867	0.0159055	899.0577424
275.823	-275.823				
233.197	-233.197	+	233.209	0.0236367	899.0629529
		+	200.709	0.0383649	900.6488657
200.704	-200.704				
198.991	-198.991	+	198.995	0.0392356	900.6507289
		+	19.381	0.0418733	902.4352065
193.807	-193.807				
219.733	-219.733	+	219.741	0.027842	903.4167261
		+	238.789	0.0222696	903.5352016
238.775	-238.775				
224.195	-224.195	+	224.204	0.0263969	904.4667731
		+	216.555	0.0299721	904.4729325
216.548	-216.548				
191.857	-191.857	+	191.861	0.0437584	906.4842064
		+	272.632	0.0166505	906.5238226
272.592	-272.592				
187.494	-187.494	+	187.497	0.0472895	908.4766618
		+	247.026	0.0201706	911.4433627

247.007	-247.007				
370.948	-370.948	+	371.841	0.00877193	912.4747946
		+	268.061	0.0179337	912.9877693
268.027	-268.027				
217.334	-217.334	+	217.342	0.0292407	913.4587408
		+	218.695	0.028329	913.4611149
218.687	-218.687				
20.639	-20.639	+	206.395	0.0349377	913.4771829
		+	184.899	0.0498156	913.4830865
184.896	-184.896				
200.552	-200.552	+	200.556	0.0384206	914.0310899
		+	19.094	0.0443169	914.4660584
190.937	-190.937				
301.401	-301.401	+	301.496	0.0137027	914.4881130
		+	313.079	0.0142727	914.4921122
312.943	-312.943				
200.284	-200.284	+	200.289	0.0386085	915.4555912
		+	19.332	0.0423291	916.4746536
193.316	-193.316				
298.892	-298.892	+	29.898	0.0142821	918.4862641
		+	203.465	0.0362018	918.4991055
20.346	-20.346				
204.023	-204.023	+	204.028	0.0364473	918.5009170
		+	238.441	0.0223244	919.4883132
238.427	-238.427				
202.841	-202.841	+	202.846	0.0367311	919.4919272
		+	184.765	0.0498107	920.0416500
184.763	-184.763				
219.568	-219.568	+	219.577	0.0278261	920.4986310
		+	224.486	0.0264215	920.5021194
224.476	-224.476				
198.977	-198.977	+	198.982	0.039196	920.5377096
		+	203.592	0.0362845	921.0441838
203.587	-203.587				
202.998	-202.998	+	203.003	0.0368171	921.5057767
		+	219.311	0.0278297	921.5435015
219.302	-219.302				
20.023	-20.023	+	200.235	0.0386441	934.4563934

		+	190.676	0.0445505	934.5553018
190.673	-190.673				
210.386	-210.386	+	210.393	0.0329083	936.4978147
		+	211.053	0.0320338	936.5009366
211.047	-211.047				
201.455	-201.455	+	20.146	0.0379854	936.5225225
		+	253.335	0.0186419	936.9852080
253.312	-253.312				
244.299	-244.299	+	244.316	0.021504	937.4979531
		+	265.692	0.0187297	937.5026399
26.566	-26.566				
232.699	-232.699	+	232.711	0.0233843	938.0482176
		+	249.394	0.018984	938.0510706
249.374	-249.374				
208.151	-208.151	+	208.157	0.034215	939.0533028
		+	199.081	0.0391037	939.4959315
199.077	-199.077				
327.291	-327.291	+	327.503	0.0122454	940.5062592
		+	261.822	0.0192293	941.4923186
261.793	-261.793				
270.783	-270.783	+	27.082	0.0165299	941.5084686
		+	239.404	0.0221333	941.5122404
239.389	-239.389				
211.925	-211.925	+	211.931	0.0316453	943.4126478
		+	212.223	0.0312933	943.4673131
212.216	-212.216				
432.131	-432.131	+	43.944	0.00625	943.4726481
		+	189.017	0.045645	944.4969737
189.014	-189.014				
223.759	-223.759	+	223.768	0.026446	945.5088918
		+	224.024	0.0263927	945.5107285
224.015	-224.015				
226.336	-226.336	+	226.346	0.0256165	946.4659746
		+	225.997	0.0257704	946.5182387
225.988	-225.988				
225.105	-225.105	+	225.114	0.0258351	946.5202709
		+	210.323	0.0329689	947.5194656

210.317	-210.317				
210.278	-210.278	+	210.285	0.033009	947.5216688
		+	21.717	0.029422	948.4747480
217.162	-217.162				
299.501	-299.501	+	29.959	0.0144207	948.5138619
		+	242.701	0.0218775	948.5250185
242.685	-242.685				
236.322	-236.322	+	236.336	0.0233205	949.4770528
		+	196.506	0.0403762	949.4832814
196.502	-196.502				
223.034	-223.034	+	223.043	0.0263186	950.1165353
		+	186.847	0.0480152	952.5362299
186.844	-186.844				
199.111	-199.111	+	199.115	0.0391433	953.5477267
		+	213.474	0.0309799	954.0231856
213.468	-213.468				
191.044	-191.044	+	191.048	0.0443317	960.0320472
		+	212.647	0.0310543	960.5477036
212.641	-212.641				
194.664	-194.664	+	194.668	0.041686	961.4837645
		+	234.956	0.0235568	961.5542668
234.943	-234.943				
278.265	-278.265	+	278.313	0.0153362	962.4885083
		+	282.135	0.0152182	962.4918908
282.082	-282.082				
186.341	-186.341	+	186.344	0.0486669	963.4953071
		+	18.866	0.046098	963.6551912
188.656	-188.656				
217.496	-217.496	+	217.503	0.0291564	964.0771407
		+	247.936	0.0200578	964.5479717
247.917	-247.917				
233.979	-233.979	+	233.991	0.0236636	968.1269367
		+	365.227	0.00881159	970.4565038
364.513	-364.513				
190.402	-190.402	+	190.406	0.0448175	970.4957268
		+	192.049	0.0436843	970.6531452
192.045	-192.045				
212.156	-212.156	+	212.163	0.0313235	974.5493862

		+	212.152	0.0313043	974.5503725
212.146	-212.146				
200.508	-200.508	+	200.512	0.0385554	975.5530323
		+	191.575	0.0438173	977.5484921
191.571	-191.571				
242.218	-242.218	+	242.234	0.0219416	977.5644670
		+	25.508	0.0189683	978.6552446
255.057	-255.057				
225.316	-225.316	+	225.326	0.0260376	979.6582426
		+	203.066	0.0368564	981.6487923
203.061	-203.061				
196.821	-196.821	+	196.825	0.0402507	981.6514065
		+	237.414	0.0227686	982.6568406
2.374	-2.374				
211.351	-211.351	+	211.357	0.0317597	985.4769951
		+	192.096	0.0435872	986.0586470
192.092	-192.092				
192.913	-192.913	+	192.916	0.0426173	986.0612088
		+	186.611	0.0484272	988.5062645
186.608	-186.608				
2.871	-2.871	+	287.161	0.0144261	988.5961419
		+	238.166	0.0223676	988.6015378
238.152	-238.152				
245.477	-245.477	+	245.495	0.02108	989.5140761
		+	224.829	0.0260108	989.6047454
22.482	-22.482				
193.898	-193.898	+	193.902	0.0417815	990.0348271
		+	267.624	0.0179827	990.5184050
26.759	-26.759				
276.262	-276.262	+	276.307	0.0158233	990.5230821
		+	258.244	0.0181866	991.5266530
258.218	-258.218				
258.639	-258.639	+	258.665	0.0181733	992.6340881
		+	300.225	0.0137162	993.6375089
300.134	-300.134				
191.073	-191.073	+	191.076	0.0443516	998.6842776
		+	238.508	0.0222692	1000.6370110

238.493	-238.493				
201.281	-201.281	+	201.286	0.0381312	1001.6119780
		+	227.433	0.0251816	1001.6373500
227.422	-227.422				
214.056	-214.056	+	214.063	0.0312478	1001.6411190
		+	196.912	0.0402705	1008.6066110
196.908	-196.908				
21.373	-21.373	+	213.737	0.0310535	1009.6783220
		+	207.324	0.0343128	1009.6822370
207.319	-207.319				
211.479	-211.479	+	211.485	0.0315553	1011.2784840
		+	203.316	0.0365681	1011.2810840
203.311	-203.311				
186.085	-186.085	+	186.088	0.0489153	1012.5049720
		+	23.994	0.0220582	1014.6164420
239.925	-239.925				
241.827	-241.827	+	241.843	0.0218607	1015.6189280
		+	243.161	0.0220332	1015.6207970
243.144	-243.144				
231.826	-231.826	+	231.838	0.023716	1020.6657950
		+	228.863	0.0247036	1021.6687120
228.853	-228.853				
229.604	-229.604	+	229.615	0.0246577	1021.6707160
		+	204.425	0.0362728	1024.5063960
20.442	-20.442				
215.545	-215.545	+	215.552	0.0304993	1028.5247920
		+	210.139	0.0330656	1028.6325560
210.133	-210.133				
194.087	-194.087	+	19.409	0.0417404	1028.6684280
		+	192.946	0.0425907	1028.6706140
192.942	-192.942				
187.449	-187.449	+	187.452	0.0473345	1036.0841940
		+	189.774	0.0452357	1036.6377720
189.771	-189.771				
187.113	-187.113	+	187.116	0.0477706	1036.6419690
		+	185.886	0.0492659	1038.5032650
185.883	-185.883				
195.301	-195.301	+	195.305	0.0411735	1042.6476930

		+	193.608	0.04216	1043.6493510
193.604	-193.604				
193.093	-193.093	+	193.097	0.0425434	1043.6512490
		+	200.225	0.0386243	1046.4872110
200.221	-200.221				
239.028	-239.028	+	239.042	0.0223716	1048.4877250
		+	225.253	0.0259765	1048.4919090
225.244	-225.244				
192.465	-192.465	+	192.469	0.0430785	1050.5266710
		+	200.181	0.0386557	1052.5039600
200.176	-200.176				
32.131	-32.131	+	321.486	0.0150909	1054.5381720
		+	364.063	0.00983333	1054.5412860
363.376	-363.376				
277.588	-277.588	+	277.635	0.0154583	1055.5442240
		+	205.756	0.0351274	1056.6049900
205.751	-205.751				
202.907	-202.907	+	202.912	0.036829	1064.5033780
		+	196.082	0.0404956	1066.4738970
196.078	-196.078				
197.283	-197.283	+	197.287	0.0402028	1068.5077160
		+	205.944	0.0350218	1068.5129540
205.939	-205.939				
208.661	-208.661	+	208.667	0.0340629	1070.5731120
		+	235.969	0.0231208	1074.0623280
235.956	-235.956				
231.227	-231.227	+	231.239	0.0238645	1074.5168760
		+	191.687	0.0437244	1084.5538100
191.683	-191.683				
21.124	-21.124	+	211.246	0.0318087	1090.0349620
		+	210.151	0.033105	1092.4872110
210.145	-210.145				
309.048	-309.048	+	309.168	0.0133333	1093.4959560
		+	188.765	0.0459192	1094.4938930
188.762	-188.762				
187.295	-187.295	+	187.299	0.0476182	1094.5045470
		+	255.913	0.0187528	1096.5057390

255.889	-255.889				
24.703	-24.703	+	247.049	0.0201945	1098.5033630
		+	196.526	0.0402964	1111.5244560
196.522	-196.522				
25.165	-25.165	+	251.671	0.018838	1112.5850650
		+	200.668	0.0383055	1117.5154700
200.663	-200.663				
206.557	-206.557	+	206.563	0.0348133	1121.5147790
		+	188.166	0.0465656	1122.5189310
188.163	-188.163				
189.361	-189.361	+	189.364	0.0455362	1122.5224240
		+	201.854	0.0376362	1123.5263490
201.849	-201.849				
185.872	-185.872	+	185.875	0.0492854	1123.5313420
		+	195.109	0.041183	1124.5158990
195.105	-195.105				
233.392	-233.392	+	233.404	0.0236745	1124.5250710
		+	223.927	0.0263005	1126.5346660
223.918	-223.918				
206.425	-206.425	+	206.431	0.0349207	1127.5375650
		+	204.129	0.0362978	1133.5155250
204.124	-204.124				
207.898	-207.898	+	207.904	0.0344138	1137.5359830
		+	233.094	0.0235943	1138.4952590
233.082	-233.082				
195.951	-195.951	+	195.955	0.0406844	1139.5449460
		+	239.184	0.0221184	1152.5470440
23.917	-23.917				
187.315	-187.315	+	187.318	0.0476162	1152.5570090
		+	225.381	0.0260785	1153.5565300
225.371	-225.371				
211.893	-211.893	+	211.899	0.031568	1156.6777550
		+	190.264	0.0449484	1165.5220930
190.261	-190.261				
223.347	-223.347	+	223.356	0.0264373	1166.5265220
		+	186.258	0.0487543	1167.5267370
186.255	-186.255				
197.328	-197.328	+	197.332	0.0402424	1190.6173430

222.811	-222.811	+	222.819	0.0263163	1190.6220070

Supplementary Table S4: Posthoc test HCT-8 for control cells in negative-ion mode

Control	Infected	C: ANOVA Significant	N: -Log ANOVA p value	N: ANOVA q-value	MALDI m/z
		+	436.984	0	251.0711348
430.056	-430.056				
19.899	-19.899	+	198.994	0.0306104	252.0781412
		+	291.278	0.00509953	254.0939836
291.208	-291.208				
18.981	-18.981	+	189.813	0.0362276	255.0779119
219.737	-219.737	+	219.746	0.0216456	255.0891914
		+	242.396	0.0146277	259.0838539
24.238	-24.238				
408.012	-408.012	+	411.454	0.000263473	264.0657606
		+	360.042	0.00114513	266.0821888
359.441	-359.441				
399.068	-399.068	+	401.556	0.000243094	267.0745465
		+	245.909	0.0137426	267.0893010
245.891	-245.891				
172.718	-172.718	+	17.272	0.0489608	269.0765454
		+	282.906	0.00625082	269.0813289
282.851	-282.851				
277.469	-277.469	+	277.515	0.00722269	269.1259575
		+	257.384	0.0109937	270.0892929
257.359	-257.359				
216.828	-216.828	+	216.835	0.0225681	271.0846781
		+	315.539	0.00348498	271.0927193
315.393	-315.393				
297.749	-297.749	+	297.834	0.00406991	274.0294954
		+	22.001	0.0216036	276.0457368
220.002	-220.002				
179.792	-179.792	+	179.794	0.0434913	277.0729850
		+	251.408	0.0125239	279.0885740

251.387	-251.387				
252.088	-252.088	+	25.211	0.01221	281.1042973
		+	171.943	0.0497555	282.0748182
171.941	-171.941				
179.707	-179.707	+	17.971	0.0434729	283.0837997
		+	213.566	0.0248048	283.1412661
213.559	-213.559				
194.987	-194.987	+	194.991	0.0330946	285.0724231
		+	288.795	0.00551661	286.0835517
288.731	-288.731				
22.375	-22.375	+	22.376	0.0200894	286.0987653
		+	174.006	0.0477612	288.1144680
174.004	-174.004				
276.679	-276.679	+	276.724	0.00724662	290.0241689
		+	184.299	0.0397869	290.0936739
184.296	-184.296				
174.254	-174.254	+	174.256	0.0476408	291.0888933
		+	337.916	0.00181695	292.0389954
337.622	-337.622				
187.463	-187.463	+	187.466	0.03754	292.1093044
175.752	-175.752	+	175.754	0.0465898	295.0513312
		+	238.435	0.0159068	297.0469841
238.421	-238.421				
245.296	-245.296	+	245.313	0.013811	297.0990920
		+	22.034	0.0213647	299.0938245
220.332	-220.332				
338.331	-338.331	+	338.631	0.00182624	300.1019146
		+	190.338	0.0358985	301.0853977
190.334	-190.334				
183.775	-183.775	+	183.777	0.0401816	301.1093295
		+	194.037	0.0334928	302.0934160
194.033	-194.033				
186.817	-186.817	+	18.682	0.0378479	303.0887326
		+	182.185	0.041276	303.0967571
182.183	-182.183				
190.916	-190.916	+	19.092	0.0357476	303.1012710
		+	215.914	0.0233914	303.1249812

215.907	-215.907				
22.142	-22.142	+	221.428	0.021065	304.1090099
		+	246.249	0.0137317	305.1044159
246.231	-246.231				
196.807	-196.807	+	196.811	0.0319246	305.1137019
		+	41.408	0.000266667	306.0190330
410.342	-410.342				
255.236	-255.236	+	25.526	0.0117692	306.0756567
		+	308.346	0.00366667	306.0989187
308.229	-308.229				
334.114	-334.114	+	334.377	0.00191104	307.0928905
		+	509.406	0	308.0346188
502.966	-502.966				
174.855	-174.855	+	174.858	0.0471413	308.1040290
		+	197.148	0.031781	309.0187150
197.144	-197.144				
236.041	-236.041	+	236.054	0.0165228	311.0240252
		+	26.889	0.00801957	311.1150168
268.855	-268.855				
184.146	-184.146	+	184.149	0.0398936	312.0740581
		+	504.956	0	312.1024367
497.145	-497.145				
214.853	-214.853	+	21.486	0.0240024	313.0731946
		+	176.528	0.0461744	313.0949178
176.526	-176.526				
376.223	-376.223	+	3.773	0.00100893	314.0444360
		+	194.048	0.0335095	314.0777292
194.044	-194.044				
362.758	-362.758	+	36.343	0.00117551	314.0814267
		+	234.684	0.0169038	314.1046562
234.671	-234.671				
25.429	-25.429	+	254.313	0.0117843	314.1167380
		+	20.651	0.0275835	315.0747760
206.504	-206.504				
180.734	-180.734	+	180.737	0.0429623	316.0839375
		+	181.251	0.0422726	316.1087760
181.248	-181.248				
216.914	-216.914	+	216.922	0.0225959	316.1195940

		+	216.922	0.022582	316.1203822
216.914	-216.914				
177.971	-177.971	+	177.973	0.0448367	317.0764233
		+	175.484	0.0467882	317.0805312
175.482	-175.482				
198.131	-198.131	+	198.136	0.031109	318.0994446
		+	177.625	0.0450809	318.1073689
177.623	-177.623				
2.429	-2.429	+	242.917	0.0146275	319.1201279
		+	230.244	0.017618	320.1151988
230.233	-230.233				
387.614	-387.614	+	389.244	0.000650246	321.0296025
		+	297.708	0.00405473	322.0138030
297.623	-297.623				
210.569	-210.569	+	210.576	0.0262714	322.0941317
		+	198.955	0.030759	322.1037055
19.895	-19.895				
370.345	-370.345	+	37.122	0.00122034	323.0274996
		+	37.419	0.00125217	323.0443878
373.222	-373.222				
173.763	-173.763	+	173.765	0.0478076	323.1148074
		+	375.759	0.000993407	324.0294916
374.737	-374.737				
212.908	-212.908	+	212.914	0.0249899	324.1090240
38.588	-38.588	+	38.741	0.000637681	325.0617515
		+	289.977	0.0051729	326.0649157
28.991	-28.991				
238.335	-238.335	+	23.835	0.0158592	326.0889376
		+	281.467	0.00651996	327.1096364
281.415	-281.415				
285.268	-285.268	+	285.327	0.00594438	327.1109040
203.948	-203.948	+	203.953	0.0286688	328.0583365
		+	189.831	0.036262	328.0838594
189.828	-189.828				
337.032	-337.032	+	33.732	0.00194631	329.0922171
		+	470.255	0	330.1127666

459.185	-459.185				
220.817	-220.817	+	220.825	0.0212233	331.0954343
		+	309.516	0.00371156	331.1204323
309.395	-309.395				
25.713	-25.713	+	257.155	0.0111464	332.1039922
		+	391.115	0.000666667	333.0586306
389.375	-389.375				
225.977	-225.977	+	225.987	0.0195849	333.1181174
		+	266.011	0.00862813	334.1308989
265.978	-265.978				
380.647	-380.647	+	381.911	0.000611111	337.0247982
		+	186.336	0.038068	337.1306724
186.333	-186.333				
268.173	-268.173	+	268.208	0.00823851	338.0087533
		+	186.161	0.038199	338.1023213
186.158	-186.158				
292.101	-292.101	+	292.172	0.00503103	339.0395194
		+	193.881	0.033501	339.1095938
193.877	-193.877				
374.822	-374.822	+	375.847	0.000995595	339.1128001
		+	179.596	0.0437697	340.0808131
179.594	-179.594				
472.746	-472.746	+	483.897	0	340.0961501
		+	224.208	0.0201428	340.1049224
224.199	-224.199				
182.305	-182.305	+	182.308	0.04117	340.1639426
		+	470.783	0	341.0552624
459.684	-459.684				
430.751	-430.751	+	437.807	0	341.0924332
		+	386.128	0.000627078	341.1045551
384.665	-384.665				
452.267	-452.267	+	46.275	0	341.1282212
		+	333.101	0.0020459	342.0585652
332.849	-332.849				
178.847	-178.847	+	17.885	0.0441853	342.0747035
		+	301.871	0.00409819	342.0974019

301.775	-301.775				
445.105	-445.105	+	454.647	0	342.1127478
		+	367.918	0.00120755	343.0713843
367.136	-367.136				
426.359	-426.359	+	432.618	0	343.1078485
		+	465.972	0	343.1165401
455.199	-455.199				
208.849	-208.849	+	208.855	0.0264542	343.1207937
		+	192.267	0.0348784	344.0916107
192.263	-192.263				
190.616	-190.616	+	190.619	0.0358139	344.1037209
28.416	-28.416	+	284.217	0.00622271	344.1144902
		+	463.969	0	344.1283667
45.337	-45.337				
194.643	-194.643	+	194.647	0.0332343	345.1105219
419.896	-419.896	+	425.044	0	346.0560170
		+	28.127	0.00651293	346.1067842
281.219	-281.219				
200.003	-200.003	+	200.008	0.0302476	346.1310348
		+	285.452	0.00595764	347.0392851
285.393	-285.393				
227.802	-227.802	+	227.813	0.0184569	347.1146895
		+	263.091	0.00966509	347.1259422
263.062	-263.062				
203.095	-203.095	+	2.031	0.0292654	348.0985502
		+	248.732	0.0131657	348.1101056
248.712	-248.712				
224.884	-224.884	+	224.894	0.0200534	349.1286653
		+	229.944	0.0178111	349.1306285
229.933	-229.933				
469.878	-469.878	+	481.134	0	350.0449622
		+	261.784	0.0100915	350.1155870
261.755	-261.755				
400.992	-400.992	+	403.662	0.000248588	353.0195661
		+	456.093	0	353.0488951
446.365	-446.365				
193.457	-193.457	+	193.461	0.0339703	353.1160287

		+	20.008	0.0302635	353.1253924
200.076	-200.076				
356.225	-356.225	+	356.764	0.00127938	354.0231463
		+	313.729	0.00358815	354.1779796
313.591	-313.591				
229.943	-229.943	+	229.954	0.0178235	355.0859417
		+	500.861	0	356.0928383
492.009	-492.009				
378.441	-378.441	+	379.608	0.000829157	356.1151691
		+	465.631	0	357.0872371
454.886	-454.886				
385.435	-385.435	+	38.694	0.000633094	357.0994798
		+	38.638	0.000630072	357.1006196
384.904	-384.904				
361.297	-361.297	+	361.937	0.00116364	357.1237710
		+	403.699	0.000249292	358.1078159
401.025	-401.025				
429.231	-429.231	+	436.009	0	358.1269702
		+	457.857	0	359.1031003
447.911	-447.911				
283.302	-283.302	+	283.357	0.00626448	359.1139836
		+	293.585	0.00497949	360.1081065
29.351	-29.351				
523.528	-523.528	+	523.698	0	360.1234392
		+	217.805	0.0220422	361.1309360
217.797	-217.797				
32.927	-32.927	+	329.496	0.00243981	362.1255309
		+	284.755	0.00615282	363.1209562
284.697	-284.697				
298.357	-298.357	+	298.444	0.00410063	365.1247101
17.858	-17.858	+	178.582	0.044335	366.1202861
		+	184.263	0.0398213	366.1775406
18.426	-18.426				
214.871	-214.871	+	214.878	0.0240315	367.1235888
249.361	-249.361	+	24.938	0.0129803	368.1360050
		+	31.269	0.00366574	370.0927172

312.556	-312.556				
313.366	-313.366	+	313.504	0.00357303	370.1173109
		+	390.642	0.000656716	371.0658919
388.931	-388.931				
30.405	-30.405	+	304.153	0.00415727	371.0844002
		+	468.606	0	371.1028573
457.637	-457.637				
351.569	-351.569	+	352.031	0.00163465	372.0861791
		+	466.367	0	372.1071298
455.562	-455.562				
295.568	-295.568	+	295.648	0.00451724	372.1105403
		+	228.895	0.0184554	373.0819212
228.885	-228.885				
196.914	-196.914	+	196.918	0.0320061	373.0947490
		+	407.024	0.000251429	373.1185564
40.404	-40.404				
291.479	-291.479	+	29.155	0.00510558	374.1021346
		+	259.405	0.0107156	374.1249919
259.379	-259.379				
196.626	-196.626	+	19.663	0.0319044	375.1096843
		+	197.348	0.0317026	375.1102688
197.343	-197.343				
224.148	-224.148	+	224.157	0.0201295	375.1223515
		+	185.988	0.0382198	376.1151041
185.985	-185.985				
188.504	-188.504	+	188.507	0.0369232	376.1418440
		+	272.364	0.0074509	377.1252604
272.325	-272.325				
245.743	-245.743	+	24.576	0.0137097	378.1196379
		+	269.451	0.00803529	381.1192209
269.415	-269.415				
313.362	-313.362	+	3.135	0.00356802	381.1214101
		+	181.231	0.0422541	381.1316434
181.229	-181.229				
210.195	-210.195	+	210.201	0.0264491	382.2087920
		+	390.956	0.00066	384.0866325

389.226	-389.226				
23.414	-23.414	+	234.153	0.0170218	384.1107791
		+	221.282	0.0211825	384.1323511
221.273	-221.273				
309.483	-309.483	+	309.605	0.00372169	385.0821438
		+	530.188	0	386.1137595
533.904	-533.904				
209.145	-209.145	+	20.915	0.0263639	386.1256092
		+	395.276	0.000453608	387.0977997
393.267	-393.267				
39.429	-39.429	+	396.376	0.00045953	389.1133448
		+	241.929	0.0146881	390.1207889
241.914	-241.914				
337.951	-337.951	+	338.247	0.00182003	390.1326703
		+	174.161	0.0477329	391.1045511
174.159	-174.159				
187.854	-187.854	+	187.857	0.0373637	391.1160627
		+	274.921	0.00743091	392.1362076
274.879	-274.879				
228.122	-228.122	+	228.132	0.018466	393.1196945
		+	223.423	0.0202413	393.1207077
223.414	-223.414				
29.548	-29.548	+	295.559	0.00465437	394.1150034
		+	215.065	0.0240365	395.0451602
215.058	-215.058				
175.182	-175.182	+	175.184	0.0469864	396.2248820
		+	294.496	0.00482725	397.0627749
294.419	-294.419				
221.445	-221.445	+	221.454	0.0209601	397.1163473
		+	30.416	0.00416273	397.1266658
304.057	-304.057				
184.531	-184.531	+	184.534	0.0396271	398.0449270
		+	230.199	0.0176056	399.0749918
230.188	-230.188				
379.464	-379.464	+	380.675	0.000605505	400.0932425
		+	197.627	0.0314468	401.0761891
197.623	-197.623				
234.253	-234.253	+	234.266	0.0170465	403.0466470

		+	233.914	0.0170875	403.0922330
233.901	-233.901				
216.579	-216.579	+	216.586	0.0228585	404.0140811
		+	243.937	0.0141997	404.1158540
24.392	-24.392				
214.564	-214.564	+	214.571	0.0241716	404.1361927
		+	401.276	0.000240437	405.1226721
398.811	-398.811				
33.631	-33.631	+	336.592	0.0019398	405.1322352
		+	287.409	0.00566516	406.1155407
287.347	-287.347				
180.739	-180.739	+	180.741	0.0429811	406.1266124
		+	224.048	0.0201029	406.1312867
224.039	-224.039				
207.108	-207.108	+	207.114	0.027131	407.0987439
		+	195.518	0.0325045	407.1371308
195.514	-195.514				
282.643	-282.643	+	282.697	0.00623043	408.1313426
		+	217.262	0.0224005	408.1461171
217.254	-217.254				
298.866	-298.866	+	298.954	0.00400505	410.0562072
		+	239.942	0.0152424	410.1251296
239.927	-239.927				
317.987	-317.987	+	318.146	0.00320118	412.0634589
		+	239.538	0.0152194	413.0556607
239.523	-239.523				
386.071	-386.071	+	387.611	0.000639225	413.1112429
		+	301.438	0.00407189	420.1086115
301.343	-301.343				
327.947	-327.947	+	328.164	0.00256869	420.1112172
		+	183.789	0.0401996	420.1315002
183.787	-183.787				
174.026	-174.026	+	174.028	0.0477415	420.2883276
		+	531.763	0	421.1173697
536.529	-536.529				
189.983	-189.983	+	189.986	0.0360533	421.1266074
		+	313.327	0.00356303	421.1417264

313.191	-313.191				
308.838	-308.838	+	308.957	0.00368649	422.1106525
		+	327.615	0.00255238	422.1257096
327.403	-327.403				
275.775	-275.775	+	275.819	0.00742443	423.1335577
		+	238.417	0.0158949	423.1571942
238.403	-238.403				
209.396	-209.396	+	209.402	0.0264344	424.0918424
		+	233.277	0.0172441	424.1169923
233.264	-233.264				
35.366	-35.366	+	354.155	0.00125758	424.1413228
		+	204.755	0.0282852	425.1128593
204.749	-204.749				
201.932	-201.932	+	201.937	0.0295388	425.1365498
		+	24.941	0.0130016	425.1462343
24.939	-24.939				
306.621	-306.621	+	306.733	0.00376533	426.0517295
		+	347.834	0.00173818	426.1064728
347.431	-347.431				
285.058	-285.058	+	285.116	0.00606667	426.1569377
		+	215.565	0.0235919	427.0353666
215.558	-215.558				
230.561	-230.561	+	230.572	0.0176801	427.1526055
		+	229.404	0.0180488	428.0658078
229.393	-229.393				
210.683	-210.683	+	210.689	0.0261702	428.1363303
		+	265.534	0.0089683	429.0515024
265.502	-265.502				
210.356	-210.356	+	210.362	0.0262168	429.0619370
		+	192.978	0.034372	429.0719687
192.974	-192.974				
356.986	-356.986	+	357.538	0.00128932	429.0848509
		+	204.058	0.028482	429.1330830
204.053	-204.053				
21.237	-21.237	+	212.376	0.0252358	430.1521785
		+	314.839	0.00363429	431.0870670
314.696	-314.696				
251.015	-251.015	+	251.036	0.0125347	431.1472052

		+	28.727	0.005646	433.1044514
287.208	-287.208				
179.542	-179.542	+	179.544	0.043789	433.1533654
		+	531.416	0	434.1135791
535.947	-535.947				
177.277	-177.277	+	17.728	0.0454702	434.1469374
		+	460.726	0	435.1214025
450.453	-450.453				
326.758	-326.758	+	326.966	0.00270048	436.1287552
		+	233.665	0.0172179	436.1529367
233.653	-233.653				
19.255	-19.255	+	192.554	0.0347479	437.1131287
		+	28.432	0.00623646	438.1075733
284.264	-284.264				
267.029	-267.029	+	267.062	0.0083534	438.1432575
		+	452.675	0	438.2991085
443.397	-443.397				
442.477	-442.477	+	451.607	0	438.3003678
		+	266.758	0.008516	439.1525347
266.724	-266.724				
221.493	-221.493	+	221.502	0.0209736	439.2313318
		+	48.353	0	439.3025038
472.361	-472.361				
197.448	-197.448	+	197.452	0.0316097	440.0875733
		+	220.676	0.0212524	440.1240965
220.667	-220.667				
209.073	-209.073	+	209.079	0.026384	440.1475882
		+	237.962	0.0159671	440.1559508
237.948	-237.948				
22.787	-22.787	+	22.788	0.0185075	441.1427399
		+	386.953	0.000634615	441.1680986
385.448	-385.448				
289.642	-289.642	+	289.709	0.00549129	442.0464446
		+	309.749	0.00373187	442.0792535
309.627	-309.627				
302.114	-302.114	+	302.211	0.00411414	442.0818958
		+	353.525	0.0012552	442.1027957

35.304	-35.304				
266.403	-266.403	+	266.436	0.0084913	442.1358479
		+	178.613	0.0443319	442.1522261
178.611	-178.611				
23.125	-23.125	+	231.262	0.0178182	443.1474873
		+	257.743	0.0110531	443.1556399
257.718	-257.718				
3.8	-3.8	+	381.234	0.000608295	444.0623886
		+	220.859	0.0212369	444.1069222
220.851	-220.851				
251.361	-251.361	+	251.382	0.0125134	445.0462309
		+	191.115	0.0356278	445.0769649
191.112	-191.112				
488.643	-488.643	+	498.086	0	447.1336885
		+	312.868	0.00368112	447.1427024
312.733	-312.733				
220.364	-220.364	+	220.373	0.021392	447.1559627
		+	439.775	0	448.1424315
432.414	-432.414				
416.754	-416.754	+	421.404	0.000286645	448.1659245
		+	23.601	0.0165106	449.1146938
235.997	-235.997				
345.037	-345.037	+	34.541	0.00192806	449.1235411
		+	436.718	0	449.1495707
429.831	-429.831				
428.551	-428.551	+	435.205	0.00E+00	449.1504771
		+	188.803	0.0367107	449.1681491
188.799	-188.799				
284.784	-284.784	+	284.841	0.00615965	450.1082841
		+	401.226	0.000239782	450.1447561
398.765	-398.765				
37.608	-37.608	+	377.151	0.00100668	450.1537283
		+	177.131	0.0457155	451.0930279
177.129	-177.129				
305.203	-305.203	+	305.309	0.00388375	451.1279381
		+	176.261	0.0463389	451.1364739
176.259	-176.259				
250.124	-250.124	+	250.145	0.0127821	451.1409441

		+	286.977	0.00590562	451.1529650
286.916	-286.916				
244.999	-244.999	+	245.017	0.0137672	451.1650428
		+	20.277	0.0292333	452.1365407
202.765	-202.765				
201.874	-201.874	+	201.879	0.0296364	452.1478897
		+	176.284	0.0462941	453.1180698
176.282	-176.282				
875.789	-875.789	+	639.536	0	453.1451693
		+	260.714	0.0102804	453.1522923
260.686	-260.686				
236.837	-236.837	+	236.851	0.0161658	454.1524471
		+	334.751	0.00192053	454.1635801
334.486	-334.486				
199.533	-199.533	+	199.537	0.0303603	457.1632723
		+	357.707	0.00129183	458.0415448
357.151	-357.151				
316.199	-316.199	+	316.349	0.00337176	458.0753565
		+	293.768	0.00479227	458.1665115
293.693	-293.693				
234.235	-234.235	+	234.248	0.0170341	459.0485985
		+	171.646	0.0499992	459.0514896
171.644	-171.644				
224.763	-224.763	+	224.772	0.02004	459.1786877
		+	388.726	0.000647059	460.0571687
387.125	-387.125				
175.377	-175.377	+	175.379	0.0468106	460.1459362
		+	446.019	0	461.1361625
437.702	-437.702				
191.957	-191.957	+	19.196	0.0349475	461.1639325
		+	462.734	0	462.1454850
452.253	-452.253				
472.013	-472.013	+	483.197	0	463.1037050
		+	394.471	0.000673469	463.1281910
392.517	-392.517				
205.572	-205.572	+	205.578	0.0279889	463.1363681
		+	435.044	0	463.1473495

428.414	-428.414				
324.593	-324.593	+	324.788	0.0027907	463.1530291
		+	421.937	0.000289474	464.1243506
417.216	-417.216				
336.797	-336.797	+	337.083	0.00194305	464.1363493
		+	464.745	0	464.1617170
454.076	-454.076				
464.149	-464.149	+	475.418	0	465.1187576
		+	475.128	0	465.1204642
463.865	-463.865				
350.106	-350.106	+	350.546	0.00177037	465.1442693
		+	391.095	0.000664987	466.1411063
389.356	-389.356				
289.084	-289.084	+	28.915	0.00553563	466.1525044
		+	318.042	0.00319174	466.3291093
317.884	-317.884				
316.529	-316.529	+	316.681	0.00340116	466.3309150
		+	267.552	0.00836152	467.1243032
267.518	-267.518				
20.323	-20.323	+	203.235	0.0291432	467.1477768
		+	196.411	0.0319614	467.1586711
196.407	-196.407				
321.867	-321.867	+	322.046	0.00304559	467.3342645
		+	195.117	0.0330962	468.1333275
195.113	-195.113				
257.733	-257.733	+	257.759	0.0110631	468.1523557
		+	287.029	0.00591892	469.1635047
286.968	-286.968				
229.696	-229.696	+	229.707	0.0179832	470.1469052
		+	181.295	0.0422299	472.1507580
181.292	-181.292				
178.745	-178.745	+	178.747	0.0442948	472.1743929
		+	293.805	0.00479807	474.1619221
29.373	-29.373				
341.049	-341.049	+	341.377	0.00186111	475.1156271
		+	267.847	0.00829211	475.1741137
267.812	-267.812				
521.583	-521.583	+	522.431	0	476.1247996

		+	471.322	0	477.0741704
460.196	-460.196				
376.536	-376.536	+	377.626	0.000814318	477.0831044
		+	521.375	0	477.1224705
519.979	-519.979				
412.783	-412.783	+	41.685	0.000275862	477.1330321
		+	489.049	0	477.1565314
478.281	-478.281				
176.258	-176.258	+	17.626	0.0463196	478.1155089
		+	242.389	0.0146164	478.1275583
242.373	-242.373				
20.923	-20.923	+	209.236	0.0263941	478.1362196
		+	451.716	0	478.1409343
44.257	-44.257				
297.056	-297.056	+	297.139	0.00403965	478.1621037
		+	259.641	0.0106409	479.0987167
259.614	-259.614				
244.069	-244.069	+	244.086	0.0142441	479.1008663
		+	448.219	0	479.1359476
439.575	-439.575				
476.888	-476.888	+	487.775	0	479.1448388
		+	198.427	0.0307523	479.1727993
198.423	-198.423				
189.855	-189.855	+	189.858	0.0362374	480.1183443
		+	494.327	0.00E+00	480.1564614
484.226	-484.226				
37.074	-37.074	+	371.627	0.00122814	481.1639615
		+	189.566	0.0361909	481.2974575
189.562	-189.562				
198.661	-198.661	+	198.666	0.0307865	482.1447020
		+	204.821	0.0282991	482.1584553
204.816	-204.816				
32.582	-32.582	+	326.022	0.00282132	482.1723627
		+	216.352	0.0231136	484.0570468
216.344	-216.344				
31.023	-31.023	+	310.355	0.00375758	484.1630332
		+	352.497	0.00125283	485.1583187

352.028	-352.028				
396.412	-396.412	+	398.669	0.000466844	486.0729837
		+	218.723	0.0220526	486.1440801
218.716	-218.716				
195.854	-195.854	+	195.858	0.0321575	488.1687155
		+	18.307	0.0407338	488.1710203
183.068	-183.068				
34.112	-34.112	+	341.449	0.00187086	489.0477173
		+	191.483	0.0353798	489.0946731
191.479	-191.479				
317.933	-317.933	+	318.091	0.00319645	489.1442759
		+	28.302	0.00625764	490.1030032
282.966	-282.966				
254.855	-254.855	+	254.879	0.0118152	491.1122613
		+	184.884	0.0394576	491.1244477
184.881	-184.881				
44.864	-44.864	+	458.683	0	491.1361986
		+	424.816	0	492.1185983
4.197	-4.197				
442.304	-442.304	+	451.405	0.00E+00	492.1213874
		+	530.775	0	492.1377163
534.877	-534.877				
414.757	-414.757	+	419.107	0.000279365	492.1433279
		+	358.985	0.0011272	493.0476955
358.405	-358.405				
442.685	-442.685	+	451.849	0	493.1151895
		+	395.066	0.000451282	493.1273320
393.071	-393.071				
192.457	-192.457	+	19.246	0.0347026	493.1385319
		+	435.271	0	493.1421633
428.606	-428.606				
49.275	-49.275	+	501.462	0	493.1519508
		+	41.711	0.00027673	494.1357679
41.301	-41.301				
407.594	-407.594	+	410.985	0.000262687	494.1571086
		+	698.117	0	494.1951993
127.953	-127.953				
386.961	-386.961	+	388.553	0.000645477	495.1320497

		+	18.914	0.0364133	495.1432132
189.136	-189.136				
433.257	-433.257	+	440.772	0	495.1572756
		+	447.847	0	495.1676819
439.258	-439.258				
371.317	-371.317	+	372.222	0.00123871	495.1790856
		+	35.886	0.001125	495.1814848
358.283	-358.283				
423.105	-423.105	+	428.792	0	496.1516692
		+	317.843	0.00318235	496.1629625
317.686	-317.686				
359.095	-359.095	+	359.688	0.00114059	496.1715856
		+	31.726	0.00341606	497.1573676
317.106	-317.106				
635.221	-635.221	+	577.539	0	497.1731348
		+	223.224	0.0202827	498.1538798
223.215	-223.215				
488.333	-488.333	+	497.826	0	498.1668724
		+	466.113	0	498.1764000
455.328	-455.328				
533.041	-533.041	+	529.664	0	499.1684361
		+	299.103	0.00401519	499.1746201
299.015	-299.015				
178.306	-178.306	+	178.308	0.0445629	500.1934520
		+	306.837	0.00377036	501.1534369
306.725	-306.725				
370.688	-370.688	+	371.573	0.00122553	504.1549194
		+	236.829	0.0162189	505.1039379
236.815	-236.815				
446.049	-446.049	+	455.732	0	505.1153570
		+	299.933	0.00403562	506.0979800
299.843	-299.843				
369.428	-369.428	+	370.275	0.00121776	506.1171450
		+	294.916	0.00472195	507.0586026
294.838	-294.838				
179.582	-179.582	+	179.585	0.0437318	507.1193749
		+	1.779	0.0449129	507.1205558

177.898	-177.898				
46.839	-46.839	+	479.674	0	507.1314827
		+	250.194	0.0127927	507.1546880
250.174	-250.174				
25.234	-25.234	+	252.362	0.0121736	508.1142559
		+	359.393	0.00113609	508.1263683
358.805	-358.805				
496.577	-496.577	+	504.512	0	508.1353567
		+	1.887	0.0368341	508.1525638
188.696	-188.696				
261.809	-261.809	+	261.837	0.0101009	509.1138278
		+	258.898	0.0107505	509.1354224
258.872	-258.872				
423.505	-423.505	+	429.262	0	509.1471310
		+	428.073	0	510.1314551
422.491	-422.491				
346.229	-346.229	+	346.616	0.00194555	510.1475292
		+	4.4	0	510.1523227
432.605	-432.605				
340.154	-340.154	+	340.473	0.00184828	510.3213742
		+	440.528	0	511.1628324
433.051	-433.051				
312.158	-312.158	+	312.291	0.00365049	512.1084849
		+	476.013	0	512.1464720
464.733	-464.733				
405.094	-405.094	+	408.194	0.000252874	512.1593427
		+	319.632	0.00299104	512.3369596
319.466	-319.466				
433.503	-433.503	+	441.062	0	513.1471432
		+	455.753	0	513.1582522
446.068	-446.068				
421.114	-421.114	+	426.464	0	513.1684169
		+	259.348	0.0107058	513.1753925
259.321	-259.321				
200.773	-200.773	+	200.778	0.0304004	513.3391861
		+	204.774	0.0283008	513.3407590
204.769	-204.769				
488.946	-488.946	+	498.338	0	514.1491503

		+	477.637	0	514.1510187
466.342	-466.342				
432.629	-432.629	+	440.029	0	514.1621469
		+	326.597	0.00269194	514.1726853
326.391	-326.391				
180.382	-180.382	+	180.385	0.0432908	515.0784024
		+	241.927	0.0146769	515.1539661
241.911	-241.911				
259.213	-259.213	+	25.924	0.0108095	517.0954202
		+	391.015	0.000661654	517.1485727
389.282	-389.282				
179.433	-179.433	+	179.436	0.0437701	520.1149604
		+	490.769	0	520.1485805
480.186	-480.186				
397.843	-397.843	+	400.221	0.000237197	521.1115031
		+	175.768	0.0466091	521.1342673
175.766	-175.766				
181.915	-181.915	+	181.918	0.0414343	522.1442947
		+	213.448	0.0248553	523.0781680
213.441	-213.441				
440.232	-440.232	+	448.988	0	523.1261779
		+	191.717	0.0352128	523.1624124
191.714	-191.714				
431.089	-431.089	+	438.207	0	525.1422081
		+	23.844	0.0159188	525.1778572
238.426	-238.426				
213.114	-213.114	+	213.121	0.0249673	526.1489513
		+	219.792	0.021673	526.1513869
219.784	-219.784				
260.175	-260.175	+	260.203	0.0105125	526.1638781
		+	317.777	0.0033069	527.1469937
317.621	-317.621				
31.959	-31.959	+	319.757	0.00299552	527.1578405
		+	434.844	0	528.1426138
428.245	-428.245				
172.778	-172.778	+	17.278	0.0488792	528.1551809
		+	339.255	0.00183877	528.1616605

338.949	-338.949				
315.848	-315.848	+	315.996	0.00336691	529.1462624
		+	386.163	0.000628571	529.1535063
384.698	-384.698				
325.552	-325.552	+	325.752	0.0028125	529.1642465
		+	439.587	0	529.1735657
432.256	-432.256				
213.539	-213.539	+	213.546	0.0248849	530.1448402
		+	208.949	0.0264191	530.1585444
208.943	-208.943				
295.371	-295.371	+	29.545	0.00475092	530.1655207
		+	500.578	0	530.1754070
491.662	-491.662				
204.982	-204.982	+	204.987	0.0283458	531.0744826
		+	472.591	0	531.1288620
461.408	-461.408				
172.845	-172.845	+	172.847	0.048823	531.1657147
		+	252.592	0.012123	531.1792847
25.257	-25.257				
247.847	-247.847	+	247.866	0.013399	531.1806179
		+	368.967	0.00121519	533.1451460
368.157	-368.157				
753.483	-753.483	+	613.178	0	534.1059911
		+	38.354	0.000615385	534.1289792
382.203	-382.203				
242.231	-242.231	+	242.247	0.0145716	535.1030194
		+	249.737	0.0129662	535.1626964
249.717	-249.717				
473.889	-473.889	+	484.981	0	536.1232461
		+	291.165	0.00509349	538.0960264
291.096	-291.096				
263.281	-263.281	+	263.311	0.0096926	539.1241871
		+	389.546	0.000653465	540.0552667
387.899	-387.899				
316.951	-316.951	+	317.104	0.00341108	540.1429393
18.261	-18.261	+	182.613	0.0408897	541.0583219
27.327	-27.327	+	273.311	0.00744478	541.1357150

		+	336.275	0.00193333	541.1521997
335.996	-335.996				
28.498	-28.498	+	285.037	0.00605993	541.1750091
		+	234.552	0.0169556	542.1444464
234.539	-234.539				
262.415	-262.415	+	262.445	0.00977486	542.1592630
		+	253.127	0.0121411	542.1607962
253.105	-253.105				
214.085	-214.085	+	214.092	0.0246318	543.1536791
		+	259.445	0.0107254	543.1662290
259.418	-259.418				
272.211	-272.211	+	272.251	0.00744344	544.1623250
		+	398.671	0.000468085	544.1753523
396.414	-396.414				
225.347	-225.347	+	225.356	0.0197507	545.1089113
		+	182.826	0.0408917	546.0845385
182.824	-182.824				
304.168	-304.168	+	304.272	0.0041682	546.1650132
		+	361.413	0.00115895	546.1911140
360.784	-360.784				
360.776	-360.776	+	361.405	0.00115663	547.1252261
		+	237.961	0.0159552	547.1634203
237.947	-237.947				
385.423	-385.423	+	386.927	0.000631579	549.1182160
		+	457.783	0	549.1377036
447.847	-447.847				
178.605	-178.605	+	178.607	0.044313	549.1434110
		+	21.194	0.0256437	550.1034729
211.933	-211.933				
212.603	-212.603	+	21.261	0.0251972	550.1248991
		+	189.213	0.0363797	551.1215932
18.921	-18.921				
340.639	-340.639	+	340.963	0.00185789	551.1558030
		+	192.382	0.0347965	554.1824969
192.378	-192.378				
297.642	-297.642	+	297.727	0.00405978	555.1546022
		+	43.414	0.00E+00	556.1625431

427.649	-427.649				
243.775	-243.775	+	243.792	0.0141665	556.1864170
		+	262.464	0.00978404	557.1462592
262.434	-262.434				
201.762	-201.762	+	201.767	0.0297137	557.1691502
		+	207.016	0.0272255	557.1707756
20.701	-20.701				
202.748	-202.748	+	202.753	0.0292715	558.1426428
		+	270.139	0.00808284	558.1765989
270.102	-270.102				
236.563	-236.563	+	236.577	0.016248	559.1627140
		+	257.957	0.011093	559.1745078
257.931	-257.931				
294.092	-294.092	+	294.168	0.00482139	559.1861750
		+	190.814	0.0357673	560.1184540
190.811	-190.811				
210.516	-210.516	+	210.522	0.0262103	562.1741684
		+	245.615	0.013758	562.1859178
245.598	-245.598				
294.795	-294.795	+	294.873	0.0047162	563.0980381
		+	345.902	0.00193502	563.1185347
345.524	-345.524				
198.495	-198.495	+	198.499	0.0307545	564.2016361
		+	223.739	0.0200762	565.0784428
22.373	-22.373				
330.633	-330.633	+	330.868	0.00230421	565.1138474
		+	192.471	0.0347196	565.1633576
192.467	-192.467				
228.714	-228.714	+	228.725	0.0184299	566.1169316
		+	425.541	0	567.1288642
420.323	-420.323				
315.709	-315.709	+	315.856	0.00336207	568.1753544
		+	300.931	0.00405109	569.1338490
300.838	-300.838				
475.827	-475.827	+	486.795	0	569.1837685
		+	358.256	0.00129435	570.1424776
35.769	-35.769				
225.411	-225.411	+	22.542	0.0197639	570.1659729

		+	176.142	0.0464093	570.1763674
17.614	-17.614				
308.948	-308.948	+	309.068	0.00369648	570.1870548
		+	283.853	0.0062989	571.1637635
283.797	-283.797				
350.222	-350.222	+	350.663	0.00177365	571.1980687
		+	204.279	0.0283567	571.2025156
204.274	-204.274				
275.387	-275.387	+	27.543	0.00746914	572.1229616
		+	337.537	0.00195616	572.1573814
337.246	-337.246				
176.137	-176.137	+	176.139	0.04639	572.1714358
		+	228.419	0.0184397	572.1818366
228.409	-228.409				
200.255	-200.255	+	200.259	0.0303272	573.1543664
		+	280.405	0.00677516	573.1758768
280.354	-280.354				
410.489	-410.489	+	414.245	0.000267477	574.1737934
		+	272.808	0.00747337	574.1853946
272.768	-272.768				
220.313	-220.313	+	220.321	0.0213511	574.1969939
		+	238.732	0.0154578	577.1969353
238.718	-238.718				
183.056	-183.056	+	183.058	0.0407549	579.1764022
		+	192.357	0.0347795	580.0883944
192.354	-192.354				
188.368	-188.368	+	188.371	0.0369599	580.0930351
		+	172.719	0.0489411	581.0747242
172.717	-172.717				
237.725	-237.725	+	237.739	0.0160626	581.1061962
		+	258.075	0.0111031	582.1046012
25.805	-25.805				
411.916	-411.916	+	415.864	0.000273292	582.1541504
		+	378.471	0.000817978	582.1774427
377.349	-377.349				
519.696	-519.696	+	521.187	0	583.1631131
		+	174.644	0.0472996	583.1728680

174.642	-174.642				
37.764	-37.764	+	378.774	0.00081982	583.1846078
		+	238.709	0.0154462	584.1224137
238.694	-238.694				
182.964	-182.964	+	182.967	0.0407607	584.1449128
		+	450.555	0	584.1569389
441.573	-441.573				
370.841	-370.841	+	371.731	0.00123077	584.1676696
		+	385.172	0.000622642	584.1713200
383.756	-383.756				
652.841	-652.841	+	583.799	0	584.1945446
		+	253.209	0.0119931	585.1443160
253.186	-253.186				
258.876	-258.876	+	258.903	0.0107603	585.1534834
		+	233.003	0.0173209	585.1660515
23.299	-23.299				
444.575	-444.575	+	454.037	0	585.1789113
		+	426.468	0	585.1814564
421.117	-421.117				
337.549	-337.549	+	337.842	0.00181387	586.1370706
		+	575.772	0	586.1737531
630.458	-630.458				
493.879	-493.879	+	502.372	0	586.1831026
		+	192.584	0.034765	586.2075383
192.581	-192.581				
317.463	-317.463	+	317.619	0.0032924	586.2114154
		+	212.884	0.0249751	587.1340672
212.878	-212.878				
345.002	-345.002	+	345.374	0.0019246	587.1566288
		+	182.801	0.0408734	587.1670084
182.799	-182.799				
194.031	-194.031	+	194.035	0.0334761	587.1713648
48.871	-48.871	+	498.141	0	587.1945546
40.741	-40.741	+	410.779	0.000261905	588.1878863
		+	366.579	0.00119502	588.2022066
365.832	-365.832				
585.732	-585.732	+	557.323	0	589.2087794

		+	501.456	0	589.2108648
492.742	-492.742				
351.089	-351.089	+	351.544	0.00162547	590.1815382
		+	279.206	0.0069819	592.1965482
279.157	-279.157				
209.358	-209.358	+	209.364	0.0264041	593.1823922
		+	176.493	0.0461952	594.1653551
176.491	-176.491				
411.277	-411.277	+	415.138	0.000269939	596.0857789
		+	243.889	0.0141776	597.1012127
243.872	-243.872				
302.054	-302.054	+	302.151	0.00410881	597.1439111
		+	319.844	0.003	597.1655396
319.676	-319.676				
462.955	-462.955	+	474.194	0	598.1742950
		+	3.286	0.0024359	599.1335392
328.381	-328.381				
416.619	-416.619	+	421.249	0.000285714	599.1575228
		+	492.209	0	599.1770866
481.805	-481.805				
263.564	-263.564	+	263.594	0.00962738	599.1822703
		+	186.742	0.037813	600.1162226
186.739	-186.739				
312.544	-312.544	+	312.678	0.00366064	600.1530107
		+	222.662	0.0205759	600.1773585
222.654	-222.654				
469.151	-469.151	+	480.423	0	600.1894668
		+	477.558	0	600.1905298
466.263	-466.263				
542.977	-542.977	+	535.527	0	601.1734938
		+	490.043	0	601.1846219
479.379	-479.379				
476.425	-476.425	+	487.348	0	601.1951890
		+	400.576	0.00023913	602.1674429
398.168	-398.168				
552.615	-552.615	+	540.892	0	602.1817506

		+	341.381	0.00186435	602.1947392
341.053	-341.053				
445.767	-445.767	+	455.408	0	602.2058814
		+	231.292	0.0177541	603.1641006
231.281	-231.281				
588.318	-588.318	+	558.493	0	603.1893509
		+	553.739	0	603.1905820
578.015	-578.015				
534.154	-534.154	+	530.339	0	603.2086980
		+	522.927	0	603.2107270
522.341	-522.341				
51.752	-51.752	+	519.732	0	604.1839866
		+	514.348	0	604.1937804
509.742	-509.742				
389.329	-389.329	+	391.066	0.000663317	604.2217690
		+	256.214	0.0116743	605.1816702
256.189	-256.189				
29.067	-29.067	+	290.739	0.00519718	605.1925980
		+	442.801	0	605.2052817
434.974	-434.974				
25.508	-25.508	+	255.104	0.011759	606.0763753
		+	190.049	0.0360801	606.1765104
190.046	-190.046				
23.063	-23.063	+	230.641	0.0176925	607.0793647
		+	231.258	0.0178055	607.0806928
231.246	-231.246				
192.447	-192.447	+	192.451	0.0346856	608.0822951
		+	209.775	0.026394	608.1924213
209.769	-209.769				
189.773	-189.773	+	189.776	0.0362104	609.1770711
		+	196.781	0.0319084	610.1632818
196.776	-196.776				
250.959	-250.959	+	25.098	0.0126105	611.0941661
		+	402.265	0.000245125	611.1930433
399.716	-399.716				
500.559	-500.559	+	50.759	0	612.1532254
		+	174.687	0.0471924	613.1361396

174.685	-174.685				
430.637	-430.637	+	437.672	0	613.1476697
		+	236.944	0.0161778	613.1567625
23.693	-23.693				
389.873	-389.873	+	391.645	0.000670051	613.1616333
		+	396.139	0.000457143	613.1845780
39.407	-39.407				
245.106	-245.106	+	245.123	0.0137891	614.0964331
		+	222.937	0.0203812	614.1570521
222.928	-222.928				
432.204	-432.204	+	439.526	0	614.1691935
		+	433.233	0	614.1709633
42.688	-42.688				
495.624	-495.624	+	503.761	0	615.1640880
48.886	-48.886	+	498.267	0	615.1743073
32.985	-32.985	+	33.008	0.00228939	615.1873765
		+	511.236	0	615.1979499
505.436	-505.436				
48.049	-48.049	+	49.104	0	615.2010632
		+	411.521	0.000264264	616.1727470
408.072	-408.072				
443.101	-443.101	+	452.332	0	616.1850501
		+	331.141	0.00230794	616.2050217
330.904	-330.904				
477.644	-477.644	+	488.469	0	617.1786908
		+	350.913	0.00178026	617.1842578
350.468	-350.468				
452.086	-452.086	+	462.548	0	617.1928236
		+	18.144	0.0421244	617.2051100
181.438	-181.438				
269.892	-269.892	+	269.929	0.00806693	617.2163087
		+	217.534	0.0221735	618.1646598
217.526	-217.526				
282.408	-282.408	+	282.462	0.00622367	618.1740671
		+	273.362	0.00745233	618.1866734
273.321	-273.321				
458.127	-458.127	+	46.913	0	618.1985426

		+	450.771	0	618.2007931
441.758	-441.758				
355.522	-355.522	+	356.048	0.00127203	619.1836443
		+	29.066	0.00519109	619.1957131
290.591	-290.591				
47.036	-47.036	+	481.602	0	619.2040828
		+	246.169	0.0137097	620.1771536
246.151	-246.151				
285.346	-285.346	+	285.405	0.005951	620.1860164
		+	578.641	0	620.2060974
638.239	-638.239				
411.508	-411.508	+	4.154	0.000271605	620.2161911
		+	287.811	0.00556916	621.1870176
287.748	-287.748				
370.927	-370.927	+	371.819	0.0012334	624.1165842
		+	27.048	0.00801978	624.1873269
270.443	-270.443				
256.519	-256.519	+	256.544	0.0114667	625.1362273
		+	283.644	0.00627133	625.1728413
283.589	-283.589				
367.787	-367.787	+	368.586	0.00121008	626.1328578
		+	327.664	0.00255644	627.0920689
327.451	-327.451				
207.292	-207.292	+	207.298	0.0270712	627.1528885
		+	457.694	0	627.1643685
447.768	-447.768				
247.891	-247.891	+	24.791	0.0134099	627.1877046
		+	318.955	0.00321068	628.0582917
318.792	-318.792				
353.695	-353.695	+	35.419	0.00125996	628.0617006
		+	47.683	0	628.1478922
46.554	-46.554				
214.909	-214.909	+	214.916	0.0240461	628.1725558
		+	374.097	0.00124946	629.1071245
373.132	-373.132				
248.519	-248.519	+	248.538	0.013155	629.1431905
		+	301.711	0.00407712	629.1683279

301.615	-301.615				
520.302	-520.302	+	521.588	0	629.1777757
		+	532.602	0	629.1809284
537.945	-537.945				
248.068	-248.068	+	248.087	0.0132599	629.1924565
		+	481.647	0	630.1641498
470.405	-470.405				
339.701	-339.701	+	340.015	0.00184509	630.1870933
		+	341.869	0.0018807	630.2015137
341.536	-341.536				
351.361	-351.361	+	351.819	0.00163158	631.1574981
		+	419.635	0.00028115	631.1655754
415.217	-415.217				
270.007	-270.007	+	270.044	0.00807488	631.1717723
		+	429.887	0	631.1854348
424.037	-424.037				
435.545	-435.545	+	443.476	0	631.1960284
		+	455.427	0	632.1794341
445.784	-445.784				
444.464	-444.464	+	453.909	0	632.1806776
		+	522.255	0	632.1980900
521.314	-521.314				
582.817	-582.817	+	555.985	0	632.2014154
		+	249.773	0.0129876	632.2160142
249.753	-249.753				
359.508	-359.508	+	36.011	0.00114741	633.1741977
		+	372.145	0.00123605	633.1857356
371.243	-371.243				
47.447	-47.447	+	485.527	0	633.2019600
		+	451.709	0	633.2117610
442.565	-442.565				
263.758	-263.758	+	263.788	0.00932571	634.1839159
		+	401.493	0.000242424	634.1958321
39.901	-39.901				
464.712	-464.712	+	475.991	0	634.2059010
41.069	-41.069	+	414.473	0.000269113	635.1982071

		+	415.516	0.000272446	635.2011502
41.161	-41.161				
298.594	-298.594	+	298.681	0.00399496	636.2036269
		+	316.445	0.0033815	636.2114729
316.294	-316.294				
309.889	-309.889	+	310.013	0.00374725	637.2151869
42.276	-42.276	+	428.388	0	638.1677901
		+	183.845	0.0401745	640.1350449
183.843	-183.843				
453.516	-453.516	+	464.129	0	640.1832555
		+	497.566	0	641.1437582
488.023	-488.023				
225.653	-225.653	+	225.662	0.0198038	641.1675978
		+	188.887	0.0365947	642.1267235
188.884	-188.884				
27.516	-27.516	+	275.203	0.0074538	642.1457642
		+	276.277	0.00743983	642.1527657
276.233	-276.233				
2.42	-2.42	+	242.016	0.0146167	642.1762826
		+	197.732	0.0313845	642.1977380
197.728	-197.728				
428.836	-428.836	+	435.542	0	643.1591584
		+	440.778	0	643.1609284
433.262	-433.262				
249.123	-249.123	+	249.143	0.0131097	643.1832485
		+	408.222	0.000253602	644.1655449
405.119	-405.119				
253.839	-253.839	+	253.862	0.0118927	645.1640490
		+	449.695	0	645.1755153
440.837	-440.837				
294.026	-294.026	+	294.102	0.00481553	646.1578038
		+	464.739	0	646.1779317
45.407	-45.407				
403.744	-403.744	+	406.696	0.000250712	646.1821738
		+	252.455	0.012184	646.1947968
252.433	-252.433				
226.605	-226.605	+	226.615	0.0191318	647.1782109

		+	252.539	0.0121126	647.1824260
252.517	-252.517				
440.468	-440.468	+	449.264	0	647.1911712
		+	193.369	0.0339802	647.2030430
193.365	-193.365				
182.473	-182.473	+	182.476	0.0408924	648.1625848
		+	415.203	0.000270769	648.1750652
411.334	-411.334				
289.513	-289.513	+	28.958	0.00547856	648.1866082
		+	422.487	0	648.1946975
417.691	-417.691				
190.231	-190.231	+	190.234	0.0360325	648.2128996
		+	314.741	0.0036291	649.1750698
314.599	-314.599				
491.545	-491.545	+	500.483	0	649.1860543
		+	553.757	0	649.1972037
578.052	-578.052				
435.354	-435.354	+	443.251	0	649.2067989
		+	194.404	0.0333861	650.1782886
194.401	-194.401				
365.073	-365.073	+	3.658	0.00119255	650.1914034
		+	394.476	0.000675192	650.2112203
392.521	-392.521				
190.076	-190.076	+	19.008	0.0361145	651.1622554
		+	280.817	0.0065794	651.1866297
280.766	-280.766				
204.137	-204.137	+	204.142	0.0284061	651.1958909
		+	309.702	0.00372678	651.2129685
30.958	-30.958				
332.109	-332.109	+	332.356	0.0023268	653.1779172
		+	388.025	0.000640777	654.1631377
386.463	-386.463				
245.256	-245.256	+	245.274	0.0138	655.1469245
		+	207.619	0.0269146	655.1948623
207.613	-207.613				
430.324	-430.324	+	437.302	0	656.1784137
		+	253.107	0.0121307	657.1372442

253.085	-253.085				
22.491	-22.491	+	22.492	0.0200668	657.1628144
		+	313.955	0.00359322	657.1867699
313.816	-313.816				
191.963	-191.963	+	191.966	0.0349645	658.1585468
		+	302.904	0.00412484	658.1939624
302.804	-302.804				
362.109	-362.109	+	362.766	0.00116599	659.1543306
		+	234.746	0.0169161	660.1571179
234.733	-234.733				
340.562	-340.562	+	340.885	0.00185467	660.1745866
		+	401.369	0.000241758	661.1709225
398.896	-398.896				
244.326	-244.326	+	244.343	0.0142086	662.1532646
		+	383.672	0.000616822	662.1759504
382.328	-382.328				
383.856	-383.856	+	385.276	0.000624113	662.1926387
		+	273.571	0.00746748	663.1567372
27.353	-27.353				
273.177	-273.177	+	273.217	0.00752632	663.1765676
		+	318.475	0.00320593	663.1863122
318.315	-318.315				
217.239	-217.239	+	217.247	0.0223867	663.1978060
		+	235.277	0.0167062	663.2021105
235.264	-235.264				
197.231	-197.231	+	197.235	0.0317152	664.1728508
		+	225.478	0.0197772	664.1920237
225.469	-225.469				
20.233	-20.233	+	202.335	0.0295479	665.1925972
		+	431.867	0	665.2020689
425.722	-425.722				
23.484	-23.484	+	234.853	0.0168584	666.1872458
		+	327.835	0.00256051	666.2037650
327.621	-327.621				
340.551	-340.551	+	340.874	0.00185147	667.1581165
		+	231.241	0.0177929	667.1941361
231.229	-231.229				
27.177	-27.177	+	271.809	0.00742116	667.2081364

		+	403.182	0.000247191	669.1739536
400.554	-400.554				
247.076	-247.076	+	247.094	0.013504	670.1368337
		+	279.364	0.00686567	670.1580582
279.315	-279.315				
337.201	-337.201	+	337.491	0.00195286	671.1882315
		+	366.855	0.00119751	672.1536646
366.101	-366.101				
191.584	-191.584	+	191.587	0.0351872	673.1571127
		+	201.771	0.0297296	673.4836850
201.766	-201.766				
196.209	-196.209	+	196.213	0.0319858	674.4870921
		+	181.891	0.0414549	676.1677873
181.888	-181.888				
290.161	-290.161	+	290.229	0.00518501	676.1716113
		+	319.231	0.00298214	676.2225931
319.067	-319.067				
224.265	-224.265	+	224.274	0.0201193	677.1652492
		+	276.853	0.00726931	677.1777080
276.808	-276.808				
238.982	-238.982	+	238.996	0.0153122	677.1819247
		+	215.306	0.0239538	678.1374489
215.299	-215.299				
189.513	-189.513	+	189.516	0.0361738	678.1564497
		+	248.084	0.0132492	679.1824625
248.065	-248.065				
242.235	-242.235	+	242.251	0.0145828	679.1939917
		+	200.433	0.0303405	679.2183720
200.428	-200.428				
350.536	-350.536	+	350.982	0.00178358	681.1375035
		+	283.794	0.00629199	681.1973684
283.739	-283.739				
399.906	-399.906	+	402.472	0.000246499	683.1533209
		+	252.644	0.0121333	683.2139360
252.623	-252.623				
43.106	-43.106	+	438.172	0	685.1683592
		+	190.202	0.0359637	685.2213927

190.199	-190.199				
183.433	-183.433	+	183.436	0.0404413	687.1845645
		+	198.848	0.0308185	689.1650217
198.843	-198.843				
279.125	-279.125	+	279.173	0.00696706	689.2150795
		+	241.227	0.014968	690.1739657
241.212	-241.212				
348.464	-348.464	+	348.881	0.00176059	690.2225572
		+	248.177	0.0132707	691.2059532
248.158	-248.158				
351.228	-351.228	+	351.685	0.00162852	691.2326308
		+	226.746	0.0189803	692.1878814
226.736	-226.736				
397.053	-397.053	+	399.364	0.000469333	692.1915231
		+	477.028	0	692.2155465
465.736	-465.736				
189.995	-189.995	+	189.999	0.0360876	693.1741605
		+	209.689	0.0264189	693.2135459
209.683	-209.683				
312.613	-312.613	+	312.747	0.00367085	693.2227819
		+	356.075	0.00127447	693.2476040
355.548	-355.548				
193.959	-193.959	+	193.963	0.0334739	694.1935822
		+	272.944	0.00748842	694.2046853
272.904	-272.904				
323.623	-323.623	+	323.812	0.00275651	694.2274847
		+	617.401	0	694.2317031
770.777	-770.777				
201.956	-201.956	+	201.961	0.0295546	695.1912272
		+	220.525	0.0212891	695.2144909
220.517	-220.517				
211.055	-211.055	+	211.061	0.0259427	695.2259193
		+	197.917	0.0313584	696.1854664
197.913	-197.913				
230.909	-230.909	+	23.092	0.0177426	696.2472854
		+	203.915	0.0287077	697.2029973
20.391	-20.391				
231.794	-231.794	+	231.806	0.017563	698.1923532

		+	253.308	0.0120138	699.1268791
253.286	-253.286				
180.625	-180.625	+	180.628	0.0430105	700.2289706
		+	197.293	0.0316863	700.2307805
197.289	-197.289				
42.475	-42.475	+	430.724	0	701.1433151
		+	213.438	0.024878	702.1853050
213.432	-213.432				
184.574	-184.574	+	184.576	0.0395833	703.1572319
		+	289.846	0.00540163	703.1935283
289.779	-289.779				
214.917	-214.917	+	214.925	0.0240607	704.1930467
		+	363.151	0.00116836	704.2033776
362.485	-362.485				
218.067	-218.067	+	218.075	0.0221247	704.2260025
		+	219.703	0.0216182	705.1542088
219.694	-219.694				
234.008	-234.008	+	234.021	0.0170485	705.1620988
		+	22.827	0.018427	705.2074952
228.259	-228.259				
515.551	-515.551	+	518.397	0	705.2120434
		+	249.406	0.012991	706.1681173
249.386	-249.386				
314.079	-314.079	+	31.422	0.00360851	706.1714477
		+	230.074	0.0177251	706.1943773
230.063	-230.063				
271.438	-271.438	+	271.477	0.00749402	706.2057376
		+	263.642	0.00955281	706.2165104
263.612	-263.612				
192.488	-192.488	+	192.492	0.0347537	706.2433426
		+	194.138	0.0334511	707.2022524
194.134	-194.134				
211.736	-211.736	+	211.742	0.0256835	707.2147475
		+	53.914	0	707.2276148
549.409	-549.409				
213.024	-213.024	+	213.031	0.0249525	708.1848987
		+	208.266	0.0266787	708.1981070

20.826	-20.826				
251.921	-251.921	+	251.942	0.0123345	708.2082447
		+	38.318	0.000613953	708.2112228
381.859	-381.859				
336.229	-336.229	+	33.651	0.00193656	708.2231903
		+	298.375	0.00409034	708.2331275
298.289	-298.289				
325.627	-325.627	+	325.828	0.0028169	709.2050189
		+	255.303	0.0117795	709.2160930
25.528	-25.528				
533.158	-533.158	+	529.735	0	709.2434751
		+	223.923	0.02005	710.2016871
223.914	-223.914				
440.749	-440.749	+	449.593	0	710.2265778
		+	29.113	0.00508747	710.2469642
291.061	-291.061				
188.036	-188.036	+	188.039	0.0372134	711.1976611
		+	185.057	0.0390754	711.2233779
185.054	-185.054				
233.908	-233.908	+	233.921	0.0170999	711.2330662
		+	253.298	0.0120034	712.2427739
253.275	-253.275				
397.593	-397.593	+	39.995	0.000236559	712.3719676
		+	426.325	0	713.3755095
420.995	-420.995				
278.299	-278.299	+	278.346	0.00705932	718.1827181
		+	378.893	0.000823529	718.2051731
377.755	-377.755				
185.784	-185.784	+	185.787	0.0382969	719.1651701
		+	594.613	0	719.1919999
68.622	-68.622				
242.257	-242.257	+	242.273	0.014594	719.2142155
		+	313.636	0.00357806	720.1856156
313.498	-313.498				
364.192	-364.192	+	364.898	0.00118763	720.1977757
		+	489.649	0	720.2229992
478.942	-478.942				
209.657	-209.657	+	209.663	0.0263733	721.1238657

		+	181.272	0.0422912	721.1776564
181.269	-181.269				
483.567	-483.567	+	493.755	0	721.1819265
		+	248.259	0.0132814	721.1944515
24.824	-24.824				
466.579	-466.579	+	477.875	0	721.2069631
		+	28.687	0.00589238	721.2268000
286.809	-286.809				
184.203	-184.203	+	184.205	0.0398683	722.1646357
		+	501.939	0	722.2026355
49.334	-49.334				
433.104	-433.104	+	44.059	0	722.2131314
		+	535.036	0	722.2385809
542.122	-542.122				
449.412	-449.412	+	459.555	0	722.2416268
		+	214.337	0.0245508	723.1625695
21.433	-21.433				
375.417	-375.417	+	376.463	0.00100222	723.1841975
		+	278.825	0.00695228	723.2078703
278.777	-278.777				
262.784	-262.784	+	262.814	0.00971159	723.2120456
		+	509.198	0	723.2229671
502.688	-502.688				
308.771	-308.771	+	308.891	0.00368151	723.2431672
		+	456.958	0.00E+00	724.2170185
447.121	-447.121				
453.678	-453.678	+	464.308	0	724.2269817
		+	365.721	0.00119008	724.2543924
364.995	-364.995				
236.161	-236.161	+	236.174	0.0163382	725.2122074
		+	235.258	0.0166818	725.2255293
235.245	-235.245				
489.143	-489.143	+	498.502	0	725.2384932
		+	499.803	0	725.2416277
490.715	-490.715				
255.795	-255.795	+	255.819	0.0116799	726.2214323
		+	47.563	0	726.2427196

464.356	-464.356				
243.932	-243.932	+	243.949	0.0142108	727.2157696
		+	185.223	0.0388335	727.2257046
185.221	-185.221				
264.872	-264.872	+	264.903	0.00911877	727.2540530
		+	184.528	0.0396092	729.2082129
184.525	-184.525				
223.228	-223.228	+	223.237	0.020296	731.1536110
		+	276.368	0.00723909	731.1756274
276.323	-276.323				
305.225	-305.225	+	305.332	0.00388889	732.1142596
		+	290.837	0.00507547	732.1847435
290.769	-290.769				
303.833	-303.833	+	303.935	0.00414641	732.4849415
		+	401.787	0.000243767	733.1934997
399.279	-399.279				
389.554	-389.554	+	391.305	0.000668354	734.1284962
		+	421.013	0.00028479	734.1324576
416.415	-416.415				
564.296	-564.296	+	547.016	0.00E+00	734.2026240
		+	227.874	0.0184949	734.2249449
227.864	-227.864				
173.903	-173.903	+	173.905	0.0478107	734.5371260
		+	257.063	0.0111266	735.1584565
257.039	-257.039				
297.216	-297.216	+	2.973	0.00404969	735.1618142
		+	461.908	0	735.1854979
45.151	-45.151				
395.047	-395.047	+	397.193	0.000463158	735.2072506
		+	244.623	0.0141214	735.2341550
244.606	-244.606				
374.385	-374.385	+	375.394	0.00126039	736.1833695
		+	227.464	0.0186212	736.1929633
227.454	-227.454				
242.568	-242.568	+	242.584	0.0146615	736.2060957
		+	456.836	0	736.2182311
447.015	-447.015				
290.698	-290.698	+	290.767	0.00505758	736.2220932

		+	535.183	0	737.2020846
542.377	-542.377				
427.558	-427.558	+	434.033	0	737.2135720
		+	495.428	0	737.2238610
485.504	-485.504				
205.178	-205.178	+	205.183	0.028106	737.2367583
		+	523.886	0	738.1956723
523.819	-523.819				
345.269	-345.269	+	345.645	0.00193153	738.2072449
		+	409.062	0.00025656	738.2120505
405.874	-405.874				
394.938	-394.938	+	397.074	0.000461942	738.2232249
		+	453.259	0	738.2341202
443.902	-443.902				
260.124	-260.124	+	260.151	0.0106802	739.2052221
		+	555.327	0	739.2178629
581.398	-581.398				
565.706	-565.706	+	547.729	0	739.2376695
		+	487.999	0	739.2412327
477.131	-477.131				
545.276	-545.276	+	536.834	0	740.2128734
		+	542.971	0	740.2225949
556.498	-556.498				
453.716	-453.716	+	46.435	0	740.2377697
		+	593.724	0	740.2488591
683.326	-683.326				
709.873	-709.873	+	601.576	0	740.2507063
		+	286.518	0.00588578	740.4035358
286.457	-286.457				
26.346	-26.346	+	26.349	0.00961823	741.2222445
		+	43.823	0	741.2336278
431.108	-431.108				
434.277	-434.277	+	441.979	0	741.2537103
		+	241.271	0.0149794	741.4069441
241.255	-241.255				
217.607	-217.607	+	217.615	0.0221872	742.2274480
		+	343.805	0.00190409	742.2369317

343.451	-343.451				
187.947	-187.947	+	18.795	0.037334	743.2525813
		+	271.063	0.00767825	746.1646302
271.025	-271.025				
487.405	-487.405	+	497.046	0	748.1822809
		+	205.351	0.0279757	748.5156800
205.346	-205.346				
234.071	-234.071	+	234.084	0.0169971	748.5237063
		+	175.428	0.0467472	749.1642069
175.426	-175.426				
190.898	-190.898	+	190.902	0.0357304	749.1758505
		+	450.494	0	749.1878630
441.521	-441.521				
492.805	-492.805	+	501.507	0	750.1972037
		+	335.611	0.00192371	750.5320532
335.338	-335.338				
406.411	-406.411	+	409.661	0.00025731	750.5682475
		+	201.748	0.0296978	751.1828579
201.743	-201.743				
320.317	-320.317	+	320.487	0.00301353	751.1924979
		+	45.613	0	751.2035399
446.398	-446.398				
572.505	-572.505	+	551.096	0	751.2285955
		+	553.035	0	751.2315346
576.532	-576.532				
283.634	-283.634	+	283.689	0.0062782	751.5354638
		+	216.508	0.0228894	752.1862918
216.501	-216.501				
18.966	-18.966	+	189.664	0.0362595	752.2017283
		+	459.501	0	752.2131676
449.363	-449.363				
289.832	-289.832	+	289.899	0.00527273	752.2339235
		+	382.383	0.000612529	753.1958700
381.099	-381.099				
295.007	-295.007	+	295.086	0.00472772	753.2069944
		+	562.346	0	753.2174406
597.054	-597.054				
385.488	-385.488	+	386.996	0.000636145	753.2213657

		+	171.776	0.0498975	753.2334485
171.775	-171.775				
348.596	-348.596	+	349.015	0.00176384	753.2447044
		+	250.805	0.0126928	754.1958095
250.785	-250.785				
210.885	-210.885	+	210.891	0.0261121	754.2030860
		+	384.889	0.000619718	754.2171321
383.488	-383.488				
449.112	-449.112	+	459.216	0	754.2287583
		+	458.284	0	754.2308230
448.287	-448.287				
338.899	-338.899	+	339.205	0.00183562	755.2123988
		+	462.398	0	755.2325745
451.951	-451.951				
180.098	-180.098	+	180.101	0.0434756	756.2061204
		+	290.807	0.00506949	756.2164671
290.738	-290.738				
227.816	-227.816	+	227.826	0.0184822	756.2228249
		+	331.594	0.00231169	756.2340397
331.353	-331.353				
425.799	-425.799	+	431.958	0	756.2445773
		+	229.238	0.0181947	757.2160746
229.227	-229.227				
241.324	-241.324	+	24.134	0.0149909	757.2460053
		+	205.952	0.0275787	758.1987427
205.946	-205.946				
203.092	-203.092	+	203.097	0.0292496	758.5023145
		+	253.662	0.0118722	759.1844134
25.364	-25.364				
177.252	-177.252	+	177.254	0.0454882	759.5040756
		+	20.232	0.0295003	760.2155393
202.315	-202.315				
326.176	-326.176	+	326.381	0.00283465	760.5162567
		+	189.978	0.0360361	761.1987696
189.975	-189.975				
330.251	-330.251	+	330.484	0.00229677	761.5191234
		+	328.043	0.00256459	761.5205545

327.827	-327.827				
251.106	-251.106	+	251.127	0.0125556	762.5226475
		+	228.001	0.0184533	763.2158514
22.799	-22.799				
409.639	-409.639	+	413.285	0.000265861	764.1758955
		+	229.388	0.0180975	764.1971074
229.377	-229.377				
184.478	-184.478	+	184.481	0.0396311	765.1741031
		+	190.074	0.0360973	765.1828848
190.071	-190.071				
192.708	-192.708	+	192.711	0.0347126	765.1967080
		+	499.573	0	765.2079256
490.436	-490.436				
417.201	-417.201	+	421.921	0.000288525	766.1923956
		+	326.807	0.0026962	766.2142870
326.599	-326.599				
314.409	-314.409	+	314.551	0.00362393	767.1965247
		+	390.888	0.000658354	767.2131848
389.162	-389.162				
578.665	-578.665	+	554.047	0	767.2241784
		+	438.342	0.00E+00	768.2080669
431.203	-431.203				
316.433	-316.433	+	316.584	0.0033864	768.2265032
		+	257.497	0.0110233	768.2321488
257.472	-257.472				
288.438	-288.438	+	288.502	0.00549145	768.2671171
		+	407.262	0.000252149	769.2134232
404.254	-404.254				
437.021	-437.021	+	445.218	0	769.2287180
		+	375.619	0.00126316	769.2357726
374.603	-374.603				
471.063	-471.063	+	482.284	0	769.2410584
		+	460.985	0	770.2239961
450.684	-450.684				
358.429	-358.429	+	359.009	0.00112941	770.2354825
39.885	-39.885	+	401.319	0.000241096	771.2264458
		+	317.626	0.00329722	771.2317658

31.747	-31.747				
431.344	-431.344	+	438.508	0	771.2454094
		+	243.715	0.0141555	772.1789681
243.698	-243.698				
310.688	-310.688	+	310.815	0.00377317	772.2365730
		+	443.458	0	772.2436199
43.553	-43.553				
289.414	-289.414	+	28.948	0.0054659	772.2516117
182.103	-182.103	+	182.106	0.0412819	773.2441030
		+	417.147	0.000277603	774.1950782
413.043	-413.043				
182.626	-182.626	+	182.629	0.0409079	775.1786072
		+	222.536	0.0207396	776.2086735
222.527	-222.527				
269.718	-269.718	+	269.754	0.008059	776.2134185
		+	190.762	0.0357752	776.5018524
190.759	-190.759				
284.039	-284.039	+	284.095	0.00620902	776.5115833
		+	172.451	0.0492222	776.5841924
172.449	-172.449				
176.465	-176.465	+	176.467	0.0461566	777.5055538
		+	357.236	0.00128185	778.1770334
356.689	-356.689				
189.862	-189.862	+	189.865	0.0362546	778.2268496
		+	228.952	0.0183878	778.5175038
228.941	-228.941				
386.633	-386.633	+	388.205	0.000643902	779.1875769
		+	440.586	0	780.1944478
4.331	-4.331				
474.448	-474.448	+	485.507	0	781.2035869
		+	228.231	0.0184143	782.1859676
22.822	-22.822				
497.185	-497.185	+	504.987	0	782.2075258
		+	409.798	0.000258065	782.2119924
406.533	-406.533				
233.213	-233.213	+	233.226	0.0172193	782.2234602
		+	243.049	0.0144973	782.4983903

243.032	-243.032				
257.409	-257.409	+	257.435	0.0110036	782.5007749
		+	257.803	0.011073	783.1873602
257.777	-257.777				
185.846	-185.846	+	185.849	0.0382706	783.1921582
		+	242.352	0.0146052	783.2082484
242.336	-242.336				
46.052	-46.052	+	471.663	0	783.2189516
		+	469.904	0	783.2208025
458.853	-458.853				
28.033	-28.033	+	28.038	0.00676791	784.2034348
		+	443.083	0	784.2260018
435.212	-435.212				
202.028	-202.028	+	202.033	0.0295863	784.3939576
		+	324.445	0.00276923	785.2046416
324.253	-324.253				
431.019	-431.019	+	438.124	0	785.2352004
		+	213.617	0.0248344	786.2156460
21.361	-21.361				
255.921	-255.921	+	255.945	0.0116332	786.2236045
		+	485.907	0	786.2377333
474.876	-474.876				
501.125	-501.125	+	50.802	0	786.2410026
		+	244.327	0.0142665	786.4088395
24.431	-24.431				
241.172	-241.172	+	241.188	0.0149567	786.4108803
		+	408.227	0.000254335	787.2177116
405.123	-405.123				
350.225	-350.225	+	350.667	0.00177695	787.2222847
		+	373.389	0.00124675	787.2383530
372.448	-372.448				
403.227	-403.227	+	406.125	0.00025	787.2414015
		+	320.854	0.00302262	788.2242765
320.682	-320.682				
192.768	-192.768	+	192.772	0.0345997	788.2460726
		+	252.141	0.0122307	790.1679899
25.212	-25.212				
230.895	-230.895	+	230.906	0.01773	793.1662933

		+	200.501	0.0304046	795.1842030
200.497	-200.497				
204.789	-204.789	+	204.795	0.028268	796.2031877
		+	432.835	0	797.1978329
426.543	-426.543				
240.676	-240.676	+	240.691	0.0150561	797.2124005
		+	232.731	0.0174155	798.2041251
232.719	-232.719				
36.709	-36.709	+	36.787	0.00120502	799.2147922
		+	186.329	0.0380505	799.2268242
186.326	-186.326				
342.683	-342.683	+	343.028	0.00189735	799.2528891
		+	309.016	0.00369147	801.2314205
308.896	-308.896				
191.445	-191.445	+	191.448	0.0353711	802.2348956
		+	225.755	0.0196745	803.1861188
225.745	-225.745				
510.495	-510.495	+	514.882	0	803.2463196
		+	214.017	0.0246599	807.2178212
21.401	-21.401				
230.274	-230.274	+	230.285	0.0176304	807.5064127
		+	25.232	0.0121529	808.1767033
252.299	-252.299				
383.901	-383.901	+	385.324	0.000625592	812.2474931
		+	464.422	0	812.2524972
453.782	-453.782				
286.921	-286.921	+	286.982	0.00591226	813.2320940
		+	187.682	0.0375592	813.2549186
187.679	-187.679				
303.076	-303.076	+	303.176	0.00413559	814.2372671
		+	184.034	0.0400234	814.2429584
184.031	-184.031				
366.623	-366.623	+	367.391	0.0012	814.2649982
		+	304.538	0.00402635	815.2474955
304.433	-304.433				
193.838	-193.838	+	193.841	0.0336596	817.2366444
18.574	-18.574	+	185.743	0.0382795	817.2640916

		+	361.419	0.00116129	820.5752712
36.079	-36.079				
258.676	-258.676	+	258.702	0.0107407	821.5584703
		+	351.065	0.00178692	825.2440715
350.618	-350.618				
197.864	-197.864	+	197.868	0.0313261	826.2028172
31.693	-31.693	+	317.083	0.00340611	826.2254202
27.752	-27.752	+	277.566	0.00723028	827.2348232
47.268	-47.268	+	483.835	0	827.2581648
		+	410.321	0.000259587	827.2618831
407.001	-407.001				
505.189	-505.189	+	511.055	0	828.2442826
		+	242.482	0.0146502	828.2651951
242.465	-242.465				
255.832	-255.832	+	255.856	0.0116229	829.2021742
		+	230.477	0.0176676	829.2459095
230.465	-230.465				
356.882	-356.882	+	357.433	0.00128682	829.2520489
		+	440.201	0	829.2763482
432.774	-432.774				
259.643	-259.643	+	25.967	0.0106507	830.2332129
		+	399.388	0.000470588	830.2586767
397.076	-397.076				
415.907	-415.907	+	420.428	0.000282051	830.2612519
		+	200.461	0.0303565	830.2825730
200.456	-200.456				
250.563	-250.563	+	250.583	0.0126822	831.2653411
		+	261.666	0.0100821	832.2522580
261.638	-261.638				
396.001	-396.001	+	398.223	0.00046438	832.2761310
		+	256.518	0.0115346	835.5379368
256.494	-256.494				
251.788	-251.788	+	251.809	0.0123137	835.5408069
		+	255.805	0.0116696	836.5390702
255.781	-255.781				
250.801	-250.801	+	250.822	0.0127033	836.5411089

		+	354.223	0.00126236	839.2235229
353.727	-353.727				
192.241	-192.241	+	192.244	0.0348443	839.2456822
		+	232.692	0.017466	840.2342901
23.268	-23.268				
376.047	-376.047	+	377.117	0.00100444	840.2552972
		+	22.243	0.0207261	841.2137710
222.421	-222.421				
835.349	-835.349	+	631.637	0	841.2384784
		+	58.483	0	841.2418578
65.586	-65.586				
289.029	-289.029	+	289.094	0.00552928	842.2227192
		+	40.993	0.000258824	842.2455655
406.651	-406.651				
277.931	-277.931	+	277.978	0.00702954	842.2727536
		+	189.657	0.0362423	843.2268996
189.654	-189.654				
224.251	-224.251	+	224.261	0.020106	843.2325785
		+	219.893	0.0216584	843.2432641
219.885	-219.885				
623.287	-623.287	+	573.047	0	843.2556414
		+	427.373	0	844.2382839
421.892	-421.892				
492.608	-492.608	+	501.347	0	844.2425357
		+	400.346	0.000237838	844.2570693
397.957	-397.957				
389.882	-389.882	+	391.655	0.000671756	844.2617253
		+	303.001	0.00413021	845.2346068
302.901	-302.901				
325.072	-325.072	+	325.269	0.00280811	845.2446552
		+	361.027	0.001152	845.2672956
360.406	-360.406				
554.195	-554.195	+	541.743	0	845.2716766
24.591	-24.591	+	245.928	0.0137646	846.2319359
		+	521.349	0	846.2548359

51.994	-51.994				
509.195	-509.195	+	513.958	0	846.2757240
		+	175.872	0.0465748	847.2621305
17.587	-17.587				
257.468	-257.468	+	257.494	0.0110135	847.2867868
196.271	-196.271	+	196.275	0.0319736	848.2674108
		+	292.683	0.0050491	848.2716085
29.261	-29.261				
293.919	-293.919	+	293.995	0.00480387	848.3985179
		+	282.745	0.00624401	848.4016476
282.691	-282.691				
224.669	-224.669	+	224.678	0.0201411	849.5891865
		+	22.424	0.0201561	849.5907927
22.423	-22.423				
345.767	-345.767	+	346.149	0.00194203	851.5326798
		+	341.455	0.00187413	855.2165139
341.127	-341.127				
293.055	-293.055	+	293.128	0.00496154	855.2241096
		+	245.977	0.0137756	855.2428309
24.596	-24.596				
177.629	-177.629	+	177.631	0.0451	856.2138253
		+	414.366	0.000268293	856.2252018
410.595	-410.595				
466.264	-466.264	+	477.559	0	856.2480447
		+	444.102	0	856.2527328
436.075	-436.075				
511.594	-511.594	+	515.657	0	857.2349515
		+	337.409	0.00194958	857.2562470
33.712	-33.712				
415.963	-415.963	+	420.493	0.000282958	858.2365362
		+	557.956	0	858.2419819
587.127	-587.127				
500.536	-500.536	+	507.573	0	858.2667155
		+	18.209	0.0413026	859.2154248
182.088	-182.088				
514.162	-514.162	+	517.444	0	859.2488534
		+	524.811	0	859.2511422

525.259	-525.259				
348.014	-348.014	+	348.425	0.00175092	859.2670363
		+	442.074	0	859.2718658
434.358	-434.358				
201.973	-201.973	+	201.978	0.0295704	860.2334518
		+	483.053	0	860.2554211
471.862	-471.862				
419.133	-419.133	+	424.158	0	860.2826508
		+	200.743	0.0303843	861.2542635
200.738	-200.738				
763.711	-763.711	+	615.699	0	861.2665703
		+	199.069	0.0306264	862.2481795
199.065	-199.065				
229.086	-229.086	+	229.097	0.0182663	862.2517807
		+	549.905	0	862.2666080
570.073	-570.073				
458.159	-458.159	+	469.164	0	862.2717744
		+	189.276	0.0362861	862.6579345
189.273	-189.273				
186.224	-186.224	+	186.227	0.0381092	862.6604643
		+	410.662	0.000261128	863.2824248
407.305	-407.305				
201.179	-201.179	+	201.184	0.0301723	863.5690324
		+	201.169	0.0301563	863.5704866
201.164	-201.164				
200.883	-200.883	+	200.887	0.0302682	864.5725694
		+	173.527	0.0479675	867.5274926
173.525	-173.525				
218.539	-218.539	+	218.547	0.0221527	868.2161915
		+	449.807	0	870.2275032
440.932	-440.932				
408.839	-408.839	+	412.383	0.00026506	870.2333861
		+	389.086	0.000648649	871.2140026
387.465	-387.465				
405.373	-405.373	+	408.505	0.000255072	871.2358542
		+	179.188	0.0440034	872.2161342
179.185	-179.185				
266.633	-266.633	+	266.667	0.00849952	872.2221337

		+	499.072	0	872.2463055
489.829	-489.829				
733.418	-733.418	+	608.021	0	873.2276942
		+	619.923	0	873.2318063
781.495	-781.495				
660.262	-660.262	+	586.314	0	873.2469066
		+	456.241	0	873.2519658
446.494	-446.494				
370.456	-370.456	+	371.334	0.00122293	874.2351808
		+	514.568	0	874.2620855
510.051	-510.051				
624.543	-624.543	+	57.353	0	875.2458557
		+	485.079	0	875.2663484
473.993	-473.993				
253.652	-253.652	+	253.674	0.0118825	875.2720728
		+	336.089	0.00192691	876.2456991
335.812	-335.812				
396.305	-396.305	+	398.553	0.000465608	876.2523199
		+	301.393	0.00406667	876.2660216
301.298	-301.298				
474.399	-474.399	+	48.546	0	876.2776844
		+	532.032	0	876.2811741
536.982	-536.982				
448.785	-448.785	+	458.847	0	877.2617577
		+	245.408	0.0138219	877.2770265
24.539	-24.539				
378.977	-378.977	+	380.166	0.000604119	877.2817966
		+	326.342	0.00283019	878.2650501
326.137	-326.137				
249.118	-249.118	+	249.138	0.0130989	878.2831571
		+	257.204	0.0111564	879.2655880
257.179	-257.179				
187.231	-187.231	+	187.234	0.0375169	879.2768796
		+	226.332	0.0194274	879.5640080
226.322	-226.322				
263.892	-263.892	+	263.923	0.0093346	880.5674024
		+	212.802	0.0251107	884.2054284

212.796	-212.796				
330.027	-330.027	+	330.258	0.00229308	885.2152970
		+	442.032	0	886.2257747
434.322	-434.322				
31.922	-31.922	+	319.385	0.00298659	887.2322000
		+	337.703	0.00181081	887.2571895
337.411	-337.411				
237.032	-237.032	+	237.046	0.0162018	888.2136417
		+	52.385	0	888.2415965
523.764	-523.764				
275.431	-275.431	+	275.474	0.00747683	889.2246197
		+	494.956	0	889.2459989
484.954	-484.954				
26.052	-26.052	+	260.547	0.0102709	889.2731724
		+	500.033	0	890.2571800
490.995	-490.995				
215.802	-215.802	+	21.581	0.0234195	890.2628197
		+	306.662	0.00375532	891.2426487
306.551	-306.551				
592.877	-592.877	+	560.523	0	891.2628060
		+	212.794	0.0250959	891.5972493
212.787	-212.787				
314.376	-314.376	+	314.518	0.00361878	892.2450468
		+	511.833	0	892.2728814
506.252	-506.252				
437.528	-437.528	+	445.814	0	893.2564042
		+	36.064	0.0011497	893.2758549
360.028	-360.028				
22.611	-22.611	+	22.612	0.0195253	894.2545323
		+	270.426	0.00800395	894.2771941
270.389	-270.389				
278.173	-278.173	+	27.822	0.00705185	894.2862867
		+	18.099	0.0427515	896.5447738
180.988	-180.988				
310.643	-310.643	+	310.769	0.00376796	901.2375122
		+	205.381	0.0279912	902.2435697
205.375	-205.375				
297.672	-297.672	+	297.757	0.00406484	903.2248452

		+	419.314	0.000280255	903.2533383
414.937	-414.937				
400.979	-400.979	+	403.648	0.000247887	904.2365972
		+	23.115	0.0177803	904.2559999
231.139	-231.139				
2.196	-2.196	+	219.608	0.0215501	904.6227950
		+	453.062	0	905.2425917
443.731	-443.731				
17.688	-17.688	+	176.882	0.0460202	905.2569179
		+	36.364	0.00117791	905.2678837
362.963	-362.963				
460.449	-460.449	+	471.589	0	905.2722410
		+	473.222	0	906.2524980
462.015	-462.015				
382.768	-382.768	+	384.133	0.000618267	907.2560830
		+	277.141	0.0072	907.2623434
277.095	-277.095				
434.895	-434.895	+	442.708	0	907.2736130
		+	379.377	0.000827273	908.2676901
37.822	-37.822				
251.761	-251.761	+	251.782	0.012492	908.2739580
		+	275.231	0.00746146	909.2731080
275.188	-275.188				
190.726	-190.726	+	190.729	0.0358483	910.2840617
		+	291.842	0.00512992	915.2174523
291.771	-291.771				
432.886	-432.886	+	440.333	0	917.2328197
		+	322.085	0.00305023	918.2362091
321.906	-321.906				
397.174	-397.174	+	399.495	0.000235925	919.2477316
		+	421.595	0.000287582	920.2538637
416.919	-416.919				
218.764	-218.764	+	218.772	0.0219699	921.2341454
		+	521.057	0	921.2635104
5.195	-5.195				
301.954	-301.954	+	302.051	0.00410349	922.2463201
		+	396.282	0.000458333	922.2655264

394.202	-394.202				
477.074	-477.074	+	487.946	0	923.2449835
		+	180.274	0.0433822	923.2682815
180.271	-180.271				
20.816	-20.816	+	208.166	0.0266185	923.2725300
		+	171.716	0.0499544	932.2836518
171.714	-171.714				
218.905	-218.905	+	218.913	0.0220113	935.2825989
		+	288.413	0.00548519	939.6630200
288.349	-288.349				
295.126	-295.126	+	295.205	0.0047451	940.6660492
		+	34.871	0.00175735	948.2765078
348.295	-348.295				
217.878	-217.878	+	217.886	0.0220697	948.6263565
		+	189.485	0.0362264	949.2628244
189.482	-189.482				
264.556	-264.556	+	264.587	0.00919312	949.2833380
		+	238.186	0.0158473	950.2655656
238.172	-238.172				
52.183	-52.183	+	522.593	0	950.2931900
		+	227.242	0.0189523	951.2756182
227.232	-227.232				
19.491	-19.491	+	194.914	0.0330779	952.3075793
		+	221.881	0.020888	953.2927341
221.873	-221.873				
201.043	-201.043	+	201.048	0.0301083	954.6624481
		+	173.443	0.0478895	961.2752266
173.441	-173.441				
276.872	-276.872	+	276.917	0.00727691	962.2547314
		+	466.475	0	963.2877856
455.661	-455.661				
394.311	-394.311	+	396.399	0.000460733	963.2921662
		+	364.544	0.00118519	964.2725928
363.846	-363.846				
297.813	-297.813	+	297.899	0.004075	964.2938229
		+	299.929	0.0040305	965.2763743
299.838	-299.838				
354.383	-354.383	+	35.489	0.00126476	965.3042625

		+	284.115	0.00621586	966.2620360
284.059	-284.059				
375.095	-375.095	+	37.613	0.000997792	966.2876719
		+	280.765	0.00666667	966.2923229
280.715	-280.715				
405.712	-405.712	+	408.882	0.000255814	967.2938100
		+	187.533	0.0375748	967.3174687
18.753	-18.753				
259.036	-259.036	+	259.063	0.0107898	968.2757454
		+	438.598	0	968.3036945
431.419	-431.419				
291.627	-291.627	+	291.697	0.00511772	968.6394251
		+	288.506	0.00549772	968.6418525
288.442	-288.442				
261.843	-261.843	+	261.872	0.0101104	969.6455393
		+	243.935	0.0141886	971.5661533
243.918	-243.918				
228.975	-228.975	+	228.986	0.0183146	972.5713017
		+	185.712	0.0383514	976.5910466
185.709	-185.709				
129.829	-129.829	+	705.569	0	977.2673545
		+	205.152	0.028108	977.2733234
205.147	-205.147				
187.983	-187.983	+	187.986	0.0373687	978.2536020
		+	239.216	0.0153353	978.2736604
239.201	-239.201				
220.132	-220.132	+	22.014	0.0215054	978.2975401
		+	436.447	0	979.2837779
429.602	-429.602				
354.934	-354.934	+	35.545	0.00126718	980.2665021
		+	359.501	0.00113834	980.2874068
358.911	-358.911				
393.162	-393.162	+	395.163	0.000452442	980.2920105
		+	249.493	0.013023	981.2734114
249.473	-249.473				
610.235	-610.235	+	567.875	0	981.2985164
		+	545.674	0	981.3009928

561.672	-561.672				
265.594	-265.594	+	265.626	0.00897692	982.2832040
		+	225.319	0.0197374	982.3041988
225.309	-225.309				
183.307	-183.307	+	18.331	0.0405917	982.6937784
		+	310.851	0.00377839	983.3149517
310.724	-310.724				
226.747	-226.747	+	226.757	0.0189932	984.2977506
		+	301.093	0.00405627	992.2780982
300.999	-300.999				
180.021	-180.021	+	180.023	0.0435248	992.2830971
		+	42.338	0	993.2634323
418.462	-418.462				
281.958	-281.958	+	282.011	0.00642424	994.2666867
		+	374.697	0.00125764	994.2947959
373.712	-373.712				
210.549	-210.549	+	210.555	0.0262256	994.6574714
		+	423.057	0	995.2781907
418.184	-418.184				
41.616	-41.616	+	420.719	0.000283871	995.2812974
		+	372.442	0.00124138	996.2836693
371.531	-371.531				
189.384	-189.384	+	189.387	0.0362299	996.3078208
		+	199.908	0.0303333	996.3116843
199.903	-199.903				
300.409	-300.409	+	300.502	0.00404592	996.6731947
		+	463.118	0	997.2942609
4.526	-4.526				
262.706	-262.706	+	262.736	0.00980245	997.6767282
18.612	-18.612	+	186.123	0.038164	998.2971308
17.354	-17.354	+	173.542	0.0479593	999.5966706

Supplementary Table S5: Annotations for upregulated ion signals in positive-ion mode

Input Mass	Matched Mass	Delta	ppm	Name	Formula	Adduct
	319.1362	0.0012	3.7602	ST 18:3;O;S	C18H24O4S	[M+H-H2O]+
319.1374607						
325.3211755	325.3213	0.0002	0.6148	SPB 20:3;O	C20H37NO	[M+NH4]+

	331.1362	0.0011	3.3219	ST 19:4;O;S	C19H24O4S	[M+H-H2O]+
331.1373706						
333.1531016	333.1519	0.0012	3.6020	ST 19:3;O;S	C19H26O4S	[M+H-H2O]+
343.3317952	343.3319	0.0001	0.2913	NAE 18:1	C20H39NO2	[M+NH4]+
	343.3319	0.0001	0.2913	SPB 20:2;O2	C20H39NO2	[M+NH4]+
343.3317952						
345.1531976	345.1519	0.0013	3.7665	ST 20:4;O;S	C20H26O4S	[M+H-H2O]+
	347.1675	0.0012	3.4565	ST 20:3;O;S	C20H28O4S	[M+H-H2O]+
347.1687687						
349.1480156	349.1468	0.0012	3.4369	ST 19:4;O;S	C19H24O4S	[M+H]+
	349.1468	0.0012	3.4369	ST 19:3;O2;S	C19H26O5S	[M+H-H2O]+
349.1480156						
350.1433888	350.1421	0.0013	3.7128	ST 18:5;O;S	C18H20O4S	[M+NH4]+
353.3196232	353.3203	0.0006	1.6982	ST 26:2;O	C26H42O	[M+H-H2O]+
	353.3203	0.0001	0.2830	ST 26:2;O	C26H42O	[M+H-H2O]+
353.3203860						
359.1686511	359.1675	0.0011	3.0626	ST 21:4;O;S	C21H28O4S	[M+H-H2O]+
	361.1832	0.0012	3.3224	ST 21:3;O;S	C21H30O4S	[M+H-H2O]+
361.1843408						
361.2735192	361.2737	0.0002	0.5536	FA 23:5;O	C23H36O3	[M+H]+
	361.2737	0.0002	0.5536	ST 23:2;O3	C23H36O3	[M+H]+
361.2735192						
361.2735192	361.2737	0.0002	0.5536	DG O-20:4	C23H38O4	[M+H-H2O]+
	361.2737	0.0002	0.5536	FA 23:4;O2	C23H38O4	[M+H-H2O]+
361.2735192						
361.2735192	361.2737	0.0002	0.5536	MG 20:4	C23H38O4	[M+H-H2O]+
	361.2737	0.0002	0.5536	MG O-20:5;O	C23H38O4	[M+H-H2O]+
361.2735192						
361.2735192	361.2737	0.0002	0.5536	ST 23:1;O4	C23H38O4	[M+H-H2O]+
	363.1625	0.0012	3.3043	ST 20:4;O;S	C20H26O4S	[M+H]+
363.1636839						
363.1636839	363.1625	0.0012	3.3043	ST 20:3;O2;S	C20H28O5S	[M+H-H2O]+
	364.1577	0.0011	3.0207	ST 19:5;O;S	C19H22O4S	[M+NH4]+
364.1588324						
364.1953474	364.1941	0.0013	3.5695	NAT 18:5	C20H31NO4S	[M+H-H2O]+
	366.137	0.0015	4.0968	ST 18:5;O2;S	C18H20O5S	[M+NH4]+

366.1384877						
369.3472729	369.3475	0.0003	0.8122	NAE 20:2	C22H41NO2	[M+NH4]+
	372.1546	0.0017	4.5680	SPBP 15:3;O3	C15H28NO6PNa	[M+Na]+
372.1529331						
373.1843581	373.1857	0.0013	3.4835	ST 18:1;O8	C18H28O8	[M+H]+
	373.1832	0.0012	3.2156	ST 22:4;O;S	C22H30O4S	[M+H-H2O]+
373.1843581						
375.1995820	375.1988	0.0007	1.8657	ST 22:3;O;S	C22H32O4S	[M+H-H2O]+
	375.2013	0.0010	2.6652	ST 18:0;O8	C18H30O8	[M+H]+
375.2003728						
376.1587361	376.1577	0.0010	2.6585	ST 18:4;O3;T	C20H27NO5S	[M+H-H2O]+
	376.1966	0.0012	3.1898	CAR 10:2;O4	C17H29NO8	[M+H]+
376.1953814						
377.1791788	377.1781	0.0011	2.9164	ST 21:4;O;S	C21H28O4S	[M+H]+
	377.1781	0.0011	2.9164	ST 21:3;O2;S	C21H30O5S	[M+H-H2O]+
377.1791788						
378.1746463	378.1734	0.0013	3.4376	ST 18:3;O3;T	C20H29NO5S	[M+H-H2O]+
	378.1734	0.0013	3.4376	ST 20:5;O;S	C20H24O4S	[M+NH4]+
378.1746463						
378.2114299	378.2122	0.0008	2.1152	CAR 10:1;O4	C17H31NO8	[M+H]+
	379.1574	0.0012	3.1649	ST 20:4;O2;S	C20H26O5S	[M+H]+
379.1585383						
379.1585383	379.1574	0.0012	3.1649	ST 20:3;O3;S	C20H28O6S	[M+H-H2O]+
	382.1836	0.0017	4.4481	CAR 10:2;O3	C17H29NO7Na	[M+Na]+
382.1819579						
387.1991509	387.1988	0.0003	0.7748	ST 23:4;O;S	C23H32O4S	[M+H-H2O]+
	387.2013	0.0003	0.7748	ST 19:1;O8	C19H30O8	[M+H]+
387.2010035						
388.1585608	388.1577	0.0009	2.3186	ST 19:5;O3;T	C21H27NO5S	[M+H-H2O]+
	388.1966	0.0014	3.6064	CAR 11:3;O4	C18H29NO8	[M+H]+
388.1951821						
388.1951821	388.1966	0.0014	3.6064	ST 18:2;O8	C18H26O8	[M+NH4]+
	389.217	0.0013	3.3400	ST 19:0;O8	C19H32O8	[M+H]+
389.2156576						
389.2156576	389.2145	0.0012	3.0831	ST 23:3;O;S	C23H34O4S	[M+H-H2O]+
	390.1652	0.0003	0.7689	LPE 10:1	C15H30NO7PNa	[M+Na]+
390.1648975						
390.1648975	390.1652	0.0003	0.7689	LPE O-10:2;O	C15H30NO7PNa	[M+Na]+

	390.1652	0.0003	0.7689	SPBP 15:2;O4	C15H30NO7PNa	[M+Na]+
390.1648975						
390.1746250	390.1734	0.0013	3.3319	ST 19:4;O3;T	C21H29NO5S	[M+H-H2O]+
	390.1734	0.0013	3.3319	ST 21:6;O;S	C21H24O4S	[M+NH4]+
390.1746250						
390.2111229	390.2122	0.0011	2.8190	CAR 11:2;O4	C18H31NO8	[M+H]+
	390.2122	0.0011	2.8190	ST 18:1;O8	C18H28O8	[M+NH4]+
390.2111229						
391.1949168	391.1938	0.0012	3.0675	ST 22:4;O;S	C22H30O4S	[M+H]+
	391.1938	0.0012	3.0675	ST 22:3;O2;S	C22H32O5S	[M+H-H2O]+
391.1949168						
392.1895046	392.189	0.0005	1.2749	ST 19:3;O3;T	C21H31NO5S	[M+H-H2O]+
	392.189	0.0005	1.2749	ST 21:5;O;S	C21H26O4S	[M+NH4]+
392.1895046						
392.1904242	392.189	0.0014	3.5697	ST 19:3;O3;T	C21H31NO5S	[M+H-H2O]+
	392.189	0.0014	3.5697	ST 21:5;O;S	C21H26O4S	[M+NH4]+
392.1904242						
392.2267104	392.2279	0.0012	3.0594	CAR 11:1;O4	C18H33NO8	[M+H]+
	392.2254	0.0013	3.3144	NAT 20:5	C22H35NO4S	[M+H-H2O]+
392.2267104						
392.2267104	392.2279	0.0012	3.0594	ST 18:0;O8	C18H30O8	[M+NH4]+
	393.1884	0.0018	4.5780	ST 19:1;O7	C19H30O7Na	[M+Na]+
393.1865842						
402.1745898	402.1734	0.0012	2.9838	ST 20:5;O3;T	C22H29NO5S	[M+H-H2O]+
	402.2122	0.0012	2.9835	CAR 12:3;O4	C19H31NO8	[M+H]+
402.2110343						
402.2110343	402.2098	0.0012	2.9835	CAR 10:0;O4	C17H33NO8Na	[M+Na]+
	402.2122	0.0012	2.9835	ST 19:2;O8	C19H28O8	[M+NH4]+
402.2110343						
403.1947850	403.1938	0.0010	2.4802	ST 23:5;O;S	C23H30O4S	[M+H]+
	403.1938	0.0010	2.4802	ST 23:4;O2;S	C23H32O5S	[M+H-H2O]+
403.1947850						
404.1904323	404.189	0.0014	3.4637	ST 20:4;O3;T	C22H31NO5S	[M+H-H2O]+
	404.189	0.0014	3.4637	ST 22:6;O;S	C22H26O4S	[M+NH4]+
404.1904323						
404.2265147	404.2279	0.0014	3.4634	CAR 12:2;O4	C19H33NO8	[M+H]+
	404.2254	0.0011	2.7213	NAT 21:6	C23H35NO4S	[M+H-H2O]+

404.2265147						
404.2265147	404.2279	0.0014	3.4634	ST 19:1;O8	C19H30O8	[M+NH4]+
	406.2435	0.0011	2.7077	CAR 12:1;O4	C19H35NO8	[M+H]+
406.2424444						
406.2424444	406.241	0.0014	3.4462	NAT 21:5	C23H37NO4S	[M+H-H2O]+
	406.2435	0.0011	2.7077	ST 19:0;O8	C19H32O8	[M+NH4]+
406.2424444						
407.2025922	407.204	0.0014	3.4381	ST 20:1;O7	C20H32O7Na	[M+Na]+
	408.1839	0.0012	2.9399	ST 19:4;O3;T	C21H29NO5S	[M+H]+
408.1851531						
408.1851531	408.1839	0.0012	2.9399	ST 19:3;O4;T	C21H31NO6S	[M+H-H2O]+
	408.1839	0.0012	2.9399	ST 21:5;O2;S	C21H26O5S	[M+NH4]+
408.1851531						
413.1788664	413.1781	0.0008	1.9362	ST 24:6;O2;S	C24H30O5S	[M+H-H2O]+
	418.2047	0.0011	2.6303	ST 21:4;O3;T	C23H33NO5S	[M+H-H2O]+
418.2057537						
418.2057537	418.2047	0.0011	2.6303	ST 23:6;O;S	C23H28O4S	[M+NH4]+
	418.2435	0.0009	2.1519	CAR 13:2;O4	C20H35NO8	[M+H]+
418.2426266						
418.2426266	418.2435	0.0009	2.1519	ST 20:1;O8	C20H32O8	[M+NH4]+
	420.1839	0.0012	2.8559	ST 20:5;O3;T	C22H29NO5S	[M+H]+
420.1851121						
420.1851121	420.1839	0.0012	2.8559	ST 20:4;O4;T	C22H31NO6S	[M+H-H2O]+
	420.1839	0.0012	2.8559	ST 22:6;O2;S	C22H26O5S	[M+NH4]+
420.1851121						
420.2214937	420.2203	0.0012	2.8556	NAT 21:6;O	C23H35NO5S	[M+H-H2O]+
	420.2203	0.0012	2.8556	ST 21:3;O3;T	C23H35NO5S	[M+H-H2O]+
420.2214937						
420.2214937	420.2203	0.0012	2.8556	ST 23:5;O;S	C23H30O4S	[M+NH4]+
	421.1776	0.0012	2.8492	ST 24:6;O4	C24H30O4K	[M+K]+
421.1787760						
421.1816213	421.1809	0.0007	1.6620	ST 21:1;O;S	C21H34O4SK	[M+K]+
	426.238	0.0006	1.4077	SPBP 20:3;O2	C20H38NO5PNa	[M+Na]+
426.2373468						
428.1885862	428.189	0.0004	0.9342	ST 22:6;O3;T	C24H31NO5S	[M+H-H2O]+
	428.1915	0.0001	0.2335	ST 18:2;O8;G	C20H29NO9	[M+H]+
428.1914504						
429.1741833	429.173	0.0012	2.7961	ST 24:6;O3;S	C24H30O6S	[M+H-H2O]+

	430.2047	0.0010	2.3245	ST 22:5;O3;T	C24H33NO5S	[M+H-H2O]+
430.2056849						
433.2055549	433.2043	0.0012	2.7701	ST 24:5;O2;S	C24H32O5S	[M+H]+
	433.2043	0.0012	2.7701	ST 24:4;O3;S	C24H34O6S	[M+H-H2O]+
433.2055549						
434.2008199	434.1996	0.0013	2.9940	ST 21:5;O3;T	C23H31NO5S	[M+H]+
	434.1996	0.0013	2.9940	ST 21:4;O4;T	C23H33NO6S	[M+H-H2O]+
434.2008199						
434.2008199	434.1996	0.0013	2.9940	ST 23:6;O2;S	C23H28O5S	[M+NH4]+
	434.236	0.0010	2.3029	NAT 22:6;O	C24H37NO5S	[M+H-H2O]+
434.2369750						
434.2369750	434.236	0.0010	2.3029	ST 22:3;O3;T	C24H37NO5S	[M+H-H2O]+
	434.236	0.0010	2.3029	ST 24:5;O;S	C24H32O4S	[M+NH4]+
434.2369750						
444.2215666	444.2228	0.0012	2.7013	ST 19:1;O8;G	C21H33NO9	[M+H]+
	444.2203	0.0013	2.9265	ST 23:5;O3;T	C25H35NO5S	[M+H-H2O]+
444.2215666						
444.2215666	444.2203	0.0013	2.9265	ST 25:7;O;S	C25H30O4S	[M+NH4]+
	445.2181	0.0015	3.3691	ST 18:2;O8;G	C20H29NO9	[M+NH4]+
445.2165350						
446.2012658	446.1996	0.0017	3.8100	ST 22:6;O3;T	C24H31NO5S	[M+H]+
	446.1996	0.0017	3.8100	ST 22:5;O4;T	C24H33NO6S	[M+H-H2O]+
446.2012658						
446.2368759	446.236	0.0009	2.0169	ST 23:4;O3;T	C25H37NO5S	[M+H-H2O]+
	446.236	0.0009	2.0169	ST 25:6;O;S	C25H32O4S	[M+NH4]+
446.2368759						
447.1938730	447.1932	0.0007	1.5653	ST 26:7;O4	C26H32O4K	[M+K]+
	447.2337	0.0012	2.6832	ST 18:1;O8;G	C20H31NO9	[M+NH4]+
447.2325220						
449.2117487	449.2122	0.0005	1.1131	ST 23:1;O;S	C23H38O4SK	[M+K]+
	449.2494	0.0015	3.3389	ST 18:0;O8;G	C20H33NO9	[M+NH4]+
449.2478405						
450.2320295	450.2309	0.0012	2.6653	ST 22:4;O3;T	C24H35NO5S	[M+H]+
	450.2309	0.0012	2.6653	NAT 22:6;O2	C24H37NO6S	[M+H-H2O]+
450.2320295						
450.2320295	450.2309	0.0012	2.6653	ST 22:3;O4;T	C24H37NO6S	[M+H-H2O]+
	450.2309	0.0012	2.6653	ST 24:5;O2;S	C24H32O5S	[M+NH4]+

450.2320295						
457.1082308	457.1082	0.0001	0.2188	ST 22:6;O3;S	C22H26O6SK	[M+K]+
	457.2326	0.0004	0.8748	LPA 18:2	C21H39O7PNa	[M+Na]+
457.2321743						
457.2321743	457.2326	0.0004	0.8748	LPA O-18:3;O	C21H39O7PNa	[M+Na]+
	458.2278	0.0007	1.5276	LPC 12:2	C20H38NO7PNa	[M+Na]+
458.2271266						
458.2271266	458.2278	0.0007	1.5276	LPC O-12:3;O	C20H38NO7PNa	[M+Na]+
	458.2278	0.0007	1.5276	LPE 15:2	C20H38NO7PNa	[M+Na]+
458.2271266						
458.2271266	458.2278	0.0007	1.5276	LPE O-15:3;O	C20H38NO7PNa	[M+Na]+
	458.2278	0.0007	1.5276	SPBP 20:3;O4	C20H38NO7PNa	[M+Na]+
458.2271266						
458.2357052	458.236	0.0002	0.4365	ST 24:5;O3;T	C26H37NO5S	[M+H-H2O]+
	458.236	0.0002	0.4365	ST 26:7;O;S	C26H32O4S	[M+NH4]+
458.2357052						
461.2116482	461.2122	0.0006	1.3009	ST 24:2;O;S	C24H38O4SK	[M+K]+
	461.2494	0.0019	4.1192	ST 19:1;O8;G	C21H33NO9	[M+NH4]+
461.2474443						
461.2474443	461.2452	0.0022	4.7697	ST 28:6;O3	C28H38O3K	[M+K]+
	462.2309	0.0012	2.5961	ST 23:5;O3;T	C25H35NO5S	[M+H]+
462.2320582						
462.2320582	462.2309	0.0012	2.5961	ST 23:4;O4;T	C25H37NO6S	[M+H-H2O]+
	462.2309	0.0012	2.5961	ST 25:6;O2;S	C25H32O5S	[M+NH4]+
462.2320582						
463.1913219	463.1913	0.0000	0.0000	ST 26:7;O;S	C26H32O4SNa	[M+Na]+
	463.1915	0.0002	0.4318	ST 23:2;O2;S	C23H36O5SK	[M+K]+
463.1913219						
463.2265060	463.2261	0.0004	0.8635	ST 22:6;O3;T	C24H31NO5S	[M+NH4]+
	463.265	0.0012	2.5903	ST 19:0;O8;G	C21H35NO9	[M+NH4]+
463.2638092						
471.2476332	471.2482	0.0006	1.2732	LPA 19:2	C22H41O7PNa	[M+Na]+
	471.2482	0.0006	1.2732	LPA O-19:3;O	C22H41O7PNa	[M+Na]+
471.2476332						
471.2476332	471.2466	0.0010	2.1220	LPS 13:1	C19H36NO9P	[M+NH4]+
	471.2466	0.0010	2.1220	LPS O-13:2;O	C19H36NO9P	[M+NH4]+
471.2476332						
472.2520657	472.2516	0.0005	1.0588	ST 25:5;O3;T	C27H39NO5S	[M+H-H2O]+

472.2520657	472.2516	0.0005	1.0588	ST 27:7;O;S	C27H34O4S	[M+NH4]+
473.2122307	473.2122	0.0000	0.0000	ST 25:3;O;S	C25H38O4SK	[M+K]+
474.2321724	474.2309	0.0013	2.7413	ST 24:6;O3;T	C26H35NO5S	[M+H]+
474.2321724	474.2309	0.0013	2.7413	ST 24:5;O4;T	C26H37NO6S	[M+H-H2O]+
474.2321724	474.2309	0.0013	2.7413	ST 26:7;O2;S	C26H32O5S	[M+NH4]+
474.2675295	474.2673	0.0003	0.6326	NAT 25:7;O	C27H41NO5S	[M+H-H2O]+
474.2675295	474.2673	0.0003	0.6326	ST 25:4;O3;T	C27H41NO5S	[M+H-H2O]+
474.2675295	474.2673	0.0003	0.6326	ST 27:6;O;S	C27H36O4S	[M+NH4]+
475.3049532	475.3054	0.0005	1.0520	DG 25:6;O	C28H42O6	[M+H]+
475.3049532	475.3054	0.0005	1.0520	DG O-25:7;O2	C28H42O6	[M+H]+
475.3049532	475.3054	0.0005	1.0520	FA 28:7;O4	C28H42O6	[M+H]+
475.3049532	475.3054	0.0005	1.0520	ST 22:3;O;Hex	C28H42O6	[M+H]+
475.3049532	475.3054	0.0005	1.0520	ST 28:4;O6	C28H42O6	[M+H]+
475.3049532	475.3054	0.0005	1.0520	DG 25:5;O2	C28H44O7	[M+H-H2O]+
475.3049532	475.3054	0.0005	1.0520	ST 22:1;O;GlcA	C28H44O7	[M+H-H2O]+
475.3049532	475.3054	0.0005	1.0520	ST 22:2;O2;Hex	C28H44O7	[M+H-H2O]+
475.3049532	475.3054	0.0005	1.0520	ST 28:3;O7	C28H44O7	[M+H-H2O]+
478.2377271	478.2388	0.0011	2.3001	NAT 22:4	C24H41NO4SK	[M+K]+
479.2223119	479.2226	0.0003	0.6260	ST 27:6;O;S	C27H36O4SNa	[M+Na]+
479.2223119	479.2228	0.0005	1.0434	ST 24:1;O2;S	C24H40O5SK	[M+K]+
481.2292840	481.2309	0.0017	3.5326	LPS 14:3	C20H34NO9P	[M+NH4]+
481.2292840	481.2309	0.0017	3.5326	LPS O-14:4;O	C20H34NO9P	[M+NH4]+
481.2318368	481.2326	0.0007	1.4546	LPA 20:4	C23H39O7PNa	[M+Na]+
481.2318368	481.2326	0.0007	1.4546	LPA O-20:5;O	C23H39O7PNa	[M+Na]+
	481.2326	0.0007	1.4546	PA O-20:4	C23H39O7PNa	[M+Na]+

481.2318368						
481.2318368	481.2309	0.0009	1.8702	LPS 14:3	C20H34NO9P	[M+NH4]+
	481.2309	0.0009	1.8702	LPS O-14:4;O	C20H34NO9P	[M+NH4]+
481.2318368						
481.2318368	481.2327	0.0009	1.8702	LPG O-14:0	C20H43O8PK	[M+K]+
	483.2482	0.0005	1.0347	LPA 20:3	C23H41O7PNa	[M+Na]+
483.2476980						
483.2476980	483.2482	0.0005	1.0347	LPA O-20:4;O	C23H41O7PNa	[M+Na]+
	483.2482	0.0005	1.0347	PA O-20:3	C23H41O7PNa	[M+Na]+
483.2476980						
484.2161067	484.2152	0.0009	1.8587	ST 25:7;O4;T	C27H35NO6S	[M+H-H2O]+
	484.2516	0.0002	0.4130	ST 26:6;O3;T	C28H39NO5S	[M+H-H2O]+
484.2518131						
485.2555618	485.2567	0.0012	2.4729	ST 25:2;O4;S	C25H40O7S	[M+H]+
	485.2567	0.0012	2.4729	ST 25:1;O5;S	C25H42O8S	[M+H-H2O]+
485.2555618						
485.2634049	485.2639	0.0005	1.0304	LPA 20:2	C23H43O7PNa	[M+Na]+
	485.2639	0.0005	1.0304	LPA O-20:3;O	C23H43O7PNa	[M+Na]+
485.2634049						
485.2634049	485.2639	0.0005	1.0304	PA O-20:2	C23H43O7PNa	[M+Na]+
	485.3109	0.0005	1.0303	ST 20:0;O3;Hex	C26H44O8	[M+H]+
485.3114294						
485.3114294	485.3109	0.0005	1.0303	ST 26:1;O8	C26H44O8	[M+H]+
	486.2673	0.0004	0.8226	ST 26:5;O3;T	C28H41NO5S	[M+H-H2O]+
486.2668272						
486.2668272	486.2673	0.0004	0.8226	ST 28:7;O;S	C28H36O4S	[M+NH4]+
	487.2279	0.0017	3.4891	ST 26:3;O;S	C26H40O4SK	[M+K]+
487.2262030						
489.2427566	489.2435	0.0008	1.6352	ST 26:2;O;S	C26H42O4SK	[M+K]+
	490.2258	0.0013	2.6518	ST 24:6;O4;T	C26H35NO6S	[M+H]+
490.2270477						
490.2270477	490.2258	0.0013	2.6518	ST 24:5;O5;T	C26H37NO7S	[M+H-H2O]+
	490.2258	0.0013	2.6518	ST 26:7;O3;S	C26H32O6S	[M+NH4]+
490.2270477						
491.2223642	491.2226	0.0003	0.6107	ST 28:7;O;S	C28H36O4SNa	[M+Na]+
	491.2228	0.0004	0.8143	ST 25:2;O2;S	C25H40O5SK	[M+K]+
491.2223642						
491.2582624	491.2574	0.0008	1.6285	ST 24:6;O3;T	C26H35NO5S	[M+NH4]+

	491.2592	0.0009	1.8320	ST 26:1;O;S	C26H44O4SK	[M+K]+
491.2582624						
492.2543553	492.2544	0.0001	0.2031	NAT 23:4	C25H43NO4SK	[M+K]+
	492.3812	0.0019	3.8588	LPC O-18:0	C26H56NO6P	[M+H-H2O]+
492.3793266						
492.3793266	492.3812	0.0019	3.8588	LPE O-21:0	C26H56NO6P	[M+H-H2O]+
	492.3812	0.0019	3.8588	Cer 31:6;O	C31H51NO2Na	[M+Na]+
492.3793266						
492.3793266	492.3812	0.0019	3.8588	NAE 29:6	C31H51NO2Na	[M+Na]+
	492.3813	0.0020	4.0619	Cer 28:1;O2	C28H55NO3K	[M+K]+
492.3793266						
492.3793266	492.3813	0.0020	4.0619	NAE 26:1;O	C28H55NO3K	[M+K]+
	492.3812	0.0004	0.8124	LPC O-18:0	C26H56NO6P	[M+H-H2O]+
492.3808804						
492.3808804	492.3812	0.0004	0.8124	LPE O-21:0	C26H56NO6P	[M+H-H2O]+
	492.3812	0.0003	0.6093	Cer 31:6;O	C31H51NO2Na	[M+Na]+
492.3808804						
492.3808804	492.3812	0.0003	0.6093	NAE 29:6	C31H51NO2Na	[M+Na]+
	492.3813	0.0005	1.0155	Cer 28:1;O2	C28H55NO3K	[M+K]+
492.3808804						
492.3808804	492.3813	0.0005	1.0155	NAE 26:1;O	C28H55NO3K	[M+K]+
	496.3302	0.0016	3.2237	NAT 22:0;O3	C24H49NO7S	[M+H]+
496.3286006						
496.3286006	496.3269	0.0017	3.4252	CAR 20:4;O3	C27H45NO7	[M+H]+
	496.3269	0.0017	3.4252	ST 19:0;O2;HexNAc	C27H45NO7	[M+H]+
496.3286006						
496.3286006	496.3269	0.0017	3.4252	ST 25:1;O6;G	C27H45NO7	[M+H]+
	496.3269	0.0017	3.4252	CAR 20:3;O4	C27H47NO8	[M+H-H2O]+
496.3286006						
496.3286006	496.3269	0.0017	3.4252	ST 25:0;O7;G	C27H47NO8	[M+H-H2O]+
	496.3269	0.0017	3.4252	DG 24:5;O2	C27H42O7	[M+NH4]+
496.3286006						
496.3286006	496.3269	0.0017	3.4252	ST 21:1;O;GlcA	C27H42O7	[M+NH4]+
	496.3269	0.0017	3.4252	ST 21:2;O2;Hex	C27H42O7	[M+NH4]+
496.3286006						
496.3286006	496.3269	0.0017	3.4252	ST 27:3;O7	C27H42O7	[M+NH4]+
	497.2874	0.0000	0.0000	LPG 17:1	C23H45O9P	[M+H]+

497.2873459						
497.2873459	497.2874	0.0000	0.0000	LPG O-17:2;O	C23H45O9P	[M+H]+
	497.2874	0.0000	0.0000	PA 20:0;O	C23H45O9P	[M+H]+
497.2873459						
497.2873459	497.2874	0.0000	0.0000	LPG 17:0;O	C23H47O10P	[M+H-H2O]+
	497.2874	0.0000	0.0000	DG 25:6;O	C28H42O6Na	[M+Na]+
497.2873459						
497.2873459	497.2874	0.0000	0.0000	DG O-25:7;O2	C28H42O6Na	[M+Na]+
	497.2874	0.0000	0.0000	FA 28:7;O4	C28H42O6Na	[M+Na]+
497.2873459						
497.2873459	497.2874	0.0000	0.0000	ST 22:3;O;Hex	C28H42O6Na	[M+Na]+
	497.2874	0.0000	0.0000	ST 28:4;O6	C28H42O6Na	[M+Na]+
497.2873459						
497.2873459	497.2875	0.0002	0.4022	DG 22:1;O2	C25H46O7K	[M+K]+
	504.3061	0.0003	0.5949	LPC 15:0	C23H48NO7PNa	[M+Na]+
504.3057323						
504.3057323	504.3061	0.0003	0.5949	LPC O-15:1;O	C23H48NO7PNa	[M+Na]+
504.3057323	504.3061	0.0003	0.5949	LPE 18:0	C23H48NO7PNa	[M+Na]+
	504.3061	0.0003	0.5949	LPE O-18:1;O	C23H48NO7PNa	[M+Na]+
504.3057323						
505.2331022	505.2326	0.0005	0.9896	LPA 22:6	C25H39O7PNa	[M+Na]+
	505.2326	0.0005	0.9896	PA O-22:6	C25H39O7PNa	[M+Na]+
505.2331022						
505.2331022	505.2327	0.0004	0.7917	LPA 19:1;O	C22H43O8PK	[M+K]+
	505.2327	0.0004	0.7917	LPG O-16:2	C22H43O8PK	[M+K]+
505.2331022						
505.3671694	505.367	0.0002	0.3958	NAT 24:2;O	C26H49NO5S	[M+NH4]+
	506.2337	0.0008	1.5803	NAT 23:5;O	C25H41NO5SK	[M+K]+
506.2345142						
506.2345142	506.2337	0.0008	1.5803	ST 23:2;O3;T	C25H41NO5SK	[M+K]+
	506.3687	0.0019	3.7522	CAR 19:0;O4	C26H51NO8	[M+H]+
506.3706000						
507.2473952	507.2482	0.0008	1.5771	LPA 22:5	C25H41O7PNa	[M+Na]+
	507.2482	0.0008	1.5771	LPA O-22:6;O	C25H41O7PNa	[M+Na]+
507.2473952						
507.2473952	507.2482	0.0008	1.5771	PA O-22:5	C25H41O7PNa	[M+Na]+
	507.2466	0.0008	1.5771	LPS 16:4	C22H36NO9P	[M+NH4]+

507.2473952						
507.2473952	507.2484	0.0010	1.9714	LPA 19:0;O	C22H45O8PK	[M+K]+
	507.2484	0.0010	1.9714	LPG O-16:1	C22H45O8PK	[M+K]+
507.2473952						
508.2479884	508.2493	0.0014	2.7546	NAT 23:4;O	C25H43NO5SK	[M+K]+
	508.2493	0.0014	2.7546	ST 23:1;O3;T	C25H43NO5SK	[M+K]+
508.2479884						
508.3392286	508.3398	0.0005	0.9836	LPC 17:1	C25H50NO7P	[M+H]+
	508.3398	0.0005	0.9836	LPC O-17:2;O	C25H50NO7P	[M+H]+
508.3392286						
508.3392286	508.3398	0.0005	0.9836	LPE 20:1	C25H50NO7P	[M+H]+
	508.3398	0.0005	0.9836	LPE O-20:2;O	C25H50NO7P	[M+H]+
508.3392286						
508.3392286	508.3398	0.0005	0.9836	PE O-20:1	C25H50NO7P	[M+H]+
	508.3398	0.0005	0.9836	LPC 17:0;O	C25H52NO8P	[M+H-H2O]+
508.3392286						
508.3392286	508.3398	0.0005	0.9836	LPE 20:0;O	C25H52NO8P	[M+H-H2O]+
	508.3398	0.0005	0.9836	LPS O-19:0	C25H52NO8P	[M+H-H2O]+
508.3392286						
508.3392286	508.3398	0.0005	0.9836	PE O-20:0;O	C25H52NO8P	[M+H-H2O]+
	508.3397	0.0005	0.9836	CAR 23:6	C30H47NO4Na	[M+Na]+
508.3392286						
508.3392286	508.3397	0.0005	0.9836	NAE 28:7;O2	C30H47NO4Na	[M+Na]+
	508.3397	0.0005	0.9836	ST 28:3;O3;G	C30H47NO4Na	[M+Na]+
508.3392286						
508.3392286	508.3398	0.0005	0.9836	LPA 22:2	C25H47O7P	[M+NH4]+
	508.3398	0.0005	0.9836	LPA O-22:3;O	C25H47O7P	[M+NH4]+
508.3392286						
508.3392286	508.3398	0.0005	0.9836	PA O-22:2	C25H47O7P	[M+NH4]+
	508.3399	0.0006	1.1803	CAR 20:1;O	C27H51NO5K	[M+K]+
508.3392286						
508.3392286	508.3399	0.0006	1.1803	NAE 25:2;O3	C27H51NO5K	[M+K]+
	508.3398	0.0010	1.9672	LPC 17:1	C25H50NO7P	[M+H]+
508.3407506						
508.3407506	508.3398	0.0010	1.9672	LPC O-17:2;O	C25H50NO7P	[M+H]+
	508.3398	0.0010	1.9672	LPE 20:1	C25H50NO7P	[M+H]+
508.3407506						
508.3407506	508.3398	0.0010	1.9672	LPE O-20:2;O	C25H50NO7P	[M+H]+

	508.3398	0.0010	1.9672	PE O-20:1	C25H50NO7P	[M+H]+
508.3407506						
508.3407506	508.3421	0.0014	2.7541	ST 30:6;O3;G	C32H45NO4	[M+H]+
	508.3398	0.0010	1.9672	LPC 17:0;O	C25H52NO8P	[M+H-H2O]+
508.3407506						
508.3407506	508.3398	0.0010	1.9672	LPE 20:0;O	C25H52NO8P	[M+H-H2O]+
	508.3398	0.0010	1.9672	LPS O-19:0	C25H52NO8P	[M+H-H2O]+
508.3407506						
508.3407506	508.3398	0.0010	1.9672	PE O-20:0;O	C25H52NO8P	[M+H-H2O]+
	508.3421	0.0014	2.7541	ST 30:5;O4;G	C32H47NO5	[M+H-H2O]+
508.3407506						
508.3407506	508.3397	0.0010	1.9672	CAR 23:6	C30H47NO4Na	[M+Na]+
	508.3397	0.0010	1.9672	NAE 28:7;O2	C30H47NO4Na	[M+Na]+
508.3407506						
508.3407506	508.3397	0.0010	1.9672	ST 28:3;O3;G	C30H47NO4Na	[M+Na]+
	508.3398	0.0010	1.9672	LPA 22:2	C25H47O7P	[M+NH4]+
508.3407506						
508.3407506	508.3398	0.0010	1.9672	LPA O-22:3;O	C25H47O7P	[M+NH4]+
	508.3398	0.0010	1.9672	PA O-22:2	C25H47O7P	[M+NH4]+
508.3407506						
508.3407506	508.3399	0.0009	1.7705	CAR 20:1;O	C27H51NO5K	[M+K]+
	508.3399	0.0009	1.7705	NAE 25:2;O3	C27H51NO5K	[M+K]+
508.3407506						
509.2653969	509.2663	0.0009	1.7672	LPA 24:6;O	C27H43O8P	[M+H-H2O]+
	509.2663	0.0009	1.7672	PA 24:5	C27H43O8P	[M+H-H2O]+
509.2653969						
509.2653969	509.2663	0.0009	1.7672	PA O-24:6;O	C27H43O8P	[M+H-H2O]+
	509.2664	0.0010	1.9636	DG 26:7	C29H42O5K	[M+K]+
509.2653969						
509.2653969	509.2664	0.0010	1.9636	ST 29:5;O5	C29H42O5K	[M+K]+
	509.264	0.0014	2.7491	LPG O-16:0	C22H47O8PK	[M+K]+
509.2653969						
509.3427213	509.3449	0.0021	4.1229	DG 24:1;O2	C27H50O7Na	[M+Na]+
	509.3408	0.0020	3.9266	NAT 26:6	C28H45NO4S	[M+NH4]+
509.3427213						
516.3055851	516.3061	0.0005	0.9684	LPC 16:1	C24H48NO7PNa	[M+Na]+
	516.3061	0.0005	0.9684	LPC O-16:2;O	C24H48NO7PNa	[M+Na]+
516.3055851						
516.3055851	516.3061	0.0005	0.9684	LPE 19:1	C24H48NO7PNa	[M+Na]+

	516.3061	0.0005	0.9684	LPE O-19:2;O	C24H48NO7PNa	[M+Na]+
516.3055851						
517.2738674	517.2731	0.0008	1.5466	ST 26:7;O3;T	C28H37NO5S	[M+NH4]+
	517.2748	0.0010	1.9332	ST 28:2;O;S	C28H46O4SK	[M+K]+
517.2738674						
518.3123381	518.3122	0.0001	0.1929	NAT 22:0;O3	C24H49NO7SNa	[M+Na]+
	520.3398	0.0005	0.9609	LPC 18:2	C26H50NO7P	[M+H]+
520.3392168						
520.3392168	520.3398	0.0005	0.9609	LPC O-18:3;O	C26H50NO7P	[M+H]+
	520.3398	0.0005	0.9609	LPE 21:2	C26H50NO7P	[M+H]+
520.3392168						
520.3392168	520.3398	0.0005	0.9609	LPE O-21:3;O	C26H50NO7P	[M+H]+
	520.3398	0.0005	0.9609	PE O-21:2	C26H50NO7P	[M+H]+
520.3392168						
520.3392168	520.3398	0.0005	0.9609	LPC 18:1;O	C26H52NO8P	[M+H-H2O]+
	520.3398	0.0005	0.9609	LPE 21:1;O	C26H52NO8P	[M+H-H2O]+
520.3392168						
520.3392168	520.3398	0.0005	0.9609	LPS O-20:1	C26H52NO8P	[M+H-H2O]+
	520.3398	0.0005	0.9609	PE 21:0	C26H52NO8P	[M+H-H2O]+
520.3392168						
520.3392168	520.3398	0.0005	0.9609	PE O-21:1;O	C26H52NO8P	[M+H-H2O]+
	520.3397	0.0005	0.9609	ST 29:4;O3;G	C31H47NO4Na	[M+Na]+
520.3392168						
520.3392168	520.3398	0.0005	0.9609	LPA 23:3	C26H47O7P	[M+NH4]+
	520.3398	0.0005	0.9609	LPA O-23:4;O	C26H47O7P	[M+NH4]+
520.3392168						
520.3392168	520.3398	0.0005	0.9609	PA O-23:3	C26H47O7P	[M+NH4]+
	520.3399	0.0007	1.3453	CAR 21:2;O	C28H51NO5K	[M+K]+
520.3392168						
520.3392168	520.3399	0.0007	1.3453	Cer 28:3;O4	C28H51NO5K	[M+K]+
	520.3399	0.0007	1.3453	NAE 26:3;O3	C28H51NO5K	[M+K]+
520.3392168						
520.3411708	520.3398	0.0014	2.6905	LPC 18:2	C26H50NO7P	[M+H]+
	520.3398	0.0014	2.6905	LPC O-18:3;O	C26H50NO7P	[M+H]+
520.3411708						
520.3411708	520.3398	0.0014	2.6905	LPE 21:2	C26H50NO7P	[M+H]+
	520.3398	0.0014	2.6905	LPE O-21:3;O	C26H50NO7P	[M+H]+

520.3411708						
520.3411708	520.3398	0.0014	2.6905	PE O-21:2	C26H50NO7P	[M+H]+
	520.3398	0.0014	2.6905	LPC 18:1;O	C26H52NO8P	[M+H-H2O]+
520.3411708						
520.3411708	520.3398	0.0014	2.6905	LPE 21:1;O	C26H52NO8P	[M+H-H2O]+
	520.3398	0.0014	2.6905	LPS O-20:1	C26H52NO8P	[M+H-H2O]+
520.3411708						
520.3411708	520.3398	0.0014	2.6905	PE 21:0	C26H52NO8P	[M+H-H2O]+
	520.3398	0.0014	2.6905	PE O-21:1;O	C26H52NO8P	[M+H-H2O]+
520.3411708						
520.3411708	520.3397	0.0014	2.6906	ST 29:4;O3;G	C31H47NO4Na	[M+Na]+
	520.3398	0.0014	2.6905	LPA 23:3	C26H47O7P	[M+NH4]+
520.3411708						
520.3411708	520.3398	0.0014	2.6905	LPA O-23:4;O	C26H47O7P	[M+NH4]+
	520.3398	0.0014	2.6905	PA O-23:3	C26H47O7P	[M+NH4]+
520.3411708						
520.3411708	520.3399	0.0013	2.4984	CAR 21:2;O	C28H51NO5K	[M+K]+
	520.3399	0.0013	2.4984	Cer 28:3;O4	C28H51NO5K	[M+K]+
520.3411708						
520.3411708	520.3399	0.0013	2.4984	NAE 26:3;O3	C28H51NO5K	[M+K]+
	521.3449	0.0022	4.2199	DG 25:2;O2	C28H50O7Na	[M+Na]+
521.3426250						
521.3426250	521.3449	0.0022	4.2199	ST 28:0;O7	C28H50O7Na	[M+Na]+
	521.3408	0.0019	3.6444	NAT 27:7	C29H45NO4S	[M+NH4]+
521.3426250						
522.3549188	522.3554	0.0005	0.9572	LPC 18:1	C26H52NO7P	[M+H]+
	522.3554	0.0005	0.9572	LPC O-18:2;O	C26H52NO7P	[M+H]+
522.3549188						
522.3549188	522.3554	0.0005	0.9572	LPE 21:1	C26H52NO7P	[M+H]+
	522.3554	0.0005	0.9572	LPE O-21:2;O	C26H52NO7P	[M+H]+
522.3549188						
522.3549188	522.3554	0.0005	0.9572	PE O-21:1	C26H52NO7P	[M+H]+
	522.3554	0.0005	0.9572	LPC 18:0;O	C26H54NO8P	[M+H-H2O]+
522.3549188						
522.3549188	522.3554	0.0005	0.9572	LPE 21:0;O	C26H54NO8P	[M+H-H2O]+
	522.3554	0.0005	0.9572	LPS O-20:0	C26H54NO8P	[M+H-H2O]+
522.3549188						
522.3549188	522.3554	0.0005	0.9572	PE O-21:0;O	C26H54NO8P	[M+H-H2O]+

	522.3554	0.0005	0.9572	CAR 24:6	C31H49NO4Na	[M+Na]+
522.3549188						
522.3549188	522.3554	0.0005	0.9572	NAE 29:7;O2	C31H49NO4Na	[M+Na]+
	522.3554	0.0005	0.9572	ST 29:3;O3;G	C31H49NO4Na	[M+Na]+
522.3549188						
522.3549188	522.3554	0.0005	0.9572	LPA 23:2	C26H49O7P	[M+NH4]+
	522.3554	0.0005	0.9572	LPA O-23:3;O	C26H49O7P	[M+NH4]+
522.3549188						
522.3549188	522.3554	0.0005	0.9572	PA O-23:2	C26H49O7P	[M+NH4]+
	522.3555	0.0006	1.1486	CAR 21:1;O	C28H53NO5K	[M+K]+
522.3549188						
522.3549188	522.3555	0.0006	1.1486	Cer 28:2;O4	C28H53NO5K	[M+K]+
	522.3555	0.0006	1.1486	NAE 26:2;O3	C28H53NO5K	[M+K]+
522.3549188						
522.5239147	522.5244	0.0005	0.9569	Cer 34:1;O	C34H67NO2	[M+H]+
	522.5244	0.0005	0.9569	NAE 32:1	C34H67NO2	[M+H]+
522.5239147						
522.5239147	522.5244	0.0005	0.9569	Cer 34:0;O2	C34H69NO3	[M+H-H2O]+
	522.5244	0.0005	0.9569	NAE 32:0;O	C34H69NO3	[M+H-H2O]+
522.5239147						
522.5239147	522.5244	0.0005	0.9569	FA 34:2	C34H64O2	[M+NH4]+
	523.2585	0.0004	0.7644	LPA O-23:5	C26H45O6PK	[M+K]+
523.2581710						
524.1368702	524.1351	0.0018	3.4342	ST 20:4;O7;T	C22H31NO9SK	[M+K]+
	524.3582	0.0005	0.9535	CAR 22:4;O3	C29H49NO7	[M+H]+
524.3587187						
524.3587187	524.3582	0.0005	0.9535	Cer 29:5;O6	C29H49NO7	[M+H]+
	524.3582	0.0005	0.9535	ST 21:0;O2;HexNAc	C29H49NO7	[M+H]+
524.3587187						
524.3587187	524.3582	0.0005	0.9535	ST 27:1;O6;G	C29H49NO7	[M+H]+
	524.3582	0.0005	0.9535	CAR 22:3;O4	C29H51NO8	[M+H-H2O]+
524.3587187						
524.3587187	524.3582	0.0005	0.9535	ST 27:0;O7;G	C29H51NO8	[M+H-H2O]+
	524.3582	0.0005	0.9535	DG 26:5;O2	C29H46O7	[M+NH4]+
524.3587187						
524.3587187	524.3582	0.0005	0.9535	ST 23:1;O;GlcA	C29H46O7	[M+NH4]+
	524.3582	0.0005	0.9535	ST 23:2;O2;Hex	C29H46O7	[M+NH4]+

524.3587187						
524.3587187	524.3582	0.0005	0.9535	ST 29:3;O7	C29H46O7	[M+NH4]+
	524.3615	0.0010	1.9071	NAT 24:0;O3	C26H53NO7S	[M+H]+
524.3625957						
525.3637028	525.3663	0.0026	4.9489	LPC 17:1	C25H50NO7P	[M+NH4]+
	525.3663	0.0026	4.9489	LPC O-17:2;O	C25H50NO7P	[M+NH4]+
525.3637028						
525.3637028	525.3663	0.0026	4.9489	LPE 20:1	C25H50NO7P	[M+NH4]+
	525.3663	0.0026	4.9489	LPE O-20:2;O	C25H50NO7P	[M+NH4]+
525.3637028						
525.3637028	525.3663	0.0026	4.9489	PE O-20:1	C25H50NO7P	[M+NH4]+
	526.2904	0.0019	3.6102	LPC 17:3	C25H46NO7PNa	[M+Na]+
526.2885253						
526.2885253	526.2904	0.0019	3.6102	LPC O-17:4;O	C25H46NO7PNa	[M+Na]+
	526.2904	0.0019	3.6102	LPE 20:3	C25H46NO7PNa	[M+Na]+
526.2885253						
526.2885253	526.2904	0.0019	3.6102	LPE O-20:4;O	C25H46NO7PNa	[M+Na]+
526.2885253	526.2904	0.0019	3.6102	PE O-20:3	C25H46NO7PNa	[M+Na]+
	527.2592	0.0013	2.4656	ST 29:4;O;S	C29H44O4SK	[M+K]+
527.2578473						
1037.6545290	1037.656	0.0015	1.4456	DGDG 45:10	C60H94O15	[M+H-H2O]+
	1037.6537	0.0008	0.7710	DGDG 40:3	C55H98O15K	[M+K]+
1037.6545290						
1038.6576140	1038.656	0.0016	1.5405	PS 52:8	C58H98NO10PK	[M+K]+
	1038.656	0.0016	1.5405	PS O-52:9;O	C58H98NO10PK	[M+K]+
1038.6576140						
1039.5873540	1039.5883	0.0009	0.8657	PIP 42:4;O	C51H92O17P2	[M+H]+
	1039.5882	0.0009	0.8657	PI 47:10;O	C56H89O14PNa	[M+Na]+
1039.5873540						
1039.5873540	1039.5858	0.0015	1.4429	PIP 40:1;O	C49H94O17P2Na	[M+Na]+
	1041.6039	0.0001	0.0960	PIP 42:3;O	C51H94O17P2	[M+H]+
1041.6040450						
1041.6040450	1041.6063	0.0022	2.1121	PI 49:12;O	C58H89O14P	[M+H]+
	1041.6039	0.0002	0.1920	PI 47:9;O	C56H91O14PNa	[M+Na]+
1041.6040450						
1041.6040450	1041.6015	0.0025	2.4002	PIP 40:0;O	C49H96O17P2Na	[M+Na]+
	1041.6234	0.0005	0.4800	MIPC 38:4;O4	C50H90NO18P	[M+NH4]+

1041.6238830						
1044.3288830	1044.329	0.0001	0.0958	CoA 16:0;O	C37H66N7O18P3SNa	[M+Na]+
	1044.3284	0.0005	0.4788	PIP3 30:6;O	C39H66O23P4	[M+NH4]+
1044.3288830						
1044.3317930	1044.3314	0.0004	0.3830	CoA 18:3;O	C39H64N7O18P3S	[M+H]+
1044.3317930	1044.3314	0.0004	0.3830	CoA 18:2;O2	C39H66N7O19P3S	[M+H-H2O]+
	1046.6598	0.0009	0.8599	Hex(3)-Cer 34:1;O2	C52H97NO18Na	[M+Na]+
1046.6589330						
1046.6589330	1046.6574	0.0015	1.4331	SHexCer 47:2;O5	C53H101NO14SK	[M+K]+
1046.6619800	1046.6611	0.0009	0.8599	PC 52:11;O	C60H98NO9PK	[M+K]+
	1046.6611	0.0009	0.8599	PE 55:11;O	C60H98NO9PK	[M+K]+
1046.6619800						
1046.6619800	1046.6611	0.0009	0.8599	PS O-54:11	C60H98NO9PK	[M+K]+
	1046.6692	0.0020	1.9108	PI 49:10	C58H93O13P	[M+NH4]+
1046.6711630						
1046.6711630	1046.6692	0.0020	1.9108	PI O-49:11;O	C58H93O13P	[M+NH4]+
	1046.6727	0.0015	1.4331	SHexCer 51:6;O2	C57H101NO11SK	[M+K]+
1046.6711630						
1047.3574670	1047.362	0.0045	4.2965	PIP3 29:0;O	C38H76O23P4Na	[M+Na]+
	1047.6661	0.0029	2.7681	PG 54:11;O	C60H97O11PNa	[M+Na]+
1047.6631760						
1047.6631760	1047.6662	0.0030	2.8635	PI O-48:6	C57H101O12PK	[M+K]+
	1048.6767	0.0011	1.0489	PC 52:10;O	C60H100NO9PK	[M+K]+
1048.6756290						
1048.6756290	1048.6767	0.0011	1.0489	PE 55:10;O	C60H100NO9PK	[M+K]+
	1048.6767	0.0011	1.0489	PS O-54:10	C60H100NO9PK	[M+K]+
1048.6756290						
1056.6152580	1056.6148	0.0005	0.4732	PIP 42:4;O	C51H92O17P2	[M+NH4]+
	1056.6149	0.0003	0.2839	IPC 47:5;O6	C53H96NO15PK	[M+K]+
1056.6152580						
1057.6187660	1057.6183	0.0005	0.4728	MIPC 38:4;O5	C50H90NO19P	[M+NH4]+
	1057.6224	0.0011	1.0401	DGDG 42:7	C57H94O15K	[M+K]+
1057.6213160						
1058.6275440	1058.6305	0.0029	2.7394	PIP 42:3;O	C51H94O17P2	[M+NH4]+
1058.6275440	1058.6247	0.0029	2.7394	PS 54:12	C60H94NO10PK	[M+K]+
	1058.6306	0.0030	2.8338	IPC 47:4;O6	C53H98NO15PK	[M+K]+

1058.6275440						
1058.6319020	1058.6328	0.0009	0.8502	PI 49:12;O	C58H89O14P	[M+NH4]+
	1058.6305	0.0015	1.4169	PIP 42:3;O	C51H94O17P2	[M+NH4]+
1058.6319020						
1058.6319020	1058.6306	0.0013	1.2280	IPC 47:4;O6	C53H98NO15PK	[M+K]+
	1059.6339	0.0012	1.1325	MIPC 38:3;O5	C50H92NO19P	[M+NH4]+
1059.6351690						
1065.6825490	1065.6809	0.0017	1.5952	MIPC 38:0;O5	C50H98NO19P	[M+NH4]+
	1065.685	0.0025	2.3459	DGDG 42:3	C57H102O15K	[M+K]+
1065.6825490						
1068.5734960	1068.5785	0.0050	4.6791	MIPC 41:6;O2	C53H92NO16PK	[M+K]+
	1072.3603	0.0018	1.6785	CoA 18:0;O	C39H70N7O18P3SNa	[M+Na]+
1072.3585290						
1072.3585290	1072.3597	0.0011	1.0258	PIP3 32:6;O	C41H70O23P4	[M+NH4]+
	1072.3603	0.0005	0.4663	CoA 18:0;O	C39H70N7O18P3SNa	[M+Na]+
1072.3608060						
1072.3608060	1072.3597	0.0011	1.0258	PIP3 32:6;O	C41H70O23P4	[M+NH4]+
	1073.3589	0.0041	3.8198	PIP3 36:8	C45H74O22P4	[M+H-H2O]+
1073.3630230						
1073.3630230	1073.358	0.0051	4.7514	CoA 19:4;O	C40H64N7O18P3S	[M+NH4]+
	1073.3591	0.0040	3.7266	PIP2 38:10	C47H73O19P3K	[M+K]+
1073.3630230						
1075.5319000	1075.5307	0.0012	1.1157	PIP 47:12;O	C56H86O17P2	[M+H-H2O]+
	1079.5984	0.0003	0.2779	PIP 48:9	C57H94O16P2	[M+H-H2O]+
1079.5980980						
1079.5980980	1079.5985	0.0004	0.3705	PI 50:11	C59H93O13PK	[M+K]+
	1079.5985	0.0004	0.3705	PI O-50:12;O	C59H93O13PK	[M+K]+
1079.5980980						
1079.6010630	1079.5984	0.0026	2.4083	PIP 48:9	C57H94O16P2	[M+H-H2O]+
	1079.5985	0.0025	2.3157	PI 50:11	C59H93O13PK	[M+K]+
1079.6010630						
1079.6010630	1079.5985	0.0025	2.3157	PI O-50:12;O	C59H93O13PK	[M+K]+
	1084.6461	0.0033	3.0425	PIP 44:4;O	C53H96O17P2	[M+NH4]+
1084.6428010						
1084.6428010	1084.6462	0.0034	3.1347	IPC 49:5;O6	C55H100NO15PK	[M+K]+
	1085.6454	0.0015	1.3817	PIP 48:6	C57H100O16P2	[M+H-H2O]+
1085.6468260						
1085.6468260	1085.6455	0.0013	1.1974	PI 50:8	C59H99O13PK	[M+K]+

	1085.6455	0.0013	1.1974	PI O-50:9;O	C59H99O13PK	[M+K]+
1085.6468260						
1085.7128920	1085.7135	0.0006	0.5526	DGDG 47:9	C62H100O15	[M+H]+
	1085.7111	0.0018	1.6579	DGDG 45:6	C60H102O15Na	[M+Na]+
1085.7128920						
1086.3433710	1086.342	0.0014	1.2887	CoA 20:4;O2	C41H66N7O19P3S	[M+H]+
	1086.342	0.0014	1.2887	CoA 20:3;O3	C41H68N7O20P3S	[M+H-H2O]+
1086.3433710						
1086.6575390	1086.656	0.0015	1.3804	PS 56:12	C62H98NO10PK	[M+K]+
	1086.6618	0.0010	0.9202	PIP 44:3;O	C53H98O17P2	[M+NH4]+
1086.6607990						
1086.6607990	1086.6619	0.0011	1.0123	IPC 49:4;O6	C55H102NO15PK	[M+K]+
	1087.661	0.0022	2.0227	PIP 48:5	C57H102O16P2	[M+H-H2O]+
1087.6631840						
1087.6631840	1087.6652	0.0021	1.9307	MIPC 40:3;O5	C52H96NO19P	[M+NH4]+
	1087.6611	0.0020	1.8388	PI 50:7	C59H101O13PK	[M+K]+
1087.6631840						
1087.6631840	1087.6611	0.0020	1.8388	PI O-50:8;O	C59H101O13PK	[M+K]+
	1087.7291	0.0008	0.7355	DGDG 47:8	C62H102O15	[M+H]+
1087.7283810						
1087.7283810	1087.7267	0.0016	1.4710	DGDG 45:5	C60H104O15Na	[M+Na]+
	1087.7291	0.0016	1.4710	DGDG 47:8	C62H102O15	[M+H]+
1087.7307240						
1088.7321230	1088.7314	0.0007	0.6430	PS 59:12;O	C65H104NO11P	[M+H-H2O]+
	1092.6724	0.0007	0.6406	MIPC 42:1;O2	C54H104NO16PK	[M+K]+
1092.6717480						
1096.6048580	1096.6098	0.0050	4.5595	MIPC 43:6;O2	C55H96NO16PK	[M+K]+
	1099.688	0.0028	2.5462	SQDG 52:7	C61H104O12SK	[M+K]+
1099.6908430						
1104.6120010	1104.6148	0.0028	2.5348	PIP 46:8;O	C55H92O17P2	[M+NH4]+
	1105.6141	0.0002	0.1809	PIP 50:10	C59H96O16P2	[M+H-H2O]+
1105.6142770						
1105.6142770	1105.6142	0.0001	0.0904	PI 52:12	C61H95O13PK	[M+K]+
	1106.4046	0.0035	3.1634	CoA 21:1;O2	C42H74N7O19P3S	[M+H]+
1106.4080470						
1106.4080470	1106.4046	0.0035	3.1634	CoA 21:0;O3	C42H76N7O20P3S	[M+H-H2O]+
	1107.6274	0.0003	0.2708	PIP2 43:0	C52H103O19P3	[M+H-H2O]+

1107.6276480						
1107.6276480	1107.6275	0.0002	0.1806	PIP 45:2	C54H102O16P2K	[M+K]+
	1107.6297	0.0014	1.2640	PIP 50:9	C59H98O16P2	[M+H-H2O]+
1107.6311370						
1107.6311370	1107.6298	0.0013	1.1737	PI 52:11	C61H97O13PK	[M+K]+
	1107.6298	0.0013	1.1737	PI O-52:12;O	C61H97O13PK	[M+K]+
1107.6311370						
1108.3256700	1108.3263	0.0007	0.6316	CoA 22:6;O3	C43H66N7O20P3S	[M+H-H2O]+
	1108.3239	0.0017	1.5338	CoA 20:4;O2	C41H66N7O19P3SNa	[M+Na]+
1108.3256700						
1108.6409660	1108.6461	0.0051	4.6002	PIP 46:6;O	C55H96O17P2	[M+NH4]+
	1109.6454	0.0016	1.4419	PIP 50:8	C59H100O16P2	[M+H-H2O]+
1109.6438000						
1109.6438000	1109.6431	0.0007	0.6308	PIP 45:1	C54H104O16P2K	[M+K]+
	1109.6455	0.0017	1.5320	PI 52:10	C61H99O13PK	[M+K]+
1109.6438000						
1109.6438000	1109.6455	0.0017	1.5320	PI O-52:11;O	C61H99O13PK	[M+K]+
	1111.6223	0.0037	3.3285	PIP2 42:0	C51H101O19P3	[M+H]+
1111.6185970						
1111.6185970	1111.6222	0.0036	3.2385	PIP 47:6	C56H98O16P2Na	[M+Na]+
	1111.6224	0.0038	3.4184	PIP 44:1;O	C53H102O17P2K	[M+K]+
1111.6185970						
1111.6214080	1111.6223	0.0009	0.8096	PIP2 42:0	C51H101O19P3	[M+H]+
	1111.6222	0.0008	0.7197	PIP 47:6	C56H98O16P2Na	[M+Na]+
1111.6214080						
1111.6214080	1111.6224	0.0010	0.8996	PIP 44:1;O	C53H102O17P2K	[M+K]+
	1112.6257	0.0029	2.6064	MIPC 43:6;O4	C55H96NO18PNa	[M+Na]+
1112.6228660						
1112.6228660	1112.6199	0.0030	2.6963	PIP 48:10	C57H92O16P2	[M+NH4]+
	1112.6259	0.0030	2.6963	MIPC 40:1;O5	C52H100NO19PK	[M+K]+
1112.6228660						
1122.6750480	1122.67	0.0051	4.5427	MIPC 44:5;O5	C56H100NO19P	[M+H]+
	1122.67	0.0051	4.5427	MIPC 44:4;O6	C56H102NO20P	[M+H-H2O]+
1122.6750480						
1122.6750480	1122.6701	0.0049	4.3646	Hex2Cer 46:6;O6	C58H101NO17K	[M+K]+
	1125.7037	0.0016	1.4213	SQDG 54:8	C63H106O12SK	[M+K]+
1125.7052250						
1126.6164720	1126.6204	0.0039	3.4617	MIPC 44:6;O3	C56H98NO17PK	[M+K]+

	1126.6204	0.0005	0.4438	MIPC 44:6;O3	C56H98NO17PK	[M+K]+
1126.6208560						
1127.6181520	1127.6172	0.0010	0.8868	PIP2 42:0;O	C51H101O20P3	[M+H]+
	1127.6196	0.0014	1.2416	PIP 49:9;O	C58H96O17P2	[M+H]+
1127.6181520						
1127.6181520	1127.6171	0.0010	0.8868	PIP 47:6;O	C56H98O17P2Na	[M+Na]+
	1127.6196	0.0021	1.8623	PIP 49:9;O	C58H96O17P2	[M+H]+
1127.6216960						
1127.7190800	1127.7193	0.0002	0.1773	SQDG 54:7	C63H108O12SK	[M+K]+
	1127.7193	0.0020	1.7735	SQDG 54:7	C63H108O12SK	[M+K]+
1127.7212950						
1128.7243750	1128.7239	0.0005	0.4430	PS 59:12;O	C65H104NO11PNa	[M+Na]+
	1130.7478	0.0009	0.7959	MIPC 48:5;O2	C60H108NO16P	[M+H]+
1130.7487030						
1130.7487030	1130.7478	0.0009	0.7959	MIPC 48:4;O3	C60H110NO17P	[M+H-H2O]+
	1130.748	0.0007	0.6191	Hex2Cer 50:6;O3	C62H109NO14K	[M+K]+
1130.7487030						
1130.7509800	1130.7513	0.0004	0.3537	SHexCer 53:2;O5	C59H113NO14SK	[M+K]+
	1132.7424	0.0021	1.8539	PI 54:10;O	C63H103O14P	[M+NH4]+
1132.7445120						
1143.5926240	1143.591	0.0017	1.4865	PIP2 45:5	C54H97O19P3	[M+H]+
	1143.591	0.0017	1.4865	PIP2 45:4;O	C54H99O20P3	[M+H-H2O]+
1143.5926240						
1143.5926240	1143.5909	0.0017	1.4865	PIP 50:11	C59H94O16P2Na	[M+Na]+
	1143.5911	0.0015	1.3117	PIP 47:6;O	C56H98O17P2K	[M+K]+
1143.5926240						
1144.5972250	1144.5946	0.0027	2.3589	MIPC 43:6;O5	C55H96NO19PK	[M+K]+
	1145.6066	0.0003	0.2619	PIP2 45:4	C54H99O19P3	[M+H]+
1145.6069340						
1145.6069340	1145.6066	0.0003	0.2619	PIP2 45:3;O	C54H101O20P3	[M+H-H2O]+
	1145.6066	0.0004	0.3492	PIP 50:10	C59H96O16P2Na	[M+Na]+
1145.6069340						
1145.6069340	1145.6067	0.0002	0.1746	PIP 47:5;O	C56H100O17P2K	[M+K]+
	1145.609	0.0025	2.1822	PIP 52:12;O	C61H96O17P2	[M+H-H2O]+
1145.6114480						
1147.6866410	1147.688	0.0014	1.2198	SQDG 56:11	C65H104O12SK	[M+K]+
	1148.3188	0.0015	1.3063	CoA 22:6;O3	C43H66N7O20P3SNa	[M+Na]+
1148.3173350						
1148.3173350	1148.319	0.0017	1.4804	CoA 19:1;O4	C40H70N7O21P3SK	[M+K]+

	1149.6978	0.0011	0.9568	PIP 50:5;O	C59H106O17P2	[M+H]+
1149.6989040						
1149.6989040	1149.6978	0.0011	0.9568	PI 55:11;O	C64H103O14PNa	[M+Na]+
	1149.7037	0.0010	0.8698	SQDG 56:10	C65H106O12SK	[M+K]+
1149.7026100						
1150.6028860	1150.6051	0.0023	1.9989	MIPC 42:4;O6	C54H98NO20PK	[M+K]+
	1150.7013	0.0047	4.0845	MIPC 46:5;O5	C58H104NO19P	[M+H]+
1150.7060390						
1150.7060390	1150.7013	0.0047	4.0845	MIPC 46:4;O6	C58H106NO20P	[M+H-H2O]+
	1150.7014	0.0046	3.9976	Hex2Cer 48:6;O6	C60H105NO17K	[M+K]+
1150.7060390						
1151.6116850	1151.6172	0.0055	4.7759	PIP2 44:2;O	C53H101O20P3	[M+H]+
	1151.6171	0.0055	4.7759	PIP 49:8;O	C58H98O17P2Na	[M+Na]+
1151.6116850						
1152.6148730	1152.6148	0.0001	0.0868	PIP 50:12;O	C59H92O17P2	[M+NH4]+
	1153.6328	0.0007	0.6068	PIP2 44:1;O	C53H103O20P3	[M+H]+
1153.6335770						
1153.6335770	1153.6352	0.0016	1.3869	PIP 51:10;O	C60H98O17P2	[M+H]+
	1153.6328	0.0008	0.6935	PIP 49:7;O	C58H100O17P2Na	[M+Na]+
1153.6335770						
1154.6468740	1154.6517	0.0048	4.1571	MIPC 46:6;O3	C58H102NO17PK	[M+K]+
	1154.6517	0.0007	0.6062	MIPC 46:6;O3	C58H102NO17PK	[M+K]+
1154.6510330						
1155.6483060	1155.6485	0.0002	0.1731	PIP2 44:0;O	C53H105O20P3	[M+H]+
	1155.6484	0.0001	0.0865	PIP 49:6;O	C58H102O17P2Na	[M+Na]+
1155.6483060						
1155.6520550	1155.6509	0.0012	1.0384	PIP 51:9;O	C60H100O17P2	[M+H]+
	1156.7635	0.0018	1.5561	MIPC 50:6;O2	C62H110NO16P	[M+H]+
1156.7652930						
1156.7652930	1156.7635	0.0018	1.5561	MIPC 50:5;O3	C62H112NO17P	[M+H-H2O]+
	1156.7693	0.0040	3.4579	Hex(3)-Cer 42:2;O2	C60H111NO18Na	[M+Na]+
1156.7652930						
1156.7652930	1156.7611	0.0042	3.6308	MIPC 48:3;O2	C60H112NO16PNa	[M+Na]+
	1156.7706	0.0053	4.5817	PC 60:12;O	C68H112NO9PK	[M+K]+
1156.7652930						
1156.7652930	1156.7706	0.0053	4.5817	PE 63:12;O	C68H112NO9PK	[M+K]+
	1156.7706	0.0053	4.5817	PS O-62:12	C68H112NO9PK	[M+K]+

1156.7652930						
1158.7791260	1158.7791	0.0000	0.0000	MIPC 50:5;O2	C62H112NO16P	[M+H]+
	1158.7791	0.0000	0.0000	MIPC 50:4;O3	C62H114NO17P	[M+H-H2O]+
1158.7791260						
1158.7791260	1158.7793	0.0001	0.0863	Hex2Cer 52:6;O3	C64H113NO14K	[M+K]+
	1158.7791	0.0023	1.9848	MIPC 50:5;O2	C62H112NO16P	[M+H]+
1158.7814640						
1158.7814640	1158.7791	0.0023	1.9848	MIPC 50:4;O3	C62H114NO17P	[M+H-H2O]+
	1158.7793	0.0022	1.8985	Hex2Cer 52:6;O3	C64H113NO14K	[M+K]+
1158.7814640						
1159.7844130	1159.7842	0.0003	0.2587	SQDG 61:12	C70H112O12S	[M+H-H2O]+
	1174.5968	0.0001	0.0851	PIP2 45:6;O	C54H95O20P3	[M+NH4]+
1174.5966460						
1189.5957730	1189.6007	0.0049	4.1190	M(IP)2C 32:1;O3	C50H95NO25P2	[M+NH4]+
	530.3217	0.0005	0.9428	LPC 17:1	C25H50NO7PNa	[M+Na]+
530.3211705						
530.3211705	530.3217	0.0005	0.9428	LPC O-17:2;O	C25H50NO7PNa	[M+Na]+
	530.3217	0.0005	0.9428	LPE 20:1	C25H50NO7PNa	[M+Na]+
530.3211705						
530.3211705	530.3217	0.0005	0.9428	LPE O-20:2;O	C25H50NO7PNa	[M+Na]+
	530.3217	0.0005	0.9428	PE O-20:1	C25H50NO7PNa	[M+Na]+
530.3211705						
531.2918413	531.2929	0.0010	1.8822	LPI O-14:0	C23H47O11P	[M+H]+
	531.2928	0.0010	1.8822	ST 22:1;O2;GlcA	C28H44O8Na	[M+Na]+
531.2918413						
531.2918413	531.2928	0.0010	1.8822	ST 22:2;O3;Hex	C28H44O8Na	[M+Na]+
	531.2928	0.0010	1.8822	ST 28:3;O8	C28H44O8Na	[M+Na]+
531.2918413						
531.2918413	531.2905	0.0014	2.6351	ST 29:2;O;S	C29H48O4SK	[M+K]+
	532.2493	0.0013	2.4425	NAT 25:6;O	C27H43NO5SK	[M+K]+
532.2480776						
532.2480776	532.2493	0.0013	2.4425	ST 25:3;O3;T	C27H43NO5SK	[M+K]+
	532.3374	0.0005	0.9393	LPC 17:0	C25H52NO7PNa	[M+Na]+
532.3368805						
532.3368805	532.3374	0.0005	0.9393	LPC O-17:1;O	C25H52NO7PNa	[M+Na]+
532.3368805	532.3374	0.0005	0.9393	LPE 20:0	C25H52NO7PNa	[M+Na]+
	532.3374	0.0005	0.9393	LPE O-20:1;O	C25H52NO7PNa	[M+Na]+

532.3368805						
532.3368805	532.3374	0.0005	0.9393	PE O-20:0	C25H52NO7PNa	[M+Na]+
	533.3044	0.0009	1.6876	ST 27:6;O3;T	C29H41NO5S	[M+NH4]+
533.3052728						
533.3052728	533.3061	0.0009	1.6876	ST 29:1;O;S	C29H50O4SK	[M+K]+
	534.1768	0.0018	3.3697	ST 22:5;O7;T	C24H33NO9SNa	[M+Na]+
534.1786152						
534.1786152	534.177	0.0016	2.9953	ST 19:0;O8;T	C21H37NO10SK	[M+K]+
	536.3711	0.0013	2.4237	LPC 19:1	C27H54NO7P	[M+H]+
536.3697517						
536.3697517	536.3711	0.0013	2.4237	LPC O-19:2;O	C27H54NO7P	[M+H]+
	536.3711	0.0013	2.4237	LPE 22:1	C27H54NO7P	[M+H]+
536.3697517						
536.3697517	536.3711	0.0013	2.4237	LPE O-22:2;O	C27H54NO7P	[M+H]+
	536.3711	0.0013	2.4237	PE O-22:1	C27H54NO7P	[M+H]+
536.3697517						
536.3697517	536.3711	0.0013	2.4237	LPC 19:0;O	C27H56NO8P	[M+H-H2O]+
	536.3711	0.0013	2.4237	LPE 22:0;O	C27H56NO8P	[M+H-H2O]+
536.3697517						
536.3697517	536.3711	0.0013	2.4237	LPS O-21:0	C27H56NO8P	[M+H-H2O]+
	536.3711	0.0013	2.4237	PE O-22:0;O	C27H56NO8P	[M+H-H2O]+
536.3697517						
536.3697517	536.371	0.0013	2.4237	CAR 25:6	C32H51NO4Na	[M+Na]+
	536.371	0.0013	2.4237	NAE 30:7;O2	C32H51NO4Na	[M+Na]+
536.3697517						
536.3697517	536.371	0.0013	2.4237	ST 30:3;O3;G	C32H51NO4Na	[M+Na]+
	536.3711	0.0013	2.4237	LPA 24:2	C27H51O7P	[M+NH4]+
536.3697517						
536.3697517	536.3711	0.0013	2.4237	LPA O-24:3;O	C27H51O7P	[M+NH4]+
	536.3711	0.0013	2.4237	PA O-24:2	C27H51O7P	[M+NH4]+
536.3697517						
536.3697517	536.3712	0.0014	2.6101	CAR 22:1;O	C29H55NO5K	[M+K]+
	536.3712	0.0014	2.6101	Cer 29:2;O4	C29H55NO5K	[M+K]+
536.3697517						
536.3697517	536.3712	0.0014	2.6101	NAE 27:2;O3	C29H55NO5K	[M+K]+
	536.3711	0.0004	0.7458	LPC 19:1	C27H54NO7P	[M+H]+
536.3706216						
536.3706216	536.3711	0.0004	0.7458	LPC O-19:2;O	C27H54NO7P	[M+H]+

	536.3711	0.0004	0.7458	LPE 22:1	C27H54NO7P	[M+H]+
536.3706216						
536.3706216	536.3711	0.0004	0.7458	LPE O-22:2;O	C27H54NO7P	[M+H]+
	536.3711	0.0004	0.7458	PE O-22:1	C27H54NO7P	[M+H]+
536.3706216						
536.3706216	536.3711	0.0004	0.7458	LPC 19:0;O	C27H56NO8P	[M+H-H2O]+
	536.3711	0.0004	0.7458	LPE 22:0;O	C27H56NO8P	[M+H-H2O]+
536.3706216						
536.3706216	536.3711	0.0004	0.7458	LPS O-21:0	C27H56NO8P	[M+H-H2O]+
	536.3711	0.0004	0.7458	PE O-22:0;O	C27H56NO8P	[M+H-H2O]+
536.3706216						
536.3706216	536.371	0.0004	0.7458	CAR 25:6	C32H51NO4Na	[M+Na]+
	536.371	0.0004	0.7458	NAE 30:7;O2	C32H51NO4Na	[M+Na]+
536.3706216						
536.3706216	536.371	0.0004	0.7458	ST 30:3;O3;G	C32H51NO4Na	[M+Na]+
	536.3711	0.0004	0.7458	LPA 24:2	C27H51O7P	[M+NH4]+
536.3706216						
536.3706216	536.3711	0.0004	0.7458	LPA O-24:3;O	C27H51O7P	[M+NH4]+
	536.3711	0.0004	0.7458	PA O-24:2	C27H51O7P	[M+NH4]+
536.3706216						
536.3706216	536.3712	0.0006	1.1186	CAR 22:1;O	C29H55NO5K	[M+K]+
	536.3712	0.0006	1.1186	Cer 29:2;O4	C29H55NO5K	[M+K]+
536.3706216						
536.3706216	536.3712	0.0006	1.1186	NAE 27:2;O3	C29H55NO5K	[M+K]+
	540.3061	0.0004	0.7403	LPC 18:3	C26H48NO7PNa	[M+Na]+
540.3056555						
540.3056555	540.3061	0.0004	0.7403	LPC O-18:4;O	C26H48NO7PNa	[M+Na]+
	540.3061	0.0004	0.7403	LPE 21:3	C26H48NO7PNa	[M+Na]+
540.3056555						
540.3056555	540.3061	0.0004	0.7403	LPE O-21:4;O	C26H48NO7PNa	[M+Na]+
	540.3061	0.0004	0.7403	PE O-21:3	C26H48NO7PNa	[M+Na]+
540.3056555						
546.2639560	546.265	0.0010	1.8306	NAT 26:6;O	C28H45NO5SK	[M+K]+
	546.265	0.0010	1.8306	ST 26:3;O3;T	C28H45NO5SK	[M+K]+
546.2639560						
546.2950686	546.2955	0.0004	0.7322	CerP 28:6;O2	C28H46NO6PNa	[M+Na]+
	546.2956	0.0006	1.0983	LPC 17:1	C25H50NO7PK	[M+K]+

546.2950686						
546.2950686	546.2956	0.0006	1.0983	LPC O-17:2;O	C25H50NO7PK	[M+K]+
	546.2956	0.0006	1.0983	LPE 20:1	C25H50NO7PK	[M+K]+
546.2950686						
546.2950686	546.2956	0.0006	1.0983	LPE O-20:2;O	C25H50NO7PK	[M+K]+
	546.2956	0.0006	1.0983	PE O-20:1	C25H50NO7PK	[M+K]+
546.2950686						
546.3434303	546.3435	0.0001	0.1830	NAT 24:0;O3	C26H53NO7SNa	[M+Na]+
	549.2629	0.0011	2.0027	ST 26:7;O5;T	C28H37NO7S	[M+NH4]+
549.2640042						
549.2640042	549.2647	0.0007	1.2744	ST 28:2;O3;S	C28H46O6SK	[M+K]+
	550.1717	0.0020	3.6352	ST 22:5;O8;T	C24H33NO10SNa	[M+Na]+
550.1737687						
550.3861708	550.3867	0.0005	0.9085	CerP 28:1;O3	C28H56NO7P	[M+H]+
	550.3867	0.0005	0.9085	LPC 20:1	C28H56NO7P	[M+H]+
550.3861708						
550.3861708	550.3867	0.0005	0.9085	LPC O-20:2;O	C28H56NO7P	[M+H]+
	550.3867	0.0005	0.9085	LPE 23:1	C28H56NO7P	[M+H]+
550.3861708						
550.3861708	550.3867	0.0005	0.9085	LPE O-23:2;O	C28H56NO7P	[M+H]+
	550.3867	0.0005	0.9085	PC O-20:1	C28H56NO7P	[M+H]+
550.3861708						
550.3861708	550.3867	0.0005	0.9085	PE O-23:1	C28H56NO7P	[M+H]+
	550.3867	0.0005	0.9085	CerP 28:0;O4	C28H58NO8P	[M+H-H2O]+
550.3861708						
550.3861708	550.3867	0.0005	0.9085	LPC 20:0;O	C28H58NO8P	[M+H-H2O]+
	550.3867	0.0005	0.9085	LPE 23:0;O	C28H58NO8P	[M+H-H2O]+
550.3861708						
550.3861708	550.3867	0.0005	0.9085	LPS O-22:0	C28H58NO8P	[M+H-H2O]+
	550.3867	0.0005	0.9085	PC O-20:0;O	C28H58NO8P	[M+H-H2O]+
550.3861708						
550.3861708	550.3867	0.0005	0.9085	PE O-23:0;O	C28H58NO8P	[M+H-H2O]+
	550.3867	0.0005	0.9085	CAR 26:6	C33H53NO4Na	[M+Na]+
550.3861708						
550.3861708	550.3867	0.0005	0.9085	NAE 31:7;O2	C33H53NO4Na	[M+Na]+
	550.3867	0.0005	0.9085	LPA 25:2	C28H53O7P	[M+NH4]+
550.3861708						
550.3861708	550.3867	0.0005	0.9085	LPA O-25:3;O	C28H53O7P	[M+NH4]+

	550.3867	0.0005	0.9085	PA O-25:2	C28H53O7P	[M+NH4]+
550.3861708						
550.3861708	550.3868	0.0007	1.2718	CAR 23:1;O	C30H57NO5K	[M+K]+
	550.3868	0.0007	1.2718	Cer 30:2;O4	C30H57NO5K	[M+K]+
550.3861708						
550.3861708	550.3868	0.0007	1.2718	NAE 28:2;O3	C30H57NO5K	[M+K]+
	556.3374	0.0002	0.3595	LPC 19:2	C27H52NO7PNa	[M+Na]+
556.3371745						
556.3371745	556.3374	0.0002	0.3595	LPC O-19:3;O	C27H52NO7PNa	[M+Na]+
	556.3374	0.0002	0.3595	LPE 22:2	C27H52NO7PNa	[M+Na]+
556.3371745						
556.3371745	556.3374	0.0002	0.3595	LPE O-22:3;O	C27H52NO7PNa	[M+Na]+
	556.3374	0.0002	0.3595	PE O-22:2	C27H52NO7PNa	[M+Na]+
556.3371745						
558.2954268	558.2956	0.0002	0.3582	LPC 18:2	C26H50NO7PK	[M+K]+
	558.2956	0.0002	0.3582	LPC O-18:3;O	C26H50NO7PK	[M+K]+
558.2954268						
558.2954268	558.2956	0.0002	0.3582	LPE 21:2	C26H50NO7PK	[M+K]+
	558.2956	0.0002	0.3582	LPE O-21:3;O	C26H50NO7PK	[M+K]+
558.2954268						
558.2954268	558.2956	0.0002	0.3582	PE O-21:2	C26H50NO7PK	[M+K]+
	559.3006	0.0020	3.5759	LPG 20:2	C26H49O9PNa	[M+Na]+
559.2986433						
559.2986433	559.3006	0.0020	3.5759	LPG O-20:3;O	C26H49O9PNa	[M+Na]+
	559.3006	0.0020	3.5759	PA 23:1;O	C26H49O9PNa	[M+Na]+
559.2986433						
559.2986433	559.3006	0.0020	3.5759	PG O-20:2	C26H49O9PNa	[M+Na]+
	564.3061	0.0002	0.3544	CerP 28:5;O3	C28H48NO7PNa	[M+Na]+
564.3058250						
564.3058250	564.3061	0.0002	0.3544	LPC 20:5	C28H48NO7PNa	[M+Na]+
	564.3061	0.0002	0.3544	LPE 23:5	C28H48NO7PNa	[M+Na]+
564.3058250						
564.3058250	564.3061	0.0002	0.3544	LPE O-23:6;O	C28H48NO7PNa	[M+Na]+
	564.3061	0.0002	0.3544	PC O-20:5	C28H48NO7PNa	[M+Na]+
564.3058250						
564.3058250	564.3061	0.0002	0.3544	PE O-23:5	C28H48NO7PNa	[M+Na]+
	564.3062	0.0004	0.7088	LPC 17:0;O	C25H52NO8PK	[M+K]+
564.3058250						
564.3058250	564.3062	0.0004	0.7088	LPE 20:0;O	C25H52NO8PK	[M+K]+

	564.3062	0.0004	0.7088	LPS O-19:0	C25H52NO8PK	[M+K]+
564.3058250						
564.3058250	564.3062	0.0004	0.7088	PE O-20:0;O	C25H52NO8PK	[M+K]+
	565.3112	0.0021	3.7148	LPG 19:0;O	C25H51O10PNa	[M+Na]+
565.3090615						
565.3114035	565.3112	0.0002	0.3538	LPG 19:0;O	C25H51O10PNa	[M+Na]+
	565.312	0.0005	0.8845	ST 21:4;O4;HexNAc	C29H41NO9	[M+NH4]+
565.3114035						
565.3114035	565.312	0.0005	0.8845	ST 27:5;O8;G	C29H41NO9	[M+NH4]+
	565.5666	0.0001	0.1768	Cer 36:2;O	C36H69NO2	[M+NH4]+
565.5665116						
565.5665116	565.5666	0.0001	0.1768	NAE 34:2	C36H69NO2	[M+NH4]+
	566.3122	0.0005	0.8829	NAT 26:4;O3	C28H49NO7SNa	[M+Na]+
566.3127378						
566.3127378	566.3122	0.0005	0.8829	ST 26:1;O5;T	C28H49NO7SNa	[M+Na]+
566.3215387	566.3217	0.0002	0.3532	CerP 28:4;O3	C28H50NO7PNa	[M+Na]+
	566.3217	0.0002	0.3532	LPC 20:4	C28H50NO7PNa	[M+Na]+
566.3215387						
566.3215387	566.3217	0.0002	0.3532	LPC O-20:5;O	C28H50NO7PNa	[M+Na]+
	566.3217	0.0002	0.3532	LPE 23:4	C28H50NO7PNa	[M+Na]+
566.3215387						
566.3215387	566.3217	0.0002	0.3532	LPE O-23:5;O	C28H50NO7PNa	[M+Na]+
	566.3217	0.0002	0.3532	PC O-20:4	C28H50NO7PNa	[M+Na]+
566.3215387						
566.3215387	566.3217	0.0002	0.3532	PE O-23:4	C28H50NO7PNa	[M+Na]+
	567.233	0.0001	0.1763	BMP 20:5	C26H41O10PNa	[M+Na]+
567.2330651						
567.2330651	567.233	0.0001	0.1763	PG 20:5	C26H41O10PNa	[M+Na]+
	567.2331	0.0000	0.0000	BMP 17:0;O	C23H45O11PK	[M+K]+
567.2330651						
567.2330651	567.2331	0.0000	0.0000	LPI O-14:1	C23H45O11PK	[M+K]+
	567.3276	0.0027	4.7592	ST 21:3;O4;HexNAc	C29H43NO9	[M+NH4]+
567.3248770						
567.3248770	567.3276	0.0027	4.7592	ST 27:4;O8;G	C29H43NO9	[M+NH4]+
568.3274467	568.3269	0.0006	1.0557	ST 25:6;O2;HexNAc	C33H45NO7	[M+H]+

	568.3269	0.0006	1.0557	ST 25:5;O3;HexNAc	C33H47NO8	[M+H-H2O]+
568.3274467						
568.3274467	568.3278	0.0004	0.7038	NAT 26:3;O3	C28H51NO7SNa	[M+Na]+
	568.3278	0.0004	0.7038	ST 26:0;O5;T	C28H51NO7SNa	[M+Na]+
568.3274467						
568.3274467	568.3269	0.0006	1.0557	ST 27:7;O;GlcA	C33H42O7	[M+NH4]+
568.3376904	568.3374	0.0003	0.5279	CerP 28:3;O3	C28H52NO7PNa	[M+Na]+
	568.3374	0.0003	0.5279	LPC 20:3	C28H52NO7PNa	[M+Na]+
568.3376904						
568.3376904	568.3374	0.0003	0.5279	LPC O-20:4;O	C28H52NO7PNa	[M+Na]+
	568.3374	0.0003	0.5279	LPE 23:3	C28H52NO7PNa	[M+Na]+
568.3376904						
568.3376904	568.3374	0.0003	0.5279	LPE O-23:4;O	C28H52NO7PNa	[M+Na]+
	568.3374	0.0003	0.5279	PC O-20:3	C28H52NO7PNa	[M+Na]+
568.3376904						
568.3376904	568.3374	0.0003	0.5279	PE O-23:3	C28H52NO7PNa	[M+Na]+
569.3380404	569.3368	0.0013	2.2834	LPA O-26:3	C29H55O6PK	[M+K]+
569.3412380	569.3433	0.0020	3.5128	ST 21:2;O4;HexNAc	C29H45NO9	[M+NH4]+
	569.3433	0.0020	3.5128	ST 27:3;O8;G	C29H45NO9	[M+NH4]+
569.3412380						
569.3683506	569.3684	0.0001	0.1756	ST 25:0;O3;GlcA	C31H52O9	[M+H]+
	569.3684	0.0001	0.1756	ST 25:1;O4;Hex	C31H52O9	[M+H]+
569.3683506						
569.3683506	569.3684	0.0001	0.1756	MGDG 22:2	C31H54O10	[M+H-H2O]+
	569.3684	0.0001	0.1756	ST 25:0;O5;Hex	C31H54O10	[M+H-H2O]+
569.3683506						
570.3545328	570.3554	0.0009	1.5780	CerP 30:5;O3	C30H52NO7P	[M+H]+
	570.3554	0.0009	1.5780	LPC 22:5	C30H52NO7P	[M+H]+
570.3545328						
570.3545328	570.3554	0.0009	1.5780	LPC O-22:6;O	C30H52NO7P	[M+H]+
	570.3554	0.0009	1.5780	LPE 25:5	C30H52NO7P	[M+H]+
570.3545328						
570.3545328	570.3554	0.0009	1.5780	LPE O-25:6;O	C30H52NO7P	[M+H]+
	570.3554	0.0009	1.5780	PC O-22:5	C30H52NO7P	[M+H]+
570.3545328						
570.3545328	570.3554	0.0009	1.5780	PE O-25:5	C30H52NO7P	[M+H]+

	570.3554	0.0009	1.5780	CerP 30:4;O4	C30H54NO8P	[M+H-H2O]+
570.3545328						
570.3545328	570.3554	0.0009	1.5780	LPC 22:4;O	C30H54NO8P	[M+H-H2O]+
	570.3554	0.0009	1.5780	LPE 25:4;O	C30H54NO8P	[M+H-H2O]+
570.3545328						
570.3545328	570.3554	0.0009	1.5780	LPS O-24:4	C30H54NO8P	[M+H-H2O]+
	570.3554	0.0009	1.5780	PC 22:3	C30H54NO8P	[M+H-H2O]+
570.3545328						
570.3545328	570.3554	0.0009	1.5780	PC O-22:4;O	C30H54NO8P	[M+H-H2O]+
	570.3554	0.0009	1.5780	PE 25:3	C30H54NO8P	[M+H-H2O]+
570.3545328						
570.3545328	570.3554	0.0009	1.5780	PE O-25:4;O	C30H54NO8P	[M+H-H2O]+
	570.3554	0.0009	1.5780	LPA 27:6	C30H49O7P	[M+NH4]+
570.3545328						
570.3545328	570.3554	0.0009	1.5780	LPA O-27:7;O	C30H49O7P	[M+NH4]+
	570.3554	0.0009	1.5780	PA O-27:6	C30H49O7P	[M+NH4]+
570.3545328						
570.3545328	570.3555	0.0010	1.7533	CAR 25:5;O	C32H53NO5K	[M+K]+
	570.3555	0.0010	1.7533	Cer 32:6;O4	C32H53NO5K	[M+K]+
570.3545328						
570.3545328	570.3555	0.0010	1.7533	NAE 30:6;O3	C32H53NO5K	[M+K]+
	570.3555	0.0010	1.7533	ST 30:2;O4;G	C32H53NO5K	[M+K]+
570.3545328						
572.3685687	572.3687	0.0001	0.1747	CerP 28:1;O3	C28H56NO7PNa	[M+Na]+
	572.3687	0.0001	0.1747	LPC 20:1	C28H56NO7PNa	[M+Na]+
572.3685687						
572.3685687	572.3687	0.0001	0.1747	LPC O-20:2;O	C28H56NO7PNa	[M+Na]+
	572.3687	0.0001	0.1747	LPE 23:1	C28H56NO7PNa	[M+Na]+
572.3685687						
572.3685687	572.3687	0.0001	0.1747	LPE O-23:2;O	C28H56NO7PNa	[M+Na]+
	572.3687	0.0001	0.1747	PC O-20:1	C28H56NO7PNa	[M+Na]+
572.3685687						
572.3685687	572.3687	0.0001	0.1747	PE O-23:1	C28H56NO7PNa	[M+Na]+
	576.3272	0.0000	0.0000	LPS 20:0	C26H52NO9PNa	[M+Na]+
576.3271616						
576.3271616	576.3272	0.0000	0.0000	LPS O-20:1;O	C26H52NO9PNa	[M+Na]+
	576.3272	0.0000	0.0000	PE 21:0;O	C26H52NO9PNa	[M+Na]+

576.3271616						
576.3271616	576.3272	0.0000	0.0000	PS O-20:0	C26H52NO9PNa	[M+Na]+
	577.5554	0.0002	0.3463	DG O-35:1	C38H74O4	[M+H-H2O]+
577.5552615						
583.3843688	583.3841	0.0003	0.5142	ST 26:0;O3;GlcA	C32H54O9	[M+H]+
	583.3841	0.0003	0.5142	ST 26:1;O4;Hex	C32H54O9	[M+H]+
583.3843688						
583.3843688	583.3841	0.0003	0.5142	MGDG 23:2	C32H56O10	[M+H-H2O]+
	583.3841	0.0003	0.5142	ST 26:0;O5;Hex	C32H56O10	[M+H-H2O]+
583.3843688						
583.5082923	583.5085	0.0002	0.3428	CE 12:1;O	C39H66O3	[M+H]+
	583.5085	0.0002	0.3428	CE 12:0;O2	C39H68O4	[M+H-H2O]+
583.5082923						
583.5082923	583.5085	0.0002	0.3428	DG O-36:5	C39H68O4	[M+H-H2O]+
	584.2959	0.0022	3.7652	LPS 21:3	C27H48NO9PNa	[M+Na]+
584.2937095						
584.2937095	584.2959	0.0022	3.7652	LPS O-21:4;O	C27H48NO9PNa	[M+Na]+
	584.2959	0.0022	3.7652	PE 22:3;O	C27H48NO9PNa	[M+Na]+
584.2937095						
584.2937095	584.2959	0.0022	3.7652	PS O-21:3	C27H48NO9PNa	[M+Na]+
	585.5241	0.0002	0.3416	CE 12:0;O	C39H68O3	[M+H]+
585.5238681						
585.5238681	585.5241	0.0002	0.3416	DG O-36:4	C39H70O4	[M+H-H2O]+
	587.5398	0.0006	1.0212	DG O-36:3	C39H72O4	[M+H-H2O]+
587.5392124						
588.3122104	588.3119	0.0003	0.5099	NAT 29:6;O	C31H51NO5SK	[M+K]+
	588.3119	0.0003	0.5099	ST 29:3;O3;T	C31H51NO5SK	[M+K]+
588.3122104						
589.5552125	589.5554	0.0002	0.3392	DG O-36:2	C39H74O4	[M+H-H2O]+
	590.3217	0.0001	0.1694	CerP 30:6;O3	C30H50NO7PNa	[M+Na]+
590.3215582						
590.3215582	590.3217	0.0001	0.1694	LPC 22:6	C30H50NO7PNa	[M+Na]+
	590.3217	0.0001	0.1694	LPE 25:6	C30H50NO7PNa	[M+Na]+
590.3215582						
590.3215582	590.3217	0.0001	0.1694	LPE O-25:7;O	C30H50NO7PNa	[M+Na]+
	590.3217	0.0001	0.1694	PC O-22:6	C30H50NO7PNa	[M+Na]+
590.3215582						
590.3215582	590.3217	0.0001	0.1694	PE O-25:6	C30H50NO7PNa	[M+Na]+

590.3215582	590.3219	0.0003	0.5082	LPC 19:1;O	C27H54NO8PK	[M+K]+
590.3215582	590.3219	0.0003	0.5082	LPE 22:1;O	C27H54NO8PK	[M+K]+
590.3215582	590.3219	0.0003	0.5082	LPS O-21:1	C27H54NO8PK	[M+K]+
590.3215582	590.3219	0.0003	0.5082	PE 22:0	C27H54NO8PK	[M+K]+
590.3215582	590.3219	0.0003	0.5082	PE O-22:1;O	C27H54NO8PK	[M+K]+
592.3371033	592.3374	0.0003	0.5065	CerP 30:5;O3	C30H52NO7PNa	[M+Na]+
592.3371033	592.3374	0.0003	0.5065	LPC 22:5	C30H52NO7PNa	[M+Na]+
592.3371033	592.3374	0.0003	0.5065	LPC O-22:6;O	C30H52NO7PNa	[M+Na]+
592.3371033	592.3374	0.0003	0.5065	LPE 25:5	C30H52NO7PNa	[M+Na]+
592.3371033	592.3374	0.0003	0.5065	LPE O-25:6;O	C30H52NO7PNa	[M+Na]+
592.3371033	592.3374	0.0003	0.5065	PC O-22:5	C30H52NO7PNa	[M+Na]+
592.3371033	592.3374	0.0003	0.5065	PE O-25:5	C30H52NO7PNa	[M+Na]+
592.3371033	592.3375	0.0004	0.6753	LPC 19:0;O	C27H56NO8PK	[M+K]+
592.3371033	592.3375	0.0004	0.6753	LPE 22:0;O	C27H56NO8PK	[M+K]+
592.3371033	592.3375	0.0004	0.6753	LPS O-21:0	C27H56NO8PK	[M+K]+
592.3371033	592.3375	0.0004	0.6753	PE O-22:0;O	C27H56NO8PK	[M+K]+
592.3582493	592.3585	0.0002	0.3376	LPS O-21:0;O	C27H56NO9PNa	[M+Na]+
593.1246481	593.1267	0.0021	3.5406	ST 19:5;O8;GlcA	C25H30O14K	[M+K]+
593.2483639	593.2486	0.0002	0.3371	BMP 22:6	C28H43O10PNa	[M+Na]+
593.2483639	593.2486	0.0002	0.3371	PG 22:6	C28H43O10PNa	[M+Na]+
593.2483639	593.2488	0.0004	0.6743	BMP 19:1;O	C25H47O11PK	[M+K]+
593.2483639	593.2488	0.0004	0.6743	LPI O-16:2	C25H47O11PK	[M+K]+
593.3393661	593.3368	0.0026	4.3820	LPA O-28:5	C31H55O6PK	[M+K]+
593.5866296	593.5867	0.0001	0.1685	DG O-36:0	C39H78O4	[M+H-H2O]+
	594.2521	0.0001	0.1683	ST 19:2;O7;HexNAc	C27H41NO12Na	[M+Na]+

594.2520347						
611.4156262	611.4154	0.0003	0.4907	ST 28:0;O3;GlcA	C34H58O9	[M+H]+
	611.4154	0.0003	0.4907	ST 28:1;O4;Hex	C34H58O9	[M+H]+
611.4156262						
611.4156262	611.4154	0.0003	0.4907	TG 31:3;O3	C34H58O9	[M+H]+
	611.4154	0.0003	0.4907	MGDG 25:2	C34H60O10	[M+H-H2O]+
611.4156262						
611.4156262	611.4154	0.0003	0.4907	ST 28:0;O5;Hex	C34H60O10	[M+H-H2O]+
	611.4159	0.0003	0.4907	EPC 29:2;O2	C31H61N2O6PNa	[M+Na]+
611.4156262						
611.5393275	611.5398	0.0004	0.6541	CE 14:1;O	C41H70O3	[M+H]+
	611.5398	0.0004	0.6541	CE 14:0;O2	C41H72O4	[M+H-H2O]+
611.5393275						
611.5393275	611.5398	0.0004	0.6541	DG O-38:5	C41H72O4	[M+H-H2O]+
	611.5398	0.0006	0.9811	CE 14:1;O	C41H70O3	[M+H]+
611.5403721						
611.5403721	611.5398	0.0006	0.9811	CE 14:0;O2	C41H72O4	[M+H-H2O]+
	611.5398	0.0006	0.9811	DG O-38:5	C41H72O4	[M+H-H2O]+
611.5403721						
612.6074937	612.6078	0.0003	0.4897	Cer 42:3;O	C42H79NO2	[M+H-H2O]+
	613.5554	0.0000	0.0000	CE 14:0;O	C41H72O3	[M+H]+
613.5553758						
613.5553758	613.5554	0.0000	0.0000	DG O-38:4	C41H74O4	[M+H-H2O]+
	614.6234	0.0002	0.3254	Cer 42:2;O	C42H81NO2	[M+H-H2O]+
614.6232562						
617.2460831	617.2488	0.0027	4.3742	BMP 21:3;O	C27H47O11PK	[M+K]+
	617.2488	0.0027	4.3742	LPI O-18:4	C27H47O11PK	[M+K]+
617.2460831						
617.2460831	617.2488	0.0027	4.3742	PG 21:3;O	C27H47O11PK	[M+K]+
618.2481340	618.2497	0.0016	2.5880	ST 28:5;O6;T	C30H45NO8SK	[M+K]+
	618.2521	0.0001	0.1617	ST 21:4;O7;HexNAc	C29H41NO12Na	[M+Na]+
618.2520005						
618.3411240	618.3402	0.0010	1.6172	CerP 30:5;O6	C30H52NO10P	[M+H]+
	618.3402	0.0010	1.6172	LPS 24:4;O	C30H52NO10P	[M+H]+
618.3411240						
618.3411240	618.3402	0.0010	1.6172	PS 24:3	C30H52NO10P	[M+H]+
	618.3402	0.0010	1.6172	PS O-24:4;O	C30H52NO10P	[M+H]+

618.3411240						
618.3411240	618.3402	0.0010	1.6172	PS 24:2;O	C30H54NO11P	[M+H-H2O]+
	618.3401	0.0010	1.6172	ST 27:6;O2;HexNAc	C35H49NO7Na	[M+Na]+
618.3411240						
618.3411240	618.3402	0.0010	1.6172	BMP 24:5	C30H49O10P	[M+NH4]+
	618.3402	0.0010	1.6172	LPG 24:6;O	C30H49O10P	[M+NH4]+
618.3411240						
618.3411240	618.3402	0.0010	1.6172	PG 24:5	C30H49O10P	[M+NH4]+
	618.3402	0.0010	1.6172	PG O-24:6;O	C30H49O10P	[M+NH4]+
618.3411240						
618.3411240	618.3403	0.0009	1.4555	CAR 25:5;O4	C32H53NO8K	[M+K]+
618.3411240	618.3403	0.0009	1.4555	ST 24:1;O3;HexNAc	C32H53NO8K	[M+K]+
	618.3403	0.0009	1.4555	ST 30:2;O7;G	C32H53NO8K	[M+K]+
618.3411240						
619.2635092	619.2644	0.0009	1.4533	BMP 21:2;O	C27H49O11PK	[M+K]+
	619.2644	0.0009	1.4533	LPI O-18:3	C27H49O11PK	[M+K]+
619.2635092						
619.2635092	619.2644	0.0009	1.4533	PG 21:2;O	C27H49O11PK	[M+K]+
	619.6024	0.0002	0.3228	DG O-38:1	C41H80O4	[M+H-H2O]+
619.6021869						
621.2794369	621.2799	0.0005	0.8048	BMP 24:6	C30H47O10PNa	[M+Na]+
	621.2799	0.0005	0.8048	PG 24:6	C30H47O10PNa	[M+Na]+
621.2794369						
621.2794369	621.2801	0.0006	0.9657	BMP 21:1;O	C27H51O11PK	[M+K]+
	621.2801	0.0006	0.9657	LPI O-18:2	C27H51O11PK	[M+K]+
621.2794369						
621.2794369	621.2801	0.0006	0.9657	PG 21:1;O	C27H51O11PK	[M+K]+
	621.2799	0.0006	0.9657	BMP 24:6	C30H47O10PNa	[M+Na]+
621.2805369						
621.2805369	621.2799	0.0006	0.9657	PG 24:6	C30H47O10PNa	[M+Na]+
	621.2801	0.0005	0.8048	BMP 21:1;O	C27H51O11PK	[M+K]+
621.2805369						
621.2805369	621.2801	0.0005	0.8048	LPI O-18:2	C27H51O11PK	[M+K]+
	621.2801	0.0005	0.8048	PG 21:1;O	C27H51O11PK	[M+K]+
621.2805369						
625.4314428	625.431	0.0004	0.6396	ST 29:0;O3;GlcA	C35H60O9	[M+H]+
	625.431	0.0004	0.6396	ST 29:1;O4;Hex	C35H60O9	[M+H]+

625.4314428						
625.4314428	625.431	0.0004	0.6396	TG 32:3;O3	C35H60O9	[M+H]+
	625.431	0.0004	0.6396	MGDG 26:2	C35H62O10	[M+H-H2O]+
625.4314428						
625.4314428	625.431	0.0004	0.6396	ST 29:0;O5;Hex	C35H62O10	[M+H-H2O]+
	625.4316	0.0001	0.1599	EPC 30:2;O2	C32H63N2O6PNa	[M+Na]+
625.4314428						
626.4897524	626.4908	0.0010	1.5962	CerP 36:2;O2	C36H70NO6P	[M+H-H2O]+
	626.4908	0.0010	1.5962	LPC O-28:3	C36H70NO6P	[M+H-H2O]+
626.4897524						
626.4897524	626.4908	0.0010	1.5962	LPE O-31:3	C36H70NO6P	[M+H-H2O]+
	626.4909	0.0011	1.7558	Cer 38:4;O2	C38H69NO3K	[M+K]+
626.4897524						
626.4907125	626.4908	0.0001	0.1596	CerP 36:2;O2	C36H70NO6P	[M+H-H2O]+
	626.4908	0.0001	0.1596	LPC O-28:3	C36H70NO6P	[M+H-H2O]+
626.4907125						
626.4907125	626.4908	0.0001	0.1596	LPE O-31:3	C36H70NO6P	[M+H-H2O]+
	626.4909	0.0002	0.3192	Cer 38:4;O2	C38H69NO3K	[M+K]+
626.4907125						
627.4940370	627.4943	0.0002	0.3187	CAR 28:3;O3	C35H63NO7	[M+NH4]+
	627.4943	0.0002	0.3187	Cer 35:4;O6	C35H63NO7	[M+NH4]+
627.4940370						
630.3397822	630.3402	0.0004	0.6346	CerP 31:6;O6	C31H52NO10P	[M+H]+
	630.3402	0.0004	0.6346	LPS 25:5;O	C31H52NO10P	[M+H]+
630.3397822						
630.3397822	630.3402	0.0004	0.6346	PS 25:4	C31H52NO10P	[M+H]+
	630.3402	0.0004	0.6346	PS O-25:5;O	C31H52NO10P	[M+H]+
630.3397822						
630.3397822	630.3402	0.0004	0.6346	PS 25:3;O	C31H54NO11P	[M+H-H2O]+
	630.3401	0.0003	0.4759	ST 28:7;O2;HexNAc	C36H49NO7Na	[M+Na]+
630.3397822						
630.3397822	630.3402	0.0004	0.6346	BMP 25:6	C31H49O10P	[M+NH4]+
630.3397822	630.3402	0.0004	0.6346	LPG 25:7;O	C31H49O10P	[M+NH4]+
	630.3402	0.0004	0.6346	PG 25:6	C31H49O10P	[M+NH4]+
630.3397822						
630.3397822	630.3402	0.0004	0.6346	PG O-25:7;O	C31H49O10P	[M+NH4]+
630.3397822	630.3403	0.0005	0.7932	CAR 26:6;O4	C33H53NO8K	[M+K]+

630.3397822	630.3403	0.0005	0.7932	ST 25:2;O3;HexNAc	C33H53NO8K	[M+K]+
630.3402027	630.3402	0.0000	0.0000	CerP 31:6;O6	C31H52NO10P	[M+H]+
630.3402027	630.3402	0.0000	0.0000	LPS 25:5;O	C31H52NO10P	[M+H]+
630.3402027	630.3402	0.0000	0.0000	PS 25:4	C31H52NO10P	[M+H]+
630.3402027	630.3402	0.0000	0.0000	PS O-25:5;O	C31H52NO10P	[M+H]+
630.3402027	630.3402	0.0000	0.0000	PS 25:3;O	C31H54NO11P	[M+H-H2O]+
630.3402027	630.3401	0.0001	0.1586	ST 28:7;O2;HexNAc	C36H49NO7Na	[M+Na]+
630.3402027	630.3402	0.0000	0.0000	BMP 25:6	C31H49O10P	[M+NH4]+
630.3402027	630.3402	0.0000	0.0000	LPG 25:7;O	C31H49O10P	[M+NH4]+
630.3402027	630.3402	0.0000	0.0000	PG 25:6	C31H49O10P	[M+NH4]+
630.3402027	630.3402	0.0000	0.0000	PG O-25:7;O	C31H49O10P	[M+NH4]+
630.3402027	630.3403	0.0001	0.1586	CAR 26:6;O4	C33H53NO8K	[M+K]+
630.3402027	630.3403	0.0001	0.1586	ST 25:2;O3;HexNAc	C33H53NO8K	[M+K]+
631.3433620	631.3436	0.0003	0.4752	ST 22:2;O7;HexNAc	C30H47NO12	[M+NH4]+
631.5646357	631.566	0.0013	2.0584	DG O-38:4	C41H74O4	[M+H]+
631.5646357	631.566	0.0013	2.0584	DG 38:2	C41H76O5	[M+H-H2O]+
631.5646357	631.566	0.0013	2.0584	DG O-38:3;O	C41H76O5	[M+H-H2O]+
631.5646357	631.566	0.0013	2.0584	TG O-38:2	C41H76O5	[M+H-H2O]+
631.5646357	631.5636	0.0011	1.7417	DG O-36:1	C39H76O4Na	[M+Na]+
632.3469945	632.3463	0.0007	1.1070	ST 29:2;O8;T	C31H53NO10S	[M+H]+
632.3469945	632.3477	0.0007	1.1070	CerP 33:6;O2	C33H56NO6PK	[M+K]+
632.3469945	632.3477	0.0007	1.1070	LPC O-25:7	C33H56NO6PK	[M+K]+
632.3469945	632.3477	0.0007	1.1070	LPE O-28:7	C33H56NO6PK	[M+K]+
633.2577090	633.2589	0.0012	1.8950	PA 28:7;O	C31H47O9PK	[M+K]+
633.2607873	633.2589	0.0019	3.0004	PA 28:7;O	C31H47O9PK	[M+K]+

	636.2908	0.0023	3.6147	LPS 24:6;O	C30H48NO10PNa	[M+Na]+
636.2885484						
636.2885484	636.2908	0.0023	3.6147	PS 24:5	C30H48NO10PNa	[M+Na]+
	636.2908	0.0023	3.6147	PS O-24:6;O	C30H48NO10PNa	[M+Na]+
636.2885484						
636.2885484	636.291	0.0024	3.7719	PS 21:0;O	C27H52NO11PK	[M+K]+
	636.2908	0.0007	1.1001	LPS 24:6;O	C30H48NO10PNa	[M+Na]+
636.2914890						
636.2914890	636.2908	0.0007	1.1001	PS 24:5	C30H48NO10PNa	[M+Na]+
	636.2908	0.0007	1.1001	PS O-24:6;O	C30H48NO10PNa	[M+Na]+
636.2914890						
636.2914890	636.291	0.0005	0.7858	PS 21:0;O	C27H52NO11PK	[M+K]+
637.2965855	637.2959	0.0006	0.9415	LPI 18:1;O	C27H51O13PNa	[M+Na]+
	637.2967	0.0001	0.1569	ST 23:6;O7;HexNAc	C31H41NO12	[M+NH4]+
637.2965855						
637.5763957	637.5765	0.0001	0.1568	DG 37:1	C40H76O5	[M+H]+
637.5763957	637.5765	0.0001	0.1568	DG O-37:2;O	C40H76O5	[M+H]+
	637.5765	0.0001	0.1568	TG O-37:1	C40H76O5	[M+H]+
637.5763957						
637.5763957	637.5765	0.0001	0.1568	DG 37:0;O	C40H78O6	[M+H-H2O]+
	637.5765	0.0001	0.1568	DG O-37:1;O2	C40H78O6	[M+H-H2O]+
637.5763957						
637.5763957	637.5765	0.0001	0.1568	TG O-37:0;O	C40H78O6	[M+H-H2O]+
	638.3065	0.0001	0.1567	CerP 30:6;O6	C30H50NO10PNa	[M+Na]+
638.3063938						
638.3063938	638.3065	0.0001	0.1567	LPS 24:5;O	C30H50NO10PNa	[M+Na]+
	638.3065	0.0001	0.1567	PS 24:4	C30H50NO10PNa	[M+Na]+
638.3063938						
638.3063938	638.3065	0.0001	0.1567	PS O-24:5;O	C30H50NO10PNa	[M+Na]+
	639.3116	0.0012	1.8770	LPI 18:0;O	C27H53O13PNa	[M+Na]+
639.3103867						
639.4552214	639.4579	0.0027	4.2223	CAR 28:5;O4	C35H59NO8	[M+NH4]+
639.4552214	639.4579	0.0027	4.2223	HexCer 29:5;O2	C35H59NO8	[M+NH4]+
	639.4579	0.0027	4.2223	ST 27:1;O3;HexNAc	C35H59NO8	[M+NH4]+
639.4552214						
640.3213070	640.3221	0.0008	1.2494	CerP 30:5;O6	C30H52NO10PNa	[M+Na]+

	640.3221	0.0008	1.2494	LPS 24:4;O	C30H52NO10PNa	[M+Na]+
640.3213070						
640.3213070	640.3221	0.0008	1.2494	PS 24:3	C30H52NO10PNa	[M+Na]+
	640.3221	0.0008	1.2494	PS O-24:4;O	C30H52NO10PNa	[M+Na]+
640.3213070						
640.4674625	640.4676	0.0002	0.3123	CerP 34:1;O2	C34H68NO6PNa	[M+Na]+
	640.4676	0.0002	0.3123	LPC O-26:2	C34H68NO6PNa	[M+Na]+
640.4674625						
640.4674625	640.4676	0.0002	0.3123	LPE O-29:2	C34H68NO6PNa	[M+Na]+
	641.383	0.0001	0.1559	NAT 31:7;O4	C33H53NO8S	[M+NH4]+
641.3831436						
641.4711194	641.4694	0.0017	2.6502	CE 15:4	C42H66O2K	[M+K]+
	643.2644	0.0004	0.6218	BMP 23:4;O	C29H49O11PK	[M+K]+
643.2640506						
643.2640506	643.2644	0.0004	0.6218	LPI O-20:5	C29H49O11PK	[M+K]+
	643.2644	0.0004	0.6218	PG 23:4;O	C29H49O11PK	[M+K]+
643.2640506						
644.3556078	644.3558	0.0002	0.3104	CerP 32:6;O6	C32H54NO10P	[M+H]+
	644.3558	0.0002	0.3104	LPS 26:5;O	C32H54NO10P	[M+H]+
644.3556078						
644.3556078	644.3558	0.0002	0.3104	PS 26:4	C32H54NO10P	[M+H]+
	644.3558	0.0002	0.3104	PS O-26:5;O	C32H54NO10P	[M+H]+
644.3556078						
644.3556078	644.3558	0.0002	0.3104	PS 26:3;O	C32H56NO11P	[M+H-H2O]+
644.3556078	644.3558	0.0002	0.3104	ST 29:7;O2;HexNAc	C37H51NO7Na	[M+Na]+
	644.3558	0.0002	0.3104	BMP 26:6	C32H51O10P	[M+NH4]+
644.3556078						
644.3556078	644.3558	0.0002	0.3104	LPG 26:7;O	C32H51O10P	[M+NH4]+
644.3556078	644.3558	0.0002	0.3104	PG 26:6	C32H51O10P	[M+NH4]+
	644.3558	0.0002	0.3104	PG O-26:7;O	C32H51O10P	[M+NH4]+
644.3556078						
644.3556078	644.3559	0.0003	0.4656	CAR 27:6;O4	C34H55NO8K	[M+K]+
644.3556078	644.3559	0.0003	0.4656	HexCer 28:6;O2	C34H55NO8K	[M+K]+
644.3556078	644.3559	0.0003	0.4656	ST 26:2;O3;HexNAc	C34H55NO8K	[M+K]+
	645.3593	0.0007	1.0847	ST 23:2;O7;HexNAc	C31H49NO12	[M+NH4]+

645.3585743						
648.4725538	648.4711	0.0014	2.1589	EPC 31:3;O3	C33H63N2O7P	[M+NH4]+
	648.4711	0.0014	2.1589	SM 28:3;O3	C33H63N2O7P	[M+NH4]+
648.4725538						
649.4759508	649.4786	0.0027	4.1572	CAR 30:6;O3	C37H61NO7	[M+NH4]+
	649.4786	0.0027	4.1572	ST 29:2;O2;HexNAc	C37H61NO7	[M+NH4]+
649.4759508						
652.3220180	652.3221	0.0001	0.1533	CerP 31:6;O6	C31H52NO10PNa	[M+Na]+
	652.3221	0.0001	0.1533	LPS 25:5;O	C31H52NO10PNa	[M+Na]+
652.3220180						
652.3220180	652.3221	0.0001	0.1533	PS 25:4	C31H52NO10PNa	[M+Na]+
	652.3221	0.0001	0.1533	PS O-25:5;O	C31H52NO10PNa	[M+Na]+
652.3220180						
652.4674513	652.4676	0.0002	0.3065	CerP 35:2;O2	C35H68NO6PNa	[M+Na]+
	652.4676	0.0002	0.3065	LPC O-27:3	C35H68NO6PNa	[M+Na]+
652.4674513						
652.4674513	652.4676	0.0002	0.3065	LPE O-30:3	C35H68NO6PNa	[M+Na]+
	653.4623	0.0005	0.7652	TG 34:3;O3	C37H64O9	[M+H]+
653.4628094						
653.4628094	653.4623	0.0005	0.7652	MGDG 28:2	C37H66O10	[M+H-H2O]+
	653.4629	0.0001	0.1530	EPC 32:2;O2	C34H67N2O6PNa	[M+Na]+
653.4628094						
653.4628094	653.4629	0.0001	0.1530	SM 29:2;O2	C34H67N2O6PNa	[M+Na]+
	653.4735	0.0026	3.9787	CAR 29:5;O4	C36H61NO8	[M+NH4]+
653.4709796						
653.4709796	653.4735	0.0026	3.9787	HexCer 30:5;O2	C36H61NO8	[M+NH4]+
	653.4735	0.0026	3.9787	ST 28:1;O3;HexNAc	C36H61NO8	[M+NH4]+
653.4709796						
654.6158053	654.6159	0.0001	0.1528	Cer 42:2;O	C42H81NO2Na	[M+Na]+
	656.3558	0.0002	0.3047	LPS 27:6;O	C33H54NO10P	[M+H]+
656.3556224						
656.3556224	656.3558	0.0002	0.3047	PS 27:5	C33H54NO10P	[M+H]+
	656.3558	0.0002	0.3047	PS O-27:6;O	C33H54NO10P	[M+H]+
656.3556224						
656.3556224	656.3558	0.0002	0.3047	PS 27:4;O	C33H56NO11P	[M+H-H2O]+
	656.3558	0.0002	0.3047	BMP 27:7	C33H51O10P	[M+NH4]+

656.3556224						
656.3556224	656.3558	0.0002	0.3047	PG 27:7	C33H51O10P	[M+NH4]+
656.3556224	656.3559	0.0003	0.4571	CAR 28:7;O4	C35H55NO8K	[M+K]+
656.3556224	656.3559	0.0003	0.4571	ST 27:3;O3;HexNAc	C35H55NO8K	[M+K]+
657.3586322	657.3593	0.0007	1.0649	ST 24:3;O7;HexNAc	C32H49NO12	[M+NH4]+
	657.361	0.0015	2.2819	LPI 21:1;O	C30H57O13P	[M+H]+
657.3624725						
657.3624725	657.361	0.0015	2.2819	PI 21:0	C30H57O13P	[M+H]+
	657.361	0.0015	2.2819	PI O-21:1;O	C30H57O13P	[M+H]+
657.3624725						
657.3624725	657.3609	0.0016	2.4340	MGDG 26:6	C35H54O10Na	[M+Na]+
	657.3609	0.0016	2.4340	ST 29:3;O4;GlcA	C35H54O10Na	[M+Na]+
657.3624725						
657.3624725	657.3609	0.0016	2.4340	ST 29:4;O5;Hex	C35H54O10Na	[M+Na]+
	657.3641	0.0016	2.4340	EPC 29:3;O4	C31H59N2O8PK	[M+K]+
657.3624725						
658.2743734	658.2753	0.0009	1.3672	PS 23:3;O	C29H50NO11PK	[M+K]+
	658.3715	0.0001	0.1519	CerP 33:6;O6	C33H56NO10P	[M+H]+
658.3713618						
658.3713618	658.3715	0.0001	0.1519	LPS 27:5;O	C33H56NO10P	[M+H]+
	658.3715	0.0001	0.1519	PS 27:4	C33H56NO10P	[M+H]+
658.3713618						
658.3713618	658.3715	0.0001	0.1519	PS O-27:5;O	C33H56NO10P	[M+H]+
658.3713618	658.3715	0.0001	0.1519	PS 27:3;O	C33H58NO11P	[M+H-H2O]+
658.3713618	658.3714	0.0001	0.1519	ST 30:7;O2;HexNAc	C38H53NO7Na	[M+Na]+
	658.3715	0.0001	0.1519	BMP 27:6	C33H53O10P	[M+NH4]+
658.3713618						
658.3713618	658.3715	0.0001	0.1519	LPG 27:7;O	C33H53O10P	[M+NH4]+
	658.3715	0.0001	0.1519	PG 27:6	C33H53O10P	[M+NH4]+
658.3713618						
658.3713618	658.3715	0.0001	0.1519	PG O-27:7;O	C33H53O10P	[M+NH4]+
	658.3716	0.0002	0.3038	CAR 28:6;O4	C35H57NO8K	[M+K]+
658.3713618						
658.3713618	658.3716	0.0002	0.3038	HexCer 29:6;O2	C35H57NO8K	[M+K]+
658.3713618	658.3716	0.0002	0.3038	ST 27:2;O3;HexNAc	C35H57NO8K	[M+K]+

	659.2803	0.0012	1.8202	LPI 20:4;O	C29H49O13PNa	[M+Na]+
659.2790905						
659.2790905	659.2803	0.0012	1.8202	PI 20:3	C29H49O13PNa	[M+Na]+
	659.2803	0.0012	1.8202	PI O-20:4;O	C29H49O13PNa	[M+Na]+
659.2790905						
659.2807185	659.2827	0.0020	3.0336	PI 22:6	C31H47O13P	[M+H]+
	659.2827	0.0020	3.0336	PI 22:5;O	C31H49O14P	[M+H-H2O]+
659.2807185						
659.2807185	659.2803	0.0004	0.6067	LPI 20:4;O	C29H49O13PNa	[M+Na]+
	659.2803	0.0004	0.6067	PI 20:3	C29H49O13PNa	[M+Na]+
659.2807185						
659.2807185	659.2803	0.0004	0.6067	PI O-20:4;O	C29H49O13PNa	[M+Na]+
	659.3749	0.0002	0.3033	ST 24:2;O7;HexNAc	C32H51NO12	[M+NH4]+
659.3747307						
660.2888060	660.2908	0.0020	3.0290	PS 26:7	C32H48NO10PNa	[M+Na]+
	660.291	0.0021	3.1804	PS 23:2;O	C29H52NO11PK	[M+K]+
660.2888060						
660.2905254	660.2908	0.0003	0.4543	PS 26:7	C32H48NO10PNa	[M+Na]+
	660.291	0.0004	0.6058	PS 23:2;O	C29H52NO11PK	[M+K]+
660.2905254						
660.3777385	660.379	0.0012	1.8171	CerP 35:6;O2	C35H60NO6PK	[M+K]+
660.3777385	660.379	0.0012	1.8171	LPC O-27:7	C35H60NO6PK	[M+K]+
	660.379	0.0012	1.8171	LPE O-30:7	C35H60NO6PK	[M+K]+
660.3777385						
661.2923132	661.2902	0.0021	3.1756	PA 30:7;O	C33H51O9PK	[M+K]+
662.2950355	662.2969	0.0019	2.8688	ST 30:5;O8;T	C32H49NO10SNa	[M+Na]+
662.2950355	662.2936	0.0014	2.1139	PI 21:6	C30H45O13P	[M+NH4]+
	662.2937	0.0013	1.9629	ST 24:3;O6;HexNAc	C32H49NO11K	[M+K]+
662.2950355						
662.4491660	662.4474	0.0018	2.7172	HexCer 28:2;O5	C34H63NO11	[M+H]+
662.4491660	662.4474	0.0018	2.7172	HexCer 28:1;O6	C34H65NO12	[M+H-H2O]+
	662.4504	0.0012	1.8115	EPC 31:4;O4	C33H61N2O8P	[M+NH4]+
662.4491660						
662.4491660	662.4504	0.0012	1.8115	SM 28:4;O4	C33H61N2O8P	[M+NH4]+
	662.4474	0.0018	2.7172	ST 28:0;O6;Hex	C34H60O11	[M+NH4]+

662.4491660						
662.4504984	662.4504	0.0001	0.1510	EPC 31:4;O4	C33H61N2O8P	[M+NH4]+
	662.4504	0.0001	0.1510	SM 28:4;O4	C33H61N2O8P	[M+NH4]+
662.4504984						
663.3933360	663.395	0.0017	2.5626	ST 27:0;O8;Hex	C33H58O13	[M+H]+
	663.3956	0.0023	3.4670	EPC 28:1;O6	C30H61N2O10PNa	[M+Na]+
663.3933360						
664.2837149	664.2857	0.0020	3.0108	PS 25:6;O	C31H48NO11PNa	[M+Na]+
	664.2964	0.0003	0.4516	ST 25:6;O8;HexNAc	C33H45NO13	[M+H]+
664.2960755						
664.2960755	664.2964	0.0003	0.4516	ST 27:7;O7;GlcA	C33H42O13	[M+NH4]+
	664.4676	0.0002	0.3010	CerP 36:3;O2	C36H68NO6PNa	[M+Na]+
664.4674917						
664.4674917	664.4676	0.0002	0.3010	LPC O-28:4	C36H68NO6PNa	[M+Na]+
	664.4676	0.0002	0.3010	LPE O-31:4	C36H68NO6PNa	[M+Na]+
664.4674917						
665.4709606	665.4735	0.0026	3.9070	CAR 30:6;O4	C37H61NO8	[M+NH4]+
665.4709606	665.4735	0.0026	3.9070	HexCer 31:6;O2	C37H61NO8	[M+NH4]+
	665.4735	0.0026	3.9070	ST 29:2;O3;HexNAc	C37H61NO8	[M+NH4]+
665.4709606						
666.3371162	666.3378	0.0006	0.9004	CerP 32:6;O6	C32H54NO10PNa	[M+Na]+
	666.3378	0.0006	0.9004	LPS 26:5;O	C32H54NO10PNa	[M+Na]+
666.3371162						
666.3371162	666.3378	0.0006	0.9004	PS 26:4	C32H54NO10PNa	[M+Na]+
	666.3378	0.0006	0.9004	PS O-26:5;O	C32H54NO10PNa	[M+Na]+
666.3371162						
666.4831847	666.4833	0.0001	0.1500	CerP 36:2;O2	C36H70NO6PNa	[M+Na]+
666.4831847	666.4833	0.0001	0.1500	LPC O-28:3	C36H70NO6PNa	[M+Na]+
	666.4833	0.0001	0.1500	LPE O-31:3	C36H70NO6PNa	[M+Na]+
666.4831847						
667.4865293	667.4892	0.0027	4.0450	CAR 30:5;O4	C37H63NO8	[M+NH4]+
667.4865293	667.4892	0.0027	4.0450	HexCer 31:5;O2	C37H63NO8	[M+NH4]+
	667.4892	0.0027	4.0450	ST 29:1;O3;HexNAc	C37H63NO8	[M+NH4]+
667.4865293						
667.5271735	667.5272	0.0001	0.1498	LPG O-30:0	C36H75O8P	[M+H]+
	667.5272	0.0000	0.0000	CE 14:0;O3	C41H72O5Na	[M+Na]+

667.5271735						
667.5271735	667.5272	0.0000	0.0000	DG 38:4	C41H72O5Na	[M+Na]+
667.5271735	667.5272	0.0000	0.0000	DG O-38:5;O	C41H72O5Na	[M+Na]+
	667.5272	0.0000	0.0000	TG O-38:4	C41H72O5Na	[M+Na]+
667.5271735						
667.5271735	667.5273	0.0002	0.2996	DG O-35:0;O2	C38H76O6K	[M+K]+
	668.4885	0.0010	1.4959	CAR 34:7;O3	C41H67NO7	[M+H-H2O]+
668.4894093						
668.4894093	668.4894	0.0000	0.0000	NAT 34:1;O2	C36H71NO6SNa	[M+Na]+
	668.4885	0.0010	1.4959	DG 38:9;O	C41H62O6	[M+NH4]+
668.4894093						
668.4894093	668.4885	0.0010	1.4959	DG O-38:10;O2	C41H62O6	[M+NH4]+
	668.4885	0.0010	1.4959	TG 38:8	C41H62O6	[M+NH4]+
668.4894093						
668.4894093	668.4885	0.0010	1.4959	TG O-38:9;O	C41H62O6	[M+NH4]+
	669.4936	0.0003	0.4481	TG 35:2;O3	C38H68O9	[M+H]+
669.4932666						
669.4932666	669.4936	0.0003	0.4481	MGDG 29:1	C38H70O10	[M+H-H2O]+
	669.4942	0.0009	1.3443	EPC 33:1;O2	C35H71N2O6PNa	[M+Na]+
669.4932666						
669.4932666	669.4942	0.0009	1.3443	SM 30:1;O2	C35H71N2O6PNa	[M+Na]+
	669.5007	0.0015	2.2405	CE 17:4	C44H70O2K	[M+K]+
669.5022301						
670.5053944	670.5051	0.0003	0.4474	NAT 34:0;O2	C36H73NO6SNa	[M+Na]+
674.3659270	674.3664	0.0004	0.5931	PS 27:4;O	C33H56NO11P	[M+H]+
	674.3663	0.0004	0.5931	ST 30:7;O3;HexNAc	C38H53NO8Na	[M+Na]+
674.3659270						
674.3659270	674.3664	0.0004	0.5931	BMP 27:6;O	C33H53O11P	[M+NH4]+
	674.3664	0.0004	0.5931	PG 27:6;O	C33H53O11P	[M+NH4]+
674.3659270						
674.3659270	674.3665	0.0006	0.8897	HexCer 29:6;O3	C35H57NO9K	[M+K]+
	674.3665	0.0006	0.8897	ST 27:2;O4;HexNAc	C35H57NO9K	[M+K]+
674.3659270						
677.5686701	677.5691	0.0004	0.5903	DG 37:0;O	C40H78O6Na	[M+Na]+
	677.5691	0.0004	0.5903	DG O-37:1;O2	C40H78O6Na	[M+Na]+
677.5686701						
677.5686701	677.5691	0.0004	0.5903	TG O-37:0;O	C40H78O6Na	[M+Na]+

677.5702813	677.5715	0.0012	1.7710	DG 39:3;O	C42H76O6	[M+H]+
677.5702813	677.5715	0.0012	1.7710	DG O-39:4;O2	C42H76O6	[M+H]+
677.5702813	677.5715	0.0012	1.7710	TG 39:2	C42H76O6	[M+H]+
677.5702813	677.5715	0.0012	1.7710	TG O-39:3;O	C42H76O6	[M+H]+
677.5702813	677.5715	0.0012	1.7710	DG 39:2;O2	C42H78O7	[M+H-H2O]+
677.5702813	677.5715	0.0012	1.7710	TG 39:1;O	C42H78O7	[M+H-H2O]+
677.5702813	677.5715	0.0012	1.7710	TG O-39:2;O2	C42H78O7	[M+H-H2O]+
677.5702813	677.5691	0.0012	1.7710	DG 37:0;O	C40H78O6Na	[M+Na]+
677.5702813	677.5691	0.0012	1.7710	DG O-37:1;O2	C40H78O6Na	[M+Na]+
677.5702813	677.5691	0.0012	1.7710	TG O-37:0;O	C40H78O6Na	[M+Na]+
678.3385161	678.3378	0.0008	1.1794	LPS 27:6;O	C33H54NO10PNa	[M+Na]+
678.3385161	678.3378	0.0008	1.1794	PS 27:5	C33H54NO10PNa	[M+Na]+
678.3385161	678.3378	0.0008	1.1794	PS O-27:6;O	C33H54NO10PNa	[M+Na]+
678.3385161	678.3379	0.0006	0.8845	PS 24:0;O	C30H58NO11PK	[M+K]+
678.3407334	678.3402	0.0006	0.8845	PS 29:8	C35H52NO10P	[M+H]+
678.3407334	678.3402	0.0006	0.8845	PS 29:7;O	C35H54NO11P	[M+H-H2O]+
678.3407334	678.3403	0.0005	0.7371	ST 29:6;O3;HexNAc	C37H53NO8K	[M+K]+
679.3433554	679.3429	0.0005	0.7360	LPI 21:1;O	C30H57O13PNa	[M+Na]+
679.3433554	679.3429	0.0005	0.7360	PI 21:0	C30H57O13PNa	[M+Na]+
679.3433554	679.3429	0.0005	0.7360	PI O-21:1;O	C30H57O13PNa	[M+Na]+
679.3433554	679.3436	0.0003	0.4416	ST 26:6;O7;HexNAc	C34H47NO12	[M+NH4]+
679.5845353	679.5847	0.0002	0.2943	DG O-37:0;O2	C40H80O6Na	[M+Na]+
680.3537088	680.3534	0.0003	0.4409	CerP 33:6;O6	C33H56NO10PNa	[M+Na]+
680.3537088	680.3534	0.0003	0.4409	LPS 27:5;O	C33H56NO10PNa	[M+Na]+
	680.3534	0.0003	0.4409	PS 27:4	C33H56NO10PNa	[M+Na]+

680.3537088						
680.3537088	680.3534	0.0003	0.4409	PS O-27:5;O	C33H56NO10PNa	[M+Na]+
680.4984399	680.4989	0.0005	0.7348	CerP 37:2;O2	C37H72NO6PNa	[M+Na]+
	680.4989	0.0005	0.7348	LPC O-29:3	C37H72NO6PNa	[M+Na]+
680.4984399						
680.4984399	680.4989	0.0005	0.7348	LPE O-32:3	C37H72NO6PNa	[M+Na]+
	680.4973	0.0011	1.6165	EPC 32:2;O4	C34H67N2O8P	[M+NH4]+
680.4984399						
680.4984399	680.4973	0.0011	1.6165	SM 29:2;O4	C34H67N2O8P	[M+NH4]+
	681.2646	0.0018	2.6421	PI 22:6	C31H47O13PNa	[M+Na]+
681.2628200						
681.3571085	681.3585	0.0014	2.0547	LPI 21:0;O	C30H59O13PNa	[M+Na]+
	681.3585	0.0014	2.0547	PI O-21:0;O	C30H59O13PNa	[M+Na]+
681.3571085						
681.4930952	681.4936	0.0005	0.7337	TG 36:3;O3	C39H68O9	[M+H]+
	681.4936	0.0005	0.7337	MGDG 30:2	C39H70O10	[M+H-H2O]+
681.4930952						
681.4930952	681.4942	0.0011	1.6141	EPC 34:2;O2	C36H71N2O6PNa	[M+Na]+
	681.4942	0.0011	1.6141	SM 31:2;O2	C36H71N2O6PNa	[M+Na]+
681.4930952						
682.2726997	682.2753	0.0026	3.8108	PS 25:5;O	C31H50NO11PK	[M+K]+
	682.3691	0.0016	2.3448	CerP 33:5;O6	C33H58NO10PNa	[M+Na]+
682.3674826						
682.3674826	682.3691	0.0016	2.3448	LPS 27:4;O	C33H58NO10PNa	[M+Na]+
	682.3691	0.0016	2.3448	PS 27:3	C33H58NO10PNa	[M+Na]+
682.3674826						
682.3674826	682.3691	0.0016	2.3448	PS O-27:4;O	C33H58NO10PNa	[M+Na]+
	682.3715	0.0004	0.5862	LPS 29:7;O	C35H56NO10P	[M+H]+
682.3711028						
682.3711028	682.3715	0.0004	0.5862	PS 29:6	C35H56NO10P	[M+H]+
	682.3715	0.0004	0.5862	PS O-29:7;O	C35H56NO10P	[M+H]+
682.3711028						
682.3711028	682.3715	0.0004	0.5862	IPC 29:6;O2	C35H58NO11P	[M+H-H2O]+
	682.3715	0.0004	0.5862	PS 29:5;O	C35H58NO11P	[M+H-H2O]+
682.3711028						
682.3711028	682.3715	0.0004	0.5862	PG 29:8	C35H53O10P	[M+NH4]+
682.3711028	682.3716	0.0005	0.7327	ST 29:4;O3;HexNAc	C37H57NO8K	[M+K]+

	683.3749	0.0013	1.9023	ST 26:4;O7;HexNAc	C34H51NO12	[M+NH4]+
683.3736600						
688.4650196	688.466	0.0010	1.4525	EPC 33:5;O4	C35H63N2O8P	[M+NH4]+
	688.466	0.0010	1.4525	SM 30:5;O4	C35H63N2O8P	[M+NH4]+
688.4650196						
690.2985580	690.3014	0.0028	4.0562	PS 27:7;O	C33H50NO11PNa	[M+Na]+
	691.3065	0.0001	0.1447	PI 21:2;O	C30H53O14PNa	[M+Na]+
691.3066108						
692.3146207	692.317	0.0024	3.4666	PS 27:6;O	C33H52NO11PNa	[M+Na]+
	693.3164	0.0015	2.1635	BMP 28:6	C34H55O10PK	[M+K]+
693.3179406						
693.3179406	693.3164	0.0015	2.1635	LPG 28:7;O	C34H55O10PK	[M+K]+
	693.3164	0.0015	2.1635	PG 28:6	C34H55O10PK	[M+K]+
693.3179406						
693.3179406	693.3164	0.0015	2.1635	PG O-28:7;O	C34H55O10PK	[M+K]+
	693.3222	0.0005	0.7212	PI 21:1;O	C30H55O14PNa	[M+Na]+
693.3216227						
695.4984544	695.4986	0.0002	0.2876	PA O-35:2	C38H73O7PNa	[M+Na]+
696.3481289	696.3483	0.0002	0.2872	PS 27:4;O	C33H56NO11PNa	[M+Na]+
697.3521509	697.3542	0.0021	3.0114	ST 26:5;O8;HexNAc	C34H49NO13	[M+NH4]+
	699.5323	0.0005	0.7148	PA O-37:3	C40H75O7P	[M+H]+
699.5328485						
699.5328485	699.5323	0.0005	0.7148	LPG O-34:3	C40H77O8P	[M+H-H2O]+
	699.5323	0.0005	0.7148	PA 37:1	C40H77O8P	[M+H-H2O]+
699.5328485						
699.5328485	699.5323	0.0005	0.7148	PA O-37:2;O	C40H77O8P	[M+H-H2O]+
	699.5323	0.0006	0.8577	CE 18:4;O2	C45H72O4Na	[M+Na]+
699.5328485						
699.5328485	699.5323	0.0006	0.8577	DG O-42:9	C45H72O4Na	[M+Na]+
	699.5324	0.0004	0.5718	DG 39:3	C42H76O5K	[M+K]+
699.5328485						
699.5328485	699.5324	0.0004	0.5718	DG O-39:4;O	C42H76O5K	[M+K]+
	699.5324	0.0004	0.5718	TG O-39:3	C42H76O5K	[M+K]+
699.5328485						
702.3363252	702.3378	0.0014	1.9933	PS 29:7	C35H54NO10PNa	[M+Na]+

	702.3378	0.0014	1.9933	PS O-29:8;O	C35H54NO10PNa	[M+Na]+
702.3363252						
702.3363252	702.3379	0.0016	2.2781	PS 26:2;O	C32H58NO11PK	[M+K]+
	703.3372	0.0012	1.7062	PA 33:7;O	C36H57O9PK	[M+K]+
703.3383584						
703.3383584	703.3372	0.0012	1.7062	PG O-30:8	C36H57O9PK	[M+K]+
	703.3429	0.0008	1.1374	LPI 23:3;O	C32H57O13PNa	[M+Na]+
703.3420714						
703.3420714	703.3429	0.0008	1.1374	PI 23:2	C32H57O13PNa	[M+Na]+
	703.3429	0.0008	1.1374	PI O-23:3;O	C32H57O13PNa	[M+Na]+
703.3420714						
704.3136776	704.317	0.0033	4.6854	PS 28:7;O	C34H52NO11PNa	[M+Na]+
	705.3585	0.0015	2.1266	LPI 23:2;O	C32H59O13PNa	[M+Na]+
705.3570181						
705.3570181	705.3585	0.0015	2.1266	PI 23:1	C32H59O13PNa	[M+Na]+
	705.3585	0.0015	2.1266	PI O-23:2;O	C32H59O13PNa	[M+Na]+
705.3570181						
706.3718111	706.3715	0.0004	0.5663	PS 31:8	C37H56NO10P	[M+H]+
	706.3715	0.0004	0.5663	PS 31:7;O	C37H58NO11P	[M+H-H2O]+
706.3718111						
707.3732641	707.3742	0.0009	1.2723	LPI 23:1;O	C32H61O13PNa	[M+Na]+
707.3732641	707.3742	0.0009	1.2723	PI 23:0	C32H61O13PNa	[M+Na]+
	707.3742	0.0009	1.2723	PI O-23:1;O	C32H61O13PNa	[M+Na]+
707.3732641						
707.4984743	707.4986	0.0001	0.1413	PA O-36:3	C39H73O7PNa	[M+Na]+
	707.501	0.0010	1.4134	PA O-38:6	C41H71O7P	[M+H]+
707.5019769						
707.5019769	707.501	0.0010	1.4134	PA 38:4	C41H73O8P	[M+H-H2O]+
	707.501	0.0010	1.4134	PA O-38:5;O	C41H73O8P	[M+H-H2O]+
707.5019769						
707.5019769	707.501	0.0010	1.4134	DG O-43:12	C46H68O4Na	[M+Na]+
	707.5011	0.0008	1.1307	CE 16:2;O3	C43H72O5K	[M+K]+
707.5019769						
707.5019769	707.5011	0.0008	1.1307	DG 40:6	C43H72O5K	[M+K]+
	707.5011	0.0008	1.1307	DG O-40:7;O	C43H72O5K	[M+K]+
707.5019769						
707.5019769	707.5011	0.0008	1.1307	TG O-40:6	C43H72O5K	[M+K]+

707.5970027	707.5973	0.0003	0.4240	CE 20:3;O2	C47H78O4	[M+H]+
707.5970027	707.5973	0.0003	0.4240	DG O-44:8	C47H78O4	[M+H]+
707.5970027	707.5973	0.0003	0.4240	CE 20:2;O3	C47H80O5	[M+H-H2O]+
707.5970027	707.5973	0.0003	0.4240	DG 44:6	C47H80O5	[M+H-H2O]+
707.5970027	707.5973	0.0003	0.4240	DG O-44:7;O	C47H80O5	[M+H-H2O]+
707.5970027	707.5973	0.0003	0.4240	TG O-44:6	C47H80O5	[M+H-H2O]+
708.5020147	708.5021	0.0001	0.1411	HexCer 32:2;O3	C38H71NO9Na	[M+Na]+
711.5339723	711.5347	0.0007	0.9838	DG 45:11	C48H72O5	[M+H-H2O]+
711.5339723	711.5347	0.0007	0.9838	DG O-45:12;O	C48H72O5	[M+H-H2O]+
711.5339723	711.5347	0.0007	0.9838	TG O-45:11	C48H72O5	[M+H-H2O]+
713.5475705	713.548	0.0004	0.5606	PA O-38:3	C41H77O7P	[M+H]+
713.5475705	713.548	0.0004	0.5606	PA 38:1	C41H79O8P	[M+H-H2O]+
713.5475705	713.548	0.0004	0.5606	PA O-38:2;O	C41H79O8P	[M+H-H2O]+
713.5475705	713.5479	0.0004	0.5606	CE 19:4;O2	C46H74O4Na	[M+Na]+
713.5475705	713.5479	0.0004	0.5606	DG O-43:9	C46H74O4Na	[M+Na]+
713.5475705	713.5481	0.0005	0.7007	DG 40:3	C43H78O5K	[M+K]+
713.5475705	713.5481	0.0005	0.7007	DG O-40:4;O	C43H78O5K	[M+K]+
713.5475705	713.5481	0.0005	0.7007	TG O-40:3	C43H78O5K	[M+K]+
713.5568792	713.5562	0.0007	0.9810	TG 38:1;O3	C41H76O9	[M+H]+
713.5568792	713.5562	0.0007	0.9810	MGDG 32:0	C41H78O10	[M+H-H2O]+
713.5568792	713.5568	0.0001	0.1401	EPC 36:0;O2	C38H79N2O6PNa	[M+Na]+
713.5568792	713.5568	0.0001	0.1401	SM 33:0;O2	C38H79N2O6PNa	[M+Na]+
716.3146208	716.317	0.0024	3.3505	PS 29:8;O	C35H52NO11PNa	[M+Na]+
717.3224878	717.3222	0.0003	0.4182	PI 23:3;O	C32H55O14PNa	[M+Na]+
	717.5905	0.0008	1.1148	EPC 38:1;O2	C40H81N2O6P	[M+H]+

717.5896963						
717.5896963	717.5905	0.0008	1.1148	SM 35:1;O2	C40H81N2O6P	[M+H]+
	717.5905	0.0008	1.1148	EPC 38:0;O3	C40H83N2O7P	[M+H-H2O]+
717.5896963						
717.5896963	717.5905	0.0008	1.1148	SM 35:0;O3	C40H83N2O7P	[M+H-H2O]+
	717.5905	0.0008	1.1148	CerP 40:2;O2	C40H78NO6P	[M+NH4]+
717.5896963						
717.5896963	717.5905	0.0008	1.1148	LPC O-32:3	C40H78NO6P	[M+NH4]+
	717.5905	0.0002	0.2787	EPC 38:1;O2	C40H81N2O6P	[M+H]+
717.5906498						
717.5906498	717.5905	0.0002	0.2787	SM 35:1;O2	C40H81N2O6P	[M+H]+
	717.5905	0.0002	0.2787	EPC 38:0;O3	C40H83N2O7P	[M+H-H2O]+
717.5906498						
717.5906498	717.5905	0.0002	0.2787	SM 35:0;O3	C40H83N2O7P	[M+H-H2O]+
	717.5905	0.0002	0.2787	CerP 40:2;O2	C40H78NO6P	[M+NH4]+
717.5906498						
717.5906498	717.5905	0.0002	0.2787	LPC O-32:3	C40H78NO6P	[M+NH4]+
	718.3327	0.0033	4.5940	PS 29:7;O	C35H54NO11PNa	[M+Na]+
718.3293661						
718.3307058	718.3327	0.0020	2.7842	PS 29:7;O	C35H54NO11PNa	[M+Na]+
	718.5956	0.0021	2.9224	ACer 42:1;O4	C42H81NO6Na	[M+Na]+
718.5934758						
718.5934758	718.5956	0.0021	2.9224	Cer 42:2;O5	C42H81NO6Na	[M+Na]+
	719.3378	0.0002	0.2780	PI 23:2;O	C32H57O14PNa	[M+Na]+
719.3379728						
719.6062048	719.6061	0.0001	0.1390	EPC 38:0;O2	C40H83N2O6P	[M+H]+
	719.6061	0.0001	0.1390	SM 35:0;O2	C40H83N2O6P	[M+H]+
719.6062048						
719.6062048	719.6061	0.0001	0.1390	CerP 40:1;O2	C40H80NO6P	[M+NH4]+
	719.6061	0.0001	0.1390	LPC O-32:2	C40H80NO6P	[M+NH4]+
719.6062048						
720.3460522	720.3483	0.0023	3.1929	PS 29:6;O	C35H56NO11PNa	[M+Na]+
	721.3477	0.0015	2.0794	BMP 30:6	C36H59O10PK	[M+K]+
721.3492178						
721.3492178	721.3477	0.0015	2.0794	LPG 30:7;O	C36H59O10PK	[M+K]+
	721.3477	0.0015	2.0794	PG 30:6	C36H59O10PK	[M+K]+

721.3492178						
721.3492178	721.3477	0.0015	2.0794	PG O-30:7;O	C36H59O10PK	[M+K]+
	721.3535	0.0031	4.2975	PI 23:1;O	C32H59O14PNa	[M+Na]+
721.3504016						
721.3504016	721.3477	0.0027	3.7430	BMP 30:6	C36H59O10PK	[M+K]+
	721.3477	0.0027	3.7430	LPG 30:7;O	C36H59O10PK	[M+K]+
721.3504016						
721.3504016	721.3477	0.0027	3.7430	PG 30:6	C36H59O10PK	[M+K]+
721.3504016	721.3477	0.0027	3.7430	PG O-30:7;O	C36H59O10PK	[M+K]+
722.3523537	722.3511	0.0013	1.7997	ST 30:7;O6;HexNAc	C38H53NO11Na	[M+Na]+
	722.3511	0.0012	1.6612	PI 24:5;O	C33H53O14P	[M+NH4]+
722.3523537						
722.3523537	722.3512	0.0011	1.5228	HexCer 29:6;O6	C35H57NO12K	[M+K]+
	722.3512	0.0011	1.5228	ST 27:2;O7;HexNAc	C35H57NO12K	[M+K]+
722.3523537						
724.5704276	724.5698	0.0006	0.8281	CAR 33:0;O4	C40H79NO8Na	[M+Na]+
	724.5698	0.0006	0.8281	HexCer 34:0;O2	C40H79NO8Na	[M+Na]+
724.5704276						
729.2643544	729.2647	0.0003	0.4114	PIP 21:4	C30H50O16P2	[M+H]+
729.2643544	729.2647	0.0003	0.4114	PIP 21:3;O	C30H52O17P2	[M+H-H2O]+
	729.2648	0.0004	0.5485	PI 23:5;O	C32H51O14PK	[M+K]+
729.2643544						
729.4834928	729.483	0.0005	0.6854	PA O-38:6	C41H71O7PNa	[M+Na]+
	729.4831	0.0004	0.5483	LPG O-32:2	C38H75O8PK	[M+K]+
729.4834928						
729.4834928	729.4831	0.0004	0.5483	PA 35:0	C38H75O8PK	[M+K]+
	729.4831	0.0004	0.5483	PA O-35:1;O	C38H75O8PK	[M+K]+
729.4834928						
729.5591099	729.5582	0.0009	1.2336	CE 20:3;O	C47H78O3K	[M+K]+
	729.5624	0.0015	2.0560	HexCer 34:3;O3	C40H73NO9	[M+NH4]+
729.5608669						
729.5789339	729.5793	0.0003	0.4112	PA O-39:2	C42H81O7P	[M+H]+
	729.5793	0.0003	0.4112	PA 39:0	C42H83O8P	[M+H-H2O]+
729.5789339						
729.5789339	729.5793	0.0003	0.4112	PA O-39:1;O	C42H83O8P	[M+H-H2O]+
	729.5792	0.0003	0.4112	CE 20:3;O2	C47H78O4Na	[M+Na]+

729.5789339						
729.5789339	729.5792	0.0003	0.4112	DG O-44:8	C47H78O4Na	[M+Na]+
	729.5794	0.0004	0.5483	DG 41:2	C44H82O5K	[M+K]+
729.5789339						
729.5789339	729.5794	0.0004	0.5483	DG O-41:3;O	C44H82O5K	[M+K]+
	729.5794	0.0004	0.5483	TG O-41:2	C44H82O5K	[M+K]+
729.5789339						
729.5825625	729.5816	0.0009	1.2336	CE 22:6;O2	C49H76O4	[M+H]+
	729.5816	0.0009	1.2336	DG O-46:11	C49H76O4	[M+H]+
729.5825625						
729.5825625	729.5816	0.0009	1.2336	CE 22:5;O3	C49H78O5	[M+H-H2O]+
	729.5816	0.0009	1.2336	DG 46:9	C49H78O5	[M+H-H2O]+
729.5825625						
729.5825625	729.5816	0.0009	1.2336	DG O-46:10;O	C49H78O5	[M+H-H2O]+
	729.5816	0.0009	1.2336	TG O-46:9	C49H78O5	[M+H-H2O]+
729.5825625						
731.2791179	731.2803	0.0012	1.6410	PIP 21:3	C30H52O16P2	[M+H]+
	731.2803	0.0012	1.6410	PIP 21:2;O	C30H54O17P2	[M+H-H2O]+
731.2791179						
731.2791179	731.2805	0.0013	1.7777	PI 23:4;O	C32H53O14PK	[M+K]+
	731.2803	0.0004	0.5470	PIP 21:3	C30H52O16P2	[M+H]+
731.2807116						
731.2807116	731.2803	0.0004	0.5470	PIP 21:2;O	C30H54O17P2	[M+H-H2O]+
	731.2805	0.0003	0.4102	PI 23:4;O	C32H53O14PK	[M+K]+
731.2807116						
731.4977136	731.497	0.0007	0.9569	EPC 36:4;O5	C38H71N2O9P	[M+H]+
	731.497	0.0007	0.9569	SM 33:4;O5	C38H71N2O9P	[M+H]+
731.4977136						
731.4977136	731.497	0.0007	0.9569	EPC 36:3;O6	C38H73N2O10P	[M+H-H2O]+
	731.497	0.0007	0.9569	SM 33:3;O6	C38H73N2O10P	[M+H-H2O]+
731.4977136						
731.4977136	731.4986	0.0009	1.2304	PA O-38:5	C41H73O7PNa	[M+Na]+
	731.497	0.0007	0.9569	CerP 38:5;O5	C38H68NO9P	[M+NH4]+
731.4977136						
731.4977136	731.497	0.0007	0.9569	LPS 32:4	C38H68NO9P	[M+NH4]+
	731.497	0.0007	0.9569	LPS O-32:5;O	C38H68NO9P	[M+NH4]+
731.4977136						
731.4977136	731.497	0.0007	0.9569	PC 30:4;O	C38H68NO9P	[M+NH4]+

	731.497	0.0007	0.9569	PE 33:4;O	C38H68NO9P	[M+NH4]+
731.4977136						
731.4977136	731.497	0.0007	0.9569	PS O-32:4	C38H68NO9P	[M+NH4]+
	731.4988	0.0010	1.3671	LPG O-32:1	C38H77O8PK	[M+K]+
731.4977136						
731.4977136	731.4988	0.0010	1.3671	PA O-35:0;O	C38H77O8PK	[M+K]+
	731.501	0.0011	1.5038	PA O-40:8	C43H71O7P	[M+H]+
731.5020751						
731.5020751	731.501	0.0011	1.5038	PA 40:6	C43H73O8P	[M+H-H2O]+
	731.501	0.0011	1.5038	PA O-40:7;O	C43H73O8P	[M+H-H2O]+
731.5020751						
731.5020751	731.5011	0.0009	1.2303	CE 18:4;O3	C45H72O5K	[M+K]+
	731.5011	0.0009	1.2303	DG 42:8	C45H72O5K	[M+K]+
731.5020751						
731.5020751	731.5011	0.0009	1.2303	DG O-42:9;O	C45H72O5K	[M+K]+
	731.5011	0.0009	1.2303	TG O-42:8	C45H72O5K	[M+K]+
731.5020751						
732.4975700	732.4963	0.0013	1.7748	PC O-34:8	C42H70NO7P	[M+H]+
	732.4963	0.0013	1.7748	PE O-37:8	C42H70NO7P	[M+H]+
732.4975700						
732.4975700	732.4963	0.0013	1.7748	LPC 34:7;O	C42H72NO8P	[M+H-H2O]+
	732.4963	0.0013	1.7748	PC 34:6	C42H72NO8P	[M+H-H2O]+
732.4975700						
732.4975700	732.4963	0.0013	1.7748	PC O-34:7;O	C42H72NO8P	[M+H-H2O]+
	732.4963	0.0013	1.7748	PE 37:6	C42H72NO8P	[M+H-H2O]+
732.4975700						
732.4975700	732.4963	0.0013	1.7748	PE O-37:7;O	C42H72NO8P	[M+H-H2O]+
	732.4963	0.0013	1.7748	PA O-39:9	C42H67O7P	[M+NH4]+
732.4975700						
732.5015360	732.5021	0.0006	0.8191	HexCer 34:4;O3	C40H71NO9Na	[M+Na]+
	732.5021	0.0006	0.8191	LPI O-26:0;O	C35H71O12P	[M+NH4]+
732.5015360						
733.5138512	733.5126	0.0012	1.6360	EPC 36:3;O5	C38H73N2O9P	[M+H]+
	733.5126	0.0012	1.6360	SM 33:3;O5	C38H73N2O9P	[M+H]+
733.5138512						
733.5138512	733.5126	0.0012	1.6360	EPC 36:2;O6	C38H75N2O10P	[M+H-H2O]+
	733.5126	0.0012	1.6360	SM 33:2;O6	C38H75N2O10P	[M+H-H2O]+

733.5138512						
733.5138512	733.5143	0.0004	0.5453	PA O-38:4	C41H75O7PNa	[M+Na]+
	733.5144	0.0006	0.8180	LPG O-32:0	C38H79O8PK	[M+K]+
733.5138512						
734.3043284	734.3066	0.0023	3.1322	PS 29:7;O	C35H54NO11PK	[M+K]+
	735.5042	0.0002	0.2719	MGDG 33:5	C42H70O10	[M+H]+
735.5039363						
735.5039363	735.5048	0.0008	1.0877	EPC 37:4;O3	C39H73N2O7PNa	[M+Na]+
	735.5048	0.0008	1.0877	SM 34:4;O3	C39H73N2O7PNa	[M+Na]+
735.5039363						
735.5294586	735.5283	0.0012	1.6315	EPC 36:2;O5	C38H75N2O9P	[M+H]+
	735.5283	0.0012	1.6315	SM 33:2;O5	C38H75N2O9P	[M+H]+
735.5294586						
735.5294586	735.5299	0.0004	0.5438	PA O-38:3	C41H77O7PNa	[M+Na]+
	735.5299	0.0005	0.6798	PA O-38:3	C41H77O7PNa	[M+Na]+
735.5304427						
737.5455255	737.5456	0.0000	0.0000	PA O-38:2	C41H79O7PNa	[M+Na]+
	737.5562	0.0003	0.4067	TG 40:3;O3	C43H76O9	[M+H]+
737.5558890						
737.5558890	737.5562	0.0003	0.4067	MGDG 34:2	C43H78O10	[M+H-H2O]+
	737.5568	0.0009	1.2202	EPC 38:2;O2	C40H79N2O6PNa	[M+Na]+
737.5558890						
737.5558890	737.5568	0.0009	1.2202	SM 35:2;O2	C40H79N2O6PNa	[M+Na]+
	738.5392	0.0000	0.0000	EPC 35:2;O5	C37H73N2O9P	[M+NH4]+
738.5392080						
738.5392080	738.5392	0.0000	0.0000	SM 32:2;O5	C37H73N2O9P	[M+NH4]+
	739.5678	0.0016	2.1634	HexCer 32:0;O5	C38H75NO11	[M+NH4]+
739.5693832						
739.5716497	739.5719	0.0002	0.2704	TG 40:2;O3	C43H78O9	[M+H]+
	739.5719	0.0002	0.2704	MGDG 34:1	C43H80O10	[M+H-H2O]+
739.5716497						
739.5716497	739.5724	0.0008	1.0817	EPC 38:1;O2	C40H81N2O6PNa	[M+Na]+
	739.5724	0.0008	1.0817	SM 35:1;O2	C40H81N2O6PNa	[M+Na]+
739.5716497						
741.3218693	741.3222	0.0003	0.4047	PI 25:5;O	C34H55O14PNa	[M+Na]+
	743.3321	0.0020	2.6906	PG 32:9	C38H57O10PK	[M+K]+
743.3340952						
746.2895416	746.2912	0.0017	2.2779	PIP 21:4	C30H50O16P2	[M+NH4]+

746.2912600	746.2912	0.0000	0.0000	PIP 21:4	C30H50O16P2	[M+NH4]+
750.5769684	750.5772	0.0002	0.2665	CerP 42:2;O2	C42H82NO6PNa	[M+Na]+
750.5769684	750.5772	0.0002	0.2665	LPC O-34:3	C42H82NO6PNa	[M+Na]+
750.5769684	750.5797	0.0027	3.5972	ACer 46:5;O2	C46H81NO4K	[M+K]+
750.5769684	750.5797	0.0027	3.5972	Cer 46:6;O3	C46H81NO4K	[M+K]+
751.5807613	751.5831	0.0023	3.0602	ACer 43:5;O6	C43H75NO8	[M+NH4]+
751.5807613	751.5831	0.0023	3.0602	HexCer 37:5;O2	C43H75NO8	[M+NH4]+
751.5807613	751.579	0.0018	2.3950	CE 23:5	C50H80O2K	[M+K]+
752.3392717	752.3382	0.0011	1.4621	PIP 21:1	C30H56O16P2	[M+NH4]+
752.3407802	752.3382	0.0026	3.4559	PIP 21:1	C30H56O16P2	[M+NH4]+
752.3407802	752.344	0.0033	4.3863	SHexCer 30:6;O2	C36H59NO11SK	[M+K]+
752.5926977	752.5928	0.0001	0.1329	CerP 42:1;O2	C42H84NO6PNa	[M+Na]+
752.5926977	752.5928	0.0001	0.1329	LPC O-34:2	C42H84NO6PNa	[M+Na]+
754.3541630	754.3538	0.0004	0.5303	IPC 29:6;O4	C35H58NO13PNa	[M+Na]+
754.3541630	754.3538	0.0003	0.3977	PIP 21:0	C30H58O16P2	[M+NH4]+
755.4983179	755.4986	0.0003	0.3971	PA O-40:7	C43H73O7PNa	[M+Na]+
755.4983179	755.4988	0.0004	0.5295	LPG O-34:3	C40H77O8PK	[M+K]+
755.4983179	755.4988	0.0004	0.5295	PA 37:1	C40H77O8PK	[M+K]+
755.4983179	755.4988	0.0004	0.5295	PA O-37:2;O	C40H77O8PK	[M+K]+
756.5878945	756.5878	0.0001	0.1322	CerP 41:0;O3	C41H84NO7PNa	[M+Na]+
756.5878945	756.5878	0.0001	0.1322	LPC 33:0	C41H84NO7PNa	[M+Na]+
756.5878945	756.5878	0.0001	0.1322	LPC O-33:1;O	C41H84NO7PNa	[M+Na]+
756.5878945	756.5878	0.0001	0.1322	PC O-33:0	C41H84NO7PNa	[M+Na]+
756.5878945	756.5878	0.0001	0.1322	PE O-36:0	C41H84NO7PNa	[M+Na]+
	757.296	0.0003	0.3961	PIP 23:4	C32H54O16P2	[M+H]+

757.2957156						
757.2957156	757.296	0.0003	0.3961	PIP 23:3;O	C32H56O17P2	[M+H-H2O]+
	757.2961	0.0004	0.5282	PI 25:5;O	C34H55O14PK	[M+K]+
757.2957156						
757.5914582	757.5937	0.0022	2.9039	HexCer 36:3;O3	C42H77NO9	[M+NH4]+
	757.5895	0.0019	2.5080	CE 22:3;O	C49H82O3K	[M+K]+
757.5914582						
759.6088877	759.6093	0.0004	0.5266	HexCer 36:2;O3	C42H79NO9	[M+NH4]+
	762.5392	0.0004	0.5246	EPC 37:4;O5	C39H73N2O9P	[M+NH4]+
762.5387743						
762.5387743	762.5392	0.0004	0.5246	SM 34:4;O5	C39H73N2O9P	[M+NH4]+
	762.5408	0.0002	0.2623	CerP 42:4;O3	C42H78NO7PNa	[M+Na]+
762.5409885						
762.5409885	762.5408	0.0002	0.2623	LPC 34:4	C42H78NO7PNa	[M+Na]+
	762.5408	0.0002	0.2623	LPC O-34:5;O	C42H78NO7PNa	[M+Na]+
762.5409885						
762.5409885	762.5408	0.0002	0.2623	PC O-34:4	C42H78NO7PNa	[M+Na]+
	762.5408	0.0002	0.2623	PE O-37:4	C42H78NO7PNa	[M+Na]+
762.5409885						
762.6009366	762.6007	0.0002	0.2623	CerP 42:1;O4	C42H84NO8P	[M+H]+
	762.6007	0.0002	0.2623	LPC 34:1;O	C42H84NO8P	[M+H]+
762.6009366						
762.6009366	762.6007	0.0002	0.2623	PC 34:0	C42H84NO8P	[M+H]+
	762.6007	0.0002	0.2623	PC O-34:1;O	C42H84NO8P	[M+H]+
762.6009366						
762.6009366	762.6007	0.0002	0.2623	PE 37:0	C42H84NO8P	[M+H]+
	762.6007	0.0002	0.2623	PE O-37:1;O	C42H84NO8P	[M+H]+
762.6009366						
762.6009366	762.6007	0.0002	0.2623	CerP 42:0;O5	C42H86NO9P	[M+H-H2O]+
	762.6007	0.0002	0.2623	ACer 47:6;O3	C47H81NO5Na	[M+Na]+
762.6009366						
762.6009366	762.6007	0.0002	0.2623	PA 39:1	C42H81O8P	[M+NH4]+
	762.6007	0.0002	0.2623	PA O-39:2;O	C42H81O8P	[M+NH4]+
762.6009366						
762.6009366	762.6008	0.0001	0.1311	ACer 44:1;O4	C44H85NO6K	[M+K]+
	762.6008	0.0001	0.1311	Cer 44:2;O5	C44H85NO6K	[M+K]+
762.6009366						
764.5562275	764.5565	0.0002	0.2616	CerP 42:3;O3	C42H80NO7PNa	[M+Na]+

	764.5565	0.0002	0.2616	LPC 34:3	C42H80NO7PNa	[M+Na]+
764.5562275						
764.5562275	764.5565	0.0002	0.2616	LPC O-34:4;O	C42H80NO7PNa	[M+Na]+
	764.5565	0.0002	0.2616	PC O-34:3	C42H80NO7PNa	[M+Na]+
764.5562275						
764.5562275	764.5565	0.0002	0.2616	PE O-37:3	C42H80NO7PNa	[M+Na]+
	765.5582	0.0011	1.4369	CE 23:6;O	C50H78O3K	[M+K]+
765.5593343						
765.5606543	765.5624	0.0017	2.2206	HexCer 37:6;O3	C43H73NO9	[M+NH4]+
	766.3538	0.0023	3.0012	PIP 22:1	C31H58O16P2	[M+NH4]+
766.3561075						
766.5719673	766.5721	0.0001	0.1305	CerP 42:2;O3	C42H82NO7PNa	[M+Na]+
	766.5721	0.0001	0.1305	LPC 34:2	C42H82NO7PNa	[M+Na]+
766.5719673						
766.5719673	766.5721	0.0001	0.1305	LPC O-34:3;O	C42H82NO7PNa	[M+Na]+
	766.5721	0.0001	0.1305	PC O-34:2	C42H82NO7PNa	[M+Na]+
766.5719673						
766.5719673	766.5721	0.0001	0.1305	PE O-37:2	C42H82NO7PNa	[M+Na]+
	767.3631	0.0022	2.8670	SHexCer 28:6;O6	C34H55NO15S	[M+NH4]+
767.3608575						
767.5754068	767.578	0.0026	3.3873	HexCer 37:5;O3	C43H75NO9	[M+NH4]+
	767.5739	0.0015	1.9542	CE 23:5;O	C50H80O3K	[M+K]+
767.5754068						
770.6046728	770.6058	0.0011	1.4274	CerP 44:3;O3	C44H84NO7P	[M+H]+
	770.6058	0.0011	1.4274	PC O-36:3	C44H84NO7P	[M+H]+
770.6046728						
770.6046728	770.6058	0.0011	1.4274	PE O-39:3	C44H84NO7P	[M+H]+
	770.6058	0.0011	1.4274	CerP 44:2;O4	C44H86NO8P	[M+H-H2O]+
770.6046728						
770.6046728	770.6058	0.0011	1.4274	PC 36:1	C44H86NO8P	[M+H-H2O]+
	770.6058	0.0011	1.4274	PC O-36:2;O	C44H86NO8P	[M+H-H2O]+
770.6046728						
770.6046728	770.6058	0.0011	1.4274	PE 39:1	C44H86NO8P	[M+H-H2O]+
	770.6058	0.0011	1.4274	PE O-39:2;O	C44H86NO8P	[M+H-H2O]+
770.6046728						
770.6046728	770.6034	0.0013	1.6870	CerP 42:0;O3	C42H86NO7PNa	[M+Na]+
	770.6034	0.0013	1.6870	LPC 34:0	C42H86NO7PNa	[M+Na]+

770.6046728						
770.6046728	770.6034	0.0013	1.6870	LPC O-34:1;O	C42H86NO7PNa	[M+Na]+
	770.6034	0.0013	1.6870	PC O-34:0	C42H86NO7PNa	[M+Na]+
770.6046728						
770.6046728	770.6034	0.0013	1.6870	PE O-37:0	C42H86NO7PNa	[M+Na]+
	770.6058	0.0011	1.4274	PA O-41:4	C44H81O7P	[M+NH4]+
770.6046728						
770.6046728	770.6059	0.0013	1.6870	ACer 46:3;O3	C46H85NO5K	[M+K]+
	770.6059	0.0013	1.6870	Cer 46:4;O4	C46H85NO5K	[M+K]+
770.6046728						
771.6082510	771.6093	0.0011	1.4256	HexCer 37:3;O3	C43H79NO9	[M+NH4]+
	771.6109	0.0005	0.6480	DG 43:3;O2	C46H84O7Na	[M+Na]+
771.6104393						
771.6104393	771.6109	0.0005	0.6480	TG 43:2;O	C46H84O7Na	[M+Na]+
	771.6109	0.0005	0.6480	TG O-43:3;O2	C46H84O7Na	[M+Na]+
771.6104393						
771.6104393	771.6093	0.0011	1.4256	HexCer 37:3;O3	C43H79NO9	[M+NH4]+
	772.3069	0.0000	0.0000	PIP 23:5	C32H52O16P2	[M+NH4]+
772.3069297						
772.3069297	772.307	0.0001	0.1295	IPC 28:6;O5	C34H56NO14PK	[M+K]+
	774.3225	0.0001	0.1291	PIP 23:4	C32H54O16P2	[M+NH4]+
774.3224522						
774.3224522	774.3227	0.0002	0.2583	IPC 28:5;O5	C34H58NO14PK	[M+K]+
	776.3288	0.0004	0.5152	SHexCer 28:4;O5	C34H59NO14SK	[M+K]+
776.3283728						
776.4835510	776.4837	0.0001	0.1288	CerP 41:6;O5	C41H72NO9PNa	[M+Na]+
	776.4837	0.0001	0.1288	PC 33:5;O	C41H72NO9PNa	[M+Na]+
776.4835510						
776.4835510	776.4837	0.0001	0.1288	PE 36:5;O	C41H72NO9PNa	[M+Na]+
	776.4837	0.0001	0.1288	PS O-35:5	C41H72NO9PNa	[M+Na]+
776.4835510						
776.4835510	776.4838	0.0003	0.3864	CerP 38:1;O6	C38H76NO10PK	[M+K]+
	776.4838	0.0003	0.3864	LPS 32:0;O	C38H76NO10PK	[M+K]+
776.4835510						
776.4835510	776.4838	0.0003	0.3864	PS O-32:0;O	C38H76NO10PK	[M+K]+
	776.5928	0.0001	0.1288	CerP 44:3;O2	C44H84NO6PNa	[M+Na]+
776.5926986						
777.5961410	777.5946	0.0015	1.9290	CE 25:6	C52H82O2K	[M+K]+

	778.6085	0.0002	0.2569	CerP 44:2;O2	C44H86NO6PNa	[M+Na]+
778.6082688						
779.6119065	779.6103	0.0016	2.0523	CE 25:5	C52H84O2K	[M+K]+
	780.3695	0.0032	4.1006	PIP 23:1	C32H60O16P2	[M+NH4]+
780.3726750						
780.3726750	780.3753	0.0027	3.4599	SHexCer 32:6;O2	C38H63NO11SK	[M+K]+
	780.3696	0.0031	3.9725	IPC 28:2;O5	C34H64NO14PK	[M+K]+
780.3726750						
780.5878989	780.5878	0.0001	0.1281	CerP 43:2;O3	C43H84NO7PNa	[M+Na]+
	780.5878	0.0001	0.1281	PC O-35:2	C43H84NO7PNa	[M+Na]+
780.5878989						
780.5878989	780.5878	0.0001	0.1281	PE O-38:2	C43H84NO7PNa	[M+Na]+
	781.5895	0.0002	0.2559	CE 24:5;O	C51H82O3K	[M+K]+
781.5893424						
781.5914233	781.5937	0.0022	2.8148	HexCer 38:5;O3	C44H77NO9	[M+NH4]+
	781.5895	0.0019	2.4309	CE 24:5;O	C51H82O3K	[M+K]+
781.5914233						
784.6089281	784.6086	0.0004	0.5098	ACer 48:6;O6	C48H83NO8	[M+H-H2O]+
	784.6086	0.0004	0.5098	HexCer 42:6;O2	C48H83NO8	[M+H-H2O]+
784.6089281						
784.6089281	784.6086	0.0004	0.5098	DG 45:8;O2	C48H78O7	[M+NH4]+
	784.6086	0.0004	0.5098	TG 45:7;O	C48H78O7	[M+NH4]+
784.6089281						
784.6089281	784.6086	0.0004	0.5098	TG O-45:8;O2	C48H78O7	[M+NH4]+
	784.645	0.0003	0.3823	ACer 49:6;O4	C49H85NO6	[M+H]+
784.6446581						
784.6446581	784.645	0.0003	0.3823	ACer 49:5;O5	C49H87NO7	[M+H-H2O]+
	784.645	0.0003	0.3823	Cer 49:6;O6	C49H87NO7	[M+H-H2O]+
784.6446581						
784.6446581	784.645	0.0003	0.3823	CE 22:3;O4	C49H82O6	[M+NH4]+
	784.645	0.0003	0.3823	DG 46:7;O	C49H82O6	[M+NH4]+
784.6446581						
784.6446581	784.645	0.0003	0.3823	DG O-46:8;O2	C49H82O6	[M+NH4]+
	784.645	0.0003	0.3823	TG 46:6	C49H82O6	[M+NH4]+
784.6446581						
784.6446581	784.645	0.0003	0.3823	TG O-46:7;O	C49H82O6	[M+NH4]+
	788.3382	0.0005	0.6342	PIP 24:4	C33H56O16P2	[M+NH4]+

788.3376916						
788.3376916	788.3383	0.0006	0.7611	IPC 29:5;O5	C35H60NO14PK	[M+K]+
	788.5565	0.0002	0.2536	CerP 44:5;O3	C44H80NO7PNa	[M+Na]+
788.5562539						
788.5562539	788.5565	0.0002	0.2536	PC O-36:5	C44H80NO7PNa	[M+Na]+
	788.5565	0.0002	0.2536	PE O-39:5	C44H80NO7PNa	[M+Na]+
788.5562539						
788.5562539	788.5566	0.0004	0.5073	CerP 41:0;O4	C41H84NO8PK	[M+K]+
	788.5566	0.0004	0.5073	LPC 33:0;O	C41H84NO8PK	[M+K]+
788.5562539						
788.5562539	788.5566	0.0004	0.5073	PC O-33:0;O	C41H84NO8PK	[M+K]+
	788.5566	0.0004	0.5073	PE O-36:0;O	C41H84NO8PK	[M+K]+
788.5562539						
789.5592847	789.5616	0.0023	2.9130	PG O-35:0;O	C41H83O10PNa	[M+Na]+
	789.5616	0.0011	1.3932	PG O-35:0;O	C41H83O10PNa	[M+Na]+
789.5604805						
790.5719182	790.5721	0.0002	0.2530	CerP 44:4;O3	C44H82NO7PNa	[M+Na]+
	790.5721	0.0002	0.2530	PC O-36:4	C44H82NO7PNa	[M+Na]+
790.5719182						
790.5719182	790.5721	0.0002	0.2530	PE O-39:4	C44H82NO7PNa	[M+Na]+
	791.5739	0.0015	1.8950	CE 25:7;O	C52H80O3K	[M+K]+
791.5753994						
792.3721321	792.3695	0.0026	3.2813	PIP 24:2	C33H60O16P2	[M+NH4]+
	792.3696	0.0025	3.1551	IPC 29:3;O5	C35H64NO14PK	[M+K]+
792.3721321						
792.5419526	792.5409	0.0011	1.3879	TG 45:11;O2	C48H70O8	[M+NH4]+
	792.5409	0.0011	1.3879	TG O-45:12;O3	C48H70O8	[M+NH4]+
792.5419526						
792.5881219	792.5878	0.0004	0.5047	CerP 44:3;O3	C44H84NO7PNa	[M+Na]+
	792.5878	0.0004	0.5047	PC O-36:3	C44H84NO7PNa	[M+Na]+
792.5881219						
792.5881219	792.5878	0.0004	0.5047	PE O-39:3	C44H84NO7PNa	[M+Na]+
	792.5902	0.0004	0.5047	CerP 46:6;O3	C46H82NO7P	[M+H]+
792.5905171						
792.5905171	792.5902	0.0004	0.5047	PC O-38:6	C46H82NO7P	[M+H]+
	792.5902	0.0004	0.5047	PE O-41:6	C46H82NO7P	[M+H]+
792.5905171						
792.5905171	792.5902	0.0004	0.5047	CerP 46:5;O4	C46H84NO8P	[M+H-H2O]+

	792.5902	0.0004	0.5047	PC 38:4	C46H84NO8P	[M+H-H2O]+
792.5905171						
792.5905171	792.5902	0.0004	0.5047	PC O-38:5;O	C46H84NO8P	[M+H-H2O]+
	792.5902	0.0004	0.5047	PE 41:4	C46H84NO8P	[M+H-H2O]+
792.5905171						
792.5905171	792.5902	0.0004	0.5047	PE O-41:5;O	C46H84NO8P	[M+H-H2O]+
	792.5902	0.0004	0.5047	PA O-43:7	C46H79O7P	[M+NH4]+
792.5905171						
792.5905171	792.5903	0.0002	0.2523	ACer 48:6;O3	C48H83NO5K	[M+K]+
	793.5466	0.0025	3.1504	EPC 40:4;O4	C42H79N2O8PNa	[M+Na]+
793.5441614						
793.5441614	793.5466	0.0025	3.1504	SM 37:4;O4	C42H79N2O8PNa	[M+Na]+
	793.542	0.0021	2.6464	Hex2Cer 28:2;O2	C40H73NO13	[M+NH4]+
793.5441614						
793.5870105	793.5854	0.0016	2.0162	EPC 43:6;O3	C45H81N2O7P	[M+H]+
	793.5854	0.0016	2.0162	SM 40:6;O3	C45H81N2O7P	[M+H]+
793.5870105						
793.5870105	793.5854	0.0016	2.0162	EPC 43:5;O4	C45H83N2O8P	[M+H-H2O]+
	793.5854	0.0016	2.0162	SM 40:5;O4	C45H83N2O8P	[M+H-H2O]+
793.5870105						
793.5870105	793.5854	0.0016	2.0162	PC O-37:7	C45H78NO7P	[M+NH4]+
	793.5854	0.0016	2.0162	PE O-40:7	C45H78NO7P	[M+NH4]+
793.5870105						
793.5917922	793.5937	0.0019	2.3942	HexCer 39:6;O3	C45H77NO9	[M+NH4]+
	793.5895	0.0022	2.7722	CE 25:6;O	C52H82O3K	[M+K]+
793.5917922						
794.3873746	794.3851	0.0022	2.7694	PIP 24:1	C33H62O16P2	[M+NH4]+
	794.3853	0.0021	2.6436	IPC 29:2;O5	C35H66NO14PK	[M+K]+
794.3873746						
794.6034364	794.6034	0.0000	0.0000	CerP 44:2;O3	C44H86NO7PNa	[M+Na]+
	794.6034	0.0000	0.0000	PC O-36:2	C44H86NO7PNa	[M+Na]+
794.6034364						
794.6034364	794.6034	0.0000	0.0000	PE O-39:2	C44H86NO7PNa	[M+Na]+
	794.6868	0.0002	0.2517	ACer 48:2;O5	C48H91NO7	[M+H]+
794.6865832						
794.6865832	794.6868	0.0002	0.2517	Cer 48:3;O6	C48H91NO7	[M+H]+
	794.6868	0.0002	0.2517	ACer 48:1;O6	C48H93NO8	[M+H-H2O]+

794.6865832						
794.6865832	794.6868	0.0002	0.2517	HexCer 42:1;O2	C48H93NO8	[M+H-H2O]+
	794.6868	0.0002	0.2517	DG 45:3;O2	C48H88O7	[M+NH4]+
794.6865832						
794.6865832	794.6868	0.0002	0.2517	TG 45:2;O	C48H88O7	[M+NH4]+
	794.6868	0.0002	0.2517	TG O-45:3;O2	C48H88O7	[M+NH4]+
794.6865832						
795.3908831	795.3944	0.0035	4.4003	SHexCer 30:6;O6	C36H59NO15S	[M+NH4]+
	795.6093	0.0024	3.0166	HexCer 39:5;O3	C45H79NO9	[M+NH4]+
795.6069199						
795.6069199	795.6052	0.0017	2.1367	CE 25:5;O	C52H84O3K	[M+K]+
	795.6861	0.0033	4.1474	CE 25:2;O3	C52H90O5	[M+H]+
795.6893766						
795.6893766	795.6861	0.0033	4.1474	DG 49:6	C52H90O5	[M+H]+
	795.6861	0.0033	4.1474	DG O-49:7;O	C52H90O5	[M+H]+
795.6893766						
795.6893766	795.6861	0.0033	4.1474	TG O-49:6	C52H90O5	[M+H]+
	795.6861	0.0033	4.1474	CE 25:1;O4	C52H92O6	[M+H-H2O]+
795.6893766						
795.6893766	795.6861	0.0033	4.1474	DG 49:5;O	C52H92O6	[M+H-H2O]+
	795.6861	0.0033	4.1474	DG O-49:6;O2	C52H92O6	[M+H-H2O]+
795.6893766						
795.6893766	795.6861	0.0033	4.1474	TG 49:4	C52H92O6	[M+H-H2O]+
	795.6861	0.0033	4.1474	TG O-49:5;O	C52H92O6	[M+H-H2O]+
795.6893766						
796.3051893	796.3069	0.0017	2.1349	PIP 25:7	C34H52O16P2	[M+NH4]+
	796.395	0.0009	1.1301	PE 37:10;O	C42H64NO9PK	[M+K]+
796.3940970						
796.3940970	796.395	0.0009	1.1301	PS O-36:10	C42H64NO9PK	[M+K]+
	797.2885	0.0005	0.6271	PIP 23:3;O	C32H56O17P2Na	[M+Na]+
797.2879993						
797.6627971	797.663	0.0002	0.2507	DG 46:3;O	C49H90O6Na	[M+Na]+
	797.663	0.0002	0.2507	DG O-46:4;O2	C49H90O6Na	[M+Na]+
797.6627971						
797.6627971	797.663	0.0002	0.2507	TG 46:2	C49H90O6Na	[M+Na]+
	797.663	0.0002	0.2507	TG O-46:3;O	C49H90O6Na	[M+Na]+
797.6627971						
798.4658304	798.4668	0.0010	1.2524	SHexCer 32:2;O5	C38H71NO14S	[M+H]+

	798.4668	0.0010	1.2524	SHexCer 32:1;O6	C38H73NO15S	[M+H-H2O]+
798.4658304						
798.6255574	798.6242	0.0013	1.6278	ACer 49:6;O6	C49H85NO8	[M+H-H2O]+
	798.6242	0.0013	1.6278	HexCer 43:6;O2	C49H85NO8	[M+H-H2O]+
798.6255574						
798.6255574	798.6242	0.0013	1.6278	DG 46:8;O2	C49H80O7	[M+NH4]+
	798.6242	0.0013	1.6278	TG 46:7;O	C49H80O7	[M+NH4]+
798.6255574						
798.6255574	798.6242	0.0013	1.6278	TG O-46:8;O2	C49H80O7	[M+NH4]+
	798.6371	0.0008	1.0017	CerP 46:3;O3	C46H88NO7P	[M+H]+
798.6363542						
798.6363542	798.6371	0.0008	1.0017	PC O-38:3	C46H88NO7P	[M+H]+
	798.6371	0.0008	1.0017	PE O-41:3	C46H88NO7P	[M+H]+
798.6363542						
798.6363542	798.6371	0.0008	1.0017	CerP 46:2;O4	C46H90NO8P	[M+H-H2O]+
	798.6371	0.0008	1.0017	PC 38:1	C46H90NO8P	[M+H-H2O]+
798.6363542						
798.6363542	798.6371	0.0008	1.0017	PC O-38:2;O	C46H90NO8P	[M+H-H2O]+
	798.6371	0.0008	1.0017	PE 41:1	C46H90NO8P	[M+H-H2O]+
798.6363542						
798.6363542	798.6371	0.0008	1.0017	PE O-41:2;O	C46H90NO8P	[M+H-H2O]+
	798.6371	0.0008	1.0017	PA O-43:4	C46H85O7P	[M+NH4]+
798.6363542						
798.6363542	798.6372	0.0009	1.1269	ACer 48:3;O3	C48H89NO5K	[M+K]+
	798.6372	0.0009	1.1269	Cer 48:4;O4	C48H89NO5K	[M+K]+
798.6363542						
799.6409867	799.6406	0.0004	0.5002	HexCer 39:3;O3	C45H83NO9	[M+NH4]+
	800.5565	0.0005	0.6246	CerP 45:6;O3	C45H80NO7PNa	[M+Na]+
800.5569700						
800.5569700	800.5565	0.0005	0.6246	PC O-37:6	C45H80NO7PNa	[M+Na]+
	800.5565	0.0005	0.6246	PE O-40:6	C45H80NO7PNa	[M+Na]+
800.5569700						
800.5569700	800.5566	0.0004	0.4997	CerP 42:1;O4	C42H84NO8PK	[M+K]+
	800.5566	0.0004	0.4997	LPC 34:1;O	C42H84NO8PK	[M+K]+
800.5569700						
800.5569700	800.5566	0.0004	0.4997	PC 34:0	C42H84NO8PK	[M+K]+
	800.5566	0.0004	0.4997	PC O-34:1;O	C42H84NO8PK	[M+K]+

800.5569700						
800.5569700	800.5566	0.0004	0.4997	PE 37:0	C42H84NO8PK	[M+K]+
	800.5566	0.0004	0.4997	PE O-37:1;O	C42H84NO8PK	[M+K]+
800.5569700						
800.6526380	800.6528	0.0001	0.1249	CerP 46:2;O3	C46H90NO7P	[M+H]+
	800.6528	0.0001	0.1249	PC O-38:2	C46H90NO7P	[M+H]+
800.6526380						
800.6526380	800.6528	0.0001	0.1249	PE O-41:2	C46H90NO7P	[M+H]+
	800.6528	0.0001	0.1249	CerP 46:1;O4	C46H92NO8P	[M+H-H2O]+
800.6526380						
800.6526380	800.6528	0.0001	0.1249	PC 38:0	C46H92NO8P	[M+H-H2O]+
	800.6528	0.0001	0.1249	PC O-38:1;O	C46H92NO8P	[M+H-H2O]+
800.6526380						
800.6526380	800.6528	0.0001	0.1249	PE 41:0	C46H92NO8P	[M+H-H2O]+
	800.6528	0.0001	0.1249	PE O-41:1;O	C46H92NO8P	[M+H-H2O]+
800.6526380						
800.6526380	800.6528	0.0001	0.1249	PA O-43:3	C46H87O7P	[M+NH4]+
	800.6529	0.0002	0.2498	ACer 48:2;O3	C48H91NO5K	[M+K]+
800.6526380						
800.6526380	800.6529	0.0002	0.2498	Cer 48:3;O4	C48H91NO5K	[M+K]+
	802.3175	0.0021	2.6174	PIP 24:5;O	C33H54O17P2	[M+NH4]+
802.3153138						
802.3153138	802.3176	0.0023	2.8667	IPC 29:6;O6	C35H58NO15PK	[M+K]+
	802.4993	0.0001	0.1246	PC 35:6;O	C43H74NO9PNa	[M+Na]+
802.4992167						
802.4992167	802.4993	0.0001	0.1246	PE 38:6;O	C43H74NO9PNa	[M+Na]+
	802.4993	0.0001	0.1246	PS O-37:6	C43H74NO9PNa	[M+Na]+
802.4992167						
802.4992167	802.4995	0.0003	0.3738	CerP 40:2;O6	C40H78NO10PK	[M+K]+
	802.4995	0.0003	0.3738	LPS 34:1;O	C40H78NO10PK	[M+K]+
802.4992167						
802.4992167	802.4995	0.0003	0.3738	PS 34:0	C40H78NO10PK	[M+K]+
	802.4995	0.0003	0.3738	PS O-34:1;O	C40H78NO10PK	[M+K]+
802.4992167						
802.5002253	802.5017	0.0015	1.8692	PC 37:9;O	C45H72NO9P	[M+H]+
	802.5017	0.0015	1.8692	PE 40:9;O	C45H72NO9P	[M+H]+
802.5002253						
802.5002253	802.5017	0.0015	1.8692	PS O-39:9	C45H72NO9P	[M+H]+

	802.5017	0.0015	1.8692	PS 39:7	C45H74NO10P	[M+H-H2O]+
802.5002253						
802.5002253	802.5017	0.0015	1.8692	PS O-39:8;O	C45H74NO10P	[M+H-H2O]+
	802.4993	0.0009	1.1215	PC 35:6;O	C43H74NO9PNa	[M+Na]+
802.5002253						
802.5002253	802.4993	0.0009	1.1215	PE 38:6;O	C43H74NO9PNa	[M+Na]+
	802.4993	0.0009	1.1215	PS O-37:6	C43H74NO9PNa	[M+Na]+
802.5002253						
802.5002253	802.5017	0.0015	1.8692	PA 42:10;O	C45H69O9P	[M+NH4]+
	802.5017	0.0015	1.8692	PG O-39:11	C45H69O9P	[M+NH4]+
802.5002253						
802.5002253	802.4995	0.0007	0.8723	CerP 40:2;O6	C40H78NO10PK	[M+K]+
	802.4995	0.0007	0.8723	LPS 34:1;O	C40H78NO10PK	[M+K]+
802.5002253						
802.5002253	802.4995	0.0007	0.8723	PS 34:0	C40H78NO10PK	[M+K]+
	802.4995	0.0007	0.8723	PS O-34:1;O	C40H78NO10PK	[M+K]+
802.5002253						
802.6681900	802.6684	0.0002	0.2492	CerP 46:1;O3	C46H92NO7P	[M+H]+
	802.6684	0.0002	0.2492	PC O-38:1	C46H92NO7P	[M+H]+
802.6681900						
802.6681900	802.6684	0.0002	0.2492	PE O-41:1	C46H92NO7P	[M+H]+
	802.6684	0.0002	0.2492	CerP 46:0;O4	C46H94NO8P	[M+H-H2O]+
802.6681900						
802.6681900	802.6684	0.0002	0.2492	PC O-38:0;O	C46H94NO8P	[M+H-H2O]+
	802.6684	0.0002	0.2492	PE O-41:0;O	C46H94NO8P	[M+H-H2O]+
802.6681900						
802.6681900	802.6684	0.0002	0.2492	ACer 51:6;O2	C51H89NO4Na	[M+Na]+
	802.6684	0.0002	0.2492	PA O-43:2	C46H89O7P	[M+NH4]+
802.6681900						
802.6681900	802.6685	0.0003	0.3738	ACer 48:1;O3	C48H93NO5K	[M+K]+
	802.6685	0.0003	0.3738	Cer 48:2;O4	C48H93NO5K	[M+K]+
802.6681900						
803.5026320	803.5029	0.0002	0.2489	IPC 31:1;O5	C37H72NO14P	[M+NH4]+
	804.5021	0.0032	3.9776	IPC 35:4;O3	C41H74NO12P	[M+H]+
804.5053506						
804.5053506	804.5021	0.0032	3.9776	IPC 35:3;O4	C41H76NO13P	[M+H-H2O]+
	804.5021	0.0032	3.9776	LPI 32:5	C41H71O12P	[M+NH4]+

804.5053506						
804.5053506	804.5021	0.0032	3.9776	LPI O-32:6;O	C41H71O12P	[M+NH4]+
	804.5021	0.0032	3.9776	PI O-32:5	C41H71O12P	[M+NH4]+
804.5053506						
804.5053506	804.5023	0.0031	3.8533	HexCer 37:5;O4	C43H75NO10K	[M+K]+
	804.5725	0.0007	0.8700	CerP 41:0;O6	C41H84NO10PNa	[M+Na]+
804.5732351						
804.5869063	804.5878	0.0009	1.1186	CerP 45:4;O3	C45H84NO7PNa	[M+Na]+
	804.5878	0.0009	1.1186	PC O-37:4	C45H84NO7PNa	[M+Na]+
804.5869063						
804.5869063	804.5878	0.0009	1.1186	PE O-40:4	C45H84NO7PNa	[M+Na]+
	804.5861	0.0008	0.9943	EPC 40:4;O5	C42H79N2O9P	[M+NH4]+
804.5869063						
804.5869063	804.5861	0.0008	0.9943	SM 37:4;O5	C42H79N2O9P	[M+NH4]+
	806.3488	0.0007	0.8681	PIP 24:3;O	C33H58O17P2	[M+NH4]+
806.3480713						
806.3480713	806.3489	0.0008	0.9921	IPC 29:4;O6	C35H62NO15PK	[M+K]+
	806.6269	0.0006	0.7438	CerP 44:1;O5	C44H88NO9P	[M+H]+
806.6263709						
806.6263709	806.6269	0.0006	0.7438	PC 36:0;O	C44H88NO9P	[M+H]+
	806.6269	0.0006	0.7438	PE 39:0;O	C44H88NO9P	[M+H]+
806.6263709						
806.6263709	806.6269	0.0006	0.7438	PS O-38:0	C44H88NO9P	[M+H]+
	806.6269	0.0006	0.7438	CerP 44:0;O6	C44H90NO10P	[M+H-H2O]+
806.6263709						
806.6263709	806.6269	0.0005	0.6199	ACer 49:6;O4	C49H85NO6Na	[M+Na]+
	806.6269	0.0006	0.7438	PA 41:1;O	C44H85O9P	[M+NH4]+
806.6263709						
806.6263709	806.6269	0.0006	0.7438	PG O-38:2	C44H85O9P	[M+NH4]+
	806.6271	0.0007	0.8678	ACer 46:1;O5	C46H89NO7K	[M+K]+
806.6263709						
806.6263709	806.6271	0.0007	0.8678	Cer 46:2;O6	C46H89NO7K	[M+K]+
	808.6426	0.0040	4.9466	CerP 44:0;O5	C44H90NO9P	[M+H]+
808.6385789						
808.6385789	808.6426	0.0040	4.9466	ACer 49:5;O4	C49H87NO6Na	[M+Na]+
	808.6426	0.0040	4.9466	Cer 49:6;O5	C49H87NO6Na	[M+Na]+
808.6385789						
808.6385789	808.6426	0.0040	4.9466	PA 41:0;O	C44H87O9P	[M+NH4]+

808.6385789	808.6426	0.0040	4.9466	PG O-38:1	C44H87O9P	[M+NH4]+
809.6663692	809.6654	0.0010	1.2351	CE 25:3;O4	C52H88O6	[M+H]+
809.6663692	809.6654	0.0010	1.2351	DG 49:7;O	C52H88O6	[M+H]+
809.6663692	809.6654	0.0010	1.2351	DG O-49:8;O2	C52H88O6	[M+H]+
809.6663692	809.6654	0.0010	1.2351	TG 49:6	C52H88O6	[M+H]+
809.6663692	809.6654	0.0010	1.2351	TG O-49:7;O	C52H88O6	[M+H]+
809.6663692	809.6654	0.0010	1.2351	DG 49:6;O2	C52H90O7	[M+H-H2O]+
809.6663692	809.6654	0.0010	1.2351	TG 49:5;O	C52H90O7	[M+H-H2O]+
809.6663692	809.6654	0.0010	1.2351	TG O-49:6;O2	C52H90O7	[M+H-H2O]+
811.6671728	811.6658	0.0014	1.7248	TG 45:1;O3	C48H90O9	[M+H]+
811.6671728	811.6687	0.0016	1.9712	EPC 45:3;O2	C47H91N2O6P	[M+H]+
811.6671728	811.6687	0.0016	1.9712	SM 42:3;O2	C47H91N2O6P	[M+H]+
811.6671728	811.6658	0.0014	1.7248	MGDG 39:0	C48H92O10	[M+H-H2O]+
811.6671728	811.6687	0.0016	1.9712	EPC 45:2;O3	C47H93N2O7P	[M+H-H2O]+
811.6671728	811.6687	0.0016	1.9712	SM 42:2;O3	C47H93N2O7P	[M+H-H2O]+
811.6671728	811.6663	0.0008	0.9856	EPC 43:0;O2	C45H93N2O6PNa	[M+Na]+
811.6671728	811.6663	0.0008	0.9856	SM 40:0;O2	C45H93N2O6PNa	[M+Na]+
811.6671728	811.6687	0.0016	1.9712	CerP 47:4;O2	C47H88NO6P	[M+NH4]+
811.7147932	811.715	0.0002	0.2464	DG 48:2	C51H96O5Na	[M+Na]+
811.7147932	811.715	0.0002	0.2464	DG O-48:3;O	C51H96O5Na	[M+Na]+
811.7147932	811.715	0.0002	0.2464	TG O-48:2	C51H96O5Na	[M+Na]+
812.2985776	812.3018	0.0032	3.9394	PIP 25:7;O	C34H52O17P2	[M+NH4]+
812.3016000	812.3018	0.0002	0.2462	PIP 25:7;O	C34H52O17P2	[M+NH4]+
812.6709137	812.6739	0.0029	3.5685	ACer 49:3;O4	C49H91NO6Na	[M+Na]+
	812.6739	0.0029	3.5685	Cer 49:4;O5	C49H91NO6Na	[M+Na]+

812.6709137						
814.3148981	814.3175	0.0026	3.1929	PIP 25:6;O	C34H54O17P2	[M+NH4]+
	814.3538	0.0001	0.1228	PIP 26:5	C35H58O16P2	[M+NH4]+
814.3537004						
814.3537004	814.354	0.0002	0.2456	IPC 31:6;O5	C37H62NO14PK	[M+K]+
	815.5756	0.0001	0.1226	IPC 34:0;O3	C40H80NO12P	[M+NH4]+
815.5755617						
816.3687801	816.3695	0.0007	0.8575	PIP 26:4	C35H60O16P2	[M+NH4]+
	816.3696	0.0008	0.9799	IPC 31:5;O5	C37H64NO14PK	[M+K]+
816.3687801						
816.3707496	816.3695	0.0013	1.5924	PIP 26:4	C35H60O16P2	[M+NH4]+
	816.3696	0.0011	1.3474	IPC 31:5;O5	C37H64NO14PK	[M+K]+
816.3707496						
816.5878394	816.5878	0.0001	0.1225	CerP 46:5;O3	C46H84NO7PNa	[M+Na]+
	816.5878	0.0001	0.1225	PC O-38:5	C46H84NO7PNa	[M+Na]+
816.5878394						
816.5878394	816.5878	0.0001	0.1225	PE O-41:5	C46H84NO7PNa	[M+Na]+
	816.5879	0.0001	0.1225	CerP 43:0;O4	C43H88NO8PK	[M+K]+
816.5878394						
816.5878394	816.5879	0.0001	0.1225	PC O-35:0;O	C43H88NO8PK	[M+K]+
	816.5879	0.0001	0.1225	PE O-38:0;O	C43H88NO8PK	[M+K]+
816.5878394						
817.3728343	817.3689	0.0040	4.8938	PI O-32:9	C41H63O12PK	[M+K]+
	817.5854	0.0030	3.6693	PC O-39:9	C47H78NO7P	[M+NH4]+
817.5884509						
817.5884509	817.5854	0.0030	3.6693	PE O-42:9	C47H78NO7P	[M+NH4]+
	817.5929	0.0015	1.8347	PG O-37:0;O	C43H87O10PNa	[M+Na]+
817.5913964						
818.2884879	818.2889	0.0004	0.4888	PIP2 20:1	C29H55O19P3	[M+NH4]+
	818.2889	0.0023	2.8107	PIP2 20:1	C29H55O19P3	[M+NH4]+
818.2911512						
818.3852930	818.3851	0.0002	0.2444	PIP 26:3	C35H62O16P2	[M+NH4]+
	818.3853	0.0000	0.0000	IPC 31:4;O5	C37H66NO14PK	[M+K]+
818.3852930						
818.6035184	818.6034	0.0001	0.1222	CerP 46:4;O3	C46H86NO7PNa	[M+Na]+
	818.6034	0.0001	0.1222	PC O-38:4	C46H86NO7PNa	[M+Na]+
818.6035184						
818.6035184	818.6034	0.0001	0.1222	PE O-41:4	C46H86NO7PNa	[M+Na]+

	818.6269	0.0004	0.4886	CerP 45:2;O5	C45H88NO9P	[M+H]+
818.6265427						
818.6265427	818.6269	0.0004	0.4886	PC 37:1;O	C45H88NO9P	[M+H]+
	818.6269	0.0004	0.4886	PE 40:1;O	C45H88NO9P	[M+H]+
818.6265427						
818.6265427	818.6269	0.0004	0.4886	PS O-39:1	C45H88NO9P	[M+H]+
	818.6269	0.0004	0.4886	CerP 45:1;O6	C45H90NO10P	[M+H-H2O]+
818.6265427						
818.6265427	818.6269	0.0004	0.4886	PS O-39:0;O	C45H90NO10P	[M+H-H2O]+
	818.6269	0.0004	0.4886	PA 42:2;O	C45H85O9P	[M+NH4]+
818.6265427						
818.6265427	818.6269	0.0004	0.4886	PG O-39:3	C45H85O9P	[M+NH4]+
	818.6271	0.0005	0.6108	ACer 47:2;O5	C47H89NO7K	[M+K]+
818.6265427						
818.6265427	818.6271	0.0005	0.6108	Cer 47:3;O6	C47H89NO7K	[M+K]+
	819.6052	0.0016	1.9522	CE 27:7;O	C54H84O3K	[M+K]+
819.6068150						
820.6192369	820.6191	0.0002	0.2437	CerP 46:3;O3	C46H88NO7PNa	[M+Na]+
	820.6191	0.0002	0.2437	PC O-38:3	C46H88NO7PNa	[M+Na]+
820.6192369						
820.6192369	820.6191	0.0002	0.2437	PE O-41:3	C46H88NO7PNa	[M+Na]+
	820.6215	0.0005	0.6093	CerP 48:6;O3	C48H86NO7P	[M+H]+
820.6209284						
820.6209284	820.6215	0.0005	0.6093	PC O-40:6	C48H86NO7P	[M+H]+
	820.6215	0.0005	0.6093	PE O-43:6	C48H86NO7P	[M+H]+
820.6209284						
820.6209284	820.6215	0.0005	0.6093	CerP 48:5;O4	C48H88NO8P	[M+H-H2O]+
	820.6215	0.0005	0.6093	PC 40:4	C48H88NO8P	[M+H-H2O]+
820.6209284						
820.6209284	820.6215	0.0005	0.6093	PC O-40:5;O	C48H88NO8P	[M+H-H2O]+
	820.6215	0.0005	0.6093	PE 43:4	C48H88NO8P	[M+H-H2O]+
820.6209284						
820.6209284	820.6215	0.0005	0.6093	PE O-43:5;O	C48H88NO8P	[M+H-H2O]+
	820.6215	0.0005	0.6093	PA O-45:7	C48H83O7P	[M+NH4]+
820.6209284						
820.6209284	820.6216	0.0006	0.7312	ACer 50:6;O3	C50H87NO5K	[M+K]+
	821.625	0.0015	1.8257	HexCer 41:6;O3	C47H81NO9	[M+NH4]+

821.6234442						
821.6658729	821.6654	0.0005	0.6085	CE 26:4;O4	C53H88O6	[M+H]+
	821.6654	0.0005	0.6085	DG 50:8;O	C53H88O6	[M+H]+
821.6658729						
821.6658729	821.6654	0.0005	0.6085	DG O-50:9;O2	C53H88O6	[M+H]+
	821.6654	0.0005	0.6085	TG 50:7	C53H88O6	[M+H]+
821.6658729						
821.6658729	821.6654	0.0005	0.6085	TG O-50:8;O	C53H88O6	[M+H]+
	821.6654	0.0005	0.6085	DG 50:7;O2	C53H90O7	[M+H-H2O]+
821.6658729						
821.6658729	821.6654	0.0005	0.6085	TG 50:6;O	C53H90O7	[M+H-H2O]+
	821.6654	0.0005	0.6085	TG O-50:7;O2	C53H90O7	[M+H-H2O]+
821.6658729						
822.5843563	822.5855	0.0011	1.3372	IPC 37:1;O2	C43H84NO11P	[M+H]+
	822.5855	0.0011	1.3372	PS 37:0;O	C43H84NO11P	[M+H]+
822.5843563						
822.5843563	822.5855	0.0011	1.3372	IPC 37:0;O3	C43H86NO12P	[M+H-H2O]+
	822.5855	0.0011	1.3372	LPI O-34:3	C43H81O11P	[M+NH4]+
822.5843563						
822.5843563	822.5855	0.0011	1.3372	PG 37:2;O	C43H81O11P	[M+NH4]+
	822.5856	0.0012	1.4588	HexCer 39:2;O3	C45H85NO9K	[M+K]+
822.5843563						
822.6345256	822.6347	0.0002	0.2431	CerP 46:2;O3	C46H90NO7PNa	[M+Na]+
	822.6347	0.0002	0.2431	PC O-38:2	C46H90NO7PNa	[M+Na]+
822.6345256						
822.6345256	822.6347	0.0002	0.2431	PE O-41:2	C46H90NO7PNa	[M+Na]+
	823.6406	0.0023	2.7925	HexCer 41:5;O3	C47H83NO9	[M+NH4]+
823.6382678						
823.6382678	823.6365	0.0018	2.1854	CE 27:5;O	C54H88O3K	[M+K]+
	823.6786	0.0005	0.6070	CE 24:0;O4	C51H92O6Na	[M+Na]+
823.6781353						
823.6781353	823.6786	0.0005	0.6070	DG 48:4;O	C51H92O6Na	[M+Na]+
	823.6786	0.0005	0.6070	DG O-48:5;O2	C51H92O6Na	[M+Na]+
823.6781353						
823.6781353	823.6786	0.0005	0.6070	TG 48:3	C51H92O6Na	[M+Na]+
	823.6786	0.0005	0.6070	TG O-48:4;O	C51H92O6Na	[M+Na]+
823.6781353						
823.6781353	823.677	0.0011	1.3355	ACer 48:4;O6	C48H87NO8	[M+NH4]+

	823.677	0.0011	1.3355	HexCer 42:4;O2	C48H87NO8	[M+NH4]+
823.6781353						
823.6809463	823.681	0.0001	0.1214	CE 26:3;O4	C53H90O6	[M+H]+
	823.681	0.0001	0.1214	DG 50:7;O	C53H90O6	[M+H]+
823.6809463						
823.6809463	823.681	0.0001	0.1214	DG O-50:8;O2	C53H90O6	[M+H]+
	823.681	0.0001	0.1214	TG 50:6	C53H90O6	[M+H]+
823.6809463						
823.6809463	823.681	0.0001	0.1214	TG O-50:7;O	C53H90O6	[M+H]+
	823.681	0.0001	0.1214	DG 50:6;O2	C53H92O7	[M+H-H2O]+
823.6809463						
823.6809463	823.681	0.0001	0.1214	TG 50:5;O	C53H92O7	[M+H-H2O]+
	823.681	0.0001	0.1214	TG O-50:6;O2	C53H92O7	[M+H-H2O]+
823.6809463						
824.6494876	824.6504	0.0009	1.0914	CerP 46:1;O3	C46H92NO7PNa	[M+Na]+
	824.6504	0.0009	1.0914	PC O-38:1	C46H92NO7PNa	[M+Na]+
824.6494876						
824.6494876	824.6504	0.0009	1.0914	PE O-41:1	C46H92NO7PNa	[M+Na]+
	824.6487	0.0007	0.8488	EPC 41:1;O5	C43H87N2O9P	[M+NH4]+
824.6494876						
824.6494876	824.6487	0.0007	0.8488	SM 38:1;O5	C43H87N2O9P	[M+NH4]+
	824.6504	0.0006	0.7276	CerP 46:1;O3	C46H92NO7PNa	[M+Na]+
824.6509344						
824.6509344	824.6504	0.0006	0.7276	PC O-38:1	C46H92NO7PNa	[M+Na]+
	824.6504	0.0006	0.7276	PE O-41:1	C46H92NO7PNa	[M+Na]+
824.6509344						
824.6704148	824.6739	0.0034	4.1228	ACer 50:4;O4	C50H91NO6Na	[M+Na]+
	824.6739	0.0034	4.1228	Cer 50:5;O5	C50H91NO6Na	[M+Na]+
824.6704148						
824.6704148	824.6739	0.0035	4.2441	PG O-39:0	C45H91O9P	[M+NH4]+
	824.674	0.0036	4.3654	Cer 47:0;O6	C47H95NO7K	[M+K]+
824.6704148						
824.6816930	824.6851	0.0034	4.1228	EPC 42:0;O4	C44H91N2O8P	[M+NH4]+
	824.6851	0.0034	4.1228	SM 39:0;O4	C44H91N2O8P	[M+NH4]+
824.6816930						
825.5748648	825.5752	0.0004	0.4845	EPC 43:6;O5	C45H81N2O9P	[M+H]+
	825.5752	0.0004	0.4845	SM 40:6;O5	C45H81N2O9P	[M+H]+

825.5748648						
825.5748648	825.5752	0.0004	0.4845	EPC 43:5;O6	C45H83N2O10P	[M+H-H2O]+
	825.5752	0.0004	0.4845	SM 40:5;O6	C45H83N2O10P	[M+H-H2O]+
825.5748648						
825.5748648	825.5752	0.0004	0.4845	PC 37:6;O	C45H78NO9P	[M+NH4]+
	825.5752	0.0004	0.4845	PE 40:6;O	C45H78NO9P	[M+NH4]+
825.5748648						
825.5748648	825.5752	0.0004	0.4845	PS O-39:6	C45H78NO9P	[M+NH4]+
	825.6521	0.0016	1.9379	CE 27:4;O	C54H90O3K	[M+K]+
825.6537607						
825.6940646	825.6943	0.0002	0.2422	DG 48:3;O	C51H94O6Na	[M+Na]+
	825.6943	0.0002	0.2422	DG O-48:4;O2	C51H94O6Na	[M+Na]+
825.6940646						
825.6940646	825.6943	0.0002	0.2422	TG 48:2	C51H94O6Na	[M+Na]+
	825.6943	0.0002	0.2422	TG O-48:3;O	C51H94O6Na	[M+Na]+
825.6940646						
826.5568119	826.5569	0.0000	0.0000	CerP 43:3;O6	C43H82NO10PNa	[M+Na]+
	826.5569	0.0000	0.0000	PS 37:1	C43H82NO10PNa	[M+Na]+
826.5568119						
826.5568119	826.5569	0.0000	0.0000	PS O-37:2;O	C43H82NO10PNa	[M+Na]+
	826.5616	0.0001	0.1210	TG 49:14;O	C52H72O7	[M+NH4]+
826.5615029						
827.6992700	827.7	0.0008	0.9665	EPC 46:2;O2	C48H95N2O6P	[M+H]+
	827.7	0.0008	0.9665	SM 43:2;O2	C48H95N2O6P	[M+H]+
827.6992700						
827.6992700	827.7	0.0008	0.9665	EPC 46:1;O3	C48H97N2O7P	[M+H-H2O]+
	827.7	0.0008	0.9665	SM 43:1;O3	C48H97N2O7P	[M+H-H2O]+
827.6992700						
827.6992700	827.7	0.0008	0.9665	CerP 48:3;O2	C48H92NO6P	[M+NH4]+
	828.3331	0.0035	4.2254	PIP 26:6;O	C35H56O17P2	[M+NH4]+
828.3295980						
828.3307486	828.3331	0.0024	2.8974	PIP 26:6;O	C35H56O17P2	[M+NH4]+
	828.6089	0.0001	0.1207	CerP 44:1;O5	C44H88NO9PNa	[M+Na]+
828.6088209						
828.6088209	828.6089	0.0001	0.1207	PC 36:0;O	C44H88NO9PNa	[M+Na]+
	828.6089	0.0001	0.1207	PE 39:0;O	C44H88NO9PNa	[M+Na]+
828.6088209						
828.6088209	828.6089	0.0001	0.1207	PS O-38:0	C44H88NO9PNa	[M+Na]+

828.6104189	828.6113	0.0009	1.0862	CerP 46:4;O5	C46H86NO9P	[M+H]+
828.6104189	828.6113	0.0009	1.0862	PC 38:3;O	C46H86NO9P	[M+H]+
828.6104189	828.6113	0.0009	1.0862	PE 41:3;O	C46H86NO9P	[M+H]+
828.6104189	828.6113	0.0009	1.0862	PS O-40:3	C46H86NO9P	[M+H]+
828.6104189	828.6113	0.0009	1.0862	CerP 46:3;O6	C46H88NO10P	[M+H-H2O]+
828.6104189	828.6113	0.0009	1.0862	PS 40:1	C46H88NO10P	[M+H-H2O]+
828.6104189	828.6113	0.0009	1.0862	PS O-40:2;O	C46H88NO10P	[M+H-H2O]+
828.6104189	828.6089	0.0015	1.8103	CerP 44:1;O5	C44H88NO9PNa	[M+Na]+
828.6104189	828.6089	0.0015	1.8103	PC 36:0;O	C44H88NO9PNa	[M+Na]+
828.6104189	828.6089	0.0015	1.8103	PE 39:0;O	C44H88NO9PNa	[M+Na]+
828.6104189	828.6089	0.0015	1.8103	PS O-38:0	C44H88NO9PNa	[M+Na]+
828.6104189	828.6113	0.0009	1.0862	PA 43:4;O	C46H83O9P	[M+NH4]+
828.6104189	828.6113	0.0009	1.0862	PG O-40:5	C46H83O9P	[M+NH4]+
828.6104189	828.6114	0.0010	1.2068	ACer 48:4;O5	C48H87NO7K	[M+K]+
828.6104189	828.6114	0.0010	1.2068	Cer 48:5;O6	C48H87NO7K	[M+K]+
829.3340823	829.3324	0.0017	2.0498	PIP 30:8	C39H60O16P2	[M+H-H2O]+
829.6126437	829.6129	0.0003	0.3616	TG O-54:15	C57H82O5	[M+H-H2O]+
830.6245858	830.6269	0.0024	2.8894	CerP 46:3;O5	C46H88NO9P	[M+H]+
830.6245858	830.6245	0.0000	0.0000	CerP 44:0;O5	C44H90NO9PNa	[M+Na]+
832.3643785	832.3644	0.0000	0.0000	PIP 26:4;O	C35H60O17P2	[M+NH4]+
832.3643785	832.3645	0.0001	0.1201	IPC 31:5;O6	C37H64NO15PK	[M+K]+
833.6495292	833.6501	0.0006	0.7197	TG 47:4;O3	C50H88O9	[M+H]+
833.6495292	833.6501	0.0006	0.7197	MGDG 41:3	C50H90O10	[M+H-H2O]+
833.6495292	833.6507	0.0012	1.4395	EPC 45:3;O2	C47H91N2O6PNa	[M+Na]+
833.6495292	833.6507	0.0012	1.4395	SM 42:3;O2	C47H91N2O6PNa	[M+Na]+

833.6495292						
833.6507455	833.6501	0.0006	0.7197	TG 47:4;O3	C50H88O9	[M+H]+
	833.6501	0.0006	0.7197	MGDG 41:3	C50H90O10	[M+H-H2O]+
833.6507455						
833.6507455	833.6507	0.0001	0.1200	EPC 45:3;O2	C47H91N2O6PNa	[M+Na]+
	833.6507	0.0001	0.1200	SM 42:3;O2	C47H91N2O6PNa	[M+Na]+
833.6507455						
833.6663397	833.6654	0.0010	1.1995	CE 27:5;O4	C54H88O6	[M+H]+
	833.6654	0.0010	1.1995	DG 51:9;O	C54H88O6	[M+H]+
833.6663397						
833.6663397	833.6654	0.0010	1.1995	DG O-51:10;O2	C54H88O6	[M+H]+
	833.6654	0.0010	1.1995	TG 51:8	C54H88O6	[M+H]+
833.6663397						
833.6663397	833.6654	0.0010	1.1995	TG O-51:9;O	C54H88O6	[M+H]+
	833.6654	0.0010	1.1995	DG 51:8;O2	C54H90O7	[M+H-H2O]+
833.6663397						
833.6663397	833.6654	0.0010	1.1995	TG 51:7;O	C54H90O7	[M+H-H2O]+
	833.6654	0.0010	1.1995	TG O-51:8;O2	C54H90O7	[M+H-H2O]+
833.6663397						
834.2818967	834.2838	0.0019	2.2774	PIP2 20:1;O	C29H55O20P3	[M+NH4]+
	834.3801	0.0009	1.0786	PIP 26:3;O	C35H62O17P2	[M+NH4]+
834.3791101						
834.3791101	834.3802	0.0011	1.3183	IPC 31:4;O6	C37H66NO15PK	[M+K]+
	834.3801	0.0016	1.9176	PIP 26:3;O	C35H62O17P2	[M+NH4]+
834.3816229						
834.3816229	834.3802	0.0015	1.7977	IPC 31:4;O6	C37H66NO15PK	[M+K]+
	834.6582	0.0039	4.6726	CerP 46:1;O5	C46H92NO9P	[M+H]+
834.6543495						
834.6543495	834.6582	0.0039	4.6726	PC 38:0;O	C46H92NO9P	[M+H]+
	834.6582	0.0039	4.6726	PE 41:0;O	C46H92NO9P	[M+H]+
834.6543495						
834.6543495	834.6582	0.0039	4.6726	PS O-40:0	C46H92NO9P	[M+H]+
	834.6582	0.0039	4.6726	CerP 46:0;O6	C46H94NO10P	[M+H-H2O]+
834.6543495						
834.6543495	834.6582	0.0039	4.6726	ACer 51:6;O4	C51H89NO6Na	[M+Na]+
	834.6582	0.0039	4.6726	PA 43:1;O	C46H89O9P	[M+NH4]+
834.6543495						
834.6543495	834.6582	0.0039	4.6726	PG O-40:2	C46H89O9P	[M+NH4]+

834.6543495	834.6584	0.0040	4.7924	ACer 48:1;O5	C48H93NO7K	[M+K]+
834.6543495	834.6584	0.0040	4.7924	Cer 48:2;O6	C48H93NO7K	[M+K]+
835.3839439	835.3877	0.0037	4.4291	DGDG 26:6	C41H64O15K	[M+K]+
836.2967349	836.2994	0.0027	3.2285	PIP2 20:0;O	C29H57O20P3	[M+NH4]+
837.2992722	837.2987	0.0006	0.7166	PIP2 24:2	C33H61O19P3	[M+H-H2O]+
837.2992722	837.2988	0.0005	0.5972	PIP 26:4	C35H60O16P2K	[M+K]+
837.3009422	837.2987	0.0022	2.6275	PIP2 24:2	C33H61O19P3	[M+H-H2O]+
837.3009422	837.2988	0.0021	2.5081	PIP 26:4	C35H60O16P2K	[M+K]+
837.6966538	837.6967	0.0000	0.0000	CE 27:3;O4	C54H92O6	[M+H]+
837.6966538	837.6967	0.0000	0.0000	DG 51:7;O	C54H92O6	[M+H]+
837.6966538	837.6967	0.0000	0.0000	DG O-51:8;O2	C54H92O6	[M+H]+
837.6966538	837.6967	0.0000	0.0000	TG 51:6	C54H92O6	[M+H]+
837.6966538	837.6967	0.0000	0.0000	TG O-51:7;O	C54H92O6	[M+H]+
837.6966538	837.6967	0.0000	0.0000	DG 51:6;O2	C54H94O7	[M+H-H2O]+
837.6966538	837.6967	0.0000	0.0000	TG 51:5;O	C54H94O7	[M+H-H2O]+
837.6966538	837.6967	0.0000	0.0000	TG O-51:6;O2	C54H94O7	[M+H-H2O]+
839.7089849	839.7099	0.0009	1.0718	DG 49:3;O	C52H96O6Na	[M+Na]+
839.7089849	839.7099	0.0009	1.0718	DG O-49:4;O2	C52H96O6Na	[M+Na]+
839.7089849	839.7099	0.0009	1.0718	TG 49:2	C52H96O6Na	[M+Na]+
839.7089849	839.7099	0.0009	1.0718	TG O-49:3;O	C52H96O6Na	[M+Na]+
839.7089849	839.7083	0.0007	0.8336	ACer 49:3;O6	C49H91NO8	[M+NH4]+
839.7089849	839.7083	0.0007	0.8336	HexCer 43:3;O2	C49H91NO8	[M+NH4]+
839.7104394	839.7099	0.0005	0.5954	DG 49:3;O	C52H96O6Na	[M+Na]+
839.7104394	839.7099	0.0005	0.5954	DG O-49:4;O2	C52H96O6Na	[M+Na]+
	839.7099	0.0005	0.5954	TG 49:2	C52H96O6Na	[M+Na]+

839.7104394						
839.7104394	839.7099	0.0005	0.5954	TG O-49:3;O	C52H96O6Na	[M+Na]+
	839.7487	0.0027	3.2152	CE 28:1;O3	C55H98O5	[M+H]+
839.7459913						
839.7459913	839.7463	0.0003	0.3573	DG 50:2	C53H100O5Na	[M+Na]+
	839.7463	0.0003	0.3573	DG O-50:3;O	C53H100O5Na	[M+Na]+
839.7459913						
839.7459913	839.7463	0.0003	0.3573	TG O-50:2	C53H100O5Na	[M+Na]+
	840.6089	0.0006	0.7138	CerP 45:2;O5	C45H88NO9PNa	[M+Na]+
840.6083344						
840.6083344	840.6089	0.0006	0.7138	PC 37:1;O	C45H88NO9PNa	[M+Na]+
	840.6089	0.0006	0.7138	PE 40:1;O	C45H88NO9PNa	[M+Na]+
840.6083344						
840.6083344	840.6089	0.0006	0.7138	PS O-39:1	C45H88NO9PNa	[M+Na]+
	840.6113	0.0008	0.9517	CerP 47:5;O5	C47H86NO9P	[M+H]+
840.6104769						
840.6104769	840.6113	0.0008	0.9517	PC 39:4;O	C47H86NO9P	[M+H]+
	840.6113	0.0008	0.9517	PE 42:4;O	C47H86NO9P	[M+H]+
840.6104769						
840.6104769	840.6113	0.0008	0.9517	PS O-41:4	C47H86NO9P	[M+H]+
	840.6113	0.0008	0.9517	CerP 47:4;O6	C47H88NO10P	[M+H-H2O]+
840.6104769						
840.6104769	840.6113	0.0008	0.9517	PS 41:2	C47H88NO10P	[M+H-H2O]+
	840.6113	0.0008	0.9517	PS O-41:3;O	C47H88NO10P	[M+H-H2O]+
840.6104769						
840.6104769	840.6089	0.0016	1.9034	CerP 45:2;O5	C45H88NO9PNa	[M+Na]+
	840.6089	0.0016	1.9034	PC 37:1;O	C45H88NO9PNa	[M+Na]+
840.6104769						
840.6104769	840.6089	0.0016	1.9034	PE 40:1;O	C45H88NO9PNa	[M+Na]+
	840.6089	0.0016	1.9034	PS O-39:1	C45H88NO9PNa	[M+Na]+
840.6104769						
840.6104769	840.6113	0.0008	0.9517	PA 44:5;O	C47H83O9P	[M+NH4]+
	840.6113	0.0008	0.9517	PG O-41:6	C47H83O9P	[M+NH4]+
840.6104769						
840.6104769	840.6114	0.0009	1.0706	ACer 49:5;O5	C49H87NO7K	[M+K]+
	840.6114	0.0009	1.0706	Cer 49:6;O6	C49H87NO7K	[M+K]+
840.6104769						
841.6067929	841.6065	0.0003	0.3565	EPC 44:5;O5	C46H85N2O9P	[M+H]+

	841.6065	0.0003	0.3565	SM 41:5;O5	C46H85N2O9P	[M+H]+
841.6067929						
841.6067929	841.6065	0.0003	0.3565	EPC 44:4;O6	C46H87N2O10P	[M+H-H2O]+
	841.6065	0.0003	0.3565	SM 41:4;O6	C46H87N2O10P	[M+H-H2O]+
841.6067929						
841.6067929	841.6065	0.0003	0.3565	CerP 46:6;O5	C46H82NO9P	[M+NH4]+
	841.6065	0.0003	0.3565	PC 38:5;O	C46H82NO9P	[M+NH4]+
841.6067929						
841.6067929	841.6065	0.0003	0.3565	PE 41:5;O	C46H82NO9P	[M+NH4]+
	841.6065	0.0003	0.3565	PS O-40:5	C46H82NO9P	[M+NH4]+
841.6067929						
841.6678490	841.6681	0.0002	0.2376	PA 46:2	C49H93O8P	[M+H]+
	841.6681	0.0002	0.2376	PA O-46:3;O	C49H93O8P	[M+H]+
841.6678490						
841.6678490	841.6681	0.0002	0.2376	PA 46:1;O	C49H95O9P	[M+H-H2O]+
	841.6681	0.0002	0.2376	PG O-43:2	C49H95O9P	[M+H-H2O]+
841.6678490						
841.6678490	841.668	0.0002	0.2376	CE 27:4;O3	C54H90O5Na	[M+Na]+
	841.668	0.0002	0.2376	DG 51:8	C54H90O5Na	[M+Na]+
841.6678490						
841.6678490	841.668	0.0002	0.2376	DG O-51:9;O	C54H90O5Na	[M+Na]+
	841.668	0.0002	0.2376	TG O-51:8	C54H90O5Na	[M+Na]+
841.6678490						
841.6678490	841.6682	0.0003	0.3564	DG 48:3;O	C51H94O6K	[M+K]+
	841.6682	0.0003	0.3564	DG O-48:4;O2	C51H94O6K	[M+K]+
841.6678490						
841.6678490	841.6682	0.0003	0.3564	TG 48:2	C51H94O6K	[M+K]+
	841.6682	0.0003	0.3564	TG O-48:3;O	C51H94O6K	[M+K]+
841.6678490						
841.7039784	841.7045	0.0005	0.5940	PA O-47:2	C50H97O7P	[M+H]+
	841.7045	0.0005	0.5940	PA 47:0	C50H99O8P	[M+H-H2O]+
841.7039784						
841.7039784	841.7045	0.0005	0.5940	PA O-47:1;O	C50H99O8P	[M+H-H2O]+
	841.7044	0.0004	0.4752	CE 28:3;O2	C55H94O4Na	[M+Na]+
841.7039784						
841.7039784	841.7044	0.0004	0.4752	DG O-52:8	C55H94O4Na	[M+Na]+
	841.7046	0.0006	0.7128	DG 49:2	C52H98O5K	[M+K]+

841.7039784						
841.7039784	841.7046	0.0006	0.7128	DG O-49:3;O	C52H98O5K	[M+K]+
	841.7046	0.0006	0.7128	TG O-49:2	C52H98O5K	[M+K]+
841.7039784						
842.3456503	842.3488	0.0031	3.6802	PIP 27:6;O	C36H58O17P2	[M+NH4]+
	842.4896	0.0009	1.0683	Hex2Cer 31:6;O4	C43H71NO15	[M+H]+
842.4887502						
842.4887502	842.4896	0.0009	1.0683	Hex2Cer 31:5;O5	C43H73NO16	[M+H-H2O]+
	842.4872	0.0015	1.7804	Hex2Cer 29:3;O4	C41H73NO15Na	[M+Na]+
842.4887502						
842.4887502	842.4896	0.0009	1.0683	DGDG 28:6	C43H68O15	[M+NH4]+
	842.493	0.0013	1.5430	SHexCer 34:2;O6	C40H75NO15S	[M+H]+
842.4916787						
842.4916787	842.4896	0.0020	2.3739	Hex2Cer 31:6;O4	C43H71NO15	[M+H]+
	842.4896	0.0020	2.3739	Hex2Cer 31:5;O5	C43H73NO16	[M+H-H2O]+
842.4916787						
842.4916787	842.4896	0.0020	2.3739	DGDG 28:6	C43H68O15	[M+NH4]+
	842.6716	0.0000	0.0000	HexCer 42:2;O4	C48H91NO10	[M+H]+
842.6715371						
842.6715371	842.6716	0.0000	0.0000	HexCer 42:1;O5	C48H93NO11	[M+H-H2O]+
	842.6716	0.0000	0.0000	MGDG 39:2	C48H88O10	[M+NH4]+
842.6715371						
843.6067477	843.6069	0.0002	0.2371	IPC 36:0;O3	C42H84NO12P	[M+NH4]+
	843.6133	0.0002	0.2371	DG 51:12;O2	C54H82O7	[M+H]+
843.6135718						
843.6135718	843.6133	0.0002	0.2371	TG 51:11;O	C54H82O7	[M+H]+
	843.6133	0.0002	0.2371	TG O-51:12;O2	C54H82O7	[M+H]+
843.6135718						
843.6135718	843.6133	0.0002	0.2371	TG 51:10;O2	C54H84O8	[M+H-H2O]+
	843.6133	0.0002	0.2371	TG O-51:11;O3	C54H84O8	[M+H-H2O]+
843.6135718						
844.6180637	844.6191	0.0010	1.1840	CerP 48:5;O3	C48H88NO7PNa	[M+Na]+
	844.6191	0.0010	1.1840	PC O-40:5	C48H88NO7PNa	[M+Na]+
844.6180637						
844.6180637	844.6191	0.0010	1.1840	PE O-43:5	C48H88NO7PNa	[M+Na]+
	844.6174	0.0006	0.7104	EPC 43:5;O5	C45H83N2O9P	[M+NH4]+
844.6180637						
844.6180637	844.6174	0.0006	0.7104	SM 40:5;O5	C45H83N2O9P	[M+NH4]+

	844.6192	0.0011	1.3024	CerP 45:0;O4	C45H92NO8PK	[M+K]+
844.6180637						
844.6180637	844.6192	0.0011	1.3024	PC O-37:0;O	C45H92NO8PK	[M+K]+
	844.6192	0.0011	1.3024	PE O-40:0;O	C45H92NO8PK	[M+K]+
844.6180637						
844.6292249	844.6297	0.0005	0.5920	HexCer 44:6;O4	C50H87NO10	[M+H-H2O]+
	844.6297	0.0005	0.5920	TG 47:7;O3	C50H82O9	[M+NH4]+
844.6292249						
845.6436905	845.6442	0.0005	0.5913	DG 55:14	C58H86O5	[M+H-H2O]+
	845.6442	0.0005	0.5913	TG O-55:14	C58H86O5	[M+H-H2O]+
845.6436905						
848.7694772	848.7702	0.0007	0.8247	ACer 53:2;O4	C53H101NO6	[M+H]+
	848.7702	0.0007	0.8247	Cer 53:3;O5	C53H101NO6	[M+H]+
848.7694772						
848.7694772	848.7702	0.0007	0.8247	ACer 53:1;O5	C53H103NO7	[M+H-H2O]+
	848.7702	0.0007	0.8247	Cer 53:2;O6	C53H103NO7	[M+H-H2O]+
848.7694772						
848.7694772	848.7702	0.0007	0.8247	DG 50:3;O	C53H98O6	[M+NH4]+
	848.7702	0.0007	0.8247	DG O-50:4;O2	C53H98O6	[M+NH4]+
848.7694772						
848.7694772	848.7702	0.0007	0.8247	TG 50:2	C53H98O6	[M+NH4]+
	848.7702	0.0007	0.8247	TG O-50:3;O	C53H98O6	[M+NH4]+
848.7694772						
848.7704419	848.7702	0.0003	0.3535	ACer 53:2;O4	C53H101NO6	[M+H]+
	848.7702	0.0003	0.3535	Cer 53:3;O5	C53H101NO6	[M+H]+
848.7704419						
848.7704419	848.7702	0.0003	0.3535	ACer 53:1;O5	C53H103NO7	[M+H-H2O]+
	848.7702	0.0003	0.3535	Cer 53:2;O6	C53H103NO7	[M+H-H2O]+
848.7704419						
848.7704419	848.7702	0.0003	0.3535	DG 50:3;O	C53H98O6	[M+NH4]+
	848.7702	0.0003	0.3535	DG O-50:4;O2	C53H98O6	[M+NH4]+
848.7704419						
848.7704419	848.7702	0.0003	0.3535	TG 50:2	C53H98O6	[M+NH4]+
	848.7702	0.0003	0.3535	TG O-50:3;O	C53H98O6	[M+NH4]+
848.7704419						
851.6974170	851.6971	0.0004	0.4697	TG 48:2;O3	C51H94O9	[M+H]+
	851.6971	0.0004	0.4697	MGDG 42:1	C51H96O10	[M+H-H2O]+
851.6974170						
851.6974170	851.6976	0.0002	0.2348	EPC 46:1;O2	C48H97N2O6PNa	[M+Na]+

	851.6976	0.0002	0.2348	SM 43:1;O2	C48H97N2O6PNa	[M+Na]+
851.6974170						
851.7093541	851.7099	0.0006	0.7045	CE 26:0;O4	C53H96O6Na	[M+Na]+
	851.7099	0.0006	0.7045	DG 50:4;O	C53H96O6Na	[M+Na]+
851.7093541						
851.7093541	851.7099	0.0006	0.7045	DG O-50:5;O2	C53H96O6Na	[M+Na]+
	851.7099	0.0006	0.7045	TG 50:3	C53H96O6Na	[M+Na]+
851.7093541						
851.7093541	851.7099	0.0006	0.7045	TG O-50:4;O	C53H96O6Na	[M+Na]+
	851.7083	0.0011	1.2915	ACer 50:4;O6	C50H91NO8	[M+NH4]+
851.7093541						
851.7093541	851.7083	0.0011	1.2915	HexCer 44:4;O2	C50H91NO8	[M+NH4]+
	851.7099	0.0005	0.5871	CE 26:0;O4	C53H96O6Na	[M+Na]+
851.7103929						
851.7103929	851.7099	0.0005	0.5871	DG 50:4;O	C53H96O6Na	[M+Na]+
	851.7099	0.0005	0.5871	DG O-50:5;O2	C53H96O6Na	[M+Na]+
851.7103929						
851.7103929	851.7099	0.0005	0.5871	TG 50:3	C53H96O6Na	[M+Na]+
	851.7099	0.0005	0.5871	TG O-50:4;O	C53H96O6Na	[M+Na]+
851.7103929						
852.5746330	852.5749	0.0003	0.3519	PS 41:5	C47H82NO10P	[M+H]+
	852.5749	0.0003	0.3519	PS O-41:6;O	C47H82NO10P	[M+H]+
852.5746330						
852.5746330	852.5749	0.0003	0.3519	IPC 41:5;O2	C47H84NO11P	[M+H-H2O]+
	852.5749	0.0003	0.3519	PS 41:4;O	C47H84NO11P	[M+H-H2O]+
852.5746330						
852.5746330	852.5749	0.0003	0.3519	PG 41:7	C47H79O10P	[M+NH4]+
	852.5749	0.0003	0.3519	PG O-41:8;O	C47H79O10P	[M+NH4]+
852.5746330						
852.6794723	852.68	0.0006	0.7037	EPC 43:1;O5	C45H91N2O9P	[M+NH4]+
	852.68	0.0006	0.7037	SM 40:1;O5	C45H91N2O9P	[M+NH4]+
852.6794723						
852.7130196	852.7164	0.0034	3.9873	EPC 44:0;O4	C46H95N2O8P	[M+NH4]+
	852.7164	0.0034	3.9873	SM 41:0;O4	C46H95N2O8P	[M+NH4]+
852.7130196						
853.5810880	853.5801	0.0010	1.1715	PI 35:0	C44H85O13P	[M+H]+
	853.5801	0.0010	1.1715	PI O-35:1;O	C44H85O13P	[M+H]+

853.5810880						
853.5810880	853.5824	0.0013	1.5230	MGDG 42:9	C51H80O10	[M+H]+
	853.58	0.0011	1.2887	MGDG 40:6	C49H82O10Na	[M+Na]+
853.5810880						
853.5810880	853.5818	0.0007	0.8201	SHexCer 37:2;O3	C43H81NO12S	[M+NH4]+
	853.6065	0.0003	0.3514	EPC 45:6;O5	C47H85N2O9P	[M+H]+
853.6062113						
853.6062113	853.6065	0.0003	0.3514	SM 42:6;O5	C47H85N2O9P	[M+H]+
	853.6065	0.0003	0.3514	EPC 45:5;O6	C47H87N2O10P	[M+H-H2O]+
853.6062113						
853.6062113	853.6065	0.0003	0.3514	SM 42:5;O6	C47H87N2O10P	[M+H-H2O]+
	853.6065	0.0003	0.3514	PC 39:6;O	C47H82NO9P	[M+NH4]+
853.6062113						
853.6062113	853.6065	0.0003	0.3514	PE 42:6;O	C47H82NO9P	[M+NH4]+
	853.6065	0.0003	0.3514	PS O-41:6	C47H82NO9P	[M+NH4]+
853.6062113						
853.7253567	853.7256	0.0002	0.2343	DG 50:3;O	C53H98O6Na	[M+Na]+
	853.7256	0.0002	0.2343	DG O-50:4;O2	C53H98O6Na	[M+Na]+
853.7253567						
853.7253567	853.7256	0.0002	0.2343	TG 50:2	C53H98O6Na	[M+Na]+
	853.7256	0.0002	0.2343	TG O-50:3;O	C53H98O6Na	[M+Na]+
853.7253567						
854.3459053	854.3488	0.0028	3.2773	PIP 28:7;O	C37H58O17P2	[M+NH4]+
	855.4978	0.0010	1.1689	IPC 34:4;O6	C40H72NO15P	[M+NH4]+
855.4967465						
856.3617048	856.3644	0.0027	3.1529	PIP 28:6;O	C37H60O17P2	[M+NH4]+
	857.3637	0.0014	1.6329	PIP 32:8	C41H64O16P2	[M+H-H2O]+
857.3651194						
858.2814774	858.2838	0.0023	2.6798	PIP2 22:3;O	C31H55O20P3	[M+NH4]+
	858.3706	0.0031	3.6115	SHexCer 33:6;O6	C39H65NO15SK	[M+K]+
858.3675485						
858.5705983	858.5702	0.0004	0.4659	IPC 36:0;O5	C42H84NO14P	[M+H]+
	858.5702	0.0004	0.4659	HexCer 41:6;O5	C47H81NO11Na	[M+Na]+
858.5705983						
858.5705983	858.5702	0.0004	0.4659	PI 33:0;O	C42H81O14P	[M+NH4]+
	858.5703	0.0003	0.3494	HexCer 38:1;O6	C44H85NO12K	[M+K]+
858.5705983						
861.5448343	861.5447	0.0001	0.1161	IPC 34:1;O6	C40H78NO15P	[M+NH4]+

	861.5728	0.0003	0.3482	EPC 44:6;O5	C46H83N2O9PNa	[M+Na]+
861.5724999						
861.5724999	861.5728	0.0003	0.3482	SM 41:6;O5	C46H83N2O9PNa	[M+Na]+
	861.5716	0.0009	1.0446	SHexCer 35:0;O5	C41H81NO14S	[M+NH4]+
861.5724999						
861.5724999	861.573	0.0005	0.5803	EPC 41:1;O6	C43H87N2O10PK	[M+K]+
	861.573	0.0005	0.5803	SM 38:1;O6	C43H87N2O10PK	[M+K]+
861.5724999						
862.5759072	862.5745	0.0014	1.6230	PC 44:11	C52H82NO8P	[M+H-H2O]+
	862.5745	0.0014	1.6230	PC O-44:12;O	C52H82NO8P	[M+H-H2O]+
862.5759072						
862.5759072	862.5745	0.0014	1.6230	PE 47:11	C52H82NO8P	[M+H-H2O]+
	862.5745	0.0014	1.6230	PE O-47:12;O	C52H82NO8P	[M+H-H2O]+
862.5759072						
862.5759072	862.578	0.0021	2.4346	IPC 37:0;O3	C43H86NO12PNa	[M+Na]+
	862.5932	0.0004	0.4637	CerP 47:5;O5	C47H86NO9PNa	[M+Na]+
862.5927937						
862.5927937	862.5932	0.0004	0.4637	PC 39:4;O	C47H86NO9PNa	[M+Na]+
	862.5932	0.0004	0.4637	PE 42:4;O	C47H86NO9PNa	[M+Na]+
862.5927937						
862.5927937	862.5932	0.0004	0.4637	PS O-41:4	C47H86NO9PNa	[M+Na]+
	862.5934	0.0006	0.6956	CerP 44:0;O6	C44H90NO10PK	[M+K]+
862.5927937						
863.5862415	863.5885	0.0022	2.5475	EPC 44:5;O5	C46H85N2O9PNa	[M+Na]+
	863.5885	0.0022	2.5475	SM 41:5;O5	C46H85N2O9PNa	[M+Na]+
863.5862415						
863.5862415	863.5839	0.0024	2.7791	Hex2Cer 32:3;O3	C44H79NO14	[M+NH4]+
	863.5886	0.0024	2.7791	EPC 41:0;O6	C43H89N2O10PK	[M+K]+
863.5862415						
863.5862415	863.5886	0.0024	2.7791	SM 38:0;O6	C43H89N2O10PK	[M+K]+
	864.6089	0.0003	0.3470	CerP 47:4;O5	C47H88NO9PNa	[M+Na]+
864.6086034						
864.6086034	864.6089	0.0003	0.3470	PC 39:3;O	C47H88NO9PNa	[M+Na]+
	864.6089	0.0003	0.3470	PE 42:3;O	C47H88NO9PNa	[M+Na]+
864.6086034						
864.6086034	864.6089	0.0003	0.3470	PS O-41:3	C47H88NO9PNa	[M+Na]+
	864.6113	0.0009	1.0409	PC 41:6;O	C49H86NO9P	[M+H]+
864.6104323						
864.6104323	864.6113	0.0009	1.0409	PE 44:6;O	C49H86NO9P	[M+H]+

	864.6113	0.0009	1.0409	PS O-43:6	C49H86NO9P	[M+H]+
864.6104323						
864.6104323	864.6113	0.0009	1.0409	CerP 49:6;O6	C49H88NO10P	[M+H-H2O]+
	864.6113	0.0009	1.0409	PS 43:4	C49H88NO10P	[M+H-H2O]+
864.6104323						
864.6104323	864.6113	0.0009	1.0409	PS O-43:5;O	C49H88NO10P	[M+H-H2O]+
	864.6089	0.0015	1.7349	CerP 47:4;O5	C47H88NO9PNa	[M+Na]+
864.6104323						
864.6104323	864.6089	0.0015	1.7349	PC 39:3;O	C47H88NO9PNa	[M+Na]+
	864.6089	0.0015	1.7349	PE 42:3;O	C47H88NO9PNa	[M+Na]+
864.6104323						
864.6104323	864.6089	0.0015	1.7349	PS O-41:3	C47H88NO9PNa	[M+Na]+
	864.6113	0.0009	1.0409	PA 46:7;O	C49H83O9P	[M+NH4]+
864.6104323						
864.6104323	864.6113	0.0009	1.0409	PG O-43:8	C49H83O9P	[M+NH4]+
	865.6106	0.0020	2.3105	PA O-50:11	C53H85O7P	[M+H]+
865.6125540						
865.6125540	865.6106	0.0020	2.3105	PA 50:9	C53H87O8P	[M+H-H2O]+
	865.6106	0.0020	2.3105	PA O-50:10;O	C53H87O8P	[M+H-H2O]+
865.6125540						
865.6125540	865.6107	0.0019	2.1950	CE 28:7;O3	C55H86O5K	[M+K]+
	865.6107	0.0019	2.1950	DG 52:11	C55H86O5K	[M+K]+
865.6125540						
865.6125540	865.6107	0.0019	2.1950	DG O-52:12;O	C55H86O5K	[M+K]+
	865.6107	0.0019	2.1950	TG O-52:11	C55H86O5K	[M+K]+
865.6125540						
865.7252575	865.7256	0.0003	0.3465	CE 27:0;O4	C54H98O6Na	[M+Na]+
	865.7256	0.0003	0.3465	DG 51:4;O	C54H98O6Na	[M+Na]+
865.7252575						
865.7252575	865.7256	0.0003	0.3465	DG O-51:5;O2	C54H98O6Na	[M+Na]+
	865.7256	0.0003	0.3465	TG 51:3	C54H98O6Na	[M+Na]+
865.7252575						
865.7252575	865.7256	0.0003	0.3465	TG O-51:4;O	C54H98O6Na	[M+Na]+
	866.6245	0.0005	0.5770	CerP 47:3;O5	C47H90NO9PNa	[M+Na]+
866.6250396						
866.6250396	866.6245	0.0005	0.5770	PC 39:2;O	C47H90NO9PNa	[M+Na]+
	866.6245	0.0005	0.5770	PE 42:2;O	C47H90NO9PNa	[M+Na]+

866.6250396						
866.6250396	866.6245	0.0005	0.5770	PS O-41:2	C47H90NO9PNa	[M+Na]+
	867.6837	0.0002	0.2305	PA 48:3	C51H95O8P	[M+H]+
867.6835672						
867.6835672	867.6837	0.0002	0.2305	PA O-48:4;O	C51H95O8P	[M+H]+
	867.6837	0.0002	0.2305	PA 48:2;O	C51H97O9P	[M+H-H2O]+
867.6835672						
867.6835672	867.6837	0.0002	0.2305	PG O-45:3	C51H97O9P	[M+H-H2O]+
	867.6837	0.0001	0.1152	CE 29:5;O3	C56H92O5Na	[M+Na]+
867.6835672						
867.6835672	867.6837	0.0001	0.1152	DG 53:9	C56H92O5Na	[M+Na]+
	867.6837	0.0001	0.1152	DG O-53:10;O	C56H92O5Na	[M+Na]+
867.6835672						
867.6835672	867.6837	0.0001	0.1152	TG O-53:9	C56H92O5Na	[M+Na]+
	867.6838	0.0003	0.3457	CE 26:0;O4	C53H96O6K	[M+K]+
867.6835672						
867.6835672	867.6838	0.0003	0.3457	DG 50:4;O	C53H96O6K	[M+K]+
	867.6838	0.0003	0.3457	DG O-50:5;O2	C53H96O6K	[M+K]+
867.6835672						
867.6835672	867.6838	0.0003	0.3457	TG 50:3	C53H96O6K	[M+K]+
	867.6838	0.0003	0.3457	TG O-50:4;O	C53H96O6K	[M+K]+
867.6835672						
867.7395852	867.7396	0.0000	0.0000	ACer 51:3;O6	C51H95NO8	[M+NH4]+
	867.7396	0.0000	0.0000	HexCer 45:3;O2	C51H95NO8	[M+NH4]+
867.7395852						
867.7410870	867.7412	0.0001	0.1152	DG 51:3;O	C54H100O6Na	[M+Na]+
	867.7412	0.0001	0.1152	DG O-51:4;O2	C54H100O6Na	[M+Na]+
867.7410870						
867.7410870	867.7412	0.0001	0.1152	TG 51:2	C54H100O6Na	[M+Na]+
	867.7412	0.0001	0.1152	TG O-51:3;O	C54H100O6Na	[M+Na]+
867.7410870						
867.7773539	867.7776	0.0002	0.2305	DG 52:2	C55H104O5Na	[M+Na]+
	867.7776	0.0002	0.2305	DG O-52:3;O	C55H104O5Na	[M+Na]+
867.7773539						
867.7773539	867.7776	0.0002	0.2305	TG O-52:2	C55H104O5Na	[M+Na]+
	868.6402	0.0011	1.2663	CerP 47:2;O5	C47H92NO9PNa	[M+Na]+
868.6390860						
868.6390860	868.6402	0.0011	1.2663	PC 39:1;O	C47H92NO9PNa	[M+Na]+

	868.6402	0.0011	1.2663	PE 42:1;O	C47H92NO9PNa	[M+Na]+
868.6390860						
868.6390860	868.6402	0.0011	1.2663	PS O-41:1	C47H92NO9PNa	[M+Na]+
	868.6426	0.0016	1.8420	CerP 49:5;O5	C49H90NO9P	[M+H]+
868.6410106						
868.6410106	868.6426	0.0016	1.8420	PC 41:4;O	C49H90NO9P	[M+H]+
	868.6426	0.0016	1.8420	PE 44:4;O	C49H90NO9P	[M+H]+
868.6410106						
868.6410106	868.6426	0.0016	1.8420	PS O-43:4	C49H90NO9P	[M+H]+
	868.6426	0.0016	1.8420	CerP 49:4;O6	C49H92NO10P	[M+H-H2O]+
868.6410106						
868.6410106	868.6426	0.0016	1.8420	PS 43:2	C49H92NO10P	[M+H-H2O]+
	868.6426	0.0016	1.8420	PS O-43:3;O	C49H92NO10P	[M+H-H2O]+
868.6410106						
868.6410106	868.6402	0.0008	0.9210	CerP 47:2;O5	C47H92NO9PNa	[M+Na]+
	868.6402	0.0008	0.9210	PC 39:1;O	C47H92NO9PNa	[M+Na]+
868.6410106						
868.6410106	868.6402	0.0008	0.9210	PE 42:1;O	C47H92NO9PNa	[M+Na]+
	868.6402	0.0008	0.9210	PS O-41:1	C47H92NO9PNa	[M+Na]+
868.6410106						
868.6410106	868.6426	0.0016	1.8420	PA 46:5;O	C49H87O9P	[M+NH4]+
	868.6426	0.0016	1.8420	PG O-43:6	C49H87O9P	[M+NH4]+
868.6410106						
868.6410106	868.6427	0.0017	1.9571	ACer 51:5;O5	C51H91NO7K	[M+K]+
	868.6427	0.0017	1.9571	Cer 51:6;O6	C51H91NO7K	[M+K]+
868.6410106						
868.7795334	868.7752	0.0043	4.9495	ACer 56:5;O3	C56H101NO5	[M+H]+
	868.7752	0.0043	4.9495	ACer 56:4;O4	C56H103NO6	[M+H-H2O]+
868.7795334						
868.7795334	868.7752	0.0043	4.9495	CE 29:2;O3	C56H98O5	[M+NH4]+
	868.7752	0.0043	4.9495	DG 53:6	C56H98O5	[M+NH4]+
868.7795334						
868.7795334	868.7752	0.0043	4.9495	DG O-53:7;O	C56H98O5	[M+NH4]+
	868.7752	0.0043	4.9495	TG O-53:6	C56H98O5	[M+NH4]+
868.7795334						
869.3269602	869.3249	0.0020	2.3006	PIP2 25:2	C34H63O19P3	[M+H]+
	869.3249	0.0020	2.3006	PIP2 25:1;O	C34H65O20P3	[M+H-H2O]+
869.3269602						
869.3269602	869.3249	0.0021	2.4157	PIP 30:8	C39H60O16P2Na	[M+Na]+

	869.325	0.0019	2.1856	PIP 27:3;O	C36H64O17P2K	[M+K]+
869.3269602						
869.6090202	869.6078	0.0012	1.3799	TG 56:16	C59H82O6	[M+H-H2O]+
	869.6097	0.0007	0.8050	HexCer 41:6;O6	C47H81NO12	[M+NH4]+
869.6090202						
869.6103745	869.6114	0.0010	1.1499	PI O-36:0;O	C45H89O13P	[M+H]+
	869.6113	0.0009	1.0349	MGDG 41:5	C50H86O10Na	[M+Na]+
869.6103745						
869.6103745	869.6097	0.0007	0.8050	HexCer 41:6;O6	C47H81NO12	[M+NH4]+
	869.6994	0.0003	0.3449	PA 48:2	C51H97O8P	[M+H]+
869.6991083						
869.6991083	869.6994	0.0003	0.3449	PA O-48:3;O	C51H97O8P	[M+H]+
	869.6994	0.0003	0.3449	PA 48:1;O	C51H99O9P	[M+H-H2O]+
869.6991083						
869.6991083	869.6994	0.0003	0.3449	PG O-45:2	C51H99O9P	[M+H-H2O]+
	869.6993	0.0002	0.2300	CE 29:4;O3	C56H94O5Na	[M+Na]+
869.6991083						
869.6991083	869.6993	0.0002	0.2300	DG 53:8	C56H94O5Na	[M+Na]+
	869.6993	0.0002	0.2300	DG O-53:9;O	C56H94O5Na	[M+Na]+
869.6991083						
869.6991083	869.6993	0.0002	0.2300	TG O-53:8	C56H94O5Na	[M+Na]+
	869.6995	0.0004	0.4599	DG 50:3;O	C53H98O6K	[M+K]+
869.6991083						
869.6991083	869.6995	0.0004	0.4599	DG O-50:4;O2	C53H98O6K	[M+K]+
	869.6995	0.0004	0.4599	TG 50:2	C53H98O6K	[M+K]+
869.6991083						
869.6991083	869.6995	0.0004	0.4599	TG O-50:3;O	C53H98O6K	[M+K]+
	869.6994	0.0010	1.1498	PA 48:2	C51H97O8P	[M+H]+
869.7003473						
869.7003473	869.6994	0.0010	1.1498	PA O-48:3;O	C51H97O8P	[M+H]+
	869.7017	0.0014	1.6097	CE 31:7;O3	C58H92O5	[M+H]+
869.7003473						
869.7003473	869.7017	0.0014	1.6097	DG 55:11	C58H92O5	[M+H]+
	869.7017	0.0014	1.6097	DG O-55:12;O	C58H92O5	[M+H]+
869.7003473						
869.7003473	869.7017	0.0014	1.6097	TG O-55:11	C58H92O5	[M+H]+
	869.6994	0.0010	1.1498	PA 48:1;O	C51H99O9P	[M+H-H2O]+

869.7003473						
869.7003473	869.6994	0.0010	1.1498	PG O-45:2	C51H99O9P	[M+H-H2O]+
	869.7017	0.0014	1.6097	CE 31:6;O4	C58H94O6	[M+H-H2O]+
869.7003473						
869.7003473	869.7017	0.0014	1.6097	DG 55:10;O	C58H94O6	[M+H-H2O]+
	869.7017	0.0014	1.6097	DG O-55:11;O2	C58H94O6	[M+H-H2O]+
869.7003473						
869.7003473	869.7017	0.0014	1.6097	TG 55:9	C58H94O6	[M+H-H2O]+
	869.7017	0.0014	1.6097	TG O-55:10;O	C58H94O6	[M+H-H2O]+
869.7003473						
869.7003473	869.6993	0.0010	1.1498	CE 29:4;O3	C56H94O5Na	[M+Na]+
	869.6993	0.0010	1.1498	DG 53:8	C56H94O5Na	[M+Na]+
869.7003473						
869.7003473	869.6993	0.0010	1.1498	DG O-53:9;O	C56H94O5Na	[M+Na]+
	869.6993	0.0010	1.1498	TG O-53:8	C56H94O5Na	[M+Na]+
869.7003473						
869.7003473	869.6995	0.0009	1.0348	DG 50:3;O	C53H98O6K	[M+K]+
	869.6995	0.0009	1.0348	DG O-50:4;O2	C53H98O6K	[M+K]+
869.7003473						
869.7003473	869.6995	0.0009	1.0348	TG 50:2	C53H98O6K	[M+K]+
	869.6995	0.0009	1.0348	TG O-50:3;O	C53H98O6K	[M+K]+
869.7003473						
869.7930370	869.7932	0.0002	0.2299	DG 52:1	C55H106O5Na	[M+Na]+
	869.7932	0.0002	0.2299	DG O-52:2;O	C55H106O5Na	[M+Na]+
869.7930370						
869.7930370	869.7932	0.0002	0.2299	TG O-52:1	C55H106O5Na	[M+Na]+
	870.5855	0.0005	0.5743	IPC 41:5;O2	C47H84NO11P	[M+H]+
870.5859735						
870.5859735	870.5855	0.0005	0.5743	PS 41:4;O	C47H84NO11P	[M+H]+
	870.5855	0.0005	0.5743	IPC 41:4;O3	C47H86NO12P	[M+H-H2O]+
870.5859735						
870.5859735	870.5855	0.0005	0.5743	PG 41:6;O	C47H81O11P	[M+NH4]+
	870.5856	0.0004	0.4595	HexCer 43:6;O3	C49H85NO9K	[M+K]+
870.5859735						
870.6219535	870.6219	0.0001	0.1149	CerP 48:5;O6	C48H88NO10P	[M+H]+
	870.6219	0.0001	0.1149	PS 42:3	C48H88NO10P	[M+H]+
870.6219535						
870.6219535	870.6219	0.0001	0.1149	PS O-42:4;O	C48H88NO10P	[M+H]+

870.6219535	870.6219	0.0001	0.1149	IPC 42:3;O2	C48H90NO11P	[M+H-H2O]+
870.6219535	870.6219	0.0001	0.1149	PS 42:2;O	C48H90NO11P	[M+H-H2O]+
870.6219535	870.6219	0.0001	0.1149	PG 42:5	C48H85O10P	[M+NH4]+
870.6219535	870.6219	0.0001	0.1149	PG O-42:6;O	C48H85O10P	[M+NH4]+
870.6219535	870.622	0.0000	0.0000	ACer 50:5;O6	C50H89NO8K	[M+K]+
870.6219535	870.622	0.0000	0.0000	HexCer 44:5;O2	C50H89NO8K	[M+K]+
870.6555199	870.6558	0.0003	0.3446	CerP 47:1;O5	C47H94NO9PNa	[M+Na]+
870.6555199	870.6558	0.0003	0.3446	PC 39:0;O	C47H94NO9PNa	[M+Na]+
870.6555199	870.6558	0.0003	0.3446	PE 42:0;O	C47H94NO9PNa	[M+Na]+
870.6555199	870.6558	0.0003	0.3446	PS O-41:0	C47H94NO9PNa	[M+Na]+
870.7027040	870.7029	0.0002	0.2297	HexCer 44:2;O4	C50H95NO10	[M+H]+
870.7027040	870.7029	0.0002	0.2297	HexCer 44:1;O5	C50H97NO11	[M+H-H2O]+
870.7027040	870.7029	0.0002	0.2297	MGDG 41:2	C50H92O10	[M+NH4]+
874.5365994	874.5357	0.0009	1.0291	PC 42:11	C50H78NO8PNa	[M+Na]+
874.5365994	874.5357	0.0009	1.0291	PC O-42:12;O	C50H78NO8PNa	[M+Na]+
874.5365994	874.5357	0.0009	1.0291	PE 45:11	C50H78NO8PNa	[M+Na]+
874.5365994	874.5357	0.0009	1.0291	PE O-45:12;O	C50H78NO8PNa	[M+Na]+
874.5365994	874.5359	0.0007	0.8004	PC 39:6;O	C47H82NO9PK	[M+K]+
874.5365994	874.5359	0.0007	0.8004	PE 42:6;O	C47H82NO9PK	[M+K]+
874.5365994	874.5359	0.0007	0.8004	PS O-41:6	C47H82NO9PK	[M+K]+
874.7855872	874.7858	0.0002	0.2286	ACer 55:3;O4	C55H103NO6	[M+H]+
874.7855872	874.7858	0.0002	0.2286	ACer 55:2;O5	C55H105NO7	[M+H-H2O]+
874.7855872	874.7858	0.0002	0.2286	CE 28:0;O4	C55H100O6	[M+NH4]+
874.7855872	874.7858	0.0002	0.2286	DG 52:4;O	C55H100O6	[M+NH4]+
874.7855872	874.7858	0.0002	0.2286	DG O-52:5;O2	C55H100O6	[M+NH4]+
874.7855872	874.7858	0.0002	0.2286	TG 52:3	C55H100O6	[M+NH4]+

	874.7858	0.0002	0.2286	TG O-52:4;O	C55H100O6	[M+NH4]+
874.7855872						
876.3324668	876.3307	0.0017	1.9399	PIP2 23:1;O	C32H61O20P3	[M+NH4]+
	876.7991	0.0006	0.6843	Cer 53:0;O5	C53H107NO6Na	[M+Na]+
876.7996313						
876.8013617	876.8015	0.0001	0.1141	ACer 55:2;O4	C55H105NO6	[M+H]+
	876.8015	0.0001	0.1141	ACer 55:1;O5	C55H107NO7	[M+H-H2O]+
876.8013617						
876.8013617	876.8015	0.0001	0.1141	DG 52:3;O	C55H102O6	[M+NH4]+
	876.8015	0.0001	0.1141	DG O-52:4;O2	C55H102O6	[M+NH4]+
876.8013617						
876.8013617	876.8015	0.0001	0.1141	TG 52:2	C55H102O6	[M+NH4]+
	876.8015	0.0001	0.1141	TG O-52:3;O	C55H102O6	[M+NH4]+
876.8013617						
877.5670385	877.5678	0.0007	0.7977	EPC 44:6;O6	C46H83N2O10PNa	[M+Na]+
	877.5678	0.0007	0.7977	SM 41:6;O6	C46H83N2O10PNa	[M+Na]+
877.5670385						
877.5670385	877.5665	0.0005	0.5698	SHexCer 35:0;O6	C41H81NO15S	[M+NH4]+
	877.8007	0.0040	4.5568	CE 32:2;O2	C59H104O4	[M+H]+
877.8047054						
877.8047054	877.8007	0.0040	4.5568	DG O-56:7	C59H104O4	[M+H]+
	877.8007	0.0040	4.5568	CE 32:1;O3	C59H106O5	[M+H-H2O]+
877.8047054						
877.8047054	877.8007	0.0040	4.5568	DG 56:5	C59H106O5	[M+H-H2O]+
	877.8007	0.0040	4.5568	DG O-56:6;O	C59H106O5	[M+H-H2O]+
877.8047054						
877.8047054	877.8007	0.0040	4.5568	TG O-56:5	C59H106O5	[M+H-H2O]+
	878.5882	0.0007	0.7967	CerP 47:5;O6	C47H86NO10PNa	[M+Na]+
878.5888999						
878.5888999	878.5882	0.0007	0.7967	PS 41:3	C47H86NO10PNa	[M+Na]+
	878.5882	0.0007	0.7967	PS O-41:4;O	C47H86NO10PNa	[M+Na]+
878.5888999						
878.5907823	878.5906	0.0002	0.2276	PS 43:6	C49H84NO10P	[M+H]+
	878.5906	0.0002	0.2276	PS O-43:7;O	C49H84NO10P	[M+H]+
878.5907823						
878.5907823	878.5906	0.0002	0.2276	IPC 43:6;O2	C49H86NO11P	[M+H-H2O]+
	878.5906	0.0002	0.2276	PS 43:5;O	C49H86NO11P	[M+H-H2O]+

878.5907823						
878.5907823	878.5906	0.0002	0.2276	PG 43:8	C49H81O10P	[M+NH4]+
	878.5906	0.0002	0.2276	PG O-43:9;O	C49H81O10P	[M+NH4]+
878.5907823						
878.6969892	878.6973	0.0003	0.3414	CerP 50:2;O3	C50H98NO7PNa	[M+Na]+
	878.6973	0.0003	0.3414	PC O-42:2	C50H98NO7PNa	[M+Na]+
878.6969892						
878.6969892	878.6973	0.0003	0.3414	PE O-45:2	C50H98NO7PNa	[M+Na]+
	879.3457	0.0026	2.9567	PIP2 27:2	C36H67O19P3	[M+H-H2O]+
879.3482739						
879.3482739	879.3458	0.0025	2.8430	PIP 29:4	C38H66O16P2K	[M+K]+
	879.5834	0.0018	2.0464	EPC 44:5;O6	C46H85N2O10PNa	[M+Na]+
879.5815585						
879.5815585	879.5834	0.0018	2.0464	SM 41:5;O6	C46H85N2O10PNa	[M+Na]+
	879.7396	0.0001	0.1137	ACer 52:4;O6	C52H95NO8	[M+NH4]+
879.7397006						
879.7397006	879.7396	0.0001	0.1137	HexCer 46:4;O2	C52H95NO8	[M+NH4]+
	879.7412	0.0001	0.1137	CE 28:0;O4	C55H100O6Na	[M+Na]+
879.7410984						
879.7410984	879.7412	0.0001	0.1137	DG 52:4;O	C55H100O6Na	[M+Na]+
	879.7412	0.0001	0.1137	DG O-52:5;O2	C55H100O6Na	[M+Na]+
879.7410984						
879.7410984	879.7412	0.0001	0.1137	TG 52:3	C55H100O6Na	[M+Na]+
	879.7412	0.0001	0.1137	TG O-52:4;O	C55H100O6Na	[M+Na]+
879.7410984						
880.3627548	880.3644	0.0016	1.8174	PIP 30:8;O	C39H60O17P2	[M+NH4]+
	880.5851	0.0005	0.5678	PC 44:11	C52H82NO8P	[M+H]+
880.5845411						
880.5845411	880.5851	0.0005	0.5678	PC O-44:12;O	C52H82NO8P	[M+H]+
	880.5851	0.0005	0.5678	PE 47:11	C52H82NO8P	[M+H]+
880.5845411						
880.5845411	880.5851	0.0005	0.5678	PE O-47:12;O	C52H82NO8P	[M+H]+
	880.5851	0.0005	0.5678	PC 44:10;O	C52H84NO9P	[M+H-H2O]+
880.5845411						
880.5845411	880.5851	0.0005	0.5678	PE 47:10;O	C52H84NO9P	[M+H-H2O]+
	880.5851	0.0005	0.5678	PS O-46:10	C52H84NO9P	[M+H-H2O]+
880.5845411						
880.5845411	880.5851	0.0005	0.5678	PA 49:12	C52H79O8P	[M+NH4]+

	880.6062	0.0002	0.2271	PS 43:5	C49H86NO10P	[M+H]+
880.6059789						
880.6059789	880.6062	0.0002	0.2271	PS O-43:6;O	C49H86NO10P	[M+H]+
	880.6062	0.0002	0.2271	IPC 43:5;O2	C49H88NO11P	[M+H-H2O]+
880.6059789						
880.6059789	880.6062	0.0002	0.2271	PS 43:4;O	C49H88NO11P	[M+H-H2O]+
	880.6062	0.0002	0.2271	PG 43:7	C49H83O10P	[M+NH4]+
880.6059789						
880.6059789	880.6062	0.0002	0.2271	PG O-43:8;O	C49H83O10P	[M+NH4]+
	880.7477	0.0033	3.7468	EPC 46:0;O4	C48H99N2O8P	[M+NH4]+
880.7444148						
880.7444148	880.7477	0.0033	3.7468	SM 43:0;O4	C48H99N2O8P	[M+NH4]+
	881.6114	0.0021	2.3820	PI 37:0	C46H89O13P	[M+H]+
881.6092335						
881.6092335	881.6114	0.0021	2.3820	PI O-37:1;O	C46H89O13P	[M+H]+
	881.6113	0.0021	2.3820	MGDG 42:6	C51H86O10Na	[M+Na]+
881.6092335						
881.6104292	881.6114	0.0009	1.0209	PI 37:0	C46H89O13P	[M+H]+
	881.6114	0.0009	1.0209	PI O-37:1;O	C46H89O13P	[M+H]+
881.6104292						
881.6104292	881.6113	0.0009	1.0209	MGDG 42:6	C51H86O10Na	[M+Na]+
	881.7569	0.0001	0.1134	DG 52:3;O	C55H102O6Na	[M+Na]+
881.7567963						
881.7567963	881.7569	0.0001	0.1134	DG O-52:4;O2	C55H102O6Na	[M+Na]+
	881.7569	0.0001	0.1134	TG 52:2	C55H102O6Na	[M+Na]+
881.7567963						
881.7567963	881.7569	0.0001	0.1134	TG O-52:3;O	C55H102O6Na	[M+Na]+
	883.3042	0.0044	4.9813	PIP2 25:3;O	C34H61O20P3	[M+H]+
883.3085627						
883.7149051	883.715	0.0001	0.1132	PA 49:2	C52H99O8P	[M+H]+
	883.715	0.0001	0.1132	PA O-49:3;O	C52H99O8P	[M+H]+
883.7149051						
883.7149051	883.715	0.0001	0.1132	PA 49:1;O	C52H101O9P	[M+H-H2O]+
	883.715	0.0001	0.1132	PG O-46:2	C52H101O9P	[M+H-H2O]+
883.7149051						
883.7149051	883.715	0.0001	0.1132	CE 30:4;O3	C57H96O5Na	[M+Na]+
	883.715	0.0001	0.1132	DG 54:8	C57H96O5Na	[M+Na]+
883.7149051						
883.7149051	883.715	0.0001	0.1132	DG O-54:9;O	C57H96O5Na	[M+Na]+

	883.715	0.0001	0.1132	TG O-54:8	C57H96O5Na	[M+Na]+
883.7149051						
883.7149051	883.7151	0.0002	0.2263	DG 51:3;O	C54H100O6K	[M+K]+
	883.7151	0.0002	0.2263	DG O-51:4;O2	C54H100O6K	[M+K]+
883.7149051						
883.7149051	883.7151	0.0002	0.2263	TG 51:2	C54H100O6K	[M+K]+
	883.7151	0.0002	0.2263	TG O-51:3;O	C54H100O6K	[M+K]+
883.7149051						
883.7516089	883.7514	0.0002	0.2263	PA O-50:2	C53H103O7P	[M+H]+
	883.7514	0.0002	0.2263	PA 50:0	C53H105O8P	[M+H-H2O]+
883.7516089						
883.7516089	883.7514	0.0002	0.2263	PA O-50:1;O	C53H105O8P	[M+H-H2O]+
	883.7514	0.0002	0.2263	CE 31:3;O2	C58H100O4Na	[M+Na]+
883.7516089						
883.7516089	883.7514	0.0002	0.2263	DG O-55:8	C58H100O4Na	[M+Na]+
	883.7515	0.0001	0.1132	DG 52:2	C55H104O5K	[M+K]+
883.7516089						
883.7516089	883.7515	0.0001	0.1132	DG O-52:3;O	C55H104O5K	[M+K]+
	883.7515	0.0001	0.1132	TG O-52:2	C55H104O5K	[M+K]+
883.7516089						
884.5388682	884.54	0.0011	1.2436	SHexCer 37:2;O6	C43H81NO15S	[M+H]+
	884.54	0.0007	0.7914	SHexCer 37:2;O6	C43H81NO15S	[M+H]+
884.5406520						
884.5406520	884.5412	0.0006	0.6783	PS 42:7	C48H80NO10PNa	[M+Na]+
	884.5412	0.0006	0.6783	PS O-42:8;O	C48H80NO10PNa	[M+Na]+
884.5406520						
884.5406520	884.5414	0.0007	0.7914	IPC 39:3;O2	C45H84NO11PK	[M+K]+
	884.5414	0.0007	0.7914	PS 39:2;O	C45H84NO11PK	[M+K]+
884.5406520						
885.6085692	885.608	0.0006	0.6775	SHexCer 38:1;O4	C44H85NO13S	[M+NH4]+
	885.6094	0.0008	0.9033	EPC 44:2;O5	C46H91N2O9PK	[M+K]+
885.6085692						
885.6085692	885.6094	0.0008	0.9033	SM 41:2;O5	C46H91N2O9PK	[M+K]+
	885.612	0.0013	1.4679	SQDG 40:2	C49H90O12S	[M+H-H2O]+
885.6107081						
885.6107081	885.6116	0.0009	1.0162	PC 43:10	C51H82NO8P	[M+NH4]+
	885.6116	0.0009	1.0162	PC O-43:11;O	C51H82NO8P	[M+NH4]+

885.6107081						
885.6107081	885.6116	0.0009	1.0162	PE 46:10	C51H82NO8P	[M+NH4]+
	885.6116	0.0009	1.0162	PE O-46:11;O	C51H82NO8P	[M+NH4]+
885.6107081						
885.6107081	885.6094	0.0013	1.4679	EPC 44:2;O5	C46H91N2O9PK	[M+K]+
	885.6094	0.0013	1.4679	SM 41:2;O5	C46H91N2O9PK	[M+K]+
885.6107081						
886.5931430	886.5932	0.0001	0.1128	PC 41:6;O	C49H86NO9PNa	[M+Na]+
	886.5932	0.0001	0.1128	PE 44:6;O	C49H86NO9PNa	[M+Na]+
886.5931430						
886.5931430	886.5932	0.0001	0.1128	PS O-43:6	C49H86NO9PNa	[M+Na]+
	886.5934	0.0002	0.2256	CerP 46:2;O6	C46H90NO10PK	[M+K]+
886.5931430						
886.5931430	886.5934	0.0002	0.2256	PS 40:0	C46H90NO10PK	[M+K]+
	886.5934	0.0002	0.2256	PS O-40:1;O	C46H90NO10PK	[M+K]+
886.5931430						
888.5732593	888.5725	0.0008	0.9003	PS 42:5	C48H84NO10PNa	[M+Na]+
	888.5725	0.0008	0.9003	PS O-42:6;O	C48H84NO10PNa	[M+Na]+
888.5732593						
888.5732593	888.5727	0.0006	0.6752	IPC 39:1;O2	C45H88NO11PK	[M+K]+
	888.5727	0.0006	0.6752	PS 39:0;O	C45H88NO11PK	[M+K]+
888.5732593						
888.6086429	888.6089	0.0002	0.2251	CerP 49:6;O5	C49H88NO9PNa	[M+Na]+
	888.6089	0.0002	0.2251	PC 41:5;O	C49H88NO9PNa	[M+Na]+
888.6086429						
888.6086429	888.6089	0.0002	0.2251	PE 44:5;O	C49H88NO9PNa	[M+Na]+
	888.6089	0.0002	0.2251	PS O-43:5	C49H88NO9PNa	[M+Na]+
888.6086429						
888.6086429	888.609	0.0004	0.4501	CerP 46:1;O6	C46H92NO10PK	[M+K]+
	888.609	0.0004	0.4501	PS O-40:0;O	C46H92NO10PK	[M+K]+
888.6086429						
888.6106341	888.6113	0.0007	0.7877	PC 43:8;O	C51H86NO9P	[M+H]+
	888.6113	0.0007	0.7877	PE 46:8;O	C51H86NO9P	[M+H]+
888.6106341						
888.6106341	888.6113	0.0007	0.7877	PS O-45:8	C51H86NO9P	[M+H]+
	888.6113	0.0007	0.7877	PS 45:6	C51H88NO10P	[M+H-H2O]+
888.6106341						
888.6106341	888.6113	0.0007	0.7877	PS O-45:7;O	C51H88NO10P	[M+H-H2O]+

888.6106341	888.6113	0.0007	0.7877	PA 48:9;O	C51H83O9P	[M+NH4]+
888.6106341	888.6113	0.0007	0.7877	PG O-45:10	C51H83O9P	[M+NH4]+
889.5768726	889.5776	0.0008	0.8993	PI 36:0	C45H87O13PNa	[M+Na]+
889.5768726	889.5776	0.0008	0.8993	PI O-36:1;O	C45H87O13PNa	[M+Na]+
889.5768726	889.576	0.0008	0.8993	IPC 36:1;O6	C42H82NO15P	[M+NH4]+
890.6242363	890.6245	0.0003	0.3368	CerP 49:5;O5	C49H90NO9PNa	[M+Na]+
890.6242363	890.6245	0.0003	0.3368	PC 41:4;O	C49H90NO9PNa	[M+Na]+
890.6242363	890.6245	0.0003	0.3368	PE 44:4;O	C49H90NO9PNa	[M+Na]+
890.6242363	890.6245	0.0003	0.3368	PS O-43:4	C49H90NO9PNa	[M+Na]+
890.6242363	890.6247	0.0005	0.5614	CerP 46:0;O6	C46H94NO10PK	[M+K]+
893.7566660	893.7569	0.0002	0.2238	CE 29:0;O4	C56H102O6Na	[M+Na]+
893.7566660	893.7569	0.0002	0.2238	DG 53:4;O	C56H102O6Na	[M+Na]+
893.7566660	893.7569	0.0002	0.2238	DG O-53:5;O2	C56H102O6Na	[M+Na]+
893.7566660	893.7569	0.0002	0.2238	TG 53:3	C56H102O6Na	[M+Na]+
893.7566660	893.7569	0.0002	0.2238	TG O-53:4;O	C56H102O6Na	[M+Na]+
894.6216745	894.6219	0.0002	0.2236	PS 44:5	C50H88NO10P	[M+H]+
894.6216745	894.6219	0.0002	0.2236	PS O-44:6;O	C50H88NO10P	[M+H]+
894.6216745	894.6219	0.0002	0.2236	IPC 44:5;O2	C50H90NO11P	[M+H-H2O]+
894.6216745	894.6219	0.0002	0.2236	PS 44:4;O	C50H90NO11P	[M+H-H2O]+
894.6216745	894.6219	0.0002	0.2236	PG 44:7	C50H85O10P	[M+NH4]+
894.6216745	894.6219	0.0002	0.2236	PG O-44:8;O	C50H85O10P	[M+NH4]+
895.7149201	895.715	0.0001	0.1116	PA 50:3	C53H99O8P	[M+H]+
895.7149201	895.715	0.0001	0.1116	PA O-50:4;O	C53H99O8P	[M+H]+
895.7149201	895.715	0.0001	0.1116	PA 50:2;O	C53H101O9P	[M+H-H2O]+
895.7149201	895.715	0.0001	0.1116	PG O-47:3	C53H101O9P	[M+H-H2O]+
895.7149201	895.715	0.0001	0.1116	CE 31:5;O3	C58H96O5Na	[M+Na]+

	895.715	0.0001	0.1116	DG 55:9	C58H96O5Na	[M+Na]+
895.7149201						
895.7149201	895.715	0.0001	0.1116	DG O-55:10;O	C58H96O5Na	[M+Na]+
	895.715	0.0001	0.1116	TG O-55:9	C58H96O5Na	[M+Na]+
895.7149201						
895.7149201	895.7151	0.0002	0.2233	CE 28:0;O4	C55H100O6K	[M+K]+
	895.7151	0.0002	0.2233	DG 52:4;O	C55H100O6K	[M+K]+
895.7149201						
895.7149201	895.7151	0.0002	0.2233	DG O-52:5;O2	C55H100O6K	[M+K]+
	895.7151	0.0002	0.2233	TG 52:3	C55H100O6K	[M+K]+
895.7149201						
895.7149201	895.7151	0.0002	0.2233	TG O-52:4;O	C55H100O6K	[M+K]+
	895.7725	0.0002	0.2233	DG 53:3;O	C56H104O6Na	[M+Na]+
895.7723462						
895.7723462	895.7725	0.0002	0.2233	DG O-53:4;O2	C56H104O6Na	[M+Na]+
	895.7725	0.0002	0.2233	TG 53:2	C56H104O6Na	[M+Na]+
895.7723462						
895.7723462	895.7725	0.0002	0.2233	TG O-53:3;O	C56H104O6Na	[M+Na]+
	895.8089	0.0003	0.3349	DG 54:2	C57H108O5Na	[M+Na]+
895.8085533						
895.8085533	895.8089	0.0003	0.3349	DG O-54:3;O	C57H108O5Na	[M+Na]+
	895.8089	0.0003	0.3349	TG O-54:2	C57H108O5Na	[M+Na]+
895.8085533						
896.7182671	896.7185	0.0003	0.3346	HexCer 46:3;O4	C52H97NO10	[M+H]+
	896.7185	0.0003	0.3346	HexCer 46:2;O5	C52H99NO11	[M+H-H2O]+
896.7182671						
896.7182671	896.7185	0.0003	0.3346	MGDG 43:3	C52H94O10	[M+NH4]+
	897.3562	0.0015	1.6716	PIP2 27:2	C36H67O19P3	[M+H]+
897.3577212						
897.3577212	897.3562	0.0015	1.6716	PIP2 27:1;O	C36H69O20P3	[M+H-H2O]+
	897.3562	0.0015	1.6716	PIP 32:8	C41H64O16P2Na	[M+Na]+
897.3577212						
897.3577212	897.3563	0.0014	1.5601	PIP 29:3;O	C38H68O17P2K	[M+K]+
	897.6391	0.0003	0.3342	TG 58:16	C61H86O6	[M+H-H2O]+
897.6388735						
897.6409423	897.641	0.0001	0.1114	HexCer 43:6;O6	C49H85NO12	[M+NH4]+
	897.7307	0.0012	1.3367	PA 50:2	C53H101O8P	[M+H]+

897.7294762						
897.7294762	897.7307	0.0012	1.3367	PA O-50:3;O	C53H101O8P	[M+H]+
	897.7307	0.0012	1.3367	PA 50:1;O	C53H103O9P	[M+H-H2O]+
897.7294762						
897.7294762	897.7307	0.0012	1.3367	PG O-47:2	C53H103O9P	[M+H-H2O]+
	897.7283	0.0012	1.3367	PA O-48:0;O	C51H103O8PNa	[M+Na]+
897.7294762						
897.7294762	897.7306	0.0012	1.3367	CE 31:4;O3	C58H98O5Na	[M+Na]+
	897.7306	0.0012	1.3367	DG 55:8	C58H98O5Na	[M+Na]+
897.7294762						
897.7294762	897.7306	0.0012	1.3367	DG O-55:9;O	C58H98O5Na	[M+Na]+
	897.7306	0.0012	1.3367	TG O-55:8	C58H98O5Na	[M+Na]+
897.7294762						
897.7294762	897.7308	0.0013	1.4481	DG 52:3;O	C55H102O6K	[M+K]+
	897.7308	0.0013	1.4481	DG O-52:4;O2	C55H102O6K	[M+K]+
897.7294762						
897.7294762	897.7308	0.0013	1.4481	TG 52:2	C55H102O6K	[M+K]+
	897.7308	0.0013	1.4481	TG O-52:3;O	C55H102O6K	[M+K]+
897.7294762						
897.7307950	897.7307	0.0001	0.1114	PA 50:2	C53H101O8P	[M+H]+
	897.7307	0.0001	0.1114	PA O-50:3;O	C53H101O8P	[M+H]+
897.7307950						
897.7307950	897.7307	0.0001	0.1114	PA 50:1;O	C53H103O9P	[M+H-H2O]+
	897.7307	0.0001	0.1114	PG O-47:2	C53H103O9P	[M+H-H2O]+
897.7307950						
897.7307950	897.7306	0.0002	0.2228	CE 31:4;O3	C58H98O5Na	[M+Na]+
	897.7306	0.0002	0.2228	DG 55:8	C58H98O5Na	[M+Na]+
897.7307950						
897.7307950	897.7306	0.0002	0.2228	DG O-55:9;O	C58H98O5Na	[M+Na]+
	897.7306	0.0002	0.2228	TG O-55:8	C58H98O5Na	[M+Na]+
897.7307950						
897.7307950	897.7308	0.0000	0.0000	DG 52:3;O	C55H102O6K	[M+K]+
	897.7308	0.0000	0.0000	DG O-52:4;O2	C55H102O6K	[M+K]+
897.7307950						
897.7307950	897.7308	0.0000	0.0000	TG 52:2	C55H102O6K	[M+K]+
	897.7308	0.0000	0.0000	TG O-52:3;O	C55H102O6K	[M+K]+
897.7307950						
898.7339159	898.7342	0.0003	0.3338	HexCer 46:2;O4	C52H99NO10	[M+H]+

	898.7342	0.0003	0.3338	HexCer 46:1;O5	C52H101NO11	[M+H-H2O]+
898.7339159						
898.7339159	898.7342	0.0003	0.3338	MGDG 43:2	C52H96O10	[M+NH4]+
	899.6203	0.0009	1.0004	Hex2Cer 36:5;O2	C48H83NO13	[M+NH4]+
899.6211335						
899.6211335	899.6236	0.0025	2.7789	SHexCer 39:1;O4	C45H87NO13S	[M+NH4]+
	899.7099	0.0007	0.7780	PA 49:2;O	C52H99O9P	[M+H]+
899.7092219						
899.7092219	899.7099	0.0007	0.7780	PG O-46:3	C52H99O9P	[M+H]+
	899.7099	0.0007	0.7780	PG 46:1	C52H101O10P	[M+H-H2O]+
899.7092219						
899.7092219	899.7099	0.0007	0.7780	PG O-46:2;O	C52H101O10P	[M+H-H2O]+
	899.7099	0.0007	0.7780	CE 30:4;O4	C57H96O6Na	[M+Na]+
899.7092219						
899.7092219	899.7099	0.0007	0.7780	DG 54:8;O	C57H96O6Na	[M+Na]+
	899.7099	0.0007	0.7780	DG O-54:9;O2	C57H96O6Na	[M+Na]+
899.7092219						
899.7092219	899.7099	0.0007	0.7780	TG 54:7	C57H96O6Na	[M+Na]+
	899.7099	0.0007	0.7780	TG O-54:8;O	C57H96O6Na	[M+Na]+
899.7092219						
899.7092219	899.7101	0.0008	0.8892	DG 51:3;O2	C54H100O7K	[M+K]+
	899.7101	0.0008	0.8892	TG 51:2;O	C54H100O7K	[M+K]+
899.7092219						
899.7092219	899.7101	0.0008	0.8892	TG O-51:3;O2	C54H100O7K	[M+K]+
	899.7099	0.0006	0.6669	PA 49:2;O	C52H99O9P	[M+H]+
899.7105798						
899.7105798	899.7099	0.0006	0.6669	PG O-46:3	C52H99O9P	[M+H]+
	899.7099	0.0006	0.6669	PG 46:1	C52H101O10P	[M+H-H2O]+
899.7105798						
899.7105798	899.7099	0.0006	0.6669	PG O-46:2;O	C52H101O10P	[M+H-H2O]+
	899.7099	0.0007	0.7780	CE 30:4;O4	C57H96O6Na	[M+Na]+
899.7105798						
899.7105798	899.7099	0.0007	0.7780	DG 54:8;O	C57H96O6Na	[M+Na]+
	899.7099	0.0007	0.7780	DG O-54:9;O2	C57H96O6Na	[M+Na]+
899.7105798						
899.7105798	899.7099	0.0007	0.7780	TG 54:7	C57H96O6Na	[M+Na]+
	899.7099	0.0007	0.7780	TG O-54:8;O	C57H96O6Na	[M+Na]+
899.7105798						
899.7105798	899.7101	0.0005	0.5557	DG 51:3;O2	C54H100O7K	[M+K]+

	899.7101	0.0005	0.5557	TG 51:2;O	C54H100O7K	[M+K]+
899.7105798						
899.7105798	899.7101	0.0005	0.5557	TG O-51:3;O2	C54H100O7K	[M+K]+
	901.7256	0.0002	0.2218	PA 49:1;O	C52H101O9P	[M+H]+
901.7253539						
901.7253539	901.7256	0.0002	0.2218	PG O-46:2	C52H101O9P	[M+H]+
	901.7256	0.0002	0.2218	PG 46:0	C52H103O10P	[M+H-H2O]+
901.7253539						
901.7253539	901.7256	0.0002	0.2218	PG O-46:1;O	C52H103O10P	[M+H-H2O]+
	901.7256	0.0002	0.2218	CE 30:3;O4	C57H98O6Na	[M+Na]+
901.7253539						
901.7253539	901.7256	0.0002	0.2218	DG 54:7;O	C57H98O6Na	[M+Na]+
	901.7256	0.0002	0.2218	DG O-54:8;O2	C57H98O6Na	[M+Na]+
901.7253539						
901.7253539	901.7256	0.0002	0.2218	TG 54:6	C57H98O6Na	[M+Na]+
	901.7256	0.0002	0.2218	TG O-54:7;O	C57H98O6Na	[M+Na]+
901.7253539						
901.7253539	901.7257	0.0004	0.4436	DG 51:2;O2	C54H102O7K	[M+K]+
	901.7257	0.0004	0.4436	TG 51:1;O	C54H102O7K	[M+K]+
901.7253539						
901.7253539	901.7257	0.0004	0.4436	TG O-51:2;O2	C54H102O7K	[M+K]+
	902.5882	0.0007	0.7755	PS 43:5	C49H86NO10PNa	[M+Na]+
902.5888527						
902.5888527	902.5882	0.0007	0.7755	PS O-43:6;O	C49H86NO10PNa	[M+Na]+
	902.5883	0.0005	0.5540	IPC 40:1;O2	C46H90NO11PK	[M+K]+
902.5888527						
902.5888527	902.5883	0.0005	0.5540	PS 40:0;O	C46H90NO11PK	[M+K]+
	902.5906	0.0001	0.1108	PS 45:8	C51H84NO10P	[M+H]+
902.5906496						
902.5906496	902.5906	0.0001	0.1108	PS O-45:9;O	C51H84NO10P	[M+H]+
	902.5906	0.0001	0.1108	PS 45:7;O	C51H86NO11P	[M+H-H2O]+
902.5906496						
902.5906496	902.5906	0.0001	0.1108	PG 45:10	C51H81O10P	[M+NH4]+
	902.5906	0.0001	0.1108	PG O-45:11;O	C51H81O10P	[M+NH4]+
902.5906496						
902.8169142	902.8171	0.0002	0.2215	ACer 57:3;O4	C57H107NO6	[M+H]+
	902.8171	0.0002	0.2215	ACer 57:2;O5	C57H109NO7	[M+H-H2O]+

902.8169142						
902.8169142	902.8171	0.0002	0.2215	CE 30:0;O4	C57H104O6	[M+NH4]+
	902.8171	0.0002	0.2215	DG 54:4;O	C57H104O6	[M+NH4]+
902.8169142						
902.8169142	902.8171	0.0002	0.2215	DG O-54:5;O2	C57H104O6	[M+NH4]+
	902.8171	0.0002	0.2215	TG 54:3	C57H104O6	[M+NH4]+
902.8169142						
902.8169142	902.8171	0.0002	0.2215	TG O-54:4;O	C57H104O6	[M+NH4]+
	903.5933	0.0001	0.1107	PI 37:0	C46H89O13PNa	[M+Na]+
903.5934129						
903.5934129	903.5933	0.0001	0.1107	PI O-37:1;O	C46H89O13PNa	[M+Na]+
	903.7412	0.0017	1.8811	PA 49:0;O	C52H103O9P	[M+H]+
903.7394940						
903.7394940	903.7396	0.0001	0.1107	ACer 54:6;O6	C54H95NO8	[M+NH4]+
	903.7396	0.0001	0.1107	HexCer 48:6;O2	C54H95NO8	[M+NH4]+
903.7394940						
903.7412512	903.7412	0.0000	0.0000	PA 49:0;O	C52H103O9P	[M+H]+
	903.7412	0.0000	0.0000	PG O-46:1	C52H103O9P	[M+H]+
903.7412512						
903.7412512	903.7412	0.0000	0.0000	PG O-46:0;O	C52H105O10P	[M+H-H2O]+
	903.7412	0.0000	0.0000	CE 30:2;O4	C57H100O6Na	[M+Na]+
903.7412512						
903.7412512	903.7412	0.0000	0.0000	DG 54:6;O	C57H100O6Na	[M+Na]+
	903.7412	0.0000	0.0000	DG O-54:7;O2	C57H100O6Na	[M+Na]+
903.7412512						
903.7412512	903.7412	0.0000	0.0000	TG 54:5	C57H100O6Na	[M+Na]+
	903.7412	0.0000	0.0000	TG O-54:6;O	C57H100O6Na	[M+Na]+
903.7412512						
903.7412512	903.7414	0.0001	0.1107	DG 51:1;O2	C54H104O7K	[M+K]+
	903.7414	0.0001	0.1107	TG 51:0;O	C54H104O7K	[M+K]+
903.7412512						
903.7412512	903.7414	0.0001	0.1107	TG O-51:1;O2	C54H104O7K	[M+K]+
	903.8164	0.0029	3.2086	CE 34:3;O2	C61H106O4	[M+H]+
903.8193194						
903.8193194	903.8164	0.0029	3.2086	DG O-58:8	C61H106O4	[M+H]+
	903.8164	0.0029	3.2086	CE 34:2;O3	C61H108O5	[M+H-H2O]+
903.8193194						
903.8193194	903.8164	0.0029	3.2086	DG 58:6	C61H108O5	[M+H-H2O]+

	903.8164	0.0029	3.2086	DG O-58:7;O	C61H108O5	[M+H-H2O]+
903.8193194						
903.8193194	903.8164	0.0029	3.2086	TG O-58:6	C61H108O5	[M+H-H2O]+
	903.8164	0.0043	4.7576	CE 34:3;O2	C61H106O4	[M+H]+
903.8207287						
903.8207287	903.8164	0.0043	4.7576	DG O-58:8	C61H106O4	[M+H]+
	903.8164	0.0043	4.7576	CE 34:2;O3	C61H108O5	[M+H-H2O]+
903.8207287						
903.8207287	903.8164	0.0043	4.7576	DG 58:6	C61H108O5	[M+H-H2O]+
	903.8164	0.0043	4.7576	DG O-58:7;O	C61H108O5	[M+H-H2O]+
903.8207287						
903.8207287	903.8164	0.0043	4.7576	TG O-58:6	C61H108O5	[M+H-H2O]+
	903.8252	0.0045	4.9788	CerP 53:0;O2	C53H108NO6P	[M+NH4]+
903.8207287						
904.6052341	904.6062	0.0010	1.1055	PS 45:7	C51H86NO10P	[M+H]+
	904.6062	0.0010	1.1055	PS O-45:8;O	C51H86NO10P	[M+H]+
904.6052341						
904.6052341	904.6062	0.0010	1.1055	PS 45:6;O	C51H88NO11P	[M+H-H2O]+
	904.6038	0.0014	1.5476	CerP 49:6;O6	C49H88NO10PNa	[M+Na]+
904.6052341						
904.6052341	904.6038	0.0014	1.5476	PS 43:4	C49H88NO10PNa	[M+Na]+
	904.6038	0.0014	1.5476	PS O-43:5;O	C49H88NO10PNa	[M+Na]+
904.6052341						
904.6052341	904.6062	0.0010	1.1055	PG 45:9	C51H83O10P	[M+NH4]+
	904.6062	0.0010	1.1055	PG O-45:10;O	C51H83O10P	[M+NH4]+
904.6052341						
904.6052341	904.604	0.0013	1.4371	IPC 40:0;O2	C46H92NO11PK	[M+K]+
	904.8328	0.0001	0.1105	ACer 57:2;O4	C57H109NO6	[M+H]+
904.8326454						
904.8326454	904.8328	0.0001	0.1105	ACer 57:1;O5	C57H111NO7	[M+H-H2O]+
	904.8328	0.0001	0.1105	DG 54:3;O	C57H106O6	[M+NH4]+
904.8326454						
904.8326454	904.8328	0.0001	0.1105	DG O-54:4;O2	C57H106O6	[M+NH4]+
	904.8328	0.0001	0.1105	TG 54:2	C57H106O6	[M+NH4]+
904.8326454						
904.8326454	904.8328	0.0001	0.1105	TG O-54:3;O	C57H106O6	[M+NH4]+
	905.6089	0.0003	0.3313	PI O-37:0;O	C46H91O13PNa	[M+Na]+
905.6086215						
905.6104696	905.6114	0.0009	0.9938	PI 39:2	C48H89O13P	[M+H]+

	905.6114	0.0009	0.9938	PI O-39:3;O	C48H89O13P	[M+H]+
905.6104696						
905.6104696	905.6114	0.0009	0.9938	PI 39:1;O	C48H91O14P	[M+H-H2O]+
	905.6113	0.0008	0.8834	MGDG 44:8	C53H86O10Na	[M+Na]+
905.6104696						
905.6104696	905.6089	0.0015	1.6563	PI O-37:0;O	C46H91O13PNa	[M+Na]+
	905.7569	0.0001	0.1104	PG O-46:0	C52H105O9P	[M+H]+
905.7568189						
905.7568189	905.7569	0.0000	0.0000	CE 30:1;O4	C57H102O6Na	[M+Na]+
	905.7569	0.0000	0.0000	DG 54:5;O	C57H102O6Na	[M+Na]+
905.7568189						
905.7568189	905.7569	0.0000	0.0000	DG O-54:6;O2	C57H102O6Na	[M+Na]+
	905.7569	0.0000	0.0000	TG 54:4	C57H102O6Na	[M+Na]+
905.7568189						
905.7568189	905.7569	0.0000	0.0000	TG O-54:5;O	C57H102O6Na	[M+Na]+
	905.757	0.0002	0.2208	DG 51:0;O2	C54H106O7K	[M+K]+
905.7568189						
905.7568189	905.757	0.0002	0.2208	TG O-51:0;O2	C54H106O7K	[M+K]+
	905.832	0.0040	4.4158	CE 34:2;O2	C61H108O4	[M+H]+
905.8360346						
905.8360346	905.832	0.0040	4.4158	DG O-58:7	C61H108O4	[M+H]+
	905.832	0.0040	4.4158	CE 34:1;O3	C61H110O5	[M+H-H2O]+
905.8360346						
905.8360346	905.832	0.0040	4.4158	DG 58:5	C61H110O5	[M+H-H2O]+
	905.832	0.0040	4.4158	DG O-58:6;O	C61H110O5	[M+H-H2O]+
905.8360346						
905.8360346	905.832	0.0040	4.4158	TG O-58:5	C61H110O5	[M+H-H2O]+
	906.6219	0.0001	0.1103	PS 45:6	C51H88NO10P	[M+H]+
906.6217860						
906.6217860	906.6219	0.0001	0.1103	PS O-45:7;O	C51H88NO10P	[M+H]+
	906.6219	0.0001	0.1103	IPC 45:6;O2	C51H90NO11P	[M+H-H2O]+
906.6217860						
906.6217860	906.6219	0.0001	0.1103	PS 45:5;O	C51H90NO11P	[M+H-H2O]+
	906.6219	0.0001	0.1103	PG 45:8	C51H85O10P	[M+NH4]+
906.6217860						
906.6217860	906.6219	0.0001	0.1103	PG O-45:9;O	C51H85O10P	[M+NH4]+
	907.627	0.0016	1.7628	PI 39:1	C48H91O13P	[M+H]+

907.6253648						
907.6253648	907.627	0.0016	1.7628	PI O-39:2;O	C48H91O13P	[M+H]+
	907.627	0.0016	1.7628	PI 39:0;O	C48H93O14P	[M+H-H2O]+
907.6253648						
907.6253648	907.627	0.0016	1.7628	MGDG 44:7	C53H88O10Na	[M+Na]+
	907.7725	0.0001	0.1102	CE 30:0;O4	C57H104O6Na	[M+Na]+
907.7723890						
907.7723890	907.7725	0.0001	0.1102	DG 54:4;O	C57H104O6Na	[M+Na]+
	907.7725	0.0001	0.1102	DG O-54:5;O2	C57H104O6Na	[M+Na]+
907.7723890						
907.7723890	907.7725	0.0001	0.1102	TG 54:3	C57H104O6Na	[M+Na]+
	907.7725	0.0001	0.1102	TG O-54:4;O	C57H104O6Na	[M+Na]+
907.7723890						
908.6372910	908.6375	0.0002	0.2201	PS 45:5	C51H90NO10P	[M+H]+
	908.6375	0.0002	0.2201	PS O-45:6;O	C51H90NO10P	[M+H]+
908.6372910						
908.6372910	908.6375	0.0002	0.2201	IPC 45:5;O2	C51H92NO11P	[M+H-H2O]+
	908.6375	0.0002	0.2201	PS 45:4;O	C51H92NO11P	[M+H-H2O]+
908.6372910						
908.6372910	908.6375	0.0002	0.2201	PG 45:7	C51H87O10P	[M+NH4]+
	908.6375	0.0002	0.2201	PG O-45:8;O	C51H87O10P	[M+NH4]+
908.6372910						
908.7757011	908.779	0.0033	3.6312	EPC 48:0;O4	C50H103N2O8P	[M+NH4]+
	908.779	0.0033	3.6312	SM 45:0;O4	C50H103N2O8P	[M+NH4]+
908.7757011						
909.6395983	909.6368	0.0028	3.0782	PA 52:10	C55H89O8P	[M+H]+
	909.6368	0.0028	3.0782	PA O-52:11;O	C55H89O8P	[M+H]+
909.6395983						
909.6395983	909.6427	0.0031	3.4079	PI 39:0	C48H93O13P	[M+H]+
	909.6427	0.0031	3.4079	PI O-39:1;O	C48H93O13P	[M+H]+
909.6395983						
909.6395983	909.6368	0.0028	3.0782	PA 52:9;O	C55H91O9P	[M+H-H2O]+
	909.6368	0.0028	3.0782	PG O-49:10	C55H91O9P	[M+H-H2O]+
909.6395983						
909.6395983	909.6367	0.0029	3.1881	TG O-57:16	C60H86O5Na	[M+Na]+
	909.6426	0.0030	3.2980	MGDG 44:6	C53H90O10Na	[M+Na]+
909.6395983						
909.6395983	909.6369	0.0027	2.9682	CE 30:7;O4	C57H90O6K	[M+K]+

	909.6369	0.0027	2.9682	DG 54:11;O	C57H90O6K	[M+K]+
909.6395983						
909.6395983	909.6369	0.0027	2.9682	DG O-54:12;O2	C57H90O6K	[M+K]+
	909.6369	0.0027	2.9682	TG 54:10	C57H90O6K	[M+K]+
909.6395983						
909.6395983	909.6369	0.0027	2.9682	TG O-54:11;O	C57H90O6K	[M+K]+
	909.6427	0.0017	1.8689	PI 39:0	C48H93O13P	[M+H]+
909.6409057						
909.6409057	909.6427	0.0017	1.8689	PI O-39:1;O	C48H93O13P	[M+H]+
	909.6426	0.0017	1.8689	MGDG 44:6	C53H90O10Na	[M+Na]+
909.6409057						
909.7882175	909.7882	0.0001	0.1099	DG 54:3;O	C57H106O6Na	[M+Na]+
	909.7882	0.0001	0.1099	DG O-54:4;O2	C57H106O6Na	[M+Na]+
909.7882175						
909.7882175	909.7882	0.0001	0.1099	TG 54:2	C57H106O6Na	[M+Na]+
	909.7882	0.0001	0.1099	TG O-54:3;O	C57H106O6Na	[M+Na]+
909.7882175						
910.3130329	910.3151	0.0021	2.3069	PIP2 26:5;O	C35H59O20P3	[M+NH4]+
	910.6532	0.0001	0.1098	CerP 51:6;O6	C51H92NO10P	[M+H]+
910.6532545						
910.6532545	910.6532	0.0001	0.1098	PS 45:4	C51H92NO10P	[M+H]+
	910.6532	0.0001	0.1098	PS O-45:5;O	C51H92NO10P	[M+H]+
910.6532545						
910.6532545	910.6532	0.0001	0.1098	IPC 45:4;O2	C51H94NO11P	[M+H-H2O]+
	910.6532	0.0001	0.1098	PS 45:3;O	C51H94NO11P	[M+H-H2O]+
910.6532545						
910.6532545	910.6532	0.0001	0.1098	PG 45:6	C51H89O10P	[M+NH4]+
	910.6532	0.0001	0.1098	PG O-45:7;O	C51H89O10P	[M+NH4]+
910.6532545						
910.6532545	910.6533	0.0000	0.0000	ACer 53:6;O6	C53H93NO8K	[M+K]+
	910.6533	0.0000	0.0000	HexCer 47:6;O2	C53H93NO8K	[M+K]+
910.6532545						
910.7896721	910.7858	0.0039	4.2820	ACer 58:6;O4	C58H103NO6	[M+H]+
	910.7858	0.0039	4.2820	ACer 58:5;O5	C58H105NO7	[M+H-H2O]+
910.7896721						
910.7896721	910.7858	0.0039	4.2820	CE 31:3;O4	C58H100O6	[M+NH4]+
	910.7858	0.0039	4.2820	DG 55:7;O	C58H100O6	[M+NH4]+
910.7896721						
910.7896721	910.7858	0.0039	4.2820	DG O-55:8;O2	C58H100O6	[M+NH4]+

	910.7858	0.0039	4.2820	TG 55:6	C58H100O6	[M+NH4]+
910.7896721						
910.7896721	910.7858	0.0039	4.2820	TG O-55:7;O	C58H100O6	[M+NH4]+
	911.3355	0.0035	3.8405	PIP2 27:3;O	C36H65O20P3	[M+H]+
911.3389939						
911.3389939	911.3354	0.0035	3.8405	PIP 32:9;O	C41H62O17P2Na	[M+Na]+
	911.6566	0.0002	0.2194	HexCer 44:6;O6	C50H87NO12	[M+NH4]+
911.6564780						
911.7462158	911.7463	0.0001	0.1097	PA 51:2	C54H103O8P	[M+H]+
	911.7463	0.0001	0.1097	PA O-51:3;O	C54H103O8P	[M+H]+
911.7462158						
911.7462158	911.7463	0.0001	0.1097	PA 51:1;O	C54H105O9P	[M+H-H2O]+
	911.7463	0.0001	0.1097	PG O-48:2	C54H105O9P	[M+H-H2O]+
911.7462158						
911.7462158	911.7463	0.0001	0.1097	CE 32:4;O3	C59H100O5Na	[M+Na]+
	911.7463	0.0001	0.1097	DG 56:8	C59H100O5Na	[M+Na]+
911.7462158						
911.7462158	911.7463	0.0001	0.1097	DG O-56:9;O	C59H100O5Na	[M+Na]+
	911.7463	0.0001	0.1097	TG O-56:8	C59H100O5Na	[M+Na]+
911.7462158						
911.7462158	911.7464	0.0002	0.2194	DG 53:3;O	C56H104O6K	[M+K]+
	911.7464	0.0002	0.2194	DG O-53:4;O2	C56H104O6K	[M+K]+
911.7462158						
911.7462158	911.7464	0.0002	0.2194	TG 53:2	C56H104O6K	[M+K]+
	911.7464	0.0002	0.2194	TG O-53:3;O	C56H104O6K	[M+K]+
911.7462158						
911.7932619	911.7939	0.0007	0.7677	EPC 52:2;O2	C54H107N2O6P	[M+H]+
	911.7939	0.0007	0.7677	SM 49:2;O2	C54H107N2O6P	[M+H]+
911.7932619						
911.7932619	911.7939	0.0007	0.7677	EPC 52:1;O3	C54H109N2O7P	[M+H-H2O]+
	911.7939	0.0007	0.7677	SM 49:1;O3	C54H109N2O7P	[M+H-H2O]+
911.7932619						
911.7932619	911.7939	0.0007	0.7677	CerP 54:3;O2	C54H104NO6P	[M+NH4]+
	911.8038	0.0001	0.1097	DG 54:2;O	C57H108O6Na	[M+Na]+
911.8039174						
911.8039174	911.8038	0.0001	0.1097	DG O-54:3;O2	C57H108O6Na	[M+Na]+
	911.8038	0.0001	0.1097	TG 54:1	C57H108O6Na	[M+Na]+

911.8039174						
911.8039174	911.8038	0.0001	0.1097	TG O-54:2;O	C57H108O6Na	[M+Na]+
	912.5713	0.0003	0.3287	SHexCer 39:2;O6	C45H85NO15S	[M+H]+
912.5716069						
912.5716069	912.5725	0.0009	0.9862	PS 44:7	C50H84NO10PNa	[M+Na]+
	912.5725	0.0009	0.9862	PS O-44:8;O	C50H84NO10PNa	[M+Na]+
912.5716069						
912.5716069	912.5727	0.0010	1.0958	IPC 41:3;O2	C47H88NO11PK	[M+K]+
	912.5727	0.0010	1.0958	PS 41:2;O	C47H88NO11PK	[M+K]+
912.5716069						
912.6086795	912.6077	0.0010	1.0958	SHexCer 40:1;O5	C46H89NO14S	[M+H]+
	912.6077	0.0010	1.0958	SHexCer 40:0;O6	C46H91NO15S	[M+H-H2O]+
912.6086795						
912.6086795	912.6089	0.0002	0.2192	PC 43:7;O	C51H88NO9PNa	[M+Na]+
	912.6089	0.0002	0.2192	PE 46:7;O	C51H88NO9PNa	[M+Na]+
912.6086795						
912.6086795	912.6089	0.0002	0.2192	PS O-45:7	C51H88NO9PNa	[M+Na]+
	912.609	0.0004	0.4383	CerP 48:3;O6	C48H92NO10PK	[M+K]+
912.6086795						
912.6086795	912.609	0.0004	0.4383	PS 42:1	C48H92NO10PK	[M+K]+
	912.609	0.0004	0.4383	PS O-42:2;O	C48H92NO10PK	[M+K]+
912.6086795						
913.7618788	913.762	0.0001	0.1094	PA 51:1	C54H105O8P	[M+H]+
	913.762	0.0001	0.1094	PA O-51:2;O	C54H105O8P	[M+H]+
913.7618788						
913.7618788	913.762	0.0001	0.1094	PA 51:0;O	C54H107O9P	[M+H-H2O]+
	913.762	0.0001	0.1094	PG O-48:1	C54H107O9P	[M+H-H2O]+
913.7618788						
913.7618788	913.7619	0.0001	0.1094	CE 32:3;O3	C59H102O5Na	[M+Na]+
	913.7619	0.0001	0.1094	DG 56:7	C59H102O5Na	[M+Na]+
913.7618788						
913.7618788	913.7619	0.0001	0.1094	DG O-56:8;O	C59H102O5Na	[M+Na]+
	913.7619	0.0001	0.1094	TG O-56:7	C59H102O5Na	[M+Na]+
913.7618788						
913.7618788	913.7621	0.0002	0.2189	DG 53:2;O	C56H106O6K	[M+K]+
	913.7621	0.0002	0.2189	DG O-53:3;O2	C56H106O6K	[M+K]+
913.7618788						
913.7618788	913.7621	0.0002	0.2189	TG 53:1	C56H106O6K	[M+K]+

	913.7621	0.0002	0.2189	TG O-53:2;O	C56H106O6K	[M+K]+
913.7618788						
914.6245385	914.6245	0.0000	0.0000	PC 43:6;O	C51H90NO9PNa	[M+Na]+
	914.6245	0.0000	0.0000	PE 46:6;O	C51H90NO9PNa	[M+Na]+
914.6245385						
914.6245385	914.6245	0.0000	0.0000	PS O-45:6	C51H90NO9PNa	[M+Na]+
	914.6247	0.0002	0.2187	CerP 48:2;O6	C48H94NO10PK	[M+K]+
914.6245385						
914.6245385	914.6247	0.0002	0.2187	PS 42:0	C48H94NO10PK	[M+K]+
	914.6247	0.0002	0.2187	PS O-42:1;O	C48H94NO10PK	[M+K]+
914.6245385						
914.7654652	914.7655	0.0000	0.0000	HexCer 47:1;O4	C53H103NO10	[M+H]+
	914.7655	0.0000	0.0000	HexCer 47:0;O5	C53H105NO11	[M+H-H2O]+
914.7654652						
914.7654652	914.7655	0.0000	0.0000	MGDG 44:1	C53H100O10	[M+NH4]+
	915.6262	0.0004	0.4369	PA 54:12	C57H89O8P	[M+H-H2O]+
915.6257943						
915.6257943	915.6263	0.0005	0.5461	DG 56:14	C59H88O5K	[M+K]+
	915.6263	0.0005	0.5461	TG O-56:14	C59H88O5K	[M+K]+
915.6257943						
915.7774640	915.7776	0.0002	0.2184	PA 51:0	C54H107O8P	[M+H]+
	915.7776	0.0002	0.2184	PA O-51:1;O	C54H107O8P	[M+H]+
915.7774640						
915.7774640	915.7776	0.0002	0.2184	PG O-48:0	C54H109O9P	[M+H-H2O]+
	915.7776	0.0001	0.1092	CE 32:2;O3	C59H104O5Na	[M+Na]+
915.7774640						
915.7774640	915.7776	0.0001	0.1092	DG 56:6	C59H104O5Na	[M+Na]+
	915.7776	0.0001	0.1092	DG O-56:7;O	C59H104O5Na	[M+Na]+
915.7774640						
915.7774640	915.7776	0.0001	0.1092	TG O-56:6	C59H104O5Na	[M+Na]+
	915.7777	0.0003	0.3276	DG 53:1;O	C56H108O6K	[M+K]+
915.7774640						
915.7774640	915.7777	0.0003	0.3276	DG O-53:2;O2	C56H108O6K	[M+K]+
	915.7777	0.0003	0.3276	TG 53:0	C56H108O6K	[M+K]+
915.7774640						
915.7774640	915.7777	0.0003	0.3276	TG O-53:1;O	C56H108O6K	[M+K]+
	915.7987	0.0003	0.3276	DG 53:1;O2	C56H108O7Na	[M+Na]+
915.7984189						
915.7984189	915.7987	0.0003	0.3276	TG 53:0;O	C56H108O7Na	[M+Na]+

915.7984189	915.7987	0.0003	0.3276	TG O-53:1;O2	C56H108O7Na	[M+Na]+
916.7815190	916.7811	0.0004	0.4363	HexCer 47:0;O4	C53H105NO10	[M+H]+
916.7815190	916.7811	0.0004	0.4363	MGDG 44:0	C53H102O10	[M+NH4]+
919.5667180	919.5671	0.0004	0.4350	PI O-40:6	C49H85O12PNa	[M+Na]+
919.5667180	919.5672	0.0005	0.5437	PI 37:0	C46H89O13PK	[M+K]+
919.5667180	919.5672	0.0005	0.5437	PI O-37:1;O	C46H89O13PK	[M+K]+
921.6686213	921.6695	0.0009	0.9765	SQDG 41:0	C50H96O12S	[M+H]+
921.6686213	921.6691	0.0005	0.5425	EPC 50:6;O6	C52H95N2O10P	[M+H-H2O]+
921.6686213	921.6691	0.0005	0.5425	SM 47:6;O6	C52H95N2O10P	[M+H-H2O]+
921.6686213	921.6691	0.0005	0.5425	PC 44:7;O	C52H90NO9P	[M+NH4]+
921.6686213	921.6691	0.0005	0.5425	PE 47:7;O	C52H90NO9P	[M+NH4]+
921.6686213	921.6691	0.0005	0.5425	PS O-46:7	C52H90NO9P	[M+NH4]+
921.6705232	921.6695	0.0010	1.0850	SQDG 41:0	C50H96O12S	[M+H]+
921.6705232	921.6708	0.0002	0.2170	PA O-52:8	C55H95O7PNa	[M+Na]+
921.6705232	921.6709	0.0004	0.4340	PA 49:2	C52H99O8PK	[M+K]+
921.6705232	921.6709	0.0004	0.4340	PA O-49:3;O	C52H99O8PK	[M+K]+
923.7461617	923.7463	0.0002	0.2165	PA 52:3	C55H103O8P	[M+H]+
923.7461617	923.7463	0.0002	0.2165	PA O-52:4;O	C55H103O8P	[M+H]+
923.7461617	923.7463	0.0002	0.2165	PA 52:2;O	C55H105O9P	[M+H-H2O]+
923.7461617	923.7463	0.0002	0.2165	PG O-49:3	C55H105O9P	[M+H-H2O]+
923.7461617	923.7463	0.0001	0.1083	CE 33:5;O3	C60H100O5Na	[M+Na]+
923.7461617	923.7463	0.0001	0.1083	DG 57:9	C60H100O5Na	[M+Na]+
923.7461617	923.7463	0.0001	0.1083	DG O-57:10;O	C60H100O5Na	[M+Na]+
923.7461617	923.7463	0.0001	0.1083	TG O-57:9	C60H100O5Na	[M+Na]+
	923.7464	0.0003	0.3248	CE 30:0;O4	C57H104O6K	[M+K]+

923.7461617						
923.7461617	923.7464	0.0003	0.3248	DG 54:4;O	C57H104O6K	[M+K]+
	923.7464	0.0003	0.3248	DG O-54:5;O2	C57H104O6K	[M+K]+
923.7461617						
923.7461617	923.7464	0.0003	0.3248	TG 54:3	C57H104O6K	[M+K]+
	923.7464	0.0003	0.3248	TG O-54:4;O	C57H104O6K	[M+K]+
923.7461617						
924.7490712	924.7498	0.0007	0.7570	HexCer 48:3;O4	C54H101NO10	[M+H]+
	924.7498	0.0007	0.7570	HexCer 48:2;O5	C54H103NO11	[M+H-H2O]+
924.7490712						
924.7490712	924.7498	0.0007	0.7570	MGDG 45:3	C54H98O10	[M+NH4]+
	924.7498	0.0007	0.7570	HexCer 48:3;O4	C54H101NO10	[M+H]+
924.7505174						
924.7505174	924.7498	0.0007	0.7570	HexCer 48:2;O5	C54H103NO11	[M+H-H2O]+
	924.7498	0.0007	0.7570	MGDG 45:3	C54H98O10	[M+NH4]+
924.7505174						
925.7252582	925.7256	0.0003	0.3241	PA 51:3;O	C54H101O9P	[M+H]+
	925.7256	0.0003	0.3241	PG O-48:4	C54H101O9P	[M+H]+
925.7252582						
925.7252582	925.7256	0.0003	0.3241	PG 48:2	C54H103O10P	[M+H-H2O]+
	925.7256	0.0003	0.3241	PG O-48:3;O	C54H103O10P	[M+H-H2O]+
925.7252582						
925.7252582	925.7256	0.0003	0.3241	CE 32:5;O4	C59H98O6Na	[M+Na]+
	925.7256	0.0003	0.3241	DG 56:9;O	C59H98O6Na	[M+Na]+
925.7252582						
925.7252582	925.7256	0.0003	0.3241	DG O-56:10;O2	C59H98O6Na	[M+Na]+
	925.7256	0.0003	0.3241	TG 56:8	C59H98O6Na	[M+Na]+
925.7252582						
925.7252582	925.7256	0.0003	0.3241	TG O-56:9;O	C59H98O6Na	[M+Na]+
	925.7257	0.0004	0.4321	DG 53:4;O2	C56H102O7K	[M+K]+
925.7252582						
925.7252582	925.7257	0.0004	0.4321	TG 53:3;O	C56H102O7K	[M+K]+
	925.7257	0.0004	0.4321	TG O-53:4;O2	C56H102O7K	[M+K]+
925.7252582						
925.7619640	925.762	0.0000	0.0000	PA 52:2	C55H105O8P	[M+H]+
	925.762	0.0000	0.0000	PA O-52:3;O	C55H105O8P	[M+H]+
925.7619640						
925.7619640	925.762	0.0000	0.0000	PA 52:1;O	C55H107O9P	[M+H-H2O]+

	925.762	0.0000	0.0000	PG O-49:2	C55H107O9P	[M+H-H2O]+
925.7619640						
925.7619640	925.7619	0.0000	0.0000	CE 33:4;O3	C60H102O5Na	[M+Na]+
	925.7619	0.0000	0.0000	DG 57:8	C60H102O5Na	[M+Na]+
925.7619640						
925.7619640	925.7619	0.0000	0.0000	DG O-57:9;O	C60H102O5Na	[M+Na]+
	925.7619	0.0000	0.0000	TG O-57:8	C60H102O5Na	[M+Na]+
925.7619640						
925.7619640	925.7621	0.0001	0.1080	DG 54:3;O	C57H106O6K	[M+K]+
	925.7621	0.0001	0.1080	DG O-54:4;O2	C57H106O6K	[M+K]+
925.7619640						
925.7619640	925.7621	0.0001	0.1080	TG 54:2	C57H106O6K	[M+K]+
	925.7621	0.0001	0.1080	TG O-54:3;O	C57H106O6K	[M+K]+
925.7619640						
926.7653390	926.7655	0.0001	0.1079	HexCer 48:2;O4	C54H103NO10	[M+H]+
	926.7655	0.0001	0.1079	HexCer 48:1;O5	C54H105NO11	[M+H-H2O]+
926.7653390						
926.7653390	926.7655	0.0001	0.1079	MGDG 45:2	C54H100O10	[M+NH4]+
	927.7412	0.0016	1.7246	PA 51:2;O	C54H103O9P	[M+H]+
927.7395958						
927.7395958	927.7412	0.0016	1.7246	PG O-48:3	C54H103O9P	[M+H]+
	927.7412	0.0016	1.7246	PG 48:1	C54H105O10P	[M+H-H2O]+
927.7395958						
927.7395958	927.7412	0.0016	1.7246	PG O-48:2;O	C54H105O10P	[M+H-H2O]+
	927.7388	0.0008	0.8623	PG O-46:0	C52H105O9PNa	[M+Na]+
927.7395958						
927.7395958	927.7412	0.0016	1.7246	CE 32:4;O4	C59H100O6Na	[M+Na]+
	927.7412	0.0016	1.7246	DG 56:8;O	C59H100O6Na	[M+Na]+
927.7395958						
927.7395958	927.7412	0.0016	1.7246	DG O-56:9;O2	C59H100O6Na	[M+Na]+
	927.7412	0.0016	1.7246	TG 56:7	C59H100O6Na	[M+Na]+
927.7395958						
927.7395958	927.7412	0.0016	1.7246	TG O-56:8;O	C59H100O6Na	[M+Na]+
	927.7412	0.0001	0.1078	PA 51:2;O	C54H103O9P	[M+H]+
927.7411831						
927.7411831	927.7412	0.0001	0.1078	PG O-48:3	C54H103O9P	[M+H]+
	927.7412	0.0001	0.1078	PG 48:1	C54H105O10P	[M+H-H2O]+
927.7411831						
927.7411831	927.7412	0.0001	0.1078	PG O-48:2;O	C54H105O10P	[M+H-H2O]+

	927.7412	0.0000	0.0000	CE 32:4;O4	C59H100O6Na	[M+Na]+
927.7411831						
927.7411831	927.7412	0.0000	0.0000	DG 56:8;O	C59H100O6Na	[M+Na]+
	927.7412	0.0000	0.0000	DG O-56:9;O2	C59H100O6Na	[M+Na]+
927.7411831						
927.7411831	927.7412	0.0000	0.0000	TG 56:7	C59H100O6Na	[M+Na]+
	927.7412	0.0000	0.0000	TG O-56:8;O	C59H100O6Na	[M+Na]+
927.7411831						
927.7411831	927.7414	0.0002	0.2156	DG 53:3;O2	C56H104O7K	[M+K]+
	927.7414	0.0002	0.2156	TG 53:2;O	C56H104O7K	[M+K]+
927.7411831						
927.7411831	927.7414	0.0002	0.2156	TG O-53:3;O2	C56H104O7K	[M+K]+
	927.7776	0.0000	0.0000	PA 52:1	C55H107O8P	[M+H]+
927.7776460						
927.7776460	927.7776	0.0000	0.0000	PA O-52:2;O	C55H107O8P	[M+H]+
	927.7776	0.0000	0.0000	PA 52:0;O	C55H109O9P	[M+H-H2O]+
927.7776460						
927.7776460	927.7776	0.0000	0.0000	PG O-49:1	C55H109O9P	[M+H-H2O]+
	927.7776	0.0001	0.1078	CE 33:3;O3	C60H104O5Na	[M+Na]+
927.7776460						
927.7776460	927.7776	0.0001	0.1078	DG 57:7	C60H104O5Na	[M+Na]+
	927.7776	0.0001	0.1078	DG O-57:8;O	C60H104O5Na	[M+Na]+
927.7776460						
927.7776460	927.7776	0.0001	0.1078	TG O-57:7	C60H104O5Na	[M+Na]+
	927.7777	0.0001	0.1078	DG 54:2;O	C57H108O6K	[M+K]+
927.7776460						
927.7776460	927.7777	0.0001	0.1078	DG O-54:3;O2	C57H108O6K	[M+K]+
	927.7777	0.0001	0.1078	TG 54:1	C57H108O6K	[M+K]+
927.7776460						
927.7776460	927.7777	0.0001	0.1078	TG O-54:2;O	C57H108O6K	[M+K]+
	928.6062	0.0005	0.5384	PS 47:9	C53H86NO10P	[M+H]+
928.6057367						
928.6057367	928.6062	0.0005	0.5384	PS O-47:10;O	C53H86NO10P	[M+H]+
	928.6062	0.0005	0.5384	PS 47:8;O	C53H88NO11P	[M+H-H2O]+
928.6057367						
928.6057367	928.6062	0.0005	0.5384	PG 47:11	C53H83O10P	[M+NH4]+
	928.6062	0.0005	0.5384	PG O-47:12;O	C53H83O10P	[M+NH4]+

928.6057367						
928.7445069	928.7447	0.0002	0.2153	HexCer 47:2;O5	C53H101NO11	[M+H]+
	928.7447	0.0002	0.2153	HexCer 47:1;O6	C53H103NO12	[M+H-H2O]+
928.7445069						
929.6088526	929.6089	0.0001	0.1076	PI 39:1	C48H91O13PNa	[M+Na]+
	929.6089	0.0001	0.1076	PI O-39:2;O	C48H91O13PNa	[M+Na]+
929.6088526						
929.6108213	929.6114	0.0005	0.5379	PI 41:4	C50H89O13P	[M+H]+
	929.6114	0.0005	0.5379	PI O-41:5;O	C50H89O13P	[M+H]+
929.6108213						
929.6108213	929.6114	0.0005	0.5379	PI 41:3;O	C50H91O14P	[M+H-H2O]+
	929.6113	0.0005	0.5379	MGDG 46:10	C55H86O10Na	[M+Na]+
929.6108213						
929.7567981	929.7569	0.0001	0.1076	PA 51:1;O	C54H105O9P	[M+H]+
	929.7569	0.0001	0.1076	PG O-48:2	C54H105O9P	[M+H]+
929.7567981						
929.7567981	929.7569	0.0001	0.1076	PG 48:0	C54H107O10P	[M+H-H2O]+
	929.7569	0.0001	0.1076	PG O-48:1;O	C54H107O10P	[M+H-H2O]+
929.7567981						
929.7567981	929.7569	0.0001	0.1076	CE 32:3;O4	C59H102O6Na	[M+Na]+
	929.7569	0.0001	0.1076	DG 56:7;O	C59H102O6Na	[M+Na]+
929.7567981						
929.7567981	929.7569	0.0001	0.1076	DG O-56:8;O2	C59H102O6Na	[M+Na]+
	929.7569	0.0001	0.1076	TG 56:6	C59H102O6Na	[M+Na]+
929.7567981						
929.7567981	929.7569	0.0001	0.1076	TG O-56:7;O	C59H102O6Na	[M+Na]+
	929.757	0.0002	0.2151	DG 53:2;O2	C56H106O7K	[M+K]+
929.7567981						
929.7567981	929.757	0.0002	0.2151	TG 53:1;O	C56H106O7K	[M+K]+
	929.757	0.0002	0.2151	TG O-53:2;O2	C56H106O7K	[M+K]+
929.7567981						
930.4033029	930.4013	0.0020	2.1496	MIPC 30:6;O3	C42H70NO17PK	[M+K]+
	930.5785	0.0006	0.6448	Hex2Cer 36:5;O5	C48H83NO16	[M+H]+
930.5790256						
930.5790256	930.5785	0.0006	0.6448	Hex2Cer 36:4;O6	C48H85NO17	[M+H-H2O]+
	930.5785	0.0024	2.5790	Hex2Cer 36:5;O5	C48H83NO16	[M+H]+
930.5809087						
930.5809087	930.5785	0.0024	2.5790	Hex2Cer 36:4;O6	C48H85NO17	[M+H-H2O]+

	930.5831	0.0022	2.3641	PS 44:6;O	C50H86NO11PNa	[M+Na]+
930.5809087						
930.5809087	930.5832	0.0023	2.4716	IPC 41:2;O3	C47H90NO12PK	[M+K]+
	930.6195	0.0000	0.0000	PS 45:5	C51H90NO10PNa	[M+Na]+
930.6194828						
930.6194828	930.6195	0.0000	0.0000	PS O-45:6;O	C51H90NO10PNa	[M+Na]+
	930.6196	0.0001	0.1075	IPC 42:1;O2	C48H94NO11PK	[M+K]+
930.6194828						
930.6194828	930.6196	0.0001	0.1075	PS 42:0;O	C48H94NO11PK	[M+K]+
	930.6219	0.0003	0.3224	PS 47:8	C53H88NO10P	[M+H]+
930.6215587						
930.6215587	930.6219	0.0003	0.3224	PS O-47:9;O	C53H88NO10P	[M+H]+
	930.6219	0.0003	0.3224	PS 47:7;O	C53H90NO11P	[M+H-H2O]+
930.6215587						
930.6215587	930.6219	0.0003	0.3224	PG 47:10	C53H85O10P	[M+NH4]+
	930.6219	0.0003	0.3224	PG O-47:11;O	C53H85O10P	[M+NH4]+
930.6215587						
930.7593752	930.7604	0.0010	1.0744	HexCer 47:1;O5	C53H103NO11	[M+H]+
	930.7604	0.0010	1.0744	HexCer 47:0;O6	C53H105NO12	[M+H-H2O]+
930.7593752						
930.7607181	930.7604	0.0003	0.3223	HexCer 47:1;O5	C53H103NO11	[M+H]+
	930.7604	0.0003	0.3223	HexCer 47:0;O6	C53H105NO12	[M+H-H2O]+
930.7607181						
931.5843985	931.5849	0.0005	0.5367	TG 55:14;O	C58H84O7K	[M+K]+
	931.5849	0.0005	0.5367	TG O-55:15;O2	C58H84O7K	[M+K]+
931.5843985						
931.6250391	931.6246	0.0004	0.4294	PI 39:0	C48H93O13PNa	[M+Na]+
	931.6246	0.0004	0.4294	PI O-39:1;O	C48H93O13PNa	[M+Na]+
931.6250391						
931.7725041	931.7725	0.0000	0.0000	PA 51:0;O	C54H107O9P	[M+H]+
	931.7725	0.0000	0.0000	PG O-48:1	C54H107O9P	[M+H]+
931.7725041						
931.7725041	931.7725	0.0000	0.0000	PG O-48:0;O	C54H109O10P	[M+H-H2O]+
	931.7725	0.0000	0.0000	CE 32:2;O4	C59H104O6Na	[M+Na]+
931.7725041						
931.7725041	931.7725	0.0000	0.0000	DG 56:6;O	C59H104O6Na	[M+Na]+
	931.7725	0.0000	0.0000	DG O-56:7;O2	C59H104O6Na	[M+Na]+
931.7725041						
931.7725041	931.7725	0.0000	0.0000	TG 56:5	C59H104O6Na	[M+Na]+

931.7725041	931.7725	0.0000	0.0000	TG O-56:6;O	C59H104O6Na	[M+Na]+
931.7725041	931.7727	0.0002	0.2146	DG 53:1;O2	C56H108O7K	[M+K]+
931.7725041	931.7727	0.0002	0.2146	TG 53:0;O	C56H108O7K	[M+K]+
931.7725041	931.7727	0.0002	0.2146	TG O-53:1;O2	C56H108O7K	[M+K]+
932.6367643	932.6375	0.0007	0.7506	PS 47:7	C53H90NO10P	[M+H]+
932.6367643	932.6375	0.0007	0.7506	PS O-47:8;O	C53H90NO10P	[M+H]+
932.6367643	932.6375	0.0007	0.7506	PS 47:6;O	C53H92NO11P	[M+H-H2O]+
932.6367643	932.6375	0.0007	0.7506	PG 47:9	C53H87O10P	[M+NH4]+
932.6367643	932.6375	0.0007	0.7506	PG O-47:10;O	C53H87O10P	[M+NH4]+
932.6367643	932.6353	0.0015	1.6083	IPC 42:0;O2	C48H96NO11PK	[M+K]+
932.7758845	932.776	0.0001	0.1072	HexCer 47:0;O5	C53H105NO11	[M+H]+
933.6392369	933.6402	0.0010	1.0711	PI O-39:0;O	C48H95O13PNa	[M+Na]+
933.6392369	933.6386	0.0006	0.6426	IPC 39:0;O6	C45H90NO15P	[M+NH4]+
933.6411075	933.6427	0.0015	1.6066	PI 41:2	C50H93O13P	[M+H]+
933.6411075	933.6427	0.0015	1.6066	PI O-41:3;O	C50H93O13P	[M+H]+
933.6411075	933.6427	0.0015	1.6066	PI 41:1;O	C50H95O14P	[M+H-H2O]+
933.6411075	933.6402	0.0009	0.9640	PI O-39:0;O	C48H95O13PNa	[M+Na]+
933.6411075	933.6426	0.0015	1.6066	MGDG 46:8	C55H90O10Na	[M+Na]+
933.7881776	933.7882	0.0000	0.0000	PG O-48:0	C54H109O9P	[M+H]+
933.7881776	933.7882	0.0000	0.0000	CE 32:1;O4	C59H106O6Na	[M+Na]+
933.7881776	933.7882	0.0000	0.0000	DG 56:5;O	C59H106O6Na	[M+Na]+
933.7881776	933.7882	0.0000	0.0000	DG O-56:6;O2	C59H106O6Na	[M+Na]+
933.7881776	933.7882	0.0000	0.0000	TG 56:4	C59H106O6Na	[M+Na]+
933.7881776	933.7882	0.0000	0.0000	TG O-56:5;O	C59H106O6Na	[M+Na]+
	933.7883	0.0001	0.1071	DG 53:0;O2	C56H110O7K	[M+K]+

933.7881776						
933.7881776	933.7883	0.0001	0.1071	TG O-53:0;O2	C56H110O7K	[M+K]+
	934.6532	0.0000	0.0000	PS 47:6	C53H92NO10P	[M+H]+
934.6531352						
934.6531352	934.6532	0.0000	0.0000	PS O-47:7;O	C53H92NO10P	[M+H]+
	934.6532	0.0000	0.0000	IPC 47:6;O2	C53H94NO11P	[M+H-H2O]+
934.6531352						
934.6531352	934.6532	0.0000	0.0000	PS 47:5;O	C53H94NO11P	[M+H-H2O]+
	934.6532	0.0000	0.0000	PG 47:8	C53H89O10P	[M+NH4]+
934.6531352						
934.6531352	934.6532	0.0000	0.0000	PG O-47:9;O	C53H89O10P	[M+NH4]+
	934.7947	0.0031	3.3162	EPC 50:1;O4	C52H105N2O8P	[M+NH4]+
934.7915814						
934.7915814	934.7947	0.0031	3.3162	SM 47:1;O4	C52H105N2O8P	[M+NH4]+
	935.6583	0.0009	0.9619	PI 41:1	C50H95O13P	[M+H]+
935.6573542						
935.6573542	935.6583	0.0009	0.9619	PI O-41:2;O	C50H95O13P	[M+H]+
	935.6583	0.0009	0.9619	PI 41:0;O	C50H97O14P	[M+H-H2O]+
935.6573542						
935.6573542	935.6583	0.0009	0.9619	MGDG 46:7	C55H92O10Na	[M+Na]+
	936.6688	0.0014	1.4947	PS 47:5	C53H94NO10P	[M+H]+
936.6673770						
936.6673770	936.6688	0.0014	1.4947	PS O-47:6;O	C53H94NO10P	[M+H]+
	936.6688	0.0014	1.4947	IPC 47:5;O2	C53H96NO11P	[M+H-H2O]+
936.6673770						
936.6673770	936.6688	0.0014	1.4947	PS 47:4;O	C53H96NO11P	[M+H-H2O]+
	936.6664	0.0010	1.0676	CerP 51:4;O6	C51H96NO10PNa	[M+Na]+
936.6673770						
936.6673770	936.6664	0.0010	1.0676	PS 45:2	C51H96NO10PNa	[M+Na]+
	936.6664	0.0010	1.0676	PS O-45:3;O	C51H96NO10PNa	[M+Na]+
936.6673770						
936.6673770	936.6688	0.0014	1.4947	PG 47:7	C53H91O10P	[M+NH4]+
	936.6688	0.0014	1.4947	PG O-47:8;O	C53H91O10P	[M+NH4]+
936.6673770						
937.6993640	937.7004	0.0011	1.1731	EPC 51:6;O5	C53H97N2O9P	[M+H]+
	937.7004	0.0011	1.1731	SM 48:6;O5	C53H97N2O9P	[M+H]+
937.6993640						
937.6993640	937.7004	0.0011	1.1731	EPC 51:5;O6	C53H99N2O10P	[M+H-H2O]+

	937.7004	0.0011	1.1731	SM 48:5;O6	C53H99N2O10P	[M+H-H2O]+
937.6993640						
937.6993640	937.698	0.0013	1.3864	EPC 49:3;O5	C51H99N2O9PNa	[M+Na]+
	937.698	0.0013	1.3864	SM 46:3;O5	C51H99N2O9PNa	[M+Na]+
937.6993640						
937.6993640	937.7004	0.0011	1.1731	PC 45:6;O	C53H94NO9P	[M+NH4]+
	937.7004	0.0011	1.1731	PE 48:6;O	C53H94NO9P	[M+NH4]+
937.6993640						
937.6993640	937.7004	0.0011	1.1731	PS O-47:6	C53H94NO9P	[M+NH4]+
	937.7004	0.0003	0.3199	EPC 51:6;O5	C53H97N2O9P	[M+H]+
937.7007635						
937.7007635	937.7004	0.0003	0.3199	SM 48:6;O5	C53H97N2O9P	[M+H]+
	937.7004	0.0003	0.3199	EPC 51:5;O6	C53H99N2O10P	[M+H-H2O]+
937.7007635						
937.7007635	937.7004	0.0003	0.3199	SM 48:5;O6	C53H99N2O10P	[M+H-H2O]+
	937.7004	0.0003	0.3199	PC 45:6;O	C53H94NO9P	[M+NH4]+
937.7007635						
937.7007635	937.7004	0.0003	0.3199	PE 48:6;O	C53H94NO9P	[M+NH4]+
	937.7004	0.0003	0.3199	PS O-47:6	C53H94NO9P	[M+NH4]+
937.7007635						
937.8190182	937.8195	0.0004	0.4265	DG 56:3;O	C59H110O6Na	[M+Na]+
	937.8195	0.0004	0.4265	DG O-56:4;O2	C59H110O6Na	[M+Na]+
937.8190182						
937.8190182	937.8195	0.0004	0.4265	TG 56:2	C59H110O6Na	[M+Na]+
	937.8195	0.0004	0.4265	TG O-56:3;O	C59H110O6Na	[M+Na]+
937.8190182						
937.8190182	937.8178	0.0012	1.2796	ACer 56:3;O6	C56H105NO8	[M+NH4]+
	937.8178	0.0012	1.2796	HexCer 50:3;O2	C56H105NO8	[M+NH4]+
937.8190182						
937.8204722	937.8219	0.0014	1.4928	CE 34:2;O4	C61H108O6	[M+H]+
	937.8219	0.0014	1.4928	DG 58:6;O	C61H108O6	[M+H]+
937.8204722						
937.8204722	937.8219	0.0014	1.4928	DG O-58:7;O2	C61H108O6	[M+H]+
	937.8219	0.0014	1.4928	TG 58:5	C61H108O6	[M+H]+
937.8204722						
937.8204722	937.8219	0.0014	1.4928	TG O-58:6;O	C61H108O6	[M+H]+
	937.8219	0.0014	1.4928	DG 58:5;O2	C61H110O7	[M+H-H2O]+
937.8204722						
937.8204722	937.8219	0.0014	1.4928	TG 58:4;O	C61H110O7	[M+H-H2O]+

	937.8219	0.0014	1.4928	TG O-58:5;O2	C61H110O7	[M+H-H2O]+
937.8204722						
937.8204722	937.8195	0.0010	1.0663	DG 56:3;O	C59H110O6Na	[M+Na]+
	937.8195	0.0010	1.0663	DG O-56:4;O2	C59H110O6Na	[M+Na]+
937.8204722						
937.8204722	937.8195	0.0010	1.0663	TG 56:2	C59H110O6Na	[M+Na]+
	937.8195	0.0010	1.0663	TG O-56:3;O	C59H110O6Na	[M+Na]+
937.8204722						
938.5148810	938.5155	0.0007	0.7459	IPC 41:6;O4	C47H82NO13PK	[M+K]+
	938.6845	0.0001	0.1065	CerP 53:6;O6	C53H96NO10P	[M+H]+
938.6843180						
938.6843180	938.6845	0.0001	0.1065	PS 47:4	C53H96NO10P	[M+H]+
	938.6845	0.0001	0.1065	PS O-47:5;O	C53H96NO10P	[M+H]+
938.6843180						
938.6843180	938.6845	0.0001	0.1065	IPC 47:4;O2	C53H98NO11P	[M+H-H2O]+
	938.6845	0.0001	0.1065	PS 47:3;O	C53H98NO11P	[M+H-H2O]+
938.6843180						
938.6843180	938.6845	0.0001	0.1065	PG 47:6	C53H93O10P	[M+NH4]+
	938.6845	0.0001	0.1065	PG O-47:7;O	C53H93O10P	[M+NH4]+
938.6843180						
938.6843180	938.6846	0.0003	0.3196	ACer 55:6;O6	C55H97NO8K	[M+K]+
	938.6846	0.0003	0.3196	HexCer 49:6;O2	C55H97NO8K	[M+K]+
938.6843180						
938.7036021	938.7056	0.0020	2.1306	IPC 44:0;O3	C50H100NO12P	[M+H]+
	938.7055	0.0019	2.0241	HexCer 49:6;O3	C55H97NO9Na	[M+Na]+
938.7036021						
938.7036021	938.7056	0.0020	2.1306	PI O-41:1	C50H97O12P	[M+NH4]+
	938.7057	0.0021	2.2371	HexCer 46:1;O4	C52H101NO10K	[M+K]+
938.7036021						
939.5180857	939.5189	0.0008	0.8515	MIPC 32:5;O3	C44H76NO17P	[M+NH4]+
	939.5189	0.0018	1.9159	MIPC 32:5;O3	C44H76NO17P	[M+NH4]+
939.5206918						
939.6877989	939.6879	0.0001	0.1064	HexCer 46:6;O6	C52H91NO12	[M+NH4]+
	941.5692	0.0010	1.0621	TG 56:16;O	C59H82O7K	[M+K]+
941.5681756						
941.5681756	941.5668	0.0013	1.3807	PG 46:8	C52H87O10PK	[M+K]+
	941.5668	0.0013	1.3807	PG O-46:9;O	C52H87O10PK	[M+K]+

941.5681756						
941.5723587	941.5726	0.0002	0.2124	PI 39:3;O	C48H87O14PNa	[M+Na]+
	941.8144	0.0000	0.0000	DG 55:2;O2	C58H110O7Na	[M+Na]+
941.8143594						
941.8143594	941.8144	0.0000	0.0000	TG 55:1;O	C58H110O7Na	[M+Na]+
	941.8144	0.0000	0.0000	TG O-55:2;O2	C58H110O7Na	[M+Na]+
941.8143594						
942.5794876	942.5785	0.0010	1.0609	Hex2Cer 37:6;O5	C49H83NO16	[M+H]+
	942.5785	0.0010	1.0609	Hex2Cer 37:5;O6	C49H85NO17	[M+H-H2O]+
942.5794876						
942.5811963	942.5785	0.0027	2.8645	Hex2Cer 37:6;O5	C49H83NO16	[M+H]+
	942.5785	0.0027	2.8645	Hex2Cer 37:5;O6	C49H85NO17	[M+H-H2O]+
942.5811963						
942.5811963	942.5831	0.0019	2.0157	PS 45:7;O	C51H86NO11PNa	[M+Na]+
	942.5832	0.0020	2.1218	IPC 42:3;O3	C48H90NO12PK	[M+K]+
942.5811963						
943.7152899	943.715	0.0003	0.3179	PA 54:7	C57H99O8P	[M+H]+
	943.715	0.0003	0.3179	PA O-54:8;O	C57H99O8P	[M+H]+
943.7152899						
943.7152899	943.715	0.0003	0.3179	PA 54:6;O	C57H101O9P	[M+H-H2O]+
	943.715	0.0003	0.3179	PG O-51:7	C57H101O9P	[M+H-H2O]+
943.7152899						
943.7152899	943.715	0.0003	0.3179	DG 59:13	C62H96O5Na	[M+Na]+
	943.715	0.0003	0.3179	DG O-59:14;O	C62H96O5Na	[M+Na]+
943.7152899						
943.7152899	943.715	0.0003	0.3179	TG O-59:13	C62H96O5Na	[M+Na]+
	943.7151	0.0001	0.1060	CE 32:4;O4	C59H100O6K	[M+K]+
943.7152899						
943.7152899	943.7151	0.0001	0.1060	DG 56:8;O	C59H100O6K	[M+K]+
	943.7151	0.0001	0.1060	DG O-56:9;O2	C59H100O6K	[M+K]+
943.7152899						
943.7152899	943.7151	0.0001	0.1060	TG 56:7	C59H100O6K	[M+K]+
	943.7151	0.0001	0.1060	TG O-56:8;O	C59H100O6K	[M+K]+
943.7152899						
943.8290005	943.83	0.0010	1.0595	DG 55:1;O2	C58H112O7Na	[M+Na]+
	943.83	0.0010	1.0595	TG 55:0;O	C58H112O7Na	[M+Na]+
943.8290005						
943.8290005	943.83	0.0010	1.0595	TG O-55:1;O2	C58H112O7Na	[M+Na]+

	943.8284	0.0006	0.6357	HexCer 49:1;O3	C55H107NO9	[M+NH4]+
943.8290005						
943.8308261	943.8324	0.0016	1.6952	DG 57:4;O2	C60H110O7	[M+H]+
	943.8324	0.0016	1.6952	TG 57:3;O	C60H110O7	[M+H]+
943.8308261						
943.8308261	943.8324	0.0016	1.6952	TG O-57:4;O2	C60H110O7	[M+H]+
	943.8324	0.0016	1.6952	TG 57:2;O2	C60H112O8	[M+H-H2O]+
943.8308261						
943.8308261	943.8324	0.0016	1.6952	TG O-57:3;O3	C60H112O8	[M+H-H2O]+
	943.83	0.0008	0.8476	DG 55:1;O2	C58H112O7Na	[M+Na]+
943.8308261						
943.8308261	943.83	0.0008	0.8476	TG 55:0;O	C58H112O7Na	[M+Na]+
	943.83	0.0008	0.8476	TG O-55:1;O2	C58H112O7Na	[M+Na]+
943.8308261						
947.6848088	947.6852	0.0004	0.4221	SQDG 43:1	C52H98O12S	[M+H]+
	947.6848	0.0000	0.0000	PC 46:8;O	C54H92NO9P	[M+NH4]+
947.6848088						
947.6848088	947.6848	0.0000	0.0000	PE 49:8;O	C54H92NO9P	[M+NH4]+
	947.6848	0.0000	0.0000	PS O-48:8	C54H92NO9P	[M+NH4]+
947.6848088						
948.6288674	948.63	0.0011	1.1596	IPC 45:5;O2	C51H92NO11PNa	[M+Na]+
	948.63	0.0011	1.1596	PS 45:4;O	C51H92NO11PNa	[M+Na]+
948.6288674						
948.6288674	948.6302	0.0013	1.3704	IPC 42:0;O3	C48H96NO12PK	[M+K]+
	948.6324	0.0009	0.9487	PS 47:7;O	C53H90NO11P	[M+H]+
948.6315019						
948.6315019	948.6324	0.0009	0.9487	PG 47:9;O	C53H87O11P	[M+NH4]+
	948.6302	0.0013	1.3704	IPC 42:0;O3	C48H96NO12PK	[M+K]+
948.6315019						
949.6361167	949.6376	0.0015	1.5795	PI 41:2;O	C50H93O14P	[M+H]+
	950.7056	0.0016	1.6830	IPC 45:1;O3	C51H100NO12P	[M+H]+
950.7039922						
950.7039922	950.7056	0.0016	1.6830	IPC 45:0;O4	C51H102NO13P	[M+H-H2O]+
	950.7056	0.0016	1.6830	PI O-42:2	C51H97O12P	[M+NH4]+
950.7039922						
950.7039922	950.7057	0.0017	1.7881	HexCer 47:2;O4	C53H101NO10K	[M+K]+
	953.7229	0.0005	0.5243	DG 59:13;O2	C62H96O7	[M+H]+
953.7224179						
953.7224179	953.7229	0.0005	0.5243	TG 59:12;O	C62H96O7	[M+H]+

	953.7229	0.0005	0.5243	TG O-59:13;O2	C62H96O7	[M+H]+
953.7224179						
953.7224179	953.7229	0.0005	0.5243	TG 59:11;O2	C62H98O8	[M+H-H2O]+
	953.7229	0.0005	0.5243	TG O-59:12;O3	C62H98O8	[M+H-H2O]+
953.7224179						
953.7325374	953.7317	0.0008	0.8388	EPC 52:5;O5	C54H101N2O9P	[M+H]+
	953.7317	0.0008	0.8388	SM 49:5;O5	C54H101N2O9P	[M+H]+
953.7325374						
953.7325374	953.7317	0.0008	0.8388	EPC 52:4;O6	C54H103N2O10P	[M+H-H2O]+
	953.7317	0.0008	0.8388	SM 49:4;O6	C54H103N2O10P	[M+H-H2O]+
953.7325374						
953.7325374	953.7334	0.0008	0.8388	PA O-54:6	C57H103O7PNa	[M+Na]+
	953.7317	0.0008	0.8388	CerP 54:6;O5	C54H98NO9P	[M+NH4]+
953.7325374						
953.7325374	953.7317	0.0008	0.8388	PC 46:5;O	C54H98NO9P	[M+NH4]+
	953.7317	0.0008	0.8388	PE 49:5;O	C54H98NO9P	[M+NH4]+
953.7325374						
953.7325374	953.7317	0.0008	0.8388	PS O-48:5	C54H98NO9P	[M+NH4]+
	953.7335	0.0010	1.0485	PA 51:0	C54H107O8PK	[M+K]+
953.7325374						
953.7325374	953.7335	0.0010	1.0485	PA O-51:1;O	C54H107O8PK	[M+K]+
	953.7569	0.0000	0.0000	PA 53:3;O	C56H105O9P	[M+H]+
953.7568897						
953.7568897	953.7569	0.0000	0.0000	PG O-50:4	C56H105O9P	[M+H]+
	953.7569	0.0000	0.0000	PG 50:2	C56H107O10P	[M+H-H2O]+
953.7568897						
953.7568897	953.7569	0.0000	0.0000	PG O-50:3;O	C56H107O10P	[M+H-H2O]+
	953.7569	0.0000	0.0000	CE 34:5;O4	C61H102O6Na	[M+Na]+
953.7568897						
953.7568897	953.7569	0.0000	0.0000	DG 58:9;O	C61H102O6Na	[M+Na]+
	953.7569	0.0000	0.0000	DG O-58:10;O2	C61H102O6Na	[M+Na]+
953.7568897						
953.7568897	953.7569	0.0000	0.0000	TG 58:8	C61H102O6Na	[M+Na]+
	953.7569	0.0000	0.0000	TG O-58:9;O	C61H102O6Na	[M+Na]+
953.7568897						
953.7568897	953.757	0.0001	0.1048	DG 55:4;O2	C58H106O7K	[M+K]+
	953.757	0.0001	0.1048	TG 55:3;O	C58H106O7K	[M+K]+

953.7568897						
953.7568897	953.757	0.0001	0.1048	TG O-55:4;O2	C58H106O7K	[M+K]+
	954.643	0.0000	0.0000	IPC 46:6;O3	C52H92NO12P	[M+H]+
954.6430309						
954.6430309	954.643	0.0000	0.0000	IPC 46:5;O4	C52H94NO13P	[M+H-H2O]+
	954.643	0.0000	0.0000	PI O-43:7	C52H89O12P	[M+NH4]+
954.6430309						
954.7354111	954.7368	0.0014	1.4664	HexCer 50:5;O3	C56H101NO9Na	[M+Na]+
	954.7369	0.0015	1.5711	PI O-42:0	C51H101O12P	[M+NH4]+
954.7354111						
954.7354111	954.737	0.0016	1.6759	HexCer 47:0;O4	C53H105NO10K	[M+K]+
	954.7604	0.0005	0.5237	HexCer 49:3;O5	C55H103NO11	[M+H]+
954.7608491						
954.7608491	954.7604	0.0005	0.5237	HexCer 49:2;O6	C55H105NO12	[M+H-H2O]+
	955.7725	0.0001	0.1046	PA 53:2;O	C56H107O9P	[M+H]+
955.7726199						
955.7726199	955.7725	0.0001	0.1046	PG O-50:3	C56H107O9P	[M+H]+
	955.7725	0.0001	0.1046	PG 50:1	C56H109O10P	[M+H-H2O]+
955.7726199						
955.7726199	955.7725	0.0001	0.1046	PG O-50:2;O	C56H109O10P	[M+H-H2O]+
	955.7725	0.0001	0.1046	CE 34:4;O4	C61H104O6Na	[M+Na]+
955.7726199						
955.7726199	955.7725	0.0001	0.1046	DG 58:8;O	C61H104O6Na	[M+Na]+
	955.7725	0.0001	0.1046	DG O-58:9;O2	C61H104O6Na	[M+Na]+
955.7726199						
955.7726199	955.7725	0.0001	0.1046	TG 58:7	C61H104O6Na	[M+Na]+
	955.7725	0.0001	0.1046	TG O-58:8;O	C61H104O6Na	[M+Na]+
955.7726199						
955.7726199	955.7727	0.0000	0.0000	DG 55:3;O2	C58H108O7K	[M+K]+
	955.7727	0.0000	0.0000	TG 55:2;O	C58H108O7K	[M+K]+
955.7726199						
955.7726199	955.7727	0.0000	0.0000	TG O-55:3;O2	C58H108O7K	[M+K]+
	956.6375	0.0003	0.3136	PS 49:9	C55H90NO10P	[M+H]+
956.6372179						
956.6372179	956.6375	0.0003	0.3136	PS O-49:10;O	C55H90NO10P	[M+H]+
	956.6375	0.0003	0.3136	PS 49:8;O	C55H92NO11P	[M+H-H2O]+
956.6372179						
956.6372179	956.6375	0.0003	0.3136	PG 49:11	C55H87O10P	[M+NH4]+

	956.6375	0.0003	0.3136	PG O-49:12;O	C55H87O10P	[M+NH4]+
956.6372179						
956.6586088	956.6586	0.0000	0.0000	IPC 46:5;O3	C52H94NO12P	[M+H]+
	956.6586	0.0000	0.0000	IPC 46:4;O4	C52H96NO13P	[M+H-H2O]+
956.6586088						
956.6586088	956.6586	0.0000	0.0000	PI O-43:6	C52H91O12P	[M+NH4]+
	956.6588	0.0001	0.1045	HexCer 48:6;O4	C54H95NO10K	[M+K]+
956.6586088						
956.6603626	956.661	0.0006	0.6272	TG 56:14;O3	C59H86O9	[M+NH4]+
	956.776	0.0001	0.1045	HexCer 49:2;O5	C55H105NO11	[M+H]+
956.7761007						
956.7761007	956.776	0.0001	0.1045	HexCer 49:1;O6	C55H107NO12	[M+H-H2O]+
	957.6427	0.0010	1.0442	PI 43:4	C52H93O13P	[M+H]+
957.6416269						
957.6416269	957.6427	0.0010	1.0442	PI O-43:5;O	C52H93O13P	[M+H]+
	957.6427	0.0010	1.0442	PI 43:3;O	C52H95O14P	[M+H-H2O]+
957.6416269						
957.6416269	957.6426	0.0010	1.0442	MGDG 48:10	C57H90O10Na	[M+Na]+
	957.6402	0.0014	1.4619	PI 41:1	C50H95O13PNa	[M+Na]+
957.6416269						
957.6416269	957.6402	0.0014	1.4619	PI O-41:2;O	C50H95O13PNa	[M+Na]+
	957.6621	0.0001	0.1044	Hex2Cer 39:5;O3	C51H89NO14	[M+NH4]+
957.6622551						
957.7882781	957.7882	0.0001	0.1044	PA 53:1;O	C56H109O9P	[M+H]+
	957.7882	0.0001	0.1044	PG O-50:2	C56H109O9P	[M+H]+
957.7882781						
957.7882781	957.7882	0.0001	0.1044	PG 50:0	C56H111O10P	[M+H-H2O]+
	957.7882	0.0001	0.1044	PG O-50:1;O	C56H111O10P	[M+H-H2O]+
957.7882781						
957.7882781	957.7882	0.0001	0.1044	CE 34:3;O4	C61H106O6Na	[M+Na]+
	957.7882	0.0001	0.1044	DG 58:7;O	C61H106O6Na	[M+Na]+
957.7882781						
957.7882781	957.7882	0.0001	0.1044	DG O-58:8;O2	C61H106O6Na	[M+Na]+
	957.7882	0.0001	0.1044	TG 58:6	C61H106O6Na	[M+Na]+
957.7882781						
957.7882781	957.7882	0.0001	0.1044	TG O-58:7;O	C61H106O6Na	[M+Na]+
	957.7883	0.0000	0.0000	DG 55:2;O2	C58H110O7K	[M+K]+
957.7882781						
957.7882781	957.7883	0.0000	0.0000	TG 55:1;O	C58H110O7K	[M+K]+

957.7882781	957.7883	0.0000	0.0000	TG O-55:2;O2	C58H110O7K	[M+K]+
958.6742330	958.6743	0.0001	0.1043	IPC 46:4;O3	C52H96NO12P	[M+H]+
958.6742330	958.6743	0.0001	0.1043	IPC 46:3;O4	C52H98NO13P	[M+H-H2O]+
958.6742330	958.6743	0.0001	0.1043	PI O-43:5	C52H93O12P	[M+NH4]+
958.6742330	958.6744	0.0002	0.2086	HexCer 48:5;O4	C54H97NO10K	[M+K]+
958.7918107	958.7917	0.0001	0.1043	HexCer 49:1;O5	C55H107NO11	[M+H]+
958.7918107	958.7917	0.0001	0.1043	HexCer 49:0;O6	C55H109NO12	[M+H-H2O]+
959.6778395	959.6759	0.0019	1.9798	TG 60:16;O	C63H90O7	[M+H]+
959.6778395	959.6778	0.0001	0.1042	Hex2Cer 39:4;O3	C51H91NO14	[M+NH4]+
964.5652432	964.5674	0.0022	2.2808	PS 47:10;O	C53H84NO11PNa	[M+Na]+
964.5652432	964.5676	0.0023	2.3845	IPC 44:6;O3	C50H88NO12PK	[M+K]+
965.5720422	965.5726	0.0005	0.5178	PI 41:5;O	C50H87O14PNa	[M+Na]+
965.5720422	965.5709	0.0011	1.1392	MIPC 35:5;O2	C47H82NO16P	[M+NH4]+
965.8494426	965.8491	0.0003	0.3106	ACer 58:3;O6	C58H109NO8	[M+NH4]+
965.8494426	965.8491	0.0003	0.3106	HexCer 52:3;O2	C58H109NO8	[M+NH4]+
965.8510802	965.8508	0.0003	0.3106	DG 58:3;O	C61H114O6Na	[M+Na]+
965.8510802	965.8508	0.0003	0.3106	DG O-58:4;O2	C61H114O6Na	[M+Na]+
965.8510802	965.8508	0.0003	0.3106	TG 58:2	C61H114O6Na	[M+Na]+
965.8510802	965.8508	0.0003	0.3106	TG O-58:3;O	C61H114O6Na	[M+Na]+
966.5790794	966.5761	0.0030	3.1037	Hex2Cer 37:5;O5	C49H85NO16Na	[M+Na]+
966.5790794	966.5762	0.0029	3.0003	Hex2Cer 34:0;O6	C46H89NO17K	[M+K]+
966.5813598	966.5831	0.0017	1.7588	PS 47:9;O	C53H86NO11PNa	[M+Na]+
966.5813598	966.5832	0.0019	1.9657	IPC 44:5;O3	C50H90NO12PK	[M+K]+
967.5850982	967.5866	0.0015	1.5502	MIPC 35:4;O2	C47H84NO16P	[M+NH4]+
	968.5987	0.0014	1.4454	PS 47:8;O	C53H88NO11PNa	[M+Na]+

968.5973577						
968.5973577	968.5989	0.0015	1.5486	IPC 44:4;O3	C50H92NO12PK	[M+K]+
	968.6011	0.0000	0.0000	PS 49:11;O	C55H86NO11P	[M+H]+
968.6011431						
968.6789743	968.6798	0.0008	0.8259	IPC 44:1;O5	C50H98NO14P	[M+H]+
	968.6798	0.0008	0.8259	IPC 44:0;O6	C50H100NO15P	[M+H-H2O]+
968.6789743						
968.6789743	968.6798	0.0008	0.8259	PI 41:1;O	C50H95O14P	[M+NH4]+
	968.6799	0.0009	0.9291	HexCer 46:2;O6	C52H99NO12K	[M+K]+
968.6789743						
968.6808205	968.6798	0.0011	1.1356	IPC 44:1;O5	C50H98NO14P	[M+H]+
	968.6798	0.0011	1.1356	IPC 44:0;O6	C50H100NO15P	[M+H-H2O]+
968.6808205						
968.6808205	968.6798	0.0011	1.1356	PI 41:1;O	C50H95O14P	[M+NH4]+
	968.6799	0.0009	0.9291	HexCer 46:2;O6	C52H99NO12K	[M+K]+
968.6808205						
968.6994384	968.7009	0.0014	1.4452	Hex2Cer 40:1;O2	C52H99NO13Na	[M+Na]+
	968.6974	0.0020	2.0646	TG 58:14;O2	C61H90O8	[M+NH4]+
968.6994384						
968.6994384	968.6974	0.0020	2.0646	TG O-58:15;O3	C61H90O8	[M+NH4]+
	968.7009	0.0003	0.3097	Hex2Cer 40:1;O2	C52H99NO13Na	[M+Na]+
968.7011497						
969.6032284	969.6039	0.0006	0.6188	PI 41:3;O	C50H91O14PNa	[M+Na]+
	969.6022	0.0010	1.0314	MIPC 35:3;O2	C47H86NO16P	[M+NH4]+
969.6032284						
969.6840329	969.6833	0.0008	0.8250	Hex2Cer 37:1;O5	C49H93NO16	[M+NH4]+
	969.7055	0.0014	1.4437	PC 49:10	C57H94NO8P	[M+NH4]+
969.7041206						
969.7041206	969.7055	0.0014	1.4437	PC O-49:11;O	C57H94NO8P	[M+NH4]+
	969.7055	0.0014	1.4437	PE 52:10	C57H94NO8P	[M+NH4]+
969.7041206						
969.7041206	969.7055	0.0014	1.4437	PE O-52:11;O	C57H94NO8P	[M+NH4]+
	969.7033	0.0008	0.8250	EPC 50:2;O5	C52H103N2O9PK	[M+K]+
969.7041206						
969.7041206	969.7033	0.0008	0.8250	SM 47:2;O5	C52H103N2O9PK	[M+K]+
	970.6098	0.0007	0.7212	Hex2Cer 39:6;O5	C51H87NO16	[M+H]+
970.6090940						
970.6090940	970.6098	0.0007	0.7212	Hex2Cer 39:5;O6	C51H89NO17	[M+H-H2O]+

	970.6098	0.0025	2.5757	Hex2Cer 39:6;O5	C51H87NO16	[M+H]+
970.6122476						
970.6122476	970.6098	0.0025	2.5757	Hex2Cer 39:5;O6	C51H89NO17	[M+H-H2O]+
	970.6144	0.0021	2.1636	PS 47:7;O	C53H90NO11PNa	[M+Na]+
970.6122476						
970.6122476	970.6145	0.0023	2.3696	IPC 44:3;O3	C50H94NO12PK	[M+K]+
	970.6954	0.0001	0.1030	IPC 44:0;O5	C50H100NO14P	[M+H]+
970.6953010						
970.6953010	970.6954	0.0001	0.1030	HexCer 49:6;O5	C55H97NO11Na	[M+Na]+
	970.6954	0.0001	0.1030	PI 41:0;O	C50H97O14P	[M+NH4]+
970.6953010						
970.6953010	970.6955	0.0002	0.2060	HexCer 46:1;O6	C52H101NO12K	[M+K]+
	971.6179	0.0008	0.8234	MIPC 35:2;O2	C47H88NO16P	[M+NH4]+
971.6170534						
971.6170534	971.6162	0.0009	0.9263	TG 58:15;O	C61H88O7K	[M+K]+
	971.6162	0.0009	0.9263	TG O-58:16;O2	C61H88O7K	[M+K]+
971.6170534						
971.6207551	971.6219	0.0012	1.2350	PI 43:5;O	C52H91O14P	[M+H]+
	971.6195	0.0012	1.2351	PI 41:2;O	C50H93O14PNa	[M+Na]+
971.6207551						
971.6207551	971.6179	0.0029	2.9847	MIPC 35:2;O2	C47H88NO16P	[M+NH4]+
	971.6162	0.0046	4.7344	TG 58:15;O	C61H88O7K	[M+K]+
971.6207551						
971.6207551	971.6162	0.0046	4.7344	TG O-58:16;O2	C61H88O7K	[M+K]+
	971.6254	0.0046	4.7343	SQDG 42:1	C51H96O12SK	[M+K]+
971.6207551						
971.6985607	971.6989	0.0003	0.3087	Hex2Cer 37:0;O5	C49H95NO16	[M+NH4]+
	971.6978	0.0008	0.8233	EPC 53:6;O3	C55H101N2O7PK	[M+K]+
971.6985607						
971.6985607	971.6978	0.0008	0.8233	SM 50:6;O3	C55H101N2O7PK	[M+K]+
	971.7029	0.0025	2.5728	DGDG 39:1	C54H100O15	[M+H-H2O]+
971.7004752						
971.7004752	971.6989	0.0016	1.6466	Hex2Cer 37:0;O5	C49H95NO16	[M+NH4]+
	972.3103	0.0030	3.0854	CoA 15:1	C36H62N7O17P3S	[M+H-H2O]+
972.3133318						
972.6276650	972.6254	0.0023	2.3647	Hex2Cer 39:5;O5	C51H89NO16	[M+H]+
	972.6254	0.0023	2.3647	Hex2Cer 39:4;O6	C51H91NO17	[M+H-H2O]+
972.6276650						
972.6276650	972.63	0.0024	2.4675	PS 47:6;O	C53H92NO11PNa	[M+Na]+

	972.6302	0.0025	2.5703	IPC 44:2;O3	C50H96NO12PK	[M+K]+
972.6276650						
972.6304153	972.63	0.0004	0.4113	PS 47:6;O	C53H92NO11PNa	[M+Na]+
	972.6302	0.0002	0.2056	IPC 44:2;O3	C50H96NO12PK	[M+K]+
972.6304153						
973.6289447	973.6293	0.0003	0.3081	PA 54:11;O	C57H91O9PNa	[M+Na]+
	973.6293	0.0003	0.3081	PG O-51:12	C57H91O9PNa	[M+Na]+
973.6289447						
973.6289447	973.6294	0.0005	0.5135	PG 48:6	C54H95O10PK	[M+K]+
	973.6294	0.0005	0.5135	PG O-48:7;O	C54H95O10PK	[M+K]+
973.6289447						
973.6315265	973.6318	0.0003	0.3081	TG 58:14;O	C61H90O7K	[M+K]+
	973.6318	0.0003	0.3081	TG O-58:15;O2	C61H90O7K	[M+K]+
973.6315265						
973.6978599	973.698	0.0002	0.2054	EPC 52:6;O5	C54H99N2O9PNa	[M+Na]+
	973.698	0.0002	0.2054	SM 49:6;O5	C54H99N2O9PNa	[M+Na]+
973.6978599						
973.6978599	973.6968	0.0011	1.1297	SHexCer 43:0;O5	C49H97NO14S	[M+NH4]+
	973.6982	0.0003	0.3081	EPC 49:1;O6	C51H103N2O10PK	[M+K]+
973.6978599						
973.6978599	973.6982	0.0003	0.3081	SM 46:1;O6	C51H103N2O10PK	[M+K]+
	975.6086	0.0016	1.6400	PG 50:11	C56H89O10PNa	[M+Na]+
975.6069745						
975.6069745	975.6086	0.0016	1.6400	PG O-50:12;O	C56H89O10PNa	[M+Na]+
	975.6087	0.0017	1.7425	PG 47:6;O	C53H93O11PK	[M+K]+
975.6069745						
976.6094488	976.6097	0.0002	0.2048	IPC 42:2;O6	C48H92NO15PNa	[M+Na]+
	976.6121	0.0013	1.3311	IPC 44:5;O6	C50H90NO15P	[M+H]+
976.6108009						
976.6108009	976.6121	0.0013	1.3311	MIPC 38:3;O2	C50H92NO16P	[M+H-H2O]+
	976.6097	0.0011	1.1263	IPC 42:2;O6	C48H92NO15PNa	[M+Na]+
976.6108009						
976.6108009	976.6122	0.0014	1.4335	Hex2Cer 40:5;O2	C52H91NO13K	[M+K]+
	977.6114	0.0019	1.9435	PI 45:8	C54H89O13P	[M+H]+
977.6132783						
977.6132783	977.6114	0.0019	1.9435	PI O-45:9;O	C54H89O13P	[M+H]+
	977.6114	0.0019	1.9435	PI 45:7;O	C54H91O14P	[M+H-H2O]+

977.6132783						
977.6132783	977.6156	0.0023	2.3527	Hex2Cer 37:5;O6	C49H85NO17	[M+NH4]+
	978.5103	0.0028	2.8615	PIP 38:7	C47H78O16P2	[M+NH4]+
978.5075049						
978.5120384	978.5103	0.0017	1.7373	PIP 38:7	C47H78O16P2	[M+NH4]+
	978.6253	0.0003	0.3066	IPC 42:1;O6	C48H94NO15PNa	[M+Na]+
978.6255995						
978.6385393	978.636	0.0026	2.6568	Hex2Cer 38:3;O6	C50H91NO17	[M+H]+
	978.6406	0.0020	2.0437	IPC 46:5;O3	C52H94NO12PNa	[M+Na]+
978.6385393						
978.6385393	978.6407	0.0022	2.2480	IPC 43:0;O4	C49H98NO13PK	[M+K]+
	978.6406	0.0010	1.0218	IPC 46:5;O3	C52H94NO12PNa	[M+Na]+
978.6415913						
978.6415913	978.643	0.0014	1.4306	PI O-45:9	C54H89O12P	[M+NH4]+
	978.6407	0.0009	0.9196	IPC 43:0;O4	C49H98NO13PK	[M+K]+
978.6415913						
987.6938550	987.6938	0.0000	0.0000	Hex2Cer 37:0;O6	C49H95NO17	[M+NH4]+
	992.5987	0.0024	2.4179	PS 49:10;O	C55H88NO11PNa	[M+Na]+
992.5963556						
992.5963556	992.5989	0.0025	2.5186	IPC 46:6;O3	C52H92NO12PK	[M+K]+
	993.8821	0.0000	0.0000	DG 60:3;O	C63H118O6Na	[M+Na]+
993.8820289						
993.8820289	993.8821	0.0000	0.0000	DG O-60:4;O2	C63H118O6Na	[M+Na]+
	993.8821	0.0000	0.0000	TG 60:2	C63H118O6Na	[M+Na]+
993.8820289						
993.8820289	993.8821	0.0000	0.0000	TG O-60:3;O	C63H118O6Na	[M+Na]+
	994.6144	0.0014	1.4076	PS 49:9;O	C55H90NO11PNa	[M+Na]+
994.6129757						
994.6129757	994.6145	0.0015	1.5081	IPC 46:5;O3	C52H94NO12PK	[M+K]+
	994.7165	0.0000	0.0000	Hex2Cer 42:2;O2	C54H101NO13Na	[M+Na]+
994.7165265						
995.7190079	995.7189	0.0001	0.1004	EPC 52:3;O5	C54H105N2O9PK	[M+K]+
	995.7189	0.0001	0.1004	SM 49:3;O5	C54H105N2O9PK	[M+K]+
995.7190079						
995.7208011	995.7216	0.0008	0.8034	SQDG 48:3	C57H104O12S	[M+H-H2O]+
	995.7212	0.0004	0.4017	PC 51:11	C59H96NO8P	[M+NH4]+
995.7208011						
995.7208011	995.7212	0.0004	0.4017	PC O-51:12;O	C59H96NO8P	[M+NH4]+

	995.7212	0.0004	0.4017	PE 54:11	C59H96NO8P	[M+NH4]+
995.7208011						
995.7208011	995.7212	0.0004	0.4017	PE O-54:12;O	C59H96NO8P	[M+NH4]+
	996.7322	0.0001	0.1003	Hex2Cer 42:1;O2	C54H103NO13Na	[M+Na]+
996.7322653						
997.5623420	997.5608	0.0016	1.6039	MIPC 35:5;O4	C47H82NO18P	[M+NH4]+
	997.5883	0.0001	0.1002	DGDG 41:11	C56H84O15	[M+H]+
997.5884212						
997.5913256	997.5931	0.0017	1.7041	PG 49:9;O	C55H91O11PK	[M+K]+
	997.7372	0.0016	1.6036	SQDG 48:2	C57H106O12S	[M+H-H2O]+
997.7355615						
997.7355615	997.7368	0.0013	1.3029	PC 51:10	C59H98NO8P	[M+NH4]+
	997.7368	0.0013	1.3029	PC O-51:11;O	C59H98NO8P	[M+NH4]+
997.7355615						
997.7355615	997.7368	0.0013	1.3029	PE 54:10	C59H98NO8P	[M+NH4]+
	997.7368	0.0013	1.3029	PE O-54:11;O	C59H98NO8P	[M+NH4]+
997.7355615						
997.7355615	997.7346	0.0010	1.0023	EPC 52:2;O5	C54H107N2O9PK	[M+K]+
	997.7346	0.0010	1.0023	SM 49:2;O5	C54H107N2O9PK	[M+K]+
997.7355615						
998.5687808	998.5729	0.0042	4.2060	PIP 39:4	C48H86O16P2	[M+NH4]+
	998.5731	0.0043	4.3061	IPC 44:5;O5	C50H90NO14PK	[M+K]+
998.5687808						
998.5727114	998.5729	0.0002	0.2003	PIP 39:4	C48H86O16P2	[M+NH4]+
	998.5731	0.0003	0.3004	IPC 44:5;O5	C50H90NO14PK	[M+K]+
998.5727114						
998.5923940	998.594	0.0016	1.6023	IPC 44:5;O6	C50H90NO15PNa	[M+Na]+
	999.5764	0.0002	0.2001	MIPC 35:4;O4	C47H84NO18P	[M+NH4]+
999.5762137						
1004.3384250	1004.3365	0.0019	1.8918	CoA 16:1	C37H64N7O17P3S	[M+H]+
	1004.3365	0.0019	1.8918	CoA 16:0;O	C37H66N7O18P3S	[M+H-H2O]+
1004.3384250						
1004.3407390	1004.3365	0.0042	4.1819	CoA 16:1	C37H64N7O17P3S	[M+H]+
	1004.3365	0.0042	4.1819	CoA 16:0;O	C37H66N7O18P3S	[M+H-H2O]+
1004.3407390						
1006.6018800	1006.6015	0.0004	0.3974	PI 45:10;O	C54H85O14P	[M+NH4]+
	1011.6761	0.0013	1.2850	SHexCer 45:3;O6	C51H95NO15S	[M+NH4]+
1011.6747380						
1012.3049380	1012.3052	0.0003	0.2964	CoA 17:4	C38H60N7O17P3S	[M+H]+

	1012.3052	0.0003	0.2964	CoA 17:3;O	C38H62N7O18P3S	[M+H-H2O]+
1012.3049380						
1012.6787940	1012.6766	0.0022	2.1725	PC 52:12	C60H96NO8PNa	[M+Na]+
	1012.6766	0.0022	2.1725	PE 55:12	C60H96NO8PNa	[M+Na]+
1012.6787940						
1012.6787940	1012.6767	0.0021	2.0737	PC 49:7;O	C57H100NO9PK	[M+K]+
	1012.6767	0.0021	2.0737	PE 52:7;O	C57H100NO9PK	[M+K]+
1012.6787940						
1012.6787940	1012.6767	0.0021	2.0737	PS O-51:7	C57H100NO9PK	[M+K]+
	1012.6824	0.0014	1.3825	IPC 47:3;O4	C53H100NO13PNa	[M+Na]+
1012.6810560						
1013.5839290	1013.588	0.0040	3.9464	PI O-46:9	C55H91O12PK	[M+K]+
	1013.6883	0.0010	0.9865	Hex2Cer 42:6;O4	C54H93NO15	[M+NH4]+
1013.6893050						
1013.6908940	1013.6917	0.0008	0.7892	SHexCer 45:2;O6	C51H97NO15S	[M+NH4]+
	1014.5889	0.0009	0.8871	MIPC 38:4;O2	C50H90NO16PNa	[M+Na]+
1014.5880370						
1014.5916040	1014.5913	0.0003	0.2957	MIPC 40:6;O3	C52H90NO17P	[M+H-H2O]+
	1014.6922	0.0018	1.7739	PC 52:11	C60H98NO8PNa	[M+Na]+
1014.6939730						
1014.6939730	1014.6922	0.0018	1.7739	PC O-52:12;O	C60H98NO8PNa	[M+Na]+
	1014.6922	0.0018	1.7739	PE 55:11	C60H98NO8PNa	[M+Na]+
1014.6939730						
1014.6939730	1014.6922	0.0018	1.7739	PE O-55:12;O	C60H98NO8PNa	[M+Na]+
	1014.6924	0.0016	1.5768	PC 49:6;O	C57H102NO9PK	[M+K]+
1014.6939730						
1014.6939730	1014.6924	0.0016	1.5768	PE 52:6;O	C57H102NO9PK	[M+K]+
	1014.6924	0.0016	1.5768	PS O-51:6	C57H102NO9PK	[M+K]+
1014.6939730						
1015.5991450	1015.6036	0.0045	4.4309	PI O-46:8	C55H93O12PK	[M+K]+
	1015.5941	0.0050	4.9232	SQDG 46:7	C55H92O12SK	[M+K]+
1015.5991450						
1015.6004530	1015.6036	0.0032	3.1508	PI O-46:8	C55H93O12PK	[M+K]+
	1015.6716	0.0008	0.7877	DGDG 43:7	C58H96O15	[M+H-H2O]+
1015.6723980						
1016.5992540	1016.5987	0.0005	0.4918	PS 51:12;O	C57H88NO11PNa	[M+Na]+
	1016.6046	0.0017	1.6722	MIPC 38:3;O2	C50H92NO16PNa	[M+Na]+

1016.6028570						
1016.6182940	1016.6199	0.0016	1.5738	PIP 40:2	C49H92O16P2	[M+NH4]+
	1016.62	0.0017	1.6722	IPC 45:3;O5	C51H96NO14PK	[M+K]+
1016.6182940						
1016.6219180	1016.6223	0.0003	0.2951	PI 47:11	C56H87O13P	[M+NH4]+
	1016.6223	0.0003	0.2951	PI O-47:12;O	C56H87O13P	[M+NH4]+
1016.6219180						
1017.6054350	1017.6063	0.0008	0.7862	PI 47:10;O	C56H89O14P	[M+H]+
	1017.6039	0.0015	1.4741	PIP 40:1;O	C49H94O17P2	[M+H]+
1017.6054350						
1017.6054350	1017.6039	0.0016	1.5723	PI 45:7;O	C54H91O14PNa	[M+Na]+
	1019.639	0.0000	0.0000	MIPC 36:1;O4	C48H92NO18P	[M+NH4]+
1019.6389840						
1019.6411990	1019.639	0.0022	2.1576	MIPC 36:1;O4	C48H92NO18P	[M+NH4]+
	1020.623	0.0002	0.1960	MIPC 36:0;O5	C48H94NO19P	[M+H]+
1020.6232090						
1020.6232090	1020.623	0.0002	0.1960	Hex2Cer 41:6;O5	C53H91NO16Na	[M+Na]+
	1020.6232	0.0001	0.0980	Hex2Cer 38:1;O6	C50H95NO17K	[M+K]+
1020.6232090						
1020.6449910	1020.6454	0.0004	0.3919	PC 50:10;O	C58H96NO9PK	[M+K]+
	1020.6454	0.0004	0.3919	PE 53:10;O	C58H96NO9PK	[M+K]+
1020.6449910						
1020.6449910	1020.6454	0.0004	0.3919	PS O-52:10	C58H96NO9PK	[M+K]+
	1025.5921	0.0004	0.3900	MIPC 37:5;O4	C49H86NO18P	[M+NH4]+
1025.5924600						
1030.5992720	1030.5992	0.0001	0.0970	PIP 40:3;O	C49H90O17P2	[M+NH4]+
	1030.5993	0.0000	0.0000	IPC 45:4;O6	C51H94NO15PK	[M+K]+
1030.5992720						
1030.6007910	1030.6015	0.0007	0.6792	PI 47:12;O	C56H85O14P	[M+NH4]+
	1030.5992	0.0016	1.5525	PIP 40:3;O	C49H90O17P2	[M+NH4]+
1030.6007910						
1030.6007910	1030.5993	0.0015	1.4555	IPC 45:4;O6	C51H94NO15PK	[M+K]+
	1031.3671	0.0046	4.4601	PIP3 29:0	C38H76O22P4Na	[M+Na]+
1031.3624820						
1032.3689430	1032.3678	0.0011	1.0655	CoA 18:1	C39H68N7O17P3S	[M+H]+
	1032.3678	0.0011	1.0655	CoA 18:0;O	C39H70N7O18P3S	[M+H-H2O]+
1032.3689430						
1032.3710090	1032.3678	0.0032	3.0997	CoA 18:1	C39H68N7O17P3S	[M+H]+
1032.3710090	1032.3678	0.0032	3.0997	CoA 18:0;O	C39H70N7O18P3S	[M+H-H2O]+
1035.6366290	1035.6381	0.0014	1.3518	DGDG 40:4	C55H96O15K	[M+K]+

**1036.5722660**	**1036.5734**	**0.0012**	**1.1577**	**MIPC 37:2;O3**	**C49H92NO17PK**	**[M+K]+**

Supplementary Table S6: Annotations for upregulated ion signals in negative-ion mode

Input Mass	Matched Mass	Delta	ppm	Name	Formula	Adduct
251.2023165	251.2017	0.001	2.7866	FA 16:2	C16H28O2	[M-H]-
282.2438263	282.2439	0.000	0.0000	NAE 15:1	C17H33NO2	[M-H]-
282.2438263	282.2439	0.000	0.0000	SPB 17:2;O2	C17H33NO2	[M-H]-
301.2172010	301.2173	0.000	0.3320	FA 20:5	C20H30O2	[M-H]-
301.2172010	301.2173	0.000	0.3320	ST 20:2;O2	C20H30O2	[M-H]-
303.2328955	303.233	0.000	0.3298	FA 20:4	C20H32O2	[M-H]-
303.2328955	303.233	0.000	0.3298	ST 20:1;O2	C20H32O2	[M-H]-
305.2485472	305.2486	0.000	0.3276	FA 20:3	C20H34O2	[M-H]-
305.2485472	305.2486	0.000	0.3276	ST 20:0;O2	C20H34O2	[M-H]-
315.2539707	315.2541	0.000	0.3172	FA 18:0;O2	C18H36O4	[M-H]-
315.2539707	315.2541	0.000	0.3172	MG 15:0	C18H36O4	[M-H]-
315.2539707	315.2541	0.000	0.3172	MG O-15:1;O	C18H36O4	[M-H]-
315.2539707	315.2541	0.000	0.3172	FA 17:0	C17H34O2	[M+Formate]-
315.2539707	315.2541	0.000	0.3172	FA 16:0	C16H32O2	[M+OAc]-
327.2329824	327.233	0.000	0.0000	FA 22:6	C22H32O2	[M-H]-
327.2329824	327.233	0.000	0.0000	ST 22:3;O2	C22H32O2	[M-H]-
329.2485741	329.2486	0.000	0.0000	FA 22:5	C22H34O2	[M-H]-
329.2485741	329.2486	0.000	0.0000	ST 22:2;O2	C22H34O2	[M-H]-
330.2438810	330.2439	0.000	0.0000	NAE 19:5	C21H33NO2	[M-H]-
339.3265704	339.3269	0.000	0.8841	FA 22:0	C22H44O2	[M-H]-
360.2543851	360.2544	0.000	0.0000	NAE 20:5;O	C22H35NO3	[M-H]-
362.2707854	362.2701	0.001	1.9323	NAE 20:4;O	C22H37NO3	[M-H]-

365.3420088	365.3425	0.001	1.3686	FA 24:1	C24H46O2	[M-H]-
367.3577025	367.3582	0.001	1.3611	FA 24:0	C24H48O2	[M-H]-
376.2259635	376.2258	0.000	0.2658	SPBP 18:2;O2	C18H36NO5P	[M-H]-
376.2259635	376.226	0.000	0.2658	CAR 12:1	C19H35NO4	[M+Cl]-
376.2259635	376.226	0.000	0.2658	NAE 17:2;O2	C19H35NO4	[M+Cl]-
376.2259635	376.226	0.000	0.2658	SPB 19:3;O4	C19H35NO4	[M+Cl]-
384.1929519	384.1947	0.002	4.6851	NAE 18:5;O2	C20H31NO4	[M+Cl]-
384.1929519	384.1947	0.002	4.6851	ST 18:1;O3;G	C20H31NO4	[M+Cl]-
385.1873320	385.1868	0.001	1.2981	ST 19:1;O8	C19H30O8	[M-H]-
385.1873320	385.1868	0.001	1.2981	FA 18:4;O4	C18H28O6	[M+Formate]-
385.1873320	385.1868	0.001	1.2981	ST 18:1;O6	C18H28O6	[M+Formate]-
385.1873320	385.1868	0.001	1.2981	FA 17:4;O4	C17H26O6	[M+OAc]-
388.2854437	388.2857	0.000	0.7726	NAE 22:5;O	C24H39NO3	[M-H]-
394.1631332	394.1636	0.001	1.2685	LPE 11:2;O	C16H30NO8P	[M-H]-
394.1631332	394.1636	0.001	1.2685	LPS O-10:2	C16H30NO8P	[M-H]-
394.1631332	394.1638	0.001	1.7759	CAR 10:2;O3	C17H29NO7	[M+Cl]-
394.1631332	394.1636	0.001	1.2685	SPBP 15:3;O3	C15H28NO6P	[M+Formate]-
394.1631332	394.1636	0.001	1.2685	SPBP 14:3;O3	C14H26NO6P	[M+OAc]-
395.3888422	395.3895	0.001	1.5175	FA 26:0	C26H52O2	[M-H]-
402.1436004	402.144	0.000	0.9947	NAT 11:0;O4	C13H27NO8S	[M+Formate]-
402.1436004	402.144	0.000	0.9947	NAT 10:0;O4	C12H25NO8S	[M+OAc]-
410.1565979	410.1586	0.002	4.8762	LPS 10:1	C16H30NO9P	[M-H]-
410.1565979	410.1586	0.002	4.8762	LPS O-10:2;O	C16H30NO9P	[M-H]-
410.1565979	410.1586	0.002	4.8762	LPE 10:2	C15H28NO7P	[M+Formate]-
410.1565979	410.1586	0.002	4.8762	SPBP 15:3;O4	C15H28NO7P	[M+Formate]-

410.1565979	410.1586	0.002	4.8762	SPBP 14:3;O4	C14H26NO7P	[M+OAc]-
423.2879060	423.2881	0.000	0.4725	LPA O-18:0	C21H45O6P	[M-H]-
423.2879060	423.2883	0.000	0.9450	FA 22:0;O3	C22H44O5	[M+Cl]-
423.2879060	423.2883	0.000	0.9450	MG 19:0;O	C22H44O5	[M+Cl]-
433.2712757	433.2725	0.001	2.7696	LPA O-19:2	C22H43O6P	[M-H]-
433.2712757	433.2726	0.001	3.2312	DG 20:1	C23H42O5	[M+Cl]-
433.2712757	433.2726	0.001	3.2312	DG O-20:2;O	C23H42O5	[M+Cl]-
433.2712757	433.2726	0.001	3.2312	FA 23:2;O3	C23H42O5	[M+Cl]-
433.2712757	433.2726	0.001	3.2312	MG 20:2;O	C23H42O5	[M+Cl]-
435.2866905	435.2881	0.001	3.2163	LPA O-19:1	C22H45O6P	[M-H]-
435.2866905	435.2883	0.002	3.6757	DG 20:0	C23H44O5	[M+Cl]-
435.2866905	435.2883	0.002	3.6757	DG O-20:1;O	C23H44O5	[M+Cl]-
435.2866905	435.2883	0.002	3.6757	FA 23:1;O3	C23H44O5	[M+Cl]-
435.2866905	435.2883	0.002	3.6757	MG 20:1;O	C23H44O5	[M+Cl]-
437.2312944	437.231	0.000	0.6861	LPA 17:1;O	C20H39O8P	[M-H]-
437.2312944	437.231	0.000	0.6861	LPG O-14:2	C20H39O8P	[M-H]-
437.2312944	437.231	0.000	0.6861	LPA O-16:2	C19H37O6P	[M+Formate]-
437.2312944	437.231	0.000	0.6861	LPA O-15:2	C18H35O6P	[M+OAc]-
445.2517059	445.2515	0.000	0.4492	ST 27:5;O3	C27H38O3	[M+Cl]-
446.1838357	446.1854	0.002	3.5860	NAT 18:4;O4	C20H33NO8S	[M-H]-
446.1838357	446.1854	0.002	3.5860	ST 18:1;O6;T	C20H33NO8S	[M-H]-
446.1838357	446.182	0.002	4.0342	ST 21:5;O7;G	C23H29NO8	[M-H]-
446.1838357	446.1854	0.002	3.5860	NAT 17:4;O2	C19H31NO6S	[M+Formate]-
446.1838357	446.182	0.002	4.0342	ST 20:5;O5;G	C22H27NO6	[M+Formate]-
	446.1854	0.002	3.5860	NAT 16:4;O2	C18H29NO6S	[M+OAc]-

446.1838357						
446.1838357	446.182	0.002	4.0342	ST 19:5;O5;G	C21H25NO6	[M+OAc]-
	452.1691	0.002	4.6443	LPS 12:2;O	C18H32NO10P	[M-H]-
452.1670257						
452.1670257	452.1691	0.002	4.6443	LPE 12:3;O	C17H30NO8P	[M+Formate]-
	452.1691	0.002	4.6443	LPS O-11:3	C17H30NO8P	[M+Formate]-
452.1670257						
452.1670257	452.1691	0.002	4.6443	LPE 11:3;O	C16H28NO8P	[M+OAc]-
	452.284	0.002	3.3165	NAT 23:4	C25H43NO4S	[M-H]-
452.2855385						
454.2717852	454.273	0.001	2.6416	CAR 18:4	C25H41NO4	[M+Cl]-
	454.273	0.001	2.6416	NAE 23:5;O2	C25H41NO4	[M+Cl]-
454.2717852						
454.2717852	454.273	0.001	2.6416	ST 23:1;O3;G	C25H41NO4	[M+Cl]-
	457.2361	0.000	0.2187	LPA 20:4	C23H39O7P	[M-H]-
457.2361694						
457.2361694	457.2361	0.000	0.2187	LPA O-20:5;O	C23H39O7P	[M-H]-
	457.2361	0.000	0.2187	PA O-20:4	C23H39O7P	[M-H]-
457.2361694						
457.2361694	457.2362	0.000	0.2187	DG 21:4;O	C24H38O6	[M+Cl]-
	457.2362	0.000	0.2187	DG O-21:5;O2	C24H38O6	[M+Cl]-
457.2361694						
457.2361694	457.2362	0.000	0.2187	FA 24:5;O4	C24H38O6	[M+Cl]-
	457.2362	0.000	0.2187	ST 18:1;O;Hex	C24H38O6	[M+Cl]-
457.2361694						
457.2361694	457.2362	0.000	0.2187	ST 24:2;O6	C24H38O6	[M+Cl]-
	459.2025	0.001	1.3066	ST 19:4;O2;GlcA	C25H32O8	[M-H]-
459.2030792						
459.2030792	459.2025	0.001	1.3066	ST 19:5;O3;Hex	C25H32O8	[M-H]-
	459.2025	0.001	1.3066	ST 25:6;O8	C25H32O8	[M-H]-
459.2030792						
459.2030792	459.2025	0.001	1.3066	ST 18:5;O;Hex	C24H30O6	[M+Formate]-
	459.2025	0.001	1.3066	ST 24:6;O6	C24H30O6	[M+Formate]-
459.2030792						
459.2030792	459.2025	0.001	1.3066	ST 23:6;O6	C23H28O6	[M+OAc]-
	459.2153	0.001	1.0888	LPA 19:4;O	C22H37O8P	[M-H]-
459.2148017						
459.2148017	459.2155	0.001	1.5243	DG 20:4;O2	C23H36O7	[M+Cl]-

459.2148017	459.2155	0.001	1.5243	ST 23:2;O7	C23H36O7	[M+Cl]-
459.2148017	459.2153	0.001	1.0888	LPA O-18:5	C21H35O6P	[M+Formate]-
463.0818600	463.0835	0.002	3.6710	ST 19:4;O6;S	C19H24O9S	[M+Cl]-
466.2947810	466.2939	0.001	1.9301	LPC 14:0	C22H46NO7P	[M-H]-
466.2947810	466.2939	0.001	1.9301	LPC O-14:1;O	C22H46NO7P	[M-H]-
466.2947810	466.2939	0.001	1.9301	LPE 17:0	C22H46NO7P	[M-H]-
466.2947810	466.2939	0.001	1.9301	LPE O-17:1;O	C22H46NO7P	[M-H]-
466.2947810	466.2941	0.001	1.5012	CAR 16:0;O2	C23H45NO6	[M+Cl]-
466.2947810	466.2941	0.001	1.5012	NAE 21:1;O4	C23H45NO6	[M+Cl]-
466.2947810	466.2939	0.001	1.9301	SPBP 20:1;O2	C20H42NO5P	[M+OAc]-
469.2922248	469.2936	0.001	2.9832	LPG O-16:0	C22H47O8P	[M-H]-
469.2922248	469.2936	0.001	2.9832	LPA O-18:0	C21H45O6P	[M+Formate]-
469.2922248	469.2936	0.001	2.9832	LPA O-17:0	C20H43O6P	[M+OAc]-
471.2672979	471.2672	0.000	0.2122	ST 29:6;O3	C29H40O3	[M+Cl]-
474.2632691	474.2626	0.001	1.2651	LPC 15:3	C23H42NO7P	[M-H]-
474.2632691	474.2626	0.001	1.2651	LPC O-15:4;O	C23H42NO7P	[M-H]-
474.2632691	474.2626	0.001	1.2651	LPE 18:3	C23H42NO7P	[M-H]-
474.2632691	474.2626	0.001	1.2651	LPE O-18:4;O	C23H42NO7P	[M-H]-
474.2632691	474.2628	0.001	1.0543	CAR 17:3;O2	C24H41NO6	[M+Cl]-
474.2632691	474.2628	0.001	1.0543	NAE 22:4;O4	C24H41NO6	[M+Cl]-
474.2632691	474.2628	0.001	1.0543	ST 22:0;O5;G	C24H41NO6	[M+Cl]-
476.2788830	476.2783	0.001	1.2598	LPC 15:2	C23H44NO7P	[M-H]-
476.2788830	476.2783	0.001	1.2598	LPC O-15:3;O	C23H44NO7P	[M-H]-
476.2788830	476.2783	0.001	1.2598	LPE 18:2	C23H44NO7P	[M-H]-
	476.2783	0.001	1.2598	LPE O-18:3;O	C23H44NO7P	[M-H]-

476.2788830						
476.2788830	476.2784	0.000	0.8398	CAR 17:2;O2	C24H43NO6	[M+Cl]-
	476.2784	0.000	0.8398	NAE 22:3;O4	C24H43NO6	[M+Cl]-
476.2788830						
476.2809102	476.2806	0.000	0.6299	ST 28:7;O3;G	C30H39NO4	[M-H]-
	478.1848	0.001	1.6730	LPS 14:3;O	C20H34NO10P	[M-H]-
478.1855635						
478.1855635	478.1849	0.001	1.2547	ST 19:1;O8;G	C21H33NO9	[M+Cl]-
	478.1848	0.001	1.6730	LPE 14:4;O	C19H32NO8P	[M+Formate]-
478.1855635						
479.0761262	479.0784	0.002	4.8009	ST 19:4;O7;S	C19H24O10S	[M+Cl]-
	479.257	0.002	3.1298	DG 24:6	C27H40O5	[M+Cl]-
479.2554781						
479.2554781	479.257	0.002	3.1298	FA 27:7;O3	C27H40O5	[M+Cl]-
	479.257	0.002	3.1298	ST 27:4;O5	C27H40O5	[M+Cl]-
479.2554781						
479.2837354	479.2837	0.000	0.2086	ST 27:2;O2;S	C27H44O5S	[M-H]-
	479.3143	0.001	1.2518	LPA 21:0	C24H49O7P	[M-H]-
479.3137220						
479.3137220	479.3143	0.001	1.2518	LPA O-21:1;O	C24H49O7P	[M-H]-
	479.3143	0.001	1.2518	PA O-21:0	C24H49O7P	[M-H]-
479.3137220						
479.3137220	479.3145	0.001	1.6691	DG 22:0;O	C25H48O6	[M+Cl]-
	479.3145	0.001	1.6691	DG O-22:1;O2	C25H48O6	[M+Cl]-
479.3137220						
479.3137220	479.3145	0.001	1.6691	FA 25:1;O4	C25H48O6	[M+Cl]-
	481.1749	0.001	2.4939	ST 20:0;O8;S	C20H34O11S	[M-H]-
481.1737436						
481.1737436	481.1749	0.001	2.4939	ST 19:0;O6;S	C19H32O9S	[M+Formate]-
	481.1749	0.001	2.4939	ST 18:0;O6;S	C18H30O9S	[M+OAc]-
481.1737436						
482.2661433	482.2679	0.002	3.5250	CAR 19:5;O	C26H41NO5	[M+Cl]-
	482.2679	0.002	3.5250	NAE 24:6;O3	C26H41NO5	[M+Cl]-
482.2661433						
482.2661433	482.2679	0.002	3.5250	ST 24:2;O4;G	C26H41NO5	[M+Cl]-
	483.0967	0.001	2.6910	ST 21:6;O8;S	C21H24O11S	[M-H]-
483.0953463						
483.2153997	483.2153	0.000	0.2069	LPA 21:6;O	C24H37O8P	[M-H]-

	483.2153	0.000	0.2069	PA 21:5	C24H37O8P	[M-H]-
483.2153997						
483.2153997	483.2153	0.000	0.2069	PA O-21:6;O	C24H37O8P	[M-H]-
	483.2155	0.000	0.2069	DG 22:6;O2	C25H36O7	[M+Cl]-
483.2153997						
483.2153997	483.2155	0.000	0.2069	ST 19:2;O;GlcA	C25H36O7	[M+Cl]-
	483.2155	0.000	0.2069	ST 19:3;O2;Hex	C25H36O7	[M+Cl]-
483.2153997						
483.2153997	483.2155	0.000	0.2069	ST 25:4;O7	C25H36O7	[M+Cl]-
	484.2834	0.000	0.8260	LPE O-20:5	C25H44NO6P	[M-H]-
484.2829627						
484.2829627	484.2835	0.001	1.2389	CAR 19:4;O	C26H43NO5	[M+Cl]-
	484.2835	0.001	1.2389	NAE 24:5;O3	C26H43NO5	[M+Cl]-
484.2829627						
484.2829627	484.2835	0.001	1.2389	ST 24:1;O4;G	C26H43NO5	[M+Cl]-
	488.1879	0.002	4.5065	NAT 21:6;O2	C23H35NO6S	[M+Cl]-
488.1857406						
488.1857406	488.1879	0.002	4.5065	ST 21:3;O4;T	C23H35NO6S	[M+Cl]-
	488.1926	0.001	1.8435	ST 23:6;O8;G	C25H31NO9	[M-H]-
488.1934750						
488.1934750	488.1926	0.001	1.8435	ST 22:6;O6;G	C24H29NO7	[M+Formate]-
	488.1926	0.001	1.8435	ST 21:6;O6;G	C23H27NO7	[M+OAc]-
488.1934750						
489.1869049	489.1872	0.000	0.6133	ST 27:7;O;S	C27H34O4S	[M+Cl]-
	489.213	0.000	0.0000	ST 20:4;O3;GlcA	C26H34O9	[M-H]-
489.2130254						
489.2130254	489.213	0.000	0.0000	ST 20:5;O4;Hex	C26H34O9	[M-H]-
	489.213	0.000	0.0000	ST 19:4;O;GlcA	C25H32O7	[M+Formate]-
489.2130254						
489.2130254	489.213	0.000	0.0000	ST 19:5;O2;Hex	C25H32O7	[M+Formate]-
	489.213	0.000	0.0000	ST 25:6;O7	C25H32O7	[M+Formate]-
489.2130254						
489.2130254	489.213	0.000	0.0000	ST 18:4;O;GlcA	C24H30O7	[M+OAc]-
	489.213	0.000	0.0000	ST 18:5;O2;Hex	C24H30O7	[M+OAc]-
489.2130254						
489.2130254	489.213	0.000	0.0000	ST 24:6;O7	C24H30O7	[M+OAc]-
	490.2083	0.001	1.0200	ST 23:5;O8;G	C25H33NO9	[M-H]-

490.2077683						
490.2077683	490.2083	0.001	1.0200	ST 22:5;O6;G	C24H31NO7	[M+Formate]-
	490.2083	0.001	1.0200	ST 21:5;O6;G	C23H29NO7	[M+OAc]-
490.2077683						
490.2116515	490.2116	0.000	0.0000	ST 20:1;O7;T	C22H37NO9S	[M-H]-
	490.2116	0.000	0.0000	NAT 19:4;O3	C21H35NO7S	[M+Formate]-
490.2116515						
490.2116515	490.2116	0.000	0.0000	ST 19:1;O5;T	C21H35NO7S	[M+Formate]-
	490.2116	0.000	0.0000	NAT 18:4;O3	C20H33NO7S	[M+OAc]-
490.2116515						
490.2116515	490.2116	0.000	0.0000	ST 18:1;O5;T	C20H33NO7S	[M+OAc]-
	490.2844	0.000	0.0000	NAT 22:2;O3	C24H45NO7S	[M-H]-
490.2843663						
490.2843663	490.2844	0.000	0.0000	NAT 21:2;O	C23H43NO5S	[M+Formate]-
	490.2844	0.000	0.0000	NAT 20:2;O	C22H41NO5S	[M+OAc]-
490.2843663						
493.1743656	493.1749	0.001	1.2166	ST 21:1;O8;S	C21H34O11S	[M-H]-
	493.1749	0.001	1.2166	ST 20:1;O6;S	C20H32O9S	[M+Formate]-
493.1743656						
493.1743656	493.1749	0.001	1.2166	ST 19:1;O6;S	C19H30O9S	[M+OAc]-
	493.3171	0.002	3.0406	DG 25:4;O2	C28H46O7	[M-H]-
493.3185703						
493.3185703	493.3171	0.002	3.0406	ST 22:0;O;GlcA	C28H46O7	[M-H]-
	493.3171	0.002	3.0406	ST 22:1;O2;Hex	C28H46O7	[M-H]-
493.3185703						
493.3185703	493.3171	0.002	3.0406	ST 28:2;O7	C28H46O7	[M-H]-
	493.3171	0.002	3.0406	DG 24:4	C27H44O5	[M+Formate]-
493.3185703						
493.3185703	493.3171	0.002	3.0406	DG O-24:5;O	C27H44O5	[M+Formate]-
	493.3171	0.002	3.0406	FA 27:5;O3	C27H44O5	[M+Formate]-
493.3185703						
493.3185703	493.3171	0.002	3.0406	MG 24:5;O	C27H44O5	[M+Formate]-
	493.3171	0.002	3.0406	ST 27:2;O5	C27H44O5	[M+Formate]-
493.3185703						
493.3185703	493.3171	0.002	3.0406	DG 23:4	C26H42O5	[M+OAc]-
	493.3171	0.002	3.0406	DG O-23:5;O	C26H42O5	[M+OAc]-
493.3185703						
493.3185703	493.3171	0.002	3.0406	FA 26:5;O3	C26H42O5	[M+OAc]-

	493.3171	0.002	3.0406	MG 23:5;O	C26H42O5	[M+OAc]-
493.3185703						
493.3185703	493.3171	0.002	3.0406	ST 26:2;O5	C26H42O5	[M+OAc]-
	494.2679	0.002	3.2371	NAE 25:7;O3	C27H41NO5	[M+Cl]-
494.2663228						
494.2663228	494.2679	0.002	3.2371	ST 25:3;O4;G	C27H41NO5	[M+Cl]-
	495.2752	0.001	2.0191	ST 24:6;O;Hex	C30H40O6	[M-H]-
495.2741718						
495.2741718	495.2752	0.001	2.0191	ST 30:7;O6	C30H40O6	[M-H]-
	495.2729	0.001	2.6248	LPG 17:1	C23H45O9P	[M-H]-
495.2741718						
495.2741718	495.2729	0.001	2.6248	LPG O-17:2;O	C23H45O9P	[M-H]-
	495.2729	0.001	2.6248	PA 20:0;O	C23H45O9P	[M-H]-
495.2741718						
495.2741718	495.2752	0.001	2.0191	ST 29:7;O4	C29H38O4	[M+Formate]-
	495.2729	0.001	2.6248	LPA 19:1	C22H43O7P	[M+Formate]-
495.2741718						
495.2741718	495.2729	0.001	2.6248	LPA O-19:2;O	C22H43O7P	[M+Formate]-
	495.2752	0.001	2.0191	ST 28:7;O4	C28H36O4	[M+OAc]-
495.2741718						
495.2741718	495.2729	0.001	2.6248	LPA 18:1	C21H41O7P	[M+OAc]-
	495.2729	0.001	2.6248	LPA O-18:2;O	C21H41O7P	[M+OAc]-
495.2741718						
496.3187074	496.3199	0.001	2.4178	CAR 21:4	C28H47NO4	[M+Cl]-
	496.3199	0.001	2.4178	Cer 28:5;O3	C28H47NO4	[M+Cl]-
496.3187074						
496.3187074	496.3199	0.001	2.4178	NAE 26:5;O2	C28H47NO4	[M+Cl]-
	496.3199	0.001	2.4178	ST 26:1;O3;G	C28H47NO4	[M+Cl]-
496.3187074						
498.2635084	498.2626	0.001	1.8063	LPE 20:5	C25H42NO7P	[M-H]-
	498.2626	0.001	1.8063	PE O-20:5	C25H42NO7P	[M-H]-
498.2635084						
498.2635084	498.2628	0.001	1.4049	CAR 19:5;O2	C26H41NO6	[M+Cl]-
	498.2628	0.001	1.4049	NAE 24:6;O4	C26H41NO6	[M+Cl]-
498.2635084						
498.2635084	498.2628	0.001	1.4049	ST 18:1;O;HexNAc	C26H41NO6	[M+Cl]-
	498.2628	0.001	1.4049	ST 24:2;O5;G	C26H41NO6	[M+Cl]-

498.2635084						
499.1850689	499.1869	0.002	3.8062	LPG 15:3	C21H37O9P	[M+Cl]-
499.1850689	499.1869	0.002	3.8062	LPG O-15:4;O	C21H37O9P	[M+Cl]-
500.2790249	500.2783	0.001	1.5991	LPC 17:4	C25H44NO7P	[M-H]-
500.2790249	500.2783	0.001	1.5991	LPE 20:4	C25H44NO7P	[M-H]-
500.2790249	500.2783	0.001	1.5991	LPE O-20:5;O	C25H44NO7P	[M-H]-
500.2790249	500.2783	0.001	1.5991	PE O-20:4	C25H44NO7P	[M-H]-
500.2790249	500.2784	0.001	1.1993	CAR 19:4;O2	C26H43NO6	[M+Cl]-
500.2790249	500.2784	0.001	1.1993	NAE 24:5;O4	C26H43NO6	[M+Cl]-
500.2790249	500.2784	0.001	1.1993	ST 18:0;O;HexNAc	C26H43NO6	[M+Cl]-
500.2790249	500.2784	0.001	1.1993	ST 24:1;O5;G	C26H43NO6	[M+Cl]-
500.2803651	500.2818	0.002	2.9983	NAT 21:0;O2	C23H47NO6S	[M+Cl]-
500.2803651	500.2784	0.002	3.7979	CAR 19:4;O2	C26H43NO6	[M+Cl]-
500.2803651	500.2784	0.002	3.7979	NAE 24:5;O4	C26H43NO6	[M+Cl]-
500.2803651	500.2784	0.002	3.7979	ST 18:0;O;HexNAc	C26H43NO6	[M+Cl]-
500.2803651	500.2784	0.002	3.7979	ST 24:1;O5;G	C26H43NO6	[M+Cl]-
501.2823736	501.2811	0.001	2.5934	ST 27:1;O;S	C27H46O4S	[M+Cl]-
501.2823736	501.2834	0.001	1.9949	LPG O-15:0	C21H45O8P	[M+Formate]-
501.2823736	501.2834	0.001	1.9949	LPG O-14:0	C20H43O8P	[M+OAc]-
502.1859905	502.1849	0.001	1.9913	ST 21:3;O8;G	C23H33NO9	[M+Cl]-
502.2079344	502.2083	0.000	0.5974	ST 18:5;O4;HexNAc	C26H33NO9	[M-H]-
502.2079344	502.2083	0.000	0.5974	ST 24:6;O8;G	C26H33NO9	[M-H]-
502.2079344	502.2083	0.000	0.5974	ST 23:6;O6;G	C25H31NO7	[M+Formate]-
502.2079344	502.2083	0.000	0.5974	ST 22:6;O6;G	C24H29NO7	[M+OAc]-
502.2112457	502.2116	0.000	0.7965	ST 21:2;O7;T	C23H37NO9S	[M-H]-
502.2112457	502.2116	0.000	0.7965	NAT 20:5;O3	C22H35NO7S	[M+Formate]-

502.2112457	502.2116	0.000	0.7965	ST 20:2;O5;T	C22H35NO7S	[M+Formate]-
502.2112457	502.2116	0.000	0.7965	NAT 19:5;O3	C21H33NO7S	[M+OAc]-
502.2112457	502.2116	0.000	0.7965	ST 19:2;O5;T	C21H33NO7S	[M+OAc]-
502.2947278	502.2939	0.001	1.5927	LPC 17:3	C25H46NO7P	[M-H]-
502.2947278	502.2939	0.001	1.5927	LPC O-17:4;O	C25H46NO7P	[M-H]-
502.2947278	502.2939	0.001	1.5927	LPE 20:3	C25H46NO7P	[M-H]-
502.2947278	502.2939	0.001	1.5927	LPE O-20:4;O	C25H46NO7P	[M-H]-
502.2947278	502.2939	0.001	1.5927	PE O-20:3	C25H46NO7P	[M-H]-
502.2947278	502.2941	0.001	1.1945	CAR 19:3;O2	C26H45NO6	[M+Cl]-
502.2947278	502.2941	0.001	1.1945	NAE 24:4;O4	C26H45NO6	[M+Cl]-
502.2947278	502.2941	0.001	1.1945	ST 24:0;O5;G	C26H45NO6	[M+Cl]-
504.2257120	504.2273	0.002	3.1732	ST 21:1;O7;T	C23H39NO9S	[M-H]-
504.2257120	504.2239	0.002	3.5698	ST 18:4;O4;HexNAc	C26H35NO9	[M-H]-
504.2257120	504.2239	0.002	3.5698	ST 24:5;O8;G	C26H35NO9	[M-H]-
504.2257120	504.2273	0.002	3.1732	NAT 20:4;O3	C22H37NO7S	[M+Formate]-
504.2257120	504.2273	0.002	3.1732	ST 20:1;O5;T	C22H37NO7S	[M+Formate]-
504.2257120	504.2239	0.002	3.5698	ST 23:5;O6;G	C25H33NO7	[M+Formate]-
504.2257120	504.2273	0.002	3.1732	NAT 19:4;O3	C21H35NO7S	[M+OAc]-
504.2257120	504.2273	0.002	3.1732	ST 19:1;O5;T	C21H35NO7S	[M+OAc]-
504.2257120	504.2239	0.002	3.5698	ST 22:5;O6;G	C24H31NO7	[M+OAc]-
504.2366957	504.2368	0.000	0.1983	LPS 17:3	C23H40NO9P	[M-H]-
504.2366957	504.2368	0.000	0.1983	LPS O-17:4;O	C23H40NO9P	[M-H]-
504.2366957	504.237	0.000	0.5950	CAR 17:4;O4	C24H39NO8	[M+Cl]-
504.2366957	504.237	0.000	0.5950	ST 22:1;O7;G	C24H39NO8	[M+Cl]-
	504.2368	0.000	0.1983	LPC 14:4	C22H38NO7P	[M+Formate]-

504.2366957						
504.2366957	504.2368	0.000	0.1983	LPE 17:4	C22H38NO7P	[M+Formate]-
	504.2368	0.000	0.1983	LPE 16:4	C21H36NO7P	[M+OAc]-
504.2366957						
506.1646329	506.1668	0.002	4.3464	ST 21:6;O8;G	C23H27NO9	[M+Formate]-
	506.2066	0.002	3.3583	ST 20:1;O8;T	C22H37NO10S	[M-H]-
506.2048779						
506.2048779	506.2032	0.002	3.3583	ST 22:5;O7;G	C24H31NO8	[M+Formate]-
	506.2066	0.002	3.3583	NAT 19:4;O4	C21H35NO8S	[M+Formate]-
506.2048779						
506.2048779	506.2066	0.002	3.3583	ST 19:1;O6;T	C21H35NO8S	[M+Formate]-
	506.2032	0.002	3.3583	ST 21:5;O7;G	C23H29NO8	[M+OAc]-
506.2048779						
506.2048779	506.2066	0.002	3.3583	NAT 18:4;O4	C20H33NO8S	[M+OAc]-
	506.2066	0.002	3.3583	ST 18:1;O6;T	C20H33NO8S	[M+OAc]-
506.2048779						
506.3263029	506.3252	0.001	2.1725	LPC 17:1	C25H50NO7P	[M-H]-
	506.3252	0.001	2.1725	LPC O-17:2;O	C25H50NO7P	[M-H]-
506.3263029						
506.3263029	506.3252	0.001	2.1725	LPE 20:1	C25H50NO7P	[M-H]-
	506.3252	0.001	2.1725	LPE O-20:2;O	C25H50NO7P	[M-H]-
506.3263029						
506.3263029	506.3252	0.001	2.1725	PE O-20:1	C25H50NO7P	[M-H]-
	506.3276	0.001	2.5675	ST 30:6;O3;G	C32H45NO4	[M-H]-
506.3263029						
506.3263029	506.3254	0.001	1.7775	CAR 19:1;O2	C26H49NO6	[M+Cl]-
	506.3254	0.001	1.7775	NAE 24:2;O4	C26H49NO6	[M+Cl]-
506.3263029						
508.2330896	508.2317	0.001	2.7546	LPS 16:2;O	C22H40NO10P	[M-H]-
	508.2319	0.001	2.3611	ST 21:0;O8;G	C23H39NO9	[M+Cl]-
508.2330896						
508.2330896	508.2317	0.001	2.7546	LPC 13:3;O	C21H38NO8P	[M+Formate]-
	508.2317	0.001	2.7546	LPE 16:3;O	C21H38NO8P	[M+Formate]-
508.2330896						
508.2330896	508.2317	0.001	2.7546	LPS O-15:3	C21H38NO8P	[M+Formate]-
	508.2317	0.001	2.7546	LPC 12:3;O	C20H36NO8P	[M+OAc]-
508.2330896						
508.2330896	508.2317	0.001	2.7546	LPE 15:3;O	C20H36NO8P	[M+OAc]-

508.2330896	508.2317	0.001	2.7546	LPS O-14:3	C20H36NO8P	[M+OAc]-
509.2534894	509.2545	0.001	1.9637	ST 24:6;O;GlcA	C30H38O7	[M-H]-
509.2534894	509.2521	0.001	2.7491	BMP 17:1	C23H43O10P	[M-H]-
509.2534894	509.2521	0.001	2.7491	LPG 17:2;O	C23H43O10P	[M-H]-
509.2534894	509.2521	0.001	2.7491	LPA 19:2;O	C22H41O8P	[M+Formate]-
509.2534894	509.2521	0.001	2.7491	LPG O-16:3	C22H41O8P	[M+Formate]-
509.2534894	509.2521	0.001	2.7491	LPA 18:2;O	C21H39O8P	[M+OAc]-
509.2534894	509.2521	0.001	2.7491	LPG O-15:3	C21H39O8P	[M+OAc]-
511.1525940	511.1505	0.002	3.9127	BMP 15:4	C21H33O10P	[M+Cl]-
511.1743064	511.174	0.000	0.5869	ST 19:4;O3;GlcA	C25H32O9	[M+Cl]-
511.1743064	511.174	0.000	0.5869	ST 19:5;O4;Hex	C25H32O9	[M+Cl]-
511.1856464	511.1855	0.000	0.3912	ST 20:0;O7;S	C20H34O10S	[M+Formate]-
511.1856464	511.1855	0.000	0.3912	ST 19:0;O7;S	C19H32O10S	[M+OAc]-
514.2580500	514.2575	0.001	0.9723	LPE 20:5;O	C25H42NO8P	[M-H]-
514.2580500	514.2575	0.001	0.9723	LPS O-19:5	C25H42NO8P	[M-H]-
514.2580500	514.2575	0.001	0.9723	PE 20:4	C25H42NO8P	[M-H]-
514.2580500	514.2575	0.001	0.9723	PE O-20:5;O	C25H42NO8P	[M-H]-
514.2580500	514.2577	0.000	0.5834	CAR 19:5;O3	C26H41NO7	[M+Cl]-
514.2580500	514.2577	0.000	0.5834	ST 18:1;O2;HexNAc	C26H41NO7	[M+Cl]-
514.2580500	514.2577	0.000	0.5834	ST 24:2;O6;G	C26H41NO7	[M+Cl]-
514.3286818	514.3303	0.002	3.1108	LPC O-19:4	C27H50NO6P	[M-H]-
514.3286818	514.3303	0.002	3.1108	LPE O-22:4	C27H50NO6P	[M-H]-
514.3286818	514.3305	0.002	3.4997	CAR 21:3;O	C28H49NO5	[M+Cl]-
514.3286818	514.3305	0.002	3.4997	Cer 28:4;O4	C28H49NO5	[M+Cl]-
514.3286818	514.3305	0.002	3.4997	NAE 26:4;O3	C28H49NO5	[M+Cl]-
514.3286818	514.3305	0.002	3.4997	ST 26:0;O4;G	C28H49NO5	[M+Cl]-

	515.2182	0.002	3.4937	LPG 16:2	C22H41O9P	[M+Cl]-
515.2163899						
515.2163899	515.2182	0.002	3.4937	LPG O-16:3;O	C22H41O9P	[M+Cl]-
	516.2006	0.002	3.2933	ST 22:3;O8;G	C24H35NO9	[M+Cl]-
516.2022900						
518.1939730	518.1951	0.001	2.1228	ST 25:7;O6;G	C27H33NO7	[M+Cl]-
	518.1927	0.001	2.3157	LPS 14:1;O	C20H38NO10P	[M+Cl]-
518.1939730						
518.2050732	518.2066	0.002	2.8946	ST 21:2;O8;T	C23H37NO10S	[M-H]-
	518.2032	0.002	3.6665	ST 18:5;O5;HexNAc	C26H33NO10	[M-H]-
518.2050732						
518.2050732	518.2066	0.002	2.8946	NAT 20:5;O4	C22H35NO8S	[M+Formate]-
	518.2066	0.002	2.8946	ST 20:2;O6;T	C22H35NO8S	[M+Formate]-
518.2050732						
518.2050732	518.2032	0.002	3.6665	ST 23:6;O7;G	C25H31NO8	[M+Formate]-
	518.2066	0.002	2.8946	NAT 19:5;O4	C21H33NO8S	[M+OAc]-
518.2050732						
518.2050732	518.2066	0.002	2.8946	ST 19:2;O6;T	C21H33NO8S	[M+OAc]-
	518.2032	0.002	3.6665	ST 22:6;O7;G	C24H29NO8	[M+OAc]-
518.2050732						
518.2928854	518.2946	0.002	3.2800	NAT 27:7;O	C29H45NO5S	[M-H]-
	518.2946	0.002	3.2800	ST 27:4;O3;T	C29H45NO5S	[M-H]-
518.2928854						
519.3605714	519.3611	0.001	0.9627	FA 32:6;O	C32H52O3	[M+Cl]-
	519.3611	0.001	0.9627	MG O-29:7	C32H52O3	[M+Cl]-
519.3605714						
521.3761269	521.3767	0.001	1.1508	FA 32:5;O	C32H54O3	[M+Cl]-
	521.3767	0.001	1.1508	MG O-29:6	C32H54O3	[M+Cl]-
521.3761269						
522.2977393	522.299	0.001	2.4890	CerP 28:6;O2	C28H46NO6P	[M-H]-
	522.2992	0.001	2.6805	CAR 22:6;O	C29H45NO5	[M+Cl]-
522.2977393						
522.2977393	522.2992	0.001	2.6805	NAE 27:7;O3	C29H45NO5	[M+Cl]-
	522.2992	0.001	2.6805	ST 27:3;O4;G	C29H45NO5	[M+Cl]-
522.2977393						
522.3215031	522.3225	0.001	1.9145	ST 30:6;O4;G	C32H45NO5	[M-H]-
	522.3201	0.001	2.6803	LPC 17:1;O	C25H50NO8P	[M-H]-

522.3215031						
522.3215031	522.3201	0.001	2.6803	LPE 20:1;O	C25H50NO8P	[M-H]-
	522.3201	0.001	2.6803	LPS O-19:1	C25H50NO8P	[M-H]-
522.3215031						
522.3215031	522.3201	0.001	2.6803	PE 20:0	C25H50NO8P	[M-H]-
	522.3201	0.001	2.6803	PE O-20:1;O	C25H50NO8P	[M-H]-
522.3215031						
522.3215031	522.3203	0.001	2.2974	CAR 19:1;O3	C26H49NO7	[M+Cl]-
	522.3201	0.001	2.6803	LPC O-16:2	C24H48NO6P	[M+Formate]-
522.3215031						
522.3215031	522.3201	0.001	2.6803	LPE O-19:2	C24H48NO6P	[M+Formate]-
	522.3201	0.001	2.6803	LPC O-15:2	C23H46NO6P	[M+OAc]-
522.3215031						
522.3215031	522.3201	0.001	2.6803	LPE O-18:2	C23H46NO6P	[M+OAc]-
	522.38	0.001	1.5315	CAR 23:3;O2	C30H53NO6	[M-H]-
522.3791916						
522.3791916	522.38	0.001	1.5315	Cer 30:4;O5	C30H53NO6	[M-H]-
	522.38	0.001	1.5315	NAE 28:4;O4	C30H53NO6	[M-H]-
522.3791916						
522.3791916	522.38	0.001	1.5315	ST 28:0;O5;G	C30H53NO6	[M-H]-
	522.38	0.001	1.5315	CAR 22:3	C29H51NO4	[M+Formate]-
522.3791916						
522.3791916	522.38	0.001	1.5315	Cer 29:4;O3	C29H51NO4	[M+Formate]-
	522.38	0.001	1.5315	NAE 27:4;O2	C29H51NO4	[M+Formate]-
522.3791916						
522.3791916	522.38	0.001	1.5315	ST 27:0;O3;G	C29H51NO4	[M+Formate]-
	522.38	0.001	1.5315	CAR 21:3	C28H49NO4	[M+OAc]-
522.3791916						
522.3791916	522.38	0.001	1.5315	Cer 28:4;O3	C28H49NO4	[M+OAc]-
	522.38	0.001	1.5315	NAE 26:4;O2	C28H49NO4	[M+OAc]-
522.3791916						
522.3791916	522.38	0.001	1.5315	ST 26:0;O3;G	C28H49NO4	[M+OAc]-
	522.38	0.000	0.7657	CAR 23:3;O2	C30H53NO6	[M-H]-
522.3804288						
522.3804288	522.38	0.000	0.7657	Cer 30:4;O5	C30H53NO6	[M-H]-
	522.38	0.000	0.7657	NAE 28:4;O4	C30H53NO6	[M-H]-
522.3804288						
522.3804288	522.38	0.000	0.7657	ST 28:0;O5;G	C30H53NO6	[M-H]-

522.3804288	522.38	0.000	0.7657	CAR 22:3	C29H51NO4	[M+Formate]-
522.3804288	522.38	0.000	0.7657	Cer 29:4;O3	C29H51NO4	[M+Formate]-
522.3804288	522.38	0.000	0.7657	NAE 27:4;O2	C29H51NO4	[M+Formate]-
522.3804288	522.38	0.000	0.7657	ST 27:0;O3;G	C29H51NO4	[M+Formate]-
522.3804288	522.38	0.000	0.7657	CAR 21:3	C28H49NO4	[M+OAc]-
522.3804288	522.38	0.000	0.7657	Cer 28:4;O3	C28H49NO4	[M+OAc]-
522.3804288	522.38	0.000	0.7657	NAE 26:4;O2	C28H49NO4	[M+OAc]-
522.3804288	522.38	0.000	0.7657	ST 26:0;O3;G	C28H49NO4	[M+OAc]-
914.4523890	914.452	0.000	0.4374	MIPC 29:3;O5	C41H74NO19P	[M-H]-
914.4523890	914.452	0.000	0.4374	MIPC 28:3;O3	C40H72NO17P	[M+Formate]-
915.2743770	915.274	0.000	0.4370	PIP2 28:7;O	C37H59O20P3	[M-H]-
915.4932517	915.4948	0.002	1.7477	PG 45:12	C51H77O10P	[M+Cl]-
915.5036078	915.5029	0.001	0.7646	PI 41:10	C50H77O13P	[M-H]-
915.5036078	915.5029	0.001	0.7646	PI O-41:11;O	C50H77O13P	[M-H]-
915.5036078	915.5065	0.003	3.1676	SQDG 39:6	C48H80O12S	[M+Cl]-
915.5036078	915.5029	0.001	0.7646	PG 43:11;O	C49H75O11P	[M+Formate]-
915.5036078	915.5029	0.001	0.7646	PG 42:11;O	C48H73O11P	[M+OAc]-
915.5278142	915.5298	0.002	2.1845	SQDG 42:9	C51H80O12S	[M-H]-
916.4649427	916.4677	0.003	2.9461	MIPC 29:2;O5	C41H76NO19P	[M-H]-
916.4649427	916.4677	0.003	2.9461	MIPC 28:2;O3	C40H74NO17P	[M+Formate]-
916.4865935	916.4865	0.000	0.1091	SHexCer 37:3;O6	C43H79NO15S	[M+Cl]-
916.5569399	916.5557	0.001	1.4184	IPC 41:5;O5	C47H84NO14P	[M-H]-
916.5569399	916.5559	0.001	1.2001	Hex2Cer 36:5;O2	C48H83NO13	[M+Cl]-
916.5569399	916.5557	0.001	1.4184	IPC 40:5;O3	C46H82NO12P	[M+Formate]-
916.5569399	916.5557	0.001	1.4184	IPC 39:5;O3	C45H80NO12P	[M+OAc]-
916.5615301	916.5629	0.001	1.4183	PC 44:10	C52H84NO8P	[M+Cl]-

916.5615301	916.5629	0.001	1.4183	PC O-44:11;O	C52H84NO8P	[M+Cl]-
916.5615301	916.5629	0.001	1.4183	PE 47:10	C52H84NO8P	[M+Cl]-
916.5615301	916.5629	0.001	1.4183	PE O-47:11;O	C52H84NO8P	[M+Cl]-
917.4721504	917.4727	0.001	0.5450	SQDG 39:10	C48H72O12S	[M+Formate]-
917.4721504	917.4727	0.001	0.5450	SQDG 38:10	C47H70O12S	[M+OAc]-
917.5439600	917.5454	0.002	1.6348	SQDG 42:8	C51H82O12S	[M-H]-
918.4823833	918.4833	0.001	0.9799	MIPC 29:1;O5	C41H78NO19P	[M-H]-
918.4823833	918.4833	0.001	0.9799	MIPC 28:1;O3	C40H76NO17P	[M+Formate]-
918.5038641	918.5021	0.002	1.9597	SHexCer 37:2;O6	C43H81NO15S	[M+Cl]-
918.5038641	918.5057	0.002	2.0686	PS 44:10	C50H78NO10P	[M+Cl]-
918.5038641	918.5057	0.002	2.0686	PS O-44:11;O	C50H78NO10P	[M+Cl]-
919.4861332	919.4883	0.002	2.3926	SQDG 39:9	C48H74O12S	[M+Formate]-
919.4861332	919.4883	0.002	2.3926	SQDG 38:9	C47H72O12S	[M+OAc]-
921.2451450	921.2482	0.003	3.2565	PIP2 25:6;O	C34H55O20P3	[M+Formate]-
921.2451450	921.2482	0.003	3.2565	PIP2 24:6;O	C33H53O20P3	[M+OAc]-
921.3844500	921.3808	0.004	3.9072	PIP 32:7	C41H66O16P2	[M+Formate]-
921.3844500	921.3808	0.004	3.9072	PIP 31:7	C40H64O16P2	[M+OAc]-
922.5434806	922.5429	0.001	0.6504	IPC 37:0;O6	C43H86NO15P	[M+Cl]-
923.4454810	923.4459	0.000	0.4332	PIP 32:1	C41H78O16P2	[M+Cl]-
925.4864566	925.4873	0.001	0.8644	PI 42:12	C51H75O13P	[M-H]-
925.4864566	925.4849	0.002	1.7288	PIP 35:3	C44H80O16P2	[M-H]-
927.4053931	927.4043	0.001	1.1861	PIP2 29:0	C38H75O19P3	[M-H]-
927.4053931	927.4066	0.001	1.4018	PIP 36:9	C45H70O16P2	[M-H]-
927.4669767	927.4642	0.003	3.0190	PIP 34:3;O	C43H78O17P2	[M-H]-
	929.4434	0.003	3.2277	PIP 32:3	C41H74O16P2	[M+Formate]-

929.4463983						
929.4463983	929.4434	0.003	3.2277	PIP 31:3	C40H72O16P2	[M+OAc]-
	929.5035	0.004	3.7655	DGDG 33:6	C48H78O15	[M+Cl]-
929.5069870						
929.5069870	929.5105	0.004	3.7654	PG 46:12	C52H79O10P	[M+Cl]-
	929.5033	0.004	3.9806	PI 37:6;O	C46H77O14P	[M+Formate]-
929.5069870						
929.5069870	929.5033	0.004	3.9806	PI 36:6;O	C45H75O14P	[M+OAc]-
	930.4469	0.003	2.9018	MIPC 29:3;O6	C41H74NO20P	[M-H]-
930.4442607						
930.4442607	930.4469	0.003	2.9018	MIPC 28:3;O4	C40H72NO18P	[M+Formate]-
	930.4694	0.004	3.7615	PS 44:12;O	C50H74NO11P	[M+Cl]-
930.4658373						
932.4828286	932.485	0.002	2.3593	PS 44:11;O	C50H76NO11P	[M+Cl]-
	932.5273	0.002	1.7158	IPC 38:2;O6	C44H84NO15P	[M+Cl]-
932.5257074						
933.5037987	933.504	0.000	0.2142	SQDG 40:9	C49H76O12S	[M+Formate]-
	933.504	0.000	0.2142	SQDG 39:9	C48H74O12S	[M+OAc]-
933.5037987						
935.5440094	935.5422	0.002	1.9240	PI 39:4	C48H85O13P	[M+Cl]-
	935.5422	0.002	1.9240	PI O-39:5;O	C48H85O13P	[M+Cl]-
935.5440094						
936.4322637	936.4364	0.004	4.3783	MIPC 31:6;O5	C43H72NO19P	[M-H]-
	936.4364	0.004	4.3783	MIPC 30:6;O3	C42H70NO17P	[M+Formate]-
936.4322637						
936.4322637	936.4364	0.004	4.3783	MIPC 29:6;O3	C41H68NO17P	[M+OAc]-
	937.4276	0.001	1.0667	PI 39:11;O	C48H71O14P	[M+Cl]-
937.4265334						
937.4265334	937.4252	0.001	1.3868	PIP 32:2;O	C41H76O17P2	[M+Cl]-
	938.4439	0.002	1.8115	MIPC 32:6;O2	C44H74NO16P	[M+Cl]-
938.4455883						
939.4665885	939.4642	0.002	2.5546	PIP 35:4;O	C44H78O17P2	[M-H]-
	939.5029	0.000	0.3193	PI 43:12	C52H77O13P	[M-H]-
939.5032099						
940.4640392	940.4677	0.004	3.8279	MIPC 31:4;O5	C43H76NO19P	[M-H]-
	940.4596	0.005	4.7849	MIPC 32:5;O2	C44H76NO16P	[M+Cl]-
940.4640392						
940.4640392	940.4677	0.004	3.8279	MIPC 30:4;O3	C42H74NO17P	[M+Formate]-

	940.4677	0.004	3.8279	MIPC 29:4;O3	C41H72NO17P	[M+OAc]-
940.4640392						
941.3947129	941.399	0.004	4.5677	PIP 34:6	C43H72O16P2	[M+Cl]-
	942.5021	0.001	1.4854	SHexCer 39:4;O6	C45H81NO15S	[M+Cl]-
942.5035409						
942.5035409	942.5057	0.002	2.3342	PS 46:12	C52H78NO10P	[M+Cl]-
	943.4227	0.003	3.3919	PIP 32:4;O	C41H72O17P2	[M+Formate]-
943.4259401						
943.4259401	943.4227	0.003	3.3919	PIP 31:4;O	C40H70O17P2	[M+OAc]-
	943.4591	0.004	4.5577	PIP 33:3	C42H76O16P2	[M+Formate]-
943.4633569						
943.4633569	943.4591	0.004	4.5577	PIP 32:3	C41H74O16P2	[M+OAc]-
	944.6269	0.001	1.3762	SHexCer 42:0;O3	C48H95NO12S	[M+Cl]-
944.6282124						
944.6305962	944.6306	0.000	0.0000	PC O-47:10	C55H92NO7P	[M+Cl]-
	944.6306	0.000	0.0000	PE O-50:10	C55H92NO7P	[M+Cl]-
944.6305962						
946.4367874	946.4338	0.003	3.1698	MIPC 30:4;O4	C42H74NO18P	[M+Cl]-
	948.4575	0.003	2.7413	MIPC 28:2;O5	C40H74NO19P	[M+Formate]-
948.4548327						
948.4762999	948.4727	0.004	3.7956	MIPC 33:6;O4	C45H76NO18P	[M-H]-
	948.4727	0.004	3.7956	MIPC 32:6;O2	C44H74NO16P	[M+Formate]-
948.4762999						
948.4762999	948.4727	0.004	3.7956	MIPC 31:6;O2	C43H72NO16P	[M+OAc]-
	949.4252	0.001	0.6320	PIP 33:3;O	C42H76O17P2	[M+Cl]-
949.4257887						
950.4244457	950.4287	0.004	4.4191	MIPC 29:3;O5	C41H74NO19P	[M+Cl]-
	950.5378	0.003	2.9457	MIPC 32:0;O2	C44H86NO16P	[M+Cl]-
950.5350009						
950.5350009	950.533	0.002	2.1041	Hex2Cer 33:4;O6	C45H79NO17	[M+Formate]-
	950.533	0.002	2.1041	Hex2Cer 32:4;O6	C44H77NO17	[M+OAc]-
950.5350009						
951.5482083	951.5509	0.003	2.8375	SQDG 41:7	C50H82O12S	[M+Formate]-
	951.5509	0.003	2.8375	SQDG 40:7	C49H80O12S	[M+OAc]-
951.5482083						
951.5520890	951.5524	0.000	0.3153	PG 46:9;O	C52H85O11P	[M+Cl]-
	951.5509	0.001	1.2611	SQDG 41:7	C50H82O12S	[M+Formate]-
951.5520890						
951.5520890	951.5509	0.001	1.2611	SQDG 40:7	C49H80O12S	[M+OAc]-

	952.4596	0.003	2.9398	MIPC 33:6;O2	C45H76NO16P	[M+Cl]-
952.4624048						
953.2044895	953.2064	0.002	2.0982	PIP3 23:3	C32H58O22P4	[M+Cl]-
	953.4199	0.004	4.4052	PIP2 31:1	C40H77O19P3	[M-H]-
953.4156886						
953.4552397	953.4565	0.001	1.2586	PIP 33:1;O	C42H80O17P2	[M+Cl]-
	954.4833	0.003	2.7240	MIPC 32:4;O5	C44H78NO19P	[M-H]-
954.4858571						
954.4858571	954.4833	0.003	2.7240	MIPC 31:4;O3	C43H76NO17P	[M+Formate]-
	954.4833	0.003	2.7240	MIPC 30:4;O3	C42H74NO17P	[M+OAc]-
954.4858571						
955.4258405	955.4227	0.003	3.2446	PIP 33:5;O	C42H72O17P2	[M+Formate]-
	955.4227	0.003	3.2446	PIP 32:5;O	C41H70O17P2	[M+OAc]-
955.4258405						
957.4771164	957.4747	0.002	2.5066	PIP 34:3	C43H78O16P2	[M+Formate]-
	957.4747	0.002	2.5066	PIP 33:3	C42H76O16P2	[M+OAc]-
957.4771164						
957.5376383	957.5348	0.003	3.0286	DGDG 35:6	C50H82O15	[M+Cl]-
	957.5346	0.003	3.1330	PI 39:6;O	C48H81O14P	[M+Formate]-
957.5376383						
957.5376383	957.5346	0.003	3.1330	PI 38:6;O	C47H79O14P	[M+OAc]-
	958.4782	0.004	3.7560	MIPC 31:3;O6	C43H78NO20P	[M-H]-
958.4746541						
958.4746541	958.4702	0.005	4.6950	MIPC 32:4;O3	C44H78NO17P	[M+Cl]-
	958.4782	0.004	3.7560	MIPC 30:3;O4	C42H76NO18P	[M+Formate]-
958.4746541						
958.4746541	958.4782	0.004	3.7560	MIPC 29:3;O4	C41H74NO18P	[M+OAc]-
	959.6407	0.003	2.8136	TG 59:16;O2	C62H88O8	[M-H]-
959.6433015						
959.6433015	959.6407	0.003	2.8136	TG 58:16	C61H86O6	[M+Formate]-
	959.6407	0.003	2.8136	TG 57:16	C60H84O6	[M+OAc]-
959.6433015						
962.6420912	962.6422	0.000	0.1039	Hex2Cer 38:2;O5	C50H93NO16	[M-H]-
	962.6411	0.001	1.0388	PC 47:8	C55H94NO8P	[M+Cl]-
962.6420912						
962.6420912	962.6411	0.001	1.0388	PC O-47:9;O	C55H94NO8P	[M+Cl]-
	962.6411	0.001	1.0388	PE 50:8	C55H94NO8P	[M+Cl]-

962.6420912						
962.6420912	962.6411	0.001	1.0388	PE O-50:9;O	C55H94NO8P	[M+Cl]-
	962.6422	0.000	0.1039	Hex2Cer 37:2;O3	C49H91NO14	[M+Formate]-
962.6420912						
962.6420912	962.6422	0.000	0.1039	Hex2Cer 36:2;O3	C48H89NO14	[M+OAc]-
	963.5887	0.002	2.2831	PG 48:9	C54H89O10P	[M+Cl]-
963.5865950						
963.5865950	963.5887	0.002	2.2831	PG O-48:10;O	C54H89O10P	[M+Cl]-
	964.504	0.003	3.5251	MIPC 34:5;O4	C46H80NO18P	[M-H]-
964.5074727						
964.5074727	964.5042	0.003	3.4214	Hex2Cer 35:6;O6	C47H79NO17	[M+Cl]-
	964.504	0.003	3.5251	MIPC 33:5;O2	C45H78NO16P	[M+Formate]-
964.5074727						
964.5074727	964.504	0.003	3.5251	MIPC 32:5;O2	C44H76NO16P	[M+OAc]-
	965.3835	0.001	1.2430	PIP2 31:3;O	C40H73O20P3	[M-H]-
965.3847055						
965.4453494	965.4434	0.002	1.9680	PIP 35:6	C44H74O16P2	[M+Formate]-
	965.4434	0.002	1.9680	PIP 34:6	C43H72O16P2	[M+OAc]-
965.4453494						
966.2140820	966.2128	0.001	1.3455	CoA 10:1;O3	C31H52N7O20P3S	[M-H]-
	966.4236	0.000	0.1035	MIPC 29:3;O6	C41H74NO20P	[M+Cl]-
966.4237273						
966.5243311	966.5197	0.005	4.7593	MIPC 34:4;O4	C46H82NO18P	[M-H]-
	966.5199	0.005	4.6559	Hex2Cer 35:5;O6	C47H81NO17	[M+Cl]-
966.5243311						
966.5243311	966.5197	0.005	4.7593	MIPC 33:4;O2	C45H80NO16P	[M+Formate]-
	966.5197	0.005	4.7593	MIPC 32:4;O2	C44H78NO16P	[M+OAc]-
966.5243311						
968.4351722	968.4392	0.004	4.2336	MIPC 29:2;O6	C41H76NO20P	[M+Cl]-
	969.4148	0.001	0.8252	PIP2 31:1;O	C40H77O20P3	[M-H]-
969.4156771						
969.4156771	969.4172	0.002	1.5473	PIP 38:10;O	C47H72O17P2	[M-H]-
	969.5135	0.001	1.0314	PI 44:12;O	C53H79O14P	[M-H]-
969.5144685						
970.4540039	970.4549	0.001	0.9274	MIPC 29:1;O6	C41H78NO20P	[M+Cl]-
	971.4329	0.001	1.1323	PIP 38:9;O	C47H74O17P2	[M-H]-
971.4339783						
971.4943869	971.4927	0.002	1.6470	PI 42:12	C51H75O13P	[M+Formate]-

973.6174736	973.6175	0.000	0.1027	PI O-46:9	C55H91O12P	[M-H]-
973.6174736	973.6175	0.000	0.1027	PG 48:9	C54H89O10P	[M+Formate]-
973.6174736	973.6175	0.000	0.1027	PG O-48:10;O	C54H89O10P	[M+Formate]-
973.6174736	973.6175	0.000	0.1027	PG 47:9	C53H87O10P	[M+OAc]-
973.6174736	973.6175	0.000	0.1027	PG O-47:10;O	C53H87O10P	[M+OAc]-
974.2151763	974.2179	0.003	2.7715	CoA 12:3;O2	C33H52N7O19P3S	[M-H]-
974.2151763	974.2179	0.003	2.7715	CoA 11:3	C32H50N7O17P3S	[M+Formate]-
974.5059281	974.5095	0.004	3.6942	MIPC 32:2;O6	C44H82NO20P	[M-H]-
974.5059281	974.5015	0.005	4.6177	MIPC 33:3;O3	C45H82NO17P	[M+Cl]-
974.5059281	974.5095	0.004	3.6942	MIPC 31:2;O4	C43H80NO18P	[M+Formate]-
974.5059281	974.5095	0.004	3.6942	MIPC 30:2;O4	C42H78NO18P	[M+OAc]-
975.5533715	975.5524	0.001	1.0251	PG 48:11;O	C54H85O11P	[M+Cl]-
975.6337046	975.6332	0.001	0.5125	PI O-46:8	C55H93O12P	[M-H]-
975.6337046	975.6332	0.001	0.5125	PG 48:8	C54H91O10P	[M+Formate]-
975.6337046	975.6332	0.001	0.5125	PG O-48:9;O	C54H91O10P	[M+Formate]-
975.6337046	975.6332	0.001	0.5125	PG 47:8	C53H89O10P	[M+OAc]-
975.6337046	975.6332	0.001	0.5125	PG O-47:9;O	C53H89O10P	[M+OAc]-
977.4064982	977.407	0.001	0.5116	PIP 35:8;O	C44H70O17P2	[M+Formate]-
977.4064982	977.407	0.001	0.5116	PIP 34:8;O	C43H68O17P2	[M+OAc]-
979.4254604	979.4227	0.003	2.8588	PIP 35:7;O	C44H72O17P2	[M+Formate]-
979.4254604	979.4227	0.003	2.8588	PIP 34:7;O	C43H70O17P2	[M+OAc]-
979.5945547	979.5941	0.001	0.5104	TG 56:16;O3	C59H82O9	[M+Formate]-
983.4453424	983.4459	0.001	0.6101	PIP 37:6	C46H78O16P2	[M+Cl]-
991.5662198	991.5684	0.002	2.2187	PI 42:5;O	C51H89O14P	[M+Cl]-
991.5662198	991.5636	0.003	2.6221	DGDG 37:8	C52H82O15	[M+Formate]-
991.5662198	991.5636	0.003	2.6221	DGDG 36:8	C51H80O15	[M+OAc]-

	995.4329	0.001	0.5023	PIP 40:11;O	C49H74O17P2	[M-H]-
995.4333686						
995.5763207	995.5738	0.003	2.6116	DGDG 41:11	C56H84O15	[M-H]-
	995.5786	0.002	2.3102	PI O-45:9	C54H89O12P	[M+Cl]-
995.5763207						
523.1549555	523.1563	0.001	2.4849	ST 26:7;O4;S	C26H32O7S	[M+Cl]-
	524.2654	0.001	0.9537	ST 22:6;O2;HexNAc	C30H39NO7	[M-H]-
524.2648989						
524.2648989	524.2654	0.001	0.9537	ST 28:7;O6;G	C30H39NO7	[M-H]-
	524.2654	0.001	0.9537	ST 27:7;O4;G	C29H37NO5	[M+Formate]-
524.2648989						
524.2648989	524.2654	0.001	0.9537	ST 26:7;O4;G	C28H35NO5	[M+OAc]-
	524.3147	0.001	0.9536	CerP 28:5;O2	C28H48NO6P	[M-H]-
524.3141182						
524.3141182	524.3147	0.001	0.9536	LPE O-23:6	C28H48NO6P	[M-H]-
	524.3148	0.001	1.3351	CAR 22:5;O	C29H47NO5	[M+Cl]-
524.3141182						
524.3141182	524.3148	0.001	1.3351	Cer 29:6;O4	C29H47NO5	[M+Cl]-
	524.3148	0.001	1.3351	NAE 27:6;O3	C29H47NO5	[M+Cl]-
524.3141182						
524.3141182	524.3148	0.001	1.3351	ST 27:2;O4;G	C29H47NO5	[M+Cl]-
	526.2763	0.000	0.3800	NAT 26:6	C28H45NO4S	[M+Cl]-
526.2765355						
528.2872807	528.2863	0.001	1.8929	LPC 16:1	C24H48NO7P	[M+Cl]-
	528.2863	0.001	1.8929	LPC O-16:2;O	C24H48NO7P	[M+Cl]-
528.2872807						
528.2872807	528.2863	0.001	1.8929	LPE 19:1	C24H48NO7P	[M+Cl]-
	528.2863	0.001	1.8929	LPE O-19:2;O	C24H48NO7P	[M+Cl]-
528.2872807						
528.2872807	528.2886	0.001	2.4608	ST 29:6;O3;G	C31H43NO4	[M+Cl]-
	530.2525	0.001	2.6403	LPS 19:4	C25H42NO9P	[M-H]-
530.2538940						
530.2538940	530.2525	0.001	2.6403	LPS O-19:5;O	C25H42NO9P	[M-H]-
	530.2525	0.001	2.6403	PE 20:4;O	C25H42NO9P	[M-H]-
530.2538940						
530.2538940	530.2526	0.001	2.4517	CAR 19:5;O4	C26H41NO8	[M+Cl]-
	530.2526	0.001	2.4517	ST 18:1;O3;HexNAc	C26H41NO8	[M+Cl]-

530.2538940						
530.2538940	530.2526	0.001	2.4517	ST 24:2;O7;G	C26H41NO8	[M+Cl]-
	530.2525	0.001	2.6403	LPE 19:5	C24H40NO7P	[M+Formate]-
530.2538940						
530.2538940	530.2525	0.001	2.6403	LPE 18:5	C23H38NO7P	[M+OAc]-
	530.2793	0.002	3.0173	NAT 24:4;O4	C26H45NO8S	[M-H]-
530.2776778						
530.2776778	530.2793	0.002	3.0173	ST 24:1;O6;T	C26H45NO8S	[M-H]-
	530.2759	0.002	3.2059	ST 21:4;O3;HexNAc	C29H41NO8	[M-H]-
530.2776778						
530.2776778	530.2759	0.002	3.2059	ST 27:5;O7;G	C29H41NO8	[M-H]-
	530.2793	0.002	3.0173	NAT 23:4;O2	C25H43NO6S	[M+Formate]-
530.2776778						
530.2776778	530.2793	0.002	3.0173	ST 23:1;O4;T	C25H43NO6S	[M+Formate]-
	530.2759	0.002	3.2059	ST 20:4;O;HexNAc	C28H39NO6	[M+Formate]-
530.2776778						
530.2776778	530.2759	0.002	3.2059	ST 26:5;O5;G	C28H39NO6	[M+Formate]-
	530.2793	0.002	3.0173	NAT 22:4;O2	C24H41NO6S	[M+OAc]-
530.2776778						
530.2776778	530.2793	0.002	3.0173	ST 22:1;O4;T	C24H41NO6S	[M+OAc]-
	530.2759	0.002	3.2059	ST 19:4;O;HexNAc	C27H37NO6	[M+OAc]-
530.2776778						
530.2776778	530.2759	0.002	3.2059	ST 25:5;O5;G	C27H37NO6	[M+OAc]-
	533.2157	0.000	0.3751	BMP 18:4;O	C24H39O11P	[M-H]-
533.2159435						
533.2159435	533.2159	0.000	0.0000	ST 19:1;O4;GlcA	C25H38O10	[M+Cl]-
	533.2159	0.000	0.0000	ST 19:2;O5;Hex	C25H38O10	[M+Cl]-
533.2159435						
533.2159435	533.2157	0.000	0.3751	PA 20:4;O	C23H37O9P	[M+Formate]-
	533.2942	0.002	4.1253	ST 30:4;O3;S	C30H46O6S	[M-H]-
533.2920146						
533.2920146	533.2942	0.002	4.1253	ST 29:4;O;S	C29H44O4S	[M+Formate]-
	533.2942	0.002	4.1253	ST 28:4;O;S	C28H42O4S	[M+OAc]-
533.2920146						
533.3075573	533.3073	0.000	0.5625	ST 28:0;O2;S	C28H50O5S	[M+Cl]-
	534.2015	0.001	1.3104	ST 20:2;O7;T	C22H35NO9S	[M+Formate]-
534.2022063						
534.2022063	534.2015	0.001	1.3104	ST 19:2;O7;T	C21H33NO9S	[M+OAc]-

534.2468712	534.2474	0.001	0.9359	LPS 18:3;O	C24H42NO10P	[M-H]-
534.2468712	534.2475	0.001	1.3103	ST 23:1;O8;G	C25H41NO9	[M+Cl]-
534.2468712	534.2474	0.001	0.9359	LPC 15:4;O	C23H40NO8P	[M+Formate]-
534.2468712	534.2474	0.001	0.9359	LPE 18:4;O	C23H40NO8P	[M+Formate]-
534.2468712	534.2474	0.001	0.9359	LPS O-17:4	C23H40NO8P	[M+Formate]-
534.2468712	534.2474	0.001	0.9359	LPC 14:4;O	C22H38NO8P	[M+OAc]-
534.2468712	534.2474	0.001	0.9359	LPE 17:4;O	C22H38NO8P	[M+OAc]-
534.2468712	534.2474	0.001	0.9359	LPS O-16:4	C22H38NO8P	[M+OAc]-
535.3054088	535.3065	0.001	2.0549	ST 27:7;O;Hex	C33H44O6	[M-H]-
535.3054088	535.3042	0.001	2.4285	LPG 20:2	C26H49O9P	[M-H]-
535.3054088	535.3042	0.001	2.4285	LPG O-20:3;O	C26H49O9P	[M-H]-
535.3054088	535.3042	0.001	2.4285	PA 23:1;O	C26H49O9P	[M-H]-
535.3054088	535.3042	0.001	2.4285	PG O-20:2	C26H49O9P	[M-H]-
535.3054088	535.3043	0.001	2.0549	ST 27:0;O8	C27H48O8	[M+Cl]-
535.3054088	535.3042	0.001	2.4285	LPA 22:2	C25H47O7P	[M+Formate]-
535.3054088	535.3042	0.001	2.4285	LPA O-22:3;O	C25H47O7P	[M+Formate]-
535.3054088	535.3042	0.001	2.4285	PA O-22:2	C25H47O7P	[M+Formate]-
535.3054088	535.3042	0.001	2.4285	LPA 21:2	C24H45O7P	[M+OAc]-
535.3054088	535.3042	0.001	2.4285	LPA O-21:3;O	C24H45O7P	[M+OAc]-
535.3054088	535.3042	0.001	2.4285	PA O-21:2	C24H45O7P	[M+OAc]-
535.3552911	535.356	0.001	1.3075	DG O-29:6	C32H52O4	[M+Cl]-
535.3552911	535.356	0.001	1.3075	FA 32:6;O2	C32H52O4	[M+Cl]-
535.3552911	535.356	0.001	1.3075	MG 29:6	C32H52O4	[M+Cl]-
535.3552911	535.356	0.001	1.3075	MG O-29:7;O	C32H52O4	[M+Cl]-
536.2937434	536.2913	0.002	4.4752	LPC O-18:4	C26H48NO6P	[M+Cl]-
536.2937434	536.2913	0.002	4.4752	LPE O-21:4	C26H48NO6P	[M+Cl]-

	536.3018	0.000	0.3729	ST 24:6;O;HexNAc	C32H43NO6	[M-H]-
536.3015330						
536.3015330	536.3018	0.000	0.3729	ST 30:7;O5;G	C32H43NO6	[M-H]-
	536.3018	0.000	0.3729	ST 29:7;O3;G	C31H41NO4	[M+Formate]-
536.3015330						
536.3015330	536.3018	0.000	0.3729	ST 28:7;O3;G	C30H39NO4	[M+OAc]-
	537.2754	0.002	3.5364	LPA 23:3	C26H47O7P	[M+Cl]-
537.2735003						
537.2735003	537.2754	0.002	3.5364	LPA O-23:4;O	C26H47O7P	[M+Cl]-
	537.2754	0.002	3.5364	PA O-23:3	C26H47O7P	[M+Cl]-
537.2735003						
537.2844996	537.2834	0.001	2.0473	BMP 19:1	C25H47O10P	[M-H]-
	537.2834	0.001	2.0473	LPG 19:2;O	C25H47O10P	[M-H]-
537.2844996						
537.2844996	537.2858	0.001	2.4196	ST 26:6;O;GlcA	C32H42O7	[M-H]-
	537.2858	0.001	2.4196	ST 26:7;O2;Hex	C32H42O7	[M-H]-
537.2844996						
537.2844996	537.2834	0.001	2.0473	LPA 21:2;O	C24H45O8P	[M+Formate]-
	537.2834	0.001	2.0473	LPG O-18:3	C24H45O8P	[M+Formate]-
537.2844996						
537.2844996	537.2834	0.001	2.0473	PA 21:1	C24H45O8P	[M+Formate]-
	537.2834	0.001	2.0473	PA O-21:2;O	C24H45O8P	[M+Formate]-
537.2844996						
537.2844996	537.2834	0.001	2.0473	LPA 20:2;O	C23H43O8P	[M+OAc]-
	537.2834	0.001	2.0473	LPG O-17:3	C23H43O8P	[M+OAc]-
537.2844996						
537.2844996	537.2834	0.001	2.0473	PA 20:1	C23H43O8P	[M+OAc]-
	537.2834	0.001	2.0473	PA O-20:2;O	C23H43O8P	[M+OAc]-
537.2844996						
537.3088246	537.3069	0.002	3.5362	ST 23:1;O3;GlcA	C29H46O9	[M-H]-
	537.3069	0.002	3.5362	ST 23:2;O4;Hex	C29H46O9	[M-H]-
537.3088246						
537.3088246	537.3069	0.002	3.5362	DG 25:5;O2	C28H44O7	[M+Formate]-
	537.3069	0.002	3.5362	ST 22:1;O;GlcA	C28H44O7	[M+Formate]-
537.3088246						
537.3088246	537.3069	0.002	3.5362	ST 22:2;O2;Hex	C28H44O7	[M+Formate]-
	537.3069	0.002	3.5362	ST 28:3;O7	C28H44O7	[M+Formate]-

537.3088246						
537.3088246	537.3069	0.002	3.5362	DG 24:5;O2	C27H42O7	[M+OAc]-
	537.3069	0.002	3.5362	ST 21:1;O;GlcA	C27H42O7	[M+OAc]-
537.3088246						
537.3088246	537.3069	0.002	3.5362	ST 21:2;O2;Hex	C27H42O7	[M+OAc]-
	537.3069	0.002	3.5362	ST 27:3;O7	C27H42O7	[M+OAc]-
537.3088246						
537.3713175	537.3716	0.000	0.5583	DG O-29:5	C32H54O4	[M+Cl]-
	537.3716	0.000	0.5583	FA 32:5;O2	C32H54O4	[M+Cl]-
537.3713175						
537.3713175	537.3716	0.000	0.5583	MG 29:5	C32H54O4	[M+Cl]-
	537.3716	0.000	0.5583	MG O-29:6;O	C32H54O4	[M+Cl]-
537.3713175						
539.3247503	539.3226	0.002	4.0792	ST 23:0;O3;GlcA	C29H48O9	[M-H]-
	539.3226	0.002	4.0792	ST 23:1;O4;Hex	C29H48O9	[M-H]-
539.3247503						
539.3247503	539.3274	0.003	4.8208	LPA O-24:2	C27H53O6P	[M+Cl]-
	539.3226	0.002	4.0792	DG 25:4;O2	C28H46O7	[M+Formate]-
539.3247503						
539.3247503	539.3226	0.002	4.0792	ST 22:0;O;GlcA	C28H46O7	[M+Formate]-
	539.3226	0.002	4.0792	ST 22:1;O2;Hex	C28H46O7	[M+Formate]-
539.3247503						
539.3247503	539.3226	0.002	4.0792	ST 28:2;O7	C28H46O7	[M+Formate]-
	539.3226	0.002	4.0792	DG 24:4;O2	C27H44O7	[M+OAc]-
539.3247503						
539.3247503	539.3226	0.002	4.0792	ST 21:0;O;GlcA	C27H44O7	[M+OAc]-
	539.3226	0.002	4.0792	ST 21:1;O2;Hex	C27H44O7	[M+OAc]-
539.3247503						
539.3247503	539.3226	0.002	4.0792	ST 27:2;O7	C27H44O7	[M+OAc]-
	540.2368	0.000	0.7404	PE 21:6;O	C26H40NO9P	[M-H]-
540.2363719						
540.2363719	540.237	0.001	1.1106	ST 19:3;O3;HexNAc	C27H39NO8	[M+Cl]-
	540.237	0.001	1.1106	ST 25:4;O7;G	C27H39NO8	[M+Cl]-
540.2363719						
540.2854520	540.2863	0.001	1.4807	LPC 17:2	C25H48NO7P	[M+Cl]-
	540.2863	0.001	1.4807	LPC O-17:3;O	C25H48NO7P	[M+Cl]-
540.2854520						
540.2854520	540.2863	0.001	1.4807	LPE 20:2	C25H48NO7P	[M+Cl]-

	540.2863	0.001	1.4807	LPE O-20:3;O	C25H48NO7P	[M+Cl]-
540.2854520						
540.2854520	540.2863	0.001	1.4807	PE O-20:2	C25H48NO7P	[M+Cl]-
	540.2848	0.001	1.2956	NAT 21:1;O4	C23H45NO8S	[M+Formate]-
540.2854520						
540.2854520	540.2848	0.001	1.2956	NAT 20:1;O4	C22H43NO8S	[M+OAc]-
	541.196	0.000	0.1848	ST 21:0;O8;S	C21H36O11S	[M+Formate]-
541.1959776						
541.1959776	541.196	0.000	0.1848	ST 20:0;O8;S	C20H34O11S	[M+OAc]-
	542.2162	0.000	0.3689	ST 18:3;O4;HexNAc	C26H37NO9	[M+Cl]-
542.2164823						
542.2164823	542.2162	0.000	0.3689	ST 24:4;O8;G	C26H37NO9	[M+Cl]-
	543.2576	0.001	2.2089	LPI 14:0	C23H45O12P	[M-H]-
543.2563653						
543.2563653	543.2576	0.001	2.2089	LPI O-14:1;O	C23H45O12P	[M-H]-
	543.2553	0.001	2.0248	ST 28:3;O3;S	C28H44O6S	[M+Cl]-
543.2563653						
543.2563653	543.2576	0.001	2.2089	BMP 16:0	C22H43O10P	[M+Formate]-
	543.2576	0.001	2.2089	LPG 16:1;O	C22H43O10P	[M+Formate]-
543.2563653						
543.2563653	543.2576	0.001	2.2089	BMP 15:0	C21H41O10P	[M+OAc]-
	543.2576	0.001	2.2089	LPG 15:1;O	C21H41O10P	[M+OAc]-
543.2563653						
543.2966148	543.2964	0.000	0.5522	ST 25:4;O2;GlcA	C31H44O8	[M-H]-
	543.2964	0.000	0.5522	ST 25:5;O3;Hex	C31H44O8	[M-H]-
543.2966148						
543.2966148	543.2964	0.000	0.5522	ST 24:5;O;Hex	C30H42O6	[M+Formate]-
	543.2964	0.000	0.5522	ST 30:6;O6	C30H42O6	[M+Formate]-
543.2966148						
543.2966148	543.2964	0.000	0.5522	ST 23:5;O;Hex	C29H40O6	[M+OAc]-
	543.2964	0.000	0.5522	ST 29:6;O6	C29H40O6	[M+OAc]-
543.2966148						
544.1853084	544.1858	0.001	0.9188	ST 21:4;O7;T	C23H33NO9S	[M+Formate]-
	544.1858	0.001	0.9188	ST 20:4;O7;T	C22H31NO9S	[M+OAc]-
544.1853084						
545.2929680	545.2942	0.001	2.3840	ST 30:5;O;S	C30H44O4S	[M+Formate]-
	545.2942	0.001	2.3840	ST 29:5;O;S	C29H42O4S	[M+OAc]-
545.2929680						
546.2957995	546.2968	0.001	1.8305	LPC 16:0;O	C24H50NO8P	[M+Cl]-

	546.2968	0.001	1.8305	LPE 19:0;O	C24H50NO8P	[M+Cl]-
546.2957995						
546.2957995	546.2968	0.001	1.8305	LPS O-18:0	C24H50NO8P	[M+Cl]-
	547.2233	0.001	1.4619	LPA 23:6;O	C26H41O8P	[M+Cl]-
547.2241209						
547.2241209	547.2233	0.001	1.4619	PA 23:5	C26H41O8P	[M+Cl]-
	547.2233	0.001	1.4619	PA O-23:6;O	C26H41O8P	[M+Cl]-
547.2241209						
547.2430862	547.2444	0.001	2.5583	BMP 17:0	C23H45O10P	[M+Cl]-
	547.2444	0.001	2.5583	LPG 17:1;O	C23H45O10P	[M+Cl]-
547.2430862						
548.1933517	548.196	0.003	4.7428	ST 25:7;O7;T	C27H35NO9S	[M-H]-
	549.2106	0.002	3.8237	LPI 15:4	C24H39O12P	[M-H]-
549.2127859						
549.2127859	549.2108	0.002	3.6416	ST 19:1;O5;GlcA	C25H38O11	[M+Cl]-
	549.2108	0.002	3.6416	ST 19:2;O6;Hex	C25H38O11	[M+Cl]-
549.2127859						
549.2127859	549.2106	0.002	3.8237	BMP 17:4	C23H37O10P	[M+Formate]-
	549.2106	0.002	3.8237	BMP 16:4	C22H35O10P	[M+OAc]-
549.2127859						
549.2676775	549.2658	0.002	3.2771	ST 27:1;O4;S	C27H46O7S	[M+Cl]-
	550.2342	0.001	1.0904	LPE 20:5;O	C25H42NO8P	[M+Cl]-
550.2336154						
550.2336154	550.2342	0.001	1.0904	LPS O-19:5	C25H42NO8P	[M+Cl]-
	550.2342	0.001	1.0904	PE 20:4	C25H42NO8P	[M+Cl]-
550.2336154						
550.2336154	550.2342	0.001	1.0904	PE O-20:5;O	C25H42NO8P	[M+Cl]-
	550.2328	0.001	1.4539	ST 21:1;O7;T	C23H39NO9S	[M+Formate]-
550.2336154						
550.2336154	550.2328	0.001	1.4539	ST 20:1;O7;T	C22H37NO9S	[M+OAc]-
	550.2423	0.002	3.9982	LPS 17:3	C23H40NO9P	[M+Formate]-
550.2444849						
550.2444849	550.2423	0.002	3.9982	LPS O-17:4;O	C23H40NO9P	[M+Formate]-
	550.2423	0.002	3.9982	LPS 16:3	C22H38NO9P	[M+OAc]-
550.2444849						
550.2444849	550.2423	0.002	3.9982	LPS O-16:4;O	C22H38NO9P	[M+OAc]-
	552.3331	0.001	1.2674	ST 25:5;O;HexNAc	C33H47NO6	[M-H]-

552.3323298						
552.3323298	552.3331	0.001	1.2674	ST 30:6;O3;G	C32H45NO4	[M+Formate]-
	552.3331	0.001	1.2674	ST 29:6;O3;G	C31H43NO4	[M+OAc]-
552.3323298						
553.3045889	553.3067	0.002	3.7954	LPA 24:2	C27H51O7P	[M+Cl]-
	553.3067	0.002	3.7954	LPA O-24:3;O	C27H51O7P	[M+Cl]-
553.3045889						
553.3045889	553.3067	0.002	3.7954	PA O-24:2	C27H51O7P	[M+Cl]-
	553.3664	0.000	0.0000	LPA O-28:5	C31H55O6P	[M-H]-
553.3663193						
553.3663193	553.3665	0.000	0.3614	DG 29:4	C32H54O5	[M+Cl]-
	553.3665	0.000	0.3614	DG O-29:5;O	C32H54O5	[M+Cl]-
553.3663193						
553.3663193	553.3665	0.000	0.3614	FA 32:5;O3	C32H54O5	[M+Cl]-
	553.3665	0.000	0.3614	MG 29:5;O	C32H54O5	[M+Cl]-
553.3663193						
556.2228960	556.2222	0.001	1.2585	ST 24:4;O8;T	C26H39NO10S	[M-H]-
	556.2222	0.001	1.2585	ST 23:4;O6;T	C25H37NO8S	[M+Formate]-
556.2228960						
556.2228960	556.2222	0.001	1.2585	ST 22:4;O6;T	C24H35NO8S	[M+OAc]-
	556.3176	0.001	1.9773	LPC 18:1	C26H52NO7P	[M+Cl]-
556.3186408						
556.3186408	556.3176	0.001	1.9773	LPC O-18:2;O	C26H52NO7P	[M+Cl]-
	556.3176	0.001	1.9773	LPE 21:1	C26H52NO7P	[M+Cl]-
556.3186408						
556.3186408	556.3176	0.001	1.9773	LPE O-21:2;O	C26H52NO7P	[M+Cl]-
	556.3176	0.001	1.9773	PE O-21:1	C26H52NO7P	[M+Cl]-
556.3186408						
557.3355798	557.3331	0.002	4.3062	MGDG 20:2	C29H50O10	[M-H]-
	557.3331	0.002	4.3062	ST 23:0;O5;Hex	C29H50O10	[M-H]-
557.3355798						
557.3355798	557.338	0.002	4.3062	LPA 24:0	C27H55O7P	[M+Cl]-
	557.338	0.002	4.3062	LPA O-24:1;O	C27H55O7P	[M+Cl]-
557.3355798						
557.3355798	557.338	0.002	4.3062	PA O-24:0	C27H55O7P	[M+Cl]-
	557.3331	0.002	4.3062	ST 22:0;O3;Hex	C28H48O8	[M+Formate]-
557.3355798						
557.3355798	557.3331	0.002	4.3062	ST 28:1;O8	C28H48O8	[M+Formate]-

	557.3331	0.002	4.3062	ST 21:0;O3;Hex	C27H46O8	[M+OAc]-
557.3355798						
557.3355798	557.3331	0.002	4.3062	ST 27:1;O8	C27H46O8	[M+OAc]-
	558.3237	0.001	1.6120	NAT 24:0;O3	C26H53NO7S	[M+Cl]-
558.3227937						
559.2847555	559.2832	0.002	2.8608	ST 26:6;O;Hex	C32H44O6	[M+Cl]-
	559.2866	0.002	3.2184	ST 29:2;O3;S	C29H48O6S	[M+Cl]-
559.2847555						
559.2934766	559.2946	0.001	2.1456	ST 28:1;O6;S	C28H48O9S	[M-H]-
	559.2946	0.001	2.1456	ST 27:1;O4;S	C27H46O7S	[M+Formate]-
559.2934766						
559.2934766	559.2946	0.001	2.1456	ST 26:1;O4;S	C26H44O7S	[M+OAc]-
	559.3042	0.000	0.3576	LPG 22:4	C28H49O9P	[M-H]-
559.3043433						
559.3043433	559.3042	0.000	0.3576	LPG O-22:5;O	C28H49O9P	[M-H]-
	559.3042	0.000	0.3576	PA 25:3;O	C28H49O9P	[M-H]-
559.3043433						
559.3043433	559.3042	0.000	0.3576	PG O-22:4	C28H49O9P	[M-H]-
	559.3043	0.000	0.0000	ST 23:0;O2;GlcA	C29H48O8	[M+Cl]-
559.3043433						
559.3043433	559.3043	0.000	0.0000	ST 23:1;O3;Hex	C29H48O8	[M+Cl]-
	559.3043	0.000	0.0000	ST 29:2;O8	C29H48O8	[M+Cl]-
559.3043433						
559.3043433	559.3042	0.000	0.3576	LPA 24:4	C27H47O7P	[M+Formate]-
	559.3042	0.000	0.3576	LPA O-24:5;O	C27H47O7P	[M+Formate]-
559.3043433						
559.3043433	559.3042	0.000	0.3576	PA O-24:4	C27H47O7P	[M+Formate]-
	559.3042	0.000	0.3576	LPA 23:4	C26H45O7P	[M+OAc]-
559.3043433						
559.3043433	559.3042	0.000	0.3576	LPA O-23:5;O	C26H45O7P	[M+OAc]-
	559.3042	0.000	0.3576	PA O-23:4	C26H45O7P	[M+OAc]-
559.3043433						
559.3313145	559.331	0.000	0.5364	ST 29:0;O5;S	C29H52O8S	[M-H]-
	559.331	0.000	0.5364	ST 28:0;O3;S	C28H50O6S	[M+Formate]-
559.3313145						
559.3313145	559.331	0.000	0.5364	ST 27:0;O3;S	C27H48O6S	[M+OAc]-
	560.2994	0.003	4.9973	LPS 21:3	C27H48NO9P	[M-H]-
560.2965667						
560.2965667	560.2994	0.003	4.9973	LPS O-21:4;O	C27H48NO9P	[M-H]-

	**560.2994**	**0.003**	**4.9973**	**PE 22:3;O**	**C27H48NO9P**	**[M-H]-**
**560.2965667**						
**560.2965667**	**560.2994**	**0.003**	**4.9973**	**PS O-21:3**	**C27H48NO9P**	**[M-H]-**
	**560.2994**	**0.003**	**4.9973**	**LPC 18:4**	**C26H46NO7P**	**[M+Formate]-**
**560.2965667**						
**560.2965667**	**560.2994**	**0.003**	**4.9973**	**LPC O-18:5;O**	**C26H46NO7P**	**[M+Formate]-**
	**560.2994**	**0.003**	**4.9973**	**LPE 21:4**	**C26H46NO7P**	**[M+Formate]-**
**560.2965667**						
**560.2965667**	**560.2994**	**0.003**	**4.9973**	**LPE O-21:5;O**	**C26H46NO7P**	**[M+Formate]-**
	**560.2994**	**0.003**	**4.9973**	**PE O-21:4**	**C26H46NO7P**	**[M+Formate]-**
**560.2965667**						
**560.2965667**	**560.2994**	**0.003**	**4.9973**	**LPC 17:4**	**C25H44NO7P**	**[M+OAc]-**
	**560.2994**	**0.003**	**4.9973**	**LPE 20:4**	**C25H44NO7P**	**[M+OAc]-**
**560.2965667**						
**560.2965667**	**560.2994**	**0.003**	**4.9973**	**LPE O-20:5;O**	**C25H44NO7P**	**[M+OAc]-**
	**560.2994**	**0.003**	**4.9973**	**PE O-20:4**	**C25H44NO7P**	**[M+OAc]-**
**560.2965667**						
**560.2965667**	**560.2994**	**0.003**	**4.9973**	**PC 20:3;O**	**C28H50NO9P**	**[M-CH3]-**
	**560.3358**	**0.001**	**2.1416**	**CerP 28:3;O4**	**C28H52NO8P**	**[M-H]-**
**560.3370295**						
**560.3370295**	**560.3358**	**0.001**	**2.1416**	**LPC 20:3;O**	**C28H52NO8P**	**[M-H]-**
	**560.3358**	**0.001**	**2.1416**	**LPE 23:3;O**	**C28H52NO8P**	**[M-H]-**
**560.3370295**						
**560.3370295**	**560.3358**	**0.001**	**2.1416**	**LPS O-22:3**	**C28H52NO8P**	**[M-H]-**
	**560.3358**	**0.001**	**2.1416**	**PE 23:2**	**C28H52NO8P**	**[M-H]-**
**560.3370295**						
**560.3370295**	**560.3358**	**0.001**	**2.1416**	**PE O-23:3;O**	**C28H52NO8P**	**[M-H]-**
	**560.336**	**0.001**	**1.9631**	**CAR 22:3;O3**	**C29H51NO7**	**[M+Cl]-**
**560.3370295**						
**560.3370295**	**560.336**	**0.001**	**1.9631**	**Cer 29:4;O6**	**C29H51NO7**	**[M+Cl]-**
	**560.336**	**0.001**	**1.9631**	**ST 27:0;O6;G**	**C29H51NO7**	**[M+Cl]-**
**560.3370295**						
**560.3370295**	**560.3358**	**0.001**	**2.1416**	**LPC O-19:4**	**C27H50NO6P**	**[M+Formate]-**
	**560.3358**	**0.001**	**2.1416**	**LPE O-22:4**	**C27H50NO6P**	**[M+Formate]-**
**560.3370295**						
**560.3370295**	**560.3358**	**0.001**	**2.1416**	**LPC O-18:4**	**C26H48NO6P**	**[M+OAc]-**
	**560.3358**	**0.001**	**2.1416**	**LPE O-21:4**	**C26H48NO6P**	**[M+OAc]-**

560.3370295						
560.3370295	560.3358	0.001	2.1416	PC 21:2	C29H54NO8P	[M-CH3]-
	560.3358	0.001	2.1416	PC O-21:3;O	C29H54NO8P	[M-CH3]-
560.3370295						
562.2431586	562.2425	0.001	1.2450	ST 18:1;O5;HexNAc	C26H41NO10	[M+Cl]-
	562.2423	0.001	1.6007	LPS 18:4	C24H40NO9P	[M+Formate]-
562.2431586						
562.2431586	562.2423	0.001	1.6007	LPS O-18:5;O	C24H40NO9P	[M+Formate]-
	562.2423	0.001	1.6007	LPS 17:4	C23H38NO9P	[M+OAc]-
562.2431586						
562.3159277	562.3151	0.001	1.6005	LPS 21:2	C27H50NO9P	[M-H]-
	562.3151	0.001	1.6005	LPS O-21:3;O	C27H50NO9P	[M-H]-
562.3159277						
562.3159277	562.3151	0.001	1.6005	PE 22:2;O	C27H50NO9P	[M-H]-
	562.3151	0.001	1.6005	PS O-21:2	C27H50NO9P	[M-H]-
562.3159277						
562.3159277	562.3152	0.001	1.2449	CAR 21:3;O4	C28H49NO8	[M+Cl]-
	562.3152	0.001	1.2449	ST 26:0;O7;G	C28H49NO8	[M+Cl]-
562.3159277						
562.3159277	562.3151	0.001	1.6005	LPC 18:3	C26H48NO7P	[M+Formate]-
	562.3151	0.001	1.6005	LPC O-18:4;O	C26H48NO7P	[M+Formate]-
562.3159277						
562.3159277	562.3151	0.001	1.6005	LPE 21:3	C26H48NO7P	[M+Formate]-
	562.3151	0.001	1.6005	LPE O-21:4;O	C26H48NO7P	[M+Formate]-
562.3159277						
562.3159277	562.3151	0.001	1.6005	PE O-21:3	C26H48NO7P	[M+Formate]-
	562.3151	0.001	1.6005	LPC 17:3	C25H46NO7P	[M+OAc]-
562.3159277						
562.3159277	562.3151	0.001	1.6005	LPC O-17:4;O	C25H46NO7P	[M+OAc]-
	562.3151	0.001	1.6005	LPE 20:3	C25H46NO7P	[M+OAc]-
562.3159277						
562.3159277	562.3151	0.001	1.6005	LPE O-20:4;O	C25H46NO7P	[M+OAc]-
	562.3151	0.001	1.6005	PE O-20:3	C25H46NO7P	[M+OAc]-
562.3159277						
562.3159277	562.3151	0.001	1.6005	PC 20:2;O	C28H52NO9P	[M-CH3]-
	563.2862	0.002	2.6629	ST 24:3;O4;GlcA	C30H44O10	[M-H]-
563.2846548						
563.2846548	563.2862	0.002	2.6629	ST 24:4;O5;Hex	C30H44O10	[M-H]-

563.2846548	563.2862	0.002	2.6629	ST 23:3;O2;GlcA	C29H42O8	[M+Formate]-
563.2846548	563.2862	0.002	2.6629	ST 23:4;O3;Hex	C29H42O8	[M+Formate]-
563.2846548	563.2862	0.002	2.6629	ST 29:5;O8	C29H42O8	[M+Formate]-
563.2846548	563.2862	0.002	2.6629	ST 22:3;O2;GlcA	C28H40O8	[M+OAc]-
563.2846548	563.2862	0.002	2.6629	ST 22:4;O3;Hex	C28H40O8	[M+OAc]-
563.2846548	563.2862	0.002	2.6629	ST 28:5;O8	C28H40O8	[M+OAc]-
563.3012299	563.3014	0.000	0.3550	ST 28:7;O;GlcA	C34H44O7	[M-H]-
564.2132488	564.2135	0.000	0.3545	LPS 19:5	C25H40NO9P	[M+Cl]-
564.2132488	564.2135	0.000	0.3545	PE 20:5;O	C25H40NO9P	[M+Cl]-
564.3331814	564.3331	0.000	0.1772	ST 26:6;O;HexNAc	C34H47NO6	[M-H]-
564.3331814	564.3331	0.000	0.1772	ST 30:7;O3;G	C32H43NO4	[M+OAc]-
564.3683880	564.3671	0.001	2.3035	CerP 28:1;O4	C28H56NO8P	[M-H]-
564.3683880	564.3671	0.001	2.3035	LPC 20:1;O	C28H56NO8P	[M-H]-
564.3683880	564.3671	0.001	2.3035	LPE 23:1;O	C28H56NO8P	[M-H]-
564.3683880	564.3671	0.001	2.3035	LPS O-22:1	C28H56NO8P	[M-H]-
564.3683880	564.3671	0.001	2.3035	PE 23:0	C28H56NO8P	[M-H]-
564.3683880	564.3671	0.001	2.3035	PE O-23:1;O	C28H56NO8P	[M-H]-
564.3683880	564.3673	0.001	1.9491	CAR 22:1;O3	C29H55NO7	[M+Cl]-
564.3683880	564.3673	0.001	1.9491	Cer 29:2;O6	C29H55NO7	[M+Cl]-
564.3683880	564.3671	0.001	2.3035	LPC O-19:2	C27H54NO6P	[M+Formate]-
564.3683880	564.3671	0.001	2.3035	LPE O-22:2	C27H54NO6P	[M+Formate]-
564.3683880	564.3671	0.001	2.3035	LPC O-18:2	C26H52NO6P	[M+OAc]-
564.3683880	564.3671	0.001	2.3035	LPE O-21:2	C26H52NO6P	[M+OAc]-
564.3683880	564.3671	0.001	2.3035	PC 21:0	C29H58NO8P	[M-CH3]-
564.3683880	564.3671	0.001	2.3035	PC O-21:1;O	C29H58NO8P	[M-CH3]-
565.3155963	565.3147	0.001	1.5920	BMP 21:1	C27H51O10P	[M-H]-

	565.3147	0.001	1.5920	LPG 21:2;O	C27H51O10P	[M-H]-
565.3155963						
565.3155963	565.3147	0.001	1.5920	PG 21:1	C27H51O10P	[M-H]-
	565.3147	0.001	1.5920	PG O-21:2;O	C27H51O10P	[M-H]-
565.3155963						
565.3155963	565.3147	0.001	1.5920	LPA 23:2;O	C26H49O8P	[M+Formate]-
	565.3147	0.001	1.5920	LPG O-20:3	C26H49O8P	[M+Formate]-
565.3155963						
565.3155963	565.3147	0.001	1.5920	PA 23:1	C26H49O8P	[M+Formate]-
	565.3147	0.001	1.5920	PA O-23:2;O	C26H49O8P	[M+Formate]-
565.3155963						
565.3155963	565.3147	0.001	1.5920	LPA 22:2;O	C25H47O8P	[M+OAc]-
	565.3147	0.001	1.5920	LPG O-19:3	C25H47O8P	[M+OAc]-
565.3155963						
565.3155963	565.3147	0.001	1.5920	PA 22:1	C25H47O8P	[M+OAc]-
	565.3147	0.001	1.5920	PA O-22:2;O	C25H47O8P	[M+OAc]-
565.3155963						
566.2044549	566.2066	0.002	3.7089	ST 25:6;O8;T	C27H37NO10S	[M-H]-
	566.2066	0.002	3.7089	ST 24:6;O6;T	C26H35NO8S	[M+Formate]-
566.2044549						
566.2044549	566.2066	0.002	3.7089	ST 23:6;O6;T	C25H33NO8S	[M+OAc]-
	566.3464	0.001	1.9423	LPS 21:0	C27H54NO9P	[M-H]-
566.3474318						
566.3474318	566.3464	0.001	1.9423	LPS O-21:1;O	C27H54NO9P	[M-H]-
	566.3464	0.001	1.9423	PE 22:0;O	C27H54NO9P	[M-H]-
566.3474318						
566.3474318	566.3464	0.001	1.9423	PS O-21:0	C27H54NO9P	[M-H]-
	566.3487	0.001	2.2954	ST 26:5;O;HexNAc	C34H49NO6	[M-H]-
566.3474318						
566.3474318	566.3465	0.001	1.5891	CAR 21:1;O4	C28H53NO8	[M+Cl]-
	566.3464	0.001	1.9423	LPC 18:1	C26H52NO7P	[M+Formate]-
566.3474318						
566.3474318	566.3464	0.001	1.9423	LPC O-18:2;O	C26H52NO7P	[M+Formate]-
	566.3464	0.001	1.9423	LPE 21:1	C26H52NO7P	[M+Formate]-
566.3474318						
566.3474318	566.3464	0.001	1.9423	LPE O-21:2;O	C26H52NO7P	[M+Formate]-
	566.3464	0.001	1.9423	PE O-21:1	C26H52NO7P	[M+Formate]-

566.3474318						
566.3474318	566.3464	0.001	1.9423	LPC 17:1	C25H50NO7P	[M+OAc]-
	566.3464	0.001	1.9423	LPC O-17:2;O	C25H50NO7P	[M+OAc]-
566.3474318						
566.3474318	566.3464	0.001	1.9423	LPE 20:1	C25H50NO7P	[M+OAc]-
	566.3464	0.001	1.9423	LPE O-20:2;O	C25H50NO7P	[M+OAc]-
566.3474318						
566.3474318	566.3464	0.001	1.9423	PE O-20:1	C25H50NO7P	[M+OAc]-
	566.3487	0.001	2.2954	ST 30:6;O3;G	C32H45NO4	[M+OAc]-
566.3474318						
566.3474318	566.3464	0.001	1.9423	PC 20:0;O	C28H56NO9P	[M-CH3]-
	567.2131	0.001	1.0578	BMP 19:4	C25H41O10P	[M+Cl]-
567.2137712						
567.2137712	567.2131	0.001	1.0578	LPG 19:5;O	C25H41O10P	[M+Cl]-
	567.2366	0.001	1.4103	ST 23:4;O3;GlcA	C29H40O9	[M+Cl]-
567.2357957						
567.2357957	567.2366	0.001	1.4103	ST 23:5;O4;Hex	C29H40O9	[M+Cl]-
	567.2811	0.001	2.2916	ST 23:2;O5;GlcA	C29H44O11	[M-H]-
567.2824390						
567.2824390	567.2811	0.001	2.2916	ST 23:3;O6;Hex	C29H44O11	[M-H]-
	567.2811	0.001	2.2916	ST 22:2;O3;GlcA	C28H42O9	[M+Formate]-
567.2824390						
567.2824390	567.2811	0.001	2.2916	ST 22:3;O4;Hex	C28H42O9	[M+Formate]-
	567.2811	0.001	2.2916	ST 21:2;O3;GlcA	C27H40O9	[M+OAc]-
567.2824390						
567.2824390	567.2811	0.001	2.2916	ST 21:3;O4;Hex	C27H40O9	[M+OAc]-
	567.3223	0.001	1.0576	LPA 25:2	C28H53O7P	[M+Cl]-
567.3229253						
567.3229253	567.3223	0.001	1.0576	LPA O-25:3;O	C28H53O7P	[M+Cl]-
	567.3223	0.001	1.0576	PA O-25:2	C28H53O7P	[M+Cl]-
567.3229253						
567.3322523	567.3327	0.001	0.8813	ST 28:5;O;GlcA	C34H48O7	[M-H]-
	567.3327	0.001	0.8813	ST 28:6;O2;Hex	C34H48O7	[M-H]-
567.3322523						
567.3322523	567.3327	0.001	0.8813	TG 31:8;O	C34H48O7	[M-H]-
	568.295	0.001	2.2875	NAT 27:6;O4	C29H47NO8S	[M-H]-
568.2936459						
568.2936459	568.295	0.001	2.2875	ST 27:3;O6;T	C29H47NO8S	[M-H]-

	568.295	0.001	2.2875	NAT 26:6;O2	C28H45NO6S	[M+Formate]-
568.2936459						
568.2936459	568.295	0.001	2.2875	ST 26:3;O4;T	C28H45NO6S	[M+Formate]-
	568.295	0.001	2.2875	NAT 25:6;O2	C27H43NO6S	[M+OAc]-
568.2936459						
568.2936459	568.295	0.001	2.2875	ST 25:3;O4;T	C27H43NO6S	[M+OAc]-
	568.3256	0.001	1.7596	LPS 20:0;O	C26H52NO10P	[M-H]-
568.3265962						
568.3265962	568.3256	0.001	1.7596	PS O-20:0;O	C26H52NO10P	[M-H]-
	568.328	0.001	2.4634	ST 25:5;O2;HexNAc	C33H47NO7	[M-H]-
568.3265962						
568.3265962	568.3256	0.001	1.7596	LPC 17:1;O	C25H50NO8P	[M+Formate]-
	568.3256	0.001	1.7596	LPE 20:1;O	C25H50NO8P	[M+Formate]-
568.3265962						
568.3265962	568.3256	0.001	1.7596	LPS O-19:1	C25H50NO8P	[M+Formate]-
	568.3256	0.001	1.7596	PE 20:0	C25H50NO8P	[M+Formate]-
568.3265962						
568.3265962	568.3256	0.001	1.7596	PE O-20:1;O	C25H50NO8P	[M+Formate]-
	568.328	0.001	2.4634	ST 30:6;O4;G	C32H45NO5	[M+Formate]-
568.3265962						
568.3265962	568.3256	0.001	1.7596	LPC 16:1;O	C24H48NO8P	[M+OAc]-
	568.3256	0.001	1.7596	LPE 19:1;O	C24H48NO8P	[M+OAc]-
568.3265962						
568.3265962	568.3256	0.001	1.7596	LPS O-18:1	C24H48NO8P	[M+OAc]-
	568.328	0.001	2.4634	ST 29:6;O4;G	C31H43NO5	[M+OAc]-
568.3265962						
569.3023042	569.3016	0.001	1.2296	LPA 24:2;O	C27H51O8P	[M+Cl]-
	569.3016	0.001	1.2296	LPG O-21:3	C27H51O8P	[M+Cl]-
569.3023042						
569.3023042	569.3016	0.001	1.2296	PA 24:1	C27H51O8P	[M+Cl]-
	569.3016	0.001	1.2296	PA O-24:2;O	C27H51O8P	[M+Cl]-
569.3023042						
569.3615036	569.3613	0.000	0.3513	LPA 28:4	C31H55O7P	[M-H]-
	569.3613	0.000	0.3513	LPA O-28:5;O	C31H55O7P	[M-H]-
569.3615036						
569.3615036	569.3613	0.000	0.3513	PA O-28:4	C31H55O7P	[M-H]-
	569.3614	0.000	0.1756	DG 29:4;O	C32H54O6	[M+Cl]-
569.3615036						
569.3615036	569.3614	0.000	0.1756	DG O-29:5;O2	C32H54O6	[M+Cl]-

569.3615036	569.3614	0.000	0.1756	FA 32:5;O4	C32H54O6	[M+Cl]-
569.3615036	569.3614	0.000	0.1756	ST 26:1;O;Hex	C32H54O6	[M+Cl]-
570.2243306	570.224	0.000	0.5261	LPS 18:3;O	C24H42NO10P	[M+Cl]-
570.2470244	570.2474	0.000	0.5261	LPS 21:6;O	C27H42NO10P	[M-H]-
570.2470244	570.2474	0.000	0.5261	PS 21:5	C27H42NO10P	[M-H]-
570.2470244	570.2474	0.000	0.5261	PS O-21:6;O	C27H42NO10P	[M-H]-
570.2470244	570.2475	0.001	0.8768	ST 20:3;O4;HexNAc	C28H41NO9	[M+Cl]-
570.2470244	570.2475	0.001	0.8768	ST 26:4;O8;G	C28H41NO9	[M+Cl]-
570.2470244	570.2474	0.000	0.5261	PE 21:6	C26H40NO8P	[M+Formate]-
570.2740296	570.2742	0.000	0.3507	ST 26:3;O7;T	C28H45NO9S	[M-H]-
570.2740296	570.2742	0.000	0.3507	NAT 25:6;O3	C27H43NO7S	[M+Formate]-
570.2740296	570.2742	0.000	0.3507	ST 25:3;O5;T	C27H43NO7S	[M+Formate]-
570.2740296	570.2742	0.000	0.3507	NAT 24:6;O3	C26H41NO7S	[M+OAc]-
570.2740296	570.2742	0.000	0.3507	ST 24:3;O5;T	C26H41NO7S	[M+OAc]-
570.3083298	570.3072	0.001	1.9288	ST 24:5;O3;HexNAc	C32H45NO8	[M-H]-
570.3083298	570.3072	0.001	1.9288	ST 30:6;O7;G	C32H45NO8	[M-H]-
570.3083298	570.3072	0.001	1.9288	ST 23:5;O;HexNAc	C31H43NO6	[M+Formate]-
570.3083298	570.3072	0.001	1.9288	ST 29:6;O5;G	C31H43NO6	[M+Formate]-
570.3083298	570.3072	0.001	1.9288	ST 22:5;O;HexNAc	C30H41NO6	[M+OAc]-
570.3083298	570.3072	0.001	1.9288	ST 28:6;O5;G	C30H41NO6	[M+OAc]-
571.2445261	571.2444	0.000	0.1751	BMP 19:2	C25H45O10P	[M+Cl]-
571.2445261	571.2444	0.000	0.1751	LPG 19:3;O	C25H45O10P	[M+Cl]-
571.2782515	571.276	0.002	3.8510	ST 22:1;O6;GlcA	C28H44O12	[M-H]-
571.2782515	571.276	0.002	3.8510	ST 22:2;O7;Hex	C28H44O12	[M-H]-
	571.2808	0.003	4.5512	LPG 20:2	C26H49O9P	[M+Cl]-

571.2782515						
571.2782515	571.2808	0.003	4.5512	LPG O-20:3;O	C26H49O9P	[M+Cl]-
	571.2808	0.003	4.5512	PA 23:1;O	C26H49O9P	[M+Cl]-
571.2782515						
571.2782515	571.2808	0.003	4.5512	PG O-20:2	C26H49O9P	[M+Cl]-
	571.276	0.002	3.8510	ST 21:1;O4;GlcA	C27H42O10	[M+Formate]-
571.2782515						
571.2782515	571.276	0.002	3.8510	ST 21:2;O5;Hex	C27H42O10	[M+Formate]-
	571.276	0.002	3.8510	ST 20:1;O4;GlcA	C26H40O10	[M+OAc]-
571.2782515						
571.2782515	571.276	0.002	3.8510	ST 20:2;O5;Hex	C26H40O10	[M+OAc]-
	571.2889	0.001	2.2756	LPI 16:0	C25H49O12P	[M-H]-
571.2875518						
571.2875518	571.2889	0.001	2.2756	LPI O-16:1;O	C25H49O12P	[M-H]-
	571.2866	0.001	1.7504	ST 30:3;O3;S	C30H48O6S	[M+Cl]-
571.2875518						
571.2875518	571.2889	0.001	2.2756	BMP 18:0	C24H47O10P	[M+Formate]-
	571.2889	0.001	2.2756	LPG 18:1;O	C24H47O10P	[M+Formate]-
571.2875518						
571.2875518	571.2889	0.001	2.2756	BMP 17:0	C23H45O10P	[M+OAc]-
	571.2889	0.001	2.2756	LPG 17:1;O	C23H45O10P	[M+OAc]-
571.2875518						
571.2910907	571.2913	0.000	0.3501	ST 26:5;O3;GlcA	C32H44O9	[M-H]-
	571.2913	0.000	0.3501	ST 26:6;O4;Hex	C32H44O9	[M-H]-
571.2910907						
571.2910907	571.2913	0.000	0.3501	ST 25:5;O;GlcA	C31H42O7	[M+Formate]-
	571.2913	0.000	0.3501	ST 25:6;O2;Hex	C31H42O7	[M+Formate]-
571.2910907						
571.2910907	571.2913	0.000	0.3501	ST 24:5;O;GlcA	C30H40O7	[M+OAc]-
	571.2913	0.000	0.3501	ST 24:6;O2;Hex	C30H40O7	[M+OAc]-
571.2910907						
571.2910907	571.2913	0.000	0.3501	ST 30:7;O7	C30H40O7	[M+OAc]-
	571.3172	0.002	2.9756	LPA 24:1;O	C27H53O8P	[M+Cl]-
571.3155356						
571.3155356	571.3172	0.002	2.9756	LPG O-21:2	C27H53O8P	[M+Cl]-
	571.3172	0.002	2.9756	PA 24:0	C27H53O8P	[M+Cl]-
571.3155356						
571.3155356	571.3172	0.002	2.9756	PA O-24:1;O	C27H53O8P	[M+Cl]-

572.3025433	572.3029	0.000	0.6989	NAT 24:1;O4	C26H51NO8S	[M+Cl]-
574.3152704	574.3151	0.000	0.3482	CerP 28:4;O5	C28H50NO9P	[M-H]-
574.3152704	574.3151	0.000	0.3482	LPS 22:3	C28H50NO9P	[M-H]-
574.3152704	574.3151	0.000	0.3482	LPS O-22:4;O	C28H50NO9P	[M-H]-
574.3152704	574.3151	0.000	0.3482	PE 23:3;O	C28H50NO9P	[M-H]-
574.3152704	574.3151	0.000	0.3482	PS O-22:3	C28H50NO9P	[M-H]-
574.3152704	574.3152	0.000	0.0000	CAR 22:4;O4	C29H49NO8	[M+Cl]-
574.3152704	574.3152	0.000	0.0000	ST 21:0;O3;HexNAc	C29H49NO8	[M+Cl]-
574.3152704	574.3152	0.000	0.0000	ST 27:1;O7;G	C29H49NO8	[M+Cl]-
574.3152704	574.3151	0.000	0.3482	LPC 19:4	C27H48NO7P	[M+Formate]-
574.3152704	574.3151	0.000	0.3482	LPC O-19:5;O	C27H48NO7P	[M+Formate]-
574.3152704	574.3151	0.000	0.3482	LPE 22:4	C27H48NO7P	[M+Formate]-
574.3152704	574.3151	0.000	0.3482	LPE O-22:5;O	C27H48NO7P	[M+Formate]-
574.3152704	574.3151	0.000	0.3482	PE O-22:4	C27H48NO7P	[M+Formate]-
574.3152704	574.3151	0.000	0.3482	LPC 18:4	C26H46NO7P	[M+OAc]-
574.3152704	574.3151	0.000	0.3482	LPC O-18:5;O	C26H46NO7P	[M+OAc]-
574.3152704	574.3151	0.000	0.3482	LPE 21:4	C26H46NO7P	[M+OAc]-
574.3152704	574.3151	0.000	0.3482	LPE O-21:5;O	C26H46NO7P	[M+OAc]-
574.3152704	574.3151	0.000	0.3482	PE O-21:4	C26H46NO7P	[M+OAc]-
574.3152704	574.3151	0.000	0.3482	PC 21:3;O	C29H52NO9P	[M-CH3]-
575.2459902	575.2451	0.001	1.5646	ST 28:3;O5;S	C28H44O8S	[M+Cl]-
575.2459902	575.2474	0.001	2.4337	LPI 13:0	C22H43O12P	[M+Formate]-
575.2459902	575.2474	0.001	2.4337	LPI O-13:1;O	C22H43O12P	[M+Formate]-
575.2459902	575.2474	0.001	2.4337	LPI 12:0	C21H41O12P	[M+OAc]-
575.2459902	575.2474	0.001	2.4337	LPI O-12:1;O	C21H41O12P	[M+OAc]-
575.3251335	575.3259	0.001	1.3905	ST 29:0;O6;S	C29H52O9S	[M-H]-

	575.3259	0.001	1.3905	ST 28:0;O4;S	C28H50O7S	[M+Formate]-
575.3251335						
575.3251335	575.3259	0.001	1.3905	ST 27:0;O4;S	C27H48O7S	[M+OAc]-
	575.3355	0.001	2.2596	LPG 23:3	C29H53O9P	[M-H]-
575.3367691						
575.3367691	575.3355	0.001	2.2596	LPG O-23:4;O	C29H53O9P	[M-H]-
	575.3355	0.001	2.2596	PA 26:2;O	C29H53O9P	[M-H]-
575.3367691						
575.3367691	575.3355	0.001	2.2596	PG O-23:3	C29H53O9P	[M-H]-
	575.3356	0.001	1.9119	ST 24:0;O3;Hex	C30H52O8	[M+Cl]-
575.3367691						
575.3367691	575.3356	0.001	1.9119	ST 30:1;O8	C30H52O8	[M+Cl]-
	575.3355	0.001	2.2596	LPA 25:3	C28H51O7P	[M+Formate]-
575.3367691						
575.3367691	575.3355	0.001	2.2596	LPA O-25:4;O	C28H51O7P	[M+Formate]-
	575.3355	0.001	2.2596	PA O-25:3	C28H51O7P	[M+Formate]-
575.3367691						
575.3367691	575.3355	0.001	2.2596	LPA 24:3	C27H49O7P	[M+OAc]-
	575.3355	0.001	2.2596	LPA O-24:4;O	C27H49O7P	[M+OAc]-
575.3367691						
575.3367691	575.3355	0.001	2.2596	PA O-24:3	C27H49O7P	[M+OAc]-
	576.2943	0.001	1.0411	LPS 21:3;O	C27H48NO10P	[M-H]-
576.2948790						
576.2948790	576.2943	0.001	1.0411	PS 21:2	C27H48NO10P	[M-H]-
	576.2943	0.001	1.0411	PS O-21:3;O	C27H48NO10P	[M-H]-
576.2948790						
576.2948790	576.2945	0.000	0.6941	ST 20:0;O4;HexNAc	C28H47NO9	[M+Cl]-
	576.2945	0.000	0.6941	ST 26:1;O8;G	C28H47NO9	[M+Cl]-
576.2948790						
576.2948790	576.2943	0.001	1.0411	LPC 18:4;O	C26H46NO8P	[M+Formate]-
	576.2943	0.001	1.0411	LPE 21:4;O	C26H46NO8P	[M+Formate]-
576.2948790						
576.2948790	576.2943	0.001	1.0411	LPS O-20:4	C26H46NO8P	[M+Formate]-
	576.2943	0.001	1.0411	PE 21:3	C26H46NO8P	[M+Formate]-
576.2948790						
576.2948790	576.2943	0.001	1.0411	PE O-21:4;O	C26H46NO8P	[M+Formate]-
	576.2943	0.001	1.0411	LPC 17:4;O	C25H44NO8P	[M+OAc]-

576.2948790						
576.2948790	576.2943	0.001	1.0411	LPE 20:4;O	C25H44NO8P	[M+OAc]-
	576.2943	0.001	1.0411	LPS O-19:4	C25H44NO8P	[M+OAc]-
576.2948790						
576.2948790	576.2943	0.001	1.0411	PE 20:3	C25H44NO8P	[M+OAc]-
	576.2943	0.001	1.0411	PE O-20:4;O	C25H44NO8P	[M+OAc]-
576.2948790						
576.3325487	576.3331	0.001	0.8676	ST 27:7;O;HexNAc	C35H47NO6	[M-H]-
	576.3671	0.001	2.4290	CerP 29:2;O4	C29H56NO8P	[M-H]-
576.3684604						
576.3684604	576.3671	0.001	2.4290	LPC 21:2;O	C29H56NO8P	[M-H]-
	576.3671	0.001	2.4290	LPE 24:2;O	C29H56NO8P	[M-H]-
576.3684604						
576.3684604	576.3671	0.001	2.4290	LPS O-23:2	C29H56NO8P	[M-H]-
	576.3671	0.001	2.4290	PE 24:1	C29H56NO8P	[M-H]-
576.3684604						
576.3684604	576.3671	0.001	2.4290	PE O-24:2;O	C29H56NO8P	[M-H]-
	576.3673	0.001	2.0820	CAR 23:2;O3	C30H55NO7	[M+Cl]-
576.3684604						
576.3684604	576.3673	0.001	2.0820	Cer 30:3;O6	C30H55NO7	[M+Cl]-
	576.3671	0.001	2.4290	CerP 28:2;O2	C28H54NO6P	[M+Formate]-
576.3684604						
576.3684604	576.3671	0.001	2.4290	LPC O-20:3	C28H54NO6P	[M+Formate]-
	576.3671	0.001	2.4290	LPE O-23:3	C28H54NO6P	[M+Formate]-
576.3684604						
576.3684604	576.3671	0.001	2.4290	LPC O-19:3	C27H52NO6P	[M+OAc]-
	576.3671	0.001	2.4290	LPE O-22:3	C27H52NO6P	[M+OAc]-
576.3684604						
576.3684604	576.3671	0.001	2.4290	PC 22:1	C30H58NO8P	[M-CH3]-
	576.3671	0.001	2.4290	PC O-22:2;O	C30H58NO8P	[M-CH3]-
576.3684604						
577.3025515	577.3018	0.001	1.2125	MGDG 22:6	C31H46O10	[M-H]-
	577.3018	0.001	1.2125	ST 25:3;O4;GlcA	C31H46O10	[M-H]-
577.3025515						
577.3025515	577.3018	0.001	1.2125	ST 25:4;O5;Hex	C31H46O10	[M-H]-
	577.3018	0.001	1.2125	ST 24:3;O2;GlcA	C30H44O8	[M+Formate]-
577.3025515						
577.3025515	577.3018	0.001	1.2125	ST 24:4;O3;Hex	C30H44O8	[M+Formate]-

	577.3018	0.001	1.2125	ST 30:5;O8	C30H44O8	[M+Formate]-
577.3025515						
577.3025515	577.3018	0.001	1.2125	ST 23:3;O2;GlcA	C29H42O8	[M+OAc]-
	577.3018	0.001	1.2125	ST 23:4;O3;Hex	C29H42O8	[M+OAc]-
577.3025515						
577.3025515	577.3018	0.001	1.2125	ST 29:5;O8	C29H42O8	[M+OAc]-
	577.3171	0.001	1.5589	ST 29:7;O;GlcA	C35H46O7	[M-H]-
577.3162348						
577.3162348	577.3149	0.001	2.2518	ST 23:0;O4;Hex	C29H50O9	[M+Cl]-
	580.2448	0.001	1.3787	LPS 20:4	C26H44NO9P	[M+Cl]-
580.2440108						
580.2440108	580.2448	0.001	1.3787	LPS O-20:5;O	C26H44NO9P	[M+Cl]-
	580.2448	0.001	1.3787	PE 21:4;O	C26H44NO9P	[M+Cl]-
580.2440108						
580.2440108	580.2448	0.001	1.3787	PS O-20:4	C26H44NO9P	[M+Cl]-
	580.2433	0.001	1.2064	ST 22:1;O8;T	C24H41NO10S	[M+Formate]-
580.2440108						
580.2440108	580.2433	0.001	1.2064	ST 21:1;O8;T	C23H39NO10S	[M+OAc]-
	580.3644	0.001	1.0338	ST 27:5;O;HexNAc	C35H51NO6	[M-H]-
580.3637606						
584.2168357	584.2171	0.000	0.5135	ST 24:5;O7;T	C26H37NO9S	[M+Formate]-
	584.2171	0.000	0.5135	ST 23:5;O7;T	C25H35NO9S	[M+OAc]-
584.2168357						
585.3087715	585.3103	0.002	2.5627	ST 30:2;O6;S	C30H50O9S	[M-H]-
	585.3069	0.002	3.2462	ST 27:5;O3;GlcA	C33H46O9	[M-H]-
585.3087715						
585.3087715	585.3069	0.002	3.2462	ST 27:6;O4;Hex	C33H46O9	[M-H]-
	585.3069	0.002	3.2462	TG 30:8;O3	C33H46O9	[M-H]-
585.3087715						
585.3087715	585.3103	0.002	2.5627	ST 29:2;O4;S	C29H48O7S	[M+Formate]-
	585.3069	0.002	3.2462	ST 26:5;O;GlcA	C32H44O7	[M+Formate]-
585.3087715						
585.3087715	585.3069	0.002	3.2462	ST 26:6;O2;Hex	C32H44O7	[M+Formate]-
	585.3103	0.002	2.5627	ST 28:2;O4;S	C28H46O7S	[M+OAc]-
585.3087715						
585.3087715	585.3069	0.002	3.2462	ST 25:5;O;GlcA	C31H42O7	[M+OAc]-
	585.3069	0.002	3.2462	ST 25:6;O2;Hex	C31H42O7	[M+OAc]-
585.3087715						
585.3439248	585.3433	0.001	1.0250	ST 28:4;O2;GlcA	C34H50O8	[M-H]-

585.3439248	585.3433	0.001	1.0250	ST 28:5;O3;Hex	C34H50O8	[M-H]-
585.3439248	585.3433	0.001	1.0250	TG 31:7;O2	C34H50O8	[M-H]-
585.3439248	585.3433	0.001	1.0250	TG O-31:8;O3	C34H50O8	[M-H]-
585.3439248	585.3433	0.001	1.0250	DG 30:8;O	C33H48O6	[M+Formate]-
585.3439248	585.3433	0.001	1.0250	ST 27:5;O;Hex	C33H48O6	[M+Formate]-
585.3439248	585.3433	0.001	1.0250	TG 30:7	C33H48O6	[M+Formate]-
585.3439248	585.3433	0.001	1.0250	TG O-30:8;O	C33H48O6	[M+Formate]-
585.3439248	585.3433	0.001	1.0250	DG 29:8;O	C32H46O6	[M+OAc]-
585.3439248	585.3433	0.001	1.0250	ST 26:5;O;Hex	C32H46O6	[M+OAc]-
586.2661049	586.2658	0.000	0.5117	ST 23:6;O5;HexNAc	C31H41NO10	[M-H]-
586.2661049	586.2658	0.000	0.5117	ST 22:6;O3;HexNAc	C30H39NO8	[M+Formate]-
586.2661049	586.2658	0.000	0.5117	ST 28:7;O7;G	C30H39NO8	[M+Formate]-
586.2661049	586.2658	0.000	0.5117	ST 21:6;O3;HexNAc	C29H37NO8	[M+OAc]-
586.2661049	586.2658	0.000	0.5117	ST 27:7;O7;G	C29H37NO8	[M+OAc]-
587.2364288	587.2345	0.002	3.2355	ST 21:2;O8;GlcA	C27H40O14	[M-H]-
587.2364288	587.2345	0.002	3.2355	ST 20:2;O6;GlcA	C26H38O12	[M+Formate]-
587.2364288	587.2345	0.002	3.2355	ST 20:3;O7;Hex	C26H38O12	[M+Formate]-
587.2364288	587.2345	0.002	3.2355	ST 19:2;O6;GlcA	C25H36O12	[M+OAc]-
587.2364288	587.2345	0.002	3.2355	ST 19:3;O7;Hex	C25H36O12	[M+OAc]-
588.2839213	588.2848	0.001	1.5299	ST 26:2;O8;T	C28H47NO10S	[M-H]-
588.2839213	588.2848	0.001	1.5299	NAT 25:5;O4	C27H45NO8S	[M+Formate]-
588.2839213	588.2848	0.001	1.5299	ST 25:2;O6;T	C27H45NO8S	[M+Formate]-
588.2839213	588.2848	0.001	1.5299	NAT 24:5;O4	C26H43NO8S	[M+OAc]-
588.2839213	588.2848	0.001	1.5299	ST 24:2;O6;T	C26H43NO8S	[M+OAc]-
	589.3416	0.001	1.0181	ST 30:0;O6;S	C30H54O9S	[M-H]-

589.3421990						
589.3421990	589.343	0.001	1.3574	LPA O-28:5	C31H55O6P	[M+Cl]-
	589.3416	0.001	1.0181	ST 29:0;O4;S	C29H52O7S	[M+Formate]-
589.3421990						
589.3421990	589.3416	0.001	1.0181	ST 28:0;O4;S	C28H50O7S	[M+OAc]-
	590.31	0.002	3.5575	CerP 28:4;O6	C28H50NO10P	[M-H]-
590.3120233						
590.3120233	590.31	0.002	3.5575	LPS 22:3;O	C28H50NO10P	[M-H]-
	590.31	0.002	3.5575	PS 22:2	C28H50NO10P	[M-H]-
590.3120233						
590.3120233	590.31	0.002	3.5575	PS O-22:3;O	C28H50NO10P	[M-H]-
	590.3101	0.002	3.2186	ST 21:0;O4;HexNAc	C29H49NO9	[M+Cl]-
590.3120233						
590.3120233	590.3101	0.002	3.2186	ST 27:1;O8;G	C29H49NO9	[M+Cl]-
	590.31	0.002	3.5575	LPC 19:4;O	C27H48NO8P	[M+Formate]-
590.3120233						
590.3120233	590.31	0.002	3.5575	LPE 22:4;O	C27H48NO8P	[M+Formate]-
	590.31	0.002	3.5575	LPS O-21:4	C27H48NO8P	[M+Formate]-
590.3120233						
590.3120233	590.31	0.002	3.5575	PE 22:3	C27H48NO8P	[M+Formate]-
	590.31	0.002	3.5575	PE O-22:4;O	C27H48NO8P	[M+Formate]-
590.3120233						
590.3120233	590.31	0.002	3.5575	LPC 18:4;O	C26H46NO8P	[M+OAc]-
	590.31	0.002	3.5575	LPE 21:4;O	C26H46NO8P	[M+OAc]-
590.3120233						
590.3120233	590.31	0.002	3.5575	LPS O-20:4	C26H46NO8P	[M+OAc]-
	590.31	0.002	3.5575	PE 21:3	C26H46NO8P	[M+OAc]-
590.3120233						
590.3120233	590.31	0.002	3.5575	PE O-21:4;O	C26H46NO8P	[M+OAc]-
	590.3487	0.001	1.6939	ST 28:7;O;HexNAc	C36H49NO6	[M-H]-
590.3477523						
590.3477523	590.3464	0.001	2.3715	CerP 29:3;O5	C29H54NO9P	[M-H]-
	590.3464	0.001	2.3715	LPS 23:2	C29H54NO9P	[M-H]-
590.3477523						
590.3477523	590.3464	0.001	2.3715	LPS O-23:3;O	C29H54NO9P	[M-H]-
	590.3464	0.001	2.3715	PE 24:2;O	C29H54NO9P	[M-H]-
590.3477523						
590.3477523	590.3464	0.001	2.3715	PS O-23:2	C29H54NO9P	[M-H]-

	590.3465	0.001	2.0327	CAR 23:3;O4	C30H53NO8	[M+Cl]-
590.3477523						
590.3477523	590.3465	0.001	2.0327	ST 28:0;O7;G	C30H53NO8	[M+Cl]-
	590.3464	0.001	2.3715	CerP 28:3;O3	C28H52NO7P	[M+Formate]-
590.3477523						
590.3477523	590.3464	0.001	2.3715	LPC 20:3	C28H52NO7P	[M+Formate]-
	590.3464	0.001	2.3715	LPC O-20:4;O	C28H52NO7P	[M+Formate]-
590.3477523						
590.3477523	590.3464	0.001	2.3715	LPE 23:3	C28H52NO7P	[M+Formate]-
	590.3464	0.001	2.3715	LPE O-23:4;O	C28H52NO7P	[M+Formate]-
590.3477523						
590.3477523	590.3464	0.001	2.3715	PC O-20:3	C28H52NO7P	[M+Formate]-
	590.3464	0.001	2.3715	PE O-23:3	C28H52NO7P	[M+Formate]-
590.3477523						
590.3477523	590.3464	0.001	2.3715	LPC 19:3	C27H50NO7P	[M+OAc]-
	590.3464	0.001	2.3715	LPC O-19:4;O	C27H50NO7P	[M+OAc]-
590.3477523						
590.3477523	590.3464	0.001	2.3715	LPE 22:3	C27H50NO7P	[M+OAc]-
	590.3464	0.001	2.3715	LPE O-22:4;O	C27H50NO7P	[M+OAc]-
590.3477523						
590.3477523	590.3464	0.001	2.3715	PE O-22:3	C27H50NO7P	[M+OAc]-
	590.3464	0.001	2.3715	PC 22:2;O	C30H56NO9P	[M-CH3]-
590.3477523						
591.3332242	591.3327	0.001	0.8455	ST 30:7;O;GlcA	C36H48O7	[M-H]-
	592.328	0.001	1.8571	ST 27:7;O2;HexNAc	C35H47NO7	[M-H]-
592.3268394						
592.3268394	592.3256	0.001	2.0259	CerP 28:3;O6	C28H52NO10P	[M-H]-
	592.3256	0.001	2.0259	LPS 22:2;O	C28H52NO10P	[M-H]-
592.3268394						
592.3268394	592.3256	0.001	2.0259	PS 22:1	C28H52NO10P	[M-H]-
	592.3256	0.001	2.0259	PS O-22:2;O	C28H52NO10P	[M-H]-
592.3268394						
592.3268394	592.3258	0.001	1.6883	ST 27:0;O8;G	C29H51NO9	[M+Cl]-
	592.3256	0.001	2.0259	LPC 19:3;O	C27H50NO8P	[M+Formate]-
592.3268394						
592.3268394	592.3256	0.001	2.0259	LPE 22:3;O	C27H50NO8P	[M+Formate]-
	592.3256	0.001	2.0259	LPS O-21:3	C27H50NO8P	[M+Formate]-
592.3268394						
592.3268394	592.3256	0.001	2.0259	PE 22:2	C27H50NO8P	[M+Formate]-

592.3268394	592.3256	0.001	2.0259	PE O-22:3;O	C27H50NO8P	[M+Formate]-
592.3268394	592.3256	0.001	2.0259	LPC 18:3;O	C26H48NO8P	[M+OAc]-
592.3268394	592.3256	0.001	2.0259	LPE 21:3;O	C26H48NO8P	[M+OAc]-
592.3268394	592.3256	0.001	2.0259	LPS O-20:3	C26H48NO8P	[M+OAc]-
592.3268394	592.3256	0.001	2.0259	PE 21:2	C26H48NO8P	[M+OAc]-
592.3268394	592.3256	0.001	2.0259	PE O-21:3;O	C26H48NO8P	[M+OAc]-
593.2158453	593.2159	0.000	0.1686	ST 24:6;O4;GlcA	C30H38O10	[M+Cl]-
594.2024189	594.2015	0.001	1.6829	ST 25:7;O7;T	C27H35NO9S	[M+Formate]-
595.2915462	595.2913	0.000	0.5040	ST 28:7;O3;GlcA	C34H44O9	[M-H]-
595.2915462	595.2913	0.000	0.5040	ST 27:7;O;GlcA	C33H42O7	[M+Formate]-
595.2915462	595.2913	0.000	0.5040	ST 26:7;O;GlcA	C32H40O7	[M+OAc]-
595.3143398	595.3124	0.002	3.1916	ST 25:2;O5;GlcA	C31H48O11	[M-H]-
595.3143398	595.3124	0.002	3.1916	ST 25:3;O6;Hex	C31H48O11	[M-H]-
595.3143398	595.3124	0.002	3.1916	ST 24:2;O3;GlcA	C30H46O9	[M+Formate]-
595.3143398	595.3124	0.002	3.1916	ST 24:3;O4;Hex	C30H46O9	[M+Formate]-
595.3143398	595.3124	0.002	3.1916	ST 23:2;O3;GlcA	C29H44O9	[M+OAc]-
595.3143398	595.3124	0.002	3.1916	ST 23:3;O4;Hex	C29H44O9	[M+OAc]-
596.2266586	596.2268	0.000	0.1677	ST 21:5;O5;HexNAc	C29H39NO10	[M+Cl]-
596.2372636	596.2349	0.002	4.0253	ST 20:4;O8;HexNAc	C28H39NO13	[M-H]-
596.2372636	596.2397	0.002	4.0252	LPS 20:4;O	C26H44NO10P	[M+Cl]-
596.2372636	596.2397	0.002	4.0252	PS 20:3	C26H44NO10P	[M+Cl]-
596.2372636	596.2397	0.002	4.0252	PS O-20:4;O	C26H44NO10P	[M+Cl]-
596.2372636	596.2349	0.002	4.0253	ST 19:4;O6;HexNAc	C27H37NO11	[M+Formate]-
596.2372636	596.2349	0.002	4.0253	ST 18:4;O6;HexNAc	C26H35NO11	[M+OAc]-
	597.232	0.002	4.0185	ST 20:0;O7;GlcA	C26H42O13	[M+Cl]-

597.2343381						
597.2343381	597.232	0.002	4.0185	ST 20:1;O8;Hex	C26H42O13	[M+Cl]-
	597.2318	0.003	4.3534	LPI 15:3	C24H41O12P	[M+Formate]-
597.2343381						
597.2343381	597.2318	0.003	4.3534	LPI O-15:4;O	C24H41O12P	[M+Formate]-
	597.2318	0.003	4.3534	LPI 14:3	C23H39O12P	[M+OAc]-
597.2343381						
597.2343381	597.2318	0.003	4.3534	LPI O-14:4;O	C23H39O12P	[M+OAc]-
	597.2601	0.003	4.3532	BMP 21:3	C27H47O10P	[M+Cl]-
597.2627043						
597.2627043	597.2601	0.003	4.3532	LPG 21:4;O	C27H47O10P	[M+Cl]-
	597.2601	0.003	4.3532	PG 21:3	C27H47O10P	[M+Cl]-
597.2627043						
597.2627043	597.2601	0.003	4.3532	PG O-21:4;O	C27H47O10P	[M+Cl]-
	597.3069	0.000	0.6697	ST 28:6;O3;GlcA	C34H46O9	[M-H]-
597.3065103						
597.3065103	597.3069	0.000	0.6697	ST 28:7;O4;Hex	C34H46O9	[M-H]-
	597.3069	0.000	0.6697	ST 27:6;O;GlcA	C33H44O7	[M+Formate]-
597.3065103						
597.3065103	597.3069	0.000	0.6697	ST 27:7;O2;Hex	C33H44O7	[M+Formate]-
	597.3069	0.000	0.6697	ST 26:6;O;GlcA	C32H42O7	[M+OAc]-
597.3065103						
597.3065103	597.3069	0.000	0.6697	ST 26:7;O2;Hex	C32H42O7	[M+OAc]-
	599.3226	0.000	0.6674	ST 28:5;O3;GlcA	C34H48O9	[M-H]-
599.3222123						
599.3222123	599.3226	0.000	0.6674	ST 28:6;O4;Hex	C34H48O9	[M-H]-
	599.3226	0.000	0.6674	TG 31:8;O3	C34H48O9	[M-H]-
599.3222123						
599.3222123	599.3226	0.000	0.6674	ST 27:5;O;GlcA	C33H46O7	[M+Formate]-
	599.3226	0.000	0.6674	ST 27:6;O2;Hex	C33H46O7	[M+Formate]-
599.3222123						
599.3222123	599.3226	0.000	0.6674	TG 30:8;O	C33H46O7	[M+Formate]-
	599.3226	0.000	0.6674	ST 26:5;O;GlcA	C32H44O7	[M+OAc]-
599.3222123						
599.3222123	599.3226	0.000	0.6674	ST 26:6;O2;Hex	C32H44O7	[M+OAc]-
	600.2848	0.000	0.6664	ST 27:3;O8;T	C29H47NO10S	[M-H]-
600.2843685						
600.2843685	600.2848	0.000	0.6664	NAT 26:6;O4	C28H45NO8S	[M+Formate]-

600.2843685	600.2848	0.000	0.6664	ST 26:3;O6;T	C28H45NO8S	[M+Formate]-
600.2843685	600.2848	0.000	0.6664	NAT 25:6;O4	C27H43NO8S	[M+OAc]-
600.2843685	600.2848	0.000	0.6664	ST 25:3;O6;T	C27H43NO8S	[M+OAc]-
600.3074757	600.3074	0.000	0.1666	LPS 21:1	C27H52NO9P	[M+Cl]-
600.3074757	600.3074	0.000	0.1666	LPS O-21:2;O	C27H52NO9P	[M+Cl]-
600.3074757	600.3074	0.000	0.1666	PE 22:1;O	C27H52NO9P	[M+Cl]-
600.3074757	600.3074	0.000	0.1666	PS O-21:1	C27H52NO9P	[M+Cl]-
601.3159148	601.3147	0.001	1.9956	BMP 24:4	C30H51O10P	[M-H]-
601.3159148	601.3147	0.001	1.9956	LPG 24:5;O	C30H51O10P	[M-H]-
601.3159148	601.3147	0.001	1.9956	PG 24:4	C30H51O10P	[M-H]-
601.3159148	601.3147	0.001	1.9956	PG O-24:5;O	C30H51O10P	[M-H]-
601.3159148	601.3149	0.001	1.6630	ST 25:1;O3;GlcA	C31H50O9	[M+Cl]-
601.3159148	601.3149	0.001	1.6630	ST 25:2;O4;Hex	C31H50O9	[M+Cl]-
601.3159148	601.3147	0.001	1.9956	LPA 26:5;O	C29H49O8P	[M+Formate]-
601.3159148	601.3147	0.001	1.9956	LPG O-23:6	C29H49O8P	[M+Formate]-
601.3159148	601.3147	0.001	1.9956	PA 26:4	C29H49O8P	[M+Formate]-
601.3159148	601.3147	0.001	1.9956	PA O-26:5;O	C29H49O8P	[M+Formate]-
601.3159148	601.3147	0.001	1.9956	LPA 25:5;O	C28H47O8P	[M+OAc]-
601.3159148	601.3147	0.001	1.9956	LPG O-22:6	C28H47O8P	[M+OAc]-
601.3159148	601.3147	0.001	1.9956	PA 25:4	C28H47O8P	[M+OAc]-
601.3159148	601.3147	0.001	1.9956	PA O-25:5;O	C28H47O8P	[M+OAc]-
601.3262785	601.3278	0.002	2.4945	LPG 22:1	C28H55O9P	[M+Cl]-
601.3262785	601.3278	0.002	2.4945	LPG O-22:2;O	C28H55O9P	[M+Cl]-
601.3262785	601.3278	0.002	2.4945	PA 25:0;O	C28H55O9P	[M+Cl]-
601.3262785	601.3278	0.002	2.4945	PG O-22:1	C28H55O9P	[M+Cl]-
601.3423397	601.343	0.001	1.1641	LPA O-29:6	C32H55O6P	[M+Cl]-

601.3423397	601.3416	0.001	1.3304	ST 30:1;O4;S	C30H52O7S	[M+Formate]-
601.3423397	601.3416	0.001	1.3304	ST 29:1;O4;S	C29H50O7S	[M+OAc]-
602.3366850	602.3368	0.000	0.3320	ST 28:1;O7;T	C30H53NO9S	[M-H]-
602.3366850	602.3368	0.000	0.3320	NAT 27:4;O3	C29H51NO7S	[M+Formate]-
602.3366850	602.3368	0.000	0.3320	ST 27:1;O5;T	C29H51NO7S	[M+Formate]-
602.3366850	602.3368	0.000	0.3320	NAT 26:4;O3	C28H49NO7S	[M+OAc]-
602.3366850	602.3368	0.000	0.3320	ST 26:1;O5;T	C28H49NO7S	[M+OAc]-
603.3054887	603.307	0.002	2.6520	BMP 21:0	C27H53O10P	[M+Cl]-
603.3054887	603.307	0.002	2.6520	LPG 21:1;O	C27H53O10P	[M+Cl]-
603.3054887	603.307	0.002	2.6520	PG 21:0	C27H53O10P	[M+Cl]-
603.3054887	603.307	0.002	2.6520	PG O-21:1;O	C27H53O10P	[M+Cl]-
603.3338883	603.3335	0.000	0.6630	EPC 28:5;O2	C30H53N2O6P	[M+Cl]-
603.3684301	603.3691	0.001	1.1602	TG 35:10	C38H52O6	[M-H]-
604.3268780	604.3256	0.001	2.1512	CerP 29:4;O6	C29H52NO10P	[M-H]-
604.3268780	604.3256	0.001	2.1512	LPS 23:3;O	C29H52NO10P	[M-H]-
604.3268780	604.3256	0.001	2.1512	PS 23:2	C29H52NO10P	[M-H]-
604.3268780	604.3256	0.001	2.1512	PS O-23:3;O	C29H52NO10P	[M-H]-
604.3268780	604.3258	0.001	1.8202	ST 22:0;O4;HexNAc	C30H51NO9	[M+Cl]-
604.3268780	604.3258	0.001	1.8202	ST 28:1;O8;G	C30H51NO9	[M+Cl]-
604.3268780	604.3256	0.001	2.1512	CerP 28:4;O4	C28H50NO8P	[M+Formate]-
604.3268780	604.3256	0.001	2.1512	LPC 20:4;O	C28H50NO8P	[M+Formate]-
604.3268780	604.3256	0.001	2.1512	LPE 23:4;O	C28H50NO8P	[M+Formate]-
604.3268780	604.3256	0.001	2.1512	LPS O-22:4	C28H50NO8P	[M+Formate]-
604.3268780	604.3256	0.001	2.1512	PC 20:3	C28H50NO8P	[M+Formate]-
604.3268780	604.3256	0.001	2.1512	PC O-20:4;O	C28H50NO8P	[M+Formate]-

604.3268780						
604.3268780	604.3256	0.001	2.1512	PE 23:3	C28H50NO8P	[M+Formate]-
	604.3256	0.001	2.1512	PE O-23:4;O	C28H50NO8P	[M+Formate]-
604.3268780						
604.3268780	604.3256	0.001	2.1512	LPC 19:4;O	C27H48NO8P	[M+OAc]-
	604.3256	0.001	2.1512	LPE 22:4;O	C27H48NO8P	[M+OAc]-
604.3268780						
604.3268780	604.3256	0.001	2.1512	LPS O-21:4	C27H48NO8P	[M+OAc]-
	604.3256	0.001	2.1512	PE 22:3	C27H48NO8P	[M+OAc]-
604.3268780						
604.3268780	604.3256	0.001	2.1512	PE O-22:4;O	C27H48NO8P	[M+OAc]-
	606.3436	0.001	1.9791	ST 28:7;O2;HexNAc	C36H49NO7	[M-H]-
606.3448764						
607.2431676	607.243	0.000	0.3294	ST 26:2;O8;S	C26H42O11S	[M+Formate]-
	607.243	0.000	0.3294	ST 25:2;O8;S	C25H40O11S	[M+OAc]-
607.2431676						
608.2149909	608.2171	0.002	3.4527	ST 26:7;O7;T	C28H37NO9S	[M+Formate]-
	608.2171	0.002	3.4527	ST 25:7;O7;T	C27H35NO9S	[M+OAc]-
608.2149909						
608.2264717	608.2268	0.000	0.4932	ST 22:6;O5;HexNAc	C30H39NO10	[M+Cl]-
	609.2108	0.002	2.6263	ST 24:6;O5;GlcA	C30H38O11	[M+Cl]-
609.2124473						
609.2124473	609.2142	0.002	2.7905	ST 27:3;O8;S	C27H42O11S	[M+Cl]-
	609.2237	0.000	0.3283	BMP 21:5;O	C27H43O11P	[M+Cl]-
609.2235197						
609.2235197	609.2237	0.000	0.3283	PG 21:5;O	C27H43O11P	[M+Cl]-
	609.2965	0.001	1.4771	LPG 23:4	C29H51O9P	[M+Cl]-
609.2956144						
609.2956144	609.2965	0.001	1.4771	LPG O-23:5;O	C29H51O9P	[M+Cl]-
	609.2965	0.001	1.4771	PA 26:3;O	C29H51O9P	[M+Cl]-
609.2956144						
609.2956144	609.2965	0.001	1.4771	PG O-23:4	C29H51O9P	[M+Cl]-
	609.295	0.001	0.9847	ST 27:0;O7;S	C27H48O10S	[M+Formate]-
609.2956144						
609.2956144	609.295	0.001	0.9847	ST 26:0;O7;S	C26H46O10S	[M+OAc]-
	609.3045	0.001	1.8053	LPI 19:2	C28H51O12P	[M-H]-
609.3056022						
609.3056022	609.3045	0.001	1.8053	LPI O-19:3;O	C28H51O12P	[M-H]-

	609.3069	0.001	2.1336	ST 29:7;O3;GlcA	C35H46O9	[M-H]-
609.3056022						
609.3056022	609.3047	0.001	1.4771	ST 23:0;O6;Hex	C29H50O11	[M+Cl]-
	609.3045	0.001	1.8053	BMP 21:2	C27H49O10P	[M+Formate]-
609.3056022						
609.3056022	609.3045	0.001	1.8053	LPG 21:3;O	C27H49O10P	[M+Formate]-
	609.3045	0.001	1.8053	PG 21:2	C27H49O10P	[M+Formate]-
609.3056022						
609.3056022	609.3045	0.001	1.8053	PG O-21:3;O	C27H49O10P	[M+Formate]-
	609.3069	0.001	2.1336	ST 28:7;O;GlcA	C34H44O7	[M+Formate]-
609.3056022						
609.3056022	609.3045	0.001	1.8053	BMP 20:2	C26H47O10P	[M+OAc]-
	609.3045	0.001	1.8053	LPG 20:3;O	C26H47O10P	[M+OAc]-
609.3056022						
609.3056022	609.3045	0.001	1.8053	PG 20:2	C26H47O10P	[M+OAc]-
	609.3045	0.001	1.8053	PG O-20:3;O	C26H47O10P	[M+OAc]-
609.3056022						
609.3056022	609.3069	0.001	2.1336	ST 27:7;O;GlcA	C33H42O7	[M+OAc]-
	610.3151	0.002	2.9493	LPS 25:6	C31H50NO9P	[M-H]-
610.3168403						
610.3168403	610.3151	0.002	2.9493	LPS O-25:7;O	C31H50NO9P	[M-H]-
	610.3151	0.002	2.9493	PE 26:6;O	C31H50NO9P	[M-H]-
610.3168403						
610.3168403	610.3151	0.002	2.9493	PS O-25:6	C31H50NO9P	[M-H]-
	610.3152	0.002	2.6216	CAR 25:7;O4	C32H49NO8	[M+Cl]-
610.3168403						
610.3168403	610.3152	0.002	2.6216	ST 24:3;O3;HexNAc	C32H49NO8	[M+Cl]-
	610.3152	0.002	2.6216	ST 30:4;O7;G	C32H49NO8	[M+Cl]-
610.3168403						
610.3168403	610.3186	0.002	2.9493	NAT 27:3;O4	C29H53NO8S	[M+Cl]-
	610.3186	0.002	2.9493	ST 27:0;O6;T	C29H53NO8S	[M+Cl]-
610.3168403						
610.3168403	610.3151	0.002	2.9493	LPE 25:7	C30H48NO7P	[M+Formate]-
	610.3151	0.002	2.9493	PE O-25:7	C30H48NO7P	[M+Formate]-
610.3168403						
610.3168403	610.3151	0.002	2.9493	PC 24:6;O	C32H52NO9P	[M-CH3]-
	610.4009	0.002	3.6042	CerP 31:1;O2	C31H62NO6P	[M+Cl]-
610.4030919						
610.4030919	610.4009	0.002	3.6042	LPC O-23:2	C31H62NO6P	[M+Cl]-

	610.4009	0.002	3.6042	LPE O-26:2	C31H62NO6P	[M+Cl]-
610.4030919						
611.2368250	611.2345	0.002	3.7629	ST 23:4;O8;GlcA	C29H40O14	[M-H]-
	611.2394	0.003	4.0901	BMP 21:4;O	C27H45O11P	[M+Cl]-
611.2368250						
611.2368250	611.2394	0.003	4.0901	LPI O-18:5	C27H45O11P	[M+Cl]-
	611.2394	0.003	4.0901	PG 21:4;O	C27H45O11P	[M+Cl]-
611.2368250						
611.2368250	611.2345	0.002	3.7629	ST 22:4;O6;GlcA	C28H38O12	[M+Formate]-
	611.2345	0.002	3.7629	ST 22:5;O7;Hex	C28H38O12	[M+Formate]-
611.2368250						
611.2368250	611.2345	0.002	3.7629	ST 21:4;O6;GlcA	C27H36O12	[M+OAc]-
	611.2345	0.002	3.7629	ST 21:5;O7;Hex	C27H36O12	[M+OAc]-
611.2368250						
611.3231126	611.3226	0.001	0.8179	ST 29:6;O3;GlcA	C35H48O9	[M-H]-
	611.3226	0.001	0.8179	ST 29:7;O4;Hex	C35H48O9	[M-H]-
611.3231126						
611.3231126	611.3226	0.001	0.8179	TG 32:9;O3	C35H48O9	[M-H]-
	611.3226	0.001	0.8179	ST 28:6;O;GlcA	C34H46O7	[M+Formate]-
611.3231126						
611.3231126	611.3226	0.001	0.8179	ST 28:7;O2;Hex	C34H46O7	[M+Formate]-
	611.3226	0.001	0.8179	ST 27:6;O;GlcA	C33H44O7	[M+OAc]-
611.3231126						
611.3231126	611.3226	0.001	0.8179	ST 27:7;O2;Hex	C33H44O7	[M+OAc]-
	613.2057	0.000	0.4892	ST 23:5;O6;GlcA	C29H38O12	[M+Cl]-
613.2059961						
613.2059961	613.2057	0.000	0.4892	ST 23:6;O7;Hex	C29H38O12	[M+Cl]-
	613.2819	0.000	0.6522	ST 28:0;O7;S	C28H50O10S	[M+Cl]-
613.2823199						
613.3023603	613.3018	0.001	0.8153	ST 28:6;O4;GlcA	C34H46O10	[M-H]-
	613.3018	0.001	0.8153	ST 28:7;O5;Hex	C34H46O10	[M-H]-
613.3023603						
613.3023603	613.3018	0.001	0.8153	ST 27:6;O2;GlcA	C33H44O8	[M+Formate]-
	613.3018	0.001	0.8153	ST 27:7;O3;Hex	C33H44O8	[M+Formate]-
613.3023603						
613.3023603	613.3018	0.001	0.8153	ST 26:6;O2;GlcA	C32H42O8	[M+OAc]-
	613.3018	0.001	0.8153	ST 26:7;O3;Hex	C32H42O8	[M+OAc]-

613.3023603						
614.3358007	614.3368	0.001	1.6278	ST 29:2;O7;T	C31H53NO9S	[M-H]-
	614.3368	0.001	1.6278	NAT 28:5;O3	C30H51NO7S	[M+Formate]-
614.3358007						
614.3358007	614.3368	0.001	1.6278	ST 28:2;O5;T	C30H51NO7S	[M+Formate]-
	614.3368	0.001	1.6278	NAT 27:5;O3	C29H49NO7S	[M+OAc]-
614.3358007						
614.3358007	614.3368	0.001	1.6278	ST 27:2;O5;T	C29H49NO7S	[M+OAc]-
	615.2658	0.002	2.6005	ST 23:2;O8;GlcA	C29H44O14	[M-H]-
615.2674414						
615.2674414	615.2658	0.002	2.6005	ST 22:2;O6;GlcA	C28H42O12	[M+Formate]-
	615.2658	0.002	2.6005	ST 22:3;O7;Hex	C28H42O12	[M+Formate]-
615.2674414						
615.2674414	615.2658	0.002	2.6005	ST 21:2;O6;GlcA	C27H40O12	[M+OAc]-
	615.2658	0.002	2.6005	ST 21:3;O7;Hex	C27H40O12	[M+OAc]-
615.2674414						
615.3061695	615.307	0.001	1.4627	BMP 22:1	C28H53O10P	[M+Cl]-
	615.307	0.001	1.4627	LPG 22:2;O	C28H53O10P	[M+Cl]-
615.3061695						
615.3061695	615.307	0.001	1.4627	PG 22:1	C28H53O10P	[M+Cl]-
	615.307	0.001	1.4627	PG O-22:2;O	C28H53O10P	[M+Cl]-
615.3061695						
615.3336633	615.3335	0.000	0.1625	EPC 29:6;O2	C31H53N2O6P	[M+Cl]-
	615.3456	0.001	2.1126	PA O-32:9	C35H53O7P	[M-H]-
615.3443307						
615.3443307	615.3434	0.001	1.4626	LPG 23:1	C29H57O9P	[M+Cl]-
	615.3434	0.001	1.4626	LPG O-23:2;O	C29H57O9P	[M+Cl]-
615.3443307						
615.3443307	615.3434	0.001	1.4626	PA 26:0;O	C29H57O9P	[M+Cl]-
	615.3434	0.001	1.4626	PG O-23:1	C29H57O9P	[M+Cl]-
615.3443307						
615.3443307	615.3458	0.002	2.4377	DG 33:9;O	C36H52O6	[M+Cl]-
	615.3458	0.002	2.4377	ST 30:6;O;Hex	C36H52O6	[M+Cl]-
615.3443307						
615.3443307	615.3458	0.002	2.4377	TG 33:8	C36H52O6	[M+Cl]-
	615.3458	0.002	2.4377	TG O-33:9;O	C36H52O6	[M+Cl]-
615.3443307						
615.3683401	615.3668	0.002	2.6001	LPG 26:4	C32H57O9P	[M-H]-

615.3683401	615.3668	0.002	2.6001	LPG O-26:5;O	C32H57O9P	[M-H]-
615.3683401	615.3668	0.002	2.6001	PA 29:3;O	C32H57O9P	[M-H]-
615.3683401	615.3668	0.002	2.6001	PG O-26:4	C32H57O9P	[M-H]-
615.3683401	615.3669	0.001	2.2751	ST 27:0;O2;GlcA	C33H56O8	[M+Cl]-
615.3683401	615.3669	0.001	2.2751	ST 27:1;O3;Hex	C33H56O8	[M+Cl]-
615.3683401	615.3669	0.001	2.2751	TG 30:3;O2	C33H56O8	[M+Cl]-
615.3683401	615.3669	0.001	2.2751	TG O-30:4;O3	C33H56O8	[M+Cl]-
615.3683401	615.3668	0.002	2.6001	LPA 28:4	C31H55O7P	[M+Formate]-
615.3683401	615.3668	0.002	2.6001	LPA O-28:5;O	C31H55O7P	[M+Formate]-
615.3683401	615.3668	0.002	2.6001	PA O-28:4	C31H55O7P	[M+Formate]-
615.3683401	615.3668	0.002	2.6001	LPA 27:4	C30H53O7P	[M+OAc]-
615.3683401	615.3668	0.002	2.6001	LPA O-27:5;O	C30H53O7P	[M+OAc]-
615.3683401	615.3668	0.002	2.6001	PA O-27:4	C30H53O7P	[M+OAc]-
616.2547597	616.253	0.002	2.7586	ST 21:3;O6;HexNAc	C29H43NO11	[M+Cl]-
616.2547597	616.2528	0.002	3.0832	LPS 21:6;O	C27H42NO10P	[M+Formate]-
616.2547597	616.2528	0.002	3.0832	PS 21:5	C27H42NO10P	[M+Formate]-
616.2547597	616.2528	0.002	3.0832	PS O-21:6;O	C27H42NO10P	[M+Formate]-
616.2547597	616.2528	0.002	3.0832	PS 20:5	C26H40NO10P	[M+OAc]-
616.3159421	616.3161	0.000	0.3245	ST 28:2;O8;T	C30H51NO10S	[M-H]-
616.3159421	616.3161	0.000	0.3245	NAT 27:5;O4	C29H49NO8S	[M+Formate]-
616.3159421	616.3161	0.000	0.3245	ST 27:2;O6;T	C29H49NO8S	[M+Formate]-
616.3159421	616.3161	0.000	0.3245	NAT 26:5;O4	C28H47NO8S	[M+OAc]-
616.3159421	616.3161	0.000	0.3245	ST 26:2;O6;T	C28H47NO8S	[M+OAc]-
617.3223705	617.3227	0.000	0.4860	BMP 22:0	C28H55O10P	[M+Cl]-
617.3223705	617.3227	0.000	0.4860	LPG 22:1;O	C28H55O10P	[M+Cl]-
617.3223705	617.3227	0.000	0.4860	PG 22:0	C28H55O10P	[M+Cl]-

	617.3227	0.000	0.4860	PG O-22:1;O	C28H55O10P	[M+Cl]-
617.3223705						
617.3842645	617.3848	0.001	0.8099	TG 36:10	C39H54O6	[M-H]-
	618.2954	0.000	0.1617	ST 26:2;O7;T	C28H47NO9S	[M+Formate]-
618.2952554						
618.2952554	618.2954	0.000	0.1617	ST 25:2;O7;T	C27H45NO9S	[M+OAc]-
	618.3318	0.000	0.1617	ST 28:1;O8;T	C30H53NO10S	[M-H]-
618.3318200						
618.3318200	618.3318	0.000	0.1617	NAT 27:4;O4	C29H51NO8S	[M+Formate]-
	618.3318	0.000	0.1617	ST 27:1;O6;T	C29H51NO8S	[M+Formate]-
618.3318200						
618.3318200	618.3318	0.000	0.1617	NAT 26:4;O4	C28H49NO8S	[M+OAc]-
	618.3318	0.000	0.1617	ST 26:1;O6;T	C28H49NO8S	[M+OAc]-
618.3318200						
618.4881892	618.4868	0.001	2.2636	CerP 34:0;O2	C34H70NO6P	[M-H]-
	618.4868	0.001	2.2636	LPC O-26:1	C34H70NO6P	[M-H]-
618.4881892						
618.4881892	618.4868	0.001	2.2636	LPE O-29:1	C34H70NO6P	[M-H]-
	618.487	0.001	1.9402	CAR 28:0;O	C35H69NO5	[M+Cl]-
618.4881892						
618.4881892	618.487	0.001	1.9402	Cer 35:1;O4	C35H69NO5	[M+Cl]-
	618.487	0.001	1.9402	NAE 33:1;O3	C35H69NO5	[M+Cl]-
618.4881892						
619.3264189	619.3253	0.001	1.7761	BMP 24:3;O	C30H53O11P	[M-H]-
	619.3253	0.001	1.7761	LPI O-21:4	C30H53O11P	[M-H]-
619.3264189						
619.3264189	619.3253	0.001	1.7761	PG 24:3;O	C30H53O11P	[M-H]-
	619.3255	0.001	1.6147	MGDG 22:3	C31H52O10	[M+Cl]-
619.3264189						
619.3264189	619.3255	0.001	1.6147	ST 25:0;O4;GlcA	C31H52O10	[M+Cl]-
	619.3255	0.001	1.6147	ST 25:1;O5;Hex	C31H52O10	[M+Cl]-
619.3264189						
619.3264189	619.3253	0.001	1.7761	LPG 23:4	C29H51O9P	[M+Formate]-
	619.3253	0.001	1.7761	LPG O-23:5;O	C29H51O9P	[M+Formate]-
619.3264189						
619.3264189	619.3253	0.001	1.7761	PA 26:3;O	C29H51O9P	[M+Formate]-
	619.3253	0.001	1.7761	PG O-23:4	C29H51O9P	[M+Formate]-

619.3264189						
619.3264189	619.3253	0.001	1.7761	LPG 22:4	C28H49O9P	[M+OAc]-
	619.3253	0.001	1.7761	LPG O-22:5;O	C28H49O9P	[M+OAc]-
619.3264189						
619.3264189	619.3253	0.001	1.7761	PA 25:3;O	C28H49O9P	[M+OAc]-
	619.3253	0.001	1.7761	PG O-22:4	C28H49O9P	[M+OAc]-
619.3264189						
620.3122985	620.3125	0.000	0.3224	CerP 30:5;O4	C30H52NO8P	[M+Cl]-
	620.3125	0.000	0.3224	LPC 22:5;O	C30H52NO8P	[M+Cl]-
620.3122985						
620.3122985	620.3125	0.000	0.3224	LPE 25:5;O	C30H52NO8P	[M+Cl]-
	620.3125	0.000	0.3224	LPS O-24:5	C30H52NO8P	[M+Cl]-
620.3122985						
620.3122985	620.3125	0.000	0.3224	PC 22:4	C30H52NO8P	[M+Cl]-
	620.3125	0.000	0.3224	PC O-22:5;O	C30H52NO8P	[M+Cl]-
620.3122985						
620.3122985	620.3125	0.000	0.3224	PE 25:4	C30H52NO8P	[M+Cl]-
	620.3125	0.000	0.3224	PE O-25:5;O	C30H52NO8P	[M+Cl]-
620.3122985						
622.2256641	622.2272	0.002	2.4107	ST 19:2;O8;HexNAc	C27H41NO13	[M+Cl]-
	622.2425	0.001	1.1250	ST 23:6;O5;HexNAc	C31H41NO10	[M+Cl]-
622.2431543						
623.3132060	623.3121	0.001	1.7648	LPG 24:4	C30H53O9P	[M+Cl]-
	623.3121	0.001	1.7648	LPG O-24:5;O	C30H53O9P	[M+Cl]-
623.3132060						
623.3132060	623.3121	0.001	1.7648	PA 27:3;O	C30H53O9P	[M+Cl]-
	623.3121	0.001	1.7648	PG O-24:4	C30H53O9P	[M+Cl]-
623.3132060						
624.2138957	624.212	0.002	3.0438	ST 26:7;O8;T	C28H37NO10S	[M+Formate]-
	624.212	0.002	3.0438	ST 25:7;O8;T	C27H35NO10S	[M+OAc]-
624.2138957						
624.2346827	624.2346	0.000	0.1602	PS 21:4;O	C27H44NO11P	[M+Cl]-
	624.3059	0.000	0.1602	ST 25:0;O8;T	C27H49NO10S	[M+Formate]-
624.3060752						
624.3060752	624.3059	0.000	0.1602	ST 24:0;O8;T	C26H47NO10S	[M+OAc]-
	624.3802	0.002	3.6837	CerP 31:2;O3	C31H60NO7P	[M+Cl]-
624.3824784						
624.3824784	624.3802	0.002	3.6837	LPC 23:2	C31H60NO7P	[M+Cl]-

	624.3802	0.002	3.6837	LPC O-23:3;O	C31H60NO7P	[M+Cl]-
624.3824784						
624.3824784	624.3802	0.002	3.6837	LPE 26:2	C31H60NO7P	[M+Cl]-
	624.3802	0.002	3.6837	LPE O-26:3;O	C31H60NO7P	[M+Cl]-
624.3824784						
624.3824784	624.3802	0.002	3.6837	PC O-23:2	C31H60NO7P	[M+Cl]-
	624.3802	0.002	3.6837	PE O-26:2	C31H60NO7P	[M+Cl]-
624.3824784						
625.2036679	625.2057	0.002	3.3589	ST 24:6;O6;GlcA	C30H38O12	[M+Cl]-
	625.2186	0.002	3.6787	LPI 18:5	C27H43O12P	[M+Cl]-
625.2163021						
625.2163021	625.2138	0.003	3.9986	ST 22:5;O7;GlcA	C28H36O13	[M+Formate]-
	625.2138	0.003	3.9986	ST 22:6;O8;Hex	C28H36O13	[M+Formate]-
625.2163021						
625.2163021	625.2138	0.003	3.9986	ST 21:5;O7;GlcA	C27H34O13	[M+OAc]-
	625.2138	0.003	3.9986	ST 21:6;O8;Hex	C27H34O13	[M+OAc]-
625.2163021						
625.2868647	625.2866	0.000	0.4798	ST 25:3;O7;GlcA	C31H46O13	[M-H]-
	625.2866	0.000	0.4798	ST 25:4;O8;Hex	C31H46O13	[M-H]-
625.2868647						
625.2868647	625.2866	0.000	0.4798	ST 24:3;O5;GlcA	C30H44O11	[M+Formate]-
	625.2866	0.000	0.4798	ST 24:4;O6;Hex	C30H44O11	[M+Formate]-
625.2868647						
625.2868647	625.2866	0.000	0.4798	ST 23:3;O5;GlcA	C29H42O11	[M+OAc]-
	625.2866	0.000	0.4798	ST 23:4;O6;Hex	C29H42O11	[M+OAc]-
625.2868647						
626.2954876	626.2947	0.001	1.2774	PS 20:1;O	C26H48NO11P	[M+Formate]-
	626.3958	0.002	3.8314	CerP 31:1;O3	C31H62NO7P	[M+Cl]-
626.3981997						
626.3981997	626.3958	0.002	3.8314	LPC 23:1	C31H62NO7P	[M+Cl]-
	626.3958	0.002	3.8314	LPC O-23:2;O	C31H62NO7P	[M+Cl]-
626.3981997						
626.3981997	626.3958	0.002	3.8314	LPE 26:1	C31H62NO7P	[M+Cl]-
	626.3958	0.002	3.8314	LPE O-26:2;O	C31H62NO7P	[M+Cl]-
626.3981997						
626.3981997	626.3958	0.002	3.8314	PC O-23:1	C31H62NO7P	[M+Cl]-
	626.3958	0.002	3.8314	PE O-26:1	C31H62NO7P	[M+Cl]-
626.3981997						
627.3432120	627.3434	0.000	0.3188	LPG 24:2	C30H57O9P	[M+Cl]-

627.3432120	627.3434	0.000	0.3188	LPG O-24:3;O	C30H57O9P	[M+Cl]-
627.3432120	627.3434	0.000	0.3188	PA 27:1;O	C30H57O9P	[M+Cl]-
627.3432120	627.3434	0.000	0.3188	PG O-24:2	C30H57O9P	[M+Cl]-
628.4139637	628.4115	0.003	3.9783	CerP 31:0;O3	C31H64NO7P	[M+Cl]-
628.4139637	628.4115	0.003	3.9783	LPC 23:0	C31H64NO7P	[M+Cl]-
628.4139637	628.4115	0.003	3.9783	LPC O-23:1;O	C31H64NO7P	[M+Cl]-
628.4139637	628.4115	0.003	3.9783	LPE 26:0	C31H64NO7P	[M+Cl]-
628.4139637	628.4115	0.003	3.9783	LPE O-26:1;O	C31H64NO7P	[M+Cl]-
628.4139637	628.4115	0.003	3.9783	PC O-23:0	C31H64NO7P	[M+Cl]-
628.4139637	628.4115	0.003	3.9783	PE O-26:0	C31H64NO7P	[M+Cl]-
629.3128848	629.3098	0.003	4.9260	MGDG 23:5	C32H50O10	[M+Cl]-
629.3128848	629.3098	0.003	4.9260	ST 26:2;O4;GlcA	C32H50O10	[M+Cl]-
629.3128848	629.3098	0.003	4.9260	ST 26:3;O5;Hex	C32H50O10	[M+Cl]-
629.3466306	629.346	0.001	0.9534	BMP 26:4	C32H55O10P	[M-H]-
629.3466306	629.346	0.001	0.9534	LPG 26:5;O	C32H55O10P	[M-H]-
629.3466306	629.346	0.001	0.9534	PG 26:4	C32H55O10P	[M-H]-
629.3466306	629.346	0.001	0.9534	PG O-26:5;O	C32H55O10P	[M-H]-
629.3466306	629.3462	0.000	0.6356	ST 27:1;O3;GlcA	C33H54O9	[M+Cl]-
629.3466306	629.3462	0.000	0.6356	ST 27:2;O4;Hex	C33H54O9	[M+Cl]-
629.3466306	629.3462	0.000	0.6356	TG 30:4;O3	C33H54O9	[M+Cl]-
629.3466306	629.346	0.001	0.9534	LPA 28:5;O	C31H53O8P	[M+Formate]-
629.3466306	629.346	0.001	0.9534	LPG O-25:6	C31H53O8P	[M+Formate]-
629.3466306	629.346	0.001	0.9534	PA 28:4	C31H53O8P	[M+Formate]-
629.3466306	629.346	0.001	0.9534	PA O-28:5;O	C31H53O8P	[M+Formate]-
	629.346	0.001	0.9534	LPA 27:5;O	C30H51O8P	[M+OAc]-

629.3466306						
629.3466306	629.346	0.001	0.9534	LPG O-24:6	C30H51O8P	[M+OAc]-
	629.346	0.001	0.9534	PA 27:4	C30H51O8P	[M+OAc]-
629.3466306						
629.3466306	629.346	0.001	0.9534	PA O-27:5;O	C30H51O8P	[M+OAc]-
	631.302	0.001	1.5840	BMP 22:1;O	C28H53O11P	[M+Cl]-
631.3029600						
631.3029600	631.302	0.001	1.5840	LPI O-19:2	C28H53O11P	[M+Cl]-
	631.302	0.001	1.5840	PG 22:1;O	C28H53O11P	[M+Cl]-
631.3029600						
631.3029600	631.3043	0.001	2.2176	ST 29:6;O2;GlcA	C35H48O8	[M+Cl]-
	631.3043	0.001	2.2176	ST 29:7;O3;Hex	C35H48O8	[M+Cl]-
631.3029600						
631.3029600	631.3043	0.001	2.2176	TG 32:9;O2	C35H48O8	[M+Cl]-
	631.3648	0.001	1.4255	EPC 30:5;O2	C32H57N2O6P	[M+Cl]-
631.3639305						
632.3470015	632.3474	0.000	0.6326	ST 29:1;O8;T	C31H55NO10S	[M-H]-
	632.3474	0.000	0.6326	NAT 28:4;O4	C30H53NO8S	[M+Formate]-
632.3470015						
632.3470015	632.3474	0.000	0.6326	ST 28:1;O6;T	C30H53NO8S	[M+Formate]-
	632.3474	0.000	0.6326	NAT 27:4;O4	C29H51NO8S	[M+OAc]-
632.3470015						
632.3470015	632.3474	0.000	0.6326	ST 27:1;O6;T	C29H51NO8S	[M+OAc]-
	632.3569	0.001	2.0558	CerP 31:4;O6	C31H56NO10P	[M-H]-
632.3582450						
632.3582450	632.3569	0.001	2.0558	LPS 25:3;O	C31H56NO10P	[M-H]-
	632.3569	0.001	2.0558	PS 25:2	C31H56NO10P	[M-H]-
632.3582450						
632.3582450	632.3569	0.001	2.0558	PS O-25:3;O	C31H56NO10P	[M-H]-
	632.3571	0.001	1.8977	ST 24:0;O4;HexNAc	C32H55NO9	[M+Cl]-
632.3582450						
632.3582450	632.3571	0.001	1.8977	ST 30:1;O8;G	C32H55NO9	[M+Cl]-
	632.3569	0.001	2.0558	CerP 30:4;O4	C30H54NO8P	[M+Formate]-
632.3582450						
632.3582450	632.3569	0.001	2.0558	LPC 22:4;O	C30H54NO8P	[M+Formate]-
	632.3569	0.001	2.0558	LPE 25:4;O	C30H54NO8P	[M+Formate]-
632.3582450						
632.3582450	632.3569	0.001	2.0558	LPS O-24:4	C30H54NO8P	[M+Formate]-

	632.3569	0.001	2.0558	PC 22:3	C30H54NO8P	[M+Formate]-
632.3582450						
632.3582450	632.3569	0.001	2.0558	PC O-22:4;O	C30H54NO8P	[M+Formate]-
	632.3569	0.001	2.0558	PE 25:3	C30H54NO8P	[M+Formate]-
632.3582450						
632.3582450	632.3569	0.001	2.0558	PE O-25:4;O	C30H54NO8P	[M+Formate]-
	632.3569	0.001	2.0558	CerP 29:4;O4	C29H52NO8P	[M+OAc]-
632.3582450						
632.3582450	632.3569	0.001	2.0558	LPC 21:4;O	C29H52NO8P	[M+OAc]-
	632.3569	0.001	2.0558	LPE 24:4;O	C29H52NO8P	[M+OAc]-
632.3582450						
632.3582450	632.3569	0.001	2.0558	LPS O-23:4	C29H52NO8P	[M+OAc]-
	632.3569	0.001	2.0558	PC 21:3	C29H52NO8P	[M+OAc]-
632.3582450						
632.3582450	632.3569	0.001	2.0558	PC O-21:4;O	C29H52NO8P	[M+OAc]-
	632.3569	0.001	2.0558	PE 24:3	C29H52NO8P	[M+OAc]-
632.3582450						
632.3582450	632.3569	0.001	2.0558	PE O-24:4;O	C29H52NO8P	[M+OAc]-
	633.2965	0.000	0.6316	LPG 25:6	C31H51O9P	[M+Cl]-
633.2960411						
633.2960411	633.2965	0.000	0.6316	LPG O-25:7;O	C31H51O9P	[M+Cl]-
	633.2965	0.000	0.6316	PA 28:5;O	C31H51O9P	[M+Cl]-
633.2960411						
633.2960411	633.2965	0.000	0.6316	PG O-25:6	C31H51O9P	[M+Cl]-
	633.3176	0.002	2.8422	BMP 22:0;O	C28H55O11P	[M+Cl]-
633.3158178						
633.3158178	633.3176	0.002	2.8422	LPI O-19:1	C28H55O11P	[M+Cl]-
	633.3176	0.002	2.8422	PG 22:0;O	C28H55O11P	[M+Cl]-
633.3158178						
634.2925239	634.2917	0.001	1.2612	CerP 30:6;O5	C30H50NO9P	[M+Cl]-
	634.2917	0.001	1.2612	LPS 24:5	C30H50NO9P	[M+Cl]-
634.2925239						
634.2925239	634.2917	0.001	1.2612	LPS O-24:6;O	C30H50NO9P	[M+Cl]-
	634.2917	0.001	1.2612	PC 22:5;O	C30H50NO9P	[M+Cl]-
634.2925239						
634.2925239	634.2917	0.001	1.2612	PE 25:5;O	C30H50NO9P	[M+Cl]-
	634.3055	0.002	3.7837	ST 30:6;O5;T	C32H47NO7S	[M+Formate]-
634.3031504						
634.3031504	634.3055	0.002	3.7837	ST 29:6;O5;T	C31H45NO7S	[M+OAc]-

635.3234493	635.3234	0.000	0.1574	EPC 28:5;O4	C30H53N2O8P	[M+Cl]-
636.3072588	636.3074	0.000	0.1572	CerP 30:5;O5	C30H52NO9P	[M+Cl]-
636.3072588	636.3074	0.000	0.1572	LPS 24:4	C30H52NO9P	[M+Cl]-
636.3072588	636.3074	0.000	0.1572	LPS O-24:5;O	C30H52NO9P	[M+Cl]-
636.3072588	636.3074	0.000	0.1572	PC 22:4;O	C30H52NO9P	[M+Cl]-
636.3072588	636.3074	0.000	0.1572	PE 25:4;O	C30H52NO9P	[M+Cl]-
636.3072588	636.3074	0.000	0.1572	PS O-24:4	C30H52NO9P	[M+Cl]-
637.2270039	637.2269	0.000	0.1569	ST 22:2;O8;GlcA	C28H42O14	[M+Cl]-
637.3270633	637.3263	0.001	1.0983	SQDG 21:1	C30H54O12S	[M-H]-
637.3270633	637.3278	0.001	1.0983	LPG 25:4	C31H55O9P	[M+Cl]-
637.3270633	637.3278	0.001	1.0983	LPG O-25:5;O	C31H55O9P	[M+Cl]-
637.3270633	637.3278	0.001	1.0983	PA 28:3;O	C31H55O9P	[M+Cl]-
637.3270633	637.3278	0.001	1.0983	PG O-25:4	C31H55O9P	[M+Cl]-
637.3270633	637.3263	0.001	1.0983	ST 29:0;O7;S	C29H52O10S	[M+Formate]-
637.3270633	637.3263	0.001	1.0983	ST 28:0;O7;S	C28H50O10S	[M+OAc]-
637.3361564	637.3358	0.000	0.4707	LPI 21:2	C30H55O12P	[M-H]-
637.3361564	637.3358	0.000	0.4707	LPI O-21:3;O	C30H55O12P	[M-H]-
637.3361564	637.3358	0.000	0.4707	PI O-21:2	C30H55O12P	[M-H]-
637.3361564	637.336	0.000	0.1569	ST 25:0;O6;Hex	C31H54O11	[M+Cl]-
637.3361564	637.3358	0.000	0.4707	BMP 23:2	C29H53O10P	[M+Formate]-
637.3361564	637.3358	0.000	0.4707	LPG 23:3;O	C29H53O10P	[M+Formate]-
637.3361564	637.3358	0.000	0.4707	PG 23:2	C29H53O10P	[M+Formate]-
637.3361564	637.3358	0.000	0.4707	PG O-23:3;O	C29H53O10P	[M+Formate]-
637.3361564	637.3358	0.000	0.4707	BMP 22:2	C28H51O10P	[M+OAc]-
	637.3358	0.000	0.4707	LPG 22:3;O	C28H51O10P	[M+OAc]-

637.3361564						
637.3361564	637.3358	0.000	0.4707	PG 22:2	C28H51O10P	[M+OAc]-
	637.3358	0.000	0.4707	PG O-22:3;O	C28H51O10P	[M+OAc]-
637.3361564						
638.2372825	638.2374	0.000	0.1567	ST 23:6;O6;HexNAc	C31H41NO11	[M+Cl]-
	638.2866	0.001	1.7234	CerP 29:5;O6	C29H50NO10P	[M+Cl]-
638.2855216						
638.2855216	638.2866	0.001	1.7234	LPS 23:4;O	C29H50NO10P	[M+Cl]-
	638.2866	0.001	1.7234	PS 23:3	C29H50NO10P	[M+Cl]-
638.2855216						
638.2855216	638.2866	0.001	1.7234	PS O-23:4;O	C29H50NO10P	[M+Cl]-
	638.323	0.000	0.3133	CerP 30:4;O5	C30H54NO9P	[M+Cl]-
638.3232774						
638.3232774	638.323	0.000	0.3133	LPS 24:3	C30H54NO9P	[M+Cl]-
	638.323	0.000	0.3133	LPS O-24:4;O	C30H54NO9P	[M+Cl]-
638.3232774						
638.3232774	638.323	0.000	0.3133	PC 22:3;O	C30H54NO9P	[M+Cl]-
	638.323	0.000	0.3133	PE 25:3;O	C30H54NO9P	[M+Cl]-
638.3232774						
638.3232774	638.323	0.000	0.3133	PS O-24:3	C30H54NO9P	[M+Cl]-
	639.3434	0.000	0.6256	LPG 25:3	C31H57O9P	[M+Cl]-
639.3438357						
639.3438357	639.3434	0.000	0.6256	LPG O-25:4;O	C31H57O9P	[M+Cl]-
	639.3434	0.000	0.6256	PA 28:2;O	C31H57O9P	[M+Cl]-
639.3438357						
639.3438357	639.3434	0.000	0.6256	PG O-25:3	C31H57O9P	[M+Cl]-
	641.2273	0.001	1.7155	ST 29:6;O8;S	C29H40O11S	[M+Formate]-
641.2262809						
641.2262809	641.2273	0.001	1.7155	ST 28:6;O8;S	C28H38O11S	[M+OAc]-
	641.3331	0.000	0.0000	ST 30:6;O4;GlcA	C36H50O10	[M-H]-
641.3331495						
641.3331495	641.3331	0.000	0.0000	ST 30:7;O5;Hex	C36H50O10	[M-H]-
	641.3331	0.000	0.0000	ST 29:6;O2;GlcA	C35H48O8	[M+Formate]-
641.3331495						
641.3331495	641.3331	0.000	0.0000	ST 29:7;O3;Hex	C35H48O8	[M+Formate]-
	641.3331	0.000	0.0000	TG 32:9;O2	C35H48O8	[M+Formate]-
641.3331495						
641.3331495	641.3331	0.000	0.0000	ST 28:6;O2;GlcA	C34H46O8	[M+OAc]-

641.3331495	641.3331	0.000	0.0000	ST 28:7;O3;Hex	C34H46O8	[M+OAc]-
642.2139687	642.2145	0.001	0.9343	ST 28:7;O8;T	C30H41NO10S	[M+Cl]-
642.3056119	642.3049	0.001	1.0898	PS 25:5;O	C31H50NO11P	[M-H]-
642.3056119	642.3051	0.001	0.9341	ST 24:3;O5;HexNAc	C32H49NO10	[M+Cl]-
642.3056119	642.3049	0.001	1.0898	LPS 24:6	C30H48NO9P	[M+Formate]-
642.3056119	642.3049	0.001	1.0898	PC 22:6;O	C30H48NO9P	[M+Formate]-
642.3056119	642.3049	0.001	1.0898	PE 25:6;O	C30H48NO9P	[M+Formate]-
642.3056119	642.3049	0.001	1.0898	PS O-24:6	C30H48NO9P	[M+Formate]-
642.3056119	642.3049	0.001	1.0898	LPS 23:6	C29H46NO9P	[M+OAc]-
642.3056119	642.3049	0.001	1.0898	PC 21:6;O	C29H46NO9P	[M+OAc]-
642.3056119	642.3049	0.001	1.0898	PE 24:6;O	C29H46NO9P	[M+OAc]-
642.3056119	642.3049	0.001	1.0898	PS O-23:6	C29H46NO9P	[M+OAc]-
642.3545094	642.3543	0.000	0.3114	CerP 30:2;O5	C30H58NO9P	[M+Cl]-
642.3545094	642.3543	0.000	0.3114	LPS 24:1	C30H58NO9P	[M+Cl]-
642.3545094	642.3543	0.000	0.3114	LPS O-24:2;O	C30H58NO9P	[M+Cl]-
642.3545094	642.3543	0.000	0.3114	PC 22:1;O	C30H58NO9P	[M+Cl]-
642.3545094	642.3543	0.000	0.3114	PE 25:1;O	C30H58NO9P	[M+Cl]-
642.3545094	642.3543	0.000	0.3114	PS O-24:1	C30H58NO9P	[M+Cl]-
642.3932128	642.3907	0.003	3.8917	CerP 31:1;O4	C31H62NO8P	[M+Cl]-
642.3932128	642.3907	0.003	3.8917	LPC 23:1;O	C31H62NO8P	[M+Cl]-
642.3932128	642.3907	0.003	3.8917	LPE 26:1;O	C31H62NO8P	[M+Cl]-
642.3932128	642.3907	0.003	3.8917	LPS O-25:1	C31H62NO8P	[M+Cl]-
642.3932128	642.3907	0.003	3.8917	PC 23:0	C31H62NO8P	[M+Cl]-
642.3932128	642.3907	0.003	3.8917	PC O-23:1;O	C31H62NO8P	[M+Cl]-
642.3932128	642.3907	0.003	3.8917	PE 26:0	C31H62NO8P	[M+Cl]-
642.3932128	642.3907	0.003	3.8917	PE O-26:1;O	C31H62NO8P	[M+Cl]-

	643.3981	0.001	0.9325	LPG 28:4	C34H61O9P	[M-H]-
643.3974754						
643.3974754	643.3981	0.001	0.9325	LPG O-28:5;O	C34H61O9P	[M-H]-
	643.3981	0.001	0.9325	PA 31:3;O	C34H61O9P	[M-H]-
643.3974754						
643.3974754	643.3981	0.001	0.9325	PG O-28:4	C34H61O9P	[M-H]-
	643.3982	0.001	1.2434	ST 29:0;O2;GlcA	C35H60O8	[M+Cl]-
643.3974754						
643.3974754	643.3982	0.001	1.2434	ST 29:1;O3;Hex	C35H60O8	[M+Cl]-
	643.3982	0.001	1.2434	TG 32:3;O2	C35H60O8	[M+Cl]-
643.3974754						
643.3974754	643.3982	0.001	1.2434	TG O-32:4;O3	C35H60O8	[M+Cl]-
	643.3981	0.001	0.9325	LPA 30:4	C33H59O7P	[M+Formate]-
643.3974754						
643.3974754	643.3981	0.001	0.9325	LPA O-30:5;O	C33H59O7P	[M+Formate]-
	643.3981	0.001	0.9325	PA O-30:4	C33H59O7P	[M+Formate]-
643.3974754						
643.3974754	643.3981	0.001	0.9325	LPA 29:4	C32H57O7P	[M+OAc]-
	643.3981	0.001	0.9325	LPA O-29:5;O	C32H57O7P	[M+OAc]-
643.3974754						
643.3974754	643.3981	0.001	0.9325	PA O-29:4	C32H57O7P	[M+OAc]-
	644.4064	0.002	3.5692	CerP 31:0;O4	C31H64NO8P	[M+Cl]-
644.4086331						
644.4086331	644.4064	0.002	3.5692	LPC 23:0;O	C31H64NO8P	[M+Cl]-
	644.4064	0.002	3.5692	LPE 26:0;O	C31H64NO8P	[M+Cl]-
644.4086331						
644.4086331	644.4064	0.002	3.5692	LPS O-25:0	C31H64NO8P	[M+Cl]-
	644.4064	0.002	3.5692	PC O-23:0;O	C31H64NO8P	[M+Cl]-
644.4086331						
644.4086331	644.4064	0.002	3.5692	PE O-26:0;O	C31H64NO8P	[M+Cl]-
	645.3045	0.002	2.9443	LPI 22:5	C31H51O12P	[M-H]-
645.3064970						
645.3064970	645.3045	0.002	2.9443	LPI O-22:6;O	C31H51O12P	[M-H]-
	645.3045	0.002	2.9443	PI O-22:5	C31H51O12P	[M-H]-
645.3064970						
645.3064970	645.3047	0.002	2.7894	ST 26:2;O5;GlcA	C32H50O11	[M+Cl]-
	645.3047	0.002	2.7894	ST 26:3;O6;Hex	C32H50O11	[M+Cl]-

645.3064970						
645.3064970	645.3045	0.002	2.9443	BMP 24:5	C30H49O10P	[M+Formate]-
645.3064970	645.3045	0.002	2.9443	LPG 24:6;O	C30H49O10P	[M+Formate]-
645.3064970	645.3045	0.002	2.9443	PG 24:5	C30H49O10P	[M+Formate]-
645.3064970	645.3045	0.002	2.9443	PG O-24:6;O	C30H49O10P	[M+Formate]-
645.3064970	645.3045	0.002	2.9443	BMP 23:5	C29H47O10P	[M+OAc]-
645.3064970	645.3045	0.002	2.9443	LPG 23:6;O	C29H47O10P	[M+OAc]-
645.3064970	645.3045	0.002	2.9443	PG 23:5	C29H47O10P	[M+OAc]-
645.3064970	645.3045	0.002	2.9443	PG O-23:6;O	C29H47O10P	[M+OAc]-
645.3430335	645.3441	0.001	1.7045	EPC 30:6;O3	C32H55N2O7P	[M+Cl]-
646.3140406	646.3129	0.001	1.8567	PS 22:0;O	C28H54NO11P	[M+Cl]-
646.3140406	646.3152	0.001	1.8567	ST 27:6;O3;HexNAc	C35H49NO8	[M+Cl]-
646.3473532	646.3492	0.002	2.9396	CerP 29:1;O6	C29H58NO10P	[M+Cl]-
646.3473532	646.3492	0.002	2.9396	LPS 23:0;O	C29H58NO10P	[M+Cl]-
646.3473532	646.3492	0.002	2.9396	PS O-23:0;O	C29H58NO10P	[M+Cl]-
646.3627601	646.3631	0.000	0.4641	ST 30:1;O8;T	C32H57NO10S	[M-H]-
646.3627601	646.3631	0.000	0.4641	NAT 29:4;O4	C31H55NO8S	[M+Formate]-
646.3627601	646.3631	0.000	0.4641	ST 29:1;O6;T	C31H55NO8S	[M+Formate]-
646.3627601	646.3631	0.000	0.4641	NAT 28:4;O4	C30H53NO8S	[M+OAc]-
646.3627601	646.3631	0.000	0.4641	ST 28:1;O6;T	C30H53NO8S	[M+OAc]-
646.3745821	646.3726	0.002	3.0942	CerP 32:4;O6	C32H58NO10P	[M-H]-
646.3745821	646.3726	0.002	3.0942	LPS 26:3;O	C32H58NO10P	[M-H]-
646.3745821	646.3726	0.002	3.0942	PS 26:2	C32H58NO10P	[M-H]-
646.3745821	646.3726	0.002	3.0942	PS O-26:3;O	C32H58NO10P	[M-H]-
646.3745821	646.3727	0.002	2.7848	ST 25:0;O4;HexNAc	C33H57NO9	[M+Cl]-
646.3745821	646.3726	0.002	3.0942	CerP 31:4;O4	C31H56NO8P	[M+Formate]-

	646.3726	0.002	3.0942	LPC 23:4;O	C31H56NO8P	[M+Formate]-
646.3745821						
646.3745821	646.3726	0.002	3.0942	LPE 26:4;O	C31H56NO8P	[M+Formate]-
	646.3726	0.002	3.0942	LPS O-25:4	C31H56NO8P	[M+Formate]-
646.3745821						
646.3745821	646.3726	0.002	3.0942	PC 23:3	C31H56NO8P	[M+Formate]-
	646.3726	0.002	3.0942	PC O-23:4;O	C31H56NO8P	[M+Formate]-
646.3745821						
646.3745821	646.3726	0.002	3.0942	PE 26:3	C31H56NO8P	[M+Formate]-
	646.3726	0.002	3.0942	PE O-26:4;O	C31H56NO8P	[M+Formate]-
646.3745821						
646.3745821	646.3726	0.002	3.0942	CerP 30:4;O4	C30H54NO8P	[M+OAc]-
	646.3726	0.002	3.0942	LPC 22:4;O	C30H54NO8P	[M+OAc]-
646.3745821						
646.3745821	646.3726	0.002	3.0942	LPE 25:4;O	C30H54NO8P	[M+OAc]-
	646.3726	0.002	3.0942	LPS O-24:4	C30H54NO8P	[M+OAc]-
646.3745821						
646.3745821	646.3726	0.002	3.0942	PC 22:3	C30H54NO8P	[M+OAc]-
	646.3726	0.002	3.0942	PC O-22:4;O	C30H54NO8P	[M+OAc]-
646.3745821						
646.3745821	646.3726	0.002	3.0942	PE 25:3	C30H54NO8P	[M+OAc]-
	646.3726	0.002	3.0942	PE O-25:4;O	C30H54NO8P	[M+OAc]-
646.3745821						
647.3138953	647.3121	0.002	2.7807	LPG 26:6	C32H53O9P	[M+Cl]-
	647.3121	0.002	2.7807	LPG O-26:7;O	C32H53O9P	[M+Cl]-
647.3138953						
647.3138953	647.3121	0.002	2.7807	PA 29:5;O	C32H53O9P	[M+Cl]-
	647.3121	0.002	2.7807	PG O-26:6	C32H53O9P	[M+Cl]-
647.3138953						
647.3231861	647.3234	0.000	0.3090	EPC 29:6;O4	C31H53N2O8P	[M+Cl]-
	647.3333	0.001	0.7724	BMP 23:0;O	C29H57O11P	[M+Cl]-
647.3337354						
647.3337354	647.3333	0.001	0.7724	LPI O-20:1	C29H57O11P	[M+Cl]-
	647.3333	0.001	0.7724	PG 23:0;O	C29H57O11P	[M+Cl]-
647.3337354						
648.2451981	648.2429	0.002	3.5481	ST 21:3;O8;HexNAc	C29H43NO13	[M+Cl]-
	648.3237	0.002	2.7764	ST 21:0;O7;HexNAc	C29H49NO12	[M+Formate]-
648.3254932						
648.3254932	648.3237	0.002	2.7764	ST 20:0;O7;HexNAc	C28H47NO12	[M+OAc]-

	648.3438	0.000	0.1542	CerP 32:5;O4	C32H56NO8P	[M+Cl]-
648.3437077						
648.3437077	648.3438	0.000	0.1542	LPC 24:5;O	C32H56NO8P	[M+Cl]-
	648.3438	0.000	0.1542	LPE 27:5;O	C32H56NO8P	[M+Cl]-
648.3437077						
648.3437077	648.3438	0.000	0.1542	LPS O-26:5	C32H56NO8P	[M+Cl]-
	648.3438	0.000	0.1542	PC 24:4	C32H56NO8P	[M+Cl]-
648.3437077						
648.3437077	648.3438	0.000	0.1542	PC O-24:5;O	C32H56NO8P	[M+Cl]-
	648.3438	0.000	0.1542	PE 27:4	C32H56NO8P	[M+Cl]-
648.3437077						
648.3437077	648.3438	0.000	0.1542	PE O-27:5;O	C32H56NO8P	[M+Cl]-
	649.2269	0.001	2.1564	ST 23:3;O8;GlcA	C29H42O14	[M+Cl]-
649.2254668						
649.3368763	649.3358	0.001	1.5400	LPI 22:3	C31H55O12P	[M-H]-
	649.3358	0.001	1.5400	LPI O-22:4;O	C31H55O12P	[M-H]-
649.3368763						
649.3368763	649.3358	0.001	1.5400	PI O-22:3	C31H55O12P	[M-H]-
	649.336	0.001	1.3860	ST 26:0;O5;GlcA	C32H54O11	[M+Cl]-
649.3368763						
649.3368763	649.336	0.001	1.3860	ST 26:1;O6;Hex	C32H54O11	[M+Cl]-
	649.3358	0.001	1.5400	BMP 24:3	C30H53O10P	[M+Formate]-
649.3368763						
649.3368763	649.3358	0.001	1.5400	LPG 24:4;O	C30H53O10P	[M+Formate]-
	649.3358	0.001	1.5400	PG 24:3	C30H53O10P	[M+Formate]-
649.3368763						
649.3368763	649.3358	0.001	1.5400	PG O-24:4;O	C30H53O10P	[M+Formate]-
	649.3358	0.001	1.5400	BMP 23:3	C29H51O10P	[M+OAc]-
649.3368763						
649.3368763	649.3358	0.001	1.5400	LPG 23:4;O	C29H51O10P	[M+OAc]-
	649.3358	0.001	1.5400	PG 23:3	C29H51O10P	[M+OAc]-
649.3368763						
649.3368763	649.3358	0.001	1.5400	PG O-23:4;O	C29H51O10P	[M+OAc]-
	652.3023	0.001	1.0731	CerP 30:5;O6	C30H52NO10P	[M+Cl]-
652.3029971						
652.3029971	652.3023	0.001	1.0731	LPS 24:4;O	C30H52NO10P	[M+Cl]-
	652.3023	0.001	1.0731	PS 24:3	C30H52NO10P	[M+Cl]-

652.3029971						
652.3029971	652.3023	0.001	1.0731	PS O-24:4;O	C30H52NO10P	[M+Cl]-
	654.2323	0.001	1.8342	ST 23:6;O7;HexNAc	C31H41NO12	[M+Cl]-
654.2335189						
655.3039370	655.302	0.002	3.0520	BMP 24:3;O	C30H53O11P	[M+Cl]-
	655.302	0.002	3.0520	LPI O-21:4	C30H53O11P	[M+Cl]-
655.3039370						
655.3039370	655.302	0.002	3.0520	PG 24:3;O	C30H53O11P	[M+Cl]-
	655.3253	0.001	2.1363	BMP 27:6;O	C33H53O11P	[M-H]-
655.3238863						
655.3238863	655.3253	0.001	2.1363	PG 27:6;O	C33H53O11P	[M-H]-
	655.3255	0.002	2.4415	MGDG 25:6	C34H52O10	[M+Cl]-
655.3238863						
655.3238863	655.3255	0.002	2.4415	ST 28:3;O4;GlcA	C34H52O10	[M+Cl]-
	655.3255	0.002	2.4415	ST 28:4;O5;Hex	C34H52O10	[M+Cl]-
655.3238863						
655.3238863	655.3253	0.001	2.1363	LPG 26:7	C32H51O9P	[M+Formate]-
	655.3253	0.001	2.1363	PA 29:6;O	C32H51O9P	[M+Formate]-
655.3238863						
655.3238863	655.3253	0.001	2.1363	PG O-26:7	C32H51O9P	[M+Formate]-
	655.3253	0.001	2.1363	LPG 25:7	C31H49O9P	[M+OAc]-
655.3238863						
655.3238863	655.3253	0.001	2.1363	PA 28:6;O	C31H49O9P	[M+OAc]-
	655.3253	0.001	2.1363	PG O-25:7	C31H49O9P	[M+OAc]-
655.3238863						
655.3382264	655.3383	0.000	0.1526	BMP 25:2	C31H57O10P	[M+Cl]-
	655.3383	0.000	0.1526	LPG 25:3;O	C31H57O10P	[M+Cl]-
655.3382264						
655.3382264	655.3383	0.000	0.1526	PG 25:2	C31H57O10P	[M+Cl]-
	655.3383	0.000	0.1526	PG O-25:3;O	C31H57O10P	[M+Cl]-
655.3382264						
656.3719171	656.3723	0.000	0.6094	ST 30:6;O;HexNAc	C38H55NO6	[M+Cl]-
	657.232	0.001	2.1301	ST 25:5;O7;GlcA	C31H42O13	[M+Cl]-
657.2333733						
657.2333733	657.232	0.001	2.1301	ST 25:6;O8;Hex	C31H42O13	[M+Cl]-
	657.5252	0.000	0.1521	CE 18:5;O	C45H70O3	[M-H]-
657.5252865						
658.3877884	658.3878	0.000	0.0000	PE O-32:9	C37H58NO7P	[M-H]-

658.3877884	658.388	0.000	0.3038	ST 30:5;O;HexNAc	C38H57NO6	[M+Cl]-
658.4080639	658.409	0.001	1.3669	CerP 34:4;O5	C34H62NO9P	[M-H]-
658.4080639	658.409	0.001	1.3669	LPS 28:3	C34H62NO9P	[M-H]-
658.4080639	658.409	0.001	1.3669	LPS O-28:4;O	C34H62NO9P	[M-H]-
658.4080639	658.409	0.001	1.3669	PE 29:3;O	C34H62NO9P	[M-H]-
658.4080639	658.409	0.001	1.3669	PS O-28:3	C34H62NO9P	[M-H]-
658.4080639	658.4091	0.001	1.6707	CAR 28:4;O4	C35H61NO8	[M+Cl]-
658.4080639	658.4091	0.001	1.6707	HexCer 29:4;O2	C35H61NO8	[M+Cl]-
658.4080639	658.4091	0.001	1.6707	ST 27:0;O3;HexNAc	C35H61NO8	[M+Cl]-
658.4080639	658.409	0.001	1.3669	CerP 33:4;O3	C33H60NO7P	[M+Formate]-
658.4080639	658.409	0.001	1.3669	LPC 25:4	C33H60NO7P	[M+Formate]-
658.4080639	658.409	0.001	1.3669	LPC O-25:5;O	C33H60NO7P	[M+Formate]-
658.4080639	658.409	0.001	1.3669	LPE 28:4	C33H60NO7P	[M+Formate]-
658.4080639	658.409	0.001	1.3669	LPE O-28:5;O	C33H60NO7P	[M+Formate]-
658.4080639	658.409	0.001	1.3669	PC O-25:4	C33H60NO7P	[M+Formate]-
658.4080639	658.409	0.001	1.3669	PE O-28:4	C33H60NO7P	[M+Formate]-
658.4080639	658.409	0.001	1.3669	CerP 32:4;O3	C32H58NO7P	[M+OAc]-
658.4080639	658.409	0.001	1.3669	LPC 24:4	C32H58NO7P	[M+OAc]-
658.4080639	658.409	0.001	1.3669	LPC O-24:5;O	C32H58NO7P	[M+OAc]-
658.4080639	658.409	0.001	1.3669	LPE 27:4	C32H58NO7P	[M+OAc]-
658.4080639	658.409	0.001	1.3669	LPE O-27:5;O	C32H58NO7P	[M+OAc]-
658.4080639	658.409	0.001	1.3669	PC O-24:4	C32H58NO7P	[M+OAc]-
658.4080639	658.409	0.001	1.3669	PE O-27:4	C32H58NO7P	[M+OAc]-
658.4080639	658.409	0.001	1.3669	PC 27:3;O	C35H64NO9P	[M-CH3]-
658.4108978	658.409	0.002	2.8857	CerP 34:4;O5	C34H62NO9P	[M-H]-
658.4108978	658.409	0.002	2.8857	LPS 28:3	C34H62NO9P	[M-H]-

658.4108978	658.409	0.002	2.8857	LPS O-28:4;O	C34H62NO9P	[M-H]-
658.4108978	658.409	0.002	2.8857	PE 29:3;O	C34H62NO9P	[M-H]-
658.4108978	658.409	0.002	2.8857	PS O-28:3	C34H62NO9P	[M-H]-
658.4108978	658.4125	0.002	2.4301	NAT 30:0;O4	C32H65NO8S	[M+Cl]-
658.4108978	658.4091	0.002	2.7339	CAR 28:4;O4	C35H61NO8	[M+Cl]-
658.4108978	658.4091	0.002	2.7339	HexCer 29:4;O2	C35H61NO8	[M+Cl]-
658.4108978	658.4091	0.002	2.7339	ST 27:0;O3;HexNAc	C35H61NO8	[M+Cl]-
658.4108978	658.409	0.002	2.8857	CerP 33:4;O3	C33H60NO7P	[M+Formate]-
658.4108978	658.409	0.002	2.8857	LPC 25:4	C33H60NO7P	[M+Formate]-
658.4108978	658.409	0.002	2.8857	LPC O-25:5;O	C33H60NO7P	[M+Formate]-
658.4108978	658.409	0.002	2.8857	LPE 28:4	C33H60NO7P	[M+Formate]-
658.4108978	658.409	0.002	2.8857	LPE O-28:5;O	C33H60NO7P	[M+Formate]-
658.4108978	658.409	0.002	2.8857	PC O-25:4	C33H60NO7P	[M+Formate]-
658.4108978	658.409	0.002	2.8857	PE O-28:4	C33H60NO7P	[M+Formate]-
658.4108978	658.409	0.002	2.8857	CerP 32:4;O3	C32H58NO7P	[M+OAc]-
658.4108978	658.409	0.002	2.8857	LPC 24:4	C32H58NO7P	[M+OAc]-
658.4108978	658.409	0.002	2.8857	LPC O-24:5;O	C32H58NO7P	[M+OAc]-
658.4108978	658.409	0.002	2.8857	LPE 27:4	C32H58NO7P	[M+OAc]-
658.4108978	658.409	0.002	2.8857	LPE O-27:5;O	C32H58NO7P	[M+OAc]-
658.4108978	658.409	0.002	2.8857	PC O-24:4	C32H58NO7P	[M+OAc]-
658.4108978	658.409	0.002	2.8857	PE O-27:4	C32H58NO7P	[M+OAc]-
658.4108978	658.409	0.002	2.8857	PC 27:3;O	C35H64NO9P	[M-CH3]-
659.3331302	659.3333	0.000	0.1517	BMP 24:1;O	C30H57O11P	[M+Cl]-
659.3331302	659.3333	0.000	0.1517	LPI O-21:2	C30H57O11P	[M+Cl]-
	659.3333	0.000	0.1517	PG 24:1;O	C30H57O11P	[M+Cl]-

659.3331302						
660.4263594	660.4246	0.002	2.7255	CerP 34:3;O5	C34H64NO9P	[M-H]-
	660.4246	0.002	2.7255	LPS 28:2	C34H64NO9P	[M-H]-
660.4263594						
660.4263594	660.4246	0.002	2.7255	LPS O-28:3;O	C34H64NO9P	[M-H]-
	660.4246	0.002	2.7255	PE 29:2;O	C34H64NO9P	[M-H]-
660.4263594						
660.4263594	660.4246	0.002	2.7255	PS O-28:2	C34H64NO9P	[M-H]-
	660.4248	0.002	2.4227	CAR 28:3;O4	C35H63NO8	[M+Cl]-
660.4263594						
660.4263594	660.4248	0.002	2.4227	HexCer 29:3;O2	C35H63NO8	[M+Cl]-
	660.4246	0.002	2.7255	CerP 33:3;O3	C33H62NO7P	[M+Formate]-
660.4263594						
660.4263594	660.4246	0.002	2.7255	LPC 25:3	C33H62NO7P	[M+Formate]-
	660.4246	0.002	2.7255	LPC O-25:4;O	C33H62NO7P	[M+Formate]-
660.4263594						
660.4263594	660.4246	0.002	2.7255	LPE 28:3	C33H62NO7P	[M+Formate]-
	660.4246	0.002	2.7255	LPE O-28:4;O	C33H62NO7P	[M+Formate]-
660.4263594						
660.4263594	660.4246	0.002	2.7255	PC O-25:3	C33H62NO7P	[M+Formate]-
	660.4246	0.002	2.7255	PE O-28:3	C33H62NO7P	[M+Formate]-
660.4263594						
660.4263594	660.4246	0.002	2.7255	CerP 32:3;O3	C32H60NO7P	[M+OAc]-
	660.4246	0.002	2.7255	LPC 24:3	C32H60NO7P	[M+OAc]-
660.4263594						
660.4263594	660.4246	0.002	2.7255	LPC O-24:4;O	C32H60NO7P	[M+OAc]-
	660.4246	0.002	2.7255	LPE 27:3	C32H60NO7P	[M+OAc]-
660.4263594						
660.4263594	660.4246	0.002	2.7255	LPE O-27:4;O	C32H60NO7P	[M+OAc]-
	660.4246	0.002	2.7255	PC O-24:3	C32H60NO7P	[M+OAc]-
660.4263594						
660.4263594	660.4246	0.002	2.7255	PE O-27:3	C32H60NO7P	[M+OAc]-
	660.4246	0.002	2.7255	PC 27:2;O	C35H66NO9P	[M-CH3]-
660.4263594						
661.3032977	661.303	0.000	0.4536	SQDG 20:0	C29H54O12S	[M+Cl]-
	661.3263	0.001	0.7561	SQDG 23:3	C32H54O12S	[M-H]-
661.3258010						
661.3258010	661.3263	0.001	0.7561	ST 30:2;O7;S	C30H50O10S	[M+OAc]-

	662.323	0.001	1.2079	CerP 32:6;O5	C32H54NO9P	[M+Cl]-
662.3237802						
662.3237802	662.323	0.001	1.2079	LPS 26:5	C32H54NO9P	[M+Cl]-
	662.323	0.001	1.2079	LPS O-26:6;O	C32H54NO9P	[M+Cl]-
662.3237802						
662.3237802	662.323	0.001	1.2079	PC 24:5;O	C32H54NO9P	[M+Cl]-
	662.323	0.001	1.2079	PE 27:5;O	C32H54NO9P	[M+Cl]-
662.3237802						
662.3237802	662.323	0.001	1.2079	PS O-26:5	C32H54NO9P	[M+Cl]-
	662.358	0.000	0.0000	ST 29:1;O7;T	C31H55NO9S	[M+Formate]-
662.3579548						
662.3579548	662.358	0.000	0.0000	ST 28:1;O7;T	C30H53NO9S	[M+OAc]-
	663.3183	0.001	1.2061	EPC 29:6;O5	C31H53N2O9P	[M+Cl]-
663.3174400						
664.3741946	664.3751	0.001	1.3547	CerP 33:4;O4	C33H60NO8P	[M+Cl]-
	664.3751	0.001	1.3547	LPC 25:4;O	C33H60NO8P	[M+Cl]-
664.3741946						
664.3741946	664.3751	0.001	1.3547	LPE 28:4;O	C33H60NO8P	[M+Cl]-
	664.3751	0.001	1.3547	LPS O-27:4	C33H60NO8P	[M+Cl]-
664.3741946						
664.3741946	664.3751	0.001	1.3547	PC 25:3	C33H60NO8P	[M+Cl]-
	664.3751	0.001	1.3547	PC O-25:4;O	C33H60NO8P	[M+Cl]-
664.3741946						
664.3741946	664.3751	0.001	1.3547	PE 28:3	C33H60NO8P	[M+Cl]-
	664.3751	0.001	1.3547	PE O-28:4;O	C33H60NO8P	[M+Cl]-
664.3741946						
664.3741946	664.3736	0.001	0.9031	ST 29:0;O7;T	C31H57NO9S	[M+Formate]-
	664.3736	0.001	0.9031	ST 28:0;O7;T	C30H55NO9S	[M+OAc]-
664.3741946						
665.2858765	665.2863	0.000	0.6012	BMP 25:5;O	C31H51O11P	[M+Cl]-
	665.2863	0.000	0.6012	LPI O-22:6	C31H51O11P	[M+Cl]-
665.2858765						
665.2858765	665.2863	0.000	0.6012	PG 25:5;O	C31H51O11P	[M+Cl]-
	665.2849	0.001	1.5031	SQDG 20:3	C29H48O12S	[M+Formate]-
665.2858765						
667.3377997	667.3383	0.001	0.7492	BMP 26:3	C32H57O10P	[M+Cl]-
	667.3383	0.001	0.7492	LPG 26:4;O	C32H57O10P	[M+Cl]-
667.3377997						
667.3377997	667.3383	0.001	0.7492	PG 26:3	C32H57O10P	[M+Cl]-

667.3377997	667.3383	0.001	0.7492	PG O-26:4;O	C32H57O10P	[M+Cl]-
667.3377997	667.3369	0.001	1.3486	ST 30:0;O8;S	C30H54O11S	[M+Formate]-
667.3377997	667.3369	0.001	1.3486	ST 29:0;O8;S	C29H52O11S	[M+OAc]-
667.3751828	667.3747	0.001	0.7492	LPG 27:3	C33H61O9P	[M+Cl]-
667.3751828	667.3747	0.001	0.7492	LPG O-27:4;O	C33H61O9P	[M+Cl]-
667.3751828	667.3747	0.001	0.7492	PA 30:2;O	C33H61O9P	[M+Cl]-
667.3751828	667.3747	0.001	0.7492	PG O-27:3	C33H61O9P	[M+Cl]-
668.3339888	668.3336	0.000	0.5985	CerP 31:4;O6	C31H56NO10P	[M+Cl]-
668.3339888	668.3336	0.000	0.5985	LPS 25:3;O	C31H56NO10P	[M+Cl]-
668.3339888	668.3336	0.000	0.5985	PS 25:2	C31H56NO10P	[M+Cl]-
668.3339888	668.3336	0.000	0.5985	PS O-25:3;O	C31H56NO10P	[M+Cl]-
669.3547212	669.354	0.001	1.0458	BMP 26:2	C32H59O10P	[M+Cl]-
669.3547212	669.354	0.001	1.0458	LPG 26:3;O	C32H59O10P	[M+Cl]-
669.3547212	669.354	0.001	1.0458	PG 26:2	C32H59O10P	[M+Cl]-
669.3547212	669.354	0.001	1.0458	PG O-26:3;O	C32H59O10P	[M+Cl]-
669.3653236	669.3652	0.000	0.1494	EPC 29:3;O5	C31H59N2O9P	[M+Cl]-
670.3866516	670.3856	0.001	1.4917	CerP 32:2;O5	C32H62NO9P	[M+Cl]-
670.3866516	670.3856	0.001	1.4917	LPS 26:1	C32H62NO9P	[M+Cl]-
670.3866516	670.3856	0.001	1.4917	LPS O-26:2;O	C32H62NO9P	[M+Cl]-
670.3866516	670.3856	0.001	1.4917	PC 24:1;O	C32H62NO9P	[M+Cl]-
670.3866516	670.3856	0.001	1.4917	PE 27:1;O	C32H62NO9P	[M+Cl]-
670.3866516	670.3856	0.001	1.4917	PS O-26:1	C32H62NO9P	[M+Cl]-
674.3071796	674.3101	0.003	4.4490	ST 28:7;O4;HexNAc	C36H49NO9	[M+Cl]-
674.3820212	674.3827	0.001	1.0380	PE 32:8	C37H58NO8P	[M-H]-
	674.3827	0.001	1.0380	PE O-32:9;O	C37H58NO8P	[M-H]-

674.3820212						
674.3820212	674.3829	0.001	1.3346	ST 30:5;O2;HexNAc	C38H57NO7	[M+Cl]-
	674.3827	0.001	1.0380	PC 30:8	C38H60NO8P	[M-CH3]-
674.3820212						
674.4041802	674.4039	0.000	0.4448	CerP 34:4;O6	C34H62NO10P	[M-H]-
	674.4039	0.000	0.4448	LPS 28:3;O	C34H62NO10P	[M-H]-
674.4041802						
674.4041802	674.4039	0.000	0.4448	PS 28:2	C34H62NO10P	[M-H]-
	674.4039	0.000	0.4448	PS O-28:3;O	C34H62NO10P	[M-H]-
674.4041802						
674.4041802	674.404	0.000	0.1483	HexCer 29:4;O3	C35H61NO9	[M+Cl]-
	674.404	0.000	0.1483	ST 27:0;O4;HexNAc	C35H61NO9	[M+Cl]-
674.4041802						
674.4041802	674.4039	0.000	0.4448	CerP 33:4;O4	C33H60NO8P	[M+Formate]-
	674.4039	0.000	0.4448	LPC 25:4;O	C33H60NO8P	[M+Formate]-
674.4041802						
674.4041802	674.4039	0.000	0.4448	LPE 28:4;O	C33H60NO8P	[M+Formate]-
	674.4039	0.000	0.4448	LPS O-27:4	C33H60NO8P	[M+Formate]-
674.4041802						
674.4041802	674.4039	0.000	0.4448	PC 25:3	C33H60NO8P	[M+Formate]-
	674.4039	0.000	0.4448	PC O-25:4;O	C33H60NO8P	[M+Formate]-
674.4041802						
674.4041802	674.4039	0.000	0.4448	PE 28:3	C33H60NO8P	[M+Formate]-
	674.4039	0.000	0.4448	PE O-28:4;O	C33H60NO8P	[M+Formate]-
674.4041802						
674.4041802	674.4039	0.000	0.4448	CerP 32:4;O4	C32H58NO8P	[M+OAc]-
	674.4039	0.000	0.4448	LPC 24:4;O	C32H58NO8P	[M+OAc]-
674.4041802						
674.4041802	674.4039	0.000	0.4448	LPE 27:4;O	C32H58NO8P	[M+OAc]-
	674.4039	0.000	0.4448	LPS O-26:4	C32H58NO8P	[M+OAc]-
674.4041802						
674.4041802	674.4039	0.000	0.4448	PC 24:3	C32H58NO8P	[M+OAc]-
	674.4039	0.000	0.4448	PC O-24:4;O	C32H58NO8P	[M+OAc]-
674.4041802						
674.4041802	674.4039	0.000	0.4448	PE 27:3	C32H58NO8P	[M+OAc]-
	674.4039	0.000	0.4448	PE O-27:4;O	C32H58NO8P	[M+OAc]-
674.4041802						
675.3439579	675.3434	0.001	0.7404	LPG 28:6	C34H57O9P	[M+Cl]-

	675.3434	0.001	0.7404	LPG O-28:7;O	C34H57O9P	[M+Cl]-
675.3439579						
675.3439579	675.3434	0.001	0.7404	PA 31:5;O	C34H57O9P	[M+Cl]-
	675.3434	0.001	0.7404	PG O-28:6	C34H57O9P	[M+Cl]-
675.3439579						
676.3031144	676.3023	0.001	1.1829	LPS 26:6;O	C32H52NO10P	[M+Cl]-
	676.3023	0.001	1.1829	PS 26:5	C32H52NO10P	[M+Cl]-
676.3031144						
676.3031144	676.3023	0.001	1.1829	PS O-26:6;O	C32H52NO10P	[M+Cl]-
	676.3256	0.001	1.9222	PS 29:8	C35H52NO10P	[M-H]-
676.3243489						
676.3243489	676.3258	0.001	2.0700	ST 28:6;O4;HexNAc	C36H51NO9	[M+Cl]-
	676.4195	0.003	3.6959	CerP 34:3;O6	C34H64NO10P	[M-H]-
676.4220500						
676.4220500	676.4195	0.003	3.6959	LPS 28:2;O	C34H64NO10P	[M-H]-
	676.4195	0.003	3.6959	PS 28:1	C34H64NO10P	[M-H]-
676.4220500						
676.4220500	676.4195	0.003	3.6959	PS O-28:2;O	C34H64NO10P	[M-H]-
	676.4197	0.002	3.5481	HexCer 29:3;O3	C35H63NO9	[M+Cl]-
676.4220500						
676.4220500	676.4195	0.003	3.6959	CerP 33:3;O4	C33H62NO8P	[M+Formate]-
	676.4195	0.003	3.6959	LPC 25:3;O	C33H62NO8P	[M+Formate]-
676.4220500						
676.4220500	676.4195	0.003	3.6959	LPE 28:3;O	C33H62NO8P	[M+Formate]-
	676.4195	0.003	3.6959	LPS O-27:3	C33H62NO8P	[M+Formate]-
676.4220500						
676.4220500	676.4195	0.003	3.6959	PC 25:2	C33H62NO8P	[M+Formate]-
	676.4195	0.003	3.6959	PC O-25:3;O	C33H62NO8P	[M+Formate]-
676.4220500						
676.4220500	676.4195	0.003	3.6959	PE 28:2	C33H62NO8P	[M+Formate]-
	676.4195	0.003	3.6959	PE O-28:3;O	C33H62NO8P	[M+Formate]-
676.4220500						
676.4220500	676.4195	0.003	3.6959	CerP 32:3;O4	C32H60NO8P	[M+OAc]-
	676.4195	0.003	3.6959	LPC 24:3;O	C32H60NO8P	[M+OAc]-
676.4220500						
676.4220500	676.4195	0.003	3.6959	LPE 27:3;O	C32H60NO8P	[M+OAc]-
	676.4195	0.003	3.6959	LPS O-26:3	C32H60NO8P	[M+OAc]-
676.4220500						
676.4220500	676.4195	0.003	3.6959	PC 24:2	C32H60NO8P	[M+OAc]-

676.4220500	676.4195	0.003	3.6959	PC O-24:3;O	C32H60NO8P	[M+OAc]-
676.4220500	676.4195	0.003	3.6959	PE 27:2	C32H60NO8P	[M+OAc]-
676.4220500	676.4195	0.003	3.6959	PE O-27:3;O	C32H60NO8P	[M+OAc]-
678.2837586	678.2816	0.002	3.2435	PS 25:5;O	C31H50NO11P	[M+Cl]-
678.3541181	678.3543	0.000	0.2948	CerP 33:5;O5	C33H58NO9P	[M+Cl]-
678.3541181	678.3543	0.000	0.2948	LPS 27:4	C33H58NO9P	[M+Cl]-
678.3541181	678.3543	0.000	0.2948	LPS O-27:5;O	C33H58NO9P	[M+Cl]-
678.3541181	678.3543	0.000	0.2948	PC 25:4;O	C33H58NO9P	[M+Cl]-
678.3541181	678.3543	0.000	0.2948	PE 28:4;O	C33H58NO9P	[M+Cl]-
678.3541181	678.3543	0.000	0.2948	PS O-27:4	C33H58NO9P	[M+Cl]-
678.3963105	678.3988	0.003	3.6851	PS 27:1;O	C33H62NO11P	[M-H]-
678.3963105	678.399	0.003	3.8326	HexCer 28:3;O4	C34H61NO10	[M+Cl]-
678.3963105	678.3988	0.003	3.6851	CerP 32:3;O5	C32H60NO9P	[M+Formate]-
678.3963105	678.3988	0.003	3.6851	LPS 26:2	C32H60NO9P	[M+Formate]-
678.3963105	678.3988	0.003	3.6851	LPS O-26:3;O	C32H60NO9P	[M+Formate]-
678.3963105	678.3988	0.003	3.6851	PC 24:2;O	C32H60NO9P	[M+Formate]-
678.3963105	678.3988	0.003	3.6851	PE 27:2;O	C32H60NO9P	[M+Formate]-
678.3963105	678.3988	0.003	3.6851	PS O-26:2	C32H60NO9P	[M+Formate]-
678.3963105	678.3988	0.003	3.6851	CerP 31:3;O5	C31H58NO9P	[M+OAc]-
678.3963105	678.3988	0.003	3.6851	LPS 25:2	C31H58NO9P	[M+OAc]-
678.3963105	678.3988	0.003	3.6851	LPS O-25:3;O	C31H58NO9P	[M+OAc]-
678.3963105	678.3988	0.003	3.6851	PC 23:2;O	C31H58NO9P	[M+OAc]-
678.3963105	678.3988	0.003	3.6851	PE 26:2;O	C31H58NO9P	[M+OAc]-
678.3963105	678.3988	0.003	3.6851	PS O-25:2	C31H58NO9P	[M+OAc]-
	681.3257	0.002	2.4951	PI 22:2;O	C31H55O14P	[M-H]-

681.3273545						
681.3273545	681.3259	0.002	2.2016	ST 26:0;O7;GlcA	C32H54O13	[M+Cl]-
	681.3259	0.002	2.2016	ST 26:1;O8;Hex	C32H54O13	[M+Cl]-
681.3273545						
681.3273545	681.3288	0.002	2.2016	EPC 29:5;O6	C31H55N2O10P	[M+Cl]-
	681.3257	0.002	2.4951	LPI 21:3	C30H53O12P	[M+Formate]-
681.3273545						
681.3273545	681.3257	0.002	2.4951	LPI O-21:4;O	C30H53O12P	[M+Formate]-
	681.3257	0.002	2.4951	PI O-21:3	C30H53O12P	[M+Formate]-
681.3273545						
681.3273545	681.3257	0.002	2.4951	LPI 20:3	C29H51O12P	[M+OAc]-
	681.3257	0.002	2.4951	LPI O-20:4;O	C29H51O12P	[M+OAc]-
681.3273545						
681.3273545	681.3257	0.002	2.4951	PI O-20:3	C29H51O12P	[M+OAc]-
	681.3927	0.002	2.3481	TG 38:10	C41H58O6	[M+Cl]-
681.3943192						
682.3242808	682.3267	0.002	3.5174	ST 30:5;O7;T	C32H49NO9S	[M+OAc]-
	683.406	0.000	0.2927	LPG 28:2	C34H65O9P	[M+Cl]-
683.4062713						
683.4062713	683.406	0.000	0.2927	LPG O-28:3;O	C34H65O9P	[M+Cl]-
	683.406	0.000	0.2927	PA 31:1;O	C34H65O9P	[M+Cl]-
683.4062713						
683.4062713	683.406	0.000	0.2927	PG O-28:2	C34H65O9P	[M+Cl]-
	684.3285	0.002	2.1919	PS 25:2;O	C31H56NO11P	[M+Cl]-
684.3270503						
686.3063358	686.303	0.003	4.9541	ST 23:3;O8;HexNAc	C31H47NO13	[M+Formate]-
	686.303	0.003	4.9541	ST 22:3;O8;HexNAc	C30H45NO13	[M+OAc]-
686.3063358						
687.3647103	687.3646	0.000	0.1455	BMP 26:1;O	C32H61O11P	[M+Cl]-
	687.3646	0.000	0.1455	LPI O-23:2	C32H61O11P	[M+Cl]-
687.3647103						
687.3647103	687.3646	0.000	0.1455	PG 26:1;O	C32H61O11P	[M+Cl]-
	687.3758	0.001	1.6003	EPC 29:2;O6	C31H61N2O10P	[M+Cl]-
687.3768480						
688.3445224	688.3469	0.002	3.4866	HexCer 28:6;O5	C34H55NO11	[M+Cl]-
	688.3469	0.002	3.4866	ST 26:2;O6;HexNAc	C34H55NO11	[M+Cl]-
688.3445224						
688.3445224	688.3467	0.002	3.1961	CerP 32:6;O6	C32H54NO10P	[M+Formate]-

688.3445224	688.3467	0.002	3.1961	LPS 26:5;O	C32H54NO10P	[M+Formate]-
688.3445224	688.3467	0.002	3.1961	PS 26:4	C32H54NO10P	[M+Formate]-
688.3445224	688.3467	0.002	3.1961	PS O-26:5;O	C32H54NO10P	[M+Formate]-
688.3445224	688.3467	0.002	3.1961	CerP 31:6;O6	C31H52NO10P	[M+OAc]-
688.3445224	688.3467	0.002	3.1961	LPS 25:5;O	C31H52NO10P	[M+OAc]-
688.3445224	688.3467	0.002	3.1961	PS 25:4	C31H52NO10P	[M+OAc]-
688.3445224	688.3467	0.002	3.1961	PS O-25:5;O	C31H52NO10P	[M+OAc]-
688.3627944	688.362	0.001	1.1622	PE 32:9;O	C37H56NO9P	[M-H]-
688.3627944	688.3622	0.001	0.8716	ST 30:6;O3;HexNAc	C38H55NO8	[M+Cl]-
689.5628157	689.5645	0.002	2.4653	CE 17:0;O	C44H78O3	[M+Cl]-
690.3036361	690.3051	0.001	2.0281	ST 28:7;O5;HexNAc	C36H49NO10	[M+Cl]-
690.3431613	690.3413	0.002	2.7523	PS 30:8	C36H54NO10P	[M-H]-
690.3431613	690.3414	0.002	2.4625	ST 29:6;O4;HexNAc	C37H53NO9	[M+Cl]-
691.3521361	691.3536	0.002	2.1697	PA O-35:10	C38H57O7P	[M+Cl]-
692.2964809	692.2972	0.001	1.0111	PS 26:5;O	C32H52NO11P	[M+Cl]-
693.3046202	693.3047	0.000	0.1442	ST 30:6;O5;GlcA	C36H50O11	[M+Cl]-
693.3046202	693.3047	0.000	0.1442	ST 30:7;O6;Hex	C36H50O11	[M+Cl]-
693.3167805	693.3176	0.001	1.1539	BMP 27:5;O	C33H55O11P	[M+Cl]-
693.3167805	693.3176	0.001	1.1539	LPI O-24:6	C33H55O11P	[M+Cl]-
693.3167805	693.3176	0.001	1.1539	PG 27:5;O	C33H55O11P	[M+Cl]-
693.3167805	693.3162	0.001	0.8654	SQDG 22:3	C31H52O12S	[M+Formate]-
693.3167805	693.3162	0.001	0.8654	SQDG 21:3	C30H50O12S	[M+OAc]-
694.3135925	694.3129	0.001	1.0082	PS 26:4;O	C32H54NO11P	[M+Cl]-
694.3472248	694.3492	0.002	2.8804	CerP 33:5;O6	C33H58NO10P	[M+Cl]-
694.3472248	694.3492	0.002	2.8804	LPS 27:4;O	C33H58NO10P	[M+Cl]-
694.3472248	694.3492	0.002	2.8804	PS 27:3	C33H58NO10P	[M+Cl]-

	694.3492	0.002	2.8804	PS O-27:4;O	C33H58NO10P	[M+Cl]-
694.3472248						
695.4065857	695.406	0.001	0.8628	LPG 29:3	C35H65O9P	[M+Cl]-
	695.406	0.001	0.8628	LPG O-29:4;O	C35H65O9P	[M+Cl]-
695.4065857						
695.4065857	695.406	0.001	0.8628	PA 32:2;O	C35H65O9P	[M+Cl]-
	695.406	0.001	0.8628	PG O-29:3	C35H65O9P	[M+Cl]-
695.4065857						
698.3824116	698.3805	0.002	2.7206	CerP 33:3;O6	C33H62NO10P	[M+Cl]-
	698.3805	0.002	2.7206	LPS 27:2;O	C33H62NO10P	[M+Cl]-
698.3824116						
698.3824116	698.3805	0.002	2.7206	PS 27:1	C33H62NO10P	[M+Cl]-
	698.3805	0.002	2.7206	PS O-27:2;O	C33H62NO10P	[M+Cl]-
698.3824116						
698.4177009	698.4191	0.001	2.0045	PE O-35:10	C40H62NO7P	[M-H]-
	698.4169	0.001	1.1454	CerP 34:2;O5	C34H66NO9P	[M+Cl]-
698.4177009						
698.4177009	698.4169	0.001	1.1454	LPS 28:1	C34H66NO9P	[M+Cl]-
	698.4169	0.001	1.1454	LPS O-28:2;O	C34H66NO9P	[M+Cl]-
698.4177009						
698.4177009	698.4169	0.001	1.1454	PC 26:1;O	C34H66NO9P	[M+Cl]-
	698.4169	0.001	1.1454	PE 29:1;O	C34H66NO9P	[M+Cl]-
698.4177009						
698.4177009	698.4169	0.001	1.1454	PS O-28:1	C34H66NO9P	[M+Cl]-
	700.2742	0.002	2.5704	ST 25:5;O8;HexNAc	C33H47NO13	[M+Cl]-
700.2759178						
703.3133723	703.31	0.003	4.6921	PI 24:5;O	C33H53O14P	[M-H]-
	703.3102	0.003	4.5499	ST 28:3;O7;GlcA	C34H52O13	[M+Cl]-
703.3133723						
703.3133723	703.3102	0.003	4.5499	ST 28:4;O8;Hex	C34H52O13	[M+Cl]-
	703.31	0.003	4.6921	LPI 23:6	C32H51O12P	[M+Formate]-
703.3133723						
703.3133723	703.31	0.003	4.6921	PI O-23:6	C32H51O12P	[M+Formate]-
	703.31	0.003	4.6921	LPI 22:6	C31H49O12P	[M+OAc]-
703.3133723						
703.3133723	703.31	0.003	4.6921	PI O-22:6	C31H49O12P	[M+OAc]-
	704.3207	0.003	4.1174	ST 29:7;O5;HexNAc	C37H51NO10	[M+Cl]-

704.3236112						
704.3338740	704.3336	0.000	0.4259	LPS 28:6;O	C34H56NO10P	[M+Cl]-
	704.3336	0.000	0.4259	PS 28:5	C34H56NO10P	[M+Cl]-
704.3338740						
704.3338740	704.3336	0.000	0.4259	PS O-28:6;O	C34H56NO10P	[M+Cl]-
	704.3418	0.003	4.5432	HexCer 28:6;O6	C34H55NO12	[M+Cl]-
704.3450448						
704.3450448	704.3418	0.003	4.5432	ST 26:2;O7;HexNAc	C34H55NO12	[M+Cl]-
	704.3417	0.003	4.8272	PS 26:4;O	C32H54NO11P	[M+Formate]-
704.3450448						
704.3450448	704.3417	0.003	4.8272	PS 25:4;O	C31H52NO11P	[M+OAc]-
	705.3024	0.002	3.1192	PI 21:1;O	C30H55O14P	[M+Cl]-
705.3045918						
705.3332967	705.3329	0.000	0.5671	PA 35:10	C38H55O8P	[M+Cl]-
	705.3339	0.001	0.8507	ST 26:1;O8;GlcA	C32H52O14	[M+Formate]-
705.3332967						
705.3332967	705.3339	0.001	0.8507	ST 25:1;O8;GlcA	C31H50O14	[M+OAc]-
	705.3409	0.002	2.9773	PA 33:9;O	C36H53O9P	[M+Formate]-
705.3430507						
705.3430507	705.3409	0.002	2.9773	PA 32:9;O	C35H51O9P	[M+OAc]-
	705.354	0.000	0.4253	BMP 29:5	C35H59O10P	[M+Cl]-
705.3536492						
705.3536492	705.354	0.000	0.4253	LPG 29:6;O	C35H59O10P	[M+Cl]-
	705.354	0.000	0.4253	PG 29:5	C35H59O10P	[M+Cl]-
705.3536492						
705.3536492	705.354	0.000	0.4253	PG O-29:6;O	C35H59O10P	[M+Cl]-
	706.3492	0.001	1.9820	CerP 34:6;O6	C34H58NO10P	[M+Cl]-
706.3478083						
706.3478083	706.3492	0.001	1.9820	LPS 28:5;O	C34H58NO10P	[M+Cl]-
	706.3492	0.001	1.9820	PS 28:4	C34H58NO10P	[M+Cl]-
706.3478083						
706.3478083	706.3492	0.001	1.9820	PS O-28:5;O	C34H58NO10P	[M+Cl]-
	706.3656	0.001	1.9820	ST 24:0;O8;HexNAc	C32H55NO13	[M+Formate]-
706.3641289						
706.3641289	706.3656	0.001	1.9820	ST 23:0;O8;HexNAc	C31H53NO13	[M+OAc]-
	707.2969	0.000	0.5655	LPI 24:6	C33H53O12P	[M+Cl]-
707.2964459						
707.2964459	707.2969	0.000	0.5655	PI O-24:6	C33H53O12P	[M+Cl]-

707.3150206	707.318	0.003	4.2414	PI 21:0;O	C30H57O14P	[M+Cl]-
708.3538821	708.3518	0.002	2.9646	PS 30:7;O	C36H56NO11P	[M-H]-
708.3538821	708.352	0.002	2.6823	ST 29:5;O5;HexNAc	C37H55NO10	[M+Cl]-
708.3538821	708.3518	0.002	2.9646	PE 30:8;O	C35H54NO9P	[M+Formate]-
708.3538821	708.3518	0.002	2.9646	PS O-29:8	C35H54NO9P	[M+Formate]-
708.3538821	708.3518	0.002	2.9646	PE 29:8;O	C34H52NO9P	[M+OAc]-
708.3538821	708.3518	0.002	2.9646	PS O-28:8	C34H52NO9P	[M+OAc]-
708.3738688	708.3731	0.001	0.9882	HexCer 28:4;O6	C34H59NO12	[M+Cl]-
708.3738688	708.3731	0.001	0.9882	ST 26:0;O7;HexNAc	C34H59NO12	[M+Cl]-
708.3738688	708.373	0.001	1.2705	PS 26:2;O	C32H58NO11P	[M+Formate]-
708.3738688	708.373	0.001	1.2705	PS 25:2;O	C31H56NO11P	[M+OAc]-
709.3145085	709.3125	0.002	2.8196	LPI 24:5	C33H55O12P	[M+Cl]-
709.3145085	709.3125	0.002	2.8196	LPI O-24:6;O	C33H55O12P	[M+Cl]-
709.3145085	709.3125	0.002	2.8196	PI O-24:5	C33H55O12P	[M+Cl]-
709.3369267	709.3358	0.001	1.5507	PG 29:8	C35H53O10P	[M+Formate]-
709.3369267	709.3358	0.001	1.5507	PG 28:8	C34H51O10P	[M+OAc]-
709.4225778	709.4239	0.001	1.8325	PA O-39:11	C42H63O7P	[M-H]-
709.4225778	709.4217	0.001	1.2686	LPG 30:3	C36H67O9P	[M+Cl]-
709.4225778	709.4217	0.001	1.2686	LPG O-30:4;O	C36H67O9P	[M+Cl]-
709.4225778	709.4217	0.001	1.2686	PA 33:2;O	C36H67O9P	[M+Cl]-
709.4225778	709.4217	0.001	1.2686	PG O-30:3	C36H67O9P	[M+Cl]-
709.4225778	709.424	0.002	2.1144	DG 40:11;O	C43H62O6	[M+Cl]-
709.4225778	709.424	0.002	2.1144	TG 40:10	C43H62O6	[M+Cl]-
709.4225778	709.424	0.002	2.1144	TG O-40:11;O	C43H62O6	[M+Cl]-
711.4378920	711.4373	0.001	0.8434	LPG 30:2	C36H69O9P	[M+Cl]-
711.4378920	711.4373	0.001	0.8434	LPG O-30:3;O	C36H69O9P	[M+Cl]-

711.4378920	711.4373	0.001	0.8434	PA 33:1;O	C36H69O9P	[M+Cl]-
711.4378920	711.4373	0.001	0.8434	PG O-30:2	C36H69O9P	[M+Cl]-
712.3967740	712.3984	0.002	2.2459	PE 35:10	C40H60NO8P	[M-H]-
712.3967740	712.3962	0.001	0.8422	CerP 34:3;O6	C34H64NO10P	[M+Cl]-
712.3967740	712.3962	0.001	0.8422	LPS 28:2;O	C34H64NO10P	[M+Cl]-
712.3967740	712.3962	0.001	0.8422	PS 28:1	C34H64NO10P	[M+Cl]-
712.3967740	712.3962	0.001	0.8422	PS O-28:2;O	C34H64NO10P	[M+Cl]-
716.3746731	716.378	0.003	4.7461	IPC 29:5;O3	C35H60NO12P	[M-H]-
716.3746731	716.3782	0.004	4.8857	HexCer 30:6;O5	C36H59NO11	[M+Cl]-
716.3746731	716.3782	0.004	4.8857	ST 28:2;O6;HexNAc	C36H59NO11	[M+Cl]-
716.3746731	716.378	0.003	4.7461	CerP 34:6;O6	C34H58NO10P	[M+Formate]-
716.3746731	716.378	0.003	4.7461	LPS 28:5;O	C34H58NO10P	[M+Formate]-
716.3746731	716.378	0.003	4.7461	PS 28:4	C34H58NO10P	[M+Formate]-
716.3746731	716.378	0.003	4.7461	PS O-28:5;O	C34H58NO10P	[M+Formate]-
716.3746731	716.378	0.003	4.7461	CerP 33:6;O6	C33H56NO10P	[M+OAc]-
716.3746731	716.378	0.003	4.7461	LPS 27:5;O	C33H56NO10P	[M+OAc]-
716.3746731	716.378	0.003	4.7461	PS 27:4	C33H56NO10P	[M+OAc]-
716.3746731	716.378	0.003	4.7461	PS O-27:5;O	C33H56NO10P	[M+OAc]-
717.2925742	717.2895	0.003	4.3218	ST 28:4;O8;GlcA	C34H50O14	[M+Cl]-
717.2925742	717.2893	0.003	4.6007	PI 23:6	C32H49O13P	[M+Formate]-
717.2925742	717.2893	0.003	4.6007	PI 22:6	C31H47O13P	[M+OAc]-
717.4938492	717.4947	0.001	1.2544	TG 39:5;O3	C42H70O9	[M-H]-
717.4938492	717.4947	0.001	1.2544	DG 38:6;O2	C41H68O7	[M+Formate]-
717.4938492	717.4947	0.001	1.2544	TG 38:5;O	C41H68O7	[M+Formate]-
	717.4947	0.001	1.2544	TG O-38:6;O2	C41H68O7	[M+Formate]-

717.4938492						
717.4938492	717.4947	0.001	1.2544	DG 37:6;O2	C40H66O7	[M+OAc]-
	717.4947	0.001	1.2544	TG 37:5;O	C40H66O7	[M+OAc]-
717.4938492						
717.4938492	717.4947	0.001	1.2544	TG O-37:6;O2	C40H66O7	[M+OAc]-
	718.3129	0.002	3.3412	PS 28:6;O	C34H54NO11P	[M+Cl]-
718.3152600						
719.3334135	719.3333	0.000	0.2780	BMP 29:6;O	C35H57O11P	[M+Cl]-
	719.3333	0.000	0.2780	LPI O-26:7	C35H57O11P	[M+Cl]-
719.3334135						
719.3334135	719.3333	0.000	0.2780	PG 29:6;O	C35H57O11P	[M+Cl]-
	719.3566	0.000	0.5561	PG 32:9;O	C38H57O11P	[M-H]-
719.3561399						
719.3561399	719.3566	0.000	0.5561	PA 34:9;O	C37H55O9P	[M+Formate]-
	719.3566	0.000	0.5561	PA 33:9;O	C36H53O9P	[M+OAc]-
719.3561399						
720.3275602	720.3285	0.001	1.2494	IPC 28:6;O2	C34H56NO11P	[M+Cl]-
	720.3285	0.001	1.2494	PS 28:5;O	C34H56NO11P	[M+Cl]-
720.3275602						
720.3649399	720.3649	0.000	0.0000	CerP 35:6;O6	C35H60NO10P	[M+Cl]-
	720.3649	0.000	0.0000	LPS 29:5;O	C35H60NO10P	[M+Cl]-
720.3649399						
720.3649399	720.3649	0.000	0.0000	PS 29:4	C35H60NO10P	[M+Cl]-
	720.3649	0.000	0.0000	PS O-29:5;O	C35H60NO10P	[M+Cl]-
720.3649399						
721.3746430	721.3722	0.002	3.3270	PG 32:8;O	C38H59O11P	[M-H]-
	721.3724	0.002	3.0497	MGDG 30:8	C39H58O10	[M+Cl]-
721.3746430						
721.3746430	721.3722	0.002	3.3270	PA 34:8;O	C37H57O9P	[M+Formate]-
	721.3722	0.002	3.3270	PA 33:8;O	C36H55O9P	[M+OAc]-
721.3746430						
722.3728903	722.3757	0.003	3.8761	ST 29:4;O8;HexNAc	C37H57NO13	[M-H]-
	722.3757	0.003	3.8761	ST 28:4;O6;HexNAc	C36H55NO11	[M+Formate]-
722.3728903						
722.3728903	722.3757	0.003	3.8761	ST 27:4;O6;HexNAc	C35H53NO11	[M+OAc]-
	722.3805	0.003	3.8761	CerP 35:5;O6	C35H62NO10P	[M+Cl]-
722.3833697						
722.3833697	722.3805	0.003	3.8761	LPS 29:4;O	C35H62NO10P	[M+Cl]-

	722.3805	0.003	3.8761	PS 29:3	C35H62NO10P	[M+Cl]-
722.3833697						
722.3833697	722.3805	0.003	3.8761	PS O-29:4;O	C35H62NO10P	[M+Cl]-
	722.3958	0.003	4.0144	PE O-34:9	C39H62NO7P	[M+Cl]-
722.3928540						
723.3157865	723.3187	0.003	4.0093	SQDG 25:4	C34H56O12S	[M+Cl]-
	723.3515	0.002	2.9032	PI O-28:8	C37H57O12P	[M-H]-
723.3535565						
723.3535565	723.3515	0.002	2.9032	PG 30:8	C36H55O10P	[M+Formate]-
	723.3515	0.002	2.9032	PG 29:8	C35H53O10P	[M+OAc]-
723.3535565						
723.4027746	723.4031	0.000	0.5529	PA 39:11	C42H61O8P	[M-H]-
	723.4033	0.001	0.6912	TG 40:11;O	C43H60O7	[M+Cl]-
723.4027746						
724.3770270	724.3751	0.002	2.7610	PC 30:8	C38H60NO8P	[M+Cl]-
	724.3751	0.002	2.7610	PE 33:8	C38H60NO8P	[M+Cl]-
724.3770270						
724.3770270	724.3751	0.002	2.7610	PE O-33:9;O	C38H60NO8P	[M+Cl]-
	724.3831	0.000	0.1380	PS 31:6;O	C37H60NO11P	[M-H]-
724.3830579						
724.3830579	724.3833	0.000	0.2761	ST 30:4;O5;HexNAc	C38H59NO10	[M+Cl]-
	724.3831	0.000	0.1380	LPS 30:7	C36H58NO9P	[M+Formate]-
724.3830579						
724.3830579	724.3831	0.000	0.1380	PC 28:7;O	C36H58NO9P	[M+Formate]-
	724.3831	0.000	0.1380	PE 31:7;O	C36H58NO9P	[M+Formate]-
724.3830579						
724.3830579	724.3831	0.000	0.1380	PS O-30:7	C36H58NO9P	[M+Formate]-
	724.3831	0.000	0.1380	LPS 29:7	C35H56NO9P	[M+OAc]-
724.3830579						
724.3830579	724.3831	0.000	0.1380	PC 27:7;O	C35H56NO9P	[M+OAc]-
	724.3831	0.000	0.1380	PE 30:7;O	C35H56NO9P	[M+OAc]-
724.3830579						
724.3830579	724.3831	0.000	0.1380	PS O-29:7	C35H56NO9P	[M+OAc]-
	725.4166	0.001	0.9650	BMP 30:2	C36H67O10P	[M+Cl]-
725.4173266						
725.4173266	725.4166	0.001	0.9650	LPG 30:3;O	C36H67O10P	[M+Cl]-
	725.4166	0.001	0.9650	PG 30:2	C36H67O10P	[M+Cl]-
725.4173266						
725.4173266	725.4166	0.001	0.9650	PG O-30:3;O	C36H67O10P	[M+Cl]-

	726.3755	0.000	0.4130	IPC 28:3;O2	C34H62NO11P	[M+Cl]-
726.3757596						
726.3757596	726.3755	0.000	0.4130	PS 28:2;O	C34H62NO11P	[M+Cl]-
	726.3859	0.002	2.2027	ST 30:7;O4;HexNAc	C38H53NO9	[M+OAc]-
726.3843296						
727.3672414	727.3675	0.000	0.4124	LPI 23:2;O	C32H59O13P	[M+Formate]-
	727.3675	0.000	0.4124	PI 23:1	C32H59O13P	[M+Formate]-
727.3672414						
727.3672414	727.3675	0.000	0.4124	PI O-23:2;O	C32H59O13P	[M+Formate]-
	727.3675	0.000	0.4124	LPI 22:2;O	C31H57O13P	[M+OAc]-
727.3672414						
727.3672414	727.3675	0.000	0.4124	PI 22:1	C31H57O13P	[M+OAc]-
	727.3675	0.000	0.4124	PI O-22:2;O	C31H57O13P	[M+OAc]-
727.3672414						
728.2959266	728.2972	0.001	1.7850	PS 29:8;O	C35H52NO11P	[M+Cl]-
	728.3933	0.000	0.1373	PE 35:10;O	C40H60NO9P	[M-H]-
728.3934316						
729.3263973	729.3257	0.001	0.9598	PI 26:6;O	C35H55O14P	[M-H]-
	729.3259	0.001	0.6856	ST 30:4;O7;GlcA	C36H54O13	[M+Cl]-
729.3263973						
729.3263973	729.3259	0.001	0.6856	ST 30:5;O8;Hex	C36H54O13	[M+Cl]-
	729.3257	0.001	0.9598	LPI 25:7	C34H53O12P	[M+Formate]-
729.3263973						
729.3263973	729.3257	0.001	0.9598	PI O-25:7	C34H53O12P	[M+Formate]-
	730.5392	0.003	3.9697	CerP 40:2;O4	C40H78NO8P	[M-H]-
730.5420894						
730.5420894	730.5392	0.003	3.9697	LPC 32:2;O	C40H78NO8P	[M-H]-
	730.5392	0.003	3.9697	LPS O-34:2	C40H78NO8P	[M-H]-
730.5420894						
730.5420894	730.5392	0.003	3.9697	PE 35:1	C40H78NO8P	[M-H]-
	730.5392	0.003	3.9697	PE O-35:2;O	C40H78NO8P	[M-H]-
730.5420894						
730.5420894	730.5394	0.003	3.6959	CAR 34:2;O3	C41H77NO7	[M+Cl]-
	730.5394	0.003	3.6959	Cer 41:3;O6	C41H77NO7	[M+Cl]-
730.5420894						
730.5420894	730.5392	0.003	3.9697	CerP 39:2;O2	C39H76NO6P	[M+Formate]-
	730.5392	0.003	3.9697	LPC O-31:3	C39H76NO6P	[M+Formate]-

730.5420894						
730.5420894	730.5392	0.003	3.9697	LPE O-34:3	C39H76NO6P	[M+Formate]-
	730.5392	0.003	3.9697	CerP 38:2;O2	C38H74NO6P	[M+OAc]-
730.5420894						
730.5420894	730.5392	0.003	3.9697	LPC O-30:3	C38H74NO6P	[M+OAc]-
	730.5392	0.003	3.9697	LPE O-33:3	C38H74NO6P	[M+OAc]-
730.5420894						
730.5420894	730.5392	0.003	3.9697	PC 33:1	C41H80NO8P	[M-CH3]-
	730.5392	0.003	3.9697	PC O-33:2;O	C41H80NO8P	[M-CH3]-
730.5420894						
731.3073175	731.3051	0.002	3.0083	ST 29:4;O8;GlcA	C35H52O14	[M+Cl]-
	731.3049	0.002	3.2818	PI 24:6	C33H51O13P	[M+Formate]-
731.3073175						
731.3073175	731.3049	0.002	3.2818	PI 23:6	C32H49O13P	[M+OAc]-
	731.5468	0.001	1.5037	TG 41:4;O2	C44H76O8	[M-H]-
731.5456022						
731.5456022	731.5468	0.001	1.5037	TG O-41:5;O3	C44H76O8	[M-H]-
	731.5468	0.001	1.5037	CE 16:1;O4	C43H74O6	[M+Formate]-
731.5456022						
731.5456022	731.5468	0.001	1.5037	DG 40:5;O	C43H74O6	[M+Formate]-
	731.5468	0.001	1.5037	DG O-40:6;O2	C43H74O6	[M+Formate]-
731.5456022						
731.5456022	731.5468	0.001	1.5037	TG 40:4	C43H74O6	[M+Formate]-
	731.5468	0.001	1.5037	TG O-40:5;O	C43H74O6	[M+Formate]-
731.5456022						
731.5456022	731.5468	0.001	1.5037	CE 15:1;O4	C42H72O6	[M+OAc]-
	731.5468	0.001	1.5037	DG 39:5;O	C42H72O6	[M+OAc]-
731.5456022						
731.5456022	731.5468	0.001	1.5037	DG O-39:6;O2	C42H72O6	[M+OAc]-
	731.5468	0.001	1.5037	TG 39:4	C42H72O6	[M+OAc]-
731.5456022						
731.5456022	731.5468	0.001	1.5037	TG O-39:5;O	C42H72O6	[M+OAc]-
	732.3518	0.002	2.4578	PS 32:9;O	C38H56NO11P	[M-H]-
732.3535872						
733.3243375	733.3208	0.004	4.9092	ST 29:3;O8;GlcA	C35H54O14	[M+Cl]-
	733.438	0.001	1.9088	ST 30:0;O7;Hex	C36H64O12	[M+Formate]-
733.4366311						
733.4366311	733.438	0.001	1.9088	ST 29:0;O7;Hex	C35H62O12	[M+OAc]-

734.3668065	734.3675	0.001	0.9532	PS 32:8;O	C38H58NO11P	[M-H]-
734.3668065	734.3675	0.001	0.9532	PE 32:9;O	C37H56NO9P	[M+Formate]-
735.3261178	735.3282	0.002	2.8559	LPI 26:6	C35H57O12P	[M+Cl]-
735.3261178	735.3282	0.002	2.8559	LPI O-26:7;O	C35H57O12P	[M+Cl]-
735.3261178	735.3282	0.002	2.8559	PI O-26:6	C35H57O12P	[M+Cl]-
735.3261178	735.3234	0.003	3.8078	ST 30:6;O7;GlcA	C36H50O13	[M+Formate]-
735.3261178	735.3234	0.003	3.8078	ST 30:7;O8;Hex	C36H50O13	[M+Formate]-
735.3261178	735.3234	0.003	3.8078	ST 29:6;O7;GlcA	C35H48O13	[M+OAc]-
735.3261178	735.3234	0.003	3.8078	ST 29:7;O8;Hex	C35H48O13	[M+OAc]-
735.3632364	735.3631	0.000	0.1360	SQDG 25:3	C34H58O12S	[M+Formate]-
735.3632364	735.3631	0.000	0.1360	SQDG 24:3	C33H56O12S	[M+OAc]-
735.4844749	735.4842	0.000	0.4079	TG 42:9;O2	C45H68O8	[M-H]-
735.4844749	735.4842	0.000	0.4079	TG O-42:10;O3	C45H68O8	[M-H]-
735.4844749	735.4849	0.001	0.6798	EPC 36:3;O3	C38H73N2O7P	[M+Cl]-
735.4844749	735.4849	0.001	0.6798	SM 33:3;O3	C38H73N2O7P	[M+Cl]-
735.4844749	735.4842	0.000	0.4079	DG 41:10;O	C44H66O6	[M+Formate]-
735.4844749	735.4842	0.000	0.4079	DG O-41:11;O2	C44H66O6	[M+Formate]-
735.4844749	735.4842	0.000	0.4079	TG 41:9	C44H66O6	[M+Formate]-
735.4844749	735.4842	0.000	0.4079	TG O-41:10;O	C44H66O6	[M+Formate]-
735.4844749	735.4842	0.000	0.4079	DG 40:10;O	C43H64O6	[M+OAc]-
735.4844749	735.4842	0.000	0.4079	DG O-40:11;O2	C43H64O6	[M+OAc]-
735.4844749	735.4842	0.000	0.4079	TG 40:9	C43H64O6	[M+OAc]-
735.4844749	735.4842	0.000	0.4079	TG O-40:10;O	C43H64O6	[M+OAc]-
736.3243477	736.3234	0.001	1.2223	IPC 28:6;O3	C34H56NO12P	[M+Cl]-
736.3846869	736.3831	0.002	2.1728	PS 32:7;O	C38H60NO11P	[M-H]-
736.3846869	736.3831	0.002	2.1728	PC 29:8;O	C37H58NO9P	[M+Formate]-

736.3846869	736.3831	0.002	2.1728	PE 32:8;O	C37H58NO9P	[M+Formate]-
736.3846869	736.3831	0.002	2.1728	PS O-31:8	C37H58NO9P	[M+Formate]-
736.3846869	736.3831	0.002	2.1728	PC 28:8;O	C36H56NO9P	[M+OAc]-
736.3846869	736.3831	0.002	2.1728	PE 31:8;O	C36H56NO9P	[M+OAc]-
736.3846869	736.3831	0.002	2.1728	PS O-30:8	C36H56NO9P	[M+OAc]-
737.3069934	737.3074	0.000	0.5425	LPI 25:6;O	C34H55O13P	[M+Cl]-
737.3069934	737.3074	0.000	0.5425	PI 25:5	C34H55O13P	[M+Cl]-
737.3069934	737.3074	0.000	0.5425	PI O-25:6;O	C34H55O13P	[M+Cl]-
737.3849323	737.3883	0.003	4.4753	PI 26:2;O	C35H63O14P	[M-H]-
737.3849323	737.3885	0.004	4.7465	ST 30:0;O7;GlcA	C36H62O13	[M+Cl]-
737.3849323	737.3885	0.004	4.7465	ST 30:1;O8;Hex	C36H62O13	[M+Cl]-
737.3849323	737.3883	0.003	4.4753	LPI 25:3	C34H61O12P	[M+Formate]-
737.3849323	737.3883	0.003	4.4753	LPI O-25:4;O	C34H61O12P	[M+Formate]-
737.3849323	737.3883	0.003	4.4753	PI O-25:3	C34H61O12P	[M+Formate]-
737.3849323	737.3883	0.003	4.4753	LPI 24:3	C33H59O12P	[M+OAc]-
737.3849323	737.3883	0.003	4.4753	LPI O-24:4;O	C33H59O12P	[M+OAc]-
737.3849323	737.3883	0.003	4.4753	PI O-24:3	C33H59O12P	[M+OAc]-
737.4976846	737.4974	0.000	0.4068	BMP 32:0;O	C38H75O11P	[M-H]-
737.4976846	737.4974	0.000	0.4068	LPI O-29:1	C38H75O11P	[M-H]-
737.4976846	737.4974	0.000	0.4068	PG 32:0;O	C38H75O11P	[M-H]-
737.4976846	737.4976	0.000	0.1356	MGDG 30:0	C39H74O10	[M+Cl]-
737.4976846	737.4974	0.000	0.4068	LPG 31:1	C37H73O9P	[M+Formate]-
737.4976846	737.4974	0.000	0.4068	LPG O-31:2;O	C37H73O9P	[M+Formate]-
737.4976846	737.4974	0.000	0.4068	PA 34:0;O	C37H73O9P	[M+Formate]-
	737.4974	0.000	0.4068	PG O-31:1	C37H73O9P	[M+Formate]-

737.4976846						
737.4976846	737.4974	0.000	0.4068	LPG 30:1	C36H71O9P	[M+OAc]-
	737.4974	0.000	0.4068	LPG O-30:2;O	C36H71O9P	[M+OAc]-
737.4976846						
737.4976846	737.4974	0.000	0.4068	PA 33:0;O	C36H71O9P	[M+OAc]-
	737.4974	0.000	0.4068	PG O-30:1	C36H71O9P	[M+OAc]-
737.4976846						
737.5009255	737.5006	0.000	0.4068	EPC 36:2;O3	C38H75N2O7P	[M+Cl]-
	737.5006	0.000	0.4068	SM 33:2;O3	C38H75N2O7P	[M+Cl]-
737.5009255						
739.3545183	739.3547	0.000	0.1353	DGDG 22:6	C37H56O15	[M-H]-
	739.3547	0.000	0.1353	ST 30:4;O7;GlcA	C36H54O13	[M+Formate]-
739.3545183						
739.3545183	739.3547	0.000	0.1353	ST 30:5;O8;Hex	C36H54O13	[M+Formate]-
	739.3547	0.000	0.1353	ST 29:4;O7;GlcA	C35H52O13	[M+OAc]-
739.3545183						
739.3545183	739.3547	0.000	0.1353	ST 29:5;O8;Hex	C35H52O13	[M+OAc]-
	739.3677	0.000	0.2705	ST 29:0;O8;GlcA	C35H60O14	[M+Cl]-
739.3675526						
739.3675526	739.3675	0.000	0.0000	LPI 24:3;O	C33H59O13P	[M+Formate]-
	739.3675	0.000	0.0000	PI 24:2	C33H59O13P	[M+Formate]-
739.3675526						
739.3675526	739.3675	0.000	0.0000	PI O-24:3;O	C33H59O13P	[M+Formate]-
	739.3675	0.000	0.0000	LPI 23:3;O	C32H57O13P	[M+OAc]-
739.3675526						
739.3675526	739.3675	0.000	0.0000	PI 23:2	C32H57O13P	[M+OAc]-
	739.3675	0.000	0.0000	PI O-23:3;O	C32H57O13P	[M+OAc]-
739.3675526						
739.5556861	739.5526	0.003	4.1917	EPC 37:0;O2	C39H81N2O6P	[M+Cl]-
	739.5526	0.003	4.1917	SM 34:0;O2	C39H81N2O6P	[M+Cl]-
739.5556861						
740.3438160	740.3452	0.001	1.8910	SHexCer 28:4;O3	C34H59NO12S	[M+Cl]-
	740.3418	0.002	2.7015	ST 29:5;O7;HexNAc	C37H55NO12	[M+Cl]-
740.3438160						
740.3789567	740.3782	0.001	0.9455	ST 30:4;O6;HexNAc	C38H59NO11	[M+Cl]-
	740.378	0.001	1.2156	LPS 30:7;O	C36H58NO10P	[M+Formate]-
740.3789567						
740.3789567	740.378	0.001	1.2156	PS 30:6	C36H58NO10P	[M+Formate]-

740.3789567	740.378	0.001	1.2156	PS O-30:7;O	C36H58NO10P	[M+Formate]-
740.3789567	740.378	0.001	1.2156	LPS 29:7;O	C35H56NO10P	[M+OAc]-
740.3789567	740.378	0.001	1.2156	PS 29:6	C35H56NO10P	[M+OAc]-
740.3789567	740.378	0.001	1.2156	PS O-29:7;O	C35H56NO10P	[M+OAc]-
740.3812817	740.3816	0.000	0.4052	SHexCer 29:3;O2	C35H63NO11S	[M+Cl]-
741.3750957	741.3751	0.000	0.0000	LPI 26:3	C35H63O12P	[M+Cl]-
741.3750957	741.3751	0.000	0.0000	LPI O-26:4;O	C35H63O12P	[M+Cl]-
741.3750957	741.3751	0.000	0.0000	PI O-26:3	C35H63O12P	[M+Cl]-
743.3449452	743.3413	0.004	4.8430	PI 27:6;O	C36H57O14P	[M-H]-
743.3449452	743.3413	0.004	4.8430	LPI 26:7	C35H55O12P	[M+Formate]-
743.3449452	743.3413	0.004	4.8430	PI O-26:7	C35H55O12P	[M+Formate]-
743.3449452	743.3413	0.004	4.8430	LPI 25:7	C34H53O12P	[M+OAc]-
743.3449452	743.3413	0.004	4.8430	PI O-25:7	C34H53O12P	[M+OAc]-
744.4136770	744.4129	0.001	1.0747	SHexCer 29:1;O2	C35H67NO11S	[M+Cl]-
745.3240511	745.3208	0.003	4.4276	ST 30:4;O8;GlcA	C36H54O14	[M+Cl]-
745.3240511	745.3206	0.004	4.6960	LPI 25:7;O	C34H53O13P	[M+Formate]-
745.3240511	745.3206	0.004	4.6960	PI 25:6	C34H53O13P	[M+Formate]-
745.3240511	745.3206	0.004	4.6960	PI O-25:7;O	C34H53O13P	[M+Formate]-
745.3240511	745.3206	0.004	4.6960	PI 24:6	C33H51O13P	[M+OAc]-
747.3761426	747.3726	0.004	4.6831	PI 27:4;O	C36H61O14P	[M-H]-
747.3761426	747.3798	0.004	4.9506	PA 38:10	C41H61O8P	[M+Cl]-
747.3761426	747.3726	0.004	4.6831	LPI 26:5	C35H59O12P	[M+Formate]-
747.3761426	747.3726	0.004	4.6831	LPI O-26:6;O	C35H59O12P	[M+Formate]-
747.3761426	747.3726	0.004	4.6831	PI O-26:5	C35H59O12P	[M+Formate]-
747.3761426	747.3726	0.004	4.6831	LPI 25:5	C34H57O12P	[M+OAc]-
747.3761426	747.3726	0.004	4.6831	LPI O-25:6;O	C34H57O12P	[M+OAc]-

	747.3726	0.004	4.6831	PI O-25:5	C34H57O12P	[M+OAc]-
747.3761426						
747.4148017	747.4162	0.001	1.8731	PA O-39:10	C42H65O7P	[M+Cl]-
	747.57	0.002	2.0065	CE 19:1;O3	C46H80O5	[M+Cl]-
747.5685017						
747.5685017	747.57	0.002	2.0065	DG 43:5	C46H80O5	[M+Cl]-
	747.57	0.002	2.0065	DG O-43:6;O	C46H80O5	[M+Cl]-
747.5685017						
747.5685017	747.57	0.002	2.0065	TG O-43:5	C46H80O5	[M+Cl]-
	747.57	0.002	2.1403	CE 19:1;O3	C46H80O5	[M+Cl]-
747.5715463						
747.5715463	747.57	0.002	2.1403	DG 43:5	C46H80O5	[M+Cl]-
	747.57	0.002	2.1403	DG O-43:6;O	C46H80O5	[M+Cl]-
747.5715463						
747.5715463	747.57	0.002	2.1403	TG O-43:5	C46H80O5	[M+Cl]-
	748.3751	0.003	4.0087	PE 35:10	C40H60NO8P	[M+Cl]-
748.3720922						
748.5724029	748.5733	0.001	1.2023	ACer 44:4;O6	C44H79NO8	[M-H]-
	748.5733	0.001	1.2023	HexCer 38:4;O2	C44H79NO8	[M-H]-
748.5724029						
748.5724029	748.5733	0.001	1.2023	ACer 43:4;O4	C43H77NO6	[M+Formate]-
	748.5733	0.001	1.2023	Cer 43:5;O5	C43H77NO6	[M+Formate]-
748.5724029						
748.5724029	748.5733	0.001	1.2023	ACer 42:4;O4	C42H75NO6	[M+OAc]-
	748.5733	0.001	1.2023	Cer 42:5;O5	C42H75NO6	[M+OAc]-
748.5724029						
749.3437239	749.3438	0.000	0.1335	LPI 27:6	C36H59O12P	[M+Cl]-
	749.3438	0.000	0.1335	LPI O-27:7;O	C36H59O12P	[M+Cl]-
749.3437239						
749.3437239	749.3438	0.000	0.1335	PI O-27:6	C36H59O12P	[M+Cl]-
	749.3914	0.000	0.2669	EPC 34:6;O6	C36H63N2O10P	[M+Cl]-
749.3916612						
749.3916612	749.3914	0.000	0.2669	SM 31:6;O6	C36H63N2O10P	[M+Cl]-
	749.4319	0.001	1.0675	PA O-39:9	C42H67O7P	[M+Cl]-
749.4310781						
750.3486921	750.3471	0.002	2.1323	IPC 28:5;O6	C34H58NO15P	[M-H]-
	753.4947	0.000	0.3981	TG 42:8;O3	C45H70O9	[M-H]-

753.4943844						
753.4943844	753.4947	0.000	0.3981	DG 41:9;O2	C44H68O7	[M+Formate]-
	753.4947	0.000	0.3981	TG 41:8;O	C44H68O7	[M+Formate]-
753.4943844						
753.4943844	753.4947	0.000	0.3981	TG O-41:9;O2	C44H68O7	[M+Formate]-
	753.4947	0.000	0.3981	DG 40:9;O2	C43H66O7	[M+OAc]-
753.4943844						
753.4943844	753.4947	0.000	0.3981	TG 40:8;O	C43H66O7	[M+OAc]-
	753.4947	0.000	0.3981	TG O-40:9;O2	C43H66O7	[M+OAc]-
753.4943844						
755.3544457	755.3544	0.000	0.1324	LPI 26:4;O	C35H61O13P	[M+Cl]-
	755.3544	0.000	0.1324	PI 26:3	C35H61O13P	[M+Cl]-
755.3544457						
755.3544457	755.3544	0.000	0.1324	PI O-26:4;O	C35H61O13P	[M+Cl]-
	755.3626	0.003	3.7068	DGDG 20:2	C35H60O15	[M+Cl]-
755.3654530						
755.3654530	755.3625	0.003	3.9716	PI 24:2;O	C33H59O14P	[M+Formate]-
	755.3625	0.003	3.9716	PI 23:2;O	C32H57O14P	[M+OAc]-
755.3654530						
756.3259477	756.3285	0.003	3.4377	PS 31:8;O	C37H56NO11P	[M+Cl]-
	756.3368	0.001	0.9255	ST 29:5;O8;HexNAc	C37H55NO13	[M+Cl]-
756.3374274						
756.3422707	756.3401	0.002	2.7765	SHexCer 28:4;O4	C34H59NO13S	[M+Cl]-
	756.4246	0.001	0.7932	PE 37:10;O	C42H64NO9P	[M-H]-
756.4240341						
756.4240341	756.4246	0.001	0.7932	PS O-36:10	C42H64NO9P	[M-H]-
	756.4246	0.001	0.7932	PC 35:10;O	C43H66NO9P	[M-CH3]-
756.4240341						
757.3455922	757.3475	0.002	2.5088	SQDG 27:6	C36H56O12S	[M+Formate]-
	757.3475	0.002	2.5088	SQDG 26:6	C35H54O12S	[M+OAc]-
757.3455922						
757.3846510	757.3853	0.001	0.7922	BMP 33:7	C39H63O10P	[M+Cl]-
	757.3853	0.001	0.7922	PG 33:7	C39H63O10P	[M+Cl]-
757.3846510						
757.3846510	757.3853	0.001	0.7922	PG O-33:8;O	C39H63O10P	[M+Cl]-
	758.3558	0.000	0.3956	SHexCer 28:3;O4	C34H61NO13S	[M+Cl]-
758.3554737						
758.5733422	758.5705	0.003	3.6912	CerP 42:2;O4	C42H82NO8P	[M-H]-

	758.5705	0.003	3.6912	LPC 34:2;O	C42H82NO8P	[M-H]-
758.5733422						
758.5733422	758.5705	0.003	3.6912	PE 37:1	C42H82NO8P	[M-H]-
	758.5705	0.003	3.6912	PE O-37:2;O	C42H82NO8P	[M-H]-
758.5733422						
758.5733422	758.5707	0.003	3.4275	ACer 43:2;O5	C43H81NO7	[M+Cl]-
	758.5707	0.003	3.4275	Cer 43:3;O6	C43H81NO7	[M+Cl]-
758.5733422						
758.5733422	758.5705	0.003	3.6912	CerP 41:2;O2	C41H80NO6P	[M+Formate]-
	758.5705	0.003	3.6912	LPC O-33:3	C41H80NO6P	[M+Formate]-
758.5733422						
758.5733422	758.5705	0.003	3.6912	CerP 40:2;O2	C40H78NO6P	[M+OAc]-
	758.5705	0.003	3.6912	LPC O-32:3	C40H78NO6P	[M+OAc]-
758.5733422						
758.5733422	758.5705	0.003	3.6912	PC 35:1	C43H84NO8P	[M-CH3]-
	758.5705	0.003	3.6912	PC O-35:2;O	C43H84NO8P	[M-CH3]-
758.5733422						
759.3381405	759.3362	0.002	2.5022	LPI 26:7;O	C35H55O13P	[M+Formate]-
	759.3362	0.002	2.5022	PI 26:6	C35H55O13P	[M+Formate]-
759.3381405						
759.3381405	759.3362	0.002	2.5022	PI O-26:7;O	C35H55O13P	[M+Formate]-
	759.3362	0.002	2.5022	LPI 25:7;O	C34H53O13P	[M+OAc]-
759.3381405						
759.3381405	759.3362	0.002	2.5022	PI 25:6	C34H53O13P	[M+OAc]-
	759.3362	0.002	2.5022	PI O-25:7;O	C34H53O13P	[M+OAc]-
759.3381405						
760.3726280	760.3714	0.001	1.5782	SHexCer 28:2;O4	C34H63NO13S	[M+Cl]-
	761.4166	0.001	1.0507	BMP 33:5	C39H67O10P	[M+Cl]-
761.4157882						
761.4157882	761.4166	0.001	1.0507	LPG 33:6;O	C39H67O10P	[M+Cl]-
	761.4166	0.001	1.0507	PG 33:5	C39H67O10P	[M+Cl]-
761.4157882						
761.4157882	761.4166	0.001	1.0507	PG O-33:6;O	C39H67O10P	[M+Cl]-
	761.4723	0.000	0.3940	EPC 35:4;O5	C37H69N2O9P	[M+Formate]-
761.4725649						
761.4725649	761.4723	0.000	0.3940	SM 32:4;O5	C37H69N2O9P	[M+Formate]-
	761.4723	0.000	0.3940	EPC 34:4;O5	C36H67N2O9P	[M+OAc]-
761.4725649						
761.4725649	761.4723	0.000	0.3940	SM 31:4;O5	C36H67N2O9P	[M+OAc]-

	761.5856	0.001	1.5757	CE 20:1;O3	C47H82O5	[M+Cl]-
761.5843851						
761.5843851	761.5856	0.001	1.5757	DG 44:5	C47H82O5	[M+Cl]-
	761.5856	0.001	1.5757	DG O-44:6;O	C47H82O5	[M+Cl]-
761.5843851						
761.5843851	761.5856	0.001	1.5757	TG O-44:5	C47H82O5	[M+Cl]-
	762.5443	0.001	1.7048	CerP 44:6;O3	C44H78NO7P	[M-H]-
762.5430084						
762.5430084	762.5443	0.001	1.7048	PE O-39:6	C44H78NO7P	[M-H]-
	762.5445	0.002	1.9671	ACer 45:6;O4	C45H77NO6	[M+Cl]-
762.5430084						
762.5430084	762.5443	0.001	1.7048	PC O-37:6	C45H80NO7P	[M-CH3]-
	763.4322	0.000	0.3930	BMP 33:4	C39H69O10P	[M+Cl]-
763.4319444						
763.4319444	763.4322	0.000	0.3930	LPG 33:5;O	C39H69O10P	[M+Cl]-
	763.4322	0.000	0.3930	PG 33:4	C39H69O10P	[M+Cl]-
763.4319444						
763.4319444	763.4322	0.000	0.3930	PG O-33:5;O	C39H69O10P	[M+Cl]-
	764.378	0.002	2.4857	PS 32:8	C38H58NO10P	[M+Formate]-
764.3761267						
764.3761267	764.378	0.002	2.4857	PS O-32:9;O	C38H58NO10P	[M+Formate]-
	764.378	0.002	2.4857	PS 31:8	C37H56NO10P	[M+OAc]-
764.3761267						
764.4371645	764.4356	0.002	2.0930	IPC 31:3;O4	C37H68NO13P	[M-H]-
	764.4357	0.001	1.8314	HexCer 32:4;O6	C38H67NO12	[M+Cl]-
764.4371645						
764.4371645	764.4357	0.001	1.8314	ST 30:0;O7;HexNAc	C38H67NO12	[M+Cl]-
	764.4356	0.002	2.0930	IPC 30:3;O2	C36H66NO11P	[M+Formate]-
764.4371645						
764.4371645	764.4356	0.002	2.0930	PS 30:2;O	C36H66NO11P	[M+Formate]-
	764.4356	0.002	2.0930	IPC 29:3;O2	C35H64NO11P	[M+OAc]-
764.4371645						
764.4371645	764.4356	0.002	2.0930	PS 29:2;O	C35H64NO11P	[M+OAc]-
	764.4428	0.000	0.3924	PC O-34:9	C42H68NO7P	[M+Cl]-
764.4424044						
764.4424044	764.4428	0.000	0.3924	PE O-37:9	C42H68NO7P	[M+Cl]-
	764.5447	0.002	3.1391	CerP 40:1;O6	C40H80NO10P	[M-H]-

764.5471111						
764.5471111	764.5447	0.002	3.1391	LPS 34:0;O	C40H80NO10P	[M-H]-
	764.5447	0.002	3.1391	PS O-34:0;O	C40H80NO10P	[M-H]-
764.5471111						
764.5471111	764.5449	0.002	2.8775	HexCer 35:1;O3	C41H79NO9	[M+Cl]-
	764.5447	0.002	3.1391	CerP 39:1;O4	C39H78NO8P	[M+Formate]-
764.5471111						
764.5471111	764.5447	0.002	3.1391	LPC 31:1;O	C39H78NO8P	[M+Formate]-
	764.5447	0.002	3.1391	LPE 34:1;O	C39H78NO8P	[M+Formate]-
764.5471111						
764.5471111	764.5447	0.002	3.1391	LPS O-33:1	C39H78NO8P	[M+Formate]-
	764.5447	0.002	3.1391	PC 31:0	C39H78NO8P	[M+Formate]-
764.5471111						
764.5471111	764.5447	0.002	3.1391	PC O-31:1;O	C39H78NO8P	[M+Formate]-
	764.5447	0.002	3.1391	PE 34:0	C39H78NO8P	[M+Formate]-
764.5471111						
764.5471111	764.5447	0.002	3.1391	PE O-34:1;O	C39H78NO8P	[M+Formate]-
	764.5447	0.002	3.1391	CerP 38:1;O4	C38H76NO8P	[M+OAc]-
764.5471111						
764.5471111	764.5447	0.002	3.1391	LPC 30:1;O	C38H76NO8P	[M+OAc]-
	764.5447	0.002	3.1391	LPE 33:1;O	C38H76NO8P	[M+OAc]-
764.5471111						
764.5471111	764.5447	0.002	3.1391	LPS O-32:1	C38H76NO8P	[M+OAc]-
	764.5447	0.002	3.1391	PC 30:0	C38H76NO8P	[M+OAc]-
764.5471111						
764.5471111	764.5447	0.002	3.1391	PC O-30:1;O	C38H76NO8P	[M+OAc]-
	764.5447	0.002	3.1391	PE 33:0	C38H76NO8P	[M+OAc]-
764.5471111						
764.5471111	764.5447	0.002	3.1391	PE O-33:1;O	C38H76NO8P	[M+OAc]-
	765.3387	0.002	2.4826	LPI 27:6;O	C36H59O13P	[M+Cl]-
765.3368662						
765.3368662	765.3387	0.002	2.4826	PI 27:5	C36H59O13P	[M+Cl]-
	765.3387	0.002	2.4826	PI O-27:6;O	C36H59O13P	[M+Cl]-
765.3368662						
766.3831717	766.3856	0.003	3.2621	PC 32:9;O	C40H62NO9P	[M+Cl]-
	766.3856	0.003	3.2621	PE 35:9;O	C40H62NO9P	[M+Cl]-
766.3831717						
766.3831717	766.3856	0.003	3.2621	PS O-34:9	C40H62NO9P	[M+Cl]-

767.3165278	767.318	0.002	1.9549	PI 26:5;O	C35H57O14P	[M+Cl]-
769.3947211	769.3934	0.001	1.8196	LPI 30:7;O	C39H63O13P	[M-H]-
769.3947211	769.3934	0.001	1.8196	PI 30:6	C39H63O13P	[M-H]-
769.3947211	769.3934	0.001	1.8196	PI O-30:7;O	C39H63O13P	[M-H]-
769.3947211	769.3934	0.001	1.8196	BMP 32:7;O	C38H61O11P	[M+Formate]-
769.3947211	769.3934	0.001	1.8196	PG 32:7;O	C38H61O11P	[M+Formate]-
769.3947211	769.3934	0.001	1.8196	BMP 31:7;O	C37H59O11P	[M+OAc]-
769.3947211	769.3934	0.001	1.8196	PG 31:7;O	C37H59O11P	[M+OAc]-
770.4990393	770.4978	0.001	1.6872	CerP 41:5;O6	C41H74NO10P	[M-H]-
770.4990393	770.4978	0.001	1.6872	PS 35:3	C41H74NO10P	[M-H]-
770.4990393	770.4978	0.001	1.6872	PS O-35:4;O	C41H74NO10P	[M-H]-
770.4990393	770.4979	0.001	1.4276	HexCer 36:5;O3	C42H73NO9	[M+Cl]-
770.4990393	770.4978	0.001	1.6872	CerP 40:5;O4	C40H72NO8P	[M+Formate]-
770.4990393	770.4978	0.001	1.6872	LPC 32:5;O	C40H72NO8P	[M+Formate]-
770.4990393	770.4978	0.001	1.6872	LPS O-34:5	C40H72NO8P	[M+Formate]-
770.4990393	770.4978	0.001	1.6872	PC 32:4	C40H72NO8P	[M+Formate]-
770.4990393	770.4978	0.001	1.6872	PC O-32:5;O	C40H72NO8P	[M+Formate]-
770.4990393	770.4978	0.001	1.6872	PE 35:4	C40H72NO8P	[M+Formate]-
770.4990393	770.4978	0.001	1.6872	PE O-35:5;O	C40H72NO8P	[M+Formate]-
770.4990393	770.4978	0.001	1.6872	CerP 39:5;O4	C39H70NO8P	[M+OAc]-
770.4990393	770.4978	0.001	1.6872	LPC 31:5;O	C39H70NO8P	[M+OAc]-
770.4990393	770.4978	0.001	1.6872	LPE 34:5;O	C39H70NO8P	[M+OAc]-
770.4990393	770.4978	0.001	1.6872	LPS O-33:5	C39H70NO8P	[M+OAc]-
770.4990393	770.4978	0.001	1.6872	PC 31:4	C39H70NO8P	[M+OAc]-
770.4990393	770.4978	0.001	1.6872	PC O-31:5;O	C39H70NO8P	[M+OAc]-
770.4990393	770.4978	0.001	1.6872	PE 34:4	C39H70NO8P	[M+OAc]-

	770.4978	0.001	1.6872	PE O-34:5;O	C39H70NO8P	[M+OAc]-
770.4990393						
770.5011388	770.4978	0.003	4.4127	CerP 41:5;O6	C41H74NO10P	[M-H]-
	770.4978	0.003	4.4127	PS 35:3	C41H74NO10P	[M-H]-
770.5011388						
770.5011388	770.4978	0.003	4.4127	PS O-35:4;O	C41H74NO10P	[M-H]-
	770.4979	0.003	4.1532	HexCer 36:5;O3	C42H73NO9	[M+Cl]-
770.5011388						
770.5011388	770.4978	0.003	4.4127	CerP 40:5;O4	C40H72NO8P	[M+Formate]-
	770.4978	0.003	4.4127	LPC 32:5;O	C40H72NO8P	[M+Formate]-
770.5011388						
770.5011388	770.4978	0.003	4.4127	LPS O-34:5	C40H72NO8P	[M+Formate]-
	770.4978	0.003	4.4127	PC 32:4	C40H72NO8P	[M+Formate]-
770.5011388						
770.5011388	770.4978	0.003	4.4127	PC O-32:5;O	C40H72NO8P	[M+Formate]-
	770.4978	0.003	4.4127	PE 35:4	C40H72NO8P	[M+Formate]-
770.5011388						
770.5011388	770.4978	0.003	4.4127	PE O-35:5;O	C40H72NO8P	[M+Formate]-
	770.4978	0.003	4.4127	CerP 39:5;O4	C39H70NO8P	[M+OAc]-
770.5011388						
770.5011388	770.4978	0.003	4.4127	LPC 31:5;O	C39H70NO8P	[M+OAc]-
	770.4978	0.003	4.4127	LPE 34:5;O	C39H70NO8P	[M+OAc]-
770.5011388						
770.5011388	770.4978	0.003	4.4127	LPS O-33:5	C39H70NO8P	[M+OAc]-
	770.4978	0.003	4.4127	PC 31:4	C39H70NO8P	[M+OAc]-
770.5011388						
770.5011388	770.4978	0.003	4.4127	PC O-31:5;O	C39H70NO8P	[M+OAc]-
	770.4978	0.003	4.4127	PE 34:4	C39H70NO8P	[M+OAc]-
770.5011388						
770.5011388	770.4978	0.003	4.4127	PE O-34:5;O	C39H70NO8P	[M+OAc]-
	772.335	0.001	0.6474	SHexCer 28:4;O5	C34H59NO14S	[M+Cl]-
772.3355865						
773.2558447	773.2556	0.000	0.2586	PIP 21:4	C30H50O16P2	[M+Formate]-
	773.2556	0.000	0.2586	PIP 20:4	C29H48O16P2	[M+OAc]-
773.2558447						
773.3260923	773.3284	0.002	2.9742	PIP 24:2	C33H60O16P2	[M-H]-
	773.3438	0.002	2.5862	PI O-29:8	C38H59O12P	[M+Cl]-

773.3417849						
774.3483830	774.3507	0.002	2.9702	SHexCer 28:3;O5	C34H61NO14S	[M+Cl]-
	774.3507	0.001	0.9040	SHexCer 28:3;O5	C34H61NO14S	[M+Cl]-
774.3514260						
774.3628932	774.3602	0.003	3.4867	IPC 28:3;O5	C34H62NO14P	[M+Cl]-
	774.3871	0.000	0.3874	SHexCer 29:2;O4	C35H65NO13S	[M+Cl]-
774.3868088						
775.3349247	775.3313	0.004	4.6432	DGDG 22:6	C37H56O15	[M+Cl]-
	775.3312	0.004	4.9011	PI 26:6;O	C35H55O14P	[M+Formate]-
775.3349247						
775.3349247	775.3312	0.004	4.9011	PI 25:6;O	C34H53O14P	[M+OAc]-
	775.344	0.001	1.8057	PIP 24:1	C33H62O16P2	[M-H]-
775.3454548						
776.4047014	776.4064	0.002	2.1896	PE 37:10	C42H64NO8P	[M+Cl]-
	776.4027	0.002	2.5760	SHexCer 29:1;O4	C35H67NO13S	[M+Cl]-
776.4047014						
777.3870808	777.3889	0.002	2.4441	SQDG 32:8	C41H62O12S	[M-H]-
	778.3573	0.002	2.1841	PS 32:9;O	C38H56NO11P	[M+Formate]-
778.3555893						
778.4186976	778.4184	0.000	0.3854	SHexCer 29:0;O4	C35H69NO13S	[M+Cl]-
	778.422	0.000	0.3854	PC 34:9	C42H66NO8P	[M+Cl]-
778.4217154						
778.4217154	778.422	0.000	0.3854	PE 37:9	C42H66NO8P	[M+Cl]-
	778.422	0.000	0.3854	PE O-37:10;O	C42H66NO8P	[M+Cl]-
778.4217154						
778.4427869	778.4417	0.001	1.4131	SHexCer 32:3;O4	C38H69NO13S	[M-H]-
	778.4431	0.000	0.5138	CerP 39:5;O6	C39H70NO10P	[M+Cl]-
778.4427869						
778.4427869	778.4431	0.000	0.5138	LPS 33:4;O	C39H70NO10P	[M+Cl]-
	778.4431	0.000	0.5138	PS 33:3	C39H70NO10P	[M+Cl]-
778.4427869						
778.4427869	778.4431	0.000	0.5138	PS O-33:4;O	C39H70NO10P	[M+Cl]-
	778.4417	0.001	1.4131	SHexCer 31:3;O2	C37H67NO11S	[M+Formate]-
778.4427869						
778.4427869	778.4417	0.001	1.4131	SHexCer 30:3;O2	C36H65NO11S	[M+OAc]-
	779.2581	0.003	3.9781	PIP 22:3	C31H54O16P2	[M+Cl]-
779.2550429						
779.4262985	779.4272	0.001	1.1547	BMP 33:4;O	C39H69O11P	[M+Cl]-

	779.4272	0.001	1.1547	LPI O-30:5	C39H69O11P	[M+Cl]-
779.4262985						
779.4262985	779.4272	0.001	1.1547	PG 33:4;O	C39H69O11P	[M+Cl]-
	779.4257	0.001	0.7698	SQDG 28:2	C37H66O12S	[M+Formate]-
779.4262985						
779.4262985	779.4257	0.001	0.7698	SQDG 27:2	C36H64O12S	[M+OAc]-
	779.4376	0.000	0.0000	TG 41:11;O3	C44H62O9	[M+Formate]-
779.4375642						
779.4375642	779.4376	0.000	0.0000	TG 40:11;O3	C43H60O9	[M+OAc]-
	779.4657	0.001	1.5395	PA 43:11	C46H69O8P	[M-H]-
779.4645508						
779.4645508	779.4657	0.001	1.5395	PA O-43:12;O	C46H69O8P	[M-H]-
	779.4635	0.001	1.2829	BMP 34:3	C40H73O10P	[M+Cl]-
779.4645508						
779.4645508	779.4635	0.001	1.2829	LPG 34:4;O	C40H73O10P	[M+Cl]-
	779.4635	0.001	1.2829	PG 34:3	C40H73O10P	[M+Cl]-
779.4645508						
779.4645508	779.4635	0.001	1.2829	PG O-34:4;O	C40H73O10P	[M+Cl]-
	779.4659	0.001	1.7961	DG 44:12;O2	C47H68O7	[M+Cl]-
779.4645508						
779.4645508	779.4659	0.001	1.7961	TG 44:11;O	C47H68O7	[M+Cl]-
	779.4659	0.001	1.7961	TG O-44:12;O2	C47H68O7	[M+Cl]-
779.4645508						
780.3970555	780.3976	0.001	0.7688	SHexCer 28:0;O5	C34H67NO14S	[M+Cl]-
	780.4377	0.001	0.7688	PC 34:8	C42H68NO8P	[M+Cl]-
780.4370805						
780.4370805	780.4377	0.001	0.7688	PC O-34:9;O	C42H68NO8P	[M+Cl]-
	780.4377	0.001	0.7688	PE 37:8	C42H68NO8P	[M+Cl]-
780.4370805						
780.4370805	780.4377	0.001	0.7688	PE O-37:9;O	C42H68NO8P	[M+Cl]-
	781.4428	0.000	0.5119	BMP 33:3;O	C39H71O11P	[M+Cl]-
781.4424260						
781.4424260	781.4428	0.000	0.5119	LPI O-30:4	C39H71O11P	[M+Cl]-
	781.4428	0.000	0.5119	PG 33:3;O	C39H71O11P	[M+Cl]-
781.4424260						
781.4424260	781.4414	0.001	1.4077	SQDG 28:1	C37H68O12S	[M+Formate]-
	781.4414	0.001	1.4077	SQDG 27:1	C36H66O12S	[M+OAc]-
781.4424260						
783.3849888	783.3857	0.001	0.8936	LPI 28:4;O	C37H65O13P	[M+Cl]-

	783.3857	0.001	0.8936	PI 28:3	C37H65O13P	[M+Cl]-
783.3849888						
783.3849888	783.3857	0.001	0.8936	PI O-28:4;O	C37H65O13P	[M+Cl]-
	784.3714	0.002	2.0398	SHexCer 30:4;O4	C36H63NO13S	[M+Cl]-
784.3729768						
785.3659066	785.365	0.001	1.2733	PI 27:3;O	C36H63O14P	[M+Cl]-
	786.3871	0.001	0.7630	SHexCer 30:3;O4	C36H65NO13S	[M+Cl]-
786.3864810						
786.4947312	786.4927	0.002	2.5429	IPC 35:4;O2	C41H74NO11P	[M-H]-
	786.4927	0.002	2.5429	PS 35:3;O	C41H74NO11P	[M-H]-
786.4947312						
786.4947312	786.4929	0.002	2.4158	HexCer 36:5;O4	C42H73NO10	[M+Cl]-
	786.4927	0.002	2.5429	CerP 40:5;O5	C40H72NO9P	[M+Formate]-
786.4947312						
786.4947312	786.4927	0.002	2.5429	LPS 34:4	C40H72NO9P	[M+Formate]-
	786.4927	0.002	2.5429	LPS O-34:5;O	C40H72NO9P	[M+Formate]-
786.4947312						
786.4947312	786.4927	0.002	2.5429	PC 32:4;O	C40H72NO9P	[M+Formate]-
	786.4927	0.002	2.5429	PE 35:4;O	C40H72NO9P	[M+Formate]-
786.4947312						
786.4947312	786.4927	0.002	2.5429	PS O-34:4	C40H72NO9P	[M+Formate]-
	786.4927	0.002	2.5429	CerP 39:5;O5	C39H70NO9P	[M+OAc]-
786.4947312						
786.4947312	786.4927	0.002	2.5429	LPS 33:4	C39H70NO9P	[M+OAc]-
	786.4927	0.002	2.5429	LPS O-33:5;O	C39H70NO9P	[M+OAc]-
786.4947312						
786.4947312	786.4927	0.002	2.5429	PC 31:4;O	C39H70NO9P	[M+OAc]-
	786.4927	0.002	2.5429	PE 34:4;O	C39H70NO9P	[M+OAc]-
786.4947312						
786.4947312	786.4927	0.002	2.5429	PS O-33:4	C39H70NO9P	[M+OAc]-
	787.3758	0.003	3.5561	DGDG 22:5	C37H58O15	[M+Formate]-
787.3729905						
787.3729905	787.3758	0.003	3.5561	DGDG 21:5	C36H56O15	[M+OAc]-
	787.3806	0.003	3.3021	PI 27:2;O	C36H65O14P	[M+Cl]-
787.3832349						
787.4434947	787.4439	0.000	0.5080	SQDG 29:0	C38H72O12S	[M+Cl]-
	789.4196	0.004	4.9403	PI 30:4;O	C39H67O14P	[M-H]-

789.4234611						
789.4234611	789.4268	0.003	4.1802	PA 41:10	C44H67O8P	[M+Cl]-
	789.4268	0.003	4.1802	PA O-41:11;O	C44H67O8P	[M+Cl]-
789.4234611						
789.4234611	789.4196	0.004	4.9403	LPI 29:5	C38H65O12P	[M+Formate]-
	789.4196	0.004	4.9403	LPI O-29:6;O	C38H65O12P	[M+Formate]-
789.4234611						
789.4234611	789.4196	0.004	4.9403	PI O-29:5	C38H65O12P	[M+Formate]-
	789.4196	0.004	4.9403	LPI 28:5	C37H63O12P	[M+OAc]-
789.4234611						
789.4234611	789.4196	0.004	4.9403	LPI O-28:6;O	C37H63O12P	[M+OAc]-
	789.4196	0.004	4.9403	PI O-28:5	C37H63O12P	[M+OAc]-
789.4234611						
790.4069234	790.4068	0.000	0.2530	IPC 33:6;O2	C39H66NO11P	[M+Cl]-
	790.4068	0.000	0.2530	PS 33:5;O	C39H66NO11P	[M+Cl]-
790.4069234						
791.4158178	791.4141	0.002	2.1481	PI O-33:9	C42H65O12P	[M-H]-
	791.4141	0.002	2.1481	PG 35:9	C41H63O10P	[M+Formate]-
791.4158178						
791.4158178	791.4141	0.002	2.1481	PG O-35:10;O	C41H63O10P	[M+Formate]-
	791.4141	0.002	2.1481	PG 34:9	C40H61O10P	[M+OAc]-
791.4158178						
791.4382851	791.4384	0.000	0.1264	EPC 37:6;O6	C39H69N2O10P	[M+Cl]-
	791.4384	0.000	0.1264	SM 34:6;O6	C39H69N2O10P	[M+Cl]-
791.4382851						
791.4382851	791.4376	0.001	0.8845	TG 42:12;O3	C45H62O9	[M+Formate]-
	791.4635	0.001	0.6317	PG 35:4	C41H73O10P	[M+Cl]-
791.4640155						
791.4640155	791.4635	0.001	0.6317	PG O-35:5;O	C41H73O10P	[M+Cl]-
	792.3976	0.001	0.8834	SHexCer 29:1;O5	C35H67NO14S	[M+Cl]-
792.3983807						
792.4026163	792.4013	0.001	1.6406	PE 37:10;O	C42H64NO9P	[M+Cl]-
	792.4013	0.001	1.6406	PS O-36:10	C42H64NO9P	[M+Cl]-
792.4026163						
792.4235241	792.4224	0.001	1.3881	IPC 33:5;O2	C39H68NO11P	[M+Cl]-
	792.4224	0.001	1.3881	PS 33:4;O	C39H68NO11P	[M+Cl]-
792.4235241						
794.3538652	794.3558	0.002	2.3919	SHexCer 31:6;O4	C37H61NO13S	[M+Cl]-

	794.4169	0.002	2.1399	PC 34:9;O	C42H66NO9P	[M+Cl]-
794.4152141						
794.4152141	794.4169	0.002	2.1399	PE 37:9;O	C42H66NO9P	[M+Cl]-
	794.4169	0.002	2.1399	PS O-36:9	C42H66NO9P	[M+Cl]-
794.4152141						
794.4152141	794.4133	0.002	2.3917	SHexCer 29:0;O5	C35H69NO14S	[M+Cl]-
	795.3857	0.002	2.2631	LPI 29:5;O	C38H65O13P	[M+Cl]-
795.3839388						
795.3839388	795.3857	0.002	2.2631	PI 29:4	C38H65O13P	[M+Cl]-
	795.3857	0.002	2.2631	PI O-29:5;O	C38H65O13P	[M+Cl]-
795.3839388						
795.3972892	795.3939	0.003	4.2746	DGDG 23:3	C38H64O15	[M+Cl]-
	795.4009	0.004	4.6517	PG 36:9	C42H65O10P	[M+Cl]-
795.3972892						
795.3972892	795.4009	0.004	4.6517	PG O-36:10;O	C42H65O10P	[M+Cl]-
	795.3938	0.004	4.4003	PI 27:3;O	C36H63O14P	[M+Formate]-
795.3972892						
795.3972892	795.3938	0.004	4.4003	PI 26:3;O	C35H61O14P	[M+OAc]-
	796.3714	0.001	1.3813	SHexCer 31:5;O4	C37H63NO13S	[M+Cl]-
796.3725386						
796.4324574	796.4326	0.000	0.1256	PC 34:8;O	C42H68NO9P	[M+Cl]-
	796.4326	0.000	0.1256	PE 37:8;O	C42H68NO9P	[M+Cl]-
796.4324574						
796.4324574	796.4326	0.000	0.1256	PS O-36:8	C42H68NO9P	[M+Cl]-
	797.3788	0.002	2.6336	SQDG 30:7	C39H60O12S	[M+Formate]-
797.3766331						
797.3766331	797.3788	0.002	2.6336	SQDG 29:7	C38H58O12S	[M+OAc]-
	798.3871	0.001	1.2525	SHexCer 31:4;O4	C37H65NO13S	[M+Cl]-
798.3860663						
799.3452113	799.344	0.001	1.5012	PIP 26:3	C35H62O16P2	[M-H]-
	799.5366	0.000	0.2501	MGDG 38:7	C47H76O10	[M-H]-
799.5367912						
799.5367912	799.5374	0.001	0.7504	EPC 38:1;O5	C40H81N2O9P	[M+Cl]-
	799.5374	0.001	0.7504	SM 35:1;O5	C40H81N2O9P	[M+Cl]-
799.5367912						
799.5367912	799.5366	0.000	0.2501	TG 43:7;O2	C46H74O8	[M+Formate]-
	799.5366	0.000	0.2501	TG O-43:8;O3	C46H74O8	[M+Formate]-
799.5367912						
799.5367912	799.5366	0.000	0.2501	TG 42:7;O2	C45H72O8	[M+OAc]-

799.5367912	799.5366	0.000	0.2501	TG O-42:8;O3	C45H72O8	[M+OAc]-
800.5119444	800.5083	0.004	4.4971	IPC 36:4;O2	C42H76NO11P	[M-H]-
800.5119444	800.5083	0.004	4.4971	PS 36:3;O	C42H76NO11P	[M-H]-
800.5119444	800.5085	0.003	4.2473	HexCer 37:5;O4	C43H75NO10	[M+Cl]-
800.5119444	800.5083	0.004	4.4971	CerP 41:5;O5	C41H74NO9P	[M+Formate]-
800.5119444	800.5083	0.004	4.4971	PC 33:4;O	C41H74NO9P	[M+Formate]-
800.5119444	800.5083	0.004	4.4971	PE 36:4;O	C41H74NO9P	[M+Formate]-
800.5119444	800.5083	0.004	4.4971	PS O-35:4	C41H74NO9P	[M+Formate]-
800.5119444	800.5083	0.004	4.4971	CerP 40:5;O5	C40H72NO9P	[M+OAc]-
800.5119444	800.5083	0.004	4.4971	LPS 34:4	C40H72NO9P	[M+OAc]-
800.5119444	800.5083	0.004	4.4971	LPS O-34:5;O	C40H72NO9P	[M+OAc]-
800.5119444	800.5083	0.004	4.4971	PC 32:4;O	C40H72NO9P	[M+OAc]-
800.5119444	800.5083	0.004	4.4971	PE 35:4;O	C40H72NO9P	[M+OAc]-
800.5119444	800.5083	0.004	4.4971	PS O-34:4	C40H72NO9P	[M+OAc]-
801.4235617	801.4196	0.004	4.9911	PI 31:5;O	C40H67O14P	[M-H]-
801.4235617	801.4268	0.003	3.9929	PA 42:11	C45H67O8P	[M+Cl]-
801.4235617	801.4268	0.003	3.9929	PA O-42:12;O	C45H67O8P	[M+Cl]-
801.4235617	801.4196	0.004	4.9911	LPI 30:6	C39H65O12P	[M+Formate]-
801.4235617	801.4196	0.004	4.9911	LPI O-30:7;O	C39H65O12P	[M+Formate]-
801.4235617	801.4196	0.004	4.9911	PI O-30:6	C39H65O12P	[M+Formate]-
801.4235617	801.4196	0.004	4.9911	LPI 29:6	C38H63O12P	[M+OAc]-
801.4235617	801.4196	0.004	4.9911	LPI O-29:7;O	C38H63O12P	[M+OAc]-
801.4235617	801.4196	0.004	4.9911	PI O-29:6	C38H63O12P	[M+OAc]-
802.3563561	802.3551	0.001	1.4956	IPC 29:4;O6	C35H62NO15P	[M+Cl]-
	802.5604	0.003	4.2364	CerP 43:3;O6	C43H82NO10P	[M-H]-

802.5637336						
802.5637336	802.5604	0.003	4.2364	PS 37:1	C43H82NO10P	[M-H]-
	802.5604	0.003	4.2364	PS O-37:2;O	C43H82NO10P	[M-H]-
802.5637336						
802.5637336	802.5605	0.003	3.9872	HexCer 38:3;O3	C44H81NO9	[M+Cl]-
	802.5604	0.003	4.2364	CerP 42:3;O4	C42H80NO8P	[M+Formate]-
802.5637336						
802.5637336	802.5604	0.003	4.2364	LPC 34:3;O	C42H80NO8P	[M+Formate]-
	802.5604	0.003	4.2364	PC 34:2	C42H80NO8P	[M+Formate]-
802.5637336						
802.5637336	802.5604	0.003	4.2364	PC O-34:3;O	C42H80NO8P	[M+Formate]-
	802.5604	0.003	4.2364	PE 37:2	C42H80NO8P	[M+Formate]-
802.5637336						
802.5637336	802.5604	0.003	4.2364	PE O-37:3;O	C42H80NO8P	[M+Formate]-
	802.5604	0.003	4.2364	CerP 41:3;O4	C41H78NO8P	[M+OAc]-
802.5637336						
802.5637336	802.5604	0.003	4.2364	LPC 33:3;O	C41H78NO8P	[M+OAc]-
	802.5604	0.003	4.2364	PC 33:2	C41H78NO8P	[M+OAc]-
802.5637336						
802.5637336	802.5604	0.003	4.2364	PC O-33:3;O	C41H78NO8P	[M+OAc]-
	802.5604	0.003	4.2364	PE 36:2	C41H78NO8P	[M+OAc]-
802.5637336						
802.5637336	802.5604	0.003	4.2364	PE O-36:3;O	C41H78NO8P	[M+OAc]-
	803.4046	0.001	1.4936	SQDG 34:9	C43H64O12S	[M-H]-
803.4033497						
803.4146667	803.4141	0.001	0.7468	PG 36:10	C42H63O10P	[M+Formate]-
	803.4141	0.001	0.7468	PG 35:10	C41H61O10P	[M+OAc]-
803.4146667						
803.4383586	803.4352	0.003	3.8584	PI 31:4;O	C40H69O14P	[M-H]-
	803.4352	0.003	3.8584	LPI 30:5	C39H67O12P	[M+Formate]-
803.4383586						
803.4383586	803.4352	0.003	3.8584	LPI O-30:6;O	C39H67O12P	[M+Formate]-
	803.4352	0.003	3.8584	PI O-30:5	C39H67O12P	[M+Formate]-
803.4383586						
803.4383586	803.4352	0.003	3.8584	LPI 29:5	C38H65O12P	[M+OAc]-
	803.4352	0.003	3.8584	LPI O-29:6;O	C38H65O12P	[M+OAc]-
803.4383586						
803.4383586	803.4352	0.003	3.8584	PI O-29:5	C38H65O12P	[M+OAc]-

803.5673376	803.5679	0.001	0.6222	MGDG 38:5	C47H80O10	[M-H]-
803.5673376	803.5679	0.001	0.6222	TG 43:5;O2	C46H78O8	[M+Formate]-
803.5673376	803.5679	0.001	0.6222	TG O-43:6;O3	C46H78O8	[M+Formate]-
803.5673376	803.5679	0.001	0.6222	TG 42:5;O2	C45H76O8	[M+OAc]-
803.5673376	803.5679	0.001	0.6222	TG O-42:6;O3	C45H76O8	[M+OAc]-
805.3561583	805.3546	0.002	1.8625	PIP 25:1;O	C34H64O17P2	[M-H]-
805.4552658	805.454	0.001	1.4898	EPC 38:6;O6	C40H71N2O10P	[M+Cl]-
805.4552658	805.454	0.001	1.4898	SM 35:6;O6	C40H71N2O10P	[M+Cl]-
805.4552658	805.4532	0.002	2.4831	TG 43:12;O3	C46H64O9	[M+Formate]-
805.4552658	805.4532	0.002	2.4831	TG 42:12;O3	C45H62O9	[M+OAc]-
806.3662657	806.3638	0.002	2.9763	SHexCer 30:6;O5	C36H59NO14S	[M+Formate]-
806.3662657	806.3638	0.002	2.9763	SHexCer 29:6;O5	C35H57NO14S	[M+OAc]-
806.4146515	806.4133	0.001	1.7361	SHexCer 30:1;O5	C36H69NO14S	[M+Cl]-
807.3744945	807.3726	0.002	2.3533	PI 32:9;O	C41H61O14P	[M-H]-
807.3844372	807.3857	0.001	1.6101	LPI 30:6;O	C39H65O13P	[M+Cl]-
807.3844372	807.3857	0.001	1.6101	PI 30:5	C39H65O13P	[M+Cl]-
807.3844372	807.3857	0.001	1.6101	PI O-30:6;O	C39H65O13P	[M+Cl]-
809.4143009	809.4166	0.002	2.8416	PG 37:9	C43H67O10P	[M+Cl]-
809.4143009	809.4166	0.002	2.8416	PG O-37:10;O	C43H67O10P	[M+Cl]-
809.5221022	809.5209	0.001	1.4824	MGDG 39:9	C48H74O10	[M-H]-
809.5221022	809.5217	0.000	0.4941	EPC 39:3;O5	C41H79N2O9P	[M+Cl]-
809.5221022	809.5217	0.000	0.4941	SM 36:3;O5	C41H79N2O9P	[M+Cl]-
809.5221022	809.5209	0.001	1.4824	TG 44:9;O2	C47H72O8	[M+Formate]-
809.5221022	809.5209	0.001	1.4824	TG O-44:10;O3	C47H72O8	[M+Formate]-
809.5221022	809.5209	0.001	1.4824	TG 43:9;O2	C46H70O8	[M+OAc]-
809.5221022	809.5209	0.001	1.4824	TG O-43:10;O3	C46H70O8	[M+OAc]-

	809.5491	0.001	1.4823	PA O-46:10	C49H79O7P	[M-H]-
809.5478884						
809.5478884	809.5469	0.001	1.2353	PA 40:1;O	C43H83O9P	[M+Cl]-
	809.5469	0.001	1.2353	PG O-37:2	C43H83O9P	[M+Cl]-
809.5478884						
809.5478884	809.5492	0.001	1.7294	CE 23:6;O4	C50H78O6	[M+Cl]-
	809.5492	0.001	1.7294	DG 47:10;O	C50H78O6	[M+Cl]-
809.5478884						
809.5478884	809.5492	0.001	1.7294	DG O-47:11;O2	C50H78O6	[M+Cl]-
	809.5492	0.001	1.7294	TG 47:9	C50H78O6	[M+Cl]-
809.5478884						
809.5478884	809.5492	0.001	1.7294	TG O-47:10;O	C50H78O6	[M+Cl]-
	810.3683	0.004	4.8126	IPC 29:5;O6	C35H60NO15P	[M+Formate]-
810.3643398						
810.3643398	810.3683	0.004	4.8126	IPC 28:5;O6	C34H58NO15P	[M+OAc]-
	810.4082	0.001	1.2339	SHexCer 29:0;O6	C35H69NO15S	[M+Cl]-
810.4072220						
810.4120747	810.4118	0.000	0.2468	PS 36:8	C42H66NO10P	[M+Cl]-
	810.4118	0.000	0.2468	PS O-36:9;O	C42H66NO10P	[M+Cl]-
810.4120747						
810.6864131	810.6828	0.004	4.4407	ACer 48:1;O6	C48H93NO8	[M-H]-
	810.6828	0.004	4.4407	HexCer 42:1;O2	C48H93NO8	[M-H]-
810.6864131						
810.6864131	810.6828	0.004	4.4407	ACer 47:1;O4	C47H91NO6	[M+Formate]-
	810.6828	0.004	4.4407	Cer 47:2;O5	C47H91NO6	[M+Formate]-
810.6864131						
810.6864131	810.6828	0.004	4.4407	ACer 46:1;O4	C46H89NO6	[M+OAc]-
	810.6828	0.004	4.4407	Cer 46:2;O5	C46H89NO6	[M+OAc]-
810.6864131						
811.3833096	811.3806	0.003	3.3277	PI 29:4;O	C38H65O14P	[M+Cl]-
	811.3959	0.001	0.8627	PG 36:9;O	C42H65O11P	[M+Cl]-
811.3951452						
811.3951452	811.3944	0.001	0.8627	SQDG 31:7	C40H62O12S	[M+Formate]-
	811.3944	0.001	0.8627	SQDG 30:7	C39H60O12S	[M+OAc]-
811.3951452						
811.4435786	811.4439	0.000	0.3697	SQDG 31:2	C40H72O12S	[M+Cl]-
	812.4472	0.000	0.1231	SHexCer 32:2;O6	C38H71NO15S	[M-H]-

812.4470882						
812.4470882	812.4472	0.000	0.1231	SHexCer 31:2;O4	C37H69NO13S	[M+Formate]-
	812.4472	0.000	0.1231	SHexCer 30:2;O4	C36H67NO13S	[M+OAc]-
812.4470882						
813.3967935	813.3963	0.001	0.6147	PI 29:3;O	C38H67O14P	[M+Cl]-
	813.5076	0.001	0.8605	PA 44:9;O	C47H75O9P	[M-H]-
813.5069301						
813.5069301	813.5076	0.001	0.8605	PG O-41:10	C47H75O9P	[M-H]-
	813.5078	0.001	0.9834	TG 45:9;O2	C48H74O8	[M+Cl]-
813.5069301						
813.5069301	813.5078	0.001	0.9834	TG O-45:10;O3	C48H74O8	[M+Cl]-
	813.5054	0.002	1.8439	LPI O-32:2	C41H79O11P	[M+Cl]-
813.5069301						
813.5069301	813.5054	0.002	1.8439	PG 35:1;O	C41H79O11P	[M+Cl]-
	813.5076	0.001	0.8605	PA O-43:10	C46H73O7P	[M+Formate]-
813.5069301						
813.5069301	813.5076	0.001	0.8605	PA O-42:10	C45H71O7P	[M+OAc]-
	814.4068	0.002	1.8418	PS 35:7;O	C41H66NO11P	[M+Cl]-
814.4052794						
815.3358089	815.339	0.003	3.9247	PIP 26:3;O	C35H62O17P2	[M-H]-
	815.3908	0.003	3.4339	PI O-32:8	C41H65O12P	[M+Cl]-
815.3936127						
815.4138884	815.4119	0.002	2.4527	PI 29:2;O	C38H69O14P	[M+Cl]-
	815.4352	0.002	2.6979	PI 32:5;O	C41H69O14P	[M-H]-
815.4374342						
815.4374342	815.4352	0.002	2.6979	LPI 31:6	C40H67O12P	[M+Formate]-
	815.4352	0.002	2.6979	LPI O-31:7;O	C40H67O12P	[M+Formate]-
815.4374342						
815.4374342	815.4352	0.002	2.6979	PI O-31:6	C40H67O12P	[M+Formate]-
	815.4352	0.002	2.6979	LPI 30:6	C39H65O12P	[M+OAc]-
815.4374342						
815.4374342	815.4352	0.002	2.6979	LPI O-30:7;O	C39H65O12P	[M+OAc]-
	815.4352	0.002	2.6979	PI O-30:6	C39H65O12P	[M+OAc]-
815.4374342						
816.3966648	816.3976	0.001	1.2249	SHexCer 31:3;O5	C37H67NO14S	[M+Cl]-
	816.421	0.003	3.1846	SHexCer 34:6;O5	C40H67NO14S	[M-H]-
816.4235432						
816.4235432	816.4224	0.001	1.3473	PS 35:6;O	C41H68NO11P	[M+Cl]-

	816.5396	0.003	4.0414	IPC 37:3;O2	C43H80NO11P	[M-H]-
816.5429501						
816.5429501	816.5396	0.003	4.0414	PS 37:2;O	C43H80NO11P	[M-H]-
	816.5398	0.003	3.7965	HexCer 38:4;O4	C44H79NO10	[M+Cl]-
816.5429501						
816.5429501	816.5396	0.003	4.0414	CerP 42:4;O5	C42H78NO9P	[M+Formate]-
	816.5396	0.003	4.0414	PC 34:3;O	C42H78NO9P	[M+Formate]-
816.5429501						
816.5429501	816.5396	0.003	4.0414	PE 37:3;O	C42H78NO9P	[M+Formate]-
	816.5396	0.003	4.0414	PS O-36:3	C42H78NO9P	[M+Formate]-
816.5429501						
816.5429501	816.5396	0.003	4.0414	CerP 41:4;O5	C41H76NO9P	[M+OAc]-
	816.5396	0.003	4.0414	PC 33:3;O	C41H76NO9P	[M+OAc]-
816.5429501						
816.5429501	816.5396	0.003	4.0414	PE 36:3;O	C41H76NO9P	[M+OAc]-
	816.5396	0.003	4.0414	PS O-35:3	C41H76NO9P	[M+OAc]-
816.5429501						
817.4175750	817.4202	0.003	3.3031	SQDG 35:9	C44H66O12S	[M-H]-
	817.4145	0.003	3.7924	LPI 30:6;O	C39H65O13P	[M+Formate]-
817.4175750						
817.4175750	817.4145	0.003	3.7924	PI 30:5	C39H65O13P	[M+Formate]-
	817.4145	0.003	3.7924	PI O-30:6;O	C39H65O13P	[M+Formate]-
817.4175750						
817.4175750	817.4145	0.003	3.7924	LPI 29:6;O	C38H63O13P	[M+OAc]-
	817.4145	0.003	3.7924	PI 29:5	C38H63O13P	[M+OAc]-
817.4175750						
817.4175750	817.4145	0.003	3.7924	PI O-29:6;O	C38H63O13P	[M+OAc]-
	819.4066	0.001	1.5865	PIP 27:0	C36H70O16P2	[M-H]-
819.4053696						
819.4348850	819.4359	0.001	1.2204	SQDG 35:8	C44H68O12S	[M-H]-
	820.3926	0.002	2.1941	SHexCer 30:2;O6	C36H67NO15S	[M+Cl]-
820.3943614						
820.3943614	820.3962	0.002	2.1941	PS 37:10	C43H64NO10P	[M+Cl]-
	821.3788	0.002	2.0697	SQDG 32:9	C41H60O12S	[M+Formate]-
821.3770922						
821.4522179	821.4515	0.001	0.8522	SQDG 35:7	C44H70O12S	[M-H]-
	821.453	0.001	0.9739	PA 42:9;O	C45H71O9P	[M+Cl]-
821.4522179						
821.4522179	821.453	0.001	0.9739	PG O-39:10	C45H71O9P	[M+Cl]-

	822.5291	0.003	3.1610	PS 39:5	C45H78NO10P	[M-H]-
822.5264357						
822.5264357	822.5291	0.003	3.1610	PS O-39:6;O	C45H78NO10P	[M-H]-
	822.5291	0.003	3.1610	PC 36:6	C44H76NO8P	[M+Formate]-
822.5264357						
822.5264357	822.5291	0.003	3.1610	PC O-36:7;O	C44H76NO8P	[M+Formate]-
	822.5291	0.003	3.1610	PE 39:6	C44H76NO8P	[M+Formate]-
822.5264357						
822.5264357	822.5291	0.003	3.1610	PE O-39:7;O	C44H76NO8P	[M+Formate]-
	822.5291	0.003	3.1610	PC 35:6	C43H74NO8P	[M+OAc]-
822.5264357						
822.5264357	822.5291	0.003	3.1610	PC O-35:7;O	C43H74NO8P	[M+OAc]-
	822.5291	0.003	3.1610	PE 38:6	C43H74NO8P	[M+OAc]-
822.5264357						
822.5264357	822.5291	0.003	3.1610	PE O-38:7;O	C43H74NO8P	[M+OAc]-
	823.3944	0.000	0.4858	SQDG 32:8	C41H62O12S	[M+Formate]-
823.3940321						
823.3940321	823.3944	0.000	0.4858	SQDG 31:8	C40H60O12S	[M+OAc]-
	823.5366	0.000	0.3643	MGDG 40:9	C49H76O10	[M-H]-
823.5368599						
823.5368599	823.5374	0.001	0.6071	EPC 40:3;O5	C42H81N2O9P	[M+Cl]-
	823.5374	0.001	0.6071	SM 37:3;O5	C42H81N2O9P	[M+Cl]-
823.5368599						
823.5368599	823.5366	0.000	0.3643	TG 45:9;O2	C48H74O8	[M+Formate]-
	823.5366	0.000	0.3643	TG O-45:10;O3	C48H74O8	[M+Formate]-
823.5368599						
823.5368599	823.5366	0.000	0.3643	TG 44:9;O2	C47H72O8	[M+OAc]-
	823.5366	0.000	0.3643	TG O-44:10;O3	C47H72O8	[M+OAc]-
823.5368599						
825.3556556	825.3597	0.004	4.8464	PIP 28:4	C37H64O16P2	[M-H]-
	825.3832	0.003	3.6347	PI 31:8	C40H61O13P	[M+Formate]-
825.3862386						
825.3862386	825.3832	0.003	3.6347	PI 30:8	C39H59O13P	[M+OAc]-
	828.434	0.000	0.4828	SHexCer 33:3;O4	C39H71NO13S	[M+Cl]-
828.4343793						
829.4166639	829.4145	0.002	2.6525	LPI 31:7;O	C40H65O13P	[M+Formate]-
	829.4145	0.002	2.6525	PI 31:6	C40H65O13P	[M+Formate]-

829.4166639						
829.4166639	829.4145	0.002	2.6525	PI O-31:7;O	C40H65O13P	[M+Formate]-
	829.4145	0.002	2.6525	LPI 30:7;O	C39H63O13P	[M+OAc]-
829.4166639						
829.4166639	829.4145	0.002	2.6525	PI 30:6	C39H63O13P	[M+OAc]-
	829.4145	0.002	2.6525	PI O-30:7;O	C39H63O13P	[M+OAc]-
829.4166639						
829.4268824	829.4276	0.001	0.8440	PI 30:2;O	C39H71O14P	[M+Cl]-
	831.3493	0.003	3.2477	PI 31:8;O	C40H61O14P	[M+Cl]-
831.3465936						
831.3966458	831.3939	0.003	3.2476	DGDG 26:6	C41H64O15	[M+Cl]-
	831.3938	0.003	3.4881	PI 30:6;O	C39H63O14P	[M+Formate]-
831.3966458						
831.3966458	831.3938	0.003	3.4881	PI 29:6;O	C38H61O14P	[M+OAc]-
	832.3926	0.001	1.6819	SHexCer 31:3;O6	C37H67NO15S	[M+Cl]-
832.3939525						
832.4071766	832.4043	0.003	3.4839	PS 36:10;O	C42H62NO11P	[M+Formate]-
	832.4043	0.003	3.4839	PS 35:10;O	C41H60NO11P	[M+OAc]-
832.4071766						
832.4276874	832.4289	0.001	1.5617	SHexCer 32:2;O5	C38H71NO14S	[M+Cl]-
	832.4336	0.001	1.0812	Hex2Cer 28:5;O6	C40H67NO17	[M-H]-
832.4327623						
832.4327623	832.4326	0.000	0.2403	PE 40:11;O	C45H68NO9P	[M+Cl]-
	832.4326	0.000	0.2403	PS O-39:11	C45H68NO9P	[M+Cl]-
832.4327623						
834.4136677	834.4118	0.002	2.1572	PS 38:10	C44H66NO10P	[M+Cl]-
	834.4541	0.000	0.2397	IPC 32:1;O5	C38H74NO14P	[M+Cl]-
834.4542899						
836.3862175	836.3839	0.002	2.7499	IPC 31:6;O6	C37H62NO15P	[M+Formate]-
	836.3839	0.002	2.7499	IPC 30:6;O6	C36H60NO15P	[M+OAc]-
836.3862175						
836.4637198	836.4639	0.000	0.2391	PC 37:9;O	C45H72NO9P	[M+Cl]-
	836.4639	0.000	0.2391	PE 40:9;O	C45H72NO9P	[M+Cl]-
836.4637198						
836.4637198	836.4639	0.000	0.2391	PS O-39:9	C45H72NO9P	[M+Cl]-
	837.5311	0.003	3.1044	TG 50:14;O2	C53H74O8	[M-H]-
837.5336967						
837.5336967	837.5311	0.003	3.1044	TG 49:14	C52H72O6	[M+Formate]-

	839.2791	0.003	3.4553	PIP2 23:2	C32H59O19P3	[M-H]-
839.2762274						
839.6064607	839.6051	0.001	1.6675	EPC 42:1;O4	C44H89N2O8P	[M+Cl]-
	839.6051	0.001	1.6675	SM 39:1;O4	C44H89N2O8P	[M+Cl]-
839.6064607						
840.4161907	840.4152	0.001	1.1899	IPC 31:4;O6	C37H66NO15P	[M+Formate]-
	840.4152	0.001	1.1899	IPC 30:4;O6	C36H64NO15P	[M+OAc]-
840.4161907						
840.4225731	840.4224	0.000	0.2380	PS 37:8;O	C43H68NO11P	[M+Cl]-
	840.5396	0.003	3.9260	IPC 39:5;O2	C45H80NO11P	[M-H]-
840.5429509						
840.5429509	840.5396	0.003	3.9260	PS 39:4;O	C45H80NO11P	[M-H]-
	840.5398	0.003	3.6881	HexCer 40:6;O4	C46H79NO10	[M+Cl]-
840.5429509						
840.5429509	840.5396	0.003	3.9260	CerP 44:6;O5	C44H78NO9P	[M+Formate]-
	840.5396	0.003	3.9260	PC 36:5;O	C44H78NO9P	[M+Formate]-
840.5429509						
840.5429509	840.5396	0.003	3.9260	PE 39:5;O	C44H78NO9P	[M+Formate]-
	840.5396	0.003	3.9260	PS O-38:5	C44H78NO9P	[M+Formate]-
840.5429509						
840.5429509	840.5396	0.003	3.9260	CerP 43:6;O5	C43H76NO9P	[M+OAc]-
	840.5396	0.003	3.9260	PC 35:5;O	C43H76NO9P	[M+OAc]-
840.5429509						
840.5429509	840.5396	0.003	3.9260	PE 38:5;O	C43H76NO9P	[M+OAc]-
	840.5396	0.003	3.9260	PS O-37:5	C43H76NO9P	[M+OAc]-
840.5429509						
841.2552816	841.2583	0.003	3.6850	PIP2 22:2;O	C31H57O20P3	[M-H]-
	841.3313	0.002	2.4960	PIP 25:1;O	C34H64O17P2	[M+Cl]-
841.3333942						
841.4052988	841.4064	0.001	1.3073	PI O-34:9	C43H67O12P	[M+Cl]-
	841.5389	0.001	0.9506	PA 46:9;O	C49H79O9P	[M-H]-
841.5380910						
841.5380910	841.5389	0.001	0.9506	PG O-43:10	C49H79O9P	[M-H]-
	841.5391	0.001	1.1883	TG 47:9;O2	C50H78O8	[M+Cl]-
841.5380910						
841.5380910	841.5391	0.001	1.1883	TG O-47:10;O3	C50H78O8	[M+Cl]-
	841.5367	0.001	1.6636	LPI O-34:2	C43H83O11P	[M+Cl]-
841.5380910						
841.5380910	841.5367	0.001	1.6636	PG 37:1;O	C43H83O11P	[M+Cl]-

	841.5389	0.001	0.9506	PA O-45:10	C48H77O7P	[M+Formate]-
841.5380910						
841.5380910	841.5389	0.001	0.9506	PA O-44:10	C47H75O7P	[M+OAc]-
	842.3864	0.001	0.5936	IPC 32:5;O6	C38H66NO15P	[M+Cl]-
842.3859483						
843.3859519	843.3857	0.000	0.3557	PI 33:8	C42H65O13P	[M+Cl]-
	843.3857	0.000	0.3557	PI O-33:9;O	C42H65O13P	[M+Cl]-
843.3859519						
843.4334629	843.4359	0.002	2.8455	SQDG 37:10	C46H68O12S	[M-H]-
	844.4289	0.001	1.5395	SHexCer 33:3;O5	C39H71NO14S	[M+Cl]-
844.4276377						
845.4029485	845.4013	0.002	1.8926	PI 33:7	C42H67O13P	[M+Cl]-
	845.4013	0.002	1.8926	PI O-33:8;O	C42H67O13P	[M+Cl]-
845.4029485						
845.4524951	845.4515	0.001	1.1828	SQDG 37:9	C46H70O12S	[M-H]-
	845.453	0.001	0.5914	PA 44:11;O	C47H71O9P	[M+Cl]-
845.4524951						
846.4455313	846.4446	0.001	1.0633	SHexCer 33:2;O5	C39H73NO14S	[M+Cl]-
	847.4672	0.001	1.0620	SQDG 37:8	C46H72O12S	[M-H]-
847.4662774						
847.5039385	847.505	0.001	1.2979	PA 45:9	C48H77O8P	[M+Cl]-
	847.505	0.001	1.2979	PA O-45:10;O	C48H77O8P	[M+Cl]-
847.5039385						
848.4245759	848.4239	0.001	0.8251	SHexCer 32:2;O6	C38H71NO15S	[M+Cl]-
	849.3808	0.004	4.9448	PIP 26:1	C35H66O16P2	[M+Formate]-
849.3850679						
849.3850679	849.3808	0.004	4.9448	PIP 25:1	C34H64O16P2	[M+OAc]-
	850.4068	0.002	2.7046	PS 38:10;O	C44H66NO11P	[M+Cl]-
850.4044763						
850.5222486	850.524	0.002	1.9988	PS 40:6;O	C46H78NO11P	[M-H]-
	850.524	0.002	1.9988	PC 37:7;O	C45H76NO9P	[M+Formate]-
850.5222486						
850.5222486	850.524	0.002	1.9988	PE 40:7;O	C45H76NO9P	[M+Formate]-
	850.524	0.002	1.9988	PS O-39:7	C45H76NO9P	[M+Formate]-
850.5222486						
850.5222486	850.524	0.002	1.9988	PC 36:7;O	C44H74NO9P	[M+OAc]-
	850.524	0.002	1.9988	PE 39:7;O	C44H74NO9P	[M+OAc]-

850.5222486						
850.5222486	850.524	0.002	1.9988	PS O-38:7	C44H74NO9P	[M+OAc]-
	851.2063	0.000	0.1175	PIP2 21:5	C30H49O19P3	[M+Formate]-
851.2064188						
851.2064188	851.2063	0.000	0.1175	PIP2 20:5	C29H47O19P3	[M+OAc]-
	852.4552	0.001	1.6423	SHexCer 32:0;O6	C38H75NO15S	[M+Cl]-
852.4565577						
852.4565577	852.4588	0.002	2.5808	PS 39:8	C45H72NO10P	[M+Cl]-
	852.4588	0.002	2.5808	PS O-39:9;O	C45H72NO10P	[M+Cl]-
852.4565577						
853.4046619	853.4064	0.002	2.1092	PI O-35:10	C44H67O12P	[M+Cl]-
	854.4744	0.001	0.7022	PS 39:7	C45H74NO10P	[M+Cl]-
854.4738029						
854.4738029	854.4744	0.001	0.7022	PS O-39:8;O	C45H74NO10P	[M+Cl]-
	854.5342	0.001	0.9362	PE 44:10;O	C49H78NO9P	[M-H]-
854.5349484						
854.5349484	854.5342	0.001	0.9362	PS O-43:10	C49H78NO9P	[M-H]-
	854.5342	0.001	0.9362	PC O-40:11	C48H76NO7P	[M+Formate]-
854.5349484						
854.5349484	854.5342	0.001	0.9362	PE O-43:11	C48H76NO7P	[M+Formate]-
	854.5342	0.001	0.9362	PC O-39:11	C47H74NO7P	[M+OAc]-
854.5349484						
854.5349484	854.5342	0.001	0.9362	PE O-42:11	C47H74NO7P	[M+OAc]-
	854.5342	0.001	0.9362	PC 42:10;O	C50H80NO9P	[M-CH3]-
854.5349484						
855.6015135	855.5992	0.002	2.6882	MGDG 42:7	C51H84O10	[M-H]-
	855.6	0.002	1.7532	EPC 42:1;O5	C44H89N2O9P	[M+Cl]-
855.6015135						
855.6015135	855.6	0.002	1.7532	SM 39:1;O5	C44H89N2O9P	[M+Cl]-
	855.5992	0.002	2.6882	TG 47:7;O2	C50H82O8	[M+Formate]-
855.6015135						
855.6015135	855.5992	0.002	2.6882	TG O-47:8;O3	C50H82O8	[M+Formate]-
	855.5992	0.002	2.6882	TG 46:7;O2	C49H80O8	[M+OAc]-
855.6015135						
855.6015135	855.5992	0.002	2.6882	TG O-46:8;O3	C49H80O8	[M+OAc]-
	856.4653	0.001	1.1676	SHexCer 35:3;O4	C41H75NO13S	[M+Cl]-
856.4663403						
856.6050921	856.6073	0.002	2.5683	CerP 47:4;O6	C47H88NO10P	[M-H]-

856.6050921	856.6073	0.002	2.5683	PS 41:2	C47H88NO10P	[M-H]-
856.6050921	856.6073	0.002	2.5683	PS O-41:3;O	C47H88NO10P	[M-H]-
856.6050921	856.6075	0.002	2.8017	HexCer 42:4;O3	C48H87NO9	[M+Cl]-
856.6050921	856.6073	0.002	2.5683	CerP 46:4;O4	C46H86NO8P	[M+Formate]-
856.6050921	856.6073	0.002	2.5683	PC 38:3	C46H86NO8P	[M+Formate]-
856.6050921	856.6073	0.002	2.5683	PC O-38:4;O	C46H86NO8P	[M+Formate]-
856.6050921	856.6073	0.002	2.5683	PE 41:3	C46H86NO8P	[M+Formate]-
856.6050921	856.6073	0.002	2.5683	PE O-41:4;O	C46H86NO8P	[M+Formate]-
856.6050921	856.6073	0.002	2.5683	CerP 45:4;O4	C45H84NO8P	[M+OAc]-
856.6050921	856.6073	0.002	2.5683	PC 37:3	C45H84NO8P	[M+OAc]-
856.6050921	856.6073	0.002	2.5683	PC O-37:4;O	C45H84NO8P	[M+OAc]-
856.6050921	856.6073	0.002	2.5683	PE 40:3	C45H84NO8P	[M+OAc]-
856.6050921	856.6073	0.002	2.5683	PE O-40:4;O	C45H84NO8P	[M+OAc]-
857.4246085	857.4223	0.002	2.6825	PIP 30:2	C39H72O16P2	[M-H]-
857.5333813	857.5338	0.000	0.4665	PG 43:9	C49H79O10P	[M-H]-
857.5333813	857.5338	0.000	0.4665	PG O-43:10;O	C49H79O10P	[M-H]-
857.5333813	857.534	0.001	0.6997	TG 47:9;O3	C50H78O9	[M+Cl]-
857.5333813	857.5338	0.000	0.4665	PA 45:9	C48H77O8P	[M+Formate]-
857.5333813	857.5338	0.000	0.4665	PA O-45:10;O	C48H77O8P	[M+Formate]-
857.5333813	857.5338	0.000	0.4665	PA 44:9	C47H75O8P	[M+OAc]-
857.5333813	857.5338	0.000	0.4665	PA O-44:10;O	C47H75O8P	[M+OAc]-
857.5540790	857.5549	0.001	1.0495	PI O-37:4	C46H83O12P	[M-H]-
857.5540790	857.5549	0.001	1.0495	PG 39:4	C45H81O10P	[M+Formate]-
857.5540790	857.5549	0.001	1.0495	PG O-39:5;O	C45H81O10P	[M+Formate]-
857.5540790	857.5549	0.001	1.0495	PG 38:4	C44H79O10P	[M+OAc]-
857.5540790	857.5549	0.001	1.0495	PG O-38:5;O	C44H79O10P	[M+OAc]-

	857.6148	0.002	2.4487	MGDG 42:6	C51H86O10	[M-H]-
857.6169101						
857.6169101	857.6156	0.001	1.5158	EPC 42:0;O5	C44H91N2O9P	[M+Cl]-
	857.6156	0.001	1.5158	SM 39:0;O5	C44H91N2O9P	[M+Cl]-
857.6169101						
857.6169101	857.6148	0.002	2.4487	TG 47:6;O2	C50H84O8	[M+Formate]-
	857.6148	0.002	2.4487	TG O-47:7;O3	C50H84O8	[M+Formate]-
857.6169101						
857.6169101	857.6148	0.002	2.4487	TG 46:6;O2	C49H82O8	[M+OAc]-
	857.6148	0.002	2.4487	TG O-46:7;O3	C49H82O8	[M+OAc]-
857.6169101						
858.4448416	858.4446	0.000	0.3495	SHexCer 34:3;O5	C40H73NO14S	[M+Cl]-
	859.4252	0.002	2.0944	DGDG 28:6	C43H68O15	[M+Cl]-
859.4270188						
859.4270188	859.4251	0.002	2.3271	PI 32:6;O	C41H67O14P	[M+Formate]-
	859.4251	0.002	2.3271	PI 31:6;O	C40H65O14P	[M+OAc]-
859.4270188						
860.4247406	860.4239	0.001	1.0460	SHexCer 33:3;O6	C39H71NO15S	[M+Cl]-
	861.4101	0.003	3.8309	SQDG 35:10	C44H64O12S	[M+Formate]-
861.4067933						
862.4049304	862.4068	0.002	2.0872	PS 39:11;O	C45H66NO11P	[M+Cl]-
	862.4759	0.002	1.7392	SHexCer 34:1;O5	C40H77NO14S	[M+Cl]-
862.4774133						
862.4774133	862.4795	0.002	2.4348	PC 39:10;O	C47H74NO9P	[M+Cl]-
	862.4795	0.002	2.4348	PE 42:10;O	C47H74NO9P	[M+Cl]-
862.4774133						
862.4774133	862.4795	0.002	2.4348	PS O-41:10	C47H74NO9P	[M+Cl]-
	862.5392	0.001	1.5072	PE 46:12	C51H78NO8P	[M-H]-
862.5379869						
862.5379869	862.537	0.001	1.0434	CerP 45:5;O6	C45H82NO10P	[M+Cl]-
	862.537	0.001	1.0434	PS 39:3	C45H82NO10P	[M+Cl]-
862.5379869						
862.5379869	862.537	0.001	1.0434	PS O-39:4;O	C45H82NO10P	[M+Cl]-
	862.5392	0.001	1.5072	PC 44:12	C52H80NO8P	[M-CH3]-
862.5379869						
863.4133977	863.4119	0.002	1.7373	PI 33:6;O	C42H69O14P	[M+Cl]-
	863.4483	0.003	3.8219	LPI 34:6;O	C43H73O13P	[M+Cl]-

863.4450181						
863.4450181	863.4483	0.003	3.8219	PI 34:5	C43H73O13P	[M+Cl]-
	863.4483	0.003	3.8219	PI O-34:6;O	C43H73O13P	[M+Cl]-
863.4450181						
863.5577271	863.5574	0.000	0.3474	PG 40:3	C46H85O10P	[M+Cl]-
	863.5574	0.000	0.3474	PG O-40:4;O	C46H85O10P	[M+Cl]-
863.5577271						
864.4173736	864.4152	0.002	2.5451	MIPC 28:5;O3	C40H68NO17P	[M-H]-
	864.4154	0.002	2.3137	Hex2Cer 29:6;O5	C41H67NO16	[M+Cl]-
864.4173736						
864.4173736	864.4152	0.002	2.5451	IPC 33:6;O6	C39H66NO15P	[M+Formate]-
	864.4152	0.002	2.5451	IPC 32:6;O6	C38H64NO15P	[M+OAc]-
864.4173736						
866.4359877	866.4381	0.002	2.4237	PS 39:9;O	C45H70NO11P	[M+Cl]-
	868.4537	0.001	0.6909	PS 39:8;O	C45H72NO11P	[M+Cl]-
868.4531410						
869.2671932	869.2687	0.002	1.7256	PIP 28:8;O	C37H56O17P2	[M+Cl]-
	869.3626	0.001	1.0352	PIP 27:1;O	C36H68O17P2	[M+Cl]-
869.3634794						
869.5333274	869.5338	0.001	0.5750	PG 44:10	C50H79O10P	[M-H]-
	869.5338	0.001	0.5750	PG O-44:11;O	C50H79O10P	[M-H]-
869.5333274						
869.5333274	869.534	0.001	0.8050	TG 48:10;O3	C51H78O9	[M+Cl]-
	869.5338	0.001	0.5750	PA 46:10	C49H77O8P	[M+Formate]-
869.5333274						
869.5333274	869.5338	0.001	0.5750	PA O-46:11;O	C49H77O8P	[M+Formate]-
	869.5338	0.001	0.5750	PA 45:10	C48H75O8P	[M+OAc]-
869.5333274						
869.5333274	869.5338	0.001	0.5750	PA O-45:11;O	C48H75O8P	[M+OAc]-
	869.5454	0.001	0.6900	SQDG 38:4	C47H82O12S	[M-H]-
869.5448070						
869.5989445	869.6026	0.004	4.1398	EPC 45:5;O6	C47H87N2O10P	[M-H]-
	869.6026	0.004	4.1398	EPC 44:5;O4	C46H85N2O8P	[M+Formate]-
869.5989445						
869.5989445	869.6026	0.004	4.1398	SM 41:5;O4	C46H85N2O8P	[M+Formate]-
	869.6026	0.004	4.1398	EPC 43:5;O4	C45H83N2O8P	[M+OAc]-
869.5989445						
869.5989445	869.6026	0.004	4.1398	SM 40:5;O4	C45H83N2O8P	[M+OAc]-

	869.6026	0.004	4.1398	SM 43:5;O6	C48H89N2O10P	[M-CH3]-
869.5989445						
869.6010844	869.6026	0.002	1.7249	EPC 45:5;O6	C47H87N2O10P	[M-H]-
	869.6026	0.002	1.7249	EPC 44:5;O4	C46H85N2O8P	[M+Formate]-
869.6010844						
869.6010844	869.6026	0.002	1.7249	SM 41:5;O4	C46H85N2O8P	[M+Formate]-
	869.6026	0.002	1.7249	EPC 43:5;O4	C45H83N2O8P	[M+OAc]-
869.6010844						
869.6010844	869.6026	0.002	1.7249	SM 40:5;O4	C45H83N2O8P	[M+OAc]-
	869.6026	0.002	1.7249	SM 43:5;O6	C48H89N2O10P	[M-CH3]-
869.6010844						
870.4676556	870.4694	0.002	1.9530	PS 39:7;O	C45H74NO11P	[M+Cl]-
	870.5502	0.003	3.9056	IPC 40:5;O3	C46H82NO12P	[M-H]-
870.5467501						
870.5467501	870.5432	0.004	4.1353	Hex2Cer 30:0;O6	C42H81NO17	[M-H]-
	870.5504	0.004	4.1353	HexCer 41:6;O5	C47H81NO11	[M+Cl]-
870.5467501						
870.5467501	870.5502	0.003	3.9056	CerP 45:6;O6	C45H80NO10P	[M+Formate]-
	870.5502	0.003	3.9056	PS 39:4	C45H80NO10P	[M+Formate]-
870.5467501						
870.5467501	870.5502	0.003	3.9056	PS O-39:5;O	C45H80NO10P	[M+Formate]-
	870.5432	0.004	4.1353	Hex2Cer 29:0;O4	C41H79NO15	[M+Formate]-
870.5467501						
870.5467501	870.5502	0.003	3.9056	CerP 44:6;O6	C44H78NO10P	[M+OAc]-
	870.5502	0.003	3.9056	PS 38:4	C44H78NO10P	[M+OAc]-
870.5467501						
870.5467501	870.5502	0.003	3.9056	PS O-38:5;O	C44H78NO10P	[M+OAc]-
	870.5432	0.004	4.1353	Hex2Cer 28:0;O4	C40H77NO15	[M+OAc]-
870.5467501						
873.4455243	873.4479	0.002	2.7477	PG 42:12	C48H71O10P	[M+Cl]-
	875.3753	0.001	1.4851	PIP 32:7	C41H66O16P2	[M-H]-
875.3766422						
875.4970889	875.4985	0.001	1.5991	SQDG 39:8	C48H76O12S	[M-H]-
	877.391	0.002	2.2795	PIP 32:6	C41H68O16P2	[M-H]-
877.3929734						
877.4770495	877.4792	0.002	2.3932	PG 42:10	C48H75O10P	[M+Cl]-
	877.4792	0.002	2.3932	PG O-42:11;O	C48H75O10P	[M+Cl]-
877.4770495						
878.4242280	878.4214	0.003	3.3014	SHexCer 34:6;O6	C40H67NO15S	[M+Formate]-

	878.4214	0.003	3.3014	SHexCer 33:6;O6	C39H65NO15S	[M+OAc]-
878.4242280						
879.4161253	879.4126	0.004	4.0936	SQDG 37:10	C46H68O12S	[M+Cl]-
	881.3859	0.002	1.9288	PIP 31:5;O	C40H68O17P2	[M-H]-
881.3875638						
881.4247326	881.4223	0.002	2.7229	PIP 32:4	C41H72O16P2	[M-H]-
	881.454	0.001	0.6807	DGDG 29:7	C44H68O15	[M+Formate]-
881.4534516						
881.4534516	881.454	0.001	0.6807	DGDG 28:7	C43H66O15	[M+OAc]-
	885.456	0.001	1.4682	PI 39:11	C48H71O13P	[M-H]-
885.4572223						
886.4155517	886.4126	0.003	3.2716	MIPC 28:4;O2	C40H70NO16P	[M+Cl]-
	887.4329	0.002	2.3664	PIP 31:2;O	C40H74O17P2	[M-H]-
887.4349882						
887.4737286	887.4716	0.002	2.3663	PI 39:10	C48H73O13P	[M-H]-
	887.4716	0.002	2.3663	PI O-39:11;O	C48H73O13P	[M-H]-
887.4737286						
887.4737286	887.4752	0.001	1.5775	SQDG 37:6	C46H76O12S	[M+Cl]-
	887.4716	0.002	2.3663	PG 41:11;O	C47H71O11P	[M+Formate]-
887.4737286						
887.4737286	887.4716	0.002	2.3663	PG 40:11;O	C46H69O11P	[M+OAc]-
	887.5444	0.000	0.2253	PG 44:9;O	C50H81O11P	[M-H]-
887.5446102						
887.5446102	887.5446	0.000	0.1127	MGDG 42:9	C51H80O10	[M+Cl]-
	887.5444	0.000	0.2253	PA 46:9;O	C49H79O9P	[M+Formate]-
887.5446102						
887.5446102	887.5444	0.000	0.2253	PG O-43:10	C49H79O9P	[M+Formate]-
	887.5444	0.000	0.2253	PA 45:9;O	C48H77O9P	[M+OAc]-
887.5446102						
887.5446102	887.5444	0.000	0.2253	PG O-42:10	C48H77O9P	[M+OAc]-
	888.4552	0.001	1.3507	SHexCer 35:3;O6	C41H75NO15S	[M+Cl]-
888.4563553						
888.5284343	888.5279	0.001	0.5627	SHexCer 37:1;O4	C43H83NO13S	[M+Cl]-
	889.4792	0.002	2.2485	PG 43:11	C49H75O10P	[M+Cl]-
889.4771893						
889.4771893	889.4792	0.002	2.2485	PG O-43:12;O	C49H75O10P	[M+Cl]-
	889.5141	0.000	0.4497	SQDG 40:8	C49H78O12S	[M-H]-

889.5137185						
890.4350651	890.4381	0.003	3.3691	PS 41:11;O	C47H70NO11P	[M+Cl]-
	890.5472	0.002	1.9089	PC 42:9	C50H82NO8P	[M+Cl]-
890.5455433						
890.5455433	890.5472	0.002	1.9089	PC O-42:10;O	C50H82NO8P	[M+Cl]-
	890.5472	0.002	1.9089	PE 45:9	C50H82NO8P	[M+Cl]-
890.5455433						
890.5455433	890.5472	0.002	1.9089	PE O-45:10;O	C50H82NO8P	[M+Cl]-
	890.5436	0.002	2.2458	SHexCer 37:0;O4	C43H85NO13S	[M+Cl]-
890.5455433						
891.4050502	891.4066	0.002	1.7949	PIP 33:6	C42H70O16P2	[M-H]-
	891.5298	0.002	2.3555	SQDG 40:7	C49H80O12S	[M-H]-
891.5276974						
891.5276974	891.5264	0.001	1.4582	MGDG 42:12	C51H74O10	[M+Formate]-
	893.3626	0.001	0.7836	PIP 29:3;O	C38H68O17P2	[M+Cl]-
893.3633321						
893.4060751	893.407	0.001	1.1193	PIP 28:1;O	C37H70O17P2	[M+Formate]-
	893.407	0.001	1.1193	PIP 27:1;O	C36H68O17P2	[M+OAc]-
893.4060751						
895.3754543	895.3782	0.003	3.1272	PIP 29:2;O	C38H70O17P2	[M+Cl]-
	896.4239	0.001	1.1155	SHexCer 36:6;O6	C42H71NO15S	[M+Cl]-
896.4248395						
896.4446398	896.4414	0.003	3.5697	MIPC 29:4;O4	C41H72NO18P	[M-H]-
	896.4416	0.003	3.3466	Hex2Cer 30:5;O6	C42H71NO17	[M+Cl]-
896.4446398						
896.4446398	896.4414	0.003	3.5697	MIPC 28:4;O2	C40H70NO16P	[M+Formate]-
	897.4409	0.004	4.1228	DGDG 31:8	C46H70O15	[M+Cl]-
897.4446129						
897.4446129	897.4407	0.004	4.3457	PI 35:8;O	C44H69O14P	[M+Formate]-
	897.4407	0.004	4.3457	PI 34:8;O	C43H67O14P	[M+OAc]-
897.4446129						
899.4363960	899.4329	0.004	3.8913	PIP 32:3;O	C41H74O17P2	[M-H]-
	900.5374	0.001	0.9994	IPC 38:2;O4	C44H84NO13P	[M+Cl]-
900.5365573						
900.5365573	900.536	0.001	0.6663	SHexCer 36:1;O5	C42H81NO14S	[M+Formate]-
	900.536	0.001	0.6663	SHexCer 35:1;O5	C41H79NO14S	[M+OAc]-
900.5365573						
901.4536910	901.4509	0.003	3.1061	PI 39:11;O	C48H71O14P	[M-H]-
	901.4722	0.003	3.6607	DGDG 31:6	C46H74O15	[M+Cl]-

901.4755188						
901.4755188	901.4792	0.004	4.1044	PG 44:12	C50H75O10P	[M+Cl]-
	901.472	0.004	3.8825	PI 35:6;O	C44H73O14P	[M+Formate]-
901.4755188						
901.4755188	901.472	0.004	3.8825	PI 34:6;O	C43H71O14P	[M+OAc]-
	901.5471	0.001	1.5529	TG 50:13;O3	C53H76O9	[M+Formate]-
901.5485198						
901.5485198	901.5471	0.001	1.5529	TG 49:13;O3	C52H74O9	[M+OAc]-
	903.4948	0.002	2.4350	PG 44:11	C50H77O10P	[M+Cl]-
903.4926880						
903.4926880	903.4948	0.002	2.4350	PG O-44:12;O	C50H77O10P	[M+Cl]-
	904.4537	0.001	0.6634	PS 42:11;O	C48H72NO11P	[M+Cl]-
904.4530736						
905.3956232	905.399	0.003	3.7553	PIP 31:3	C40H72O16P2	[M+Cl]-
	905.4223	0.002	1.8776	PIP 34:6	C43H72O16P2	[M-H]-
905.4240072						
905.5075596	905.5105	0.003	3.2026	PG 44:10	C50H79O10P	[M+Cl]-
	905.5105	0.003	3.2026	PG O-44:11;O	C50H79O10P	[M+Cl]-
905.5075596						
907.4537904	907.4534	0.000	0.4408	PI O-39:11	C48H73O12P	[M+Cl]-
	909.4536	0.002	1.7593	PIP 34:4	C43H76O16P2	[M-H]-
909.4551804						
911.4735802	911.4752	0.002	1.7554	SQDG 39:8	C48H76O12S	[M+Cl]-
	912.3919	0.003	3.2881	MIPC 29:6;O3	C41H68NO17P	[M+Cl]-
912.3949106						
912.4339515	912.4364	0.002	2.6303	MIPC 29:4;O5	C41H72NO19P	[M-H]-
	912.4364	0.002	2.6303	MIPC 28:4;O3	C40H70NO17P	[M+Formate]-
912.4339515						
913.4859025	913.4849	0.001	1.0947	PIP 34:2	C43H80O16P2	[M-H]-
	913.4873	0.001	1.5326	PI 41:11	C50H75O13P	[M-H]-
913.4859025						
913.4859025	913.4873	0.001	1.5326	PG 43:12;O	C49H73O11P	[M+Formate]-
	913.4873	0.001	1.5326	PG 42:12;O	C48H71O11P	[M+OAc]-
913.4859025						
914.4476867	914.452	0.004	4.7023	MIPC 29:3;O5	C41H74NO19P	[M-H]-
	914.4439	0.004	4.1555	MIPC 30:4;O2	C42H74NO16P	[M+Cl]-
914.4476867						
914.4476867	914.452	0.004	4.7023	MIPC 28:3;O3	C40H72NO17P	[M+Formate]-

Supplementary Table S7: Annotations for downregulated ion signals in positive-ion mode

Input Mass	Matched Mass	Delta	ppm	Name	Formula	Adduct
312.3258246	312.3261		0.9605	SPB 20:1;O	C20H41NO	[M+H]+
312.3258246	312.3261	0.0003	0.9605	SPB 20:0;O2	C20H43NO2	[M+H-H2O]+
313.2344929	313.2349		1.2770	MG O-13:0;O	C16H34O4Na	[M+Na]+
324.1919527	324.1934	0.0015	4.6269	SPBP 14:1;O2	C14H30NO5P	[M+H]+
324.1919527	324.1934		4.6269	SPBP 14:0;O3	C14H32NO6P	[M+H-H2O]+
324.1919527	324.1935	0.0016	4.9353	NAE 14:1;O	C16H31NO3K	[M+K]+
324.1919527	324.1935	0.0016	4.9353	SPB 16:2;O3	C16H31NO3K	[M+K]+
325.1979807	325.1985		1.8450	FA 16:1;O3	C16H30O5Na	[M+Na]+
325.1979807	325.1985	0.0006	1.8450	MG 13:1;O	C16H30O5Na	[M+Na]+
327.2137600	327.2142	0.0004	1.2224	FA 16:0;O3	C16H32O5Na	[M+Na]+
327.2137600	327.2142	0.0004	1.2224	MG 13:0;O	C16H32O5Na	[M+Na]+
339.2528015	339.253	0.0002	0.5895	FA 20:3;O2	C20H34O4	[M+H]+
339.2528015	339.253	0.0002	0.5895	MG 17:3	C20H34O4	[M+H]+
339.2528015	339.253	0.0002	0.5895	MG O-17:4;O	C20H34O4	[M+H]+
339.2528015	339.253	0.0002	0.5895	ST 20:0;O4	C20H34O4	[M+H]+
339.2528015	339.253	0.0002	0.5895	FA 20:2;O3	C20H36O5	[M+H-H2O]+
339.2528015	339.253	0.0002	0.5895	MG 17:2;O	C20H36O5	[M+H-H2O]+
342.1881899	342.1887	0.0005	1.4612	NAE 13:1;O4	C15H29NO6Na	[M+Na]+
342.2991495	342.3003	0.0011	3.2136	NAE 18:1;O	C20H39NO3	[M+H]+
342.2991495	342.3003	0.0011	3.2136	SPB 20:2;O3	C20H39NO3	[M+H]+
342.2991495	342.3003	0.0011	3.2136	NAE 18:0;O2	C20H41NO4	[M+H-H2O]+
342.2991495	342.3003	0.0011	3.2136	SPB 20:1;O4	C20H41NO4	[M+H-H2O]+
342.2991495	342.3003	0.0011	3.2136	FA 20:2;O	C20H36O3	[M+NH4]+

342.2991495	342.3003	0.0011	3.2136	MG O-17:3	C20H36O3	[M+NH4]+
344.1464660	344.147	0.0005	1.4529	NAE 12:1;O4	C14H27NO6K	[M+K]+
344.2040063	344.2044	0.0003	0.8716	NAE 13:0;O4	C15H31NO6Na	[M+Na]+
344.2421278	344.2431	0.0010	2.9049	CAR 11:1;O	C18H33NO5	[M+H]+
344.2421278	344.2431	0.0010	2.9049	NAE 16:2;O3	C18H33NO5	[M+H]+
344.2421278	344.2431	0.0010	2.9049	CAR 11:0;O2	C18H35NO6	[M+H-H2O]+
344.2421278	344.2431	0.0010	2.9049	NAE 16:1;O4	C18H35NO6	[M+H-H2O]+
344.2421278	344.2431	0.0010	2.9049	FA 18:3;O3	C18H30O5	[M+NH4]+
344.2421278	344.2431	0.0010	2.9049	MG 15:3;O	C18H30O5	[M+NH4]+
344.2421278	344.2431	0.0010	2.9049	ST 18:0;O5	C18H30O5	[M+NH4]+
346.0929795	346.0931	0.0001	0.2889	NAT 10:2;O3	C12H21NO7SNa	[M+Na]+
346.1737427	346.1754	0.0016	4.6219	SPBP 14:1;O2	C14H30NO5PNa	[M+Na]+
351.2494329	351.249	0.0005	1.4235	NAE 14:1;O4	C16H31NO6	[M+NH4]+
351.2504608	351.2506	0.0001	0.2847	FA 19:1;O2	C19H36O4Na	[M+Na]+
351.2504608	351.2506	0.0001	0.2847	MG 16:1	C19H36O4Na	[M+Na]+
351.2504608	351.2506	0.0001	0.2847	MG O-16:2;O	C19H36O4Na	[M+Na]+
353.2660582	353.2662	0.0002	0.5661	FA 19:0;O2	C19H38O4Na	[M+Na]+
353.2660582	353.2662	0.0002	0.5661	MG 16:0	C19H38O4Na	[M+Na]+
353.2660582	353.2662	0.0002	0.5661	MG O-16:1;O	C19H38O4Na	[M+Na]+
355.2084988	355.2091	0.0006	1.6891	FA 17:1;O4	C17H32O6Na	[M+Na]+
356.2039426	356.2044	0.0004	1.1230	NAE 14:1;O4	C16H31NO6Na	[M+Na]+
356.2411170	356.2407	0.0004	1.1228	NAE 15:0;O3	C17H35NO5Na	[M+Na]+
357.2243288	357.2248		1.1197	FA 17:0;O4	C17H34O6Na	[M+Na]+
358.2192995	358.22	0.0007	1.9541	NAE 14:0;O4	C16H33NO6Na	[M+Na]+
364.1031108	364.1037	0.0005	1.3732	NAT 10:1;O4	C12H23NO8SNa	[M+Na]+
367.2081596	367.2091	0.0009	2.4509	FA 18:2;O4	C18H32O6Na	[M+Na]+

	367.2843	0.0001	0.2723	FA 22:3;O2	C22H38O4	[M+H]+
367.2841813						
367.2841813	367.28430.0001		0.2723	MG 19:3	C22H38O4	[M+H]+
	367.2843	0.0001	0.2723	MG O-19:4;O	C22H38O4	[M+H]+
367.2841813						
367.2841813	367.28430.0001		0.2723	ST 22:0;O4	C22H38O4	[M+H]+
	367.2843	0.0001	0.2723	FA 22:2;O3	C22H40O5	[M+H-H2O]+
367.2841813						
367.2841813	367.28430.0001		0.2723	MG 19:2;O	C22H40O5	[M+H-H2O]+
	369.2248	0.0003	0.8125	FA 18:1;O4	C18H34O6Na	[M+Na]+
369.2244099						
369.2608285	369.26110.0003		0.8124	FA 19:0;O3	C19H38O5Na	[M+Na]+
	369.2611	0.0003	0.8124	MG 16:0;O	C19H38O5Na	[M+Na]+
369.2608285						
370.2194340	370.220.0006		1.6207	CAR 10:0;O2	C17H33NO6Na	[M+Na]+
	370.22	0.0006	1.6207	NAE 15:1;O4	C17H33NO6Na	[M+Na]+
370.2194340						
370.2205635	370.220.0006		1.6207	CAR 10:0;O2	C17H33NO6Na	[M+Na]+
	370.22	0.0006	1.6207	NAE 15:1;O4	C17H33NO6Na	[M+Na]+
370.2205635						
370.3316916	370.33160.0001		0.2700	NAE 20:1;O	C22H43NO3	[M+H]+
	370.3316	0.0001	0.2700	NAE 20:0;O2	C22H45NO4	[M+H-H2O]+
370.3316916						
370.3316916	370.33160.0001		0.2700	FA 22:2;O	C22H40O3	[M+NH4]+
	370.3316	0.0001	0.2700	MG O-19:3	C22H40O3	[M+NH4]+
370.3316916						
371.2395957	371.24040.0008		2.1549	FA 18:0;O4	C18H36O6Na	[M+Na]+
	371.2404	0.0001	0.2694	FA 18:0;O4	C18H36O6Na	[M+Na]+
371.2405419						
372.2353043	372.23570.0003		0.8059	NAE 15:0;O4	C17H35NO6Na	[M+Na]+
	375.2142	0.0008	2.1321	FA 20:4;O3	C20H32O5Na	[M+Na]+
375.2134333						
375.2134333	375.21420.0008		2.1321	MG 17:4;O	C20H32O5Na	[M+Na]+
	375.2142	0.0008	2.1321	ST 20:1;O5	C20H32O5Na	[M+Na]+
375.2134333						
375.2237899	375.22550.0017		4.5306	SPBP 14:0;O4	C14H32NO7P	[M+NH4]+
	382.2564	0.0004	1.0464	CAR 12:0;O	C19H37NO5Na	[M+Na]+
382.2559581						
382.2559581	382.25640.0004		1.0464	NAE 17:1;O3	C19H37NO5Na	[M+Na]+
	383.2404	0.0010	2.6093	FA 19:1;O4	C19H36O6Na	[M+Na]+

383.2394126						
383.2409789	383.2404	0.0006	1.5656	FA 19:1;O4	C19H36O6Na	[M+Na]+
	384.2357	0.0004	1.0410	CAR 11:0;O2	C18H35NO6Na	[M+Na]+
384.2352420						
384.2352420	384.2357	0.0004	1.0410	NAE 16:1;O4	C18H35NO6Na	[M+Na]+
	384.272	0.0002	0.5205	NAE 17:0;O3	C19H39NO5Na	[M+Na]+
384.2718066						
385.2556900	385.2561	0.0004	1.0383	FA 19:0;O4	C19H38O6Na	[M+Na]+
	386.2149	0.0001	0.2589	CAR 10:0;O3	C17H33NO7Na	[M+Na]+
386.2150057						
388.1028036	388.1037	0.0009	2.3190	NAT 12:3;O4	C14H23NO8SNa	[M+Na]+
	393.2248		1.5258	FA 20:3;O4	C20H34O6Na	[M+Na]+
393.2241452						
393.2241452	393.2248	0.0006	1.5258	ST 20:0;O6	C20H34O6Na	[M+Na]+
	396.2357	0.0006	1.5143	CAR 12:1;O2	C19H35NO6Na	[M+Na]+
396.2350646						
396.2350646	396.2357	0.0006	1.5143	NAE 17:2;O4	C19H35NO6Na	[M+Na]+
	397.2462	0.0017	4.2795	LPE O-12:2	C17H34NO6P	[M+NH4]+
397.2444859						
397.2444859	397.2462	0.0017	4.2795	SPBP 17:2;O3	C17H34NO6P	[M+NH4]+
	397.2561	0.0005	1.2586	FA 20:1;O4	C20H38O6Na	[M+Na]+
397.2555976						
398.2508717	398.2513	0.0004	1.0044	CAR 12:0;O2	C19H37NO6Na	[M+Na]+
	398.2513	0.0004	1.0044	NAE 17:1;O4	C19H37NO6Na	[M+Na]+
398.2508717						
400.2286722	400.2306	0.0019	4.7473	CAR 11:0;O3	C18H35NO7Na	[M+Na]+
	400.2306	0.0002	0.4997	CAR 11:0;O3	C18H35NO7Na	[M+Na]+
400.2307765						
400.3418830	400.3421	0.0002	0.4996	CAR 16:0	C23H45NO4	[M+H]+
	400.3421	0.0002	0.4996	NAE 21:1;O2	C23H45NO4	[M+H]+
400.3418830						
400.3418830	400.3421	0.0002	0.4996	NAE 21:0;O3	C23H47NO5	[M+H-H2O]+
	400.3421	0.0002	0.4996	DG O-20:2	C23H42O4	[M+NH4]+
400.3418830						
400.3418830	400.3421	0.0002	0.4996	FA 23:2;O2	C23H42O4	[M+NH4]+
	400.3421	0.0002	0.4996	MG 20:2	C23H42O4	[M+NH4]+
400.3418830						
400.3418830	400.3421	0.0002	0.4996	MG O-20:3;O	C23H42O4	[M+NH4]+
	407.1135	0.0007	1.7194	ST 18:3;O4;S	C18H24O7SNa	[M+Na]+

407.1127429						
407.2387980	407.23880.0000		0.0000	CAR 11:2;O4	C18H31NO8	[M+NH4]+
	407.2428	0.0013	3.1922	DG 20:5;O	C23H34O6	[M+H]+
407.2415284						
407.2415284	407.24280.0013		3.1922	FA 23:6;O4	C23H34O6	[M+H]+
	407.2428	0.0013	3.1922	ST 23:3;O6	C23H34O6	[M+H]+
407.2415284						
407.2415284	407.24280.0013		3.1922	DG 20:4;O2	C23H36O7	[M+H-H2O]+
	407.2428	0.0013	3.1922	ST 23:2;O7	C23H36O7	[M+H-H2O]+
407.2415284						
407.2415284	407.24040.0011		2.7011	FA 21:3;O4	C21H36O6Na	[M+Na]+
	407.2404	0.0011	2.7011	ST 21:0;O6	C21H36O6Na	[M+Na]+
407.2415284						
411.3077645	411.30810.0003		0.7294	FA 22:0;O3	C22H44O5Na	[M+Na]+
	411.3081	0.0003	0.7294	MG 19:0;O	C22H44O5Na	[M+Na]+
411.3077645						
412.1781658	412.17880.0007		1.6983	ST 18:3;O4;T	C20H29NO6S	[M+H]+
	412.1788	0.0007	1.6983	NAT 18:5;O3	C20H31NO7S	[M+H-H2O]+
412.1781658						
412.1781658	412.17880.0007		1.6983	ST 18:2;O5;T	C20H31NO7S	[M+H-H2O]+
	412.1788	0.0007	1.6983	ST 20:4;O3;S	C20H26O6S	[M+NH4]+
412.1781658						
413.2482695	413.24690.0014		3.3878	NAT 19:5	C21H33NO4S	[M+NH4]+
	414.2462	0.0004	0.9656	CAR 12:0;O3	C19H37NO7Na	[M+Na]+
414.2457787						
414.2821627	414.28260.0004		0.9655	NAE 18:0;O4	C20H41NO6Na	[M+Na]+
	415.2439	0.0005	1.2041	ST 18:1;O6;G	C20H31NO7	[M+NH4]+
415.2433265						
416.2252921	416.22550.0002		0.4805	CAR 11:0;O4	C18H35NO8Na	[M+Na]+
	417.2458		0.4793	ST 25:3;O;S	C25H38O4S	[M+H-H2O]+
417.2455632						
418.1965813	418.19650.0001		0.2391	LPE 12:1	C17H34NO7PNa	[M+Na]+
	418.1965	0.0001	0.2391	LPE O-12:2;O	C17H34NO7PNa	[M+Na]+
418.1965813						
418.1965813	418.19650.0001		0.2391	SPBP 17:2;O4	C17H34NO7PNa	[M+Na]+
	419.2768	0.0006	1.4310	DG 20:2	C23H40O5Na	[M+Na]+
419.2761924						
419.2761924	419.27680.0006		1.4310	DG O-20:3;O	C23H40O5Na	[M+Na]+
	419.2768	0.0006	1.4310	FA 23:3;O3	C23H40O5Na	[M+Na]+

419.2761924						
419.2761924	419.2768	0.0006	1.4310	MG 20:3;O	C23H40O5Na	[M+Na]+
	419.2768	0.0006	1.4310	ST 23:0;O5	C23H40O5Na	[M+Na]+
419.2761924						
419.2761924	419.2752	0.0010	2.3851	CAR 13:2;O3	C20H35NO7	[M+NH4]+
	422.2149	0.0003	0.7105	CAR 13:3;O3	C20H33NO7Na	[M+Na]+
422.2146427						
422.2146427	422.2149	0.0003	0.7105	ST 18:0;O6;G	C20H33NO7Na	[M+Na]+
	423.2717	0.0003	0.7088	FA 22:2;O4	C22H40O6Na	[M+Na]+
423.2714329						
424.1952666	424.1966	0.0013	3.0646	ST 19:3;O7;G	C21H29NO8	[M+H]+
	424.1966	0.0013	3.0646	ST 19:2;O8;G	C21H31NO9	[M+H-H2O]+
424.1952666						
424.1952666	424.1942	0.0011	2.5932	CAR 12:3;O4	C19H31NO8Na	[M+Na]+
	424.1966	0.0013	3.0646	ST 21:5;O8	C21H26O8	[M+NH4]+
424.1952666						
426.2820947	426.2826	0.0005	1.1729	CAR 14:0;O2	C21H41NO6Na	[M+Na]+
	426.2826	0.0005	1.1729	NAE 19:1;O4	C21H41NO6Na	[M+Na]+
426.2820947						
427.3025803	427.303	0.0004	0.9361	FA 22:0;O4	C22H44O6Na	[M+Na]+
	428.2619	0.0003	0.7005	CAR 13:0;O3	C20H39NO7Na	[M+Na]+
428.2615234						
428.2976742	428.2983	0.0006	1.4009	NAE 19:0;O4	C21H43NO6Na	[M+Na]+
	429.1133	0.0018	4.1947	ST 21:5;O2;S	C21H26O5SK	[M+K]+
429.1150460						
429.2224794	429.2231	0.0007	1.6309	ST 18:2;O7;G	C20H29NO8	[M+NH4]+
	429.2554	0.0012	2.7955	ST 28:6;O	C28H38OK	[M+K]+
429.2566000						
429.2973105	429.2975	0.0002	0.4659	DG O-22:4	C25H42O4Na	[M+Na]+
	429.2975	0.0002	0.4659	FA 25:4;O2	C25H42O4Na	[M+Na]+
429.2973105						
429.2973105	429.2975	0.0002	0.4659	MG 22:4	C25H42O4Na	[M+Na]+
	429.2975	0.0002	0.4659	MG O-22:5;O	C25H42O4Na	[M+Na]+
429.2973105						
429.2973105	429.2975	0.0002	0.4659	ST 25:1;O4	C25H42O4Na	[M+Na]+
	436.2071	0.0010	2.2925	LPE 12:0;O	C17H36NO8PNa	[M+Na]+
436.2060275						
436.2060275	436.2071	0.0010	2.2925	LPS O-11:0	C17H36NO8PNa	[M+Na]+
	436.233	0.0008	1.8339	ST 21:3;O6;G	C23H33NO7	[M+H]+

436.2321733						
436.2321733	436.2330.0008		1.8339	ST 21:2;O7;G	C23H35NO8	[M+H-H2O]+
	436.233	0.0008	1.8339	ST 23:5;O7	C23H30O7	[M+NH4]+
436.2321733						
438.2975672	438.29790.0003		0.6845	LPC O-13:1	C21H44NO6P	[M+H]+
	438.2979	0.0003	0.6845	LPE O-16:1	C21H44NO6P	[M+H]+
438.2975672						
438.2975672	438.29790.0003		0.6845	LPC O-13:0;O	C21H46NO7P	[M+H-H2O]+
	438.2979		0.6845	LPE O-16:0;O	C21H46NO7P	[M+H-H2O]+
438.2975672						
438.2975672	438.29790.0003		0.6845	NAE 24:6;O	C26H41NO3Na	[M+Na]+
	438.2979	0.0003	0.6845	LPA O-18:2	C21H41O6P	[M+NH4]+
438.2975672						
438.2975672	438.2980.0004		0.9126	CAR 16:0	C23H45NO4K	[M+K]+
	438.298	0.0004	0.9126	NAE 21:1;O2	C23H45NO4K	[M+K]+
438.2975672						
439.2258018	439.22610.0003		0.6830	ST 20:4;O3;T	C22H31NO5S	[M+NH4]+
	439.3014	0.0005	1.1382	CAR 13:0;O4	C20H39NO8	[M+NH4]+
439.3018409						
439.3388827	439.33940.0005		1.1381	DG O-21:0;O	C24H48O5Na	[M+Na]+
	439.3394	0.0005	1.1381	FA 24:0;O3	C24H48O5Na	[M+Na]+
439.3388827						
439.3388827	439.33940.0005		1.1381	MG 21:0;O	C24H48O5Na	[M+Na]+
	440.2983	0.0006	1.3627	CAR 15:0;O2	C22H43NO6Na	[M+Na]+
440.2976626						
440.2976626	440.29830.0006		1.3627	NAE 20:1;O4	C22H43NO6Na	[M+Na]+
	440.3135	0.0003	0.6813	LPC O-13:0	C21H46NO6P	[M+H]+
440.3132441						
440.3132441	440.31350.0003		0.6813	LPE O-16:0	C21H46NO6P	[M+H]+
	440.3135	0.0003	0.6813	NAE 24:5;O	C26H43NO3Na	[M+Na]+
440.3132441						
440.3132441	440.31350.0003		0.6813	LPA O-18:1	C21H43O6P	[M+NH4]+
	440.3137	0.0004	0.9084	NAE 21:0;O2	C23H47NO4K	[M+K]+
440.3132441						
441.2566496	441.25540.0012		2.7195	ST 29:7;O	C29H38OK	[M+K]+
	441.3187	0.0012	2.7191	DG O-20:0;O2	C23H46O6Na	[M+Na]+
441.3174311						
441.3174311	441.31870.0012		2.7191	FA 23:0;O4	C23H46O6Na	[M+Na]+
	442.2775	0.0006	1.3566	CAR 14:0;O3	C21H41NO7Na	[M+Na]+

442.2768774						
443.2614299	443.2614	0.0000	0.0000	ST 27:4;O;S	C27H40O4S	[M+H-H2O]+
	443.2752	0.0021	4.7375	CAR 15:4;O3	C22H35NO7	[M+NH4]+
443.2730233						
443.2730233	443.2752	0.0021	4.7375	ST 20:1;O6;G	C22H35NO7	[M+NH4]+
	443.2711	0.0020	4.5119	ST 29:6;O	C29H40OK	[M+K]+
443.2730233						
444.1537417	444.1548	0.0011	2.4766	LPE 13:3	C18H32NO7PK	[M+K]+
	445.1679	0.0005	1.1232	ST 24:6;O4;S	C24H30O7S	[M+H-H2O]+
445.1684585						
450.2118890	450.2122	0.0004	0.8885	ST 21:4;O7;G	C23H31NO8	[M+H]+
	450.2122	0.0004	0.8885	ST 21:3;O8;G	C23H33NO9	[M+H-H2O]+
450.2118890						
450.2118890	450.2122	0.0004	0.8885	ST 23:6;O8	C23H28O8	[M+NH4]+
	450.2462	0.0013	2.8873	CAR 15:3;O3	C22H37NO7Na	[M+Na]+
450.2448764						
450.2448764	450.2462	0.0013	2.8873	ST 20:0;O6;G	C22H37NO7Na	[M+Na]+
	455.2024	0.0003	0.6590	ST 19:4;O8;G	C21H27NO9	[M+NH4]+
455.2027155						
455.2722951	455.2711	0.0012	2.6358	ST 30:7;O	C30H40OK	[M+K]+
	456.1629	0.0003	0.6577	ST 20:5;O7;G	C22H27NO8Na	[M+Na]+
456.1625690						
456.1625690	456.1629	0.0004	0.8769	BMP 11:3;O	C17H27O11P	[M+NH4]+
	456.2932	0.0004	0.8766	CAR 15:0;O3	C22H43NO7Na	[M+Na]+
456.2927310						
456.3287382	456.3296	0.0008	1.7531	NAE 21:0;O4	C23H47NO6Na	[M+Na]+
	457.2771		2.8429	ST 28:4;O;S	C28H42O4S	[M+H-H2O]+
457.2757801						
457.2877344	457.2867	0.0010	2.1868	ST 30:6;O	C30H42OK	[M+K]+
	458.2723	0.0001	0.2182	NAT 25:7	C27H41NO4S	[M+H-H2O]+
458.2724329						
458.2724329	458.2724	0.0000	0.0000	CAR 14:0;O4	C21H41NO8Na	[M+Na]+
	459.266	0.0013	2.8306	ST 29:6;O2	C29H40O2K	[M+K]+
459.2673150						
459.2927604	459.2927	0.0000	0.0000	ST 28:3;O;S	C28H44O4S	[M+H-H2O]+
	460.1004	0.0022	4.7816	ST 18:5;O8;G	C20H23NO9K	[M+K]+
460.1026418						
460.2791995	460.2798	0.0006	1.3036	LPC O-13:1	C21H44NO6PNa	[M+Na]+
	460.2798	0.0006	1.3036	LPE O-16:1	C21H44NO6PNa	[M+Na]+

460.2791995						
462.2447773	462.24620.0014		3.0287	CAR 16:4;O3	C23H37NO7Na	[M+Na]+
	462.2462	0.0014	3.0287	ST 21:1;O6;G	C23H37NO7Na	[M+Na]+
462.2447773						
462.2447773	462.24630.0015		3.2450	LPG 12:0;O	C18H37O10P	[M+NH4]+
	462.2955	0.0003	0.6489	LPC O-13:0	C21H46NO6PNa	[M+Na]+
462.2957802						
462.2957802	462.29550.0003		0.6489	LPE O-16:0	C21H46NO6PNa	[M+Na]+
	466.1378	0.0022	4.7196	ST 18:3;O8;S	C18H24O11S	[M+NH4]+
466.1355194						
466.2762327	466.27750.0013		2.7880	CAR 16:2;O3	C23H41NO7Na	[M+Na]+
	466.3655	0.0004	0.8577	Cer 29:5;O	C29H49NO2Na	[M+Na]+
466.3651761						
466.3651761	466.36550.0004		0.8577	NAE 27:5	C29H49NO2Na	[M+Na]+
	466.3657	0.0005	1.0721	NAE 24:0;O	C26H53NO3K	[M+K]+
466.3651761						
467.1372823	467.1370.0003		0.6422	ST 22:6;O6;S	C22H26O9S	[M+H]+
	467.137	0.0003	0.6422	ST 22:5;O7;S	C22H28O10S	[M+H-H2O]+
467.1372823						
468.3288165	468.32960.0007		1.4947	CAR 17:0;O2	C24H47NO6Na	[M+Na]+
	468.3296	0.0007	1.4947	NAE 22:1;O4	C24H47NO6Na	[M+Na]+
468.3288165						
468.3444660	468.34480.0004		0.8541	LPC O-15:0	C23H50NO6P	[M+H]+
	468.3448	0.0004	0.8541	LPE O-18:0	C23H50NO6P	[M+H]+
468.3444660						
468.3444660	468.34480.0003		0.6406	Cer 28:5;O2	C28H47NO3Na	[M+Na]+
	468.3448	0.0003	0.6406	NAE 26:5;O	C28H47NO3Na	[M+Na]+
468.3444660						
468.3444660	468.34480.0004		0.8541	LPA O-20:1	C23H47O6P	[M+NH4]+
	468.345	0.0005	1.0676	NAE 23:0;O2	C25H51NO4K	[M+K]+
468.3444660						
469.3481597	469.350.0018		3.8351	DG O-22:0;O2	C25H50O6Na	[M+Na]+
	469.35	0.0018	3.8351	FA 25:0;O4	C25H50O6Na	[M+Na]+
469.3481597						
471.2566480	471.25640.0003		0.6366	ST 28:6;O;S	C28H38O4S	[M+H]+
	471.2564	0.0003	0.6366	ST 28:5;O2;S	C28H40O5S	[M+H-H2O]+
471.2566480						
471.2923334	471.29270.0004		0.8487	ST 29:4;O;S	C29H44O4S	[M+H-H2O]+
	472.288	0.0011	2.3291	NAT 26:7	C28H43NO4S	[M+H-H2O]+

472.2868622						
472.2868622	472.2881	0.0012	2.5408	CAR 15:0;O4	C22H43NO8Na	[M+Na]+
	472.2857	0.0011	2.3291	NAT 21:0	C23H47NO4SK	[M+K]+
472.2868622						
473.2847440	473.2857	0.0010	2.1129	CAR 16:4;O4	C23H37NO8	[M+NH4]+
	473.2857		2.1129	ST 21:1;O7;G	C23H37NO8	[M+NH4]+
473.2847440						
476.2967653	476.2983	0.0015	3.1493	CAR 18:3;O2	C25H43NO6Na	[M+Na]+
	476.2983	0.0015	3.1493	NAE 23:4;O4	C25H43NO6Na	[M+Na]+
476.2967653						
476.2967653	476.2983	0.0015	3.1493	ST 23:0;O5;G	C25H43NO6Na	[M+Na]+
	476.2983	0.0015	3.1493	LPG O-14:0;O	C20H43O9P	[M+NH4]+
476.2967653						
478.2758423	478.2775	0.0017	3.5544	CAR 17:3;O3	C24H41NO7Na	[M+Na]+
	478.2775	0.0017	3.5544	ST 22:0;O6;G	C24H41NO7Na	[M+Na]+
478.2758423						
479.2726106	479.2744	0.0018	3.7557	LPG O-15:0	C21H45O8PNa	[M+Na]+
	480.2568	0.0009	1.8740	CAR 16:3;O4	C23H39NO8Na	[M+Na]+
480.2558867						
480.2558867	480.2568	0.0009	1.8740	ST 21:0;O7;G	C23H39NO8Na	[M+Na]+
	482.2642	0.0020	4.1471	LPC O-15:4	C23H42NO6PNa	[M+Na]+
482.2622400						
482.2622400	482.2642	0.0020	4.1471	LPE O-18:4	C23H42NO6PNa	[M+Na]+
	483.2176	0.0008	1.6556	ST 26:5;O2;S	C26H36O5SNa	[M+Na]+
483.2167812						
483.2167812	483.2159	0.0008	1.6556	ST 21:5;O5;T	C23H31NO7S	[M+NH4]+
	483.2177	0.0009	1.8625	ST 23:0;O3;S	C23H40O6SK	[M+K]+
483.2167812						
484.2844941	484.2857	0.0012	2.4779	NAT 22:1	C24H47NO4SK	[M+K]+
	484.3245	0.0005	1.0324	CAR 17:0;O3	C24H47NO7Na	[M+Na]+
484.3239635						
486.3034144	486.3036	0.0002	0.4113	NAT 27:7	C29H45NO4S	[M+H-H2O]+
	486.3037	0.0003	0.6169	CAR 16:0;O4	C23H45NO8Na	[M+Na]+
486.3034144						
487.2428955	487.2431	0.0002	0.4105	LPA 19:2;O	C22H41O8PNa	[M+Na]+
	487.2431	0.0002	0.4105	LPG O-16:3	C22H41O8PNa	[M+Na]+
487.2428955						
487.2628471	487.265	0.0022	4.5150	ST 21:2;O8;G	C23H35NO9	[M+NH4]+
	487.2609	0.0019	3.8993	ST 30:7;O3	C30H40O3K	[M+K]+

487.2628471						
487.2850342	487.2852	0.0002	0.4104	ST 27:2;O;S	C27H44O4SNa	[M+Na]+
	487.3629	0.0023	4.7193	DG 24:1;O2	C27H50O7	[M+H]+
487.3606525						
491.2360630	491.238	0.0020	4.0713	LPG 15:1	C21H41O9PNa	[M+Na]+
	491.238	0.0020	4.0713	LPG O-15:2;O	C21H41O9PNa	[M+Na]+
491.2360630						
492.2317340	492.2333	0.0016	3.2505	LPS 14:0	C20H40NO9PNa	[M+Na]+
	492.2333	0.0016	3.2505	LPS O-14:1;O	C20H40NO9PNa	[M+Na]+
492.2317340						
496.2870930	496.2881	0.0010	2.0150	CAR 17:2;O4	C24H43NO8Na	[M+Na]+
	496.2857	0.0014	2.8210	NAT 23:2	C25H47NO4SK	[M+K]+
496.2870930						
496.3030180	496.3034	0.0004	0.8060	LPC 15:1;O	C23H46NO8P	[M+H]+
	496.3034	0.0004	0.8060	LPE 18:1;O	C23H46NO8P	[M+H]+
496.3030180						
496.3030180	496.3034	0.0004	0.8060	LPS O-17:1	C23H46NO8P	[M+H]+
	496.3034	0.0004	0.8060	LPS O-17:0;O	C23H48NO9P	[M+H-H2O]+
496.3030180						
496.3030180	496.3033	0.0003	0.6045	CAR 21:6;O	C28H43NO5Na	[M+Na]+
	496.3033	0.0003	0.6045	NAE 26:7;O3	C28H43NO5Na	[M+Na]+
496.3030180						
496.3030180	496.3033	0.0003	0.6045	ST 26:3;O4;G	C28H43NO5Na	[M+Na]+
	496.3034		0.8060	LPA 20:2;O	C23H43O8P	[M+NH4]+
496.3030180						
496.3030180	496.3034	0.0004	0.8060	LPG O-17:3	C23H43O8P	[M+NH4]+
	496.3034	0.0004	0.8060	PA 20:1	C23H43O8P	[M+NH4]+
496.3030180						
496.3030180	496.3034	0.0004	0.8060	PA O-20:2;O	C23H43O8P	[M+NH4]+
	496.3035	0.0005	1.0074	CAR 18:1;O2	C25H47NO6K	[M+K]+
496.3030180						
496.3030180	496.3035	0.0005	1.0074	NAE 23:2;O4	C25H47NO6K	[M+K]+
	498.3037	0.0005	1.0034	CAR 17:1;O4	C24H45NO8Na	[M+Na]+
498.3032085						
499.1861957	499.1858	0.0004	0.8013	LPA 19:4;O	C22H37O8PK	[M+K]+
	499.2852	0.0007	1.4020	ST 28:3;O;S	C28H44O4SNa	[M+Na]+
499.2859946						
499.3585465	499.3564	0.0021	4.2054	NAT 25:4	C27H47NO4S	[M+NH4]+
	499.3629	0.0020	4.0051	DG 25:2;O2	C28H50O7	[M+H]+

499.3608990						
499.3608990	499.3629	0.0020	4.0051	ST 28:0;O7	C28H50O7	[M+H]+
	500.1891	0.0005	0.9996	ST 22:5;O8;G	C24H31NO9Na	[M+Na]+
500.1886221						
500.1918946	500.1925	0.0006	1.1995	ST 19:1;O7;T	C21H35NO9SNa	[M+Na]+
	500.2538	0.0004	0.7996	LPC O-15:3	C23H44NO6PK	[M+K]+
500.2533406						
500.2533406	500.2538	0.0004	0.7996	LPE O-18:3	C23H44NO6PK	[M+K]+
	502.254	0.0004	0.7964	LPC 14:2;O	C22H42NO8PNa	[M+Na]+
502.2536344						
502.2536344	502.254	0.0004	0.7964	LPE 17:2;O	C22H42NO8PNa	[M+Na]+
	502.254	0.0004	0.7964	LPS O-16:2	C22H42NO8PNa	[M+Na]+
502.2536344						
503.3173740	503.3165	0.0008	1.5895	ST 28:1;O;S	C28H48O4SNa	[M+Na]+
	506.2643	0.0004	0.7901	LPC 14:0	C22H46NO7PK	[M+K]+
506.2639174						
506.2639174	506.2643	0.0004	0.7901	LPC O-14:1;O	C22H46NO7PK	[M+K]+
	506.2643	0.0004	0.7901	LPE 17:0	C22H46NO7PK	[M+K]+
506.2639174						
506.2639174	506.2643	0.0004	0.7901	LPE O-17:1;O	C22H46NO7PK	[M+K]+
	507.0722	0.0009	1.7749	ST 21:6;O7;S	C21H24O10SK	[M+K]+
507.0712926						
508.3030031	508.3034	0.0004	0.7869	LPC 16:2;O	C24H46NO8P	[M+H]+
	508.3034	0.0004	0.7869	LPE 19:2;O	C24H46NO8P	[M+H]+
508.3030031						
508.3030031	508.3034	0.0004	0.7869	LPS O-18:2	C24H46NO8P	[M+H]+
	508.3034	0.0004	0.7869	LPS 18:0	C24H48NO9P	[M+H-H2O]+
508.3030031						
508.3030031	508.3034	0.0004	0.7869	LPS O-18:1;O	C24H48NO9P	[M+H-H2O]+
	508.3033	0.0003	0.5902	ST 27:4;O4;G	C29H43NO5Na	[M+Na]+
508.3030031						
508.3030031	508.3034	0.0004	0.7869	LPA 21:3;O	C24H43O8P	[M+NH4]+
	508.3034	0.0004	0.7869	LPG O-18:4	C24H43O8P	[M+NH4]+
508.3030031						
508.3030031	508.3034	0.0004	0.7869	PA 21:2	C24H43O8P	[M+NH4]+
	508.3034	0.0004	0.7869	PA O-21:3;O	C24H43O8P	[M+NH4]+
508.3030031						
508.3030031	508.3035	0.0005	0.9837	CAR 19:2;O2	C26H47NO6K	[M+K]+
	508.3035	0.0005	0.9837	NAE 24:3;O4	C26H47NO6K	[M+K]+

508.3030031						
509.2446013	509.24290.0017		3.3383	LPA O-22:5	C25H43O6PK	[M+K]+
	509.3085		3.9269	ST 26:0;O8	C26H46O8Na	[M+Na]+
509.3064971						
509.3064971	509.30440.0021		4.1233	NAT 25:7;O	C27H41NO5S	[M+NH4]+
	509.3044	0.0021	4.1233	ST 25:4;O3;T	C27H41NO5S	[M+NH4]+
509.3064971						
509.4923252	509.49280.0005		0.9814	FA 33:1;O	C33H64O3	[M+H]+
	509.4928	0.0005	0.9814	MG O-30:2	C33H64O3	[M+H]+
509.4923252						
509.4923252	509.49280.0005		0.9814	DG O-30:0	C33H66O4	[M+H-H2O]+
	509.4928	0.0005	0.9814	FA 33:0;O2	C33H66O4	[M+H-H2O]+
509.4923252						
509.4923252	509.49280.0005		0.9814	MG 30:0	C33H66O4	[M+H-H2O]+
	509.4928	0.0005	0.9814	MG O-30:1;O	C33H66O4	[M+H-H2O]+
509.4923252						
510.3033008	510.30370.0004		0.7838	CAR 18:2;O4	C25H45NO8Na	[M+Na]+
	512.0987	0.0004	0.7811	ST 18:4;O8;T	C20H27NO10SK	[M+K]+
512.0983553						
513.2177220	513.21530.0024		4.6764	ST 25:4;O6;S	C25H36O9S	[M+H]+
	513.2153	0.0024	4.6764	ST 25:3;O7;S	C25H38O10S	[M+H-H2O]+
513.2177220						
514.3489699	514.35030.0013		2.5275	CAR 22:4;O	C29H49NO5Na	[M+Na]+
	514.3503	0.0013	2.5275	Cer 29:5;O4	C29H49NO5Na	[M+Na]+
514.3489699						
514.3489699	514.35030.0013		2.5275	NAE 27:5;O3	C29H49NO5Na	[M+Na]+
	514.3503	0.0013	2.5275	ST 27:1;O4;G	C29H49NO5Na	[M+Na]+
514.3489699						
514.3489699	514.35030.0014		2.7219	LPA 21:0;O	C24H49O8P	[M+NH4]+
	514.3503	0.0014	2.7219	LPG O-18:1	C24H49O8P	[M+NH4]+
514.3489699						
514.3489699	514.35030.0014		2.7219	PA O-21:0;O	C24H49O8P	[M+NH4]+
	514.3504	0.0015	2.9163	NAE 24:0;O4	C26H53NO6K	[M+K]+
514.3489699						
514.3508044	514.35030.0005		0.9721	CAR 22:4;O	C29H49NO5Na	[M+Na]+
	514.3503	0.0005	0.9721	Cer 29:5;O4	C29H49NO5Na	[M+Na]+
514.3508044						
514.3508044	514.35030.0005		0.9721	NAE 27:5;O3	C29H49NO5Na	[M+Na]+
	514.3503	0.0005	0.9721	ST 27:1;O4;G	C29H49NO5Na	[M+Na]+

514.3508044						
514.3508044	514.35030.0005		0.9721	LPA 21:0;O	C24H49O8P	[M+NH4]+
	514.3503	0.0005	0.9721	LPG O-18:1	C24H49O8P	[M+NH4]+
514.3508044						
514.3508044	514.35030.0005		0.9721	PA O-21:0;O	C24H49O8P	[M+NH4]+
	514.3504	0.0004	0.7777	NAE 24:0;O4	C26H53NO6K	[M+K]+
514.3508044						
515.2130260	515.21230.0007		1.3587	ST 18:2;O6;GlcA	C24H34O12	[M+H]+
	515.2123	0.0007	1.3587	ST 18:3;O7;Hex	C24H34O12	[M+H]+
515.2130260						
515.2130260	515.21230.0007		1.3587	ST 18:1;O7;GlcA	C24H36O13	[M+H-H2O]+
	515.2123	0.0007	1.3587	ST 18:2;O8;Hex	C24H36O13	[M+H-H2O]+
515.2130260						
515.3308164	515.33270.0019		3.6869	CAR 19:4;O4	C26H43NO8	[M+NH4]+
	515.3327	0.0019	3.6869	ST 18:0;O3;HexNAc	C26H43NO8	[M+NH4]+
515.3308164						
515.3308164	515.33270.0019		3.6869	ST 24:1;O7;G	C26H43NO8	[M+NH4]+
516.2846723	516.28510.0004		0.7748	LPC O-16:2	C24H48NO6PK	[M+K]+
	516.2851		0.7748	LPE O-19:2	C24H48NO6PK	[M+K]+
516.2846723						
516.3135199	516.3142	0.0007	1.3558	NAT 28:7;O	C30H47NO5S	[M+H-H2O]+
	516.3142	0.0007	1.3558	ST 28:4;O3;T	C30H47NO5S	[M+H-H2O]+
516.3135199						
516.3135199	516.3142	0.0007	1.3558	ST 30:6;O;S	C30H42O4S	[M+NH4]+
	517.2885	0.0011	2.1265	LPS 15:0;O	C21H42NO10P	[M+NH4]+
517.2873143						
518.2258082	518.228	0.0022	4.2452	LPC 14:2;O	C22H42NO8PK	[M+K]+
	518.228	0.0022	4.2452	LPE 17:2;O	C22H42NO8PK	[M+K]+
518.2258082						
518.2258082	518.228	0.0022	4.2452	LPS O-16:2	C22H42NO8PK	[M+K]+
	519.2047	0.0010	1.9260	ST 27:7;O5;S	C27H34O8S	[M+H]+
519.2056874						
519.2056874	519.2047	0.0010	1.9260	ST 27:6;O6;S	C27H36O9S	[M+H-H2O]+
	520.3164	0.0005	0.9610	LPC O-16:0	C24H52NO6PK	[M+K]+
520.3158592						
520.3158592	520.3164	0.0005	0.9610	LPE O-19:0	C24H52NO6PK	[M+K]+
	521.1654	0.0025	4.7969	ST 19:5;O7;GlcA	C25H30O13	[M+H-H2O]+

521.1678862						
521.1678862	521.1701	0.0022	4.2213	PA 21:6	C24H35O8PK	[M+K]+
521.3190212	521.3214	0.0024	4.6037	LPG O-18:0	C24H51O8PNa	[M+Na]+
	521.3221	0.0001	0.1918	ST 20:3;O2;HexNAc	C28H41NO7	[M+NH4]+
521.3222112						
521.3222112	521.3221	0.0001	0.1918	ST 26:4;O6;G	C28H41NO7	[M+NH4]+
	523.1632	0.0011	2.1026	ST 25:7;O7;S	C25H30O10S	[M+H]+
523.1643400						
523.1643400	523.1632	0.0011	2.1026	ST 25:6;O8;S	C25H32O11S	[M+H-H2O]+
525.2170412	525.2153	0.0018	3.4272	ST 26:5;O6;S	C26H36O9S	[M+H]+
	525.2153	0.0018	3.4272	ST 26:4;O7;S	C26H38O10S	[M+H-H2O]+
525.2170412						
526.3343889	526.335	0.0006	1.1400	CAR 19:1;O4	C26H49NO8Na	[M+Na]+
	528.1874	0.0009	1.7039	ST 20:2;O8;T	C22H35NO10SNa	[M+Na]+
528.1883354						
529.1661204	529.1657	0.0004	0.7559	ST 26:6;O4;S	C26H34O7SK	[M+K]+
	529.1916	0.0007	1.3228	ST 18:3;O7;GlcA	C24H32O13	[M+H]+
529.1922627						
529.1922627	529.1916	0.0007	1.3228	ST 18:4;O8;Hex	C24H32O13	[M+H]+
	529.1916	0.0007	1.3228	ST 18:2;O8;GlcA	C24H34O14	[M+H-H2O]+
529.1922627						
529.2348227	529.2327	0.0021	3.9680	LPA 21:3;O	C24H43O8PK	[M+K]+
	529.2327	0.0021	3.9680	LPG O-18:4	C24H43O8PK	[M+K]+
529.2348227						
529.2348227	529.2327	0.0021	3.9680	PA 21:2	C24H43O8PK	[M+K]+
	529.2327	0.0021	3.9680	PA O-21:3;O	C24H43O8PK	[M+K]+
529.2348227						
530.2848128	530.2853	0.0005	0.9429	LPC 16:2;O	C24H46NO8PNa	[M+Na]+
	530.2853	0.0005	0.9429	LPE 19:2;O	C24H46NO8PNa	[M+Na]+
530.2848128						
530.2848128	530.2853	0.0005	0.9429	LPS O-18:2	C24H46NO8PNa	[M+Na]+
	531.2887	0.0002	0.3764	ST 27:7;O3;T	C29H39NO5S	[M+NH4]+
531.2885429						
531.2885429	531.2905	0.0019	3.5762	ST 29:2;O;S	C29H48O4SK	[M+K]+
534.2950830	534.2955	0.0004	0.7486	LPE O-22:6	C27H46NO6PNa	[M+Na]+
	534.2956		1.1230	LPC 16:0	C24H50NO7PK	[M+K]+
534.2950830						
534.2950830	534.29560.0006		1.1230	LPC O-16:1;O	C24H50NO7PK	[M+K]+

534.2950830	534.2956	0.0006	1.1230	LPE 19:0	C24H50NO7PK	[M+K]+
534.2950830	534.29560.0006		1.1230	LPE O-19:1;O	C24H50NO7PK	[M+K]+
535.1577010	535.1576	0.0001	0.1869	ST 18:3;O5;GlcA	C24H32O11K	[M+K]+
535.1577010	535.15760.0001		0.1869	ST 18:4;O6;Hex	C24H32O11K	[M+K]+
535.1620829	535.1632	0.0012	2.2423	ST 26:7;O8;S	C26H32O11S	[M+H-H2O]+
535.1620829	535.16080.0012		2.2423	ST 24:5;O7;S	C24H32O10SNa	[M+Na]+
535.1620829	535.161	0.0011	2.0555	ST 21:0;O8;S	C21H36O11SK	[M+K]+
535.1777971	535.17860.0008		1.4948	ST 18:3;O6;GlcA	C24H32O12Na	[M+Na]+
535.1777971	535.1786	0.0008	1.4948	ST 18:4;O7;Hex	C24H32O12Na	[M+Na]+
535.2984612	535.30060.0022		4.1098	LPG 18:0	C24H49O9PNa	[M+Na]+
535.2984612	535.3006	0.0022	4.1098	LPG O-18:1;O	C24H49O9PNa	[M+Na]+
536.2937025	536.29590.0022		4.1022	LPS O-17:0;O	C23H48NO9PNa	[M+Na]+
536.3320580	536.3347	0.0026	4.8477	LPC 18:2;O	C26H50NO8P	[M+H]+
536.3320580	536.33470.0026		4.8477	LPE 21:2;O	C26H50NO8P	[M+H]+
536.3320580	536.3347	0.0026	4.8477	LPS O-20:2	C26H50NO8P	[M+H]+
536.3320580	536.33470.0026		4.8477	PE 21:1	C26H50NO8P	[M+H]+
536.3320580	536.3347	0.0026	4.8477	PE O-21:2;O	C26H50NO8P	[M+H]+
536.3320580	536.33470.0026		4.8477	LPS 20:0	C26H52NO9P	[M+H-H2O]+
536.3320580	536.3347	0.0026	4.8477	LPS O-20:1;O	C26H52NO9P	[M+H-H2O]+
536.3320580	536.33470.0026		4.8477	PE 21:0;O	C26H52NO9P	[M+H-H2O]+
536.3320580	536.3347	0.0026	4.8477	PS O-20:0	C26H52NO9P	[M+H-H2O]+
536.3320580	536.33460.0026		4.8477	ST 29:4;O4;G	C31H47NO5Na	[M+Na]+
536.3320580	536.3347	0.0026	4.8477	LPA 23:3;O	C26H47O8P	[M+NH4]+
536.3320580	536.33470.0026		4.8477	LPG O-20:4	C26H47O8P	[M+NH4]+
536.3320580	536.3347	0.0026	4.8477	PA 23:2	C26H47O8P	[M+NH4]+
536.3320580	536.33470.0026		4.8477	PA O-23:3;O	C26H47O8P	[M+NH4]+
	537.1401	0.0020	3.7234	ST 23:5;O8;S	C23H30O11SNa	[M+Na]+

537.1421168						
537.3362190	537.3357	0.0006	1.1166	NAT 27:7;O	C29H45NO5S	[M+NH4]+
	537.3357	0.0006	1.1166	ST 27:4;O3;T	C29H45NO5S	[M+NH4]+
537.3362190						
537.5236130	537.5241	0.0005	0.9302	MG O-32:2	C35H68O3	[M+H]+
	537.5241	0.0005	0.9302	DG O-32:0	C35H70O4	[M+H-H2O]+
537.5236130						
537.5236130	537.5241	0.0005	0.9302	MG 32:0	C35H70O4	[M+H-H2O]+
	537.5241	0.0005	0.9302	MG O-32:1;O	C35H70O4	[M+H-H2O]+
537.5236130						
540.2085753	540.2075	0.0010	1.8511	ST 19:5;O6;GlcA	C25H30O12	[M+NH4]+
	540.3483	0.0003	0.5552	NAT 26:1	C28H55NO4SK	[M+K]+
540.3486362						
542.2040038	542.2054	0.0014	2.5820	ST 23:5;O8;T	C25H35NO10S	[M+H]+
	542.203		1.8443	ST 21:2;O8;T	C23H37NO10SNa	[M+Na]+
542.2040038						
542.2040038	542.2054	0.0014	2.5820	ST 25:6;O7;S	C25H32O10S	[M+NH4]+
	542.2547	0.0009	1.6597	ST 26:5;O4;T	C28H41NO6SNa	[M+Na]+
542.2537968						
542.2537968	542.2548	0.0010	1.8442	NAT 23:3;O3	C25H45NO7SK	[M+K]+
	542.2548	0.0010	1.8442	ST 23:0;O5;T	C25H45NO7SK	[M+K]+
542.2537968						
543.2466865	543.2484	0.0017	3.1293	LPA 22:3;O	C25H45O8PK	[M+K]+
	543.2484	0.0017	3.1293	LPG O-19:4	C25H45O8PK	[M+K]+
543.2466865						
543.2466865	543.2484	0.0017	3.1293	PA 22:2	C25H45O8PK	[M+K]+
	543.2484	0.0017	3.1293	PA O-22:3;O	C25H45O8PK	[M+K]+
543.2466865						
544.3154045	544.3164	0.0010	1.8372	LPC O-18:2	C26H52NO6PK	[M+K]+
	544.3164	0.0010	1.8372	LPE O-21:2	C26H52NO6PK	[M+K]+
544.3154045						
545.1458843	545.1452	0.0007	1.2841	ST 25:7;O7;S	C25H30O10SNa	[M+Na]+
	545.1453	0.0005	0.9172	ST 22:2;O8;S	C22H34O11SK	[M+K]+
545.1458843						
546.3323971	546.332	0.0004	0.7322	LPC O-18:1	C26H54NO6PK	[M+K]+
	546.332	0.0004	0.7322	LPE O-21:1	C26H54NO6PK	[M+K]+
546.3323971						
547.2981629	547.3006	0.0025	4.5679	LPG 19:1	C25H49O9PNa	[M+Na]+

	547.3006	0.0025	4.5679	LPG O-19:2;O	C25H49O9PNa	[M+Na]+
547.2981629						
547.2981629	547.3006	0.0025	4.5679	PA 22:0;O	C25H49O9PNa	[M+Na]+
	548.3477	0.0007	1.2766	LPC O-18:0	C26H56NO6PK	[M+K]+
548.3470003						
548.3470003	548.3477	0.0007	1.2766	LPE O-21:0	C26H56NO6PK	[M+K]+
	549.3398	0.0009	1.6383	ST 23:0;O3;Hex	C29H50O8Na	[M+Na]+
549.3388997						
549.3388997	549.3398	0.0009	1.6383	ST 29:1;O8	C29H50O8Na	[M+Na]+
	549.3422	0.0001	0.1820	ST 25:2;O2;GlcA	C31H48O8	[M+H]+
549.3421062						
549.3421062	549.3422	0.0001	0.1820	ST 25:3;O3;Hex	C31H48O8	[M+H]+
	549.3422	0.0001	0.1820	ST 25:1;O3;GlcA	C31H50O9	[M+H-H2O]+
549.3421062						
549.3421062	549.3422	0.0001	0.1820	ST 25:2;O4;Hex	C31H50O9	[M+H-H2O]+
	549.3527	0.0009	1.6383	LPG O-20:0	C26H55O8PNa	[M+Na]+
549.3517816						
552.2654667	552.2651	0.0004	0.7243	ST 18:0;O7;GlcA	C24H38O13	[M+NH4]+
	552.2651	0.0004	0.7243	ST 18:1;O8;Hex	C24H38O13	[M+NH4]+
552.2654667						
553.2728896	553.2748	0.0019	3.4341	LPI O-14:0	C23H47O11PNa	[M+Na]+
	553.2756	0.0027	4.8800	ST 19:4;O5;HexNAc	C27H37NO10	[M+NH4]+
553.2728896						
555.3614766	555.364	0.0025	4.5016	CAR 22:5;O4	C29H47NO8	[M+NH4]+
	555.364	0.0025	4.5016	ST 21:1;O3;HexNAc	C29H47NO8	[M+NH4]+
555.3614766						
555.3614766	555.364	0.0025	4.5016	ST 27:2;O7;G	C29H47NO8	[M+NH4]+
556.3452217	556.3431	0.0021	3.7746	NAT 29:6	C31H51NO4SNa	[M+Na]+
556.3452217	556.3432	0.0020	3.5949	NAT 26:1;O	C28H55NO5SK	[M+K]+
557.3412612	557.3433	0.0020	3.5885	ST 20:1;O4;HexNAc	C28H45NO9	[M+NH4]+
	557.3433		3.5885	ST 26:2;O8;G	C28H45NO9	[M+NH4]+
557.3412612						
557.3995828	557.39830.0013		2.3323	NAT 28:4;O	C30H53NO5S	[M+NH4]+
	557.3983	0.0013	2.3323	ST 28:1;O3;T	C30H53NO5S	[M+NH4]+
557.3995828						
557.4006975	557.39830.0024		4.3057	NAT 28:4;O	C30H53NO5S	[M+NH4]+
	557.3983	0.0024	4.3057	ST 28:1;O3;T	C30H53NO5S	[M+NH4]+

557.4006975						
561.1218320	561.11910.0027		4.8118	ST 25:7;O7;S	C25H30O10SK	[M+K]+
	561.4489	0.0002	0.3562	DG 30:1	C33H62O5Na	[M+Na]+
561.4487662						
561.4487662	561.44890.0002		0.3562	DG O-30:2;O	C33H62O5Na	[M+Na]+
	561.4489	0.0002	0.3562	FA 33:2;O3	C33H62O5Na	[M+Na]+
561.4487662						
561.4487662	561.44890.0002		0.3562	MG 30:2;O	C33H62O5Na	[M+Na]+
	561.4489	0.0002	0.3562	TG O-30:1	C33H62O5Na	[M+Na]+
561.4487662						
562.2744630	562.27520.0007		1.2449	LPS 18:1;O	C24H46NO10PNa	[M+Na]+
	562.3268	0.0001	0.1778	CerP 29:5;O2	C29H50NO6PNa	[M+Na]+
562.3266530						
562.3266530	562.32680.0001		0.1778	LPC O-21:6	C29H50NO6PNa	[M+Na]+
	562.3268	0.0001	0.1778	LPE O-24:6	C29H50NO6PNa	[M+Na]+
562.3266530						
562.3266530	562.32690.0003		0.5335	LPC 18:0	C26H54NO7PK	[M+K]+
	562.3269	0.0003	0.5335	LPC O-18:1;O	C26H54NO7PK	[M+K]+
562.3266530						
562.3266530	562.32690.0003		0.5335	LPE 21:0	C26H54NO7PK	[M+K]+
	562.3269	0.0003	0.5335	LPE O-21:1;O	C26H54NO7PK	[M+K]+
562.3266530						
562.3266530	562.32690.0003		0.5335	PE O-21:0	C26H54NO7PK	[M+K]+
	563.3319	0.0011	1.9527	LPG 20:0	C26H53O9PNa	[M+Na]+
563.3308646						
563.3308646	563.33190.0011		1.9527	LPG O-20:1;O	C26H53O9PNa	[M+Na]+
	563.3319	0.0011	1.9527	PG O-20:0	C26H53O9PNa	[M+Na]+
563.3308646						
563.4644585	563.46460.0001		0.1775	DG 30:0	C33H64O5Na	[M+Na]+
	563.4646	0.0001	0.1775	DG O-30:1;O	C33H64O5Na	[M+Na]+
563.4644585						
563.4644585	563.46460.0001		0.1775	FA 33:1;O3	C33H64O5Na	[M+Na]+
	563.4646	0.0001	0.1775	MG 30:1;O	C33H64O5Na	[M+Na]+
563.4644585						
563.4644585	563.46460.0001		0.1775	TG O-30:0	C33H64O5Na	[M+Na]+
	564.2473	0.0009	1.5950	ST 25:3;O8;S	C25H38O11S	[M+NH4]+
564.2482075						
564.2482075	564.24870.0005		0.8861	LPE 22:6	C27H44NO7PK	[M+K]+
	564.2487	0.0005	0.8861	PE O-22:6	C27H44NO7PK	[M+K]+

564.2482075						
565.2524526	565.25370.0012		2.1229	LPG 21:6	C27H43O9PNa	[M+Na]+
	565.2537	0.0012	2.1229	PA 24:5;O	C27H43O9PNa	[M+Na]+
565.2524526						
565.2524526	565.25370.0012		2.1229	PG O-21:6	C27H43O9PNa	[M+Na]+
	565.2538	0.0014	2.4768	BMP 18:0	C24H47O10PK	[M+K]+
565.2524526						
565.2524526	565.25380.0014		2.4768	LPG 18:1;O	C24H47O10PK	[M+K]+
	566.2643	0.0001	0.1766	LPC 19:5	C27H46NO7PK	[M+K]+
566.2642096						
566.2642096	566.26430.0001		0.1766	LPE 22:5	C27H46NO7PK	[M+K]+
	566.2643		0.1766	LPE O-22:6;O	C27H46NO7PK	[M+K]+
566.2642096						
566.2642096	566.2643	0.0001	0.1766	PE O-22:5	C27H46NO7PK	[M+K]+
	566.3663	0.0004	0.7063	CAR 22:2;O4	C29H53NO8Na	[M+Na]+
566.3659827						
568.2416044	568.2436	0.0020	3.5196	LPC 18:5;O	C26H44NO8PK	[M+K]+
	568.2436	0.0020	3.5196	LPE 21:5;O	C26H44NO8PK	[M+K]+
568.2416044						
568.2416044	568.2436	0.0020	3.5196	LPS O-20:5	C26H44NO8PK	[M+K]+
	568.2436	0.0020	3.5196	PE 21:4	C26H44NO8PK	[M+K]+
568.2416044						
568.2416044	568.2436	0.0020	3.5196	PE O-21:5;O	C26H44NO8PK	[M+K]+
	570.3637	0.0022	3.8572	ST 22:0;O4;HexNAc	C30H51NO9	[M+H]+
570.3614143						
570.3614143	570.3637	0.0022	3.8572	ST 28:1;O8;G	C30H51NO9	[M+H]+
	570.3587	0.0027	4.7339	NAT 30:6	C32H53NO4SNa	[M+Na]+
570.3614143						
570.3614143	570.3637	0.0022	3.8572	ST 24:1;O3;GlcA	C30H48O9	[M+NH4]+
	570.3637	0.0022	3.8572	ST 24:2;O4;Hex	C30H48O9	[M+NH4]+
570.3614143						
570.3614143	570.3589	0.0025	4.3832	NAT 27:1;O	C29H57NO5SK	[M+K]+
574.2879221	574.2858	0.0021	3.6567	ST 19:1;O7;HexNAc	C27H43NO12	[M+H]+
	574.2858	0.0021	3.6567	ST 19:0;O8;HexNAc	C27H45NO13	[M+H-H2O]+
574.2879221						
574.2879221	574.2858	0.0021	3.6567	ST 21:2;O6;GlcA	C27H40O12	[M+NH4]+
574.2879221	574.2858	0.0021	3.6567	ST 21:3;O7;Hex	C27H40O12	[M+NH4]+

	574.2906	0.0026		4.5273	LPC 18:2;O	C26H50NO8PK	[M+K]+
574.2879221							
574.2879221	574.2906	0.0026		4.5273	LPE 21:2;O	C26H50NO8PK	[M+K]+
	574.2906	0.0026		4.5273	LPS O-20:2	C26H50NO8PK	[M+K]+
574.2879221							
574.2879221	574.2906	0.0026		4.5273	PE 21:1	C26H50NO8PK	[M+K]+
	574.2906	0.0026		4.5273	PE O-21:2;O	C26H50NO8PK	[M+K]+
574.2879221							
576.3049014	576.3061	0.0012		2.0822	CerP 29:6;O3	C29H48NO7PNa	[M+Na]+
	576.3061	0.0012		2.0822	LPC 21:6	C29H48NO7PNa	[M+Na]+
576.3049014							
576.3049014	576.3061	0.0012		2.0822	LPE 24:6	C29H48NO7PNa	[M+Na]+
	576.3061	0.0012		2.0822	PC O-21:6	C29H48NO7PNa	[M+Na]+
576.3049014							
576.3049014	576.3061	0.0012		2.0822	PE O-24:6	C29H48NO7PNa	[M+Na]+
	576.3062	0.0013		2.2557	LPC 18:1;O	C26H52NO8PK	[M+K]+
576.3049014							
576.3049014	576.3062	0.0013		2.2557	LPE 21:1;O	C26H52NO8PK	[M+K]+
	576.3062	0.0013		2.2557	LPS O-20:1	C26H52NO8PK	[M+K]+
576.3049014							
576.3049014	576.3062	0.0013		2.2557	PE 21:0	C26H52NO8PK	[M+K]+
	576.3062	0.0013		2.2557	PE O-21:1;O	C26H52NO8PK	[M+K]+
576.3049014							
578.2045232	578.203	0.0015		2.5942	ST 24:5;O8;T	C26H37NO10SNa	[M+Na]+
583.2841137	583.2854	0.0013		2.2288	LPI O-15:0;O	C24H49O12PNa	[M+Na]+
584.2855913	584.2854	0.0002		0.3423	ST 24:6;O5;HexNAc	C32H43NO10	[M+H-H2O]+
	584.5248		0.1711		CAR 28:0;O	C35H69NO5	[M+H]+
584.5247034							
584.5247034	584.5248	0.0001		0.1711	Cer 35:1;O4	C35H69NO5	[M+H]+
	584.5248	0.0001		0.1711	NAE 33:1;O3	C35H69NO5	[M+H]+
584.5247034							
584.5247034	584.5248	0.0001		0.1711	Cer 35:0;O5	C35H71NO6	[M+H-H2O]+
	584.5248	0.0001		0.1711	NAE 33:0;O4	C35H71NO6	[M+H-H2O]+
584.5247034							
584.5247034	584.5248	0.0001		0.1711	DG 32:1	C35H66O5	[M+NH4]+
	584.5248	0.0001		0.1711	DG O-32:2;O	C35H66O5	[M+NH4]+

584.5247034						
584.5247034	584.5248	0.0001	0.1711	MG 32:2;O	C35H66O5	[M+NH4]+
584.5247034	584.5248	0.0001	0.1711	TG O-32:1	C35H66O5	[M+NH4]+
	585.3746	0.0024	4.0999	ST 22:1;O4;HexNAc	C30H49NO9	[M+NH4]+
585.3721158						
585.3721158	585.3746	0.0024	4.0999	ST 28:2;O8;G	C30H49NO9	[M+NH4]+
586.2295382	586.2317	0.0021	3.5822	ST 27:6;O8;S	C27H36O11S	[M+NH4]+
	586.2317	0.0010	1.7058	ST 27:6;O8;S	C27H36O11S	[M+NH4]+
586.2306359						
586.3321974	586.3327	0.0005	0.8528	NAT 30:6	C32H53NO4SK	[M+K]+
	587.2334	0.0004	0.6812	ST 21:3;O8;GlcA	C27H38O14	[M+H]+
587.2338225						
588.2460760	588.2473	0.0012	2.0400	ST 27:5;O8;S	C27H38O11S	[M+NH4]+
	588.3061	0.0001	0.1700	LPE 25:7	C30H48NO7PNa	[M+Na]+
588.3059182						
588.3059182	588.3061	0.0001	0.1700	PE O-25:7	C30H48NO7PNa	[M+Na]+
	588.3062	0.0003	0.5099	LPC 19:2;O	C27H52NO8PK	[M+K]+
588.3059182						
588.3059182	588.3062	0.0003	0.5099	LPE 22:2;O	C27H52NO8PK	[M+K]+
	588.3062	0.0003	0.5099	LPS O-21:2	C27H52NO8PK	[M+K]+
588.3059182						
588.3059182	588.3062	0.0003	0.5099	PE 22:1	C27H52NO8PK	[M+K]+
	588.3062	0.0003	0.5099	PE O-22:2;O	C27H52NO8PK	[M+K]+
588.3059182						
589.3085840	589.3112	0.0026	4.4119	BMP 21:1	C27H51O10PNa	[M+Na]+
	589.3112	0.0026	4.4119	LPG 21:2;O	C27H51O10PNa	[M+Na]+
589.3085840						
589.3085840	589.3112	0.0026	4.4119	PG 21:1	C27H51O10PNa	[M+Na]+
	589.3112	0.0026	4.4119	PG O-21:2;O	C27H51O10PNa	[M+Na]+
589.3085840						
589.4797142	589.4802	0.0005	0.8482	DG 32:1	C35H66O5Na	[M+Na]+
	589.4802	0.0005	0.8482	DG O-32:2;O	C35H66O5Na	[M+Na]+
589.4797142						
589.4797142	589.4802	0.0005	0.8482	MG 32:2;O	C35H66O5Na	[M+Na]+
	589.4802	0.0005	0.8482	TG O-32:1	C35H66O5Na	[M+Na]+
589.4797142						
589.4803551	589.4802	0.0001	0.1696	DG 32:1	C35H66O5Na	[M+Na]+
	589.4802	0.0001	0.1696	DG O-32:2;O	C35H66O5Na	[M+Na]+

589.4803551						
589.4803551	589.4802	0.0001	0.1696	MG 32:2;O	C35H66O5Na	[M+Na]+
	589.4802	0.0001	0.1696	TG O-32:1	C35H66O5Na	[M+Na]+
589.4803551						
590.4834383	590.4813	0.0022	3.7258	NAT 31:0;O	C33H67NO5S	[M+H]+
591.4746881	591.4748	0.0001	0.1691	LPA O-30:1	C33H67O6P	[M+H]+
	591.4748		0.1691	LPA O-30:0;O	C33H69O7P	[M+H-H2O]+
591.4746881						
591.4746881	591.47480.0001		0.1691	CE 11:1;O	C38H64O3Na	[M+Na]+
	591.4749	0.0002	0.3381	DG O-32:1	C35H68O4K	[M+K]+
591.4746881						
591.4746881	591.47490.0002		0.3381	MG 32:1	C35H68O4K	[M+K]+
	591.4749	0.0002	0.3381	MG O-32:2;O	C35H68O4K	[M+K]+
591.4746881						
591.4957920	591.49590.0001		0.1691	DG 32:0	C35H68O5Na	[M+Na]+
	591.4959	0.0001	0.1691	DG O-32:1;O	C35H68O5Na	[M+Na]+
591.4957920						
591.4957920	591.49590.0001		0.1691	MG 32:1;O	C35H68O5Na	[M+Na]+
	591.4959	0.0001	0.1691	TG O-32:0	C35H68O5Na	[M+Na]+
591.4957920						
593.4908570	593.49040.0004		0.6740	LPA O-30:0	C33H69O6P	[M+H]+
	593.4904	0.0004	0.6740	CE 11:0;O	C38H66O3Na	[M+Na]+
593.4908570						
593.4908570	593.49060.0003		0.5055	DG O-32:0	C35H70O4K	[M+K]+
	593.4906	0.0003	0.5055	MG 32:0	C35H70O4K	[M+K]+
593.4908570						
593.4908570	593.49060.0003		0.5055	MG O-32:1;O	C35H70O4K	[M+K]+
	597.3746	0.0024	4.0176	ST 23:2;O4;HexNAc	C31H49NO9	[M+NH4]+
597.3721310						
597.3721310	597.37460.0024		4.0176	ST 29:3;O8;G	C31H49NO9	[M+NH4]+
	599.3902	0.0028	4.6714	ST 23:1;O4;HexNAc	C31H51NO9	[M+NH4]+
599.3874479						
599.3874479	599.39020.0028		4.6714	ST 29:2;O8;G	C31H51NO9	[M+NH4]+
602.2046757	602.2030.0016		2.6569	ST 26:7;O8;T	C28H37NO10SNa	[M+Na]+
	602.2443	0.0001	0.1660	ST 21:4;O8;GlcA	C27H36O14	[M+NH4]+
602.2444533						
602.3238734	602.32410.0002		0.3320	PE 28:8	C33H50NO8P	[M+H-H2O]+
	605.4541	0.0000	0.0000	LPA 30:1	C33H65O7P	[M+H]+

605.4540747						
605.4540747	605.45410.0000		0.0000	LPA O-30:2;O	C33H65O7P	[M+H]+
	605.4541	0.0000	0.0000	PA O-30:1	C33H65O7P	[M+H]+
605.4540747						
605.4540747	605.45410.0000		0.0000	LPA 30:0;O	C33H67O8P	[M+H-H2O]+
	605.4541	0.0000	0.0000	LPG O-27:1	C33H67O8P	[M+H-H2O]+
605.4540747						
605.4540747	605.45410.0000		0.0000	PA O-30:0;O	C33H67O8P	[M+H-H2O]+
	605.454	0.0000	0.0000	CE 11:2;O2	C38H62O4Na	[M+Na]+
605.4540747						
605.4540747	605.4540.0000		0.0000	DG O-35:7	C38H62O4Na	[M+Na]+
	605.4542	0.0001	0.1652	DG 32:1	C35H66O5K	[M+K]+
605.4540747						
605.4540747	605.45420.0001		0.1652	DG O-32:2;O	C35H66O5K	[M+K]+
	605.4542	0.0001	0.1652	MG 32:2;O	C35H66O5K	[M+K]+
605.4540747						
605.4540747	605.45420.0001		0.1652	TG O-32:1	C35H66O5K	[M+K]+
	606.3378	0.0025	4.1231	LPS 21:0;O	C27H54NO10PNa	[M+Na]+
606.3352852						
606.3352852	606.33780.0025		4.1231	PS O-21:0;O	C27H54NO10PNa	[M+Na]+
	606.4551	0.0009	1.4840	NAT 34:5	C36H63NO4S	[M+H]+
606.4559021						
606.4559021	606.45510.0009		1.4840	NAT 34:4;O	C36H65NO5S	[M+H-H2O]+
	607.4697		0.8231	LPA 30:0	C33H67O7P	[M+H]+
607.4691926						
607.4691926	607.4697	0.0005	0.8231	LPA O-30:1;O	C33H67O7P	[M+H]+
	607.4697	0.0005	0.8231	PA O-30:0	C33H67O7P	[M+H]+
607.4691926						
607.4691926	607.4697	0.0005	0.8231	LPG O-27:0	C33H69O8P	[M+H-H2O]+
	607.4697	0.0005	0.8231	CE 11:1;O2	C38H64O4Na	[M+Na]+
607.4691926						
607.4691926	607.4697	0.0005	0.8231	DG O-35:6	C38H64O4Na	[M+Na]+
	607.4681	0.0011	1.8108	CAR 28:5;O2	C35H59NO6	[M+NH4]+
607.4691926						
607.4691926	607.4681	0.0011	1.8108	Cer 35:6;O5	C35H59NO6	[M+NH4]+
607.4691926	607.4681	0.0011	1.8108	NAE 33:6;O4	C35H59NO6	[M+NH4]+
	607.4681	0.0011	1.8108	ST 27:1;O;HexNAc	C35H59NO6	[M+NH4]+
607.4691926						
607.4691926	607.4698	0.0006	0.9877	DG 32:0	C35H68O5K	[M+K]+

607.4691926	607.4698	0.0006	0.9877	DG O-32:1;O	C35H68O5K	[M+K]+
607.4691926	607.4698	0.0006	0.9877	MG 32:1;O	C35H68O5K	[M+K]+
607.4691926	607.4698	0.0006	0.9877	TG O-32:0	C35H68O5K	[M+K]+
607.4705401	607.4697	0.0008	1.3169	LPA 30:0	C33H67O7P	[M+H]+
607.4705401	607.4697	0.0008	1.3169	LPA O-30:1;O	C33H67O7P	[M+H]+
607.4705401	607.4697	0.0008	1.3169	PA O-30:0	C33H67O7P	[M+H]+
607.4705401	607.4697	0.0008	1.3169	LPG O-27:0	C33H69O8P	[M+H-H2O]+
607.4705401	607.4697	0.0009	1.4816	CE 11:1;O2	C38H64O4Na	[M+Na]+
607.4705401	607.4697	0.0009	1.4816	DG O-35:6	C38H64O4Na	[M+Na]+
607.4705401	607.4714	0.0009	1.4816	NAT 30:1;O2	C32H63NO6S	[M+NH4]+
607.4705401	607.4698	0.0007	1.1523	DG 32:0	C35H68O5K	[M+K]+
607.4705401	607.4698	0.0007	1.1523	DG O-32:1;O	C35H68O5K	[M+K]+
607.4705401	607.4698	0.0007	1.1523	MG 32:1;O	C35H68O5K	[M+K]+
607.4705401	607.4698	0.0007	1.1523	TG O-32:0	C35H68O5K	[M+K]+
608.3056736	608.3065	0.0009	1.4795	ST 23:4;O6;HexNAc	C31H45NO11	[M+H]+
608.3056736	608.3065	0.0009	1.4795	ST 23:3;O7;HexNAc	C31H47NO12	[M+H-H2O]+
608.3056736	608.3041	0.0015	2.4659	ST 21:1;O6;HexNAc	C29H47NO11Na	[M+Na]+
608.3056736	608.3065	0.0009	1.4795	ST 25:5;O5;GlcA	C31H42O11	[M+NH4]+
608.3056736	608.3065	0.0009	1.4795	ST 25:6;O6;Hex	C31H42O11	[M+NH4]+
613.4032968	613.4059	0.0026	4.2386	ST 24:1;O4;HexNAc	C32H53NO9	[M+NH4]+
613.4032968	613.4059	0.0026	4.2386	ST 30:2;O8;G	C32H53NO9	[M+NH4]+
614.3226420	614.3219	0.0008	1.3022	CerP 29:3;O4	C29H54NO8PK	[M+K]+
614.3226420	614.3219	0.0008	1.3022	LPC 21:3;O	C29H54NO8PK	[M+K]+
614.3226420	614.3219	0.0008	1.3022	LPE 24:3;O	C29H54NO8PK	[M+K]+
614.3226420	614.3219	0.0008	1.3022	LPS O-23:3	C29H54NO8PK	[M+K]+
	614.3219		1.3022	PC 21:2	C29H54NO8PK	[M+K]+

614.3226420						
614.3226420	614.3219	0.0008	1.3022	PC O-21:3;O	C29H54NO8PK	[M+K]+
	614.3219	0.0008	1.3022	PE 24:2	C29H54NO8PK	[M+K]+
614.3226420						
614.3226420	614.3219	0.0008	1.3022	PE O-24:3;O	C29H54NO8PK	[M+K]+
	616.3374	0.0006	0.9735	LPE 27:7	C32H52NO7PNa	[M+Na]+
616.3367637						
616.3367637	616.3374	0.0006	0.9735	PE O-27:7	C32H52NO7PNa	[M+Na]+
	616.3375	0.0007	1.1357	CerP 29:2;O4	C29H56NO8PK	[M+K]+
616.3367637						
616.3367637	616.3375	0.0007	1.1357	LPC 21:2;O	C29H56NO8PK	[M+K]+
	616.3375	0.0007	1.1357	LPE 24:2;O	C29H56NO8PK	[M+K]+
616.3367637						
616.3367637	616.3375	0.0007	1.1357	LPS O-23:2	C29H56NO8PK	[M+K]+
	616.3375	0.0007	1.1357	PC 21:1	C29H56NO8PK	[M+K]+
616.3367637						
616.3367637	616.3375	0.0007	1.1357	PC O-21:2;O	C29H56NO8PK	[M+K]+
	616.3375	0.0007	1.1357	PE 24:1	C29H56NO8PK	[M+K]+
616.3367637						
616.3367637	616.3375	0.0007	1.1357	PE O-24:2;O	C29H56NO8PK	[M+K]+
	617.3602	0.0016	2.5917	PA O-32:9	C35H53O7P	[M+H]+
617.3617197						
617.3617197	617.3602	0.0016	2.5917	PA 32:7	C35H55O8P	[M+H-H2O]+
	617.3602	0.0016	2.5917	PA O-32:8;O	C35H55O8P	[M+H-H2O]+
617.3617197						
617.3617197	617.3603	0.0014	2.2677	DG 34:9	C37H54O5K	[M+K]+
	617.3603	0.0014	2.2677	TG O-34:9	C37H54O5K	[M+K]+
617.3617197						
617.5113599	617.5115	0.0002	0.3239	DG 34:1	C37H70O5Na	[M+Na]+
	617.5115	0.0002	0.3239	DG O-34:2;O	C37H70O5Na	[M+Na]+
617.5113599						
617.5113599	617.5115	0.0002	0.3239	MG 34:2;O	C37H70O5Na	[M+Na]+
	617.5115	0.0002	0.3239	TG O-34:1	C37H70O5Na	[M+Na]+
617.5113599						
618.3033573	618.3038	0.0004	0.6469	PS 23:4;O	C29H48NO11P	[M+H]+
	618.3037	0.0004	0.6469	ST 26:7;O3;HexNAc	C34H45NO8Na	[M+Na]+
618.3033573						
618.3033573	618.3038	0.0004	0.6469	BMP 23:6;O	C29H45O11P	[M+NH4]+
618.3033573	618.3038	0.0004	0.6469	PG 23:6;O	C29H45O11P	[M+NH4]+

618.3033573	618.3039	0.0005	0.8087	ST 23:2;O4;HexNAc	C31H49NO9K	[M+K]+
618.3033573	618.3039	0.0005	0.8087	ST 29:3;O8;G	C31H49NO9K	[M+K]+
618.3650608	618.3637	0.0014	2.2640	ST 26:4;O4;HexNAc	C34H51NO9	[M+H]+
618.3650608	618.367	0.0020	3.2343	ST 29:1;O7;T	C31H55NO9S	[M+H]+
618.3650608	618.3637	0.0014	2.2640	ST 26:3;O5;HexNAc	C34H53NO10	[M+H-H2O]+
618.3650608	618.367	0.0020	3.2343	ST 29:0;O8;T	C31H57NO10S	[M+H-H2O]+
618.3650608	618.3637	0.0014	2.2640	ST 28:5;O3;GlcA	C34H48O9	[M+NH4]+
618.3650608	618.3637	0.0014	2.2640	ST 28:6;O4;Hex	C34H48O9	[M+NH4]+
618.3650608	618.3637	0.0014	2.2640	TG 31:8;O3	C34H48O9	[M+NH4]+
619.3680979	619.3688		1.1302	ST 25:0;O7;Hex	C31H54O12	[M+H]+
620.3525307	620.3534	0.0009	1.4508	CerP 28:1;O6	C28H56NO10PNa	[M+Na]+
620.3525307	620.3534	0.0009	1.4508	LPS 22:0;O	C28H56NO10PNa	[M+Na]+
620.3525307	620.3534	0.0009	1.4508	PS O-22:0;O	C28H56NO10PNa	[M+Na]+
625.4032252	625.4059	0.0026	4.1573	ST 25:2;O4;HexNAc	C33H53NO9	[M+NH4]+
628.2850863	628.2857	0.0006	0.9550	PS 22:3;O	C28H48NO11PNa	[M+Na]+
628.3369038	628.3374	0.0005	0.7958	PE O-28:8	C33H52NO7PNa	[M+Na]+
628.3369038	628.3375	0.0006	0.9549	CerP 30:3;O4	C30H56NO8PK	[M+K]+
628.3369038	628.3375	0.0006	0.9549	LPC 22:3;O	C30H56NO8PK	[M+K]+
628.3369038	628.3375	0.0006	0.9549	LPE 25:3;O	C30H56NO8PK	[M+K]+
628.3369038	628.3375	0.0006	0.9549	LPS O-24:3	C30H56NO8PK	[M+K]+
628.3369038	628.3375	0.0006	0.9549	PC 22:2	C30H56NO8PK	[M+K]+
628.3369038	628.3375	0.0006	0.9549	PC O-22:3;O	C30H56NO8PK	[M+K]+
628.3369038	628.3375	0.0006	0.9549	PE 25:2	C30H56NO8PK	[M+K]+
628.3369038	628.3375	0.0006	0.9549	PE O-25:3;O	C30H56NO8PK	[M+K]+
630.3540195	630.3554	0.0014	2.2210	PE 30:8	C35H54NO8P	[M+H-H2O]+

630.3540195	630.353	0.0010	1.5864	LPC 25:7	C33H54NO7PNa	[M+Na]+
630.3540195	630.353	0.0010	1.5864	LPE 28:7	C33H54NO7PNa	[M+Na]+
630.3540195	630.353	0.0010	1.5864	PC O-25:7	C33H54NO7PNa	[M+Na]+
630.3540195	630.353	0.0010	1.5864	PE O-28:7	C33H54NO7PNa	[M+Na]+
630.3540195	630.3532	0.0009	1.4278	CerP 30:2;O4	C30H58NO8PK	[M+K]+
630.3540195	630.3532	0.0009	1.4278	LPC 22:2;O	C30H58NO8PK	[M+K]+
630.3540195	630.3532	0.0009	1.4278	LPE 25:2;O	C30H58NO8PK	[M+K]+
630.3540195	630.3532	0.0009	1.4278	LPS O-24:2	C30H58NO8PK	[M+K]+
630.3540195	630.3532	0.0009	1.4278	PC 22:1	C30H58NO8PK	[M+K]+
630.3540195	630.3532	0.0009	1.4278	PC O-22:2;O	C30H58NO8PK	[M+K]+
630.3540195	630.3532	0.0009	1.4278	PE 25:1	C30H58NO8PK	[M+K]+
630.3540195	630.3532	0.0009	1.4278	PE O-25:2;O	C30H58NO8PK	[M+K]+
630.3875097	630.3878	0.0003	0.4759	EPC 29:6;O4	C31H53N2O8P	[M+NH4]+
633.4853143	633.4854	0.0000	0.0000	LPA 32:1	C35H69O7P	[M+H]+
633.4853143	633.4854	0.0000	0.0000	LPA O-32:2;O	C35H69O7P	[M+H]+
633.4853143	633.4854	0.0000	0.0000	PA O-32:1	C35H69O7P	[M+H]+
633.4853143	633.4854	0.0000	0.0000	LPA 32:0;O	C35H71O8P	[M+H-H2O]+
633.4853143	633.4854	0.0000	0.0000	LPG O-29:1	C35H71O8P	[M+H-H2O]+
633.4853143	633.4854	0.0000	0.0000	PA O-32:0;O	C35H71O8P	[M+H-H2O]+
633.4853143	633.4853	0.0000	0.0000	CE 13:2;O2	C40H66O4Na	[M+Na]+
633.4853143	633.4853	0.0000	0.0000	DG O-37:7	C40H66O4Na	[M+Na]+
633.4853143	633.4855		0.3157	DG 34:1	C37H70O5K	[M+K]+
633.4853143	633.4855	0.0002	0.3157	DG O-34:2;O	C37H70O5K	[M+K]+
633.4853143	633.4855	0.0002	0.3157	MG 34:2;O	C37H70O5K	[M+K]+
633.4853143	633.4855	0.0002	0.3157	TG O-34:1	C37H70O5K	[M+K]+
	634.3174	0.0013	2.0494	NAT 30:6;O3	C32H53NO7SK	[M+K]+

634.3161302						
634.3161302	634.3174	0.0013	2.0494	ST 30:3;O5;T	C32H53NO7SK	[M+K]+
	634.3269	0.0025	3.9412	CerP 32:6;O3	C32H54NO7PK	[M+K]+
634.3294522						
634.3294522	634.3269	0.0025	3.9412	LPC 24:6	C32H54NO7PK	[M+K]+
	634.3269	0.0025	3.9412	LPE 27:6	C32H54NO7PK	[M+K]+
634.3294522						
634.3294522	634.3269	0.0025	3.9412	LPE O-27:7;O	C32H54NO7PK	[M+K]+
	634.3269	0.0025	3.9412	PC O-24:6	C32H54NO7PK	[M+K]+
634.3294522						
634.3294522	634.3269	0.0025	3.9412	PE O-27:6	C32H54NO7PK	[M+K]+
	634.3327	0.0020	3.1529	PS 22:0;O	C28H54NO11PNa	[M+Na]+
634.3306982						
634.4883133	634.4889	0.0005	0.7880	HexCer 28:0;O3	C34H67NO9	[M+H]+
	634.4889	0.0005	0.7880	TG 31:0;O3	C34H64O9	[M+NH4]+
634.4883133						
635.3262609	635.3273	0.0011	1.7314	ST 24:0;O8;GlcA	C30H50O14	[M+H]+
	635.3343	0.0002	0.3148	PA 31:8;O	C34H51O9P	[M+H]+
635.3341110						
635.3341110	635.3343	0.0002	0.3148	BMP 28:7	C34H53O10P	[M+H-H2O]+
	635.3343	0.0002	0.3148	PG 28:7	C34H53O10P	[M+H-H2O]+
635.3341110						
635.3341110	635.3343	0.0002	0.3148	PG O-28:8;O	C34H53O10P	[M+H-H2O]+
	635.3345	0.0003	0.4722	DG 33:9;O2	C36H52O7K	[M+K]+
635.3341110						
635.3341110	635.3345	0.0003	0.4722	ST 30:5;O;GlcA	C36H52O7K	[M+K]+
	635.3345	0.0003	0.4722	ST 30:6;O2;Hex	C36H52O7K	[M+K]+
635.3341110						
635.3341110	635.3345	0.0003	0.4722	TG 33:8;O	C36H52O7K	[M+K]+
635.3341110	635.3345	0.0003	0.4722	TG O-33:9;O2	C36H52O7K	[M+K]+
	635.3749	0.0008	1.2591	ST 22:0;O7;HexNAc	C30H51NO12	[M+NH4]+
635.3741220						
635.5010696	635.501	0.0001	0.1574	LPA 32:0	C35H71O7P	[M+H]+
635.5010696	635.501	0.0001	0.1574	LPA O-32:1;O	C35H71O7P	[M+H]+
	635.501	0.0001	0.1574	PA O-32:0	C35H71O7P	[M+H]+
635.5010696						
635.5010696	635.501	0.0001	0.1574	LPG O-29:0	C35H73O8P	[M+H-H2O]+
	635.501	0.0001	0.1574	CE 13:1;O2	C40H68O4Na	[M+Na]+

635.5010696						
635.5010696	635.501	0.0001	0.1574	DG O-37:6	C40H68O4Na	[M+Na]+
	635.5011	0.0001	0.1574	DG 34:0	C37H72O5K	[M+K]+
635.5010696						
635.5010696	635.5011	0.0001	0.1574	DG O-34:1;O	C37H72O5K	[M+K]+
	635.5011	0.0001	0.1574	MG 34:1;O	C37H72O5K	[M+K]+
635.5010696						
635.5010696	635.5011	0.0001	0.1574	TG O-34:0	C37H72O5K	[M+K]+
639.3437022	639.3421	0.0016	2.5026	PA O-32:9	C35H53O7PNa	[M+Na]+
	639.3423		2.1898	LPA 29:4;O	C32H57O8PK	[M+K]+
639.3437022						
639.3437022	639.3423	0.0014	2.1898	LPG O-26:5	C32H57O8PK	[M+K]+
	639.3423	0.0014	2.1898	PA 29:3	C32H57O8PK	[M+K]+
639.3437022						
639.3437022	639.3423	0.0014	2.1898	PA O-29:4;O	C32H57O8PK	[M+K]+
	640.3796	0.0024	3.7478	NAT 34:7	C36H59NO4SK	[M+K]+
640.3771987						
641.3523841	641.3532	0.0008	1.2474	ST 27:2;O6;GlcA	C33H52O12	[M+H]+
	641.3532	0.0008	1.2474	ST 27:3;O7;Hex	C33H52O12	[M+H]+
641.3523841						
641.3523841	641.3532	0.0008	1.2474	ST 27:1;O7;GlcA	C33H54O13	[M+H-H2O]+
	641.3532	0.0008	1.2474	ST 27:2;O8;Hex	C33H54O13	[M+H-H2O]+
641.3523841						
641.3523841	641.3537	0.0014	2.1829	EPC 28:4;O5	C30H55N2O9PNa	[M+Na]+
	641.4153	0.0001	0.1559	LPA 30:2;O	C33H63O8PNa	[M+Na]+
641.4151630						
641.4151630	641.4153	0.0001	0.1559	LPG O-27:3	C33H63O8PNa	[M+Na]+
	641.4153	0.0001	0.1559	PA 30:1	C33H63O8PNa	[M+Na]+
641.4151630						
641.4151630	641.4153	0.0001	0.1559	PA O-30:2;O	C33H63O8PNa	[M+Na]+
	642.353	0.0015	2.3352	PE O-29:8	C34H54NO7PNa	[M+Na]+
642.3545372						
642.3545372	642.3532	0.0014	2.1795	CerP 31:3;O4	C31H58NO8PK	[M+K]+
	642.3532	0.0014	2.1795	LPC 23:3;O	C31H58NO8PK	[M+K]+
642.3545372						
642.3545372	642.3532	0.0014	2.1795	LPE 26:3;O	C31H58NO8PK	[M+K]+
	642.3532	0.0014	2.1795	LPS O-25:3	C31H58NO8PK	[M+K]+
642.3545372						
642.3545372	642.3532	0.0014	2.1795	PC 23:2	C31H58NO8PK	[M+K]+

642.3545372	642.3532	0.0014	2.1795	PC O-23:3;O	C31H58NO8PK	[M+K]+
642.3545372	642.3532	0.0014	2.1795	PE 26:2	C31H58NO8PK	[M+K]+
642.3545372	642.3532	0.0014	2.1795	PE O-26:3;O	C31H58NO8PK	[M+K]+
644.3684020	644.3687	0.0003	0.4656	LPC 26:7	C34H56NO7PNa	[M+Na]+
644.3684020	644.3687	0.0003	0.4656	LPE 29:7	C34H56NO7PNa	[M+Na]+
644.3684020	644.3687	0.0003	0.4656	PC O-26:7	C34H56NO7PNa	[M+Na]+
644.3684020	644.3687	0.0003	0.4656	PE O-29:7	C34H56NO7PNa	[M+Na]+
644.3684020	644.3688	0.0004	0.6208	CerP 31:2;O4	C31H60NO8PK	[M+K]+
644.3684020	644.3688	0.0004	0.6208	LPC 23:2;O	C31H60NO8PK	[M+K]+
644.3684020	644.3688	0.0004	0.6208	LPE 26:2;O	C31H60NO8PK	[M+K]+
644.3684020	644.3688	0.0004	0.6208	LPS O-25:2	C31H60NO8PK	[M+K]+
644.3684020	644.3688	0.0004	0.6208	PC 23:1	C31H60NO8PK	[M+K]+
644.3684020	644.3688	0.0004	0.6208	PC O-23:2;O	C31H60NO8PK	[M+K]+
644.3684020	644.3688	0.0004	0.6208	PE 26:1	C31H60NO8PK	[M+K]+
644.3684020	644.3688	0.0004	0.6208	PE O-26:2;O	C31H60NO8PK	[M+K]+
645.3721056	645.3738	0.0017	2.6341	BMP 25:1	C31H59O10PNa	[M+Na]+
645.3721056	645.3738	0.0017	2.6341	LPG 25:2;O	C31H59O10PNa	[M+Na]+
645.3721056	645.3738	0.0017	2.6341	PG 25:1	C31H59O10PNa	[M+Na]+
645.3721056	645.3738		2.6341	PG O-25:2;O	C31H59O10PNa	[M+Na]+
646.3170701	646.3174	0.0004	0.6189	NAT 31:7;O3	C33H53NO7SK	[M+K]+
648.3652702	648.366	0.0007	1.0796	PE 30:8	C35H54NO8P	[M+H]+
648.3652702	648.366	0.0007	1.0796	LPS 29:7	C35H56NO9P	[M+H-H2O]+
648.3652702	648.366	0.0007	1.0796	PC 27:7;O	C35H56NO9P	[M+H-H2O]+
648.3652702	648.366	0.0007	1.0796	PE 30:7;O	C35H56NO9P	[M+H-H2O]+
648.3652702	648.366	0.0007	1.0796	PS O-29:7	C35H56NO9P	[M+H-H2O]+
648.3652702	648.366	0.0007	1.0796	PA 32:9	C35H51O8P	[M+NH4]+

648.3652702	648.3661	0.0008	1.2339	ST 29:5;O;HexNAc	C37H55NO6K	[M+K]+
653.3252791	653.3272	0.0020	3.0613	LPI 19:0;O	C28H55O13PNa	[M+Na]+
653.3972896	653.3965	0.0007	1.0713	PA O-36:10	C39H59O7P	[M+H-H2O]+
654.3277790	654.3273	0.0005	0.7641	ST 28:7;O6;HexNAc	C36H49NO11	[M+H-H2O]+
654.3277790	654.3282	0.0005	0.7641	ST 29:2;O8;T	C31H53NO10SNa	[M+Na]+
654.3539431	654.3532	0.0008	1.2226	CerP 32:4;O4	C32H58NO8PK	[M+K]+
654.3539431	654.3532	0.0008	1.2226	LPC 24:4;O	C32H58NO8PK	[M+K]+
654.3539431	654.3532	0.0008	1.2226	LPE 27:4;O	C32H58NO8PK	[M+K]+
654.3539431	654.3532	0.0008	1.2226	LPS O-26:4	C32H58NO8PK	[M+K]+
654.3539431	654.3532	0.0008	1.2226	PC 24:3	C32H58NO8PK	[M+K]+
654.3539431	654.3532	0.0008	1.2226	PC O-24:4;O	C32H58NO8PK	[M+K]+
654.3539431	654.3532	0.0008	1.2226	PE 27:3	C32H58NO8PK	[M+K]+
654.3539431	654.3532	0.0008	1.2226	PE O-27:4;O	C32H58NO8PK	[M+K]+
655.3174635	655.316	0.0014	2.1364	PA O-32:9	C35H53O7PK	[M+K]+
655.4672325	655.4673	0.0001	0.1526	LPA 32:1	C35H69O7PNa	[M+Na]+
655.4672325	655.4673	0.0001	0.1526	LPA O-32:2;O	C35H69O7PNa	[M+Na]+
655.4672325	655.4673	0.0001	0.1526	PA O-32:1	C35H69O7PNa	[M+Na]+
656.3682871	656.3687	0.0004	0.6094	PE O-30:8	C35H56NO7PNa	[M+Na]+
656.3682871	656.3674	0.0009	1.3712	SQDG 21:1	C30H54O12S	[M+NH4]+
656.3682871	656.3688	0.0005	0.7618	CerP 32:3;O4	C32H60NO8PK	[M+K]+
656.3682871	656.3688	0.0005	0.7618	LPC 24:3;O	C32H60NO8PK	[M+K]+
656.3682871	656.3688	0.0005	0.7618	LPE 27:3;O	C32H60NO8PK	[M+K]+
656.3682871	656.3688	0.0005	0.7618	LPS O-26:3	C32H60NO8PK	[M+K]+
656.3682871	656.3688	0.0005	0.7618	PC 24:2	C32H60NO8PK	[M+K]+
656.3682871	656.3688	0.0005	0.7618	PC O-24:3;O	C32H60NO8PK	[M+K]+
	656.3688	0.0005	0.7618	PE 27:2	C32H60NO8PK	[M+K]+

656.3682871						
656.3682871	656.36880.0005		0.7618	PE O-27:3;O	C32H60NO8PK	[M+K]+
	656.4708	0.0000	0.0000	HexCer 28:0;O3	C34H67NO9Na	[M+Na]+
656.4707940						
657.2968404	657.29530.0015		2.2821	PA 31:8	C34H51O8PK	[M+K]+
	657.3163		0.4564	PA 31:8;O	C34H51O9PNa	[M+Na]+
657.3159645						
657.3159645	657.3164	0.0005	0.7607	BMP 25:3	C31H55O10PK	[M+K]+
	657.3164	0.0005	0.7607	LPG 25:4;O	C31H55O10PK	[M+K]+
657.3159645						
657.3159645	657.3164	0.0005	0.7607	PG 25:3	C31H55O10PK	[M+K]+
	657.3164	0.0005	0.7607	PG O-25:4;O	C31H55O10PK	[M+K]+
657.3159645						
657.3881396	657.3874	0.0007	1.0648	EPC 31:6;O5	C33H57N2O9P	[M+H]+
	657.3874	0.0007	1.0648	SM 28:6;O5	C33H57N2O9P	[M+H]+
657.3881396						
657.3881396	657.3874	0.0007	1.0648	EPC 31:5;O6	C33H59N2O10P	[M+H-H2O]+
	657.3874	0.0007	1.0648	SM 28:5;O6	C33H59N2O10P	[M+H-H2O]+
657.3881396						
657.3881396	657.3891	0.0009	1.3691	LPA 33:7	C36H59O7PNa	[M+Na]+
	657.3891	0.0009	1.3691	PA O-33:7	C36H59O7PNa	[M+Na]+
657.3881396						
657.3881396	657.3874	0.0007	1.0648	LPS 27:6	C33H54NO9P	[M+NH4]+
	657.3874	0.0007	1.0648	LPS O-27:7;O	C33H54NO9P	[M+NH4]+
657.3881396						
657.3881396	657.3874	0.0007	1.0648	PC 25:6;O	C33H54NO9P	[M+NH4]+
	657.3874	0.0007	1.0648	PE 28:6;O	C33H54NO9P	[M+NH4]+
657.3881396						
657.3881396	657.3874	0.0007	1.0648	PS O-27:6	C33H54NO9P	[M+NH4]+
	657.3892	0.0011	1.6733	LPA 30:2;O	C33H63O8PK	[M+K]+
657.3881396						
657.3881396	657.3892	0.0011	1.6733	LPG O-27:3	C33H63O8PK	[M+K]+
	657.3892	0.0011	1.6733	PA 30:1	C33H63O8PK	[M+K]+
657.3881396						
657.3881396	657.3892	0.0011	1.6733	PA O-30:2;O	C33H63O8PK	[M+K]+
	657.3915	0.0012	1.8254	PA O-35:10	C38H57O7P	[M+H]+
657.3926386						
657.3926386	657.3915	0.0012	1.8254	PA 35:8	C38H59O8P	[M+H-H2O]+

	657.3915	0.0012	1.8254	PA O-35:9;O	C38H59O8P	[M+H-H2O]+
657.3926386						
657.3926386	657.3916	0.0011	1.6733	DG 37:10	C40H58O5K	[M+K]+
657.3926386	657.3916	0.0011	1.6733	TG O-37:10	C40H58O5K	[M+K]+
	658.3198	0.0025	3.7975	ST 25:4;O6;HexNAc	C33H49NO11Na	[M+Na]+
658.3173241						
661.3260822	661.3252	0.0009	1.3609	SQDG 23:4	C32H52O12S	[M+H]+
661.3260822	661.3266	0.0005	0.7561	LPA 31:7;O	C34H55O8PK	[M+K]+
	661.3266	0.0005	0.7561	PA 31:6	C34H55O8PK	[M+K]+
661.3260822						
661.3260822	661.3266	0.0005	0.7561	PA O-31:7;O	C34H55O8PK	[M+K]+
	661.5167	0.0000	0.0000	LPA 34:1	C37H73O7P	[M+H]+
661.5166485						
661.5166485	661.5167	0.0000	0.0000	LPA O-34:2;O	C37H73O7P	[M+H]+
	661.5167	0.0000	0.0000	PA O-34:1	C37H73O7P	[M+H]+
661.5166485						
661.5166485	661.5167	0.0000	0.0000	LPA 34:0;O	C37H75O8P	[M+H-H2O]+
	661.5167	0.0000	0.0000	LPG O-31:1	C37H75O8P	[M+H-H2O]+
661.5166485						
661.5166485	661.5167	0.0000	0.0000	PA O-34:0;O	C37H75O8P	[M+H-H2O]+
661.5166485	661.5166	0.0000	0.0000	CE 15:2;O2	C42H70O4Na	[M+Na]+
	661.5166	0.0000		DG O-39:7	C42H70O4Na	[M+Na]+
661.5166485						
661.5166485	661.51680.0001		0.1512	DG 36:1	C39H74O5K	[M+K]+
	661.5168	0.0001	0.1512	DG O-36:2;O	C39H74O5K	[M+K]+
661.5166485						
661.5166485	661.51680.0001		0.1512	TG O-36:1	C39H74O5K	[M+K]+
	662.5202	0.0004	0.6038	HexCer 30:0;O3	C36H71NO9	[M+H]+
662.5206024						
662.5206024	662.52020.0004		0.6038	TG 33:0;O3	C36H68O9	[M+NH4]+
	664.5276	0.0001	0.1505	CerP 36:0;O3	C36H74NO7P	[M+H]+
664.5274557						
664.5274557	664.52760.0001		0.1505	LPC 28:0	C36H74NO7P	[M+H]+
	664.5276	0.0001	0.1505	LPC O-28:1;O	C36H74NO7P	[M+H]+
664.5274557						
664.5274557	664.52760.0001		0.1505	LPE 31:0	C36H74NO7P	[M+H]+
	664.5276	0.0001	0.1505	LPE O-31:1;O	C36H74NO7P	[M+H]+

664.5274557						
664.5274557	664.52760.0001		0.1505	PC O-28:0	C36H74NO7P	[M+H]+
	664.5276	0.0001	0.1505	PE O-31:0	C36H74NO7P	[M+H]+
664.5274557						
664.5274557	664.52750.0001		0.1505	CAR 34:5	C41H71NO4Na	[M+Na]+
	664.5275	0.0001	0.1505	Cer 41:6;O3	C41H71NO4Na	[M+Na]+
664.5274557						
664.5274557	664.52760.0001		0.1505	LPA 33:1	C36H71O7P	[M+NH4]+
	664.5276	0.0001	0.1505	LPA O-33:2;O	C36H71O7P	[M+NH4]+
664.5274557						
664.5274557	664.52760.0001		0.1505	PA O-33:1	C36H71O7P	[M+NH4]+
	664.5277	0.0002	0.3010	CAR 31:0;O	C38H75NO5K	[M+K]+
664.5274557						
664.5274557	664.52770.0002		0.3010	Cer 38:1;O4	C38H75NO5K	[M+K]+
	670.2753	0.0026	3.8790	PS 24:4;O	C30H50NO11PK	[M+K]+
670.2726649						
670.3126695	670.31170.0010		1.4918	CerP 31:5;O6	C31H54NO10PK	[M+K]+
	670.3117	0.0010	1.4918	LPS 25:4;O	C31H54NO10PK	[M+K]+
670.3126695						
670.3126695	670.31170.0010		1.4918	PS 25:3	C31H54NO10PK	[M+K]+
	670.3117	0.0010	1.4918	PS O-25:4;O	C31H54NO10PK	[M+K]+
670.3126695						
670.4244725	670.42310.0014		2.0882	PE O-34:9	C39H62NO7P	[M+H-H2O]+
	671.4395	0.0010	1.4893	EPC 33:5;O4	C35H63N2O8P	[M+H]+
671.4385133						
671.4385133	671.43950.0010		1.4893	SM 30:5;O4	C35H63N2O8P	[M+H]+
	671.4395	0.0010	1.4893	EPC 33:4;O5	C35H65N2O9P	[M+H-H2O]+
671.4385133						
671.4385133	671.43950.0010		1.4893	SM 30:4;O5	C35H65N2O9P	[M+H-H2O]+
	671.4371	0.0014	2.0851	EPC 31:2;O4	C33H65N2O8PNa	[M+Na]+
671.4385133						
671.4385133	671.43710.0014		2.0851	SM 28:2;O4	C33H65N2O8PNa	[M+Na]+
	671.4395	0.0010	1.4893	CerP 35:6;O4	C35H60NO8P	[M+NH4]+
671.4385133						
671.4385133	671.43950.0010		1.4893	LPC 27:6;O	C35H60NO8P	[M+NH4]+
	671.4395	0.0010	1.4893	LPE 30:6;O	C35H60NO8P	[M+NH4]+
671.4385133						
671.4385133	671.43950.0010		1.4893	LPS O-29:6	C35H60NO8P	[M+NH4]+
	671.4395	0.0010	1.4893	PC 27:5	C35H60NO8P	[M+NH4]+

671.4385133						
671.4385133	671.43950.0010		1.4893	PC O-27:6;O	C35H60NO8P	[M+NH4]+
	671.4395		1.4893	PE 30:5	C35H60NO8P	[M+NH4]+
671.4385133						
671.4385133	671.4395	0.0010	1.4893	PE O-30:6;O	C35H60NO8P	[M+NH4]+
	671.4435	0.0013	1.9361	PA O-37:8	C40H65O7P	[M+H-H2O]+
671.4421696						
671.4421696	671.4412	0.0009	1.3404	LPA 32:1	C35H69O7PK	[M+K]+
	671.4412	0.0009	1.3404	LPA O-32:2;O	C35H69O7PK	[M+K]+
671.4421696						
671.4421696	671.4412	0.0009	1.3404	PA O-32:1	C35H69O7PK	[M+K]+
	671.4436	0.0014	2.0851	DG O-39:10	C42H64O4K	[M+K]+
671.4421696						
672.4442758	672.4446	0.0003	0.4461	CAR 30:5;O4	C37H63NO8Na	[M+Na]+
672.4442758	672.4446	0.0003	0.4461	HexCer 31:5;O2	C37H63NO8Na	[M+Na]+
	672.4446	0.0003	0.4461	ST 29:1;O3;HexNAc	C37H63NO8Na	[M+Na]+
672.4442758						
672.4442758	672.4446	0.0003	0.4461	BMP 26:0;O	C32H63O11P	[M+NH4]+
672.4442758	672.4446	0.0003	0.4461	LPI O-23:1	C32H63O11P	[M+NH4]+
	672.4446	0.0003	0.4461	PG 26:0;O	C32H63O11P	[M+NH4]+
672.4442758						
672.4442758	672.4447	0.0005	0.7436	HexCer 28:0;O3	C34H67NO9K	[M+K]+
	673.2959	0.0025	3.7131	LPI 21:4;O	C30H51O13PNa	[M+Na]+
673.2934699						
673.2934699	673.2959	0.0025	3.7131	PI 21:3	C30H51O13PNa	[M+Na]+
	673.2959	0.0025	3.7131	PI O-21:4;O	C30H51O13PNa	[M+Na]+
673.2934699						
675.3071351	675.3059	0.0013	1.9251	PA 31:7;O	C34H53O9PK	[M+K]+
	675.3059	0.0013	1.9251	PG O-28:8	C34H53O9PK	[M+K]+
675.3071351						
678.5066671	678.5068	0.0002	0.2948	CerP 36:1;O4	C36H72NO8P	[M+H]+
	678.5068	0.0002	0.2948	LPC 28:1;O	C36H72NO8P	[M+H]+
678.5066671						
678.5066671	678.5068	0.0002	0.2948	LPE 31:1;O	C36H72NO8P	[M+H]+
	678.5068	0.0002	0.2948	LPS O-30:1	C36H72NO8P	[M+H]+
678.5066671						
678.5066671	678.5068	0.0002	0.2948	PC 28:0	C36H72NO8P	[M+H]+

678.5066671	678.5068	0.0002	0.2948	PC O-28:1;O	C36H72NO8P	[M+H]+
678.5066671	678.5068	0.0002	0.2948	PE 31:0	C36H72NO8P	[M+H]+
678.5066671	678.5068	0.0002	0.2948	PE O-31:1;O	C36H72NO8P	[M+H]+
678.5066671	678.5068	0.0002	0.2948	CerP 36:0;O5	C36H74NO9P	[M+H-H2O]+
678.5066671	678.5068	0.0002	0.2948	LPS O-30:0;O	C36H74NO9P	[M+H-H2O]+
678.5066671	678.5068	0.0001	0.1474	CAR 34:6;O	C41H69NO5Na	[M+Na]+
678.5066671	678.5068	0.0002	0.2948	LPA 33:2;O	C36H69O8P	[M+NH4]+
678.5066671	678.5068	0.0002	0.2948	LPG O-30:3	C36H69O8P	[M+NH4]+
678.5066671	678.5068	0.0002	0.2948	PA 33:1	C36H69O8P	[M+NH4]+
678.5066671	678.5068	0.0002	0.2948	PA O-33:2;O	C36H69O8P	[M+NH4]+
678.5066671	678.5069	0.0003	0.4421	CAR 31:1;O2	C38H73NO6K	[M+K]+
678.5066671	678.5069	0.0003	0.4421	Cer 38:2;O5	C38H73NO6K	[M+K]+
679.5095284	679.5103	0.0008	1.1773	HexCer 29:1;O4	C35H67NO10	[M+NH4]+
679.5104966	679.5103		0.2943	HexCer 29:1;O4	C35H67NO10	[M+NH4]+
683.4047724	683.4047	0.0001	0.1463	PA O-35:8	C38H61O7PNa	[M+Na]+
683.4047724	683.4049	0.0001	0.1463	LPA 32:3;O	C35H65O8PK	[M+K]+
683.4047724	683.4049	0.0001	0.1463	LPG O-29:4	C35H65O8PK	[M+K]+
683.4047724	683.4049	0.0001	0.1463	PA 32:2	C35H65O8PK	[M+K]+
683.4047724	683.4049	0.0001	0.1463	PA O-32:3;O	C35H65O8PK	[M+K]+
684.3984808	684.3987	0.0002	0.2922	SQDG 23:1	C32H58O12S	[M+NH4]+
684.4564910	684.4559	0.0006	0.8766	EPC 30:2;O6	C32H63N2O10P	[M+NH4]+
685.4192724	685.4187	0.0005	0.7295	EPC 33:6;O5	C35H61N2O9P	[M+H]+
685.4192724	685.4187	0.0005	0.7295	SM 30:6;O5	C35H61N2O9P	[M+H]+
685.4192724	685.4187	0.0005	0.7295	EPC 33:5;O6	C35H63N2O10P	[M+H-H2O]+
685.4192724	685.4187	0.0005	0.7295	SM 30:5;O6	C35H63N2O10P	[M+H-H2O]+

685.4192724	685.4204	0.0011	1.6049	PA O-35:7	C38H63O7PNa	[M+Na]+
685.4192724	685.4187	0.0005	0.7295	LPS 29:6	C35H58NO9P	[M+NH4]+
685.4192724	685.4187	0.0005	0.7295	LPS O-29:7;O	C35H58NO9P	[M+NH4]+
685.4192724	685.4187	0.0005	0.7295	PC 27:6;O	C35H58NO9P	[M+NH4]+
685.4192724	685.4187	0.0005	0.7295	PE 30:6;O	C35H58NO9P	[M+NH4]+
685.4192724	685.4187	0.0005	0.7295	PS O-29:6	C35H58NO9P	[M+NH4]+
685.4208259	685.4204	0.0005	0.7295	PA O-35:7	C38H63O7PNa	[M+Na]+
685.4208259	685.4205	0.0003	0.4377	LPA 32:2;O	C35H67O8PK	[M+K]+
685.4208259	685.4205	0.0003	0.4377	LPG O-29:3	C35H67O8PK	[M+K]+
685.4208259	685.4205	0.0003	0.4377	PA 32:1	C35H67O8PK	[M+K]+
685.4208259	685.4205	0.0003	0.4377	PA O-32:2;O	C35H67O8PK	[M+K]+
685.4584055	685.4591	0.0007	1.0212	PA O-38:8	C41H67O7P	[M+H-H2O]+
685.4584055	685.4593	0.0009	1.3130	DG O-40:10	C43H66O4K	[M+K]+
685.4613222	685.4591	0.0022	3.2095	PA O-38:8	C41H67O7P	[M+H-H2O]+
685.4613222	685.4634	0.0020	2.9177	HexCer 30:5;O4	C36H61NO10	[M+NH4]+
685.4613222	685.4634	0.0020	2.9177	ST 28:1;O5;HexNAc	C36H61NO10	[M+NH4]+
685.4613222	685.4593	0.0021	3.0636	DG O-40:10	C43H66O4K	[M+K]+
686.3825567	686.3816	0.0009	1.3112	PE 33:9;O	C38H58NO9P	[M+H-H2O]+
686.3825567	686.3816	0.0009	1.3112	PS O-32:9	C38H58NO9P	[M+H-H2O]+
686.4155285	686.4156	0.0001	0.1457	LPC 29:7	C37H62NO7PNa	[M+Na]+
686.4155285	686.4156	0.0001	0.1457	LPE 32:7	C37H62NO7PNa	[M+Na]+
686.4155285	686.4156	0.0001	0.1457	PC O-29:7	C37H62NO7PNa	[M+Na]+
686.4155285	686.4156	0.0001	0.1457	PE O-32:7	C37H62NO7PNa	[M+Na]+
686.4155285	686.4158	0.0002	0.2914	CerP 34:2;O4	C34H66NO8PK	[M+K]+
686.4155285	686.4158	0.0002	0.2914	LPC 26:2;O	C34H66NO8PK	[M+K]+
	686.4158		0.2914	LPE 29:2;O	C34H66NO8PK	[M+K]+

686.4155285						
686.4155285	686.4158	0.0002	0.2914	LPS O-28:2	C34H66NO8PK	[M+K]+
	686.4158	0.0002	0.2914	PC 26:1	C34H66NO8PK	[M+K]+
686.4155285						
686.4155285	686.4158	0.0002	0.2914	PC O-26:2;O	C34H66NO8PK	[M+K]+
	686.4158	0.0002	0.2914	PE 29:1	C34H66NO8PK	[M+K]+
686.4155285						
686.4155285	686.4158	0.0002	0.2914	PE O-29:2;O	C34H66NO8PK	[M+K]+
686.4237502	686.4238	0.0001	0.1457	HexCer 31:6;O3	C37H61NO9Na	[M+Na]+
	686.4238	0.0001	0.1457	ST 29:2;O4;HexNAc	C37H61NO9Na	[M+Na]+
686.4237502						
686.4237502	686.4239	0.0001	0.1457	LPI 23:1	C32H61O12P	[M+NH4]+
	686.4239	0.0001	0.1457	LPI O-23:2;O	C32H61O12P	[M+NH4]+
686.4237502						
686.4237502	686.4239	0.0001	0.1457	PI O-23:1	C32H61O12P	[M+NH4]+
686.4237502	686.424	0.0003	0.4370	HexCer 28:1;O4	C34H65NO10K	[M+K]+
	686.5095	0.0004	0.5827	CerP 36:0;O3	C36H74NO7PNa	[M+Na]+
686.5090817						
686.5090817	686.5095	0.0004	0.5827	LPC 28:0	C36H74NO7PNa	[M+Na]+
	686.5095	0.0004	0.5827	LPC O-28:1;O	C36H74NO7PNa	[M+Na]+
686.5090817						
686.5090817	686.5095	0.0004	0.5827	LPE 31:0	C36H74NO7PNa	[M+Na]+
	686.5095	0.0004	0.5827	LPE O-31:1;O	C36H74NO7PNa	[M+Na]+
686.5090817						
686.5090817	686.5095	0.0004	0.5827	PC O-28:0	C36H74NO7PNa	[M+Na]+
	686.5095	0.0004	0.5827	PE O-31:0	C36H74NO7PNa	[M+Na]+
686.5090817						
686.5106124	686.5119	0.0013	1.8936	CerP 38:3;O3	C38H72NO7P	[M+H]+
	686.5119	0.0013	1.8936	LPC 30:3	C38H72NO7P	[M+H]+
686.5106124						
686.5106124	686.5119	0.0013	1.8936	LPC O-30:4;O	C38H72NO7P	[M+H]+
	686.5119	0.0013	1.8936	LPE 33:3	C38H72NO7P	[M+H]+
686.5106124						
686.5106124	686.5119	0.0013	1.8936	LPE O-33:4;O	C38H72NO7P	[M+H]+
	686.5119	0.0013	1.8936	PC O-30:3	C38H72NO7P	[M+H]+
686.5106124						
686.5106124	686.5119	0.0013	1.8936	PE O-33:3	C38H72NO7P	[M+H]+
	686.5119	0.0013	1.8936	CerP 38:2;O4	C38H74NO8P	[M+H-H2O]+

686.5106124						
686.5106124	686.5119	0.0013	1.8936	LPC 30:2;O	C38H74NO8P	[M+H-H2O]+
	686.5119	0.0013	1.8936	LPE 33:2;O	C38H74NO8P	[M+H-H2O]+
686.5106124						
686.5106124	686.5119	0.0013	1.8936	LPS O-32:2	C38H74NO8P	[M+H-H2O]+
	686.5119	0.0013	1.8936	PC 30:1	C38H74NO8P	[M+H-H2O]+
686.5106124						
686.5106124	686.5119	0.0013	1.8936	PC O-30:2;O	C38H74NO8P	[M+H-H2O]+
	686.5119	0.0013	1.8936	PE 33:1	C38H74NO8P	[M+H-H2O]+
686.5106124						
686.5106124	686.5119	0.0013	1.8936	PE O-33:2;O	C38H74NO8P	[M+H-H2O]+
	686.5095	0.0011	1.6023	CerP 36:0;O3	C36H74NO7PNa	[M+Na]+
686.5106124						
686.5106124	686.5095	0.0011	1.6023	LPC 28:0	C36H74NO7PNa	[M+Na]+
686.5106124	686.5095	0.0011	1.6023	LPC O-28:1;O	C36H74NO7PNa	[M+Na]+
	686.5095		1.6023	LPE 31:0	C36H74NO7PNa	[M+Na]+
686.5106124						
686.5106124	686.50950.0011		1.6023	LPE O-31:1;O	C36H74NO7PNa	[M+Na]+
	686.5095	0.0011	1.6023	PC O-28:0	C36H74NO7PNa	[M+Na]+
686.5106124						
686.5106124	686.50950.0011		1.6023	PE O-31:0	C36H74NO7PNa	[M+Na]+
	686.5119	0.0013	1.8936	PA O-35:4	C38H69O7P	[M+NH4]+
686.5106124						
686.5106124	686.5120.0014		2.0393	CAR 33:3;O	C40H73NO5K	[M+K]+
	686.512	0.0014	2.0393	Cer 40:4;O4	C40H73NO5K	[M+K]+
686.5106124						
687.2154575	687.21790.0024		3.4923	PI 20:5;O	C29H45O14PK	[M+K]+
	688.3375	0.0002	0.2906	PC 27:7	C35H56NO8PK	[M+K]+
688.3377238						
688.3377238	688.33750.0002		0.2906	PE 30:7	C35H56NO8PK	[M+K]+
	688.3375	0.0002	0.2906	PE O-30:8;O	C35H56NO8PK	[M+K]+
688.3377238						
693.5622003	693.56240.0002		0.2884	HexCer 31:0;O3	C37H73NO9	[M+NH4]+
	695.4435	0.0000	0.0000	PA O-39:10	C42H65O7P	[M+H-H2O]+
695.4435459						
698.4158003	698.41560.0002		0.2864	PC O-30:8	C38H62NO7PNa	[M+Na]+
	698.4156	0.0002	0.2864	PE O-33:8	C38H62NO7PNa	[M+Na]+
698.4158003						
698.4158003	698.41580.0000		0.0000	CerP 35:3;O4	C35H66NO8PK	[M+K]+

698.4158003	698.4158	0.0000	0.0000	LPC 27:3;O	C35H66NO8PK	[M+K]+
698.4158003	698.4158	0.0000	0.0000	LPE 30:3;O	C35H66NO8PK	[M+K]+
698.4158003	698.4158	0.0000	0.0000	LPS O-29:3	C35H66NO8PK	[M+K]+
698.4158003	698.4158	0.0000	0.0000	PC 27:2	C35H66NO8PK	[M+K]+
698.4158003	698.4158	0.0000	0.0000	PC O-27:3;O	C35H66NO8PK	[M+K]+
698.4158003	698.4158	0.0000	0.0000	PE 30:2	C35H66NO8PK	[M+K]+
698.4158003	698.4158	0.0000	0.0000	PE O-30:3;O	C35H66NO8PK	[M+K]+
698.5564016	698.5565	0.0001	0.1432	CAR 33:2;O4	C40H75NO8	[M+H]+
698.5564016	698.5565	0.0001	0.1432	HexCer 34:2;O2	C40H75NO8	[M+H]+
698.5564016	698.5565	0.0001	0.1432	HexCer 34:1;O3	C40H77NO9	[M+H-H2O]+
698.5564016	698.5565	0.0001	0.1432	TG 37:2;O2	C40H72O8	[M+NH4]+
698.5564016	698.5565	0.0001	0.1432	TG O-37:3;O3	C40H72O8	[M+NH4]+
699.4549013	699.4555	0.0006	0.8578	PS 27:0;O	C33H64NO11P	[M+NH4]+
699.4638917	699.4619	0.0020	2.8593	TG 42:12	C45H62O6	[M+H]+
699.4638917	699.4619	0.0020	2.8593	DG 42:12;O2	C45H64O7	[M+H-H2O]+
699.4638917	699.4619	0.0020	2.8593	TG 42:11;O	C45H64O7	[M+H-H2O]+
699.4638917	699.4619	0.0020	2.8593	TG O-42:12;O2	C45H64O7	[M+H-H2O]+
700.4333991	700.4337	0.0003	0.4283	PE O-35:10	C40H62NO7P	[M+H]+
700.4333991	700.4337	0.0003	0.4283	PC 32:8	C40H64NO8P	[M+H-H2O]+
700.4333991	700.4337	0.0003	0.4283	PC O-32:9;O	C40H64NO8P	[M+H-H2O]+
700.4333991	700.4337	0.0003	0.4283	PE 35:8	C40H64NO8P	[M+H-H2O]+
700.4333991	700.4337	0.0003	0.4283	PE O-35:9;O	C40H64NO8P	[M+H-H2O]+
700.4762274	700.4759		0.4283	CAR 32:5;O4	C39H67NO8Na	[M+Na]+
700.4762274	700.4759	0.0003	0.4283	HexCer 33:5;O2	C39H67NO8Na	[M+Na]+
700.4762274	700.4759	0.0003	0.4283	BMP 28:0;O	C34H67O11P	[M+NH4]+
700.4762274	700.4759	0.0003	0.4283	LPI O-25:1	C34H67O11P	[M+NH4]+

700.4762274	700.4759	0.0003	0.4283	PG 28:0;O	C34H67O11P	[M+NH4]+
700.4762274	700.4760.0002		0.2855	HexCer 30:0;O3	C36H71NO9K	[M+K]+
700.4886686	700.4888	0.0001	0.1428	CerP 36:1;O4	C36H72NO8PNa	[M+Na]+
700.4886686	700.48880.0001		0.1428	LPC 28:1;O	C36H72NO8PNa	[M+Na]+
700.4886686	700.4888	0.0001	0.1428	LPE 31:1;O	C36H72NO8PNa	[M+Na]+
700.4886686	700.48880.0001		0.1428	LPS O-30:1	C36H72NO8PNa	[M+Na]+
700.4886686	700.4888	0.0001	0.1428	PC 28:0	C36H72NO8PNa	[M+Na]+
700.4886686	700.48880.0001		0.1428	PC O-28:1;O	C36H72NO8PNa	[M+Na]+
700.4886686	700.4888	0.0001	0.1428	PE 31:0	C36H72NO8PNa	[M+Na]+
700.4886686	700.48880.0001		0.1428	PE O-31:1;O	C36H72NO8PNa	[M+Na]+
700.4906152	700.4912	0.0006	0.8565	CerP 38:4;O4	C38H70NO8P	[M+H]+
700.4906152	700.49120.0006		0.8565	LPC 30:4;O	C38H70NO8P	[M+H]+
700.4906152	700.4912	0.0006	0.8565	LPE 33:4;O	C38H70NO8P	[M+H]+
700.4906152	700.49120.0006		0.8565	LPS O-32:4	C38H70NO8P	[M+H]+
700.4906152	700.4912	0.0006	0.8565	PC 30:3	C38H70NO8P	[M+H]+
700.4906152	700.49120.0006		0.8565	PC O-30:4;O	C38H70NO8P	[M+H]+
700.4906152	700.4912	0.0006	0.8565	PE 33:3	C38H70NO8P	[M+H]+
700.4906152	700.49120.0006		0.8565	PE O-33:4;O	C38H70NO8P	[M+H]+
700.4906152	700.4912	0.0006	0.8565	CerP 38:3;O5	C38H72NO9P	[M+H-H2O]+
700.4906152	700.49120.0006		0.8565	LPS 32:2	C38H72NO9P	[M+H-H2O]+
700.4906152	700.4912	0.0006	0.8565	LPS O-32:3;O	C38H72NO9P	[M+H-H2O]+
700.4906152	700.49120.0006		0.8565	PC 30:2;O	C38H72NO9P	[M+H-H2O]+
700.4906152	700.4912	0.0006	0.8565	PE 33:2;O	C38H72NO9P	[M+H-H2O]+
700.4906152	700.49120.0006		0.8565	PS O-32:2	C38H72NO9P	[M+H-H2O]+
700.4906152	700.4912	0.0006	0.8565	LPG O-32:6	C38H67O8P	[M+NH4]+
700.4906152	700.49120.0006		0.8565	PA 35:4	C38H67O8P	[M+NH4]+
	700.4912	0.0006	0.8565	PA O-35:5;O	C38H67O8P	[M+NH4]+

700.4906152						
700.4906152	700.4913	0.0007	0.9993	CAR 33:4;O2	C40H71NO6K	[M+K]+
	700.4913	0.0007	0.9993	Cer 40:5;O5	C40H71NO6K	[M+K]+
700.4906152						
701.4340989	701.4364	0.0023	3.2790	BMP 29:1	C35H67O10PNa	[M+Na]+
	701.4364	0.0023	3.2790	LPG 29:2;O	C35H67O10PNa	[M+Na]+
701.4340989						
701.4340989	701.4364	0.0023	3.2790	PG 29:1	C35H67O10PNa	[M+Na]+
	701.4364	0.0023	3.2790	PG O-29:2;O	C35H67O10PNa	[M+Na]+
701.4340989						
701.4921607	701.4904	0.0017	2.4234	PA O-39:7	C42H71O7P	[M+H-H2O]+
	701.4906		2.2809	CE 17:4;O2	C44H70O4K	[M+K]+
701.4921607						
701.4921607	701.4906	0.0016	2.2809	DG O-41:9	C44H70O4K	[M+K]+
	707.4035	0.0010	1.4136	SQDG 26:2	C35H62O12S	[M+H]+
707.4024421						
707.4024421	707.4031	0.0006	0.8482	PE 32:9;O	C37H56NO9P	[M+NH4]+
	707.5374	0.0001	0.1413	PA O-39:4	C42H77O7P	[M+H-H2O]+
707.5375351						
707.5375351	707.5375	0.0000	0.0000	CE 17:1;O2	C44H76O4K	[M+K]+
	707.5375	0.0000	0.0000	DG O-41:6	C44H76O4K	[M+K]+
707.5375351						
708.4054239	708.4082	0.0028	3.9525	IPC 28:3;O3	C34H62NO12P	[M+H]+
	708.4082	0.0028	3.9525	IPC 28:2;O4	C34H64NO13P	[M+H-H2O]+
708.4054239						
708.4054239	708.4082	0.0028	3.9525	LPI 25:4	C34H59O12P	[M+NH4]+
	708.4082	0.0028	3.9525	LPI O-25:5;O	C34H59O12P	[M+NH4]+
708.4054239						
708.4054239	708.4082	0.0028	3.9525	PI O-25:4	C34H59O12P	[M+NH4]+
708.4054239	708.4084	0.0029	4.0937	HexCer 30:4;O4	C36H63NO10K	[M+K]+
	708.4084	0.0029	4.0937	ST 28:0;O5;HexNAc	C36H63NO10K	[M+K]+
708.4054239						
709.3973833	709.3954	0.0020	2.8193	EPC 33:5;O4	C35H63N2O8PK	[M+K]+
	709.3954	0.0020	2.8193	SM 30:5;O4	C35H63N2O8PK	[M+K]+
709.3973833						
711.4360967	711.436	0.0001	0.1406	PA O-37:8	C40H65O7PNa	[M+Na]+
	711.4362	0.0001	0.1406	LPA 34:3;O	C37H69O8PK	[M+K]+

711.4360967						
711.4360967	711.4362	0.0001	0.1406	LPG O-31:4	C37H69O8PK	[M+K]+
711.4360967	711.4362	0.0001	0.1406	PA 34:2	C37H69O8PK	[M+K]+
	711.4362	0.0001	0.1406	PA O-34:3;O	C37H69O8PK	[M+K]+
711.4360967						
712.3573477	712.3585	0.0011	1.5442	PE 32:9;O	C37H56NO9PNa	[M+Na]+
	712.3586	0.0013	1.8249	CerP 34:5;O6	C34H60NO10PK	[M+K]+
712.3573477						
712.3573477	712.3586	0.0013	1.8249	LPS 28:4;O	C34H60NO10PK	[M+K]+
	712.3586	0.0013	1.8249	PS 28:3	C34H60NO10PK	[M+K]+
712.3573477						
712.3573477	712.3586	0.0013	1.8249	PS O-28:4;O	C34H60NO10PK	[M+K]+
	712.4395	0.0004	0.5615	IPC 28:1;O3	C34H66NO12P	[M+H]+
712.4391648						
712.4391648	712.4395	0.0004	0.5615	IPC 28:0;O4	C34H68NO13P	[M+H-H2O]+
	712.4395	0.0004	0.5615	LPI 25:2	C34H63O12P	[M+NH4]+
712.4391648						
712.4391648	712.4395	0.0004	0.5615	LPI O-25:3;O	C34H63O12P	[M+NH4]+
	712.4395	0.0004	0.5615	PI O-25:2	C34H63O12P	[M+NH4]+
712.4391648						
712.4391648	712.4397	0.0005	0.7018	HexCer 30:2;O4	C36H67NO10K	[M+K]+
	712.4395	0.0008	1.1229	IPC 28:1;O3	C34H66NO12P	[M+H]+
712.4403034						
712.4403034	712.4395	0.0008	1.1229	IPC 28:0;O4	C34H68NO13P	[M+H-H2O]+
	712.4395	0.0008	1.1229	LPI 25:2	C34H63O12P	[M+NH4]+
712.4403034						
712.4403034	712.4395	0.0008	1.1229	LPI O-25:3;O	C34H63O12P	[M+NH4]+
712.4403034	712.4395	0.0008	1.1229	PI O-25:2	C34H63O12P	[M+NH4]+
	712.4397		0.9825	HexCer 30:2;O4	C36H67NO10K	[M+K]+
712.4403034						
713.4340928	713.4364	0.0023	3.2238	BMP 30:2	C36H67O10PNa	[M+Na]+
	713.4364	0.0023	3.2238	LPG 30:3;O	C36H67O10PNa	[M+Na]+
713.4340928						
713.4340928	713.4364	0.0023	3.2238	PG 30:2	C36H67O10PNa	[M+Na]+
	713.4364	0.0023	3.2238	PG O-30:3;O	C36H67O10PNa	[M+Na]+
713.4340928						
713.4428686	713.4412	0.0017	2.3828	TG 42:12;O2	C45H62O8	[M+H-H2O]+
	713.4517	0.0002	0.2803	PA O-37:7	C40H67O7PNa	[M+Na]+

713.4518465						
713.4518465	713.4518	0.0000	0.0000	LPA 34:2;O	C37H71O8PK	[M+K]+
	713.4518	0.0000	0.0000	LPG O-31:3	C37H71O8PK	[M+K]+
713.4518465						
713.4518465	713.4518	0.0000	0.0000	PA 34:1	C37H71O8PK	[M+K]+
	713.4518	0.0000	0.0000	PA O-34:2;O	C37H71O8PK	[M+K]+
713.4518465						
714.4551929	714.4552	0.0000	0.0000	IPC 28:0;O3	C34H68NO12P	[M+H]+
	714.4551	0.0000	0.0000	HexCer 33:6;O3	C39H65NO9Na	[M+Na]+
714.4551929						
714.4551929	714.4552	0.0000	0.0000	LPI 25:1	C34H65O12P	[M+NH4]+
	714.4552	0.0000	0.0000	LPI O-25:2;O	C34H65O12P	[M+NH4]+
714.4551929						
714.4551929	714.4552	0.0000	0.0000	PI O-25:1	C34H65O12P	[M+NH4]+
	714.4553	0.0001	0.1400	HexCer 30:1;O4	C36H69NO10K	[M+K]+
714.4551929						
714.4834896	714.4833	0.0002	0.2799	CerP 40:6;O2	C40H70NO6PNa	[M+Na]+
	714.4833	0.0002	0.2799	LPC O-32:7	C40H70NO6PNa	[M+Na]+
714.4834896						
714.4834896	714.4834	0.0000	0.0000	CerP 37:1;O3	C37H74NO7PK	[M+K]+
	714.4834	0.0000	0.0000	LPC 29:1	C37H74NO7PK	[M+K]+
714.4834896						
714.4834896	714.4834	0.0000	0.0000	LPC O-29:2;O	C37H74NO7PK	[M+K]+
	714.4834	0.0000	0.0000	LPE 32:1	C37H74NO7PK	[M+K]+
714.4834896						
714.4834896	714.4834	0.0000	0.0000	LPE O-32:2;O	C37H74NO7PK	[M+K]+
	714.4834	0.0000	0.0000	PC O-29:1	C37H74NO7PK	[M+K]+
714.4834896						
714.4834896	714.4834	0.0000	0.0000	PE O-32:1	C37H74NO7PK	[M+K]+
	715.4868	0.0000	0.0000	IPC 28:0;O2	C34H68NO11P	[M+NH4]+
715.4868639						
715.4979111	715.4997	0.0018	2.5157	EPC 34:1;O4	C36H73N2O8PNa	[M+Na]+
715.4979111	715.4997	0.0018	2.5157	SM 31:1;O4	C36H73N2O8PNa	[M+Na]+
	716.3253	0.0021	2.9316	ST 27:5;O8;HexNAc	C35H51NO13Na	[M+Na]+
716.3232072						
716.4626026	716.4626	0.0000	0.0000	CerP 39:6;O3	C39H68NO7PNa	[M+Na]+
	716.4626	0.0000	0.0000	LPC 31:6	C39H68NO7PNa	[M+Na]+
716.4626026						
716.4626026	716.4626	0.0000	0.0000	LPC O-31:7;O	C39H68NO7PNa	[M+Na]+

716.4626026	716.4626	0.0000	0.0000	LPE 34:6	C39H68NO7PNa	[M+Na]+
716.4626026	716.4626	0.0000	0.0000	LPE O-34:7;O	C39H68NO7PNa	[M+Na]+
716.4626026	716.4626	0.0000	0.0000	PC O-31:6	C39H68NO7PNa	[M+Na]+
716.4626026	716.4626	0.0000	0.0000	PE O-34:6	C39H68NO7PNa	[M+Na]+
716.4626026	716.4627		0.1396	CerP 36:1;O4	C36H72NO8PK	[M+K]+
716.4626026	716.4627	0.0001	0.1396	LPC 28:1;O	C36H72NO8PK	[M+K]+
716.4626026	716.4627	0.0001	0.1396	LPE 31:1;O	C36H72NO8PK	[M+K]+
716.4626026	716.4627	0.0001	0.1396	LPS O-30:1	C36H72NO8PK	[M+K]+
716.4626026	716.4627	0.0001	0.1396	PC 28:0	C36H72NO8PK	[M+K]+
716.4626026	716.4627	0.0001	0.1396	PC O-28:1;O	C36H72NO8PK	[M+K]+
716.4626026	716.4627	0.0001	0.1396	PE 31:0	C36H72NO8PK	[M+K]+
716.4626026	716.4627	0.0001	0.1396	PE O-31:1;O	C36H72NO8PK	[M+K]+
716.4833072	716.4837	0.0004	0.5583	CerP 36:1;O5	C36H72NO9PNa	[M+Na]+
716.4833072	716.4837	0.0004	0.5583	LPS 30:0	C36H72NO9PNa	[M+Na]+
716.4833072	716.4837	0.0004	0.5583	LPS O-30:1;O	C36H72NO9PNa	[M+Na]+
716.4833072	716.4837	0.0004	0.5583	PC 28:0;O	C36H72NO9PNa	[M+Na]+
716.4833072	716.4837	0.0004	0.5583	PE 31:0;O	C36H72NO9PNa	[M+Na]+
716.4833072	716.4837	0.0004	0.5583	PS O-30:0	C36H72NO9PNa	[M+Na]+
718.5384286	718.5381	0.0003	0.4175	CerP 39:2;O4	C39H76NO8P	[M+H]+
718.5384286	718.5381	0.0003	0.4175	LPC 31:2;O	C39H76NO8P	[M+H]+
718.5384286	718.5381	0.0003	0.4175	LPE 34:2;O	C39H76NO8P	[M+H]+
718.5384286	718.5381	0.0003	0.4175	LPS O-33:2	C39H76NO8P	[M+H]+
718.5384286	718.5381	0.0003	0.4175	PC 31:1	C39H76NO8P	[M+H]+
718.5384286	718.5381	0.0003	0.4175	PC O-31:2;O	C39H76NO8P	[M+H]+
718.5384286	718.5381	0.0003	0.4175	PE 34:1	C39H76NO8P	[M+H]+
718.5384286	718.5381	0.0003	0.4175	PE O-34:2;O	C39H76NO8P	[M+H]+

718.5384286	718.5381	0.0003	0.4175	CerP 39:1;O5	C39H78NO9P	[M+H-H2O]+
718.5384286	718.5381	0.0003	0.4175	LPS 33:0	C39H78NO9P	[M+H-H2O]+
718.5384286	718.5381	0.0003	0.4175	LPS O-33:1;O	C39H78NO9P	[M+H-H2O]+
718.5384286	718.5381	0.0003	0.4175	PC 31:0;O	C39H78NO9P	[M+H-H2O]+
718.5384286	718.5381	0.0003	0.4175	PE 34:0;O	C39H78NO9P	[M+H-H2O]+
718.5384286	718.5381	0.0003	0.4175	PS O-33:0	C39H78NO9P	[M+H-H2O]+
718.5384286	718.5381	0.0003	0.4175	LPG O-33:4	C39H73O8P	[M+NH4]+
718.5384286	718.5381	0.0003	0.4175	PA 36:2	C39H73O8P	[M+NH4]+
718.5384286	718.5381	0.0003	0.4175	PA O-36:3;O	C39H73O8P	[M+NH4]+
718.5384286	718.5382	0.0002	0.2783	CAR 34:2;O2	C41H77NO6K	[M+K]+
718.5384286	718.5382	0.0002	0.2783	Cer 41:3;O5	C41H77NO6K	[M+K]+
719.4067130	719.4049	0.0019	2.6411	LPG O-32:7	C38H65O8PK	[M+K]+
719.4067130	719.4049	0.0019	2.6411	PA 35:5	C38H65O8PK	[M+K]+
719.4067130	719.4049	0.0019	2.6411	PA O-35:6;O	C38H65O8PK	[M+K]+
719.5416219	719.5416	0.0000	0.0000	HexCer 32:2;O4	C38H71NO10	[M+NH4]+
720.5431559	720.5409	0.0023	3.1920	ACer 42:5;O6	C42H73NO8	[M+H]+
720.5431559	720.5409		3.1920	HexCer 36:5;O2	C42H73NO8	[M+H]+
720.5431559	720.5409	0.0023	3.1920	HexCer 36:4;O3	C42H75NO9	[M+H-H2O]+
720.5431559	720.5409	0.0023	3.1920	TG 39:5;O2	C42H70O8	[M+NH4]+
720.5431559	720.5409	0.0023	3.1920	TG O-39:6;O3	C42H70O8	[M+NH4]+
721.3627023	721.3616	0.0011	1.5249	SQDG 29:7	C38H58O12S	[M+H-H2O]+
721.4552988	721.4555	0.0002	0.2772	SQDG 28:0	C37H70O12S	[M+H-H2O]+
721.4552988	721.4551	0.0002	0.2772	PC 31:8	C39H62NO8P	[M+NH4]+
721.4552988	721.4551	0.0002	0.2772	PE 34:8	C39H62NO8P	[M+NH4]+
721.4552988	721.4551	0.0002	0.2772	PE O-34:9;O	C39H62NO8P	[M+NH4]+
723.3769367	723.3773	0.0003	0.4147	SQDG 29:6	C38H60O12S	[M+H-H2O]+

726.3435273	726.3460.0025		3.4419	ST 29:6;O7;HexNAc	C37H53NO12Na	[M+Na]+
726.3435273	726.3461	0.0026	3.5796	ST 26:1;O8;HexNAc	C34H57NO13K	[M+K]+
726.4469514	726.44690.0000		0.0000	PC O-32:8	C40H66NO7PNa	[M+Na]+
726.4469514	726.4469	0.0000	0.0000	PE O-35:8	C40H66NO7PNa	[M+Na]+
726.4469514	726.44710.0001		0.1377	CerP 37:3;O4	C37H70NO8PK	[M+K]+
726.4469514	726.4471	0.0001	0.1377	LPC 29:3;O	C37H70NO8PK	[M+K]+
726.4469514	726.44710.0001		0.1377	LPE 32:3;O	C37H70NO8PK	[M+K]+
726.4469514	726.4471	0.0001	0.1377	LPS O-31:3	C37H70NO8PK	[M+K]+
726.4469514	726.44710.0001		0.1377	PC 29:2	C37H70NO8PK	[M+K]+
726.4469514	726.4471	0.0001	0.1377	PC O-29:3;O	C37H70NO8PK	[M+K]+
726.4469514	726.44710.0001		0.1377	PE 32:2	C37H70NO8PK	[M+K]+
726.4469514	726.4471	0.0001	0.1377	PE O-32:3;O	C37H70NO8PK	[M+K]+
727.4956366	727.49320.0024		3.2990	TG 44:12	C47H66O6	[M+H]+
727.4956366	727.4932	0.0024	3.2990	DG 44:12;O2	C47H68O7	[M+H-H2O]+
727.4956366	727.49320.0024		3.2990	TG 44:11;O	C47H68O7	[M+H-H2O]+
727.4956366	727.4932	0.0024	3.2990	TG O-44:12;O2	C47H68O7	[M+H-H2O]+
728.3350923	728.33240.0027		3.7071	PE 32:9;O	C37H56NO9PK	[M+K]+
730.5148764	730.5146	0.0003	0.4107	CerP 41:5;O2	C41H74NO6PNa	[M+Na]+
730.5148764	730.51460.0003		0.4107	LPC O-33:6	C41H74NO6PNa	[M+Na]+
730.5148764	730.5147	0.0001	0.1369	CerP 38:0;O3	C38H78NO7PK	[M+K]+
730.5148764	730.51470.0001		0.1369	LPC 30:0	C38H78NO7PK	[M+K]+
730.5148764	730.5147	0.0001	0.1369	LPC O-30:1;O	C38H78NO7PK	[M+K]+
730.5148764	730.51470.0001		0.1369	LPE 33:0	C38H78NO7PK	[M+K]+
730.5148764	730.5147	0.0001	0.1369	LPE O-33:1;O	C38H78NO7PK	[M+K]+
730.5148764	730.51470.0001		0.1369	PC O-30:0	C38H78NO7PK	[M+K]+
	730.5147	0.0001	0.1369	PE O-33:0	C38H78NO7PK	[M+K]+

730.5148764						
734.3428238	734.3430.0002		0.2724	LPS 30:7;O	C36H58NO10PK	[M+K]+
	734.343		0.2724	PS 30:6	C36H58NO10PK	[M+K]+
734.3428238						
734.3428238	734.3430.0002		0.2724	PS O-30:7;O	C36H58NO10PK	[M+K]+
	735.3773	0.0004	0.5439	SQDG 30:7	C39H60O12S	[M+H-H2O]+
735.3776947						
735.4144657	735.41610.0017		2.3116	DGDG 21:2	C36H62O15	[M+H]+
	735.6011	0.0018	2.4470	EPC 38:0;O3	C40H83N2O7P	[M+H]+
735.5992175						
735.5992175	735.60110.0018		2.4470	SM 35:0;O3	C40H83N2O7P	[M+H]+
	735.6011	0.0018	2.4470	CerP 40:1;O3	C40H80NO7P	[M+NH4]+
735.5992175						
735.5992175	735.60110.0018		2.4470	LPC 32:1	C40H80NO7P	[M+NH4]+
	735.6011	0.0018	2.4470	LPC O-32:2;O	C40H80NO7P	[M+NH4]+
735.5992175						
735.5992175	735.60110.0018		2.4470	PC O-32:1	C40H80NO7P	[M+NH4]+
	735.6011	0.0018	2.4470	PE O-35:1	C40H80NO7P	[M+NH4]+
735.5992175						
736.4370173	736.43710.0001		0.1358	IPC 28:0;O3	C34H68NO12PNa	[M+Na]+
	737.3943	0.0004	0.5425	PA O-38:10	C41H63O7PK	[M+K]+
737.3946766						
737.4274741	737.42670.0008		1.0849	EPC 35:5;O4	C37H67N2O8PK	[M+K]+
	737.4267	0.0008	1.0849	SM 32:5;O4	C37H67N2O8PK	[M+K]+
737.4274741						
737.4686068	737.47120.0026		3.5256	IPC 30:3;O2	C36H66NO11P	[M+NH4]+
	737.4712	0.0026	3.5256	PS 30:2;O	C36H66NO11P	[M+NH4]+
737.4686068						
737.4710486	737.47120.0001		0.1356	IPC 30:3;O2	C36H66NO11P	[M+NH4]+
	737.4712	0.0001	0.1356	PS 30:2;O	C36H66NO11P	[M+NH4]+
737.4710486						
738.3763699	738.37430.0021		2.8441	CerP 36:6;O6	C36H62NO10PK	[M+K]+
	738.3743	0.0021	2.8441	LPS 30:5;O	C36H62NO10PK	[M+K]+
738.3763699						
738.3763699	738.37430.0021		2.8441	PS 30:4	C36H62NO10PK	[M+K]+
	738.3743	0.0021	2.8441	PS O-30:5;O	C36H62NO10PK	[M+K]+
738.3763699						
739.5290130	739.52960.0006		0.8113	DG 46:13	C49H70O5	[M+H]+
	739.5296	0.0006	0.8113	TG O-46:13	C49H70O5	[M+H]+

739.5290130						
739.5290130	739.52960.0006		0.8113	DG 46:12;O	C49H72O6	[M+H-H2O]+
	739.5296	0.0006	0.8113	DG O-46:13;O2	C49H72O6	[M+H-H2O]+
739.5290130						
739.5290130	739.52960.0006		0.8113	TG 46:11	C49H72O6	[M+H-H2O]+
	739.5296	0.0006	0.8113	TG O-46:12;O	C49H72O6	[M+H-H2O]+
739.5290130						
739.5318488	739.53140.0004		0.5409	HexCer 31:1;O6	C37H71NO12	[M+NH4]+
	739.5507	0.0020	2.7043	DG 43:8;O2	C46H74O7	[M+H]+
739.5526827						
739.5526827	739.55070.0020		2.7043	TG 43:7;O	C46H74O7	[M+H]+
	739.5507	0.0020	2.7043	TG O-43:8;O2	C46H74O7	[M+H]+
739.5526827						
739.5526827	739.55070.0020		2.7043	TG 43:6;O2	C46H76O8	[M+H-H2O]+
	739.5507	0.0020	2.7043	TG O-43:7;O3	C46H76O8	[M+H-H2O]+
739.5526827						
740.3890230	740.38860.0005		0.6753	SHexCer 28:3;O5C34H61NO14S		[M+H]+
	740.3886	0.0005	0.6753	SHexCer 28:2;O6C34H63NO15S		[M+H-H2O]+
740.3890230						
740.3890230	740.38980.0008		1.0805	PE 34:9;O	C39H60NO9PNa	[M+Na]+
	740.3898		1.0805	PS O-33:9	C39H60NO9PNa	[M+Na]+
740.3890230						
740.3890230	740.38990.0009		1.2156	CerP 36:5;O6	C36H64NO10PK	[M+K]+
	740.3899	0.0009	1.2156	LPS 30:4;O	C36H64NO10PK	[M+K]+
740.3890230						
740.3890230	740.38990.0009		1.2156	PS 30:3	C36H64NO10PK	[M+K]+
	740.3899	0.0009	1.2156	PS O-30:4;O	C36H64NO10PK	[M+K]+
740.3890230						
740.3907608	740.39220.0014		1.8909	PS 35:10	C41H60NO10P	[M+H-H2O]+
	740.3898	0.0010	1.3506	PE 34:9;O	C39H60NO9PNa	[M+Na]+
740.3907608						
740.3907608	740.38980.0010		1.3506	PS O-33:9	C39H60NO9PNa	[M+Na]+
	740.3899	0.0008	1.0805	CerP 36:5;O6	C36H64NO10PK	[M+K]+
740.3907608						
740.3907608	740.38990.0008		1.0805	LPS 30:4;O	C36H64NO10PK	[M+K]+
	740.3899	0.0008	1.0805	PS 30:3	C36H64NO10PK	[M+K]+
740.3907608						
740.3907608	740.38990.0008		1.0805	PS O-30:4;O	C36H64NO10PK	[M+K]+
	741.4661	0.0005	0.6743	IPC 29:2;O3	C35H66NO12P	[M+NH4]+

741.4655472						
743.3447552	743.34370.0011		1.4798	SQDG 26:3	C35H60O12SK	[M+K]+
	743.5197	0.0014	1.8829	LPG 33:1	C39H77O9PNa	[M+Na]+
743.5183768						
743.5183768	743.51970.0014		1.8829	LPG O-33:2;O	C39H77O9PNa	[M+Na]+
	743.5197	0.0014	1.8829	PA 36:0;O	C39H77O9PNa	[M+Na]+
743.5183768						
743.5183768	743.51970.0014		1.8829	PG O-33:1	C39H77O9PNa	[M+Na]+
	743.5181	0.0003	0.4035	IPC 30:0;O2	C36H72NO11P	[M+NH4]+
743.5183768						
744.4940313	744.49390.0002		0.2686	CerP 41:6;O3	C41H72NO7PNa	[M+Na]+
	744.4939	0.0002	0.2686	LPC 33:6	C41H72NO7PNa	[M+Na]+
744.4940313						
744.4940313	744.49390.0002		0.2686	LPC O-33:7;O	C41H72NO7PNa	[M+Na]+
	744.4939	0.0002	0.2686	PC O-33:6	C41H72NO7PNa	[M+Na]+
744.4940313						
744.4940313	744.49390.0002		0.2686	PE O-36:6	C41H72NO7PNa	[M+Na]+
	744.494	0.0000	0.0000	CerP 38:1;O4	C38H76NO8PK	[M+K]+
744.4940313						
744.4940313	744.4940.0000		0.0000	LPC 30:1;O	C38H76NO8PK	[M+K]+
	744.494	0.0000	0.0000	LPE 33:1;O	C38H76NO8PK	[M+K]+
744.4940313						
744.4940313	744.4940.0000		0.0000	LPS O-32:1	C38H76NO8PK	[M+K]+
	744.494	0.0000	0.0000	PC 30:0	C38H76NO8PK	[M+K]+
744.4940313						
744.4940313	744.4940.0000		0.0000	PC O-30:1;O	C38H76NO8PK	[M+K]+
	744.494	0.0000	0.0000	PE 33:0	C38H76NO8PK	[M+K]+
744.4940313						
744.4940313	744.4940.0000		0.0000	PE O-33:1;O	C38H76NO8PK	[M+K]+
	744.515	0.0015	2.0147	CerP 38:1;O5	C38H76NO9PNa	[M+Na]+
744.5135148						
744.5135148	744.5150.0015		2.0147	LPS 32:0	C38H76NO9PNa	[M+Na]+
	744.515	0.0015	2.0147	LPS O-32:1;O	C38H76NO9PNa	[M+Na]+
744.5135148						
744.5135148	744.5150.0015		2.0147	PC 30:0;O	C38H76NO9PNa	[M+Na]+
	744.515	0.0015	2.0147	PE 33:0;O	C38H76NO9PNa	[M+Na]+
744.5135148						
744.5135148	744.5150.0015		2.0147	PS O-32:0	C38H76NO9PNa	[M+Na]+
	744.5256		1.0745	HexCer 34:3;O5	C40H73NO11	[M+H]+

744.5248634						
744.5248634	744.5256	0.0008	1.0745	HexCer 34:2;O6	C40H75NO12	[M+H-H2O]+
	744.5773	0.0015	2.0146	ACer 45:6;O5	C45H77NO7	[M+H]+
744.5788017						
744.5788017	744.5773	0.0015	2.0146	ACer 45:5;O6	C45H79NO8	[M+H-H2O]+
	744.5773	0.0015	2.0146	HexCer 39:5;O2	C45H79NO8	[M+H-H2O]+
744.5788017						
744.5788017	744.5773	0.0015	2.0146	DG 42:7;O2	C45H74O7	[M+NH4]+
	744.5773	0.0015	2.0146	TG 42:6;O	C45H74O7	[M+NH4]+
744.5788017						
744.5788017	744.5773	0.0015	2.0146	TG O-42:7;O2	C45H74O7	[M+NH4]+
	745.4191	0.0005	0.6708	SQDG 29:4	C38H64O12S	[M+H]+
745.4196316						
745.4196316	745.4205	0.0009	1.2074	PA 37:6	C40H67O8PK	[M+K]+
	745.4205	0.0009	1.2074	PA O-37:7;O	C40H67O8PK	[M+K]+
745.4196316						
745.4196316	745.4165	0.0031	4.1587	EPC 33:3;O6	C35H67N2O10PK	[M+K]+
	745.4165	0.0031	4.1587	SM 30:3;O6	C35H67N2O10PK	[M+K]+
745.4196316						
745.4205609	745.4205	0.0000	0.0000	PA 37:6	C40H67O8PK	[M+K]+
	745.4205	0.0000	0.0000	PA O-37:7;O	C40H67O8PK	[M+K]+
745.4205609						
746.4235543	746.4239	0.0003	0.4019	IPC 31:5;O3	C37H64NO12P	[M+H]+
	746.4239	0.0003	0.4019	IPC 31:4;O4	C37H66NO13P	[M+H-H2O]+
746.4235543						
746.4235543	746.4239	0.0003	0.4019	LPI 28:6	C37H61O12P	[M+NH4]+
	746.4239	0.0003	0.4019	LPI O-28:7;O	C37H61O12P	[M+NH4]+
746.4235543						
746.4235543	746.4239	0.0003	0.4019	PI O-28:6	C37H61O12P	[M+NH4]+
	746.424	0.0004	0.5359	HexCer 33:6;O4	C39H65NO10K	[M+K]+
746.4235543						
748.5484093	748.5487	0.0003	0.4008	CerP 40:2;O5	C40H78NO9P	[M+H]+
	748.5487	0.0003	0.4008	LPS 34:1	C40H78NO9P	[M+H]+
748.5484093						
748.5484093	748.5487	0.0003	0.4008	LPS O-34:2;O	C40H78NO9P	[M+H]+
	748.5487	0.0003	0.4008	PC 32:1;O	C40H78NO9P	[M+H]+
748.5484093						
748.5484093	748.5487	0.0003	0.4008	PE 35:1;O	C40H78NO9P	[M+H]+
	748.5487	0.0003	0.4008	PS O-34:1	C40H78NO9P	[M+H]+

748.5484093						
748.5484093	748.5487	0.0003	0.4008	CerP 40:1;O6	C40H80NO10P	[M+H-H2O]+
	748.5487	0.0003	0.4008	LPS 34:0;O	C40H80NO10P	[M+H-H2O]+
748.5484093						
748.5484093	748.5487	0.0003	0.4008	PS O-34:0;O	C40H80NO10P	[M+H-H2O]+
	748.5487	0.0003	0.4008	LPG 34:3	C40H75O9P	[M+NH4]+
748.5484093						
748.5484093	748.5487	0.0003	0.4008	LPG O-34:4;O	C40H75O9P	[M+NH4]+
	748.5487	0.0003	0.4008	PA 37:2;O	C40H75O9P	[M+NH4]+
748.5484093						
748.5484093	748.5487	0.0003	0.4008	PG O-34:3	C40H75O9P	[M+NH4]+
	748.5488	0.0004	0.5344	ACer 42:2;O5	C42H79NO7K	[M+K]+
748.5484093						
748.5484093	748.5488	0.0004	0.5344	Cer 42:3;O6	C42H79NO7K	[M+K]+
	749.3929	0.0001	0.1334	SQDG 31:7	C40H62O12S	[M+H-H2O]+
749.3928018						
750.4823433	750.4821	0.0003	0.3997	SHexCer 32:2;O2	C38H71NO11S	[M+H]+
	750.4821		0.3997	SHexCer 32:1;O3	C38H73NO12S	[M+H-H2O]+
750.4823433						
750.4823433	750.4834	0.0011	1.4657	CerP 40:4;O3	C40H74NO7PK	[M+K]+
	750.4834	0.0011	1.4657	LPC 32:4	C40H74NO7PK	[M+K]+
750.4823433						
750.4823433	750.4834	0.0011	1.4657	LPC O-32:5;O	C40H74NO7PK	[M+K]+
	750.4834	0.0011	1.4657	PC O-32:4	C40H74NO7PK	[M+K]+
750.4823433						
750.4823433	750.4834	0.0011	1.4657	PE O-35:4	C40H74NO7PK	[M+K]+
	751.4086	0.0008	1.0647	SQDG 31:6	C40H64O12S	[M+H-H2O]+
751.4077178						
751.4856314	751.4868	0.0012	1.5968	IPC 31:3;O2	C37H68NO11P	[M+NH4]+
	751.4868	0.0012	1.5968	PS 31:2;O	C37H68NO11P	[M+NH4]+
751.4856314						
752.4384834	752.4402	0.0017	2.2593	SHexCer 34:6;O2	C40H67NO11S	[M+H-H2O]+
	752.5072	0.0005	0.6644	IPC 32:1;O2	C38H74NO11P	[M+H]+
752.5077472						
752.5077472	752.5072	0.0005	0.6644	PS 32:0;O	C38H74NO11P	[M+H]+
	752.5072	0.0005	0.6644	IPC 32:0;O3	C38H76NO12P	[M+H-H2O]+
752.5077472						
752.5077472	752.5072	0.0005	0.6644	BMP 32:2;O	C38H71O11P	[M+NH4]+
	752.5072	0.0005	0.6644	LPI O-29:3	C38H71O11P	[M+NH4]+

752.5077472						
752.5077472	752.50720.0005		0.6644	PG 32:2;O	C38H71O11P	[M+NH4]+
	752.5073	0.0004	0.5316	HexCer 34:2;O3	C40H75NO9K	[M+K]+
752.5077472						
753.4460704	753.44660.0005		0.6636	PA 39:8	C42H67O8PNa	[M+Na]+
	753.4466	0.0005	0.6636	PA O-39:9;O	C42H67O8PNa	[M+Na]+
753.4460704						
753.4460704	753.4450.0011		1.4600	PS 33:7	C39H62NO10P	[M+NH4]+
	753.445	0.0011	1.4600	PS O-33:8;O	C39H62NO10P	[M+NH4]+
753.4460704						
753.4460704	753.44670.0007		0.9291	LPG 33:4	C39H71O9PK	[M+K]+
	753.4467	0.0007	0.9291	LPG O-33:5;O	C39H71O9PK	[M+K]+
753.4460704						
753.4460704	753.44670.0007		0.9291	PA 36:3;O	C39H71O9PK	[M+K]+
	753.4467	0.0007	0.9291	PG O-33:4	C39H71O9PK	[M+K]+
753.4460704						
756.4781618	756.47860.0004		0.5288	CerP 38:3;O6	C38H72NO10PNa	[M+Na]+
	756.4786	0.0004	0.5288	LPS 32:2;O	C38H72NO10PNa	[M+Na]+
756.4781618						
756.4781618	756.47860.0004		0.5288	PS 32:1	C38H72NO10PNa	[M+Na]+
	756.4786	0.0004	0.5288	PS O-32:2;O	C38H72NO10PNa	[M+Na]+
756.4781618						
756.4817467	756.4810.0007		0.9253	CerP 40:6;O6	C40H70NO10P	[M+H]+
	756.481	0.0007	0.9253	LPS 34:5;O	C40H70NO10P	[M+H]+
756.4817467						
756.4817467	756.4810.0007		0.9253	PS 34:4	C40H70NO10P	[M+H]+
	756.481	0.0007	0.9253	PS O-34:5;O	C40H70NO10P	[M+H]+
756.4817467						
756.4817467	756.4810.0007		0.9253	IPC 34:4;O2	C40H72NO11P	[M+H-H2O]+
	756.481	0.0007	0.9253	PS 34:3;O	C40H72NO11P	[M+H-H2O]+
756.4817467						
756.4817467	756.4810.0007		0.9253	BMP 34:6	C40H67O10P	[M+NH4]+
	756.481	0.0007	0.9253	LPG 34:7;O	C40H67O10P	[M+NH4]+
756.4817467						
756.4817467	756.4810.0007		0.9253	PG 34:6	C40H67O10P	[M+NH4]+
	756.481		0.9253	PG O-34:7;O	C40H67O10P	[M+NH4]+
756.4817467						
756.4817467	756.48110.0006		0.7931	ACer 42:6;O6	C42H71NO8K	[M+K]+
	756.4811	0.0006	0.7931	HexCer 36:6;O2	C42H71NO8K	[M+K]+

756.4817467						
756.4939036	756.49390.0000		0.0000	LPC 34:7	C42H72NO7PNa	[M+Na]+
	756.4939	0.0000	0.0000	PC O-34:7	C42H72NO7PNa	[M+Na]+
756.4939036						
756.4939036	756.49390.0000		0.0000	PE O-37:7	C42H72NO7PNa	[M+Na]+
	756.494	0.0001	0.1322	CerP 39:2;O4	C39H76NO8PK	[M+K]+
756.4939036						
756.4939036	756.4940.0001		0.1322	LPC 31:2;O	C39H76NO8PK	[M+K]+
	756.494	0.0001	0.1322	LPE 34:2;O	C39H76NO8PK	[M+K]+
756.4939036						
756.4939036	756.4940.0001		0.1322	LPS O-33:2	C39H76NO8PK	[M+K]+
	756.494	0.0001	0.1322	PC 31:1	C39H76NO8PK	[M+K]+
756.4939036						
756.4939036	756.4940.0001		0.1322	PC O-31:2;O	C39H76NO8PK	[M+K]+
	756.494	0.0001	0.1322	PE 34:1	C39H76NO8PK	[M+K]+
756.4939036						
756.4939036	756.4940.0001		0.1322	PE O-34:2;O	C39H76NO8PK	[M+K]+
	756.5302	0.0004	0.5287	CerP 43:6;O2	C43H76NO6PNa	[M+Na]+
756.5297963						
756.5297963	756.53040.0006		0.7931	CerP 40:1;O3	C40H80NO7PK	[M+K]+
	756.5304	0.0006	0.7931	LPC 32:1	C40H80NO7PK	[M+K]+
756.5297963						
756.5297963	756.53040.0006		0.7931	LPC O-32:2;O	C40H80NO7PK	[M+K]+
	756.5304	0.0006	0.7931	PC O-32:1	C40H80NO7PK	[M+K]+
756.5297963						
756.5297963	756.53040.0006		0.7931	PE O-35:1	C40H80NO7PK	[M+K]+
	756.5302	0.0002	0.2644	CerP 43:6;O2	C43H76NO6PNa	[M+Na]+
756.5304098						
756.5304098	756.53040.0000		0.0000	CerP 40:1;O3	C40H80NO7PK	[M+K]+
	756.5304	0.0000	0.0000	LPC 32:1	C40H80NO7PK	[M+K]+
756.5304098						
756.5304098	756.53040.0000		0.0000	LPC O-32:2;O	C40H80NO7PK	[M+K]+
	756.5304	0.0000	0.0000	PC O-32:1	C40H80NO7PK	[M+K]+
756.5304098						
756.5304098	756.53040.0000		0.0000	PE O-35:1	C40H80NO7PK	[M+K]+
	758.5306	0.0023	3.0322	CerP 39:1;O5	C39H78NO9PNa	[M+Na]+
758.5283728						
758.5283728	758.53060.0023		3.0322	LPS 33:0	C39H78NO9PNa	[M+Na]+
	758.5306	0.0023	3.0322	LPS O-33:1;O	C39H78NO9PNa	[M+Na]+

758.5283728						
758.5283728	758.53060.0023		3.0322	PC 31:0;O	C39H78NO9PNa	[M+Na]+
	758.5306	0.0023	3.0322	PE 34:0;O	C39H78NO9PNa	[M+Na]+
758.5283728						
758.5283728	758.53060.0023		3.0322	PS O-33:0	C39H78NO9PNa	[M+Na]+
	758.5483	0.0014	1.8456	PC O-37:7	C45H78NO7P	[M+H-H2O]+
758.5468922						
758.5468922	758.54830.0014		1.8456	PE O-40:7	C45H78NO7P	[M+H-H2O]+
	758.5459	0.0010	1.3183	CerP 43:5;O2	C43H78NO6PNa	[M+Na]+
758.5468922						
758.5468922	758.5460.0008		1.0546	CerP 40:0;O3	C40H82NO7PK	[M+K]+
	758.546	0.0008	1.0546	LPC 32:0	C40H82NO7PK	[M+K]+
758.5468922						
758.5468922	758.5460.0008		1.0546	LPC O-32:1;O	C40H82NO7PK	[M+K]+
	758.546		1.0546	PC O-32:0	C40H82NO7PK	[M+K]+
758.5468922						
758.5468922	758.5460.0008		1.0546	PE O-35:0	C40H82NO7PK	[M+K]+
	759.551	0.0004	0.5266	LPG 34:0	C40H81O9PNa	[M+Na]+
759.5506248						
759.5506248	759.5510.0004		0.5266	LPG O-34:1;O	C40H81O9PNa	[M+Na]+
	759.551	0.0004	0.5266	PG O-34:0	C40H81O9PNa	[M+Na]+
759.5506248						
760.3959488	760.3950.0009		1.1836	LPS 33:7	C39H64NO9PK	[M+K]+
	760.395	0.0009	1.1836	PC 31:7;O	C39H64NO9PK	[M+K]+
760.3959488						
760.3959488	760.3950.0009		1.1836	PE 34:7;O	C39H64NO9PK	[M+K]+
	760.395	0.0009	1.1836	PS O-33:7	C39H64NO9PK	[M+K]+
760.3959488						
762.5019545	762.50280.0009		1.1803	EPC 36:5;O6	C38H69N2O10P	[M+NH4]+
	762.5028	0.0009	1.1803	SM 33:5;O6	C38H69N2O10P	[M+NH4]+
762.5019545						
763.3744109	763.37360.0008		1.0480	PA 39:11	C42H61O8PK	[M+K]+
	763.5061	0.0007	0.9168	PA O-44:11	C47H73O7P	[M+H-H2O]+
763.5053668						
763.5053668	763.50620.0008		1.0478	DG O-46:13	C49H72O4K	[M+K]+
	763.6324	0.0004	0.5238	EPC 40:0;O3	C42H87N2O7P	[M+H]+
763.6319455						
763.6319455	763.63240.0004		0.5238	SM 37:0;O3	C42H87N2O7P	[M+H]+
	763.6324	0.0004	0.5238	CerP 42:1;O3	C42H84NO7P	[M+NH4]+

763.6319455						
763.6319455	763.63240.0004		0.5238	LPC 34:1	C42H84NO7P	[M+NH4]+
	763.6324	0.0004	0.5238	LPC O-34:2;O	C42H84NO7P	[M+NH4]+
763.6319455						
763.6319455	763.63240.0004		0.5238	PC O-34:1	C42H84NO7P	[M+NH4]+
	763.6324	0.0004	0.5238	PE O-37:1	C42H84NO7P	[M+NH4]+
763.6319455						
765.3254503	765.32220.0032		4.1812	PIP 22:1;O	C31H58O17P2	[M+H]+
	765.3222	0.0033	4.3119	PI 27:7;O	C36H55O14PNa	[M+Na]+
765.3254503						
765.3254503	765.32810.0026		3.3972	SQDG 28:6	C37H58O12SK	[M+K]+
	767.4035	0.0014	1.8243	SQDG 31:7	C40H62O12S	[M+H]+
767.4020790						
767.4020790	767.40110.0010		1.3031	SQDG 29:4	C38H64O12SNa	[M+Na]+
	767.4008	0.0012	1.5637	EPC 35:6;O6	C37H65N2O10PK	[M+K]+
767.4020790						
767.4020790	767.40080.0012		1.5637	SM 32:6;O6	C37H65N2O10PK	[M+K]+
	768.4058	0.0004	0.5206	IPC 31:5;O3	C37H64NO12PNa	[M+Na]+
768.4054815						
768.4054815	768.4060.0005		0.6507	IPC 28:0;O4	C34H68NO13PK	[M+K]+
	770.3487	0.0026	3.3751	IPC 29:6;O5	C35H58NO14PNa	[M+Na]+
770.3461056						
770.3461056	770.34880.0026		3.3751	PIP 21:0;O	C30H58O17P2	[M+NH4]+
	770.343	0.0031	4.0242	PS 33:9	C39H58NO10PK	[M+K]+
770.3461056						
770.5094535	770.50950.0001		0.1298	PC O-35:7	C43H74NO7PNa	[M+Na]+
	770.5095	0.0001	0.1298	PE O-38:7	C43H74NO7PNa	[M+Na]+
770.5094535						
770.5094535	770.50970.0002		0.2596	CerP 40:2;O4	C40H78NO8PK	[M+K]+
	770.5097	0.0002	0.2596	LPC 32:2;O	C40H78NO8PK	[M+K]+
770.5094535						
770.5094535	770.50970.0002		0.2596	LPS O-34:2	C40H78NO8PK	[M+K]+
	770.5097		0.2596	PC 32:1	C40H78NO8PK	[M+K]+
770.5094535						
770.5094535	770.50970.0002		0.2596	PC O-32:2;O	C40H78NO8PK	[M+K]+
	770.5097	0.0002	0.2596	PE 35:1	C40H78NO8PK	[M+K]+
770.5094535						
770.5094535	770.50970.0002		0.2596	PE O-35:2;O	C40H78NO8PK	[M+K]+
	770.5095	0.0006	0.7787	PC O-35:7	C43H74NO7PNa	[M+Na]+

770.5101134						
770.5101134	770.50950.0006		0.7787	PE O-38:7	C43H74NO7PNa	[M+Na]+
	770.5097	0.0005	0.6489	CerP 40:2;O4	C40H78NO8PK	[M+K]+
770.5101134						
770.5101134	770.50970.0005		0.6489	LPC 32:2;O	C40H78NO8PK	[M+K]+
	770.5097	0.0005	0.6489	LPS O-34:2	C40H78NO8PK	[M+K]+
770.5101134						
770.5101134	770.50970.0005		0.6489	PC 32:1	C40H78NO8PK	[M+K]+
	770.5097	0.0005	0.6489	PC O-32:2;O	C40H78NO8PK	[M+K]+
770.5101134						
770.5101134	770.50970.0005		0.6489	PE 35:1	C40H78NO8PK	[M+K]+
	770.5097	0.0005	0.6489	PE O-35:2;O	C40H78NO8PK	[M+K]+
770.5101134						
771.5129138	771.5130.0001		0.1296	IPC 31:1;O3	C37H72NO12P	[M+NH4]+
	772.5099	0.0026	3.3657	CerP 39:2;O6	C39H76NO10PNa	[M+Na]+
772.5073209						
772.5073209	772.50990.0026		3.3657	LPS 33:1;O	C39H76NO10PNa	[M+Na]+
	772.5099	0.0026	3.3657	PS 33:0	C39H76NO10PNa	[M+Na]+
772.5073209						
772.5073209	772.50990.0026		3.3657	PS O-33:1;O	C39H76NO10PNa	[M+Na]+
	772.5042	0.0031	4.0129	CerP 43:6;O2	C43H76NO6PK	[M+K]+
772.5073209						
772.5161696	772.51810.0020		2.5889	HexCer 33:1;O6	C39H75NO12Na	[M+Na]+
	773.5092	0.0000	0.0000	PA 40:5	C43H75O8PNa	[M+Na]+
773.5091899						
773.5091899	773.50920.0000		0.0000	PA O-40:6;O	C43H75O8PNa	[M+Na]+
	773.5093	0.0001	0.1293	LPG 34:1	C40H79O9PK	[M+K]+
773.5091899						
773.5091899	773.50930.0001		0.1293	LPG O-34:2;O	C40H79O9PK	[M+K]+
	773.5093	0.0001	0.1293	PA 37:0;O	C40H79O9PK	[M+K]+
773.5091899						
773.5091899	773.50930.0001		0.1293	PG O-34:1	C40H79O9PK	[M+K]+
	773.5116	0.0001	0.1293	PA 42:8	C45H73O8P	[M+H]+
773.5116943						
773.5116943	773.51160.0001		0.1293	PA O-42:9;O	C45H73O8P	[M+H]+
	773.5116	0.0001	0.1293	PA 42:7;O	C45H75O9P	[M+H-H2O]+
773.5116943						
773.5116943	773.51160.0001		0.1293	PG O-39:8	C45H75O9P	[M+H-H2O]+
	773.5117	0.0000	0.0000	CE 20:5;O4	C47H74O6K	[M+K]+

773.5116943						
773.5116943	773.51170.0000		0.0000	DG 44:9;O	C47H74O6K	[M+K]+
	773.5117	0.0000	0.0000	DG O-44:10;O2	C47H74O6K	[M+K]+
773.5116943						
773.5116943	773.51170.0000		0.0000	TG 44:8	C47H74O6K	[M+K]+
	773.5117	0.0000	0.0000	TG O-44:9;O	C47H74O6K	[M+K]+
773.5116943						
773.5285894	773.52870.0001		0.1293	IPC 31:0;O3	C37H74NO12P	[M+NH4]+
	773.5303	0.0000	0.0000	BMP 34:0	C40H79O10PNa	[M+Na]+
773.5302916						
773.5302916	773.53030.0000		0.0000	LPG 34:1;O	C40H79O10PNa	[M+Na]+
	773.5303	0.0000		PG 34:0	C40H79O10PNa	[M+Na]+
773.5302916						
773.5302916	773.53030.0000		0.0000	PG O-34:1;O	C40H79O10PNa	[M+Na]+
	773.548	0.0010	1.2927	PA O-43:8	C46H77O7P	[M+H]+
773.5489693						
773.5489693	773.5480.0010		1.2927	PA 43:6	C46H79O8P	[M+H-H2O]+
	773.548	0.0010	1.2927	PA O-43:7;O	C46H79O8P	[M+H-H2O]+
773.5489693						
773.5489693	773.54810.0009		1.1635	CE 21:4;O3	C48H78O5K	[M+K]+
	773.5481	0.0009	1.1635	DG 45:8	C48H78O5K	[M+K]+
773.5489693						
773.5489693	773.54810.0009		1.1635	DG O-45:9;O	C48H78O5K	[M+K]+
	773.5481	0.0009	1.1635	TG O-45:8	C48H78O5K	[M+K]+
773.5489693						
773.5505879	773.55220.0016		2.0684	HexCer 35:4;O5	C41H73NO11	[M+NH4]+
	774.5127	0.0002	0.2582	HexCer 36:5;O4	C42H73NO10Na	[M+Na]+
774.5128260						
774.5128260	774.51270.0001		0.1291	LPI 28:0;O	C37H73O13P	[M+NH4]+
	774.5127	0.0001	0.1291	PI O-28:0;O	C37H73O13P	[M+NH4]+
774.5128260						
774.5128260	774.51280.0000		0.0000	HexCer 33:0;O5	C39H77NO11K	[M+K]+
	774.5619	0.0006	0.7746	CerP 40:0;O5	C40H82NO9PNa	[M+Na]+
774.5625857						
774.5625857	774.56190.0006		0.7746	LPS O-34:0;O	C40H82NO9PNa	[M+Na]+
	775.566	0.0006	0.7736	DG 50:14	C53H76O5	[M+H-H2O]+
775.5665626						
775.5665626	775.5660.0006		0.7736	TG O-50:14	C53H76O5	[M+H-H2O]+
	775.5678	0.0013	1.6762	HexCer 35:3;O5	C41H75NO11	[M+NH4]+

775.5665626						
777.5854534	777.58510.0004		0.5144	TG 41:1;O3	C44H82O9Na	[M+Na]+
	778.4266	0.0008	1.0277	IPC 33:6;O2	C39H66NO11PNa	[M+Na]+
778.4258004						
778.4258004	778.42660.0008		1.0277	PS 33:5;O	C39H66NO11PNa	[M+Na]+
	778.4267	0.0009	1.1562	IPC 30:1;O3	C36H70NO12PK	[M+K]+
778.4258004						
778.4758463	778.4770.0011		1.4130	SHexCer 33:3;O3	C39H71NO12S	[M+H]+
	778.477	0.0011	1.4130	SHexCer 33:2;O4	C39H73NO13S	[M+H-H2O]+
778.4758463						
778.4758463	778.47460.0013		1.6699	SHexCer 31:0;O3	C37H73NO12SNa	[M+Na]+
	778.477	0.0011	1.4130	SQDG 30:3	C39H68O12S	[M+NH4]+
778.4758463						
780.4691903	780.47150.0023		2.9469	SHexCer 36:6;O2	C42H71NO11S	[M+H-H2O]+
	780.4715	0.0003	0.3844	SHexCer 36:6;O2	C42H71NO11S	[M+H-H2O]+
780.4711801						
781.4578387	781.45690.0009		1.1517	PA O-41:9	C44H71O7PK	[M+K]+
	781.4739	0.0006	0.7678	EPC 37:5;O6	C39H71N2O10PNa	[M+Na]+
781.4744077						
781.4744077	781.47390.0006		0.7678	SM 34:5;O6	C39H71N2O10PNa	[M+Na]+
	782.4004	0.0024	3.0675	PS 35:9	C41H62NO10PNa	[M+Na]+
782.4027782						
782.4027782	782.40040.0024		3.0675	PS O-35:10;O	C41H62NO10PNa	[M+Na]+
	782.4005	0.0023	2.9397	IPC 32:5;O2	C38H66NO11PK	[M+K]+
782.4027782						
782.4027782	782.40050.0023		2.9397	PS 32:4;O	C38H66NO11PK	[M+K]+
	783.375	0.0012	1.5318	SQDG 29:4	C38H64O12SK	[M+K]+
783.3762203						
784.3783786	784.37960.0012		1.5299	PS 34:9;O	C40H60NO11PNa	[M+Na]+
	784.3798		1.7848	IPC 31:5;O3	C37H64NO12PK	[M+K]+
784.3783786						
784.3819840	784.37960.0024		3.0597	PS 34:9;O	C40H60NO11PNa	[M+Na]+
	784.3798	0.0022	2.8048	IPC 31:5;O3	C37H64NO12PK	[M+K]+
784.3819840						
784.5252855	784.52520.0001		0.1275	PC O-36:7	C44H76NO7PNa	[M+Na]+
	784.5252	0.0001	0.1275	PE O-39:7	C44H76NO7PNa	[M+Na]+
784.5252855						
784.5252855	784.52530.0000		0.0000	CerP 41:2;O4	C41H80NO8PK	[M+K]+

784.5252855	784.5253	0.0000	0.0000	LPC 33:2;O	C41H80NO8PK	[M+K]+
784.5252855	784.52530.0000		0.0000	PC 33:1	C41H80NO8PK	[M+K]+
784.5252855	784.5253	0.0000	0.0000	PC O-33:2;O	C41H80NO8PK	[M+K]+
784.5252855	784.52530.0000		0.0000	PE 36:1	C41H80NO8PK	[M+K]+
784.5252855	784.5253	0.0000	0.0000	PE O-36:2;O	C41H80NO8PK	[M+K]+
785.5286366	785.52870.0000		0.0000	IPC 32:1;O3	C38H74NO12P	[M+NH4]+
785.5303330	785.5303	0.0000	0.0000	PG 35:1	C41H79O10PNa	[M+Na]+
785.5303330	785.53030.0000		0.0000	PG O-35:2;O	C41H79O10PNa	[M+Na]+
787.4868309	787.4868	0.0000	0.0000	IPC 34:6;O2	C40H68NO11P	[M+NH4]+
787.4868309	787.48680.0000		0.0000	PS 34:5;O	C40H68NO11P	[M+NH4]+
787.5635442	787.5636	0.0001	0.1270	PA O-44:8	C47H79O7P	[M+H]+
787.5635442	787.56360.0001		0.1270	PA 44:6	C47H81O8P	[M+H-H2O]+
787.5635442	787.5636	0.0001	0.1270	PA O-44:7;O	C47H81O8P	[M+H-H2O]+
787.5635442	787.56360.0000		0.0000	DG O-49:14	C52H76O4Na	[M+Na]+
787.5635442	787.5637	0.0002	0.2539	CE 22:4;O3	C49H80O5K	[M+K]+
787.5635442	787.56370.0002		0.2539	DG 46:8	C49H80O5K	[M+K]+
787.5635442	787.5637	0.0002	0.2539	DG O-46:9;O	C49H80O5K	[M+K]+
787.5635442	787.56370.0002		0.2539	TG O-46:8	C49H80O5K	[M+K]+
787.7147190	787.715	0.0003	0.3808	DG 46:0	C49H96O5Na	[M+Na]+
787.7147190	787.7150.0003		0.3808	DG O-46:1;O	C49H96O5Na	[M+Na]+
787.7147190	787.715	0.0003	0.3808	TG O-46:0	C49H96O5Na	[M+Na]+
789.3842926	789.38540.0011		1.3935	SQDG 31:7	C40H62O12SNa	[M+Na]+
791.5989403	791.5991	0.0002	0.2527	HexCer 36:2;O5	C42H79NO11	[M+NH4]+
791.6016304	791.60320.0015		1.8949	TG 44:4;O3	C47H82O9	[M+H]+
791.6016304	791.6032	0.0015	1.8949	MGDG 38:3	C47H84O10	[M+H-H2O]+
791.6016304	791.60080.0009		1.1369	TG 42:1;O3	C45H84O9Na	[M+Na]+
	791.6637	0.0002	0.2526	EPC 42:0;O3	C44H91N2O7P	[M+H]+

791.6635016						
791.6635016	791.6637	0.0002	0.2526	SM 39:0;O3	C44H91N2O7P	[M+H]+
	791.6637	0.0002	0.2526	CerP 44:1;O3	C44H88NO7P	[M+NH4]+
791.6635016						
791.6635016	791.6637	0.0002	0.2526	PC O-36:1	C44H88NO7P	[M+NH4]+
	791.6637	0.0002	0.2526	PE O-39:1	C44H88NO7P	[M+NH4]+
791.6635016						
792.3275961	792.3237	0.0039	4.9222	SHexCer 28:4;O6	C34H59NO15SK	[M+K]+
	793.5338		2.2683	IPC 34:3;O2	C40H74NO11P	[M+NH4]+
793.5319430						
793.5319430	793.5338	0.0018	2.2683	PS 34:2;O	C40H74NO11P	[M+NH4]+
	794.4484	0.0005	0.6294	SHexCer 34:5;O2	C40H69NO11SNa	[M+Na]+
794.4488396						
794.4488396	794.4485	0.0003	0.3776	SHexCer 31:0;O3	C37H73NO12SK	[M+K]+
	794.4521	0.0000	0.0000	PC O-36:10	C44H70NO7PK	[M+K]+
794.4521708						
794.4521708	794.4521	0.0000	0.0000	PE O-39:10	C44H70NO7PK	[M+K]+
	796.7389	0.0002	0.2510	ACer 49:0;O4	C49H97NO6	[M+H]+
796.7386591						
796.7386591	796.7389	0.0002	0.2510	Cer 49:1;O5	C49H97NO6	[M+H]+
	796.7389	0.0002	0.2510	Cer 49:0;O6	C49H99NO7	[M+H-H2O]+
796.7386591						
796.7386591	796.7389	0.0002	0.2510	DG 46:1;O	C49H94O6	[M+NH4]+
	796.7389	0.0002	0.2510	DG O-46:2;O2	C49H94O6	[M+NH4]+
796.7386591						
796.7386591	796.7389	0.0002	0.2510	TG 46:0	C49H94O6	[M+NH4]+
	796.7389	0.0002	0.2510	TG O-46:1;O	C49H94O6	[M+NH4]+
796.7386591						
797.6353054	797.6378	0.0025	3.1343	CerP 42:0;O5	C42H86NO9P	[M+NH4]+
	798.5396	0.0002	0.2505	SHexCer 34:0;O3	C40H79NO12S	[M+H]+
798.5397941						
798.5397941	798.5396	0.0002	0.2505	SQDG 31:0	C40H76O12S	[M+NH4]+
	798.5408	0.0000	0.0000	PC O-37:7	C45H78NO7PNa	[M+Na]+
798.5408145						
798.5408145	798.5408	0.0000	0.0000	PE O-40:7	C45H78NO7PNa	[M+Na]+
	798.541	0.0001	0.1252	CerP 42:2;O4	C42H82NO8PK	[M+K]+
798.5408145						
798.5408145	798.541	0.0001	0.1252	LPC 34:2;O	C42H82NO8PK	[M+K]+

798.5408145	798.541	0.0001	0.1252	PC 34:1	C42H82NO8PK	[M+K]+
798.5408145	798.541	0.0001	0.1252	PC O-34:2;O	C42H82NO8PK	[M+K]+
798.5408145	798.541	0.0001	0.1252	PE 37:1	C42H82NO8PK	[M+K]+
798.5408145	798.541	0.0001	0.1252	PE O-37:2;O	C42H82NO8PK	[M+K]+
799.5441868	799.5443	0.0001	0.1251	IPC 33:1;O3	C39H76NO12P	[M+NH4]+
800.3269824	800.3288	0.0018	2.2491	SHexCer 30:6;O5	C36H59NO14SK	[M+K]+
800.4836074	800.4837	0.0001	0.1249	PC 35:7;O	C43H72NO9PNa	[M+Na]+
800.4836074	800.4837	0.0001	0.1249	PE 38:7;O	C43H72NO9PNa	[M+Na]+
800.4836074	800.4837	0.0001	0.1249	PS O-37:7	C43H72NO9PNa	[M+Na]+
800.4836074	800.4838	0.0002	0.2498	CerP 40:3;O6	C40H76NO10PK	[M+K]+
800.4836074	800.4838	0.0002	0.2498	LPS 34:2;O	C40H76NO10PK	[M+K]+
800.4836074	800.4838	0.0002	0.2498	PS 34:1	C40H76NO10PK	[M+K]+
800.4836074	800.4838	0.0002	0.2498	PS O-34:2;O	C40H76NO10PK	[M+K]+
800.5383734	800.5412	0.0028	3.4976	CerP 41:2;O6	C41H80NO10PNa	[M+Na]+
800.5383734	800.5412	0.0028	3.4976	PS 35:0	C41H80NO10PNa	[M+Na]+
800.5383734	800.5412	0.0028	3.4976	PS O-35:1;O	C41H80NO10PNa	[M+Na]+
800.5383734	800.5355	0.0029	3.6226	CerP 45:6;O2	C45H80NO6PK	[M+K]+
800.5480011	800.5494	0.0014	1.7488	HexCer 35:1;O6	C41H79NO12Na	[M+Na]+
800.5480011	800.546		2.4983	TG 47:13;O	C50H70O7	[M+NH4]+
801.4870784	801.4872	0.0001	0.1248	IPC 31:2;O5	C37H70NO14P	[M+NH4]+
801.5421058	801.5429	0.0008	0.9981	PA 44:8	C47H77O8P	[M+H]+
801.5421058	801.5429	0.0008	0.9981	PA O-44:9;O	C47H77O8P	[M+H]+
801.5421058	801.5429	0.0008	0.9981	PA 44:7;O	C47H79O9P	[M+H-H2O]+
801.5421058	801.5429	0.0008	0.9981	PG O-41:8	C47H79O9P	[M+H-H2O]+
801.5421058	801.5428	0.0007	0.8733	DG 49:14	C52H74O5Na	[M+Na]+
801.5421058	801.5428	0.0007	0.8733	TG O-49:14	C52H74O5Na	[M+Na]+

801.5421058	801.543	0.0009	1.1228	CE 22:5;O4	C49H78O6K	[M+K]+
801.5421058	801.5430.0009		1.1228	DG 46:9;O	C49H78O6K	[M+K]+
801.5421058	801.543	0.0009	1.1228	DG O-46:10;O2	C49H78O6K	[M+K]+
801.5421058	801.5430.0009		1.1228	TG 46:8	C49H78O6K	[M+K]+
801.5421058	801.543	0.0009	1.1228	TG O-46:9;O	C49H78O6K	[M+K]+
801.5421058	801.54060.0015		1.8714	PA 39:0;O	C42H83O9PK	[M+K]+
801.5421058	801.5406	0.0015	1.8714	PG O-36:1	C42H83O9PK	[M+K]+
801.5543471	801.55410.0002		0.2495	PC O-38:10	C46H74NO7P	[M+NH4]+
801.5543471	801.5541	0.0002	0.2495	PE O-41:10	C46H74NO7P	[M+NH4]+
802.5454603	802.5440.0015		1.8691	HexCer 38:5;O4	C44H77NO10Na	[M+Na]+
802.5454603	802.5464	0.0009	1.1214	MGDG 37:8	C46H72O10	[M+NH4]+
802.5454603	802.5440.0015		1.8691	LPI 30:0;O	C39H77O13P	[M+NH4]+
802.5454603	802.544	0.0015	1.8691	PI O-30:0;O	C39H77O13P	[M+NH4]+
802.5454603	802.54410.0013		1.6198	HexCer 35:0;O5	C41H81NO11K	[M+K]+
803.6266237	803.6273	0.0007	0.8711	EPC 42:2;O4	C44H87N2O8P	[M+H]+
803.6266237	803.62730.0007		0.8711	SM 39:2;O4	C44H87N2O8P	[M+H]+
803.6266237	803.6273	0.0007	0.8711	EPC 42:1;O5	C44H89N2O9P	[M+H-H2O]+
803.6266237	803.62730.0007		0.8711	SM 39:1;O5	C44H89N2O9P	[M+H-H2O]+
803.6266237	803.6273	0.0007	0.8711	CerP 44:3;O4	C44H84NO8P	[M+NH4]+
803.6266237	803.62730.0007		0.8711	PC 36:2	C44H84NO8P	[M+NH4]+
803.6266237	803.6273	0.0007	0.8711	PC O-36:3;O	C44H84NO8P	[M+NH4]+
803.6266237	803.62730.0007		0.8711	PE 39:2	C44H84NO8P	[M+NH4]+
803.6266237	803.6273	0.0007	0.8711	PE O-39:3;O	C44H84NO8P	[M+NH4]+
804.6319859	804.63240.0004		0.4971	ACer 46:2;O6	C46H87NO8Na	[M+Na]+
804.6319859	804.6324	0.0004	0.4971	HexCer 40:2;O2	C46H87NO8Na	[M+Na]+
805.6428654	805.64290.0001		0.1241	EPC 42:1;O4	C44H89N2O8P	[M+H]+
	805.6429	0.0001	0.1241	SM 39:1;O4	C44H89N2O8P	[M+H]+

805.6428654						
805.6428654	805.64290.0001		0.1241	EPC 42:0;O5	C44H91N2O9P	[M+H-H2O]+
	805.6429	0.0001	0.1241	SM 39:0;O5	C44H91N2O9P	[M+H-H2O]+
805.6428654						
805.6428654	805.64290.0001		0.1241	CerP 44:2;O4	C44H86NO8P	[M+NH4]+
	805.6429		0.1241	PC 36:1	C44H86NO8P	[M+NH4]+
805.6428654						
805.6428654	805.64290.0001		0.1241	PC O-36:2;O	C44H86NO8P	[M+NH4]+
	805.6429	0.0001	0.1241	PE 39:1	C44H86NO8P	[M+NH4]+
805.6428654						
805.6428654	805.64290.0001		0.1241	PE O-39:2;O	C44H86NO8P	[M+NH4]+
	805.6793	0.0024	2.9789	EPC 43:0;O3	C45H93N2O7P	[M+H]+
805.6769291						
805.6769291	805.67930.0024		2.9789	SM 40:0;O3	C45H93N2O7P	[M+H]+
	805.6793	0.0024	2.9789	CerP 45:1;O3	C45H90NO7P	[M+NH4]+
805.6769291						
805.6769291	805.67930.0024		2.9789	PC O-37:1	C45H90NO7P	[M+NH4]+
	805.6793	0.0024	2.9789	PE O-40:1	C45H90NO7P	[M+NH4]+
805.6769291						
807.5094313	807.510.0006		0.7430	DGDG 26:1	C41H74O15	[M+H]+
	807.51	0.0011	1.3622	DGDG 26:1	C41H74O15	[M+H]+
807.5111428						
807.5111428	807.5130.0019		2.3529	IPC 34:4;O3	C40H72NO12P	[M+NH4]+
	808.3914	0.0039	4.8244	SHexCer 30:2;O5	C36H67NO14SK	[M+K]+
808.3952694						
808.4861842	808.48750.0014		1.7316	SHexCer 34:3;O4	C40H73NO13S	[M+H]+
	808.4875	0.0014	1.7316	SHexCer 34:2;O5	C40H75NO14S	[M+H-H2O]+
808.4861842						
808.4861842	808.48510.0011		1.3606	SHexCer 32:0;O4	C38H75NO13SNa	[M+Na]+
	808.4971	0.0011	1.3605	IPC 34:3;O4	C40H74NO13P	[M+H]+
808.4981407						
808.4981407	808.49710.0011		1.3605	IPC 34:2;O5	C40H76NO14P	[M+H-H2O]+
	808.4971	0.0011	1.3605	LPI 31:4;O	C40H71O13P	[M+NH4]+
808.4981407						
808.4981407	808.49710.0011		1.3605	PI 31:3	C40H71O13P	[M+NH4]+
	808.4971	0.0011	1.3605	PI O-31:4;O	C40H71O13P	[M+NH4]+
808.4981407						
808.4981407	808.49720.0010		1.2369	HexCer 36:4;O5	C42H75NO11K	[M+K]+

	808.5028	0.0003	0.3711	SHexCer 38:6;O2	C44H75NO11S	[M+H-H2O]+
808.5031357						
809.3972497	809.40020.0029		3.5829	LPI 31:6	C40H67O12PK	[M+K]+
	809.4002	0.0029	3.5829	LPI O-31:7;O	C40H67O12PK	[M+K]+
809.3972497						
809.3972497	809.40020.0029		3.5829	PI O-31:6	C40H67O12PK	[M+K]+
	809.4682	0.0010	1.2354	DGDG 28:5	C43H70O15	[M+H-H2O]+
809.4691344						
809.4709816	809.47120.0002		0.2471	PS 36:8;O	C42H66NO11P	[M+NH4]+
	809.4882	0.0005	0.6177	PA O-43:9	C46H75O7PK	[M+K]+
809.4887091						
809.4915043	809.49230.0008		0.9883	IPC 33:4;O4	C39H70NO13P	[M+NH4]+
	810.3953	0.0006	0.7404	PS 36:10;O	C42H62NO11PNa	[M+Na]+
810.3958353						
810.3958353	810.39540.0004		0.4936	IPC 33:6;O3	C39H66NO12PK	[M+K]+
	810.4763	0.0011	1.3572	IPC 33:3;O5	C39H72NO14P	[M+H]+
810.4752648						
810.4752648	810.47630.0011		1.3572	IPC 33:2;O6	C39H74NO15P	[M+H-H2O]+
	810.4739	0.0014	1.7274	IPC 31:0;O5	C37H74NO14PNa	[M+Na]+
810.4752648						
810.4752648	810.47630.0011		1.3572	PI 30:3;O	C39H69O14P	[M+NH4]+
	810.4764	0.0012	1.4806	HexCer 35:4;O6	C41H73NO12K	[M+K]+
810.4752648						
810.7893120	810.79090.0016		1.9734	Cer 51:0;O4	C51H103NO5	[M+H]+
	810.7909		1.9734	DG 48:0	C51H100O5	[M+NH4]+
810.7893120						
810.7893120	810.79090.0016		1.9734	DG O-48:1;O	C51H100O5	[M+NH4]+
	810.7909	0.0016	1.9734	TG O-48:0	C51H100O5	[M+NH4]+
810.7893120						
810.7909201	810.79090.0000		0.0000	Cer 51:0;O4	C51H103NO5	[M+H]+
	810.7909	0.0000	0.0000	DG 48:0	C51H100O5	[M+NH4]+
810.7909201						
810.7909201	810.79090.0000		0.0000	DG O-48:1;O	C51H100O5	[M+NH4]+
	810.7909	0.0000	0.0000	TG O-48:0	C51H100O5	[M+NH4]+
810.7909201						
811.4845647	811.48380.0007		0.8626	DGDG 28:4	C43H72O15	[M+H-H2O]+
	811.7902	0.0040	4.9274	DG O-52:3	C55H104O4	[M+H-H2O]+
811.7941952						
813.5023908	813.50250.0001		0.1229	PS 36:6;O	C42H70NO11P	[M+NH4]+

815.7461302	815.7463	0.0002	0.2452	DG 48:0	C51H100O5Na	[M+Na]+
815.7461302	815.7463	0.0002	0.2452	DG O-48:1;O	C51H100O5Na	[M+Na]+
815.7461302	815.7463	0.0002	0.2452	TG O-48:0	C51H100O5Na	[M+Na]+
816.5290409	816.529	0.0000	0.0000	SHexCer 37:4;O2	C43H77NO11S	[M+H]+
816.5290409	816.529	0.0000	0.0000	SHexCer 37:3;O3	C43H79NO12S	[M+H-H2O]+
816.5319977	816.5304	0.0016	1.9595	CerP 45:6;O3	C45H80NO7PK	[M+K]+
816.5319977	816.5304	0.0016	1.9595	PC O-37:6	C45H80NO7PK	[M+K]+
816.5319977	816.5304	0.0016	1.9595	PE O-40:6	C45H80NO7PK	[M+K]+
817.6784605	817.6793	0.0009	1.1007	EPC 44:1;O3	C46H93N2O7P	[M+H]+
817.6784605	817.6793	0.0009	1.1007	SM 41:1;O3	C46H93N2O7P	[M+H]+
817.6784605	817.6793	0.0009	1.1007	EPC 44:0;O4	C46H95N2O8P	[M+H-H2O]+
817.6784605	817.6793	0.0009	1.1007	SM 41:0;O4	C46H95N2O8P	[M+H-H2O]+
817.6784605	817.6793	0.0009	1.1007	CerP 46:2;O3	C46H90NO7P	[M+NH4]+
817.6784605	817.6793	0.0009	1.1007	PC O-38:2	C46H90NO7P	[M+NH4]+
817.6784605	817.6793	0.0009	1.1007	PE O-41:2	C46H90NO7P	[M+NH4]+
817.6808344	817.6793	0.0015	1.8345	EPC 44:1;O3	C46H93N2O7P	[M+H]+
817.6808344	817.6793	0.0015	1.8345	SM 41:1;O3	C46H93N2O7P	[M+H]+
817.6808344	817.6793	0.0015	1.8345	EPC 44:0;O4	C46H95N2O8P	[M+H-H2O]+
817.6808344	817.6793	0.0015	1.8345	SM 41:0;O4	C46H95N2O8P	[M+H-H2O]+
817.6808344	817.6793	0.0015	1.8345	CerP 46:2;O3	C46H90NO7P	[M+NH4]+
817.6808344	817.6793	0.0015	1.8345	PC O-38:2	C46H90NO7P	[M+NH4]+
817.6808344	817.6793	0.0015	1.8345	PE O-41:2	C46H90NO7P	[M+NH4]+
819.3754142	819.375	0.0004	0.4882	SQDG 32:7	C41H64O12SK	[M+K]+
819.6576412	819.6586	0.0009	1.0980	EPC 43:1;O4	C45H91N2O8P	[M+H]+
819.6576412	819.6586	0.0009	1.0980	SM 40:1;O4	C45H91N2O8P	[M+H]+
819.6576412	819.6586	0.0009	1.0980	EPC 43:0;O5	C45H93N2O9P	[M+H-H2O]+
	819.6586	0.0009	1.0980	SM 40:0;O5	C45H93N2O9P	[M+H-H2O]+

819.6576412						
819.6576412	819.65860.0009		1.0980	CerP 45:2;O4	C45H88NO8P	[M+NH4]+
	819.6586		1.0980	PC 37:1	C45H88NO8P	[M+NH4]+
819.6576412						
819.6576412	819.65860.0009		1.0980	PC O-37:2;O	C45H88NO8P	[M+NH4]+
	819.6586	0.0009	1.0980	PE 40:1	C45H88NO8P	[M+NH4]+
819.6576412						
819.6576412	819.65860.0009		1.0980	PE O-40:2;O	C45H88NO8P	[M+NH4]+
	820.3914	0.0035	4.2663	SHexCer 31:3;O5	C37H67NO14SK	[M+K]+
820.3948789						
821.4366080	821.43640.0002		0.2435	PG 39:11	C45H67O10PNa	[M+Na]+
	821.4366	0.0001	0.1217	LPI O-33:7	C42H71O11PK	[M+K]+
821.4366080						
821.4366080	821.43660.0001		0.1217	PG 36:6;O	C42H71O11PK	[M+K]+
	822.4668	0.0011	1.3374	SHexCer 34:4;O5	C40H71NO14S	[M+H]+
822.4656647						
822.4656647	822.46680.0011		1.3374	SHexCer 34:3;O6	C40H73NO15S	[M+H-H2O]+
	822.4644	0.0013	1.5806	SHexCer 32:1;O5	C38H73NO14SNa	[M+Na]+
822.4656647						
822.4788742	822.47970.0008		0.9727	SHexCer 36:5;O2	C42H73NO11SNa	[M+Na]+
	822.4798	0.0009	1.0943	SHexCer 33:0;O3	C39H77NO12SK	[M+K]+
822.4788742						
822.4836473	822.48460.0009		1.0942	Hex2Cer 28:3;O5	C40H71NO16	[M+H]+
	822.4846	0.0009	1.0942	Hex2Cer 28:2;O6	C40H73NO17	[M+H-H2O]+
822.4836473						
822.4836473	822.48340.0002		0.2432	PC O-38:10	C46H74NO7PK	[M+K]+
	822.4834	0.0002	0.2432	PE O-41:10	C46H74NO7PK	[M+K]+
822.4836473						
822.6433623	822.64290.0004		0.4862	HexCer 40:1;O3	C46H89NO9Na	[M+Na]+
	823.4675	0.0012	1.4573	PA 43:9	C46H73O8PK	[M+K]+
823.4686912						
823.4686912	823.46750.0012		1.4573	PA O-43:10;O	C46H73O8PK	[M+K]+
	823.4716	0.0007	0.8501	IPC 33:5;O5	C39H68NO14P	[M+NH4]+
823.4708787						
823.4849924	823.48380.0012		1.4572	DGDG 29:5	C44H72O15	[M+H-H2O]+
	823.4868	0.0018	2.1858	PS 37:8;O	C43H68NO11P	[M+NH4]+
823.4849924						
824.7694327	824.77020.0007		0.8487	ACer 51:0;O4	C51H101NO6	[M+H]+

824.7694327	824.7702	0.0007	0.8487	Cer 51:1;O5	C51H101NO6	[M+H]+
824.7694327	824.7702	0.0007	0.8487	Cer 51:0;O6	C51H103NO7	[M+H-H2O]+
824.7694327	824.7702	0.0007	0.8487	DG 48:1;O	C51H98O6	[M+NH4]+
824.7694327	824.7702	0.0007	0.8487	DG O-48:2;O2	C51H98O6	[M+NH4]+
824.7694327	824.7702	0.0007	0.8487	TG 48:0	C51H98O6	[M+NH4]+
824.7694327	824.7702	0.0007	0.8487	TG O-48:1;O	C51H98O6	[M+NH4]+
824.7705093	824.7702	0.0004	0.4850	ACer 51:0;O4	C51H101NO6	[M+H]+
824.7705093	824.7702	0.0004	0.4850	Cer 51:1;O5	C51H101NO6	[M+H]+
824.7705093	824.7702	0.0004	0.4850	Cer 51:0;O6	C51H103NO7	[M+H-H2O]+
824.7705093	824.7702	0.0004	0.4850	DG 48:1;O	C51H98O6	[M+NH4]+
824.7705093	824.7702	0.0004	0.4850	DG O-48:2;O2	C51H98O6	[M+NH4]+
824.7705093	824.7702	0.0004	0.4850	TG 48:0	C51H98O6	[M+NH4]+
824.7705093	824.7702	0.0004	0.4850	TG O-48:1;O	C51H98O6	[M+NH4]+
825.7734674	825.7694	0.0040	4.8440	CE 28:0;O2	C55H100O4	[M+H]+
825.7734674	825.7694		4.8440	DG O-52:5	C55H100O4	[M+H]+
825.7734674	825.7694	0.0040	4.8440	DG 52:3	C55H102O5	[M+H-H2O]+
825.7734674	825.7694	0.0040	4.8440	DG O-52:4;O	C55H102O5	[M+H-H2O]+
825.7734674	825.7694	0.0040	4.8440	TG O-52:3	C55H102O5	[M+H-H2O]+
826.4989035	826.4981	0.0008	0.9679	SHexCer 34:2;O5	C40H75NO14S	[M+H]+
826.4989035	826.4981	0.0008	0.9679	SHexCer 34:1;O6	C40H77NO15S	[M+H-H2O]+
826.4989035	826.4993	0.0004	0.4840	PC 37:8;O	C45H74NO9PNa	[M+Na]+
826.4989035	826.4993	0.0004	0.4840	PE 40:8;O	C45H74NO9PNa	[M+Na]+
826.4989035	826.4993	0.0004	0.4840	PS O-39:8	C45H74NO9PNa	[M+Na]+
826.4989035	826.4995	0.0006	0.7260	CerP 42:4;O6	C42H78NO10PK	[M+K]+
826.4989035	826.4995	0.0006	0.7260	PS 36:2	C42H78NO10PK	[M+K]+
826.4989035	826.4995	0.0006	0.7260	PS O-36:3;O	C42H78NO10PK	[M+K]+

826.5004771	826.5017	0.0013	1.5729	PC 39:11;O	C47H72NO9P	[M+H]+
826.5004771	826.5017	0.0013	1.5729	PE 42:11;O	C47H72NO9P	[M+H]+
826.5004771	826.5017	0.0013	1.5729	PS O-41:11	C47H72NO9P	[M+H]+
826.5004771	826.5017	0.0013	1.5729	PS 41:9	C47H74NO10P	[M+H-H2O]+
826.5004771	826.5017	0.0013	1.5729	PS O-41:10;O	C47H74NO10P	[M+H-H2O]+
826.5004771	826.4993	0.0011	1.3309	PC 37:8;O	C45H74NO9PNa	[M+Na]+
826.5004771	826.4993	0.0011	1.3309	PE 40:8;O	C45H74NO9PNa	[M+Na]+
826.5004771	826.4993	0.0011	1.3309	PS O-39:8	C45H74NO9PNa	[M+Na]+
826.5004771	826.5017	0.0013	1.5729	PA 44:12;O	C47H69O9P	[M+NH4]+
826.5004771	826.4995	0.0010	1.2099	CerP 42:4;O6	C42H78NO10PK	[M+K]+
826.5004771	826.4995	0.0010	1.2099	PS 36:2	C42H78NO10PK	[M+K]+
826.5004771	826.4995	0.0010	1.2099	PS O-36:3;O	C42H78NO10PK	[M+K]+
827.5027550	827.5029	0.0001	0.1208	IPC 33:3;O5	C39H72NO14P	[M+NH4]+
829.7047741	829.7045	0.0003	0.3616	PA O-46:1	C49H97O7P	[M+H]+
829.7047741	829.7045	0.0003	0.3616	PA O-46:0;O	C49H99O8P	[M+H-H2O]+
829.7047741	829.7044	0.0003	0.3616	CE 27:2;O2	C54H94O4Na	[M+Na]+
829.7047741	829.7044	0.0003	0.3616	DG O-51:7	C54H94O4Na	[M+Na]+
829.7047741	829.7046	0.0002	0.2410	DG 48:1	C51H98O5K	[M+K]+
829.7047741	829.7046	0.0002	0.2410	DG O-48:2;O	C51H98O5K	[M+K]+
829.7047741	829.7046	0.0002	0.2410	TG O-48:1	C51H98O5K	[M+K]+
830.3766502	830.3757	0.0009	1.0838	SHexCer 32:5;O5	C38H65NO14SK	[M+K]+
830.4432997	830.4426	0.0007	0.8429	IPC 33:4;O5	C39H70NO14PNa	[M+Na]+
830.5291226	830.5294	0.0003	0.3612	SHexCer 34:0;O5	C40H79NO14S	[M+H]+
830.5309734	830.5306	0.0003	0.3612	PC 37:6;O	C45H78NO9PNa	[M+Na]+
830.5309734	830.5306	0.0003	0.3612	PE 40:6;O	C45H78NO9PNa	[M+Na]+
830.5309734	830.5306	0.0003	0.3612	PS O-39:6	C45H78NO9PNa	[M+Na]+

830.5309734	830.5308		0.2408	CerP 42:2;O6	C42H82NO10PK	[M+K]+
830.5309734	830.5308	0.0002	0.2408	PS 36:0	C42H82NO10PK	[M+K]+
830.5309734	830.5308	0.0002	0.2408	PS O-36:1;O	C42H82NO10PK	[M+K]+
830.7069567	830.708	0.0010	1.2038	HexCer 42:0;O3	C48H95NO9	[M+H]+
830.7069567	830.708	0.0010	1.2038	TG 45:0;O3	C48H92O9	[M+NH4]+
831.5342096	831.5342	0.0000	0.0000	IPC 33:1;O5	C39H76NO14P	[M+NH4]+
831.7191639	831.7201	0.0009	1.0821	PA O-46:0	C49H99O7P	[M+H]+
831.7191639	831.7201	0.0009	1.0821	CE 27:1;O2	C54H96O4Na	[M+Na]+
831.7191639	831.7201	0.0009	1.0821	DG O-51:6	C54H96O4Na	[M+Na]+
831.7191639	831.7185	0.0007	0.8416	ACer 51:5;O4	C51H91NO6	[M+NH4]+
831.7191639	831.7185	0.0007	0.8416	Cer 51:6;O5	C51H91NO6	[M+NH4]+
831.7191639	831.7202	0.0011	1.3226	DG 48:0	C51H100O5K	[M+K]+
831.7191639	831.7202	0.0011	1.3226	DG O-48:1;O	C51H100O5K	[M+K]+
831.7191639	831.7202	0.0011	1.3226	TG O-48:0	C51H100O5K	[M+K]+
831.7207260	831.7201	0.0006	0.7214	PA O-46:0	C49H99O7P	[M+H]+
831.7207260	831.7201	0.0007	0.8416	CE 27:1;O2	C54H96O4Na	[M+Na]+
831.7207260	831.7201	0.0007	0.8416	DG O-51:6	C54H96O4Na	[M+Na]+
831.7207260	831.7202	0.0005	0.6012	DG 48:0	C51H100O5K	[M+K]+
831.7207260	831.7202	0.0005	0.6012	DG O-48:1;O	C51H100O5K	[M+K]+
831.7207260	831.7202	0.0005	0.6012	TG O-48:0	C51H100O5K	[M+K]+
832.4533711	832.4525	0.0008	0.9610	PS 37:6	C43H72NO10PK	[M+K]+
832.4533711	832.4525	0.0008	0.9610	PS O-37:7;O	C43H72NO10PK	[M+K]+
832.7234776	832.7266	0.0031	3.7227	EPC 45:1;O2	C47H95N2O6P	[M+NH4]+
832.7234776	832.7266	0.0031	3.7227	SM 42:1;O2	C47H95N2O6P	[M+NH4]+
833.6746265	833.6742	0.0004	0.4798	EPC 44:1;O4	C46H93N2O8P	[M+H]+
833.6746265	833.6742	0.0004	0.4798	SM 41:1;O4	C46H93N2O8P	[M+H]+
	833.6742	0.0004	0.4798	EPC 44:0;O5	C46H95N2O9P	[M+H-H2O]+

833.6746265						
833.6746265	833.67420.0004		0.4798	SM 41:0;O5	C46H95N2O9P	[M+H-H2O]+
	833.6742	0.0004	0.4798	CerP 46:2;O4	C46H90NO8P	[M+NH4]+
833.6746265						
833.6746265	833.67420.0004		0.4798	PC 38:1	C46H90NO8P	[M+NH4]+
	833.6742	0.0004	0.4798	PC O-38:2;O	C46H90NO8P	[M+NH4]+
833.6746265						
833.6746265	833.67420.0004		0.4798	PE 41:1	C46H90NO8P	[M+NH4]+
	833.6742	0.0004	0.4798	PE O-41:2;O	C46H90NO8P	[M+NH4]+
833.6746265						
834.5875480	834.58780.0003		0.3595	TG 48:11;O2	C51H76O8	[M+NH4]+
	834.5878	0.0003	0.3595	TG O-48:12;O3	C51H76O8	[M+NH4]+
834.5875480						
835.4867623	835.48680.0001		0.1197	PS 38:9;O	C44H68NO11P	[M+NH4]+
	837.5025	0.0000	0.0000	PS 38:8;O	C44H70NO11P	[M+NH4]+
837.5024450						
838.8219333	838.82220.0003		0.3576	Cer 53:0;O4	C53H107NO5	[M+H]+
	838.8222		0.3576	DG 50:0	C53H104O5	[M+NH4]+
838.8219333						
838.8219333	838.82220.0003		0.3576	DG O-50:1;O	C53H104O5	[M+NH4]+
	838.8222	0.0003	0.3576	TG O-50:0	C53H104O5	[M+NH4]+
838.8219333						
839.4641003	839.46650.0024		2.8590	IPC 33:5;O6	C39H68NO15P	[M+NH4]+
	839.4624	0.0017	2.0251	PA 43:9;O	C46H73O9PK	[M+K]+
839.4641003						
839.4641003	839.46240.0017		2.0251	PG O-40:10	C46H73O9PK	[M+K]+
	843.7776	0.0001	0.1185	DG 50:0	C53H104O5Na	[M+Na]+
843.7774545						
843.7774545	843.77760.0001		0.1185	DG O-50:1;O	C53H104O5Na	[M+Na]+
	843.7776	0.0001	0.1185	TG O-50:0	C53H104O5Na	[M+Na]+
843.7774545						
844.4223519	844.42190.0005		0.5921	IPC 33:5;O6	C39H68NO15PNa	[M+Na]+
	844.7752	0.0042	4.9717	ACer 54:3;O3	C54H101NO5	[M+H]+
844.7794907						
844.7794907	844.77520.0042		4.9717	Cer 54:4;O4	C54H101NO5	[M+H]+
	844.7752	0.0042	4.9717	ACer 54:2;O4	C54H103NO6	[M+H-H2O]+
844.7794907						
844.7794907	844.77520.0042		4.9717	Cer 54:3;O5	C54H103NO6	[M+H-H2O]+
	844.7752	0.0042	4.9717	CE 27:0;O3	C54H98O5	[M+NH4]+

844.7794907						
844.7794907	844.7752	0.0042	4.9717	DG 51:4	C54H98O5	[M+NH4]+
	844.7752	0.0042	4.9717	DG O-51:5;O	C54H98O5	[M+NH4]+
844.7794907						
844.7794907	844.7752	0.0042	4.9717	TG O-51:4	C54H98O5	[M+NH4]+
	845.6994	0.0004	0.4730	PA 46:0	C49H97O8P	[M+H]+
845.6989722						
845.6989722	845.6994	0.0004	0.4730	PA O-46:1;O	C49H97O8P	[M+H]+
	845.6994	0.0004	0.4730	PG O-43:0	C49H99O9P	[M+H-H2O]+
845.6989722						
845.6989722	845.6993	0.0004	0.4730	CE 27:2;O3	C54H94O5Na	[M+Na]+
	845.6993	0.0004	0.4730	DG 51:6	C54H94O5Na	[M+Na]+
845.6989722						
845.6989722	845.6993	0.0004	0.4730	DG O-51:7;O	C54H94O5Na	[M+Na]+
	845.6993	0.0004	0.4730	TG O-51:6	C54H94O5Na	[M+Na]+
845.6989722						
845.6989722	845.6977	0.0012	1.4189	ACer 51:6;O5	C51H89NO7	[M+NH4]+
	845.6995	0.0005	0.5912	DG 48:1;O	C51H98O6K	[M+K]+
845.6989722						
845.6989722	845.6995	0.0005	0.5912	DG O-48:2;O2	C51H98O6K	[M+K]+
	845.6995	0.0005	0.5912	TG 48:0	C51H98O6K	[M+K]+
845.6989722						
845.6989722	845.6995	0.0005	0.5912	TG O-48:1;O	C51H98O6K	[M+K]+
	845.7017	0.0007	0.8277	CE 29:5;O3	C56H92O5	[M+H]+
845.7010555						
845.7010555	845.7017	0.0007	0.8277	DG 53:9	C56H92O5	[M+H]+
	845.7017	0.0007	0.8277	DG O-53:10;O	C56H92O5	[M+H]+
845.7010555						
845.7010555	845.7017	0.0007	0.8277	TG O-53:9	C56H92O5	[M+H]+
	845.7017	0.0007	0.8277	CE 29:4;O4	C56H94O6	[M+H-H2O]+
845.7010555						
845.7010555	845.7017	0.0007	0.8277	DG 53:8;O	C56H94O6	[M+H-H2O]+
	845.7017	0.0007	0.8277	DG O-53:9;O2	C56H94O6	[M+H-H2O]+
845.7010555						
845.7010555	845.7017	0.0007	0.8277	TG 53:7	C56H94O6	[M+H-H2O]+
	845.7017		0.8277	TG O-53:8;O	C56H94O6	[M+H-H2O]+
845.7010555						
846.5561508	846.5573	0.0012	1.4175	Hex2Cer 32:3;O3	C44H79NO14	[M+H]+
	846.5573	0.0012	1.4175	Hex2Cer 32:2;O4	C44H81NO15	[M+H-H2O]+

846.5561508						
846.5561508	846.5549	0.0012	1.4175	Hex2Cer 30:0;O3	C42H81NO14Na	[M+Na]+
	846.7029	0.0003	0.3543	HexCer 42:0;O4	C48H95NO10	[M+H]+
846.7025614						
846.7025614	846.7029	0.0003	0.3543	MGDG 39:0	C48H92O10	[M+NH4]+
	848.48	0.0009	1.0607	SHexCer 34:2;O5	C40H75NO14SNa	[M+Na]+
848.4791487						
848.4813714	848.4825	0.0011	1.2964	SHexCer 36:5;O5	C42H73NO14S	[M+H]+
	848.4825	0.0011	1.2964	SHexCer 36:4;O6	C42H75NO15S	[M+H-H2O]+
848.4813714						
848.4813714	848.48	0.0013	1.5322	SHexCer 34:2;O5	C40H75NO14SNa	[M+Na]+
	848.6586	0.0003	0.3535	HexCer 42:2;O3	C48H91NO9Na	[M+Na]+
848.6583099						
848.6609403	848.661	0.0001	0.1178	HexCer 44:5;O3	C50H89NO9	[M+H]+
	848.661	0.0001	0.1178	HexCer 44:4;O4	C50H91NO10	[M+H-H2O]+
848.6609403						
848.6609403	848.661	0.0001	0.1178	TG 47:5;O3	C50H86O9	[M+NH4]+
	849.4677	0.0003	0.3532	PG 41:11	C47H71O10PNa	[M+Na]+
849.4674131						
849.4674131	849.4679	0.0004	0.4709	PG 38:6;O	C44H75O11PK	[M+K]+
	850.5017	0.0003	0.3527	PS 43:11	C49H74NO10P	[M+H-H2O]+
850.5014763						
850.5014763	850.5017	0.0003	0.3527	PS O-43:12;O	C49H74NO10P	[M+H-H2O]+
	855.6626	0.0003	0.3506	PA O-50:7	C53H93O7P	[M+H-H2O]+
855.6622812						
855.6622812	855.6627	0.0004	0.4675	CE 28:4;O2	C55H92O4K	[M+K]+
	855.6627	0.0004	0.4675	DG O-52:9	C55H92O4K	[M+K]+
855.6622812						
857.6351552	857.6354	0.0003	0.3498	EPC 43:1;O5	C45H91N2O9PNa	[M+Na]+
	857.6354	0.0003	0.3498	SM 40:1;O5	C45H91N2O9PNa	[M+Na]+
857.6351552						
858.7386898	858.7393	0.0006	0.6987	HexCer 44:0;O3	C50H99NO9	[M+H]+
	858.7393	0.0006	0.6987	TG 47:0;O3	C50H96O9	[M+NH4]+
858.7386898						
859.6891904	859.6899	0.0007	0.8142	EPC 46:2;O4	C48H95N2O8P	[M+H]+
	859.6899	0.0007	0.8142	SM 43:2;O4	C48H95N2O8P	[M+H]+
859.6891904						
859.6891904	859.6899	0.0007	0.8142	EPC 46:1;O5	C48H97N2O9P	[M+H-H2O]+
	859.6899	0.0007	0.8142	SM 43:1;O5	C48H97N2O9P	[M+H-H2O]+

859.6891904						
859.6891904	859.68990.0007		0.8142	CerP 48:3;O4	C48H92NO8P	[M+NH4]+
	859.6899	0.0007	0.8142	PC 40:2	C48H92NO8P	[M+NH4]+
859.6891904						
859.6891904	859.68990.0007		0.8142	PC O-40:3;O	C48H92NO8P	[M+NH4]+
	859.6899	0.0007	0.8142	PE 43:2	C48H92NO8P	[M+NH4]+
859.6891904						
859.6891904	859.68990.0007		0.8142	PE O-43:3;O	C48H92NO8P	[M+NH4]+
	859.6899	0.0006	0.6979	EPC 46:2;O4	C48H95N2O8P	[M+H]+
859.6904796						
859.6904796	859.68990.0006		0.6979	SM 43:2;O4	C48H95N2O8P	[M+H]+
	859.6899	0.0006	0.6979	EPC 46:1;O5	C48H97N2O9P	[M+H-H2O]+
859.6904796						
859.6904796	859.68990.0006		0.6979	SM 43:1;O5	C48H97N2O9P	[M+H-H2O]+
	859.6899		0.6979	CerP 48:3;O4	C48H92NO8P	[M+NH4]+
859.6904796						
859.6904796	859.68990.0006		0.6979	PC 40:2	C48H92NO8P	[M+NH4]+
	859.6899	0.0006	0.6979	PC O-40:3;O	C48H92NO8P	[M+NH4]+
859.6904796						
859.6904796	859.68990.0006		0.6979	PE 43:2	C48H92NO8P	[M+NH4]+
	859.6899	0.0006	0.6979	PE O-43:3;O	C48H92NO8P	[M+NH4]+
859.6904796						
859.7514987	859.75140.0001		0.1163	PA O-48:0	C51H103O7P	[M+H]+
	859.7514	0.0001	0.1163	CE 29:1;O2	C56H100O4Na	[M+Na]+
859.7514987						
859.7514987	859.75140.0001		0.1163	DG O-53:6	C56H100O4Na	[M+Na]+
	859.7515	0.0000	0.0000	DG 50:0	C53H104O5K	[M+K]+
859.7514987						
859.7514987	859.75150.0000		0.0000	DG O-50:1;O	C53H104O5K	[M+K]+
	859.7515	0.0000	0.0000	TG O-50:0	C53H104O5K	[M+K]+
859.7514987						
860.7550511	860.75790.0028		3.2529	EPC 47:1;O2	C49H99N2O6P	[M+NH4]+
	860.7579	0.0028	3.2529	SM 44:1;O2	C49H99N2O6P	[M+NH4]+
860.7550511						
861.4882594	861.48880.0006		0.6965	PI O-36:7	C45H75O12PNa	[M+Na]+
	861.489	0.0007	0.8125	LPI 33:2;O	C42H79O13PK	[M+K]+
861.4882594						
861.4882594	861.4890.0007		0.8125	PI 33:1	C42H79O13PK	[M+K]+
	861.489	0.0007	0.8125	PI O-33:2;O	C42H79O13PK	[M+K]+

861.4882594						
861.4910567	861.49120.0002		0.2322	PI O-38:10	C47H73O12P	[M+H]+
	861.4912	0.0002	0.2322	PI 38:8	C47H75O13P	[M+H-H2O]+
861.4910567						
861.4910567	861.49120.0002		0.2322	PI O-38:9;O	C47H75O13P	[M+H-H2O]+
	861.4914	0.0003	0.3482	MGDG 40:10	C49H74O10K	[M+K]+
861.4910567						
864.4551413	864.4540.0012		1.3882	SHexCer 34:2;O5C40H75NO14SK		[M+K]+
	864.6324	0.0002	0.2313	IPC 40:1;O2	C46H90NO11P	[M+H]+
864.6321979						
864.6321979	864.63240.0002		0.2313	PS 40:0;O	C46H90NO11P	[M+H]+
	864.6324	0.0002	0.2313	IPC 40:0;O3	C46H92NO12P	[M+H-H2O]+
864.6321979						
864.6321979	864.63240.0002		0.2313	PG 40:2;O	C46H87O11P	[M+NH4]+
	864.6325	0.0003	0.3470	HexCer 42:2;O3	C48H91NO9K	[M+K]+
864.6321979						
866.6480334	866.64810.0000		0.0000	IPC 40:0;O2	C46H92NO11P	[M+H]+
	866.648	0.0000	0.0000	ACer 51:6;O6	C51H89NO8Na	[M+Na]+
866.6480334						
866.6480334	866.6480.0000		0.0000	HexCer 45:6;O2	C51H89NO8Na	[M+Na]+
	866.6481	0.0000	0.0000	PG 40:1;O	C46H89O11P	[M+NH4]+
866.6480334						
866.6480334	866.64820.0002		0.2308	HexCer 42:1;O3	C48H93NO9K	[M+K]+
	866.8171	0.0003	0.3461	ACer 54:0;O4	C54H107NO6	[M+H]+
866.8168031						
866.8168031	866.81710.0003		0.3461	Cer 54:1;O5	C54H107NO6	[M+H]+
	866.8171	0.0003	0.3461	Cer 54:0;O6	C54H109NO7	[M+H-H2O]+
866.8168031						
866.8168031	866.81710.0003		0.3461	DG 51:1;O	C54H104O6	[M+NH4]+
	866.8171	0.0003	0.3461	DG O-51:2;O2	C54H104O6	[M+NH4]+
866.8168031						
866.8168031	866.81710.0003		0.3461	TG 51:0	C54H104O6	[M+NH4]+
	866.8171		0.3461	TG O-51:1;O	C54H104O6	[M+NH4]+
866.8168031						
868.6637319	868.66370.0000		0.0000	ACer 51:5;O6	C51H91NO8Na	[M+Na]+
	868.6637	0.0000	0.0000	HexCer 45:5;O2	C51H91NO8Na	[M+Na]+
868.6637319						
868.6637319	868.66370.0000		0.0000	PG 40:0;O	C46H91O11P	[M+NH4]+
	868.6638	0.0001	0.1151	HexCer 42:0;O3	C48H95NO9K	[M+K]+

868.6637319						
871.6144358	871.61470.0003		0.3442	EPC 43:2;O6	C45H89N2O10PNa	[M+Na]+
	871.6147	0.0003	0.3442	SM 40:2;O6	C45H89N2O10PNa	[M+Na]+
871.6144358						
872.6172848	872.61640.0009		1.0314	PC 43:8	C51H86NO8P	[M+H]+
	872.6164	0.0009	1.0314	PC O-43:9;O	C51H86NO8P	[M+H]+
872.6172848						
872.6172848	872.61640.0009		1.0314	PE 46:8	C51H86NO8P	[M+H]+
	872.6164	0.0009	1.0314	PE O-46:9;O	C51H86NO8P	[M+H]+
872.6172848						
872.6172848	872.61640.0009		1.0314	PC 43:7;O	C51H88NO9P	[M+H-H2O]+
	872.6164	0.0009	1.0314	PE 46:7;O	C51H88NO9P	[M+H-H2O]+
872.6172848						
872.6172848	872.61640.0009		1.0314	PS O-45:7	C51H88NO9P	[M+H-H2O]+
	872.6164	0.0009	1.0314	PA 48:9	C51H83O8P	[M+NH4]+
872.6172848						
872.6172848	872.61640.0009		1.0314	PA O-48:10;O	C51H83O8P	[M+NH4]+
	873.5159	0.0009	1.0303	SQDG 35:1	C44H82O12SK	[M+K]+
873.5149763						
873.7269870	873.7290.0020		2.2890	ACer 53:6;O5	C53H93NO7	[M+NH4]+
	873.7307	0.0003	0.3434	PA 48:0	C51H101O8P	[M+H]+
873.7310153						
873.7310153	873.73070.0003		0.3434	PA O-48:1;O	C51H101O8P	[M+H]+
	873.7307	0.0003	0.3434	PG O-45:0	C51H103O9P	[M+H-H2O]+
873.7310153						
873.7310153	873.73060.0004		0.4578	CE 29:2;O3	C56H98O5Na	[M+Na]+
	873.7306	0.0004	0.4578	DG 53:6	C56H98O5Na	[M+Na]+
873.7310153						
873.7310153	873.73060.0004		0.4578	DG O-53:7;O	C56H98O5Na	[M+Na]+
	873.7306	0.0004	0.4578	TG O-53:6	C56H98O5Na	[M+Na]+
873.7310153						
873.7310153	873.73080.0002		0.2289	DG 50:1;O	C53H102O6K	[M+K]+
	873.7308	0.0002	0.2289	DG O-50:2;O2	C53H102O6K	[M+K]+
873.7310153						
873.7310153	873.73080.0002		0.2289	TG 50:0	C53H102O6K	[M+K]+
	873.7308	0.0002	0.2289	TG O-50:1;O	C53H102O6K	[M+K]+
873.7310153						
874.5435620	874.5440.0004		0.4574	IPC 39:5;O4	C45H80NO13P	[M+H]+
	874.544	0.0004	0.4574	IPC 39:4;O5	C45H82NO14P	[M+H-H2O]+

874.5435620						
874.5435620	874.5440.0004		0.4574	PI 36:5	C45H77O13P	[M+NH4]+
	874.544	0.0004	0.4574	PI O-36:6;O	C45H77O13P	[M+NH4]+
874.5435620						
874.5435620	874.54410.0006		0.6861	HexCer 41:6;O5	C47H81NO11K	[M+K]+
	874.7342	0.0001	0.1143	HexCer 44:0;O4	C50H99NO10	[M+H]+
874.7340561						
874.7340561	874.73420.0001		0.1143	MGDG 41:0	C50H96O10	[M+NH4]+
	875.5045	0.0003	0.3427	PI O-37:7	C46H77O12PNa	[M+Na]+
875.5041973						
875.5041973	875.50460.0004		0.4569	LPI 34:2;O	C43H81O13PK	[M+K]+
	875.5046		0.4569	PI 34:1	C43H81O13PK	[M+K]+
875.5041973						
875.5041973	875.50460.0004		0.4569	PI O-34:2;O	C43H81O13PK	[M+K]+
	876.5315	0.0015	1.7113	Hex2Cer 32:4;O5	C44H77NO16	[M+H]+
876.5329835						
876.5329835	876.53150.0015		1.7113	Hex2Cer 32:3;O6	C44H79NO17	[M+H-H2O]+
	879.5706	0.0017	1.9328	IPC 38:4;O4	C44H80NO13P	[M+NH4]+
879.5688924						
879.5688924	879.56640.0024		2.7286	PA O-48:9	C51H85O7PK	[M+K]+
	881.4366	0.0008	0.9076	PG 41:11;O	C47H71O11PK	[M+K]+
881.4357137						
881.5365707	881.53270.0039		4.4241	PG 45:12	C51H77O10P	[M+H]+
	881.5327	0.0039	4.4241	PG 45:11;O	C51H79O11P	[M+H-H2O]+
881.5365707						
881.5365707	881.54030.0037		4.1972	SHexCer 37:4;O5	C43H77NO14S	[M+NH4]+
	881.5328	0.0037	4.1972	TG 50:12;O2	C53H78O8K	[M+K]+
881.5365707						
881.5365707	881.53280.0037		4.1972	TG O-50:13;O3	C53H78O8K	[M+K]+
	884.6351	0.0008	0.9043	CerP 47:2;O6	C47H92NO10PNa	[M+Na]+
884.6343480						
884.6343480	884.63510.0008		0.9043	PS 41:0	C47H92NO10PNa	[M+Na]+
	884.6351	0.0008	0.9043	PS O-41:1;O	C47H92NO10PNa	[M+Na]+
884.6343480						
885.4262263	885.4220.0043		4.8564	SQDG 37:9	C46H70O12SK	[M+K]+
	889.7831	0.0004	0.4495	DG 51:0;O2	C54H106O7Na	[M+Na]+
889.7826871						
889.7826871	889.78310.0004		0.4495	TG O-51:0;O2	C54H106O7Na	[M+Na]+
	889.7815	0.0012	1.3486	HexCer 45:0;O3	C51H101NO9	[M+NH4]+

889.7826871						
890.4681900	890.4661	0.0020	2.2460	MIPC 31:6;O2	C43H72NO16P	[M+H]+
	890.4661	0.0020	2.2460	MIPC 31:5;O3	C43H74NO17P	[M+H-H2O]+
890.4681900						
890.4681900	890.4696	0.0014	1.5722	SHexCer 36:3;O5	C42H77NO14SK	[M+K]+
	890.4733	0.0011	1.2353	PE 44:12;O	C49H74NO9PK	[M+K]+
890.4722210						
890.4722210	890.4733	0.0011	1.2353	PS O-43:12	C49H74NO9PK	[M+K]+
	892.4667	0.0005	0.5602	Hex2Cer 29:2;O6	C41H75NO17K	[M+K]+
892.4671614						
892.4720425	892.4735	0.0015	1.6807	PS 42:11;O	C48H72NO11PNa	[M+Na]+
	892.5063	0.0000	0.0000	SHexCer 36:2;O6	C42H79NO15SNa	[M+Na]+
892.5062181						
892.5062181	892.5053	0.0009	1.0084	DGDG 32:9	C47H70O15	[M+NH4]+
	893.5346	0.0002	0.2238	MIPC 28:0;O3	C40H78NO17P	[M+NH4]+
893.5347246						
894.5033257	894.5046	0.0012	1.3415	PC 41:10;O	C49H78NO9PK	[M+K]+
	894.5046	0.0012	1.3415	PE 44:10;O	C49H78NO9PK	[M+K]+
894.5033257						
894.5033257	894.5046	0.0012	1.3415	PS O-43:10	C49H78NO9PK	[M+K]+
	895.4967	0.0010	1.1167	PI 38:8;O	C47H75O14P	[M+H]+
895.4957276						
895.4957276	895.4943	0.0014	1.5634	PI 36:5;O	C45H77O14PNa	[M+Na]+
	896.4978	0.0004	0.4462	Hex2Cer 32:5;O5	C44H75NO16Na	[M+Na]+
896.4982200						
896.4982200	896.498	0.0003	0.3346	Hex2Cer 29:0;O6	C41H79NO17K	[M+K]+
	896.5048	0.0033	3.6810	PS 42:9;O	C48H76NO11PNa	[M+Na]+
896.5015173						
896.5015173	896.4978	0.0037	4.1272	Hex2Cer 32:5;O5	C44H75NO16Na	[M+Na]+
	896.505		3.9040	IPC 39:5;O3	C45H80NO12PK	[M+K]+
896.5015173						
896.5015173	896.498	0.0036	4.0156	Hex2Cer 29:0;O6	C41H79NO17K	[M+K]+
	896.5164	0.0010	1.1154	SHexCer 39:5;O4	C45H79NO13SNa	[M+Na]+
896.5174799						
896.5174799	896.5166	0.0009	1.0039	SHexCer 36:0;O5	C42H83NO14SK	[M+K]+
	897.4889	0.0023	2.5627	PIP 34:2	C43H80O16P2	[M+H-H2O]+
897.4865925						
897.4865925	897.4888	0.0022	2.4513	PI O-39:10	C48H75O12PNa	[M+Na]+
	897.489	0.0024	2.6741	PI 36:4	C45H79O13PK	[M+K]+

897.4865925						
897.4865925	897.4890.0024		2.6741	PI O-36:5;O	C45H79O13PK	[M+K]+
	898.49	0.0009	1.0017	MIPC 28:0;O3	C40H78NO17PNa	[M+Na]+
898.4890717						
900.6488657	900.64770.0012		1.3324	PC 45:8	C53H90NO8P	[M+H]+
	900.6477	0.0012	1.3324	PC O-45:9;O	C53H90NO8P	[M+H]+
900.6488657						
900.6488657	900.64770.0012		1.3324	PE 48:8	C53H90NO8P	[M+H]+
	900.6477	0.0012	1.3324	PE O-48:9;O	C53H90NO8P	[M+H]+
900.6488657						
900.6488657	900.64770.0012		1.3324	PC 45:7;O	C53H92NO9P	[M+H-H2O]+
	900.6477	0.0012	1.3324	PE 48:7;O	C53H92NO9P	[M+H-H2O]+
900.6488657						
900.6488657	900.64770.0012		1.3324	PS O-47:7	C53H92NO9P	[M+H-H2O]+
	900.6477	0.0012	1.3324	PA 50:9	C53H87O8P	[M+NH4]+
900.6488657						
900.6488657	900.64770.0012		1.3324	PA O-50:10;O	C53H87O8P	[M+NH4]+
	900.6477	0.0031	3.4420	PC 45:8	C53H90NO8P	[M+H]+
900.6507289						
900.6507289	900.64770.0031		3.4420	PC O-45:9;O	C53H90NO8P	[M+H]+
	900.6477	0.0031	3.4420	PE 48:8	C53H90NO8P	[M+H]+
900.6507289						
900.6507289	900.64770.0031		3.4420	PE O-48:9;O	C53H90NO8P	[M+H]+
	900.6477	0.0031	3.4420	PC 45:7;O	C53H92NO9P	[M+H-H2O]+
900.6507289						
900.6507289	900.64770.0031		3.4420	PE 48:7;O	C53H92NO9P	[M+H-H2O]+
	900.6477	0.0031	3.4420	PS O-47:7	C53H92NO9P	[M+H-H2O]+
900.6507289						
900.6507289	900.65350.0028		3.1089	HexCer 45:5;O4	C51H91NO10Na	[M+Na]+
	900.6536	0.0028	3.1089	PI O-37:0;O	C46H91O13P	[M+NH4]+
900.6507289						
900.6507289	900.64770.0031		3.4420	PA 50:9	C53H87O8P	[M+NH4]+
	900.6477	0.0031	3.4420	PA O-50:10;O	C53H87O8P	[M+NH4]+
900.6507289						
900.6507289	900.65370.0029		3.2199	HexCer 42:0;O5	C48H95NO11K	[M+K]+
	902.4332	0.0020	2.2162	SHexCer 36:5;O6	C42H73NO15SK	[M+K]+
902.4352065						
903.5352016	903.53580.0006		0.6641	PI O-39:7	C48H81O12PNa	[M+Na]+
	903.5359	0.0007	0.7747	PI 36:1	C45H85O13PK	[M+K]+

903.5352016						
903.5352016	903.5359	0.0007	0.7747	PI O-36:2;O	C45H85O13PK	[M+K]+
	903.5383	0.0031	3.4310	MGDG 43:10	C52H80O10K	[M+K]+
903.5352016						
904.4667731	904.4665	0.0002	0.2211	MIPC 28:2;O5	C40H74NO19P	[M+H]+
	904.4665	0.0002	0.2211	MIPC 28:1;O6	C40H76NO20P	[M+H-H2O]+
904.4667731						
904.4667731	904.4667	0.0001	0.1106	Hex2Cer 30:3;O6	C42H75NO17K	[M+K]+
	904.4735		0.6634	PS 43:12;O	C49H72NO11PNa	[M+Na]+
904.4729325						
906.4842064	906.4822	0.0020	2.2063	MIPC 28:1;O5	C40H76NO19P	[M+H]+
	906.4822	0.0020	2.2063	MIPC 28:0;O6	C40H78NO20P	[M+H-H2O]+
906.4842064						
906.4842064	906.4823	0.0019	2.0960	Hex2Cer 30:2;O6	C42H77NO17K	[M+K]+
	906.5243	0.0005	0.5516	SHexCer 39:5;O6	C45H79NO15S	[M+H]+
906.5238226						
908.4766618	908.4767	0.0001	0.1101	MIPC 31:5;O3	C43H74NO17P	[M+H]+
	908.4767	0.0001	0.1101	MIPC 31:4;O4	C43H76NO18P	[M+H-H2O]+
908.4766618						
908.4766618	908.4768	0.0002	0.2201	Hex2Cer 33:6;O4	C45H75NO15K	[M+K]+
	911.4471	0.0038	4.1692	PI O-39:11	C48H73O12PK	[M+K]+
911.4433627						
912.4747946	912.475	0.0002	0.2192	SHexCer 38:6;O6	C44H75NO15SNa	[M+Na]+
	913.4628	0.0040	4.3789	PI O-39:10	C48H75O12PK	[M+K]+
913.4587408						
913.4611149	913.4628	0.0017	1.8611	PI O-39:10	C48H75O12PK	[M+K]+
	913.4814	0.0042	4.5978	PIP 32:0	C41H80O16P2Na	[M+Na]+
913.4771829						
913.4830865	913.4838	0.0007	0.7663	PIP 34:3	C43H78O16P2	[M+H]+
	913.4838	0.0007	0.7663	PIP 34:2;O	C43H80O17P2	[M+H-H2O]+
913.4830865						
913.4830865	913.4837	0.0007	0.7663	PI 39:9	C48H75O13PNa	[M+Na]+
	913.4837	0.0007	0.7663	PI O-39:10;O	C48H75O13PNa	[M+Na]+
913.4830865						
913.4830865	913.4839	0.0008	0.8758	PI 36:4;O	C45H79O14PK	[M+K]+
	914.4873	0.0008	0.8748	MIPC 30:3;O4	C42H76NO18P	[M+H]+
914.4881130						
914.4881130	914.4873	0.0008	0.8748	MIPC 30:2;O5	C42H78NO19P	[M+H-H2O]+
	914.4874	0.0007	0.7655	Hex2Cer 32:4;O5	C44H77NO16K	[M+K]+

914.4881130						
914.4921122	914.49060.0015		1.6403	SHexCer 38:5;O6	C44H77NO15SNa	[M+Na]+
	914.4944	0.0023	2.5151	PS 42:8;O	C48H78NO11PK	[M+K]+
914.4921122						
918.4862641	918.48920.0029		3.1574	PS 44:12;O	C50H74NO11PNa	[M+Na]+
	918.4974	0.0017	1.8508	MIPC 33:6;O2	C45H76NO16P	[M+H]+
918.4991055						
918.4991055	918.49740.0017		1.8508	MIPC 33:5;O3	C45H78NO17P	[M+H-H2O]+
	918.5009	0.0018	1.9597	SHexCer 38:3;O5	C44H81NO14SK	[M+K]+
918.4991055						
918.5009170	918.50090.0000		0.0000	SHexCer 38:3;O5	C44H81NO14SK	[M+K]+
	919.4886	0.0003	0.3263	PG 45:12	C51H77O10PK	[M+K]+
919.4883132						
919.4919272	919.49440.0024		2.6101	PIP 33:1;O	C42H80O17P2	[M+H]+
	919.4943	0.0024	2.6101	PI 38:7;O	C47H77O14PNa	[M+Na]+
919.4919272						
919.4919272	919.48860.0033		3.5890	PG 45:12	C51H77O10PK	[M+K]+
	920.4978	0.0008	0.8691	MIPC 29:1;O5	C41H78NO19P	[M+H]+
920.4986310						
920.4986310	920.49780.0008		0.8691	MIPC 29:0;O6	C41H80NO20P	[M+H-H2O]+
	920.498	0.0007	0.7605	Hex2Cer 31:2;O6	C43H79NO17K	[M+K]+
920.4986310						
920.5021194	920.50480.0027		2.9332	PS 44:11;O	C50H76NO11PNa	[M+Na]+
	920.5376	0.0001	0.1086	SHexCer 38:2;O6	C44H83NO15SNa	[M+Na]+
920.5377096						
921.5057767	921.50420.0015		1.6278	PG 45:11	C51H79O10PK	[M+K]+
	921.5042		1.6278	PG O-45:12;O	C51H79O10PK	[M+K]+
921.5057767						
921.5435015	921.54470.0012		1.3022	IPC 39:6;O6	C45H78NO15P	[M+NH4]+
	934.456	0.0004	0.4281	MIPC 32:6;O5	C44H74NO19P	[M+H-H2O]+
934.4563934						
934.5553018	934.55560.0003		0.3210	SHexCer 41:5;O6	C47H83NO15S	[M+H]+
	936.5233	0.0007	0.7474	PI 40:10;O	C49H75O14P	[M+NH4]+
936.5225225						
936.5225225	936.52090.0016		1.7085	PIP 33:1;O	C42H80O17P2	[M+NH4]+
	936.521	0.0015	1.6017	IPC 38:2;O6	C44H84NO15PK	[M+K]+
936.5225225						
937.4979531	937.49920.0012		1.2800	PG 45:11;O	C51H79O11PK	[M+K]+
	937.5033	0.0006	0.6400	MIPC 32:6;O3	C44H74NO17P	[M+NH4]+

937.5026399						
939.4959315	939.4970.0011		1.1708	PIP 34:1	C43H82O16P2Na	[M+Na]+
	940.5063	0.0000	0.0000	SHexCer 40:6;O6	C46H79NO15SNa	[M+Na]+
940.5062592						
941.4923186	941.49410.0018		1.9119	PI O-41:10	C50H79O12PK	[M+K]+
	941.5127	0.0042	4.4609	PIP 34:0	C43H84O16P2Na	[M+Na]+
941.5084686						
941.5122404	941.51270.0004		0.4248	PIP 34:0	C43H84O16P2Na	[M+Na]+
	943.4732	0.0006	0.6359	PIP 38:7	C47H78O16P2	[M+H-H2O]+
943.4726481						
943.4726481	943.47330.0007		0.7419	PI 40:9	C49H77O13PK	[M+K]+
	943.4733	0.0007	0.7419	PI O-40:10;O	C49H77O13PK	[M+K]+
943.4726481						
944.4969737	944.49780.0009		0.9529	MIPC 31:3;O5	C43H78NO19P	[M+H]+
	944.4978	0.0009	0.9529	MIPC 31:2;O6	C43H80NO20P	[M+H-H2O]+
944.4969737						
944.4969737	944.49540.0015		1.5881	MIPC 29:0;O5	C41H80NO19PNa	[M+Na]+
	944.498	0.0010	1.0588	Hex2Cer 33:4;O6	C45H79NO17K	[M+K]+
944.4969737						
945.5088918	945.510.0011		1.1634	PIP 35:2;O	C44H82O17P2	[M+H]+
	945.51	0.0011	1.1634	PI 40:8;O	C49H79O14PNa	[M+Na]+
945.5088918						
945.5107285	945.510.0007		0.7403	PIP 35:2;O	C44H82O17P2	[M+H]+
	945.5124	0.0016	1.6922	PI 42:11;O	C51H77O14P	[M+H]+
945.5107285						
945.5107285	945.510.0008		0.8461	PI 40:8;O	C49H79O14PNa	[M+Na]+
	946.469	0.0030	3.1697	MIPC 32:4;O2	C44H78NO16PK	[M+K]+
946.4659746						
946.5182387	946.52050.0022		2.3243	PS 46:12;O	C52H78NO11PNa	[M+Na]+
	946.5205	0.0002	0.2113	PS 46:12;O	C52H78NO11PNa	[M+Na]+
946.5202709						
947.5194656	947.51990.0004		0.4222	PG 47:12	C53H81O10PK	[M+K]+
	947.5199	0.0018	1.8997	PG 47:12	C53H81O10PK	[M+K]+
947.5216688						
948.4747480	948.47160.0031		3.2684	MIPC 33:6;O5	C45H76NO19P	[M+H-H2O]+
	948.4788	0.0040	4.2173	PS 45:12;O	C51H76NO11PK	[M+K]+
948.4747480						
948.5138619	948.51510.0013		1.3706	PS 46:11	C52H80NO10PK	[M+K]+
	948.5151	0.0013	1.3706	PS O-46:12;O	C52H80NO10PK	[M+K]+

948.5138619						
948.5250185	948.5233	0.0018	1.8977	PI 41:11;O	C50H75O14P	[M+NH4]+
	949.4814	0.0043	4.5288	PIP 35:3	C44H80O16P2Na	[M+Na]+
949.4770528						
949.4832814	949.4838	0.0005	0.5266	PIP 37:6	C46H78O16P2	[M+H]+
	949.4838		0.5266	PIP 37:5;O	C46H80O17P2	[M+H-H2O]+
949.4832814						
949.4832814	949.4837	0.0005	0.5266	PI 42:12	C51H75O13PNa	[M+Na]+
	949.4839	0.0006	0.6319	PI 39:7;O	C48H79O14PK	[M+K]+
949.4832814						
952.5362299	952.5369	0.0007	0.7349	MIPC 32:1;O3	C44H84NO17PNa	[M+Na]+
	953.5443	0.0034	3.5656	SQDG 45:12	C54H80O12S	[M+H]+
953.5477267						
953.5477267	953.5515	0.0037	3.8802	PIP 38:2	C47H88O16P2	[M+H-H2O]+
	953.5514	0.0037	3.8802	PI O-43:10	C52H83O12PNa	[M+Na]+
953.5477267						
953.5477267	953.5516	0.0039	4.0900	PI 40:4	C49H87O13PK	[M+K]+
	953.5516	0.0039	4.0900	PI O-40:5;O	C49H87O13PK	[M+K]+
953.5477267						
960.5477036	960.5479	0.0002	0.2082	SHexCer 41:3;O5	C47H87NO14SK	[M+K]+
	961.4838	0.0000	0.0000	PIP 38:7	C47H78O16P2	[M+H]+
961.4837645						
961.4837645	961.4838	0.0000	0.0000	PIP 38:6;O	C47H80O17P2	[M+H-H2O]+
	961.4839	0.0001	0.1040	PI 40:8;O	C49H79O14PK	[M+K]+
961.4837645						
961.5542668	961.5565	0.0023	2.3920	PG 48:12;O	C54H83O11PNa	[M+Na]+
	961.5567	0.0024	2.4960	PI O-42:7	C51H87O12PK	[M+K]+
961.5542668						
962.4885083	962.4873	0.0012	1.2468	MIPC 34:6;O5	C46H78NO19P	[M+H-H2O]+
	962.4944	0.0025	2.5974	PS 46:12;O	C52H78NO11PK	[M+K]+
962.4918908						
963.4953071	963.497	0.0017	1.7644	PIP 36:3	C45H82O16P2Na	[M+Na]+
	963.6549	0.0003	0.3113	SHexCer 44:4;O4	C50H91NO13S	[M+NH4]+
963.6551912						
963.6551912	963.6563	0.0011	1.1415	EPC 50:5;O5	C52H97N2O9PK	[M+K]+
	963.6563	0.0011	1.1415	SM 47:5;O5	C52H97N2O9PK	[M+K]+
963.6551912						
964.5479717	964.5464	0.0015	1.5551	PS 47:10	C53H84NO10PK	[M+K]+
	964.5464	0.0015	1.5551	PS O-47:11;O	C53H84NO10PK	[M+K]+

964.5479717						
970.4565038	970.45370.0028		2.8852	MIPC 30:2;O5	C42H78NO19PK	[M+K]+
	970.4958	0.0001	0.1030	SHexCer 41:6;O6	C47H81NO15SK	[M+K]+
970.4957268						
970.6531452	970.65320.0000		0.0000	PS 50:9	C56H92NO10P	[M+H]+
	970.6532	0.0000	0.0000	PS O-50:10;O	C56H92NO10P	[M+H]+
970.6531452						
970.6531452	970.65320.0000		0.0000	PS 50:8;O	C56H94NO11P	[M+H-H2O]+
	970.6532	0.0000	0.0000	PG 50:11	C56H89O10P	[M+NH4]+
970.6531452						
970.6531452	970.65320.0000		0.0000	PG O-50:12;O	C56H89O10P	[M+NH4]+
	974.5518	0.0024	2.4627	PS 48:12;O	C54H82NO11PNa	[M+Na]+
974.5493862						
974.5503725	974.55180.0014		1.4366	PS 48:12;O	C54H82NO11PNa	[M+Na]+
	975.5512	0.0018	1.8451	PG 49:12	C55H85O10PK	[M+K]+
975.5530323						
977.5484921	977.55140.0029		2.9666	PI O-45:12	C54H83O12PNa	[M+Na]+
	977.5516	0.0031	3.1712	PI 42:6	C51H87O13PK	[M+K]+
977.5484921						
977.5484921	977.55160.0031		3.1712	PI O-42:7;O	C51H87O13PK	[M+K]+
	977.5668	0.0024	2.4551	PG 49:11	C55H87O10PK	[M+K]+
977.5644670						
977.5644670	977.56680.0024		2.4551	PG O-49:12;O	C55H87O10PK	[M+K]+
	978.6546		0.6131	SHexCer 45:3;O5	C51H95NO14S	[M+H]+
978.6552446						
978.6552446	978.65460.0006		0.6131	SHexCer 45:2;O6	C51H97NO15S	[M+H-H2O]+
	978.6558	0.0006	0.6131	PC 48:9;O	C56H94NO9PNa	[M+Na]+
978.6552446						
978.6552446	978.65580.0006		0.6131	PE 51:9;O	C56H94NO9PNa	[M+Na]+
	978.6558	0.0006	0.6131	PS O-50:9	C56H94NO9PNa	[M+Na]+
978.6552446						
978.6552446	978.6560.0007		0.7153	CerP 53:5;O6	C53H98NO10PK	[M+K]+
	978.656	0.0007	0.7153	PS 47:3	C53H98NO10PK	[M+K]+
978.6552446						
978.6552446	978.6560.0007		0.7153	PS O-47:4;O	C53H98NO10PK	[M+K]+
	979.6576	0.0006	0.6125	TG O-61:17	C64H92O5K	[M+K]+
979.6582426						
981.6487923	981.64850.0003		0.3056	DGDG 37:2	C52H94O15Na	[M+Na]+

	981.6509	0.0005	0.5093	DGDG 39:5	C54H92O15	[M+H]+
981.6514065						
982.6568406	982.659	0.0022	2.2388	IPC 44:2;O6	C50H96NO15P	[M+H]+
	982.659	0.0022	2.2388	MIPC 38:0;O2	C50H98NO16P	[M+H-H2O]+
982.6568406						
982.6568406	982.6591	0.0023	2.3406	Hex2Cer 40:2;O2	C52H97NO13K	[M+K]+
	985.4814	0.0044	4.4648	PIP2 33:0	C42H83O19P3	[M+H]+
985.4769951						
985.4769951	985.4814	0.0044	4.4648	PIP 38:6	C47H80O16P2Na	[M+Na]+
	985.4815	0.0045	4.5663	PIP 35:1;O	C44H84O17P2K	[M+K]+
985.4769951						
988.5961419	988.5968	0.0007	0.7081	MIPC 35:1;O4	C47H90NO18P	[M+H]+
	988.5968	0.0007	0.7081	MIPC 35:0;O5	C47H92NO19P	[M+H-H2O]+
988.5961419						
988.5961419	988.5969	0.0008	0.8092	Hex2Cer 37:2;O5	C49H91NO16K	[M+K]+
	988.6026	0.0010	1.0115	SHexCer 45:6;O6	C51H89NO15S	[M+H]+
988.6015378						
988.6015378	988.6002	0.0014	1.4161	SHexCer 43:3;O6	C49H91NO15SNa	[M+Na]+
	989.5151	0.0010	1.0106	PIP 40:7	C49H82O16P2	[M+H]+
989.5140761						
989.5140761	989.5151	0.0010	1.0106	PIP 40:6;O	C49H84O17P2	[M+H-H2O]+
	989.5127	0.0014	1.4148	PIP 38:4	C47H84O16P2Na	[M+Na]+
989.5140761						
989.5140761	989.5152	0.0011	1.1117	PI 42:8;O	C51H83O14PK	[M+K]+
	989.6032	0.0015	1.5158	PA 54:11;O	C57H91O9PK	[M+K]+
989.6047454						
989.6047454	989.6032	0.0015	1.5158	PG O-51:12	C57H91O9PK	[M+K]+
	990.5186	0.0002	0.2019	MIPC 36:6;O5	C48H82NO19P	[M+H-H2O]+
990.5184050						
990.5230821	990.5257	0.0026	2.6249	PS 48:12;O	C54H82NO11PK	[M+K]+
	991.5283	0.0017	1.7145	PIP 38:3	C47H86O16P2Na	[M+Na]+
991.5266530						
992.6340881	992.6339	0.0002	0.2015	SHexCer 45:4;O6	C51H93NO15S	[M+H]+
	992.6351	0.0010	1.0074	PS 50:9	C56H92NO10PNa	[M+Na]+
992.6340881						
992.6340881	992.6351	0.0010	1.0074	PS O-50:10;O	C56H92NO10PNa	[M+Na]+
	993.6386	0.0011	1.1070	IPC 44:5;O6	C50H90NO15P	[M+NH4]+
993.6375089						
993.6375089	993.6369	0.0006	0.6038	TG 61:17	C64H90O6K	[M+K]+

	998.6845	0.0002	0.2003	PS 52:9	C58H96NO10P	[M+H]+
998.6842776						
998.6842776	998.6845	0.0002	0.2003	PS O-52:10;O	C58H96NO10P	[M+H]+
	998.6845		0.2003	PS 52:8;O	C58H98NO11P	[M+H-H2O]+
998.6842776						
998.6842776	998.6845	0.0002	0.2003	PG 52:11	C58H93O10P	[M+NH4]+
	998.6845	0.0002	0.2003	PG O-52:12;O	C58H93O10P	[M+NH4]+
998.6842776						
1000.6370110	1000.6390	0.0019	1.8988	SHexCer 47:6;O5	C53H93NO14S	[M+H]+
	1000.6365	0.0005	0.4997	SHexCer 45:3;O5	C51H95NO14SNa	[M+Na]+
1000.6370110						
1001.6119780	1001.6114	0.0006	0.5990	PI 47:10	C56H89O13P	[M+H]+
	1001.6114	0.0006	0.5990	PI O-47:11;O	C56H89O13P	[M+H]+
1001.6119780						
1001.6119780	1001.6114	0.0006	0.5990	PI 47:9;O	C56H91O14P	[M+H-H2O]+
	1001.6382	0.0009	0.8985	SQDG 48:9	C57H92O12S	[M+H]+
1001.6373500						
1001.6373500	1001.6358	0.0015	1.4976	SQDG 46:6	C55H94O12SNa	[M+Na]+
	1001.6396	0.0015	1.4975	PA 56:11	C59H95O8PK	[M+K]+
1001.6411190						
1001.6411190	1001.6396	0.0015	1.4975	PA O-56:12;O	C59H95O8PK	[M+K]+
	1008.6052	0.0014	1.3881	SHexCer 46:6;O5	C52H91NO14SNa	[M+Na]+
1008.6066110						
1008.6066110	1008.6054	0.0012	1.1898	SHexCer 43:1;O6	C49H95NO15SK	[M+K]+
	1009.6782	0.0001	0.0990	Hex2Cer 39:3;O6	C51H93NO17	[M+NH4]+
1009.6783220						
1009.6822370	1009.6822	0.0000	0.0000	DGDG 41:5	C56H96O15	[M+H]+
	1012.5007	0.0043	4.2469	MIPC 33:2;O5	C45H84NO19PK	[M+K]+
1012.5049720						
1014.6164420	1014.6158	0.0006	0.5914	SHexCer 45:4;O6	C51H93NO15SNa	[M+Na]+
	1014.6148	0.0016	1.5770	DGDG 41:11	C56H84O15	[M+NH4]+
1014.6164420						
1015.6189280	1015.6189	0.0001	0.0985	PA 56:12;O	C59H93O9PK	[M+K]+
	1015.6189	0.0019	1.8708	PA 56:12;O	C59H93O9PK	[M+K]+
1015.6207970						
1020.6657950	1020.6652	0.0006	0.5879	SHexCer 47:4;O6	C53H97NO15S	[M+H]+
	1020.6664	0.0006	0.5879	PS 52:9	C58H96NO10PNa	[M+Na]+

1020.6657950						
1020.6657950	1020.6664	0.0006	0.5879	PS O-52:10;O	C58H96NO10PNa	[M+Na]+
	1020.6666	0.0008	0.7838	IPC 49:5;O2	C55H100NO11PK	[M+K]+
1020.6657950						
1020.6657950	1020.6666	0.0008	0.7838	PS 49:4;O	C55H100NO11PK	[M+K]+
	1021.6699	0.0012	1.1745	IPC 46:5;O6	C52H94NO15P	[M+NH4]+
1021.6687120						
1021.6687120	1021.6682	0.0005	0.4894	TG 63:17	C66H94O6K	[M+K]+
	1021.6682	0.0005	0.4894	TG O-63:18;O	C66H94O6K	[M+K]+
1021.6687120						
1021.6707160	1021.6715	0.0008	0.7830	PI 46:4	C55H99O13PNa	[M+Na]+
	1021.6715	0.0008	0.7830	PI O-46:5;O	C55H99O13PNa	[M+Na]+
1021.6707160						
1021.6707160	1021.6699	0.0008	0.7830	IPC 46:5;O6	C52H94NO15P	[M+NH4]+
	1028.5236	0.0012	1.1667	PIP2 35:1	C44H85O19P3	[M+NH4]+
1028.5247920						
1028.5247920	1028.526	0.0012	1.1667	PIP 42:10	C51H80O16P2	[M+NH4]+
	1028.6315	0.0011	1.0694	SHexCer 46:4;O6	C52H95NO15SNa	[M+Na]+
1028.6325560						
1028.6684280	1028.6678	0.0006	0.5833	SHexCer 47:3;O5	C53H99NO14SNa	[M+Na]+
	1028.6703	0.0004	0.3889	SHexCer 49:6;O5	C55H97NO14S	[M+H]+
1028.6706140						
1028.6706140	1028.6703	0.0004	0.3889	SHexCer 49:5;O6	C55H99NO15S	[M+H-H2O]+
	1028.6715		0.8749	PC 52:12;O	C60H96NO9PNa	[M+Na]+
1028.6706140						
1028.6706140	1028.6715	0.0009	0.8749	PE 55:12;O	C60H96NO9PNa	[M+Na]+
	1028.6715	0.0009	0.8749	PS O-54:12	C60H96NO9PNa	[M+Na]+
1028.6706140						
1028.6706140	1028.6716	0.0010	0.9721	PS 51:6	C57H100NO10PK	[M+K]+
	1028.6716	0.0010	0.9721	PS O-51:7;O	C57H100NO10PK	[M+K]+
1028.6706140						
1036.6377720	1036.6365	0.0012	1.1576	SHexCer 48:6;O5	C54H95NO14SNa	[M+Na]+
	1036.6367	0.0011	1.0611	SHexCer 45:1;O6	C51H99NO15SK	[M+K]+
1036.6377720						
1036.6419690	1036.6403	0.0016	1.5434	PS 52:9	C58H96NO10PK	[M+K]+
	1036.6403	0.0016	1.5434	PS O-52:10;O	C58H96NO10PK	[M+K]+
1036.6419690						
1038.5032650	1038.508	0.0047	4.5257	PIP2 36:3	C45H83O19P3	[M+NH4]+

1042.6476930	1042.6471	0.0006	0.5755	SHexCer 47:4;O6	C53H97NO15SNa	[M+Na]+
1042.6476930	1042.64610.0015		1.4386	DGDG 43:11	C58H88O15	[M+NH4]+
1043.6493510	1043.6502	0.0008	0.7665	PA 58:12;O	C61H97O9PK	[M+K]+
1043.6512490	1043.65020.0011		1.0540	PA 58:12;O	C61H97O9PK	[M+K]+
1046.4872110	1046.485	0.0022	2.1023	MIPC 36:6;O5	C48H82NO19PK	[M+K]+
1048.4877250	1048.49230.0046		4.3873	PIP2 37:5	C46H81O19P3	[M+NH4]+
1048.4919090	1048.4923	0.0004	0.3815	PIP2 37:5	C46H81O19P3	[M+NH4]+
1054.5381720	1054.53930.0011		1.0431	PIP2 37:2	C46H87O19P3	[M+NH4]+
1054.5412860	1054.5416	0.0003	0.2845	PIP 44:11	C53H82O16P2	[M+NH4]+
1056.6049900	1056.60540.0004		0.3786	SHexCer 47:5;O6	C53H95NO15SK	[M+K]+
1070.5731120	1070.5729	0.0002	0.1868	PIP 45:10	C54H86O16P2	[M+NH4]+
1074.5168760	1074.51630.0006		0.5584	MIPC 38:6;O5	C50H86NO19PK	[M+K]+
1084.5538100	1084.5522	0.0016	1.4753	PIP 45:11;O	C54H84O17P2	[M+NH4]+
1090.0349620	1090.03590.0009		0.8257	DG 67:1;O2	C70H136O7	[M+H]+
1090.0349620	1090.0359	0.0009	0.8257	TG 67:0;O	C70H136O7	[M+H]+
1090.0349620	1090.03590.0009		0.8257	TG O-67:1;O2	C70H136O7	[M+H]+
1090.0349620	1090.0359	0.0009	0.8257	TG O-67:0;O3	C70H138O8	[M+H-H2O]+
1092.4872110	1092.49040.0032		2.9291	M(IP)2C 29:3;O2	C47H85NO24P2	[M+H-H2O]+
1094.5045470	1094.506	0.0015	1.3705	M(IP)2C 29:2;O2	C47H87NO24P2	[M+H-H2O]+
1098.5033630	1098.5010.0024		2.1848	M(IP)2C 28:2;O2	C46H85NO24P2	[M+H]+
1098.5033630	1098.501	0.0024	2.1848	M(IP)2C 28:1;O3	C46H87NO25P2	[M+H-H2O]+
1111.5244560	1111.5260.0015		1.3495	PIP2 41:4	C50H91O19P3Na	[M+Na]+
1112.5850650	1112.5835	0.0016	1.4381	PIP 47:11;O	C56H88O17P2	[M+NH4]+
1117.5154700	1117.51540.0000		0.0000	PIP3 38:0	C47H94O22P4	[M+H-H2O]+
1117.5154700	1117.5156	0.0001	0.0895	PIP2 40:2	C49H93O19P3K	[M+K]+
1121.5147790	1121.51270.0021		1.8725	PIP2 44:9	C53H87O19P3	[M+H]+

	1121.5127	0.0021	1.8725	PIP2 44:8;O	C53H89O20P3	[M+H-H2O]+
1121.5147790						
1121.5147790	1121.51280.0019		1.6941	PIP 46:10;O	C55H88O17P2K	[M+K]+
	1123.526	0.3560		PIP2 42:5	C51H91O19P3Na	[M+Na]+
1123.5263490						
1123.5263490	1123.52610.0002		0.1780	PIP2 39:0;O	C48H95O20P3K	[M+K]+
	1123.5284	0.0030	2.6702	PIP2 44:8	C53H89O19P3	[M+H]+
1123.5313420						
1123.5313420	1123.52840.0030		2.6702	PIP2 44:7;O	C53H91O20P3	[M+H-H2O]+
	1123.5285	0.0029	2.5812	PIP 46:9;O	C55H90O17P2K	[M+K]+
1123.5313420						
1124.5158990	1124.51660.0007		0.6225	M(IP)2C 30:3;O2	C48H87NO24P2	[M+H]+
	1124.5166	0.0007	0.6225	M(IP)2C 30:2;O3	C48H89NO25P2	[M+H-H2O]+
1124.5158990						
1124.5158990	1124.51420.0017		1.5118	M(IP)2C 28:0;O2	C46H89NO24P2Na	[M+Na]+
	1124.5236	0.0015	1.3339	PIP2 43:9	C52H85O19P3	[M+NH4]+
1124.5250710						
1126.5346660	1126.53230.0024		2.1304	M(IP)2C 30:2;O2	C48H89NO24P2	[M+H]+
	1126.5323	0.0024	2.1304	M(IP)2C 30:1;O3	C48H91NO25P2	[M+H-H2O]+
1126.5346660						
1133.5155250	1133.51270.0028		2.4702	PIP2 45:10	C54H87O19P3	[M+H]+
	1133.5127	0.0028	2.4702	PIP2 45:9;O	C54H89O20P3	[M+H-H2O]+
1133.5155250						
1133.5155250	1133.51280.0027		2.3820	PIP 47:11;O	C56H88O17P2K	[M+K]+
	1137.5416	0.0056	4.9229	PIP2 43:5	C52H93O19P3Na	[M+Na]+
1137.5359830						
1138.4952590	1138.49590.0006		0.5270	M(IP)2C 30:4;O3	C48H85NO25P2	[M+H]+
	1138.4959	0.0006	0.5270	M(IP)2C 30:3;O4	C48H87NO26P2	[M+H-H2O]+
1138.4952590						
1152.5470440	1152.54790.0009		0.7809	M(IP)2C 32:3;O2	C50H91NO24P2	[M+H]+
	1152.5479	0.0009	0.7809	M(IP)2C 32:2;O3	C50H93NO25P2	[M+H-H2O]+
1152.5470440						
1152.5470440	1152.54550.0015		1.3015	M(IP)2C 30:0;O2	C48H93NO24P2Na	[M+Na]+
	1152.5549	0.0021	1.8220	PIP2 45:9	C54H89O19P3	[M+NH4]+
1152.5570090						
1156.6777550	1156.68010.0024		2.0749	PIP2 44:0	C53H105O19P3	[M+NH4]+
	1165.5279	0.0058	4.9763	M(IP)2C 28:1;O5	C46H87NO27P2	[M+NH4]+

1165.5220930						
1166.5265220	1166.52720.0006		0.5143	M(IP)2C 32:4;O3	C50H89NO25P2	[M+H]+
	1166.5272	0.0006	0.5143	M(IP)2C 32:3;O4	C50H91NO26P2	[M+H-H2O]+
1166.5265220						
1166.5265220	1166.53180.0053		4.5434	PIP3 38:1;O	C47H92O23P4	[M+NH4]+
	1167.5311	0.0044	3.7686	PIP3 42:3	C51H96O22P4	[M+H-H2O]+
1167.5267370						
1167.5267370	1167.52240.0043		3.6830	M(IP)2C 31:5;O3	C49H85NO25P2	[M+NH4]+
1167.5267370	1167.53120.0045		3.8543	PIP2 44:5	C53H95O19P3K	[M+K]+

Supplementary Table S8: Annotations for downregulated ion signals in negative-ion mode

Input Mass	Matched Mass	Delta	ppm	Name	Formula	Adduct
	303.1005	0.001	2.6394	FA 14:4;O3	C14H20O5	[M+Cl]-
303.1012710						
333.1181174	333.1166	0.002	4.5029	ST 18:4;O;S	C18H22O4S	[M-H]-
	343.1083	0.000	1.1658	LPA O-10:2	C13H25O6P	[M+Cl]-
343.1078485						
347.1146895	347.1136	0.001	3.1690	ST 18:5;O7	C18H20O7	[M-H]-
347.1259422	347.1265	0.001	1.7285	LPA 12:3	C15H25O7P	[M-H]-
	347.1267	0.001	2.3046	FA 16:4;O4	C16H24O6	[M+Cl]-
347.1259422						
349.1286653	349.1293	0.001	1.7186	ST 18:4;O7	C18H22O7	[M-H]-
	353.1161	0.000	0.2832	ST 18:4;O5	C18H22O5	[M+Cl]-
353.1160287						
356.0928383	356.094	0.001	3.3699	NAT 11:2;O2	C13H23NO6S	[M+Cl]-
	357.0876	0.000	0.8401	LPA 10:2	C13H23O7P	[M+Cl]-
357.0872371						
357.1237710	357.1239	0.000	0.5600	LPA O-11:2	C14H27O6P	[M+Cl]-
	359.1032	0.000	0.2785	LPA 10:1	C13H25O7P	[M+Cl]-
359.1031003						
359.1031003	359.1032	0.000	0.2785	LPA O-10:2;O	C13H25O7P	[M+Cl]-
	363.1214	0.001	1.3769	LPA 12:3;O	C15H25O8P	[M-H]-
363.1209562						
365.1247101	365.1242	0.001	1.3694	ST 18:4;O8	C18H22O8	[M-H]-
	367.1221	0.002	4.0858	ST 18:3;O3;S	C18H24O6S	[M-H]-
367.1235888						
370.1173109	370.1177	0.000	1.0807	NAT 10:1;O3	C12H23NO7S	[M+Formate]-

	371.1032	0.000	0.8084	LPA 11:2	C14H25O7P	[M+Cl]-
371.1028573						
371.1028573	371.1032	0.000	0.8084	LPA O-11:3;O	C14H25O7P	[M+Cl]-
	373.0825	0.001	1.3402	LPA 10:2;O	C13H23O8P	[M+Cl]-
373.0819212						
373.1185564	373.1189	0.000	0.8040	LPA 11:1	C14H27O7P	[M+Cl]-
	373.1189	0.000	0.8040	LPA O-11:2;O	C14H27O7P	[M+Cl]-
373.1185564						
376.1418440	376.1436	0.002	4.5196	NAT 14:3;O3	C16H27NO7S	[M-H]-
	376.1436	0.002	4.5196	NAT 13:3;O	C15H25NO5S	[M+Formate]-
376.1418440						
376.1418440	376.1436	0.002	4.5196	NAT 12:3;O	C14H23NO5S	[M+OAc]-
	377.1242	0.001	2.9168	ST 19:5;O8	C19H22O8	[M-H]-
377.1252604						
377.1252604	377.1242	0.001	2.9168	ST 18:5;O6	C18H20O6	[M+Formate]-
	381.132	0.000	1.0495	LPA 11:2	C14H25O7P	[M+Formate]-
381.1316434						
381.1316434	381.132	0.000	1.0495	LPA O-11:3;O	C14H25O7P	[M+Formate]-
	381.132	0.000	1.0495	LPA 10:2	C13H23O7P	[M+OAc]-
381.1316434						
384.1323511	384.1334	0.001	2.6033	NAT 11:1;O3	C13H25NO7S	[M+Formate]-
	384.1334	0.001	2.6033	NAT 10:1;O3	C12H23NO7S	[M+OAc]-
384.1323511						
385.0821438	385.0825	0.000	0.7791	LPA 11:3;O	C14H23O8P	[M+Cl]-
	386.1141	0.000	0.7770	SPBP 14:3;O4	C14H26NO7P	[M+Cl]-
386.1137595						
387.0977997	387.0981	0.000	0.7750	LPA 11:2;O	C14H25O8P	[M+Cl]-
	389.1138	0.000	1.0280	LPA 11:1;O	C14H27O8P	[M+Cl]-
389.1133448						
750.5320532	750.5291	0.003	3.9972	CerP 39:1;O6	C39H78NO10P	[M-H]-
	750.5291	0.003	3.9972	LPS 33:0;O	C39H78NO10P	[M-H]-
750.5320532						
750.5320532	750.5291	0.003	3.9972	PS O-33:0;O	C39H78NO10P	[M-H]-
	750.5292	0.003	3.7307	HexCer 34:1;O3	C40H77NO9	[M+Cl]-
750.5320532						
750.5320532	750.5291	0.003	3.9972	CerP 38:1;O4	C38H76NO8P	[M+Formate]-
	750.5291	0.003	3.9972	LPC 30:1;O	C38H76NO8P	[M+Formate]-

750.5320532						
750.5320532	750.5291	0.003	3.9972	LPE 33:1;O	C38H76NO8P	[M+Formate]-
	750.5291	0.003	3.9972	LPS O-32:1	C38H76NO8P	[M+Formate]-
750.5320532						
750.5320532	750.5291	0.003	3.9972	PC 30:0	C38H76NO8P	[M+Formate]-
	750.5291	0.003	3.9972	PC O-30:1;O	C38H76NO8P	[M+Formate]-
750.5320532						
750.5320532	750.5291	0.003	3.9972	PE 33:0	C38H76NO8P	[M+Formate]-
	750.5291	0.003	3.9972	PE O-33:1;O	C38H76NO8P	[M+Formate]-
750.5320532						
750.5320532	750.5291	0.003	3.9972	CerP 37:1;O4	C37H74NO8P	[M+OAc]-
	750.5291	0.003	3.9972	LPC 29:1;O	C37H74NO8P	[M+OAc]-
750.5320532						
750.5320532	750.5291	0.003	3.9972	LPE 32:1;O	C37H74NO8P	[M+OAc]-
	750.5291	0.003	3.9972	LPS O-31:1	C37H74NO8P	[M+OAc]-
750.5320532						
750.5320532	750.5291	0.003	3.9972	PC 29:0	C37H74NO8P	[M+OAc]-
	750.5291	0.003	3.9972	PC O-29:1;O	C37H74NO8P	[M+OAc]-
750.5320532						
750.5320532	750.5291	0.003	3.9972	PE 32:0	C37H74NO8P	[M+OAc]-
	750.5291	0.003	3.9972	PE O-32:1;O	C37H74NO8P	[M+OAc]-
750.5320532						
750.5682475	750.5655	0.003	3.7305	CerP 40:0;O5	C40H82NO9P	[M-H]-
	750.5655	0.003	3.7305	LPS O-34:0;O	C40H82NO9P	[M-H]-
750.5682475						
750.5682475	750.5656	0.003	3.4641	CAR 34:0;O4	C41H81NO8	[M+Cl]-
	750.5656	0.003	3.4641	HexCer 35:0;O2	C41H81NO8	[M+Cl]-
750.5682475						
750.5682475	750.5655	0.003	3.7305	CerP 39:0;O3	C39H80NO7P	[M+Formate]-
	750.5655	0.003	3.7305	LPC 31:0	C39H80NO7P	[M+Formate]-
750.5682475						
750.5682475	750.5655	0.003	3.7305	LPC O-31:1;O	C39H80NO7P	[M+Formate]-
	750.5655	0.003	3.7305	LPE 34:0	C39H80NO7P	[M+Formate]-
750.5682475						
750.5682475	750.5655	0.003	3.7305	LPE O-34:1;O	C39H80NO7P	[M+Formate]-
	750.5655	0.003	3.7305	PC O-31:0	C39H80NO7P	[M+Formate]-
750.5682475						
750.5682475	750.5655	0.003	3.7305	PE O-34:0	C39H80NO7P	[M+Formate]-

750.5682475	750.5655	0.003	3.7305	CerP 38:0;O3	C38H78NO7P	[M+OAc]-
750.5682475	750.5655	0.003	3.7305	LPC 30:0	C38H78NO7P	[M+OAc]-
750.5682475	750.5655	0.003	3.7305	LPC O-30:1;O	C38H78NO7P	[M+OAc]-
750.5682475	750.5655	0.003	3.7305	LPE 33:0	C38H78NO7P	[M+OAc]-
750.5682475	750.5655	0.003	3.7305	LPE O-33:1;O	C38H78NO7P	[M+OAc]-
750.5682475	750.5655	0.003	3.7305	PC O-30:0	C38H78NO7P	[M+OAc]-
750.5682475	750.5655	0.003	3.7305	PE O-33:0	C38H78NO7P	[M+OAc]-
751.2285955	751.2268	0.002	2.3961	PIP 20:3	C29H50O16P2	[M+Cl]-
751.5354638	751.5366	0.001	1.4637	MGDG 34:3	C43H76O10	[M-H]-
751.5354638	751.5366	0.001	1.4637	TG 39:3;O2	C42H74O8	[M+Formate]-
751.5354638	751.5366	0.001	1.4637	TG O-39:4;O3	C42H74O8	[M+Formate]-
751.5354638	751.5366	0.001	1.4637	TG 38:3;O2	C41H72O8	[M+OAc]-
751.5354638	751.5366	0.001	1.4637	TG O-38:4;O3	C41H72O8	[M+OAc]-
753.2447044	753.2425	0.002	2.9207	PIP 20:2	C29H52O16P2	[M+Cl]-
758.5023145	758.506	0.004	4.8780	HexCer 34:3;O6	C40H73NO12	[M-H]-
758.5023145	758.506	0.004	4.8780	HexCer 33:3;O4	C39H71NO10	[M+Formate]-
758.5023145	758.506	0.004	4.8780	HexCer 32:3;O4	C38H69NO10	[M+OAc]-
759.5040756	759.5053	0.001	1.5800	MGDG 35:6	C44H72O10	[M-H]-
759.5040756	759.5053	0.001	1.5800	TG 40:6;O2	C43H70O8	[M+Formate]-
759.5040756	759.5053	0.001	1.5800	TG O-40:7;O3	C43H70O8	[M+Formate]-
759.5040756	759.5053	0.001	1.5800	TG 39:6;O2	C42H68O8	[M+OAc]-
759.5040756	759.5053	0.001	1.5800	TG O-39:7;O3	C42H68O8	[M+OAc]-
760.5162567	760.5134	0.003	3.6817	CerP 40:3;O6	C40H76NO10P	[M-H]-
760.5162567	760.5134	0.003	3.6817	LPS 34:2;O	C40H76NO10P	[M-H]-
	760.5134	0.003	3.6817	PS 34:1	C40H76NO10P	[M-H]-

760.5162567						
760.5162567	760.5134	0.003	3.6817	PS O-34:2;O	C40H76NO10P	[M-H]-
	760.5136	0.003	3.5502	HexCer 35:3;O3	C41H75NO9	[M+Cl]-
760.5162567						
760.5162567	760.5134	0.003	3.6817	CerP 39:3;O4	C39H74NO8P	[M+Formate]-
	760.5134	0.003	3.6817	LPC 31:3;O	C39H74NO8P	[M+Formate]-
760.5162567						
760.5162567	760.5134	0.003	3.6817	LPE 34:3;O	C39H74NO8P	[M+Formate]-
	760.5134	0.003	3.6817	LPS O-33:3	C39H74NO8P	[M+Formate]-
760.5162567						
760.5162567	760.5134	0.003	3.6817	PC 31:2	C39H74NO8P	[M+Formate]-
	760.5134	0.003	3.6817	PC O-31:3;O	C39H74NO8P	[M+Formate]-
760.5162567						
760.5162567	760.5134	0.003	3.6817	PE 34:2	C39H74NO8P	[M+Formate]-
	760.5134	0.003	3.6817	PE O-34:3;O	C39H74NO8P	[M+Formate]-
760.5162567						
760.5162567	760.5134	0.003	3.6817	CerP 38:3;O4	C38H72NO8P	[M+OAc]-
	760.5134	0.003	3.6817	LPC 30:3;O	C38H72NO8P	[M+OAc]-
760.5162567						
760.5162567	760.5134	0.003	3.6817	LPE 33:3;O	C38H72NO8P	[M+OAc]-
	760.5134	0.003	3.6817	LPS O-32:3	C38H72NO8P	[M+OAc]-
760.5162567						
760.5162567	760.5134	0.003	3.6817	PC 30:2	C38H72NO8P	[M+OAc]-
	760.5134	0.003	3.6817	PC O-30:3;O	C38H72NO8P	[M+OAc]-
760.5162567						
760.5162567	760.5134	0.003	3.6817	PE 33:2	C38H72NO8P	[M+OAc]-
	760.5134	0.003	3.6817	PE O-33:3;O	C38H72NO8P	[M+OAc]-
760.5162567						
761.5191234	761.5209	0.002	2.3637	MGDG 35:5	C44H74O10	[M-H]-
	761.5209	0.002	2.3637	TG 40:5;O2	C43H72O8	[M+Formate]-
761.5191234						
761.5191234	761.5209	0.002	2.3637	TG O-40:6;O3	C43H72O8	[M+Formate]-
	761.5209	0.002	2.3637	TG 39:5;O2	C42H70O8	[M+OAc]-
761.5191234						
761.5191234	761.5209	0.002	2.3637	TG O-39:6;O3	C42H70O8	[M+OAc]-
	761.5209	0.000	0.5253	MGDG 35:5	C44H74O10	[M-H]-
761.5205545						
761.5205545	761.5209	0.000	0.5253	TG 40:5;O2	C43H72O8	[M+Formate]-

761.5205545	761.5209	0.000	0.5253	TG O-40:6;O3	C43H72O8	[M+Formate]-
761.5205545	761.5209	0.000	0.5253	TG 39:5;O2	C42H70O8	[M+OAc]-
761.5205545	761.5209	0.000	0.5253	TG O-39:6;O3	C42H70O8	[M+OAc]-
762.5226475	762.521	0.002	2.0983	CerP 41:3;O3	C41H78NO7P	[M+Cl]-
762.5226475	762.521	0.002	2.0983	LPC 33:3	C41H78NO7P	[M+Cl]-
762.5226475	762.521	0.002	2.0983	LPC O-33:4;O	C41H78NO7P	[M+Cl]-
762.5226475	762.521	0.002	2.0983	PC O-33:3	C41H78NO7P	[M+Cl]-
762.5226475	762.521	0.002	2.0983	PE O-36:3	C41H78NO7P	[M+Cl]-
765.2079256	765.2061	0.002	2.3523	PIP 20:4;O	C29H48O17P2	[M+Cl]-
767.2241784	767.2217	0.002	3.1282	PIP 20:3;O	C29H50O17P2	[M+Cl]-
769.2357726	769.2374	0.002	2.0800	PIP 20:2;O	C29H52O17P2	[M+Cl]-
769.2410584	769.2374	0.004	4.8100	PIP 20:2;O	C29H52O17P2	[M+Cl]-
776.5018524	776.5003	0.002	2.0605	CerP 41:4;O4	C41H76NO8P	[M+Cl]-
776.5018524	776.5003	0.002	2.0605	LPC 33:4;O	C41H76NO8P	[M+Cl]-
776.5018524	776.5003	0.002	2.0605	PC 33:3	C41H76NO8P	[M+Cl]-
776.5018524	776.5003	0.002	2.0605	PC O-33:4;O	C41H76NO8P	[M+Cl]-
776.5018524	776.5003	0.002	2.0605	PE 36:3	C41H76NO8P	[M+Cl]-
776.5018524	776.5003	0.002	2.0605	PE O-36:4;O	C41H76NO8P	[M+Cl]-
776.5115833	776.5083	0.003	4.2498	IPC 34:2;O2	C40H76NO11P	[M-H]-
776.5115833	776.5083	0.003	4.2498	PS 34:1;O	C40H76NO11P	[M-H]-
776.5115833	776.5085	0.003	3.9922	HexCer 35:3;O4	C41H75NO10	[M+Cl]-
776.5115833	776.5083	0.003	4.2498	CerP 39:3;O5	C39H74NO9P	[M+Formate]-
776.5115833	776.5083	0.003	4.2498	LPS 33:2	C39H74NO9P	[M+Formate]-
776.5115833	776.5083	0.003	4.2498	LPS O-33:3;O	C39H74NO9P	[M+Formate]-
	776.5083	0.003	4.2498	PC 31:2;O	C39H74NO9P	[M+Formate]-

776.5115833						
776.5115833	776.5083	0.003	4.2498	PE 34:2;O	C39H74NO9P	[M+Formate]-
	776.5083	0.003	4.2498	PS O-33:2	C39H74NO9P	[M+Formate]-
776.5115833						
776.5115833	776.5083	0.003	4.2498	CerP 38:3;O5	C38H72NO9P	[M+OAc]-
	776.5083	0.003	4.2498	LPS 32:2	C38H72NO9P	[M+OAc]-
776.5115833						
776.5115833	776.5083	0.003	4.2498	LPS O-32:3;O	C38H72NO9P	[M+OAc]-
	776.5083	0.003	4.2498	PC 30:2;O	C38H72NO9P	[M+OAc]-
776.5115833						
776.5115833	776.5083	0.003	4.2498	PE 33:2;O	C38H72NO9P	[M+OAc]-
	776.5083	0.003	4.2498	PS O-32:2	C38H72NO9P	[M+OAc]-
776.5115833						
776.5841924	776.5811	0.003	3.9919	CerP 42:1;O5	C42H84NO9P	[M-H]-
	776.5811	0.003	3.9919	PE 37:0;O	C42H84NO9P	[M-H]-
776.5841924						
776.5841924	776.5811	0.003	3.9919	PS O-36:0	C42H84NO9P	[M-H]-
	776.5813	0.003	3.7343	ACer 43:1;O6	C43H83NO8	[M+Cl]-
776.5841924						
776.5841924	776.5813	0.003	3.7343	HexCer 37:1;O2	C43H83NO8	[M+Cl]-
	776.5811	0.003	3.9919	CerP 41:1;O3	C41H82NO7P	[M+Formate]-
776.5841924						
776.5841924	776.5811	0.003	3.9919	LPC 33:1	C41H82NO7P	[M+Formate]-
	776.5811	0.003	3.9919	LPC O-33:2;O	C41H82NO7P	[M+Formate]-
776.5841924						
776.5841924	776.5811	0.003	3.9919	PC O-33:1	C41H82NO7P	[M+Formate]-
	776.5811	0.003	3.9919	PE O-36:1	C41H82NO7P	[M+Formate]-
776.5841924						
776.5841924	776.5811	0.003	3.9919	CerP 40:1;O3	C40H80NO7P	[M+OAc]-
	776.5811	0.003	3.9919	LPC 32:1	C40H80NO7P	[M+OAc]-
776.5841924						
776.5841924	776.5811	0.003	3.9919	LPC O-32:2;O	C40H80NO7P	[M+OAc]-
	776.5811	0.003	3.9919	PC O-32:1	C40H80NO7P	[M+OAc]-
776.5841924						
776.5841924	776.5811	0.003	3.9919	PE O-35:1	C40H80NO7P	[M+OAc]-
	776.5811	0.003	3.9919	PC 35:0;O	C43H86NO9P	[M-CH3]-
776.5841924						
777.5055538	777.5076	0.002	2.5723	PA 41:6;O	C44H75O9P	[M-H]-

	777.5076	0.002	2.5723	PG O-38:7	C44H75O9P	[M-H]-
777.5055538						
777.5055538	777.5078	0.002	2.8296	TG 42:6;O2	C45H74O8	[M+Cl]-
	777.5078	0.002	2.8296	TG O-42:7;O3	C45H74O8	[M+Cl]-
777.5055538						
777.5055538	777.5036	0.002	2.5723	EPC 36:3;O5	C38H73N2O9P	[M+Formate]-
	777.5036	0.002	2.5723	SM 33:3;O5	C38H73N2O9P	[M+Formate]-
777.5055538						
777.5055538	777.5076	0.002	2.5723	PA O-40:7	C43H73O7P	[M+Formate]-
	777.5036	0.002	2.5723	EPC 35:3;O5	C37H71N2O9P	[M+OAc]-
777.5055538						
777.5055538	777.5036	0.002	2.5723	SM 32:3;O5	C37H71N2O9P	[M+OAc]-
	777.5076	0.002	2.5723	PA O-39:7	C42H71O7P	[M+OAc]-
777.5055538						
778.5175038	778.5159	0.002	2.0552	CerP 41:3;O4	C41H78NO8P	[M+Cl]-
	778.5159	0.002	2.0552	LPC 33:3;O	C41H78NO8P	[M+Cl]-
778.5175038						
778.5175038	778.5159	0.002	2.0552	PC 33:2	C41H78NO8P	[M+Cl]-
	778.5159	0.002	2.0552	PC O-33:3;O	C41H78NO8P	[M+Cl]-
778.5175038						
778.5175038	778.5159	0.002	2.0552	PE 36:2	C41H78NO8P	[M+Cl]-
	778.5159	0.002	2.0552	PE O-36:3;O	C41H78NO8P	[M+Cl]-
778.5175038						
782.4983903	782.4978	0.001	0.7668	CerP 42:6;O6	C42H74NO10P	[M-H]-
	782.4978	0.001	0.7668	PS 36:4	C42H74NO10P	[M-H]-
782.4983903						
782.4983903	782.4978	0.001	0.7668	PS O-36:5;O	C42H74NO10P	[M-H]-
	782.4979	0.000	0.5112	HexCer 37:6;O3	C43H73NO9	[M+Cl]-
782.4983903						
782.4983903	782.4978	0.001	0.7668	CerP 41:6;O4	C41H72NO8P	[M+Formate]-
	782.4978	0.001	0.7668	LPC 33:6;O	C41H72NO8P	[M+Formate]-
782.4983903						
782.4983903	782.4978	0.001	0.7668	PC 33:5	C41H72NO8P	[M+Formate]-
	782.4978	0.001	0.7668	PC O-33:6;O	C41H72NO8P	[M+Formate]-
782.4983903						
782.4983903	782.4978	0.001	0.7668	PE 36:5	C41H72NO8P	[M+Formate]-
	782.4978	0.001	0.7668	PE O-36:6;O	C41H72NO8P	[M+Formate]-

782.4983903						
782.4983903	782.4978	0.001	0.7668	CerP 40:6;O4	C40H70NO8P	[M+OAc]-
	782.4978	0.001	0.7668	LPC 32:6;O	C40H70NO8P	[M+OAc]-
782.4983903						
782.4983903	782.4978	0.001	0.7668	LPS O-34:6	C40H70NO8P	[M+OAc]-
	782.4978	0.001	0.7668	PC 32:5	C40H70NO8P	[M+OAc]-
782.4983903						
782.4983903	782.4978	0.001	0.7668	PC O-32:6;O	C40H70NO8P	[M+OAc]-
	782.4978	0.001	0.7668	PE 35:5	C40H70NO8P	[M+OAc]-
782.4983903						
782.4983903	782.4978	0.001	0.7668	PE O-35:6;O	C40H70NO8P	[M+OAc]-
	782.4978	0.003	3.8339	CerP 42:6;O6	C42H74NO10P	[M-H]-
782.5007749						
782.5007749	782.4978	0.003	3.8339	PS 36:4	C42H74NO10P	[M-H]-
	782.4978	0.003	3.8339	PS O-36:5;O	C42H74NO10P	[M-H]-
782.5007749						
782.5007749	782.4979	0.003	3.5783	HexCer 37:6;O3	C43H73NO9	[M+Cl]-
	782.4978	0.003	3.8339	CerP 41:6;O4	C41H72NO8P	[M+Formate]-
782.5007749						
782.5007749	782.4978	0.003	3.8339	LPC 33:6;O	C41H72NO8P	[M+Formate]-
	782.4978	0.003	3.8339	PC 33:5	C41H72NO8P	[M+Formate]-
782.5007749						
782.5007749	782.4978	0.003	3.8339	PC O-33:6;O	C41H72NO8P	[M+Formate]-
	782.4978	0.003	3.8339	PE 36:5	C41H72NO8P	[M+Formate]-
782.5007749						
782.5007749	782.4978	0.003	3.8339	PE O-36:6;O	C41H72NO8P	[M+Formate]-
	782.4978	0.003	3.8339	CerP 40:6;O4	C40H70NO8P	[M+OAc]-
782.5007749						
782.5007749	782.4978	0.003	3.8339	LPC 32:6;O	C40H70NO8P	[M+OAc]-
	782.4978	0.003	3.8339	LPS O-34:6	C40H70NO8P	[M+OAc]-
782.5007749						
782.5007749	782.4978	0.003	3.8339	PC 32:5	C40H70NO8P	[M+OAc]-
	782.4978	0.003	3.8339	PC O-32:6;O	C40H70NO8P	[M+OAc]-
782.5007749						
782.5007749	782.4978	0.003	3.8339	PE 35:5	C40H70NO8P	[M+OAc]-
	782.4978	0.003	3.8339	PE O-35:6;O	C40H70NO8P	[M+OAc]-
782.5007749						
784.3939576	784.3962	0.002	2.8047	PS 34:7	C40H64NO10P	[M+Cl]-

784.3939576	784.3962	0.002	2.8047	PS O-34:8;O	C40H64NO10P	[M+Cl]-
786.4088395	786.4104	0.002	2.0346	SHexCer 33:6;O4	C39H65NO13S	[M-H]-
786.4088395	786.4104	0.002	2.0346	SHexCer 32:6;O2	C38H63NO11S	[M+Formate]-
786.4088395	786.4104	0.002	2.0346	SHexCer 31:6;O2	C37H61NO11S	[M+OAc]-
786.4108803	786.4104	0.001	0.6358	SHexCer 33:6;O4	C39H65NO13S	[M-H]-
786.4108803	786.4118	0.001	1.2716	LPS 34:7;O	C40H66NO10P	[M+Cl]-
786.4108803	786.4118	0.001	1.2716	PS 34:6	C40H66NO10P	[M+Cl]-
786.4108803	786.4118	0.001	1.2716	PS O-34:7;O	C40H66NO10P	[M+Cl]-
786.4108803	786.4104	0.001	0.6358	SHexCer 32:6;O2	C38H63NO11S	[M+Formate]-
786.4108803	786.4104	0.001	0.6358	SHexCer 31:6;O2	C37H61NO11S	[M+OAc]-
787.2383530	787.2349	0.004	4.4459	PIP 21:5;O	C30H48O17P2	[M+Formate]-
787.2383530	787.2349	0.004	4.4459	PIP 20:5;O	C29H46O17P2	[M+OAc]-
803.1861188	803.1852	0.001	1.1205	PIP2 21:6	C30H47O19P3	[M-H]-
807.2178212	807.2165	0.001	1.6105	PIP2 21:4	C30H51O19P3	[M-H]-
807.5064127	807.5053	0.001	1.3622	MGDG 39:10	C48H72O10	[M-H]-
807.5064127	807.5061	0.000	0.3715	EPC 39:4;O5	C41H77N2O9P	[M+Cl]-
807.5064127	807.5061	0.000	0.3715	SM 36:4;O5	C41H77N2O9P	[M+Cl]-
807.5064127	807.5053	0.001	1.3622	TG 44:10;O2	C47H70O8	[M+Formate]-
807.5064127	807.5053	0.001	1.3622	TG O-44:11;O3	C47H70O8	[M+Formate]-
807.5064127	807.5053	0.001	1.3622	TG 43:10;O2	C46H68O8	[M+OAc]-
807.5064127	807.5053	0.001	1.3622	TG O-43:11;O3	C46H68O8	[M+OAc]-
817.2366444	817.2374	0.001	0.8565	PIP 24:6;O	C33H52O17P2	[M+Cl]-
820.5752712	820.5792	0.004	4.7527	Hex2Cer 31:0;O2	C43H83NO13	[M-H]-
820.5752712	820.5792	0.004	4.7527	HexCer 36:1;O5	C42H81NO11	[M+Formate]-
	820.5792	0.004	4.7527	HexCer 35:1;O5	C41H79NO11	[M+OAc]-

820.5752712						
821.5584703	821.5573	0.001	1.4606	TG 47:9;O3	C50H78O9	[M-H]-
	821.5581	0.000	0.4869	EPC 41:3;O4	C43H83N2O8P	[M+Cl]-
821.5584703						
821.5584703	821.5581	0.000	0.4869	SM 38:3;O4	C43H83N2O8P	[M+Cl]-
	821.5573	0.001	1.4606	DG 46:10;O2	C49H76O7	[M+Formate]-
821.5584703						
821.5584703	821.5573	0.001	1.4606	TG 46:9;O	C49H76O7	[M+Formate]-
	821.5573	0.001	1.4606	TG O-46:10;O2	C49H76O7	[M+Formate]-
821.5584703						
821.5584703	821.5573	0.001	1.4606	DG 45:10;O2	C48H74O7	[M+OAc]-
	821.5573	0.001	1.4606	TG 45:9;O	C48H74O7	[M+OAc]-
821.5584703						
821.5584703	821.5573	0.001	1.4606	TG O-45:10;O2	C48H74O7	[M+OAc]-
	827.2581	0.000	0.0000	PIP 26:7	C35H54O16P2	[M+Cl]-
827.2581648						
827.2618831	827.2581	0.004	4.5935	PIP 26:7	C35H54O16P2	[M+Cl]-
	829.2738	0.003	3.1353	PIP 26:6	C35H56O16P2	[M+Cl]-
829.2763482						
835.5379368	835.5366	0.001	1.6756	MGDG 41:10	C50H76O10	[M-H]-
	835.5374	0.001	0.7181	EPC 41:4;O5	C43H81N2O9P	[M+Cl]-
835.5379368						
835.5379368	835.5374	0.001	0.7181	SM 38:4;O5	C43H81N2O9P	[M+Cl]-
	835.5366	0.001	1.6756	TG 46:10;O2	C49H74O8	[M+Formate]-
835.5379368						
835.5379368	835.5366	0.001	1.6756	TG O-46:11;O3	C49H74O8	[M+Formate]-
	835.5366	0.001	1.6756	TG 45:10;O2	C48H72O8	[M+OAc]-
835.5379368						
835.5379368	835.5366	0.001	1.6756	TG O-45:11;O3	C48H72O8	[M+OAc]-
	835.5414	0.001	0.7181	PA O-45:8	C48H81O7P	[M+Cl]-
835.5408069						
836.5390702	836.5377	0.001	1.6736	Hex2Cer 30:1;O4	C42H79NO15	[M-H]-
	836.5377	0.001	1.6736	Hex2Cer 29:1;O2	C41H77NO13	[M+Formate]-
836.5390702						
836.5390702	836.5377	0.001	1.6736	Hex2Cer 28:1;O2	C40H75NO13	[M+OAc]-
	836.5377	0.003	4.0644	Hex2Cer 30:1;O4	C42H79NO15	[M-H]-
836.5411089						
836.5411089	836.5447	0.004	4.3034	PS 40:5	C46H80NO10P	[M-H]-

	836.5447	0.004	4.3034	PS O-40:6;O	C46H80NO10P	[M-H]-
836.5411089						
836.5411089	836.5377	0.003	4.0644	Hex2Cer 29:1;O2	C41H77NO13	[M+Formate]-
	836.5447	0.004	4.3034	PC 37:6	C45H78NO8P	[M+Formate]-
836.5411089						
836.5411089	836.5447	0.004	4.3034	PC O-37:7;O	C45H78NO8P	[M+Formate]-
	836.5447	0.004	4.3034	PE 40:6	C45H78NO8P	[M+Formate]-
836.5411089						
836.5411089	836.5447	0.004	4.3034	PE O-40:7;O	C45H78NO8P	[M+Formate]-
	836.5377	0.003	4.0644	Hex2Cer 28:1;O2	C40H75NO13	[M+OAc]-
836.5411089						
836.5411089	836.5447	0.004	4.3034	PC 36:6	C44H76NO8P	[M+OAc]-
	836.5447	0.004	4.3034	PC O-36:7;O	C44H76NO8P	[M+OAc]-
836.5411089						
836.5411089	836.5447	0.004	4.3034	PE 39:6	C44H76NO8P	[M+OAc]-
	836.5447	0.004	4.3034	PE O-39:7;O	C44H76NO8P	[M+OAc]-
836.5411089						
839.2456822	839.2427	0.003	3.5747	PIP2 22:3;O	C31H55O20P3	[M-H]-
	843.253	0.003	3.0833	PIP 26:7;O	C35H54O17P2	[M+Cl]-
843.2556414						
845.2346068	845.2321	0.003	2.9578	PIP2 24:6	C33H53O19P3	[M-H]-
	845.2687	0.001	1.6563	PIP 26:6;O	C35H56O17P2	[M+Cl]-
845.2672956						
845.2716766	845.2687	0.003	3.5492	PIP 26:6;O	C35H56O17P2	[M+Cl]-
	847.2843	0.002	2.8326	PIP 26:5;O	C35H58O17P2	[M+Cl]-
847.2867868						
849.5891865	849.5886	0.001	0.7062	TG 49:9;O3	C52H82O9	[M-H]-
	849.5894	0.000	0.2354	EPC 43:3;O4	C45H87N2O8P	[M+Cl]-
849.5891865						
849.5891865	849.5894	0.000	0.2354	SM 40:3;O4	C45H87N2O8P	[M+Cl]-
	849.5886	0.001	0.7062	DG 48:10;O2	C51H80O7	[M+Formate]-
849.5891865						
849.5891865	849.5886	0.001	0.7062	TG 48:9;O	C51H80O7	[M+Formate]-
	849.5886	0.001	0.7062	TG O-48:10;O2	C51H80O7	[M+Formate]-
849.5891865						
849.5891865	849.5886	0.001	0.7062	DG 47:10;O2	C50H78O7	[M+OAc]-
	849.5886	0.001	0.7062	TG 47:9;O	C50H78O7	[M+OAc]-

849.5891865						
849.5891865	849.5886	0.001	0.7062	TG O-47:10;O2	C50H78O7	[M+OAc]-
	849.5886	0.002	2.5895	TG 49:9;O3	C52H82O9	[M-H]-
849.5907927						
849.5907927	849.5894	0.001	1.6479	EPC 43:3;O4	C45H87N2O8P	[M+Cl]-
	849.5894	0.001	1.6479	SM 40:3;O4	C45H87N2O8P	[M+Cl]-
849.5907927						
849.5907927	849.5886	0.002	2.5895	DG 48:10;O2	C51H80O7	[M+Formate]-
	849.5886	0.002	2.5895	TG 48:9;O	C51H80O7	[M+Formate]-
849.5907927						
849.5907927	849.5886	0.002	2.5895	TG O-48:10;O2	C51H80O7	[M+Formate]-
	849.5886	0.002	2.5895	DG 47:10;O2	C50H78O7	[M+OAc]-
849.5907927						
849.5907927	849.5886	0.002	2.5895	TG 47:9;O	C50H78O7	[M+OAc]-
	849.5886	0.002	2.5895	TG O-47:10;O2	C50H78O7	[M+OAc]-
849.5907927						
851.5326798	851.5323	0.000	0.4697	EPC 41:4;O6	C43H81N2O10P	[M+Cl]-
	851.5323	0.000	0.4697	SM 38:4;O6	C43H81N2O10P	[M+Cl]-
851.5326798						
851.5326798	851.5315	0.001	1.4092	TG 46:10;O3	C49H74O9	[M+Formate]-
	851.5315	0.001	1.4092	TG 45:10;O3	C48H72O9	[M+OAc]-
851.5326798						
857.2349515	857.2321	0.003	3.2663	PIP2 25:7	C34H53O19P3	[M-H]-
	857.2533	0.003	3.4995	PIP2 21:2	C30H55O19P3	[M+Formate]-
857.2562470						
857.2562470	857.2533	0.003	3.4995	PIP2 20:2	C29H53O19P3	[M+OAc]-
	859.2478	0.001	1.2802	PIP2 25:6	C34H55O19P3	[M-H]-
859.2488534						
859.2511422	859.2478	0.003	3.9569	PIP2 25:6	C34H55O19P3	[M-H]-
	859.2689	0.002	2.2112	PIP2 21:1	C30H57O19P3	[M+Formate]-
859.2670363						
859.2670363	859.2689	0.002	2.2112	PIP2 20:1	C29H55O19P3	[M+OAc]-
	859.2689	0.003	3.4913	PIP2 21:1	C30H57O19P3	[M+Formate]-
859.2718658						
859.2718658	859.2689	0.003	3.4913	PIP2 20:1	C29H55O19P3	[M+OAc]-
	861.2634	0.003	3.5994	PIP2 25:5	C34H57O19P3	[M-H]-
861.2665703						
862.6579345	862.6543	0.004	4.2891	CerP 47:1;O6	C47H94NO10P	[M-H]-

	862.6543	0.004	4.2891	PS O-41:0;O	C47H94NO10P	[M-H]-
862.6579345						
862.6579345	862.6544	0.004	4.0572	HexCer 42:1;O3	C48H93NO9	[M+Cl]-
	862.6543	0.004	4.2891	CerP 46:1;O4	C46H92NO8P	[M+Formate]-
862.6579345						
862.6579345	862.6543	0.004	4.2891	PC 38:0	C46H92NO8P	[M+Formate]-
	862.6543	0.004	4.2891	PC O-38:1;O	C46H92NO8P	[M+Formate]-
862.6579345						
862.6579345	862.6543	0.004	4.2891	PE 41:0	C46H92NO8P	[M+Formate]-
	862.6543	0.004	4.2891	PE O-41:1;O	C46H92NO8P	[M+Formate]-
862.6579345						
862.6579345	862.6543	0.004	4.2891	CerP 45:1;O4	C45H90NO8P	[M+OAc]-
	862.6543	0.004	4.2891	PC 37:0	C45H90NO8P	[M+OAc]-
862.6579345						
862.6579345	862.6543	0.004	4.2891	PC O-37:1;O	C45H90NO8P	[M+OAc]-
	862.6543	0.004	4.2891	PE 40:0	C45H90NO8P	[M+OAc]-
862.6579345						
862.6579345	862.6543	0.004	4.2891	PE O-40:1;O	C45H90NO8P	[M+OAc]-
	862.6625	0.002	2.3184	HexCer 41:0;O6	C47H93NO12	[M-H]-
862.6604643						
862.6604643	862.6625	0.002	2.3184	HexCer 40:0;O4	C46H91NO10	[M+Formate]-
	862.6625	0.002	2.3184	HexCer 39:0;O4	C45H89NO10	[M+OAc]-
862.6604643						
863.2824248	863.2791	0.003	3.8226	PIP2 25:4	C34H59O19P3	[M-H]-
	863.5679	0.001	1.3896	MGDG 43:10	C52H80O10	[M-H]-
863.5690324						
863.5690324	863.5687	0.000	0.4632	EPC 43:4;O5	C45H85N2O9P	[M+Cl]-
	863.5687	0.000	0.4632	SM 40:4;O5	C45H85N2O9P	[M+Cl]-
863.5690324						
863.5690324	863.5679	0.001	1.3896	TG 48:10;O2	C51H78O8	[M+Formate]-
	863.5679	0.001	1.3896	TG O-48:11;O3	C51H78O8	[M+Formate]-
863.5690324						
863.5690324	863.5679	0.001	1.3896	TG 47:10;O2	C50H76O8	[M+OAc]-
	863.5679	0.001	1.3896	TG O-47:11;O3	C50H76O8	[M+OAc]-
863.5690324						
863.5704866	863.5679	0.003	3.0108	MGDG 43:10	C52H80O10	[M-H]-
	863.5687	0.002	2.0844	EPC 43:4;O5	C45H85N2O9P	[M+Cl]-

863.5704866						
863.5704866	863.5687	0.002	2.0844	SM 40:4;O5	C45H85N2O9P	[M+Cl]-
	863.5727	0.002	2.5476	PA O-47:8	C50H85O7P	[M+Cl]-
863.5704866						
863.5704866	863.5679	0.003	3.0108	TG 48:10;O2	C51H78O8	[M+Formate]-
	863.5679	0.003	3.0108	TG O-48:11;O3	C51H78O8	[M+Formate]-
863.5704866						
863.5704866	863.5679	0.003	3.0108	TG 47:10;O2	C50H76O8	[M+OAc]-
	863.5679	0.003	3.0108	TG O-47:11;O3	C50H76O8	[M+OAc]-
863.5704866						
864.5725694	864.576	0.003	3.9326	PS 42:5	C48H84NO10P	[M-H]-
	864.576	0.003	3.9326	PS O-42:6;O	C48H84NO10P	[M-H]-
864.5725694						
864.5725694	864.569	0.004	4.1639	Hex2Cer 32:1;O4	C44H83NO15	[M-H]-
	864.576	0.003	3.9326	PC 39:6	C47H82NO8P	[M+Formate]-
864.5725694						
864.5725694	864.576	0.003	3.9326	PC O-39:7;O	C47H82NO8P	[M+Formate]-
	864.576	0.003	3.9326	PE 42:6	C47H82NO8P	[M+Formate]-
864.5725694						
864.5725694	864.576	0.003	3.9326	PE O-42:7;O	C47H82NO8P	[M+Formate]-
	864.569	0.004	4.1639	Hex2Cer 31:1;O2	C43H81NO13	[M+Formate]-
864.5725694						
864.5725694	864.576	0.003	3.9326	PC 38:6	C46H80NO8P	[M+OAc]-
	864.576	0.003	3.9326	PC O-38:7;O	C46H80NO8P	[M+OAc]-
864.5725694						
864.5725694	864.576	0.003	3.9326	PE 41:6	C46H80NO8P	[M+OAc]-
	864.576	0.003	3.9326	PE O-41:7;O	C46H80NO8P	[M+OAc]-
864.5725694						
864.5725694	864.569	0.004	4.1639	Hex2Cer 30:1;O2	C42H79NO13	[M+OAc]-
	867.5264	0.001	1.2680	MGDG 40:10	C49H74O10	[M+Formate]-
867.5274926						
867.5274926	867.5264	0.001	1.2680	MGDG 39:10	C48H72O10	[M+OAc]-
	871.2325	0.003	3.7877	PIP2 21:3;O	C30H53O20P3	[M+Formate]-
871.2358542						
871.2358542	871.2325	0.003	3.7877	PIP2 20:3;O	C29H51O20P3	[M+OAc]-
	873.227	0.001	0.8016	PIP2 25:7;O	C34H53O20P3	[M-H]-
873.2276942						
873.2469066	873.2482	0.001	1.4887	PIP2 21:2;O	C30H55O20P3	[M+Formate]-

	873.2482	0.001	1.4887	PIP2 20:2;O	C29H53O20P3	[M+OAc]-
873.2469066						
873.2519658	873.2482	0.004	4.3516	PIP2 21:2;O	C30H55O20P3	[M+Formate]-
	873.2482	0.004	4.3516	PIP2 20:2;O	C29H53O20P3	[M+OAc]-
873.2519658						
875.2458557	875.2427	0.003	3.6561	PIP2 25:6;O	C34H55O20P3	[M-H]-
	875.2638	0.003	2.8563	PIP2 21:1;O	C30H57O20P3	[M+Formate]-
875.2663484						
875.2663484	875.2638	0.003	2.8563	PIP2 20:1;O	C29H55O20P3	[M+OAc]-
	877.2795	0.002	2.7357	PIP2 21:0;O	C30H59O20P3	[M+Formate]-
877.2770265						
877.2770265	877.2795	0.002	2.7357	PIP2 20:0;O	C29H57O20P3	[M+OAc]-
	877.2795	0.002	2.6217	PIP2 21:0;O	C30H59O20P3	[M+Formate]-
877.2817966						
877.2817966	877.2795	0.002	2.6217	PIP2 20:0;O	C29H57O20P3	[M+OAc]-
	879.274	0.003	3.2982	PIP2 25:4;O	C34H59O20P3	[M-H]-
879.2768796						
879.5640080	879.5636	0.000	0.4548	EPC 43:4;O6	C45H85N2O10P	[M+Cl]-
	879.5636	0.000	0.4548	SM 40:4;O6	C45H85N2O10P	[M+Cl]-
879.5640080						
879.5640080	879.5628	0.001	1.3643	TG 48:10;O3	C51H78O9	[M+Formate]-
	879.5628	0.001	1.3643	TG 47:10;O3	C50H76O9	[M+OAc]-
879.5640080						
880.5674024	880.5639	0.004	3.9747	Hex2Cer 32:1;O5	C44H83NO16	[M-H]-
	880.5709	0.004	3.9747	IPC 42:6;O2	C48H84NO11P	[M-H]-
880.5674024						
880.5674024	880.5709	0.004	3.9747	PS 42:5;O	C48H84NO11P	[M-H]-
	880.5639	0.004	3.9747	Hex2Cer 31:1;O3	C43H81NO14	[M+Formate]-
880.5674024						
880.5674024	880.5709	0.004	3.9747	PC 39:6;O	C47H82NO9P	[M+Formate]-
	880.5709	0.004	3.9747	PE 42:6;O	C47H82NO9P	[M+Formate]-
880.5674024						
880.5674024	880.5709	0.004	3.9747	PS O-41:6	C47H82NO9P	[M+Formate]-
	880.5639	0.004	3.9747	Hex2Cer 30:1;O3	C42H79NO14	[M+OAc]-
880.5674024						
880.5674024	880.5709	0.004	3.9747	PC 38:6;O	C46H80NO9P	[M+OAc]-
	880.5709	0.004	3.9747	PE 41:6;O	C46H80NO9P	[M+OAc]-

880.5674024						
880.5674024	880.5709	0.004	3.9747	PS O-40:6	C46H80NO9P	[M+OAc]-
	887.2558	0.001	1.5779	PIP2 24:3	C33H59O19P3	[M+Cl]-
887.2571895						
889.2731724	889.2714	0.002	2.0241	PIP2 24:2	C33H61O19P3	[M+Cl]-
	891.5968	0.000	0.4486	PI 38:1	C47H89O13P	[M-H]-
891.5972493						
891.5972493	891.5968	0.000	0.4486	PI O-38:2;O	C47H89O13P	[M-H]-
	891.5968	0.000	0.4486	PG 40:2;O	C46H87O11P	[M+Formate]-
891.5972493						
891.5972493	891.5968	0.000	0.4486	PG 39:2;O	C45H85O11P	[M+OAc]-
	893.2533	0.003	3.4705	PIP2 24:5	C33H55O19P3	[M+Formate]-
893.2564042						
893.2564042	893.2533	0.003	3.4705	PIP2 23:5	C32H53O19P3	[M+OAc]-
	896.5447	0.000	0.1115	PS 45:10	C51H80NO10P	[M-H]-
896.5447738						
896.5447738	896.5447	0.000	0.1115	PS O-45:11;O	C51H80NO10P	[M-H]-
	896.5447	0.000	0.1115	PC 42:11	C50H78NO8P	[M+Formate]-
896.5447738						
896.5447738	896.5447	0.000	0.1115	PC O-42:12;O	C50H78NO8P	[M+Formate]-
	896.5447	0.000	0.1115	PE 45:11	C50H78NO8P	[M+Formate]-
896.5447738						
896.5447738	896.5447	0.000	0.1115	PE O-45:12;O	C50H78NO8P	[M+Formate]-
	896.5447	0.000	0.1115	PC 41:11	C49H76NO8P	[M+OAc]-
896.5447738						
896.5447738	896.5447	0.000	0.1115	PE 44:11	C49H76NO8P	[M+OAc]-
	896.5447	0.000	0.1115	PE O-44:12;O	C49H76NO8P	[M+OAc]-
896.5447738						
901.2375122	901.235	0.003	2.7740	PIP2 24:4;O	C33H57O20P3	[M+Cl]-
	903.2507	0.003	2.9892	PIP2 24:3;O	C33H59O20P3	[M+Cl]-
903.2533383						
904.6227950	904.6226	0.000	0.2211	PE O-50:12	C55H88NO7P	[M-H]-
	904.6226	0.000	0.2211	PC O-48:12	C56H90NO7P	[M-CH3]-
904.6227950						
905.2569179	905.2533	0.004	4.0873	PIP2 25:6	C34H55O19P3	[M+Formate]-
	905.2533	0.004	4.0873	PIP2 24:6	C33H53O19P3	[M+OAc]-
905.2569179						
905.2678837	905.2663	0.002	1.7674	PIP2 24:2;O	C33H61O20P3	[M+Cl]-

	915.2141	0.003	3.6057	PIP3 23:4	C32H56O22P4	[M-H]-
915.2174523						
917.2328197	917.2298	0.003	3.3797	PIP3 23:3	C32H58O22P4	[M-H]-
	919.2454	0.002	2.5021	PIP3 23:2	C32H60O22P4	[M-H]-
919.2477316						
921.2635104	921.2611	0.002	2.6051	PIP3 23:1	C32H62O22P4	[M-H]-
	923.2638	0.005	4.8740	PIP2 25:5;O	C34H57O20P3	[M+Formate]-
923.2682815						
923.2682815	923.2638	0.005	4.8740	PIP2 24:5;O	C33H55O20P3	[M+OAc]-
	923.2767	0.004	4.5490	PIP3 23:0	C32H64O22P4	[M-H]-
923.2725300						
939.6630200	939.6639	0.001	0.9578	DG 58:14	C61H92O5	[M+Cl]-
	939.6639	0.001	0.9578	TG O-58:14	C61H92O5	[M+Cl]-
939.6630200						
939.6630200	939.6615	0.002	1.5963	PA 51:5	C54H97O8P	[M+Cl]-
	939.6615	0.002	1.5963	PA O-51:6;O	C54H97O8P	[M+Cl]-
939.6630200						
940.6660492	940.6648	0.001	1.2757	IPC 46:4;O2	C52H96NO11P	[M-H]-
	940.6648	0.001	1.2757	PS 46:3;O	C52H96NO11P	[M-H]-
940.6660492						
940.6660492	940.665	0.001	1.0631	HexCer 47:5;O4	C53H95NO10	[M+Cl]-
	940.6648	0.001	1.2757	CerP 51:5;O5	C51H94NO9P	[M+Formate]-
940.6660492						
940.6660492	940.6648	0.001	1.2757	PC 43:4;O	C51H94NO9P	[M+Formate]-
	940.6648	0.001	1.2757	PE 46:4;O	C51H94NO9P	[M+Formate]-
940.6660492						
940.6660492	940.6648	0.001	1.2757	PS O-45:4	C51H94NO9P	[M+Formate]-
	940.6648	0.001	1.2757	CerP 50:5;O5	C50H92NO9P	[M+OAc]-
940.6660492						
940.6660492	940.6648	0.001	1.2757	PC 42:4;O	C50H92NO9P	[M+OAc]-
	940.6648	0.001	1.2757	PE 45:4;O	C50H92NO9P	[M+OAc]-
940.6660492						
940.6660492	940.6648	0.001	1.2757	PS O-44:4	C50H92NO9P	[M+OAc]-
	948.275	0.002	1.5818	CoA 12:0	C33H58N7O17P3S	[M-H]-
948.2765078						
948.6263565	948.6265	0.000	0.2108	Hex2Cer 37:2;O5	C49H91NO16	[M-H]-
	948.6255	0.001	0.9487	PC 46:8	C54H92NO8P	[M+Cl]-

948.6263565						
948.6263565	948.6255	0.001	0.9487	PC O-46:9;O	C54H92NO8P	[M+Cl]-
	948.6255	0.001	0.9487	PE 49:8	C54H92NO8P	[M+Cl]-
948.6263565						
948.6263565	948.6255	0.001	0.9487	PE O-49:9;O	C54H92NO8P	[M+Cl]-
	948.6265	0.000	0.2108	Hex2Cer 36:2;O3	C48H89NO14	[M+Formate]-
948.6263565						
948.6263565	948.6265	0.000	0.2108	Hex2Cer 35:2;O3	C47H87NO14	[M+OAc]-
	949.2795	0.004	4.1084	PIP2 27:6;O	C36H59O20P3	[M+Formate]-
949.2833380						
949.2833380	949.2795	0.004	4.1084	PIP2 26:6;O	C35H57O20P3	[M+OAc]-
	951.2716	0.004	4.2049	PIP3 24:1;O	C33H64O23P4	[M-H]-
951.2756182						
954.6624481	954.6652	0.003	2.9330	IPC 43:0;O5	C49H98NO14P	[M-H]-
	954.6594	0.003	3.2472	PE 51:9;O	C56H94NO9P	[M-H]-
954.6624481						
954.6624481	954.6594	0.003	3.2472	PS O-50:9	C56H94NO9P	[M-H]-
	954.6654	0.003	3.1425	Hex2Cer 38:0;O2	C50H97NO13	[M+Cl]-
954.6624481						
954.6624481	954.6652	0.003	2.9330	IPC 42:0;O3	C48H96NO12P	[M+Formate]-
	954.6594	0.003	3.2472	PC O-47:10	C55H92NO7P	[M+Formate]-
954.6624481						
954.6624481	954.6594	0.003	3.2472	PE O-50:10	C55H92NO7P	[M+Formate]-
	954.6652	0.003	2.9330	IPC 41:0;O3	C47H94NO12P	[M+OAc]-
954.6624481						
954.6624481	954.6594	0.003	3.2472	PC O-46:10	C54H90NO7P	[M+OAc]-
	954.6594	0.003	3.2472	PE O-49:10	C54H90NO7P	[M+OAc]-
954.6624481						
954.6624481	954.6594	0.003	3.2472	PC 49:9;O	C57H96NO9P	[M-CH3]-
	961.2714	0.004	3.9531	PIP2 30:8	C39H61O19P3	[M+Cl]-
961.2752266						
961.2752266	961.2795	0.004	4.3692	PIP2 28:7;O	C37H59O20P3	[M+Formate]-
	961.2795	0.004	4.3692	PIP2 27:7;O	C36H57O20P3	[M+OAc]-
961.2752266						
962.2547314	962.2543	0.001	0.5196	CoA 12:1;O	C33H56N7O18P3S	[M-H]-
	963.2871	0.001	0.7267	PIP2 30:7	C39H63O19P3	[M+Cl]-
963.2877856						
963.2921662	963.2951	0.003	3.1143	PIP2 28:6;O	C37H61O20P3	[M+Formate]-

	963.2951	0.003	3.1143	PIP2 27:6;O	C36H59O20P3	[M+OAc]-
963.2921662						
964.2725928	964.2699	0.003	2.8000	CoA 12:0;O	C33H58N7O18P3S	[M-H]-
	965.3027	0.002	1.6575	PIP2 30:6	C39H65O19P3	[M+Cl]-
965.3042625						
967.3174687	967.3184	0.001	0.9304	PIP2 30:5	C39H67O19P3	[M+Cl]-
	968.6386	0.001	0.8259	PS 50:9	C56H92NO10P	[M-H]-
968.6394251						
968.6394251	968.6386	0.001	0.8259	PS O-50:10;O	C56H92NO10P	[M-H]-
	968.6386	0.001	0.8259	PC 47:10	C55H90NO8P	[M+Formate]-
968.6394251						
968.6394251	968.6386	0.001	0.8259	PC O-47:11;O	C55H90NO8P	[M+Formate]-
	968.6386	0.001	0.8259	PE 50:10	C55H90NO8P	[M+Formate]-
968.6394251						
968.6394251	968.6386	0.001	0.8259	PE O-50:11;O	C55H90NO8P	[M+Formate]-
	968.6386	0.001	0.8259	PC 46:10	C54H88NO8P	[M+OAc]-
968.6394251						
968.6394251	968.6386	0.001	0.8259	PC O-46:11;O	C54H88NO8P	[M+OAc]-
	968.6386	0.001	0.8259	PE 49:10	C54H88NO8P	[M+OAc]-
968.6394251						
968.6394251	968.6386	0.001	0.8259	PE O-49:11;O	C54H88NO8P	[M+OAc]-
	968.6445	0.003	2.6842	IPC 43:1;O6	C49H96NO15P	[M-H]-
968.6418525						
968.6418525	968.6386	0.003	3.3036	PS 50:9	C56H92NO10P	[M-H]-
	968.6386	0.003	3.3036	PS O-50:10;O	C56H92NO10P	[M-H]-
968.6418525						
968.6418525	968.6447	0.003	2.8906	Hex2Cer 38:1;O3	C50H95NO14	[M+Cl]-
	968.6445	0.003	2.6842	IPC 42:1;O4	C48H94NO13P	[M+Formate]-
968.6418525						
968.6418525	968.6386	0.003	3.3036	PC 47:10	C55H90NO8P	[M+Formate]-
	968.6386	0.003	3.3036	PC O-47:11;O	C55H90NO8P	[M+Formate]-
968.6418525						
968.6418525	968.6386	0.003	3.3036	PE 50:10	C55H90NO8P	[M+Formate]-
	968.6386	0.003	3.3036	PE O-50:11;O	C55H90NO8P	[M+Formate]-
968.6418525						
968.6418525	968.6445	0.003	2.6842	IPC 41:1;O4	C47H92NO13P	[M+OAc]-
	968.6386	0.003	3.3036	PC 46:10	C54H88NO8P	[M+OAc]-

968.6418525						
968.6418525	968.6386	0.003	3.3036	PC O-46:11;O	C54H88NO8P	[M+OAc]-
	968.6386	0.003	3.3036	PE 49:10	C54H88NO8P	[M+OAc]-
968.6418525						
968.6418525	968.6386	0.003	3.3036	PE O-49:11;O	C54H88NO8P	[M+OAc]-
	969.6461	0.001	0.6188	TG 56:13;O2	C59H88O8	[M+Formate]-
969.6455393						
969.6455393	969.6461	0.001	0.6188	TG O-56:14;O3	C59H88O8	[M+Formate]-
	969.6461	0.001	0.6188	TG 55:13;O2	C58H86O8	[M+OAc]-
969.6455393						
969.6455393	969.6461	0.001	0.6188	TG O-55:14;O3	C58H86O8	[M+OAc]-
	971.5655	0.001	0.6176	PI 45:10	C54H85O13P	[M-H]-
971.5661533						
971.5661533	971.5655	0.001	0.6176	PI O-45:11;O	C54H85O13P	[M-H]-
	971.5655	0.001	0.6176	PG 47:11;O	C53H83O11P	[M+Formate]-
971.5661533						
971.5661533	971.5655	0.001	0.6176	PG 46:11;O	C52H81O11P	[M+OAc]-
	972.5724	0.001	1.1310	SHexCer 44:6;O6	C50H87NO15S	[M-H]-
972.5713017						
972.5713017	972.5724	0.001	1.1310	SHexCer 43:6;O4	C49H85NO13S	[M+Formate]-
	972.5724	0.001	1.1310	SHexCer 42:6;O4	C48H83NO13S	[M+OAc]-
972.5713017						
976.5910466	976.5899	0.001	1.2288	IPC 41:1;O6	C47H92NO15P	[M+Cl]-
	976.5921	0.001	1.0240	PS 46:8;O	C52H86NO11P	[M+Formate]-
976.5910466						
976.5910466	976.5921	0.001	1.0240	PS 45:8;O	C51H84NO11P	[M+OAc]-
	977.2663	0.001	1.0233	PIP2 30:8;O	C39H61O20P3	[M+Cl]-
977.2673545						
978.2536020	978.2492	0.004	4.4978	CoA 12:1;O2	C33H56N7O19P3S	[M-H]-
	978.2492	0.004	4.4978	CoA 11:1	C32H54N7O17P3S	[M+Formate]-
978.2536020						
978.2536020	978.2492	0.004	4.4978	CoA 10:1	C31H52N7O17P3S	[M+OAc]-
	979.282	0.002	1.8381	PIP2 30:7;O	C39H63O20P3	[M+Cl]-
979.2837779						
980.2665021	980.2648	0.002	1.7342	CoA 12:0;O2	C33H58N7O19P3S	[M-H]-
	980.2648	0.002	1.7342	CoA 11:0	C32H56N7O17P3S	[M+Formate]-
980.2665021						
980.2665021	980.2648	0.002	1.7342	CoA 10:0	C31H54N7O17P3S	[M+OAc]-

	981.2976	0.001	0.9172	PIP2 30:6;O	C39H65O20P3	[M+Cl]-
981.2985164						
981.3009928	981.2976	0.003	3.4648	PIP2 30:6;O	C39H65O20P3	[M+Cl]-
	982.6965	0.003	2.7475	IPC 45:0;O5	C51H102NO14P	[M-H]-
982.6937784						
982.6937784	982.6907	0.003	3.1546	PE 53:9;O	C58H98NO9P	[M-H]-
	982.6907	0.003	3.1546	PS O-52:9	C58H98NO9P	[M-H]-
982.6937784						
982.6937784	982.6967	0.003	2.9511	Hex2Cer 40:0;O2	C52H101NO13	[M+Cl]-
	982.6965	0.003	2.7475	IPC 44:0;O3	C50H100NO12P	[M+Formate]-
982.6937784						
982.6937784	982.6907	0.003	3.1546	PC O-49:10	C57H96NO7P	[M+Formate]-
	982.6907	0.003	3.1546	PE O-52:10	C57H96NO7P	[M+Formate]-
982.6937784						
982.6937784	982.6965	0.003	2.7475	IPC 43:0;O3	C49H98NO12P	[M+OAc]-
	982.6907	0.003	3.1546	PC O-48:10	C56H94NO7P	[M+OAc]-
982.6937784						
982.6937784	982.6907	0.003	3.1546	PE O-51:10	C56H94NO7P	[M+OAc]-
	982.6907	0.003	3.1546	PC 51:9;O	C59H100NO9P	[M-CH3]-
982.6937784						
983.3149517	983.3133	0.002	1.7288	PIP2 30:5;O	C39H67O20P3	[M+Cl]-
	993.2611	0.002	2.4163	PIP3 29:7	C38H62O22P4	[M-H]-
993.2634323						
994.6574714	994.6601	0.003	2.7145	IPC 45:2;O6	C51H98NO15P	[M-H]-
	994.6543	0.003	3.2172	PS 52:10	C58H94NO10P	[M-H]-
994.6574714						
994.6574714	994.6543	0.003	3.2172	PS O-52:11;O	C58H94NO10P	[M-H]-
	994.6603	0.003	2.8150	Hex2Cer 40:2;O3	C52H97NO14	[M+Cl]-
994.6574714						
994.6574714	994.6601	0.003	2.7145	IPC 44:2;O4	C50H96NO13P	[M+Formate]-
	994.6543	0.003	3.2172	PC 49:11	C57H92NO8P	[M+Formate]-
994.6574714						
994.6574714	994.6543	0.003	3.2172	PC O-49:12;O	C57H92NO8P	[M+Formate]-
	994.6543	0.003	3.2172	PE 52:11	C57H92NO8P	[M+Formate]-
994.6574714						
994.6574714	994.6543	0.003	3.2172	PE O-52:12;O	C57H92NO8P	[M+Formate]-
	994.6601	0.003	2.7145	IPC 43:2;O4	C49H94NO13P	[M+OAc]-

994.6574714						
994.6574714	994.6543	0.003	3.2172	PC 48:11	C56H90NO8P	[M+OAc]-
	994.6543	0.003	3.2172	PC O-48:12;O	C56H90NO8P	[M+OAc]-
994.6574714						
994.6574714	994.6543	0.003	3.2172	PE 51:11	C56H90NO8P	[M+OAc]-
	994.6543	0.003	3.2172	PE O-51:12;O	C56H90NO8P	[M+OAc]-
994.6574714						
995.2781907	995.2767	0.002	1.5071	PIP3 29:6	C38H64O22P4	[M-H]-
	995.2767	0.005	4.6218	PIP3 29:6	C38H64O22P4	[M-H]-
995.2812974						
996.6731947	996.6758	0.003	2.6087	IPC 45:1;O6	C51H100NO15P	[M-H]-
	996.6699	0.003	3.3110	PS 52:9	C58H96NO10P	[M-H]-
996.6731947						
996.6731947	996.6699	0.003	3.3110	PS O-52:10;O	C58H96NO10P	[M-H]-
	996.676	0.003	2.8093	Hex2Cer 40:1;O3	C52H99NO14	[M+Cl]-
996.6731947						
996.6731947	996.6758	0.003	2.6087	IPC 44:1;O4	C50H98NO13P	[M+Formate]-
	996.6699	0.003	3.3110	PC 49:10	C57H94NO8P	[M+Formate]-
996.6731947						
996.6731947	996.6699	0.003	3.3110	PC O-49:11;O	C57H94NO8P	[M+Formate]-
	996.6699	0.003	3.3110	PE 52:10	C57H94NO8P	[M+Formate]-
996.6731947						
996.6731947	996.6699	0.003	3.3110	PE O-52:11;O	C57H94NO8P	[M+Formate]-
	996.6758	0.003	2.6087	IPC 43:1;O4	C49H96NO13P	[M+OAc]-
996.6731947						
996.6731947	996.6699	0.003	3.3110	PC 48:10	C56H92NO8P	[M+OAc]-
	996.6699	0.003	3.3110	PC O-48:11;O	C56H92NO8P	[M+OAc]-
996.6731947						
996.6731947	996.6699	0.003	3.3110	PE 51:10	C56H92NO8P	[M+OAc]-
	996.6699	0.003	3.3110	PE O-51:11;O	C56H92NO8P	[M+OAc]-
996.6731947						
997.2942609	997.2924	0.002	1.9052	PIP3 29:5	C38H66O22P4	[M-H]-
	997.6774	0.001	0.7016	TG 58:13;O2	C61H92O8	[M+Formate]-
997.6767282						
997.6767282	997.6774	0.001	0.7016	TG O-58:14;O3	C61H92O8	[M+Formate]-
	997.6774	0.001	0.7016	TG 57:13;O2	C60H90O8	[M+OAc]-
997.6767282						
997.6767282	997.6774	0.001	0.7016	TG O-57:14;O3	C60H90O8	[M+OAc]-

	999.5968	0.000	0.1000	PI 47:10	C56H89O13P	[M-H]-
999.5966706						
999.5966706	999.5968	0.000	0.1000	PI O-47:11;O	C56H89O13P	[M-H]-
	999.5968	0.000	0.1000	PG 49:11;O	C55H87O11P	[M+Formate]-
999.5966706						
999.5966706	999.5968	0.000	0.1000	PG 48:11;O	C54H85O11P	[M+OAc]-
	393.1191	0.001	1.5263	ST 18:5;O7	C18H20O7	[M+Formate]-
393.1196945						
393.1207077	393.1191	0.002	4.0700	ST 18:5;O7	C18H20O7	[M+Formate]-
	397.1269	0.000	0.7554	LPA 11:2;O	C14H25O8P	[M+Formate]-
397.1266658						
397.1266658	397.1269	0.000	0.7554	LPA 10:2;O	C13H23O8P	[M+OAc]-
	404.1151	0.001	1.7322	NAT 12:1;O4	C14H27NO8S	[M+Cl]-
404.1158540						
404.1361927	404.1351	0.001	2.7219	ST 18:5;O7;G	C20H23NO8	[M-H]-
	405.1322	0.000	0.2468	ST 18:2;O8	C18H26O8	[M+Cl]-
405.1322352						
406.1312867	406.1308	0.001	1.2311	NAT 12:0;O4	C14H29NO8S	[M+Cl]-
	420.13	0.002	3.5703	ST 18:5;O8;G	C20H23NO9	[M-H]-
420.1315002						
420.1315002	420.1334	0.002	4.5224	NAT 14:4;O3	C16H25NO7S	[M+Formate]-
	420.2886	0.000	0.7138	CAR 15:0	C22H43NO4	[M+Cl]-
420.2883276						
420.2883276	420.2886	0.000	0.7138	NAE 20:1;O2	C22H43NO4	[M+Cl]-
	421.1269	0.000	0.7124	BMP 11:3	C17H27O10P	[M-H]-
421.1266074						
421.1417264	421.1423	0.001	1.4247	ST 22:6;O6	C22H26O6	[M+Cl]-
	423.1345	0.001	2.1270	LPA 15:4	C18H29O7P	[M+Cl]-
423.1335577						
423.1571942	423.158	0.001	1.8905	ST 22:5;O6	C22H28O6	[M+Cl]-
	424.1169	0.000	0.2358	ST 18:5;O6;G	C20H23NO7	[M+Cl]-
424.1169923						
425.1128593	425.1138	0.001	2.1171	LPA 14:4;O	C17H27O8P	[M+Cl]-
	425.1373	0.001	1.6465	ST 21:5;O7	C21H26O7	[M+Cl]-
425.1365498						
425.1462343	425.1453	0.001	2.1169	ST 19:4;O8	C19H24O8	[M+Formate]-
	425.1453	0.001	2.1169	ST 18:4;O8	C18H22O8	[M+OAc]-

425.1462343						
427.1526055	427.1529	0.000	0.7023	ST 21:4;O7	C21H28O7	[M+Cl]-
	429.0861	0.001	3.0297	ST 18:4;O7;S	C18H22O10S	[M-H]-
429.0848509						
429.1330830	429.1322	0.001	2.0973	ST 20:4;O8	C20H26O8	[M+Cl]-
	431.1478	0.001	1.3916	ST 20:3;O8	C20H28O8	[M+Cl]-
431.1472052						
433.1533654	433.1538	0.000	0.9235	ST 19:1;O6;S	C19H30O9S	[M-H]-
	433.1538	0.000	0.9235	ST 18:1;O4;S	C18H28O7S	[M+Formate]-
433.1533654						
434.1469374	434.1457	0.001	2.9944	ST 19:5;O8;G	C21H25NO9	[M-H]-
	434.1457	0.001	2.9944	ST 18:5;O6;G	C20H23NO7	[M+Formate]-
434.1469374						
436.1287552	436.1283	0.001	1.1465	NAT 14:4;O4	C16H25NO8S	[M+Formate]-
	436.1532	0.000	0.6878	ST 20:5;O5;G	C22H27NO6	[M+Cl]-
436.1529367						
438.1432575	438.144	0.001	1.5976	NAT 14:3;O4	C16H27NO8S	[M+Formate]-
	438.144	0.001	1.5976	NAT 13:3;O4	C15H25NO8S	[M+OAc]-
438.1432575						
438.2991085	438.299	0.000	0.2282	LPC O-13:0	C21H46NO6P	[M-H]-
	438.299	0.000	0.2282	LPE O-16:0	C21H46NO6P	[M-H]-
438.2991085						
438.2991085	438.2992	0.000	0.2282	NAE 20:0;O3	C22H45NO5	[M+Cl]-
	438.299	0.001	3.1942	LPC O-13:0	C21H46NO6P	[M-H]-
438.3003678						
438.3003678	438.299	0.001	3.1942	LPE O-16:0	C21H46NO6P	[M-H]-
	438.2992	0.001	2.7379	NAE 20:0;O3	C22H45NO5	[M+Cl]-
438.3003678						
439.1525347	439.1529	0.000	0.9108	ST 22:5;O7	C22H28O7	[M+Cl]-
	440.1482	0.001	1.3632	ST 19:4;O6;G	C21H27NO7	[M+Cl]-
440.1475882						
441.1680986	441.1686	0.001	1.1334	ST 22:4;O7	C22H30O7	[M+Cl]-
	442.1541	0.002	4.2971	ST 18:3;O6;T	C20H29NO8S	[M-H]-
442.1522261						
443.1474873	443.1478	0.000	0.6770	ST 21:4;O8	C21H28O8	[M+Cl]-
	447.1331	0.001	1.3419	ST 19:2;O7;S	C19H28O10S	[M-H]-
447.1336885						
447.1336885	447.1331	0.001	1.3419	ST 18:2;O5;S	C18H26O8S	[M+Formate]-

	447.1556	0.000	0.6709	LPG 11:1	C17H33O9P	[M+Cl]-
447.1559627						
447.1559627	447.1556	0.000	0.6709	LPG O-11:2;O	C17H33O9P	[M+Cl]-
	448.1647	0.001	2.6776	NAT 16:4;O3	C18H29NO7S	[M+Formate]-
448.1659245						
448.1659245	448.1647	0.001	2.6776	NAT 15:4;O3	C17H27NO7S	[M+OAc]-
	449.1487	0.001	2.0038	ST 19:1;O7;S	C19H30O10S	[M-H]-
449.1495707						
449.1495707	449.1487	0.001	2.0038	ST 18:1;O5;S	C18H28O8S	[M+Formate]-
	449.1487	0.002	4.0076	ST 19:1;O7;S	C19H30O10S	[M-H]-
449.1504771						
449.1504771	449.1487	0.002	4.0076	ST 18:1;O5;S	C18H28O8S	[M+Formate]-
	450.144	0.001	1.7772	NAT 15:4;O4	C17H27NO8S	[M+Formate]-
450.1447561						
450.1447561	450.144	0.001	1.7772	NAT 14:4;O4	C16H25NO8S	[M+OAc]-
	450.1535	0.000	0.6664	LPS 12:3;O	C18H30NO10P	[M-H]-
450.1537283						
451.1279381	451.128	0.000	0.0000	ST 18:1;O8;S	C18H28O11S	[M-H]-
	451.1375	0.001	2.2166	BMP 12:3;O	C18H29O11P	[M-H]-
451.1364739						
451.1364739	451.1352	0.001	2.8816	ST 23:6;O2;S	C23H28O5S	[M+Cl]-
	451.1529	0.000	0.2217	ST 23:6;O7	C23H28O7	[M+Cl]-
451.1529650						
451.1650428	451.1644	0.001	1.5515	ST 19:0;O7;S	C19H32O10S	[M-H]-
	451.1658	0.001	1.7732	LPA 17:4	C20H33O7P	[M+Cl]-
451.1650428						
451.1650428	451.1644	0.001	1.5515	ST 18:0;O5;S	C18H30O8S	[M+Formate]-
	452.1385	0.002	4.2023	ST 19:5;O6;T	C21H27NO8S	[M-H]-
452.1365407						
452.1365407	452.1385	0.002	4.2023	ST 18:5;O4;T	C20H25NO6S	[M+Formate]-
	452.1482	0.000	0.6635	ST 20:5;O6;G	C22H27NO7	[M+Cl]-
452.1478897						
453.1451693	453.1451	0.000	0.2207	LPA 16:4;O	C19H31O8P	[M+Cl]-
	453.1531	0.001	1.7654	BMP 12:2;O	C18H31O11P	[M-H]-
453.1522923						
453.1522923	453.1531	0.001	1.7654	LPG 11:3	C17H29O9P	[M+Formate]-
	454.1541	0.002	3.7432	ST 19:4;O6;T	C21H29NO8S	[M-H]-

454.1524471						
454.1524471	454.1541	0.002	3.7432	ST 18:4;O4;T	C20H27NO6S	[M+Formate]-
	454.1638	0.000	0.4404	ST 20:4;O6;G	C22H29NO7	[M+Cl]-
454.1635801						
457.1632723	457.1635	0.000	0.4375	ST 22:4;O8	C22H30O8	[M+Cl]-
	459.1791	0.000	0.8711	ST 22:3;O8	C22H32O8	[M+Cl]-
459.1786877						
461.1361625	461.1349	0.001	2.8191	BMP 11:1	C17H31O10P	[M+Cl]-
	461.1349	0.001	2.8191	LPG 11:2;O	C17H31O10P	[M+Cl]-
461.1361625						
461.1639325	461.164	0.000	0.0000	ST 24:6;O4;S	C24H30O7S	[M-H]-
	461.164	0.000	0.0000	ST 23:6;O2;S	C23H28O5S	[M+Formate]-
461.1639325						
461.1639325	461.164	0.000	0.0000	ST 22:6;O2;S	C22H26O5S	[M+OAc]-
	463.128	0.000	0.4318	ST 19:2;O8;S	C19H28O11S	[M-H]-
463.1281910						
463.1281910	463.128	0.000	0.4318	ST 18:2;O6;S	C18H26O9S	[M+Formate]-
	463.1529	0.000	0.2159	ST 18:5;O;GlcA	C24H28O7	[M+Cl]-
463.1530291						
464.1617170	464.1596	0.002	4.5243	NAT 16:4;O4	C18H29NO8S	[M+Formate]-
	464.1596	0.002	4.5243	NAT 15:4;O4	C17H27NO8S	[M+OAc]-
464.1617170						
465.1204642	465.1225	0.002	4.2999	ST 22:6;O6;S	C22H26O9S	[M-H]-
	465.1225	0.002	4.2999	ST 21:6;O4;S	C21H24O7S	[M+Formate]-
465.1204642						
465.1442693	465.1436	0.001	1.5049	ST 19:1;O8;S	C19H30O11S	[M-H]-
	465.1436	0.001	1.5049	ST 18:1;O6;S	C18H28O9S	[M+Formate]-
465.1442693						
466.1525044	466.1541	0.002	3.4323	ST 20:5;O6;T	C22H29NO8S	[M-H]-
	466.1541	0.002	3.4323	ST 19:5;O4;T	C21H27NO6S	[M+Formate]-
466.1525044						
466.1525044	466.1541	0.002	3.4323	ST 18:5;O4;T	C20H25NO6S	[M+OAc]-
	466.3303	0.001	2.5733	LPC O-15:0	C23H50NO6P	[M-H]-
466.3291093						
466.3291093	466.3303	0.001	2.5733	LPE O-18:0	C23H50NO6P	[M-H]-
	466.3305	0.001	3.0022	NAE 22:0;O3	C24H49NO5	[M+Cl]-
466.3291093						
466.3309150	466.3303	0.001	1.2866	LPC O-15:0	C23H50NO6P	[M-H]-

466.3309150	466.3303	0.001	1.2866	LPE O-18:0	C23H50NO6P	[M-H]-
466.3309150	466.3305	0.000	0.8578	NAE 22:0;O3	C24H49NO5	[M+Cl]-
467.1477768	467.1478	0.000	0.2141	ST 23:6;O8	C23H28O8	[M+Cl]-
467.1586711	467.1593	0.001	1.2844	ST 19:0;O8;S	C19H32O11S	[M-H]-
467.1586711	467.1593	0.001	1.2844	ST 18:0;O6;S	C18H30O9S	[M+Formate]-
468.1333275	468.1334	0.000	0.2136	ST 19:5;O7;T	C21H27NO9S	[M-H]-
468.1333275	468.1334	0.000	0.2136	ST 18:5;O5;T	C20H25NO7S	[M+Formate]-
468.1523557	468.1511	0.001	2.5633	ST 18:4;O8;G	C20H25NO9	[M+Formate]-
469.1635047	469.1635	0.000	0.0000	ST 23:5;O8	C23H30O8	[M+Cl]-
470.1469052	470.149	0.002	4.4667	ST 19:4;O7;T	C21H29NO9S	[M-H]-
470.1469052	470.149	0.002	4.4667	ST 18:4;O5;T	C20H27NO7S	[M+Formate]-
472.1507580	472.1509	0.000	0.2118	LPS 12:2	C18H32NO9P	[M+Cl]-
472.1507580	472.1509	0.000	0.2118	LPS O-12:3;O	C18H32NO9P	[M+Cl]-
472.1743929	472.1744	0.000	0.0000	ST 20:3;O7;G	C22H31NO8	[M+Cl]-
475.1156271	475.1142	0.002	3.1571	BMP 11:2;O	C17H29O11P	[M+Cl]-
475.1741137	475.1739	0.000	0.4209	BMP 15:4	C21H33O10P	[M-H]-
477.1565314	477.1589	0.002	4.8202	ST 24:6;O5;S	C24H30O8S	[M-H]-
477.1565314	477.1589	0.002	4.8202	ST 23:6;O3;S	C23H28O6S	[M+Formate]-
477.1565314	477.1589	0.002	4.8202	ST 22:6;O3;S	C22H26O6S	[M+OAc]-
478.1621037	478.1614	0.001	1.4639	LPS 11:0;O	C17H34NO10P	[M+Cl]-
479.1359476	479.1381	0.002	4.5916	ST 23:6;O6;S	C23H28O9S	[M-H]-
479.1359476	479.1381	0.002	4.5916	ST 22:6;O4;S	C22H26O7S	[M+Formate]-
479.1359476	479.1381	0.002	4.5916	ST 21:6;O4;S	C21H24O7S	[M+OAc]-
479.1448388	479.1455	0.001	1.2522	BMP 11:0;O	C17H33O11P	[M+Cl]-
	479.1745	0.002	3.5478	ST 24:5;O5;S	C24H32O8S	[M-H]-

479.1727993						
479.1727993	479.1745	0.002	3.5478	ST 23:5;O3;S	C23H30O6S	[M+Formate]-
	479.1745	0.002	3.5478	ST 22:5;O3;S	C22H28O6S	[M+OAc]-
479.1727993						
481.1639615	481.1635	0.001	1.0391	ST 18:4;O2;GlcA	C24H30O8	[M+Cl]-
	481.1635	0.001	1.0391	ST 18:5;O3;Hex	C24H30O8	[M+Cl]-
481.1639615						
481.1639615	481.1635	0.001	1.0391	ST 24:6;O8	C24H30O8	[M+Cl]-
	481.296	0.002	3.1166	ST 30:6;O5	C30H42O5	[M-H]-
481.2974575						
481.2974575	481.2993	0.002	3.9476	ST 27:1;O2;S	C27H46O5S	[M-H]-
	481.296	0.002	3.1166	ST 29:6;O3	C29H40O3	[M+Formate]-
481.2974575						
481.2974575	481.296	0.002	3.1166	ST 28:6;O3	C28H38O3	[M+OAc]-
	482.1433	0.001	2.9037	LPS 11:3;O	C17H28NO10P	[M+Formate]-
482.1447020						
482.1584553	482.1587	0.000	0.6222	ST 21:5;O7;G	C23H29NO8	[M+Cl]-
	482.1716	0.001	1.4518	LPE 15:4;O	C20H34NO8P	[M+Cl]-
482.1723627						
482.1723627	482.1716	0.001	1.4518	LPS O-14:4	C20H34NO8P	[M+Cl]-
	484.1647	0.002	3.5112	ST 20:4;O7;T	C22H31NO9S	[M-H]-
484.1630332						
484.1630332	484.1647	0.002	3.5112	ST 19:4;O5;T	C21H29NO7S	[M+Formate]-
	484.1647	0.002	3.5112	ST 18:4;O5;T	C20H27NO7S	[M+OAc]-
484.1630332						
486.1440801	486.144	0.000	0.2057	ST 19:4;O8;T	C21H29NO10S	[M-H]-
	486.144	0.000	0.2057	ST 18:4;O6;T	C20H27NO8S	[M+Formate]-
486.1440801						
488.1687155	488.1693	0.001	1.2291	ST 20:3;O8;G	C22H31NO9	[M+Cl]-
	488.1693	0.002	3.4824	ST 20:3;O8;G	C22H31NO9	[M+Cl]-
488.1710203						
489.1442759	489.1436	0.001	1.4311	ST 21:3;O8;S	C21H30O11S	[M-H]-
	489.1436	0.001	1.4311	ST 20:3;O6;S	C20H28O9S	[M+Formate]-
489.1442759						
489.1442759	489.1436	0.001	1.4311	ST 19:3;O6;S	C19H26O9S	[M+OAc]-
	491.1229	0.002	3.2578	ST 19:3;O7;S	C19H26O10S	[M+Formate]-
491.1244477						
491.1244477	491.1229	0.002	3.2578	ST 18:3;O7;S	C18H24O10S	[M+OAc]-

	493.1385	0.000	0.0000	ST 19:2;O7;S	C19H28O10S	[M+Formate]-
493.1385319						
493.1385319	493.1385	0.000	0.0000	ST 18:2;O7;S	C18H26O10S	[M+OAc]-
	493.1538	0.002	3.6500	ST 24:6;O6;S	C24H30O9S	[M-H]-
493.1519508						
493.1519508	493.1538	0.002	3.6500	ST 23:6;O4;S	C23H28O7S	[M+Formate]-
	493.1538	0.002	3.6500	ST 22:6;O4;S	C22H26O7S	[M+OAc]-
493.1519508						
494.1571086	494.1587	0.002	3.2378	ST 22:6;O7;G	C24H29NO8	[M+Cl]-
	494.1951	0.000	0.2023	ST 23:5;O6;G	C25H33NO7	[M+Cl]-
494.1951993						
495.1320497	495.1331	0.001	2.0197	ST 23:6;O7;S	C23H28O10S	[M-H]-
	495.1331	0.001	2.0197	ST 22:6;O5;S	C22H26O8S	[M+Formate]-
495.1320497						
495.1320497	495.1331	0.001	2.0197	ST 21:6;O5;S	C21H24O8S	[M+OAc]-
	495.1427	0.001	1.0098	ST 18:5;O3;GlcA	C24H28O9	[M+Cl]-
495.1432132						
495.1676819	495.1694	0.002	3.6351	ST 24:5;O6;S	C24H32O9S	[M-H]-
	495.1694	0.002	3.6351	ST 23:5;O4;S	C23H30O7S	[M+Formate]-
495.1676819						
495.1676819	495.1694	0.002	3.6351	ST 22:5;O4;S	C22H28O7S	[M+OAc]-
	495.1791	0.000	0.0000	ST 19:4;O2;GlcA	C25H32O8	[M+Cl]-
495.1790856						
495.1790856	495.1791	0.000	0.0000	ST 19:5;O3;Hex	C25H32O8	[M+Cl]-
	495.1791	0.000	0.0000	ST 25:6;O8	C25H32O8	[M+Cl]-
495.1790856						
495.1814848	495.1825	0.001	2.0195	ST 22:1;O5;S	C22H36O8S	[M+Cl]-
	496.1509	0.001	1.6124	LPS 14:4	C20H32NO9P	[M+Cl]-
496.1516692						
496.1629625	496.1647	0.002	3.4263	ST 21:5;O7;T	C23H31NO9S	[M-H]-
	496.1647	0.002	3.4263	ST 20:5;O5;T	C22H29NO7S	[M+Formate]-
496.1629625						
496.1629625	496.1647	0.002	3.4263	ST 19:5;O5;T	C21H27NO7S	[M+OAc]-
	497.1584	0.001	2.0114	ST 18:4;O3;GlcA	C24H30O9	[M+Cl]-
497.1573676						
497.1573676	497.1584	0.001	2.0114	ST 18:5;O4;Hex	C24H30O9	[M+Cl]-
	497.1713	0.002	3.8216	LPG 15:4	C21H35O9P	[M+Cl]-

497.1731348						
498.1538798	498.1536	0.000	0.4015	ST 21:5;O8;G	C23H29NO9	[M+Cl]-
	498.1665	0.000	0.6022	LPS 14:3	C20H34NO9P	[M+Cl]-
498.1668724						
498.1668724	498.1665	0.000	0.6022	LPS O-14:4;O	C20H34NO9P	[M+Cl]-
	498.1746	0.002	3.6132	LPS 12:2;O	C18H32NO10P	[M+Formate]-
498.1764000						
498.1764000	498.1746	0.002	3.6132	LPS 11:2;O	C17H30NO10P	[M+OAc]-
	499.174	0.001	1.2020	ST 18:3;O3;GlcA	C24H32O9	[M+Cl]-
499.1746201						
499.1746201	499.174	0.001	1.2020	ST 18:4;O4;Hex	C24H32O9	[M+Cl]-
	500.196	0.003	4.9980	ST 21:3;O7;T	C23H35NO9S	[M-H]-
500.1934520						
500.1934520	500.196	0.003	4.9980	ST 20:3;O5;T	C22H33NO7S	[M+Formate]-
	500.196	0.003	4.9980	ST 19:3;O5;T	C21H31NO7S	[M+OAc]-
500.1934520						
504.1549194	504.1545	0.000	0.7934	ST 18:3;O7;T	C20H29NO9S	[M+Formate]-
	505.1021	0.002	3.5636	ST 19:4;O8;S	C19H24O11S	[M+Formate]-
505.1039379						
505.1039379	505.1021	0.002	3.5636	ST 18:4;O8;S	C18H22O11S	[M+OAc]-
	507.1178	0.002	3.1551	ST 19:3;O8;S	C19H26O11S	[M+Formate]-
507.1193749						
507.1193749	507.1178	0.002	3.1551	ST 18:3;O8;S	C18H24O11S	[M+OAc]-
	507.1542	0.001	0.9859	ST 20:2;O7;S	C20H30O10S	[M+Formate]-
507.1546880						
507.1546880	507.1542	0.001	0.9859	ST 19:2;O7;S	C19H28O10S	[M+OAc]-
	509.1334	0.002	3.9282	ST 19:2;O8;S	C19H28O11S	[M+Formate]-
509.1354224						
509.1354224	509.1334	0.002	3.9282	ST 18:2;O8;S	C18H26O11S	[M+OAc]-
	509.1487	0.002	3.1425	ST 24:6;O7;S	C24H30O10S	[M-H]-
509.1471310						
509.1471310	509.1487	0.002	3.1425	ST 23:6;O5;S	C23H28O8S	[M+Formate]-
	509.1487	0.002	3.1425	ST 22:6;O5;S	C22H26O8S	[M+OAc]-
509.1471310						
510.1523227	510.1536	0.001	2.5483	ST 22:6;O8;G	C24H29NO9	[M+Cl]-
	510.3225	0.001	2.1555	ST 29:5;O4;G	C31H45NO5	[M-H]-
510.3213742						
510.3213742	510.3201	0.001	2.3515	LPC 16:0;O	C24H50NO8P	[M-H]-

	510.3201	0.001	2.3515	LPE 19:0;O	C24H50NO8P	[M-H]-
510.3213742						
510.3213742	510.3201	0.001	2.3515	LPS O-18:0	C24H50NO8P	[M-H]-
	510.3203	0.001	2.1555	CAR 18:0;O3	C25H49NO7	[M+Cl]-
510.3213742						
510.3213742	510.3201	0.001	2.3515	LPC O-15:1	C23H48NO6P	[M+Formate]-
	510.3201	0.001	2.3515	LPE O-18:1	C23H48NO6P	[M+Formate]-
510.3213742						
510.3213742	510.3201	0.001	2.3515	LPC O-14:1	C22H46NO6P	[M+OAc]-
	510.3201	0.001	2.3515	LPE O-17:1	C22H46NO6P	[M+OAc]-
510.3213742						
511.1628324	511.1644	0.002	2.9345	ST 24:5;O7;S	C24H32O10S	[M-H]-
	511.1644	0.002	2.9345	ST 23:5;O5;S	C23H30O8S	[M+Formate]-
511.1628324						
511.1628324	511.1644	0.002	2.9345	ST 22:5;O5;S	C22H28O8S	[M+OAc]-
	512.1458	0.001	1.3668	LPS 14:4;O	C20H32NO10P	[M+Cl]-
512.1464720						
512.1593427	512.1596	0.000	0.5858	ST 21:5;O8;T	C23H31NO10S	[M-H]-
	512.1596	0.000	0.5858	ST 20:5;O6;T	C22H29NO8S	[M+Formate]-
512.1593427						
512.1593427	512.1596	0.000	0.5858	ST 19:5;O6;T	C21H27NO8S	[M+OAc]-
	512.3382	0.001	2.3422	ST 29:4;O4;G	C31H47NO5	[M-H]-
512.3369596						
512.3369596	512.3358	0.001	2.3422	LPC O-15:0	C23H50NO6P	[M+Formate]-
	512.3358	0.001	2.3422	LPE O-18:0	C23H50NO6P	[M+Formate]-
512.3369596						
512.3369596	512.3358	0.001	2.3422	LPC O-14:0	C22H48NO6P	[M+OAc]-
	512.3358	0.001	2.3422	LPE O-17:0	C22H48NO6P	[M+OAc]-
512.3369596						
513.1582522	513.1567	0.002	3.1180	ST 21:1;O7;S	C21H34O10S	[M+Cl]-
	513.1662	0.002	4.2871	BMP 15:3	C21H35O10P	[M+Cl]-
513.1684169						
513.1684169	513.1662	0.002	4.2871	LPG 15:4;O	C21H35O10P	[M+Cl]-
	513.1743	0.001	2.1435	LPI 11:2;O	C20H35O13P	[M-H]-
513.1753925						
513.1753925	513.1743	0.001	2.1435	BMP 13:2;O	C19H33O11P	[M+Formate]-
	513.1743	0.001	2.1435	BMP 12:2;O	C18H31O11P	[M+OAc]-

513.1753925						
513.3407590	513.3433	0.003	4.8700	ST 28:0;O8	C28H50O8	[M-H]-
	513.3433	0.003	4.8700	DG 24:2;O	C27H48O6	[M+Formate]-
513.3407590						
513.3407590	513.3433	0.003	4.8700	DG O-24:3;O2	C27H48O6	[M+Formate]-
	513.3433	0.003	4.8700	FA 27:3;O4	C27H48O6	[M+Formate]-
513.3407590						
513.3407590	513.3433	0.003	4.8700	ST 27:0;O6	C27H48O6	[M+Formate]-
	513.3433	0.003	4.8700	DG 23:2;O	C26H46O6	[M+OAc]-
513.3407590						
513.3407590	513.3433	0.003	4.8700	DG O-23:3;O2	C26H46O6	[M+OAc]-
	513.3433	0.003	4.8700	FA 26:3;O4	C26H46O6	[M+OAc]-
513.3407590						
513.3407590	513.3433	0.003	4.8700	ST 26:0;O6	C26H46O6	[M+OAc]-
	514.1519	0.001	1.7505	ST 18:1;O8;T	C20H33NO10S	[M+Cl]-
514.1510187						
514.1621469	514.1614	0.001	1.3614	LPS 14:3;O	C20H34NO10P	[M+Cl]-
	517.0941	0.001	2.5140	ST 22:6;O7;S	C22H26O10S	[M+Cl]-
517.0954202						
520.1485805	520.1494	0.001	1.7303	ST 18:3;O8;T	C20H29NO10S	[M+Formate]-
	521.1334	0.001	1.5351	ST 20:3;O8;S	C20H28O11S	[M+Formate]-
521.1342673						
521.1342673	521.1334	0.001	1.5351	ST 19:3;O8;S	C19H26O11S	[M+OAc]-
	523.1644	0.002	3.6317	ST 25:6;O7;S	C25H32O10S	[M-H]-
523.1624124						
523.1624124	523.1644	0.002	3.6317	ST 24:6;O5;S	C24H30O8S	[M+Formate]-
	523.1644	0.002	3.6317	ST 23:6;O5;S	C23H28O8S	[M+OAc]-
523.1624124						
525.1422081	525.1436	0.001	2.6659	ST 24:6;O8;S	C24H30O11S	[M-H]-
	525.1436	0.001	2.6659	ST 23:6;O6;S	C23H28O9S	[M+Formate]-
525.1422081						
525.1422081	525.1436	0.001	2.6659	ST 22:6;O6;S	C22H26O9S	[M+OAc]-
	525.18	0.002	3.9986	ST 25:5;O7;S	C25H34O10S	[M-H]-
525.1778572						
525.1778572	525.18	0.002	3.9986	ST 24:5;O5;S	C24H32O8S	[M+Formate]-
	525.18	0.002	3.9986	ST 23:5;O5;S	C23H30O8S	[M+OAc]-
525.1778572						
526.1513869	526.1519	0.001	0.9503	ST 19:2;O8;T	C21H33NO10S	[M+Cl]-

526.1638781	526.1614	0.002	4.5613	LPS 15:4;O	C21H34NO10P	[M+Cl]-
527.1469937	527.1455	0.002	2.8455	BMP 15:4;O	C21H33O11P	[M+Cl]-
527.1578405	527.1593	0.001	2.6557	ST 24:5;O8;S	C24H32O11S	[M-H]-
527.1578405	527.1593	0.001	2.6557	ST 23:5;O6;S	C23H30O9S	[M+Formate]-
527.1578405	527.1593	0.001	2.6557	ST 22:5;O6;S	C22H28O9S	[M+OAc]-
528.1551809	528.1545	0.001	1.3254	ST 20:5;O7;T	C22H29NO9S	[M+Formate]-
528.1551809	528.1545	0.001	1.3254	ST 19:5;O7;T	C21H27NO9S	[M+OAc]-
529.1462624	529.1482	0.002	3.7797	ST 18:4;O5;GlcA	C24H30O11	[M+Cl]-
529.1462624	529.1482	0.002	3.7797	ST 18:5;O6;Hex	C24H30O11	[M+Cl]-
529.1535063	529.1516	0.002	3.5907	ST 21:1;O8;S	C21H34O11S	[M+Cl]-
529.1642465	529.1669	0.003	4.9134	ST 25:5;O5;S	C25H34O8S	[M+Cl]-
529.1735657	529.1749	0.001	2.6456	ST 24:4;O8;S	C24H34O11S	[M-H]-
529.1735657	529.1749	0.001	2.6456	ST 23:4;O6;S	C23H32O9S	[M+Formate]-
529.1735657	529.1749	0.001	2.6456	ST 22:4;O6;S	C22H30O9S	[M+OAc]-
531.1657147	531.1672	0.002	2.8240	ST 21:0;O8;S	C21H36O11S	[M+Cl]-
531.1657147	531.1639	0.002	3.3888	ST 18:3;O5;GlcA	C24H32O11	[M+Cl]-
531.1657147	531.1639	0.002	3.3888	ST 18:4;O6;Hex	C24H32O11	[M+Cl]-
531.1792847	531.1768	0.003	4.7065	BMP 15:2;O	C21H37O11P	[M+Cl]-
531.1792847	531.1768	0.003	4.7065	LPI O-12:3	C21H37O11P	[M+Cl]-
531.1806179	531.1825	0.002	3.5769	ST 25:4;O5;S	C25H36O8S	[M+Cl]-
535.1030194	535.1046	0.002	2.9901	ST 22:5;O8;S	C22H28O11S	[M+Cl]-
535.1626964	535.1644	0.002	3.1766	ST 26:7;O7;S	C26H32O10S	[M-H]-
535.1626964	535.1644	0.002	3.1766	ST 25:7;O5;S	C25H30O8S	[M+Formate]-
541.1521997	541.1516	0.001	1.1087	ST 22:2;O8;S	C22H34O11S	[M+Cl]-
	541.1749	0.000	0.1848	ST 25:5;O8;S	C25H34O11S	[M-H]-

541.1750091						
541.1750091	541.1749	0.000	0.1848	ST 24:5;O6;S	C24H32O9S	[M+Formate]-
	541.1749	0.000	0.1848	ST 23:5;O6;S	C23H30O9S	[M+OAc]-
541.1750091						
542.1607962	542.1621	0.001	2.3978	ST 23:6;O6;T	C25H33NO8S	[M+Cl]-
	543.1542	0.001	0.9205	ST 23:5;O7;S	C23H30O10S	[M+Formate]-
543.1536791						
543.1536791	543.1542	0.001	0.9205	ST 22:5;O7;S	C22H28O10S	[M+OAc]-
	543.1672	0.001	1.8411	ST 22:1;O8;S	C22H36O11S	[M+Cl]-
543.1662290						
544.1753523	544.1777	0.002	4.4103	ST 23:5;O6;T	C25H35NO8S	[M+Cl]-
	546.1651	0.000	0.1831	ST 20:4;O8;T	C22H31NO10S	[M+Formate]-
546.1650132						
546.1650132	546.1651	0.000	0.1831	ST 19:4;O8;T	C21H29NO10S	[M+OAc]-
	546.1934	0.002	4.2110	ST 23:4;O6;T	C25H37NO8S	[M+Cl]-
546.1911140						
549.1182160	549.1203	0.002	3.8243	ST 23:5;O8;S	C23H30O11S	[M+Cl]-
	554.1832	0.001	1.2631	ST 21:2;O8;T	C23H37NO10S	[M+Cl]-
554.1824969						
555.1546022	555.1542	0.000	0.7205	ST 24:6;O7;S	C24H30O10S	[M+Formate]-
	555.1542	0.000	0.7205	ST 23:6;O7;S	C23H28O10S	[M+OAc]-
555.1546022						
556.1864170	556.1858	0.001	1.0788	ST 22:5;O7;T	C24H33NO9S	[M+Formate]-
	556.1858	0.001	1.0788	ST 21:5;O7;T	C23H31NO9S	[M+OAc]-
556.1864170						
557.1691502	557.1698	0.001	1.2563	ST 24:5;O7;S	C24H32O10S	[M+Formate]-
	557.1698	0.001	1.2563	ST 23:5;O7;S	C23H30O10S	[M+OAc]-
557.1691502						
557.1707756	557.1698	0.001	1.6153	ST 24:5;O7;S	C24H32O10S	[M+Formate]-
	557.1698	0.001	1.6153	ST 23:5;O7;S	C23H30O10S	[M+OAc]-
557.1707756						
559.1861750	559.1855	0.001	1.2518	ST 24:4;O7;S	C24H34O10S	[M+Formate]-
	559.1855	0.001	1.2518	ST 23:4;O7;S	C23H32O10S	[M+OAc]-
559.1861750						
562.1859178	562.1883	0.002	4.2690	ST 23:4;O7;T	C25H37NO9S	[M+Cl]-
	564.2006	0.001	1.7724	ST 26:7;O8;G	C28H35NO9	[M+Cl]-
564.2016361						
568.1753544	568.1777	0.002	4.2240	ST 25:7;O6;T	C27H35NO8S	[M+Cl]-

	569.1829	0.001	1.5812	ST 24:2;O8;S	C24H38O11S	[M+Cl]-
569.1837685						
570.1659729	570.1651	0.001	1.5785	ST 22:6;O8;T	C24H31NO10S	[M+Formate]-
570.1659729	570.1651	0.001	1.5785	ST 21:6;O8;T	C23H29NO10S	[M+OAc]-
	570.1748	0.002	2.8062	ST 18:5;O6;HexNAc	C26H33NO11	[M+Cl]-
570.1763674						
571.1980687	571.1985	0.001	0.8754	ST 24:1;O8;S	C24H40O11S	[M+Cl]-
	571.2032	0.001	1.2255	ST 20:3;O8;GlcA	C26H36O14	[M-H]-
571.2025156						
571.2025156	571.2032	0.001	1.2255	ST 19:3;O6;GlcA	C25H34O12	[M+Formate]-
	571.2032	0.001	1.2255	ST 19:4;O7;Hex	C25H34O12	[M+Formate]-
571.2025156						
571.2025156	571.2032	0.001	1.2255	ST 18:3;O6;GlcA	C24H32O12	[M+OAc]-
571.2025156	571.2032	0.001	1.2255	ST 18:4;O7;Hex	C24H32O12	[M+OAc]-
572.1714358	572.1727	0.001	2.0973	ST 24:6;O7;T	C26H35NO9S	[M+Cl]-
	572.1807	0.001	1.9225	ST 22:5;O8;T	C24H33NO10S	[M+Formate]-
572.1818366						
572.1818366	572.1807	0.001	1.9225	ST 21:5;O8;T	C23H31NO10S	[M+OAc]-
	573.1567	0.002	4.0129	ST 26:6;O7;S	C26H34O10S	[M+Cl]-
573.1543664						
573.1758768	573.1744	0.001	2.4425	ST 20:4;O6;GlcA	C26H34O12	[M+Cl]-
	573.1744	0.001	2.4425	ST 20:5;O7;Hex	C26H34O12	[M+Cl]-
573.1758768						
574.1969939	574.1964	0.001	1.0449	ST 22:4;O8;T	C24H35NO10S	[M+Formate]-
	574.1964	0.001	1.0449	ST 21:4;O8;T	C23H33NO10S	[M+OAc]-
574.1969939						
577.1969353	577.196	0.001	1.5593	ST 24:3;O8;S	C24H36O11S	[M+Formate]-
	577.196	0.001	1.5593	ST 23:3;O8;S	C23H34O11S	[M+OAc]-
577.1969353						
583.1846078	583.1855	0.001	1.5432	ST 26:6;O7;S	C26H34O10S	[M+Formate]-
	583.1855	0.001	1.5432	ST 25:6;O7;S	C25H32O10S	[M+OAc]-
583.1846078						
584.1713200	584.1727	0.001	2.2254	ST 25:7;O7;T	C27H35NO9S	[M+Cl]-
	585.1461	0.002	3.0762	ST 18:5;O8;GlcA	C24H28O14	[M+Formate]-
585.1443160						
585.1660515	585.1647	0.001	2.2216	ST 25:6;O8;S	C25H32O11S	[M+Formate]-

585.1660515	585.1647	0.001	2.2216	ST 24:6;O8;S	C24H30O11S	[M+OAc]-
585.1814564	585.1825	0.001	1.7089	ST 19:4;O7;GlcA	C25H32O13	[M+Formate]-
585.1814564	585.1825	0.001	1.7089	ST 19:5;O8;Hex	C25H32O13	[M+Formate]-
585.1814564	585.1825	0.001	1.7089	ST 18:4;O7;GlcA	C24H30O13	[M+OAc]-
585.1814564	585.1825	0.001	1.7089	ST 18:5;O8;Hex	C24H30O13	[M+OAc]-
586.2075383	586.2061	0.002	2.5588	ST 19:4;O6;HexNAc	C27H37NO11	[M+Cl]-
587.1340672	587.1359	0.002	3.2360	ST 26:7;O8;S	C26H32O11S	[M+Cl]-
587.1566288	587.1537	0.003	4.9391	ST 20:5;O7;GlcA	C26H32O13	[M+Cl]-
587.1670084	587.1666	0.000	0.6812	LPI 14:4;O	C23H37O13P	[M+Cl]-
587.1713648	587.1723	0.001	1.7031	ST 27:6;O7;S	C27H36O10S	[M+Cl]-
588.1878863	588.1853	0.003	4.2504	ST 18:4;O7;HexNAc	C26H35NO12	[M+Cl]-
588.2022066	588.204	0.002	3.0602	ST 25:5;O7;T	C27H39NO9S	[M+Cl]-
589.2108648	589.2138	0.003	4.9218	ST 19:2;O7;GlcA	C25H36O13	[M+Formate]-
589.2108648	589.2138	0.003	4.9218	ST 19:3;O8;Hex	C25H36O13	[M+Formate]-
589.2108648	589.2138	0.003	4.9218	ST 18:2;O7;GlcA	C24H34O13	[M+OAc]-
589.2108648	589.2138	0.003	4.9218	ST 18:3;O8;Hex	C24H34O13	[M+OAc]-
590.1815382	590.1832	0.002	2.8805	ST 24:5;O8;T	C26H37NO10S	[M+Cl]-
592.1965482	592.1989	0.002	3.8838	ST 24:4;O8;T	C26H39NO10S	[M+Cl]-
593.1823922	593.1829	0.001	0.8429	ST 26:4;O8;S	C26H38O11S	[M+Cl]-
597.1655396	597.1647	0.001	1.3397	ST 26:7;O8;S	C26H32O11S	[M+Formate]-
597.1655396	597.1647	0.001	1.3397	ST 25:7;O8;S	C25H30O11S	[M+OAc]-
599.1822703	599.1804	0.002	3.1710	ST 26:6;O8;S	C26H34O11S	[M+Formate]-
599.1822703	599.1804	0.002	3.1710	ST 25:6;O8;S	C25H32O11S	[M+OAc]-
601.1846219	601.1822	0.002	3.9921	LPI 15:4;O	C24H39O13P	[M+Cl]-
601.1951890	601.196	0.001	1.4970	ST 26:5;O8;S	C26H36O11S	[M+Formate]-

601.1951890	601.196	0.001	1.4970	ST 25:5;O8;S	C25H34O11S	[M+OAc]-
602.1674429	602.1646	0.003	4.6499	ST 18:5;O8;HexNAc	C26H33NO13	[M+Cl]-
602.1817506	602.1832	0.002	2.4909	ST 25:6;O8;T	C27H37NO10S	[M+Cl]-
603.1905820	603.1931	0.003	4.1446	ST 19:3;O8;GlcA	C25H34O14	[M+Formate]-
603.1905820	603.1931	0.003	4.1446	ST 18:3;O8;GlcA	C24H32O14	[M+OAc]-
603.2086980	603.2117	0.003	4.9734	ST 26:4;O8;S	C26H38O11S	[M+Formate]-
603.2086980	603.2117	0.003	4.9734	ST 25:4;O8;S	C25H36O11S	[M+OAc]-
603.2107270	603.2117	0.001	1.6578	ST 26:4;O8;S	C26H38O11S	[M+Formate]-
603.2107270	603.2117	0.001	1.6578	ST 25:4;O8;S	C25H36O11S	[M+OAc]-
605.1816702	605.1829	0.001	1.9829	ST 27:5;O8;S	C27H38O11S	[M+Cl]-
611.1930433	611.1901	0.003	4.9085	ST 23:6;O6;GlcA	C29H36O12	[M+Cl]-
615.1873765	615.185	0.002	3.9013	ST 22:5;O7;GlcA	C28H36O13	[M+Cl]-
615.1873765	615.185	0.002	3.9013	ST 22:6;O8;Hex	C28H36O13	[M+Cl]-
615.1979499	615.1979	0.000	0.1625	LPI 16:4;O	C25H41O13P	[M+Cl]-
615.2010632	615.2036	0.003	4.2262	ST 29:6;O7;S	C29H40O10S	[M+Cl]-
617.1842578	617.1829	0.001	2.2684	ST 28:6;O8;S	C28H38O11S	[M+Cl]-
617.2163087	617.2135	0.003	4.5365	LPI 16:3;O	C25H43O13P	[M+Cl]-
617.2163087	617.2193	0.003	4.8605	ST 29:5;O7;S	C29H42O10S	[M+Cl]-
618.1985426	618.1959	0.003	4.2058	ST 19:4;O8;HexNAc	C27H37NO13	[M+Cl]-
619.1957131	619.1985	0.003	4.5220	ST 28:5;O8;S	C28H40O11S	[M+Cl]-
620.2060974	620.2033	0.003	4.5146	PS 21:6;O	C27H40NO11P	[M+Cl]-
627.1877046	627.185	0.003	4.3049	ST 23:6;O7;GlcA	C29H36O13	[M+Cl]-
629.1809284	629.1829	0.002	3.1787	ST 29:7;O8;S	C29H38O11S	[M+Cl]-
631.1960284	631.1985	0.003	3.9607	ST 29:6;O8;S	C29H40O11S	[M+Cl]-
633.2117610	633.2142	0.002	3.7902	ST 29:5;O8;S	C29H42O11S	[M+Cl]-

	637.2138	0.001	2.1971	ST 23:6;O7;GlcA	C29H36O13	[M+Formate]-
637.2151869						
637.2151869	637.2138	0.001	2.1971	ST 22:6;O7;GlcA	C28H34O13	[M+OAc]-
	642.1959	0.002	2.8029	ST 21:6;O8;HexNAc	C29H37NO13	[M+Cl]-
642.1977380						
673.4836850	673.4838	0.000	0.1485	DG 41:10	C44H66O5	[M-H]-
	673.4838	0.000	0.1485	DG O-41:11;O	C44H66O5	[M-H]-
673.4836850						
673.4836850	673.4838	0.000	0.1485	TG O-41:10	C44H66O5	[M-H]-
674.4870921	674.4849	0.002	3.2617	HexCer 30:1;O4	C36H69NO10	[M-H]-
	674.4849	0.002	3.2617	CAR 28:1;O4	C35H67NO8	[M+Formate]-
674.4870921						
674.4870921	674.4849	0.002	3.2617	HexCer 29:1;O2	C35H67NO8	[M+Formate]-
674.4870921	674.4849	0.002	3.2617	CAR 27:1;O4	C34H65NO8	[M+OAc]-
	674.4849	0.002	3.2617	HexCer 28:1;O2	C34H65NO8	[M+OAc]-
674.4870921						
683.2139360	683.2112	0.003	3.9519	ST 26:7;O8;GlcA	C32H40O14	[M+Cl]-
	693.2448	0.003	4.0390	PI 22:6	C31H47O13P	[M+Cl]-
693.2476040						
695.2259193	695.2241	0.002	2.5891	PI 21:6;O	C30H45O14P	[M+Cl]-
712.3719676	712.3736	0.002	2.2460	SHexCer 30:6;O2	C36H59NO11S	[M-H]-
712.3719676	712.3702	0.002	2.3864	ST 30:7;O4;HexNAc	C38H53NO9	[M+Formate]-
	712.3702	0.002	2.3864	ST 29:7;O4;HexNAc	C37H51NO9	[M+OAc]-
712.3719676						
713.3755095	713.3754	0.000	0.1402	ST 30:2;O8;GlcA	C36H58O14	[M-H]-
	713.3754	0.000	0.1402	ST 29:2;O6;GlcA	C35H56O12	[M+Formate]-
713.3755095						
713.3755095	713.3754	0.000	0.1402	ST 29:3;O7;Hex	C35H56O12	[M+Formate]-
713.3755095	713.3754	0.000	0.1402	ST 28:2;O6;GlcA	C34H54O12	[M+OAc]-
	713.3754	0.000	0.1402	ST 28:3;O7;Hex	C34H54O12	[M+OAc]-
713.3755095						
727.2157696	727.2138	0.002	2.7502	PIP 20:5;O	C29H46O17P2	[M-H]-
	732.4821	0.003	3.8226	CerP 38:3;O6	C38H72NO10P	[M-H]-
732.4849415						
732.4849415	732.4821	0.003	3.8226	LPS 32:2;O	C38H72NO10P	[M-H]-

732.4849415	732.4821	0.003	3.8226	PS 32:1	C38H72NO10P	[M-H]-
732.4849415	732.4821	0.003	3.8226	PS O-32:2;O	C38H72NO10P	[M-H]-
732.4849415	732.4823	0.003	3.5496	HexCer 33:3;O3	C39H71NO9	[M+Cl]-
732.4849415	732.4821	0.003	3.8226	CerP 37:3;O4	C37H70NO8P	[M+Formate]-
732.4849415	732.4821	0.003	3.8226	LPC 29:3;O	C37H70NO8P	[M+Formate]-
732.4849415	732.4821	0.003	3.8226	LPE 32:3;O	C37H70NO8P	[M+Formate]-
732.4849415	732.4821	0.003	3.8226	LPS O-31:3	C37H70NO8P	[M+Formate]-
732.4849415	732.4821	0.003	3.8226	PC 29:2	C37H70NO8P	[M+Formate]-
732.4849415	732.4821	0.003	3.8226	PC O-29:3;O	C37H70NO8P	[M+Formate]-
732.4849415	732.4821	0.003	3.8226	PE 32:2	C37H70NO8P	[M+Formate]-
732.4849415	732.4821	0.003	3.8226	PE O-32:3;O	C37H70NO8P	[M+Formate]-
732.4849415	732.4821	0.003	3.8226	CerP 36:3;O4	C36H68NO8P	[M+OAc]-
732.4849415	732.4821	0.003	3.8226	LPC 28:3;O	C36H68NO8P	[M+OAc]-
732.4849415	732.4821	0.003	3.8226	LPE 31:3;O	C36H68NO8P	[M+OAc]-
732.4849415	732.4821	0.003	3.8226	LPS O-30:3	C36H68NO8P	[M+OAc]-
732.4849415	732.4821	0.003	3.8226	PC 28:2	C36H68NO8P	[M+OAc]-
732.4849415	732.4821	0.003	3.8226	PC O-28:3;O	C36H68NO8P	[M+OAc]-
732.4849415	732.4821	0.003	3.8226	PE 31:2	C36H68NO8P	[M+OAc]-
732.4849415	732.4821	0.003	3.8226	PE O-31:3;O	C36H68NO8P	[M+OAc]-
734.5371260	734.5342	0.003	4.0842	CerP 39:1;O5	C39H78NO9P	[M-H]-
734.5371260	734.5342	0.003	4.0842	LPS 33:0	C39H78NO9P	[M-H]-
734.5371260	734.5342	0.003	4.0842	LPS O-33:1;O	C39H78NO9P	[M-H]-
734.5371260	734.5342	0.003	4.0842	PE 34:0;O	C39H78NO9P	[M-H]-
734.5371260	734.5342	0.003	4.0842	PS O-33:0	C39H78NO9P	[M-H]-
	734.5343	0.003	3.8119	CAR 33:1;O4	C40H77NO8	[M+Cl]-

734.5371260						
734.5371260	734.5343	0.003	3.8119	HexCer 34:1;O2	C40H77NO8	[M+Cl]-
	734.5342	0.003	4.0842	CerP 38:1;O3	C38H76NO7P	[M+Formate]-
734.5371260						
734.5371260	734.5342	0.003	4.0842	LPC 30:1	C38H76NO7P	[M+Formate]-
	734.5342	0.003	4.0842	LPC O-30:2;O	C38H76NO7P	[M+Formate]-
734.5371260						
734.5371260	734.5342	0.003	4.0842	LPE 33:1	C38H76NO7P	[M+Formate]-
	734.5342	0.003	4.0842	LPE O-33:2;O	C38H76NO7P	[M+Formate]-
734.5371260						
734.5371260	734.5342	0.003	4.0842	PC O-30:1	C38H76NO7P	[M+Formate]-
	734.5342	0.003	4.0842	PE O-33:1	C38H76NO7P	[M+Formate]-
734.5371260						
734.5371260	734.5342	0.003	4.0842	CerP 37:1;O3	C37H74NO7P	[M+OAc]-
	734.5342	0.003	4.0842	LPC 29:1	C37H74NO7P	[M+OAc]-
734.5371260						
734.5371260	734.5342	0.003	4.0842	LPC O-29:2;O	C37H74NO7P	[M+OAc]-
	734.5342	0.003	4.0842	LPE 32:1	C37H74NO7P	[M+OAc]-
734.5371260						
734.5371260	734.5342	0.003	4.0842	LPE O-32:2;O	C37H74NO7P	[M+OAc]-
	734.5342	0.003	4.0842	PC O-29:1	C37H74NO7P	[M+OAc]-
734.5371260						
734.5371260	734.5342	0.003	4.0842	PE O-32:1	C37H74NO7P	[M+OAc]-
	734.5342	0.003	4.0842	PC 32:0;O	C40H80NO9P	[M-CH3]-
734.5371260						
737.2367583	737.2345	0.002	3.1198	PIP 22:6	C31H48O16P2	[M-H]-
	740.4049	0.001	1.8909	SHexCer 32:6;O2	C38H63NO11S	[M-H]-
740.4035358						
741.4069441	741.4067	0.000	0.4046	ST 30:2;O6;GlcA	C36H58O12	[M+OAc]-
	741.4067	0.000	0.4046	ST 30:3;O7;Hex	C36H58O12	[M+OAc]-
741.4069441						
748.5156800	748.5134	0.002	3.0728	CerP 39:2;O6	C39H76NO10P	[M-H]-
	748.5134	0.002	3.0728	LPS 33:1;O	C39H76NO10P	[M-H]-
748.5156800						
748.5156800	748.5134	0.002	3.0728	PS 33:0	C39H76NO10P	[M-H]-
	748.5134	0.002	3.0728	PS O-33:1;O	C39H76NO10P	[M-H]-
748.5156800						
748.5156800	748.5136	0.002	2.8056	HexCer 34:2;O3	C40H75NO9	[M+Cl]-

748.5156800	748.5134	0.002	3.0728	CerP 38:2;O4	C38H74NO8P	[M+Formate]-
748.5156800	748.5134	0.002	3.0728	LPC 30:2;O	C38H74NO8P	[M+Formate]-
748.5156800	748.5134	0.002	3.0728	LPE 33:2;O	C38H74NO8P	[M+Formate]-
748.5156800	748.5134	0.002	3.0728	LPS O-32:2	C38H74NO8P	[M+Formate]-
748.5156800	748.5134	0.002	3.0728	PC 30:1	C38H74NO8P	[M+Formate]-
748.5156800	748.5134	0.002	3.0728	PC O-30:2;O	C38H74NO8P	[M+Formate]-
748.5156800	748.5134	0.002	3.0728	PE 33:1	C38H74NO8P	[M+Formate]-
748.5156800	748.5134	0.002	3.0728	PE O-33:2;O	C38H74NO8P	[M+Formate]-
748.5156800	748.5134	0.002	3.0728	CerP 37:2;O4	C37H72NO8P	[M+OAc]-
748.5156800	748.5134	0.002	3.0728	LPC 29:2;O	C37H72NO8P	[M+OAc]-
748.5156800	748.5134	0.002	3.0728	LPE 32:2;O	C37H72NO8P	[M+OAc]-
748.5156800	748.5134	0.002	3.0728	LPS O-31:2	C37H72NO8P	[M+OAc]-
748.5156800	748.5134	0.002	3.0728	PC 29:1	C37H72NO8P	[M+OAc]-
748.5156800	748.5134	0.002	3.0728	PC O-29:2;O	C37H72NO8P	[M+OAc]-
748.5156800	748.5134	0.002	3.0728	PE 32:1	C37H72NO8P	[M+OAc]-
748.5156800	748.5134	0.002	3.0728	PE O-32:2;O	C37H72NO8P	[M+OAc]-
748.5237063	748.5217	0.002	2.6719	HexCer 33:1;O6	C39H75NO12	[M-H]-
748.5237063	748.5217	0.002	2.6719	HexCer 32:1;O4	C38H73NO10	[M+Formate]-
748.5237063	748.5217	0.002	2.6719	HexCer 31:1;O4	C37H71NO10	[M+OAc]-

Supplementary Table S9: LipidMatch Flow settings

Settings	Adjustment
**Retention time window**	**±0.15**
**Window for matching experimental and in-silico fragments**	**±5 ppm**
**Mass accuracy window for matching experimental and in-silico precursors for full scan**	**±0.005 Da**
**MS/MS isolation Window (for determining MS/MS scans for each feature)**	**1 Da**

**Threshold for determining what the minimum signal intensity cut off for a given MS/MS ion should be (used for confirmations)**	**1000**
**Minimum number of scans required for the result to be a confirmation (MS/MS)**	**1**
**Blank Filtering**	**Yes**

Supplementary Table S10: Identified markers for upregulated ion signals in positive-ion mode

MALDI			Previous annotations. now identified			
Input Mass	Matched Mass	Delta	ppm	Annotation	Formula	Adduct
	504.3085		5.3539 0	LPE 20:3	C25H46NO7P	[M+H]+
	508.3398	0.0010	1.9672	LPC 17:1	C25H50NO7P	[M+H]+
	508.3398		1.9672 0	LPE 20:1	C25H50NO7P	[M+H]+
	522.3554	0.0005	0.9572	LPC 18:1	C26H52NO7P	[M+H]+
	526.2928		8.1704 0	LPE 22:6	C27H44NO7P	[M+H]+
	530.3241	0.0029	5.4684	LPE 22:4	C27H48NO7P	[M+H]+
504.3057323	536.3711		2.4237 0	LPE 22:1	C27H54NO7P	[M+H]+
508.3407506						
508.3407506						
522.3549188						
526.2885253						
530.3211705						
536.3697517						
756.5878945						
756.5878945						
764.5562275						
766.5719673						
770.6046728						
	756.5902	0.0023	3.0400	PC O-35:3	C43H82NO7P	[M+H]+
	756.5902		3.0400 0	PE O-38:3	C43H82NO7P	[M+H]+
	764.5589	0.0026	3.4007	PC O-36:6	C44H78NO7P	[M+H]+
	766.5745		3.2613 0	PC O-36:5	C44H80NO7P	[M+H]+
	770.6058	0.0011	1.4274	PC O-36:3	C44H84NO7P	[M+H]+
780.5878989	780.5902	0.0023	2.9465	PE O-40:5	C45H82NO7P	[M+H]+
	790.5745		3.2887 (	PC O-38:7	C46H80NO7P	[M+H]+
	794.6058	0.0024	3.0204	PC O-38:5	C46H84NO7P	[M+H]+
	804.5749		2.1129 (	PS 37:1	C43H82NO10P	[M+H]+

	811.6687	0.0016		1.9712	SM 42:3;O2	C47H91N2O6P	[M+H]+
	818.6058		2.8097		PC O-40:7	C48H84NO7P	[M+H]+
	820.6215	0.0022		2.6809	PC O-40:6	C48H86NO7P	[M+H]+
	822.6371		3.1606		PC O-40:5	C48H88NO7P	[M+H]+
	824.6528	0.0033		4.0017	PC O-40:4	C48H90NO7P	[M+H]+
	826.5593		2.9036		PS 39:4	C45H80NO10P	[M+H]+
	848.7702	0.0007		0.8247	TG 50:2	C53H98O6	[M+NH4]+
	852.5749		0.3519		PS 41:5	C47H82NO10P	[M+H]+
	874.7858	0.0002		0.2286	TG 52:3	C55H100O6	[M+NH4]+
790.5719182			2.0529		TG 52:2	C55H102O6	[M+NH4]+
794.6034364							
804.5732351							
811.6671728							
818.6035184							
820.6192369							
822.6345256							
824.6494876							
826.5568119							
848.7694772							
852.5746330							
874.7855872							
876.7996313							
880.5845411							
894.6216745							
902.8169142							
904.8326454							
906.6170440							
908.6372910							
908.7757011							
910.7896721							
934.6531352							
934.7915814							
936.6673770	876.8015						
	880.591	0.0064		7.2678	PI 36:2	C45H83O13P	[M+NH4]+
	894.6219		0.2236		PS 44:5	C50H88NO10P	[M+H]+
	902.8171	0.0002		0.2215	TG 54:3	C57H104O6	[M+NH4]+
	904.8328		0.1105		TG 54:2	C57H106O6	[M+NH4]+
	906.6148	0.0022		2.4266	DGDG 32:2	C47H84O15	[M+NH4]+
	908.6305		7.4838		DGDG 32:1	C47H86O15	[M+NH4]+
	908.7702	0.0055		6.0521	TG 55:7	C58H98O6	[M+NH4]+
	910.7858		4.2820		TG 55:6	C58H100O6	[M+NH4]+
	934.6461	0.0070		7.4895	DGDG 34:2	C49H88O15	[M+NH4]+

	934.7858		6.2046	TG 57:8	C60H100O6	[M+NH4]+
	936.6618	0.0056	5.9787	DGDG 34:1	C49H90O15	[M+NH4]+

Supplementary Table S11: Identified markers for downregulated ion signals in positive-ion mode

MALDI			Previous annotations. now identified			
**Input Mass**	**Matched Mass**	**Delta**	**ppm**	**Annotation**	**Formula**	**Adduct**
440.3132441	440.3135		0.6813	LPE O-16:0	C21H46NO6P	[M+H]+
496.3030180	496.3034	0.0004	0.8060	LPC 15:1;O	C23H46NO8P	[M+H]+
536.3320580	536.3347		4.8477	LPC 18:2;O	C26H50NO8P	[M+H]+
584.5247034	584.5248	0.0001	0.1711	DG 32:1	C35H66O5	[M+NH4]+
664.5274557	664.5276		0.1505	LPC 28:0	C36H74NO7P	[M+H]+
698.5564016	698.5565	0.0001	0.1432	HexCer 34:2;O2	C40H75NO8	[M+H]+
718.5384286	718.5381		0.4175	PC 31:1	C39H76NO8P	[M+H]+
718.5384286	718.5381	0.0003	0.4175	PE 34:1	C39H76NO8P	[M+H]+
748.5484093	748.5487		0.4008	PS O-34:1	C40H78NO9P	[M+H]+
762.5019545	762.5068	0.0049	6.4262	PE 38:7	C43H72NO8P	[M+H]+
774.5625857	774.5643		2.3239	PS O-36:2	C42H80NO9P	[M+H]+
848.6583099	848.6528	0.0055	6.4809	PC O-42:6	C50H90NO7P	[M+H]+
848.6609403	848.6528		9.6624	PC O-42:6	C50H90NO7P	[M+H]+
858.7386898	858.731	0.0077	8.9667	PC O-42:1	C50H100NO7P	[M+H]+
874.5435620	874.544	0.0004	0.4574	PI 36:5	C45H77O13P	[M+NH4]+

Supplementary Table S12: Identified markers for upregulated ion signals in negative-ion mode

MALDI			Previous annotations. now identified			
**Input Mass**	**Matched Mass**	**Delta**	**ppm**	**Annotation**	**Formula**	**Adduct**
	466.2939		1.9301	LPE 17:0	C22H46NO7P	[M-H]-

466.2947810	498.2626	0.0009		1.8063	LPE 20:5	C25H42NO7P	[M-H]-
498.2635084							
	506.3252		2.1725		LPE 20:1	C25H50NO7P	[M-H]-
506.3263029	552.3307	0.0016		2.8968	LPC 17:1	C25H50NO7P	[M+Formate]-
552.3323298							
	566.3464		1.9423		LPC 18:1	C26H52NO7P	[M+Formate]-
566.3474318	590.3464	0.0014		2.3715	LPC 20:3	C28H52NO7P	[M+Formate]-
590.3477523							
	730.5392		3.9697		PE 35:1	C40H78NO8P	[M-H]-
730.5420894	758.5705	0.0028		3.6912	PE 37:1	C42H82NO8P	[M-H]-
758.5733422							
	762.5443		1.7048		PE O-39:6	C44H78NO7P	[M-H]-
762.5430084	764.5447	0.0024		3.1391	PC 31:0	C39H78NO8P	[M+Formate]-
764.5471111							
	809.5186		4.3236		PI 32:0	C41H79O13P	[M-H]-
809.5221022	822.5291	0.0026		3.1610	PC 36:6	C44H76NO8P	[M+Formate]-
822.5264357							
	823.5342		3.1571		PI 33:0	C42H81O13P	[M-H]-
823.5368599	856.6073	0.0022		2.5683	PS 41:2	C47H88NO10P	[M-H]-
856.6050921							
856.6050921	856.6073	0.0022		2.5683	PC 38:3	C46H86NO8P	[M+Formate]-

Supplementary Table S13: Identified markers for downregulated ion signals in negative-ion mode

MALDI				Previous annotations. now identified			
Input Mass	Matched Mass	Delta		ppm	Annotation	Formula	Adduct
	438.299		3.1942	0	LPE O-16:0	C21H46NO6P	[M-H]-
438.3003678							
466.3291093	466.3303	0.0012		2.5733	LPE O-18:0	C23H50NO6P	[M-H]-
	673.4814		3.4151	0	PA 34:1	C37H71O8P	[M-H]-
673.4836850							
732.4849415	732.4821	0.0028		3.8226	PS 32:1	C38H72NO10P	[M-H]-
	734.5342		4.0842	0	PC O-30:1	C38H76NO7P	[M+Formate]-
734.5371260							
748.5237063	748.5287	0.0050		6.6798	PE O-38:6	C43H76NO7P	[M-H]-
	750.5291		3.9972	0	PC 30:0	C38H76NO8P	[M+Formate]-
750.5320532							
758.5023145	758.4978	0.0045		5.9328	PS 34:2	C40H74NO10P	[M-H]-

760.5162567	760.5134		3.6817	PS 34:1	C40H76NO10P	[M-H]-
762.5226475	762.5291	0.0064	8.3931	PC 31:1	C39H76NO8P	[M+Formate]-
776.5018524	776.5083		8.3708	PS 34:1;O	C40H76NO11P	[M-H]-
782.4983903	782.4978	0.0006	0.7668	PS 36:4	C42H74NO10P	[M-H]-
821.5584703	821.5549		4.2602	PI O-34:1	C43H83O12P	[M-H]-
835.5379368	835.5342	0.0037	4.4283	PI 34:1	C43H81O13P	[M-H]-
836.5390702	836.5447		6.6942	PS 40:5	C46H80NO10P	[M-H]-
836.5390702	836.5447	0.0056	6.6942	PC 37:6	C45H78NO8P	[M+Formate]-
863.5704866	863.5655		5.7899	PI 36:1	C45H85O13P	[M-H]-
864.5725694	864.576	0.0034	3.9326	PS 42:5	C48H84NO10P	[M-H]-
864.5725694	864.576		3.9326	PC 39:6	C47H82NO8P	[M+Formate]-
891.5972493	891.5968	0.0004	0.4486	PI 38:1	C47H89O13P	[M-H]-

Supplementary Table S14: Comparison of identified infection markers with literature[26.27]

MALDI	Lipid Maps			MS/MS	Literature
Input Mass	Matchedm Mass	ppm	Annotation	Lipid Match	Species
863.5577271	863.5655	9.0323	PI(36:1)	PI(36:1) as $[M-H]^-$	T. gondii. B. besnoiti. N. caninum
